# Amine synthesis via iron-catalysed reductive coupling of nitroarenes with alkyl halides

**DOI:** 10.1038/ncomms12494

**Published:** 2016-08-12

**Authors:** Chi Wai Cheung, Xile Hu

**Affiliations:** 1 Laboratory of Inorganic Synthesis and Catalysis, Institute of Chemical Sciences and Engineering, Ecole Polytechnique Fédérale de Lausanne (EPFL), ISIC-LSCI, BCH 3305, 1015 Lausanne, Switzerland

## Abstract

(Hetero)Aryl amines, an important class of organic molecules in medicinal chemistry, are most commonly synthesized from anilines, which are in turn synthesized by hydrogenation of nitroarenes. Amine synthesis directly from nitroarenes is attractive due to improved step economy and functional group compatibility. Despite these potential advantages, there is yet no general method for the synthesis of (hetero)aryl amines by carbon–nitrogen cross-coupling of nitroarenes. Here we report the reductive coupling of nitroarenes with alkyl halides to yield (hetero)aryl amines. A simple iron catalyst enables the coupling with numerous primary, secondary and tertiary alkyl halides. Broad scope and high functional group tolerance are demonstrated. Mechanistic study suggests that nitrosoarenes and alkyl radicals are involved as intermediates. This new C–N coupling method provides general and step-economical access to aryl amines.

## Abstract (web summary)

Direct synthesis of aryl amines from nitroarenes avoids the waste and potential functional group incompatibility of first reducing to anilines. Here, the authors report an iron catalysed reductive coupling of nitroarenes with alkyl halides to form a wide range of (hetero)aromatic amines.

## Graphical abstract

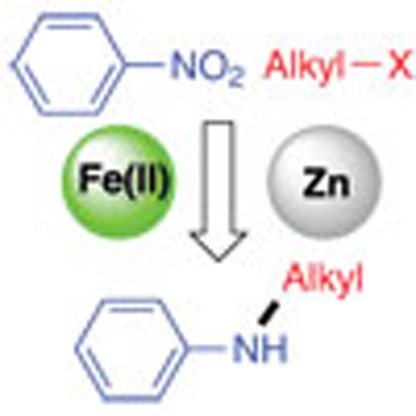

Amines are among the most important organic compounds for the chemical, materials, pharmaceutical and agrochemical industries^1^,^2^,^3^. In particular, aromatic and heteroaromatic amines occupy a privileged position in the medicinal chemistry^3^,^4^, as exemplified in the top-selling drugs such as Abilify, Crestor, Gleevec and Lidoderm^5^. The most general methods to prepare aryl and heteroaryl amines are amine-carbonyl reductive amination^6^ (Fig. 1a), direct alkylation of amines with alkyl halides^7^ (Fig. 1b), and Buchwald–Hartwig^8^,^9^,^10^ and Ullman-type carbon–nitrogen (C–N) cross-coupling reactions^11^,^12^ (Fig. 1c). These amination methods employ amines, especially anilines, as the nitrogen source, which are usually prepared in advance by the hydrogenation of nitroarene derivatives^13^. Amine synthesis using directly nitro(hetero)arenes is attractive as it eliminates the hydrogenation step, saving time and cost. Moreover, functional groups such as amine, hydroxyl and thiol have orthogonal reactivity to the nitro group, so they may be tolerated without protection^14^. On the contrary, in amine synthesis using anilines, these groups have to be protected to avoid undesirable alkylation or arylation^14^. Recently, Baran and co-workers^14^ developed a novel and elegant hydroamination approach to synthesize (hetero)aryl amines from nitro(hetero)arenes and olefins using an iron salt as catalyst, phenylsilane as hydrogen donor, and zinc (Zn) as reductant (Fig. 1d). Nevertheless, this approach was mostly efficient for the synthesis of (hetero)aryl amines substituted by a tertiary alkyl group^15^. General methods for amine synthesis from nitroarenes remain underdeveloped despite their potential advantages^14^,^16^,^17^.

Here, we report (hetero)aryl amine synthesis via iron-catalysed reductive coupling of nitro(hetero)arenes with alkyl halides (Fig. 1e). This single-step cross-coupling approach allows the liaison to various types of carbon groups. Indeed, not only tertiary alkyl halides, but also secondary and primary alkyl halides could be coupled. The method tolerates a large number of functional groups, including those that require protection under conventional amine synthesis.

## Results

### Reaction design

We recently reported Fe-catalysed reductive coupling of terminal aryl alkynes with non-activated alkyl halides to give *cis*-olefins^18^. Mechanistic study suggested that alkyl radical was involved as an intermediate, which added to terminal alkyne to form the C–C bond. Inspired by Baran's proposal that alkyl radical could add to nitrosoarene, formed by the reduction of nitroarene^14^, we envisioned that reductive coupling of nitroarenes and alkyl halides might be used to form secondary alkyl aryl amines according to the following pathways (Fig. 2): an iron(II) precatalyst (Fe(II)) is reduced by zinc to Fe(I) (refs 18, 19, 20; Fig. 2a), which is then able to activate an alkyl halide to give an alkyl radical and regenerate Fe(II) (ref. 18; Fig. 2b). Meanwhile, nitroarene is reduced by zinc to form nitrosoarene^21^,^22^ (Fig. 2c). The alkyl radical attacks the nitrogen atom of nitrosoarene to form the C–N bond^14^, and reduction of the resulting intermediate by zinc in the presence of an oxophilic Lewis acid such as chlorotrimethylsilane (TMSCl) shall give the amine product (Fig. 2c).

### Screening of reaction conditions

We commenced the study by examining the reaction of nitrobenzene (**1a**) with 2-iodooctane (**2a**) (Supplementary Tables 1–9). After a screening of reaction parameters, we found that the optimized conditions involved the use of *N*-methylpyrrolidone (NMP) as solvent, iron(II) chloride tetrahydrate (FeCl_2_·4H_2_O) as catalyst (20 mol%), Zn (3 equiv.) as reductant, and TMSCl (2 equiv.) as co-reductant. The optimal loading of 2-iodooctane was 3 equiv. The reaction completed after 16 h at 90 °C. After an acidic workup, *N*-(2-octyl)aniline (**3a**) was obtained in a nearly quantitative yield without over-alkylation (Supplementary Table 9, entry 1). Among various iron salts, FeCl_2_·4H_2_O was the best catalyst (Supplementary Table 1, entries 1–16). When FeCl_2_·4H_2_O was replaced by catalysts based on copper, cobalt, nickel, manganese, chromium, palladium and silver, the yields were lower (Supplementary Table 1, entries 17–29). When 99.99% pure FeCl_2_·4H_2_O was used, the yield was similar (Supplementary Table 8, entry 3). Without TMSCl but under otherwise optimal conditions, the yield was 73% (Supplementary Table 9, entry 3).

### Substrate scope of secondary and tertiary alkyl halides

The above conditions proved to be general for the coupling of various nitro(hetero)arenes and secondary and tertiary alkyl halides (Fig. 3). The protocol is insensitive to the electronics of nitroarenes, as nitrobenzene (**3a**) and its derivatives containing electron-withdrawing (**3b**-**3e, 3i**–**3p, 3r**) or electron-donating groups (**3f, 3g, 3q, 3s**–**3v, 5b, 5u**) all reacted to yield the corresponding secondary aryl amines in synthetically useful yields. Functional group such as trifluoromethoxy (**3b**), aryl bromo (**3c**), aryl chloro (**3d**), aryl iodo (**3i**), nitrile (**3e**), olefin (**3h**), alkyne (**3n**), ester (**3k, 3l**), keto (**3m**) and thiomethyl (**3o**) were all tolerated. Thanks to the orthogonal reactivity of nitro groups with protic functional groups, hydroxyalkyl (**3f**), amine (**3q**), amide (**3r**), and particularly phenol (**3g**) and thiophenol groups (**5u**), were tolerated without the need of protection. The boronic ester group (**3p**) was compatible to a good degree. Nitroarenes with sterically congested 2,4-xylyl (**3u**), indane (**3v**) and naphthyl (**3w**) groups were also suitable reaction partners. Importantly, nitroheteroarenes could be coupled, giving rise to various heteroaryl amines containing pyrrole (**4a**), pyrazole (**4b**), pyridine (**4c**), quinoline (**4d**), benzothiophene (**4e**), benzothiazole (**4f**), benzoxazole (**4g**), indole (**4h, 4i**), indazole (**4j**) and cuomarin (**4k**) groups. Notably, unprotected indole (**4i**) and indazole (**4j**) were compatible. With respect to the scope of alkyl halides, the reaction protocol allowed the coupling of secondary alkyl iodides with a wide range of acyclic and cyclic substituents, including hydrocarbon groups (**3a**, **3i**-**3k, 5a, 5b, 5g**, **5j, 5m** and **5n**) and functionalized alkyl groups (olefin (**4f**), ether (**5c, 5h, 5k**), amine (**5d**), ester (**5e**), phthalimide (**5f**), carbamate (**5i, 5l**)). Both non-activated (**5o**, **5p**) and activated secondary alkyl bromides (**5q–5t**) reacted to afford the corresponding aryl amines in synthetically useful yields when their loadings were increased to 5 equiv. The introduction of bulky tertiary alkyl groups has been shown to enhance the lipophilicity and metabolic stability of the drugs to improve their potency^23^,^24^. Gratifyingly, tertiary alkyl iodides (**5u**, **5v**) and bromides (**5w**, **5x**) could be coupled using this protocol to give the corresponding *N*-*tert*-alkylated amines, albeit with a higher loadings of alkyl halides (4–5 equiv.). Large-scale synthesis (10 mmol) could be achieved without the significant loss in yield (for example, for **3a**).

### Substrate scope of primary alkyl halides

**Figure 1:**
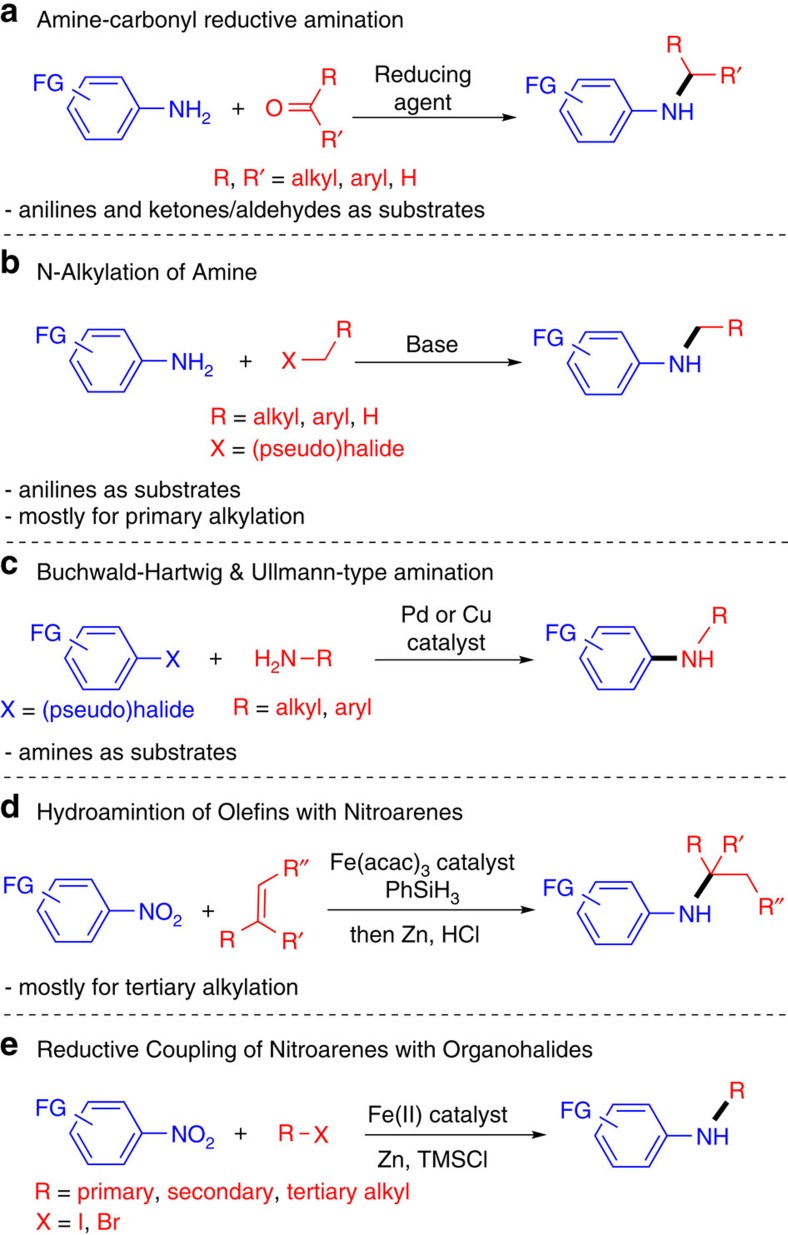
Different approaches to (hetero)aryl amines. (**a**) Reductive amination; (**b**) *N*-Alkylation of amine; (**c**) Buchwald–Hartwig and Ullmann-type coupling; (**d**) Baran's hydroamination with nitro(hetero)arenes; (**e**) Current work using reductive coupling of nitro(hetero)arenes with alkyl halides. Fe(acac)_3_, iron(III) acetylacetonate; PhSiH_3_,phenylsilane; Zn, zinc; TMSCl, chlorotrimethylsilane.

Aryl amines substituted with a primary alkyl group are ubiquitous building blocks for biologically active molecules^15^. The *N*-alkylation of aryl amines with primary alkyl halides is a common method to generate such amines (Fig. 1b), but it has a limited scope and may suffer from over-alkylation (see below)^7^. Thus, we sought to apply this reductive coupling method for the coupling of primary alkyl halides. However, the reaction conditions described above were inefficient for the coupling of primary alkyl iodide, as undesired *N*,*N*-dialkylaniline was formed in large excess relative to the desired *N*-alkylaniline (Supplementary Table 10, entry 1). A re-optimization showed that reduction of primary alkyl iodide to 1.5 equiv., additions of more Zn (5 equiv.) and TMSCl (4 equiv.), and lower concentrations of reactants led to a significant suppression of double alkylation (Supplementary Table 10, entry 8). Under the new conditions, the coupling of **1a** with 1-iodooctane (**2b**) gave *N*-octylaniline (**6a**) and *N*,*N*-dioctylaniline (**7a**) in 76 and 15% GC yields, respectively (compared with 18% **6a** and 78% **7a** under the protocol in Fig. 3) (Supplementary Table 10, entries 1 and 8). The modified protocol was applicable for the coupling of a broad range of primary alkyl iodides and bromides (Fig. 4). Both non-functionalized (**6a**–**6c**, **6r**) and functionalized alkyl halides (**6d**–**6q**) reacted efficiently to give the target secondary amines in synthetically useful yields, with only a small amount of dialkylation products (**7a**–**7r**). Functional groups such as olefin (**6d, 6q**), ether (**6e, 6m**), keto (**6f**), ester (**6g, 6n**), amide (**6h**), nitrile (**6i**), carbazole (**6j**), hydroxyl (**6o**) and chloro moieties (**6p**) were all tolerated. Molecules containing a bioactive moiety such as menthol (**6k**) and cholesterol (**6l**) were compatible as well. The reaction could also be run in a gram scale (10 mmol) with a similar yield (for example, for **6a**).

### Application

To demonstrate its potential utility for medicinal chemistry, this Fe-catalysed amination method was applied for the synthesis of drug-like molecules and their key intermediates. Using this method, *N*-2-butylaniline **3x**, an intermediate to an antiviral agent of human cytomegalovirus (**PD0084430**, **3y**)^25^, was synthesized in a 53% yield (Fig. 5a). Subsequent hydrogenolysis of **3x** provided the target compound in a 45% overall yield in two steps. Previous method using the reductive amination method gave the product in a lower overall yield (19%)^25^. The amination protocol was also successfully applied for the synthesis of antifungal agents, **5y** and **5z** (ref. 26), giving the product in a single step using commercially available nitroarenes and stable alkyl bromides (Fig. 5b). For comparison, the previous reductive amination method required a two-step sequence and involved sensitive Grignard reagents^26^.

### Comparison with direct alkylation and reductive amination

Compared with established amination methods such as direct alkylation and reductive amination, the reductive coupling method described here employs a different set of starting materials, which should be advantageous for the synthesis of certain compounds that are difficult using the former methods. This reasoning is supported by examples in Fig. 6 and Supplementary Figs 1–4 (refs 27, 28, 29, 30). The direct alkylation of aniline derivative with secondary alkyl halides^28^ normally has much lower yields than the reductive coupling (Fig. 6a,b; Supplementary Fig. 1). Even direct alkylation with a primary alkyl halide^27^ might have a much lower yield than the reductive coupling (Fig. 6c; Supplementary Fig. 2). If an alkyl halide has a functional group susceptible to nucleophilic attack of aniline, for example, 1-bromo-6-chlorohexane, only undesired double alkylation product^31^, but not the desired selective alkylation product, was obtained in 31% yield (Fig. 6d). On the contrary, the reductive coupling method gave the desired mono-alkylation product in 52% yield (Fig. 6d). Reductive amination using stoichiometric amounts of sodium triacetoxyborohydride (NaBH(OAc)_3_) proves to be a group-tolerant method for amine synthesis^30^. However, when the ketone or aldehyde reagents contain a keto or alkyl halide functional group, the amination is unsuccessful (Fig. 6e,f). On the contrary, the reductive coupling gave the desired amines in synthetically useful yields (Fig. 6e,f).

### Mechanistic investigation

A number of experiments were conducted to give some insights into the mechanism. In principle, a nitroarene can be reduced by zinc to form an aniline^32^,^33^, which can then undergoes *N*-alkylation with an alkyl halide to form the alkyl aryl amine product^7^. Indeed, it was found that in the absence of an alkyl halide, 4-nitrotoluene was reduced under the coupling conditions to form 4-methylaniline, albeit in a modest yield (Fig. 7a). However, 4-methylaniline did not react with 2-octyl iodide under the coupling conditions to yield the amine product (Fig. 7a). Furthermore, under the coupling conditions, the reactions of secondary alkyl iodides with various anilines gave the alkyl amine only in low conversion and yields, while the reductive coupling of nitroarenes with secondary alkyl iodides was efficient (Supplementary Fig. 5). Thus, direct alkylation of *in situ* formed aniline can be ruled out as the main reaction pathway.

**Figure 2:**
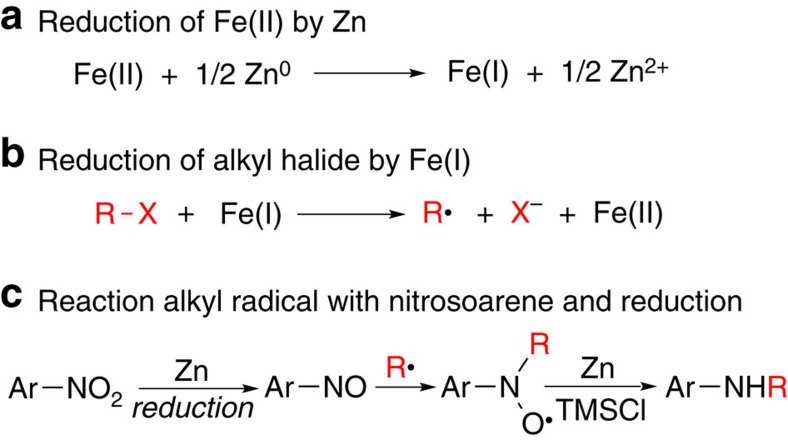
Mechanistic design for Fe-catalysed reductive coupling of nitroarene with alkyl halide. (**a**) Reduction of iron(II) to iron(I); (**b**) activation of alkyl halide by iron(I); (**c**) reaction of alkyl radical with nitrosoarene and subsequent reduction to give alkyl aryl amine. ArNO_2_, nitroarene; RX, alkyl halide; TMSCl, chlorotrimethylsilane.

Another possible reaction pathway involves the reduction of nitrobenzene by zinc and TMSCl to form nitrosoarene, which subsequently reacts with an alkyl radical generated via iron-mediated reduction of alkyl halide to form the alkyl aryl amine, as described in our initial reaction design (Fig. 2). It was found that 4-methylnitrosobenzene reacted with 2-octyl iodide under the coupling conditions to give the amine product in a 70% yield, while the parent reaction using 4-nitrotoluene had a yield of 93% (Fig. 7b). The yields are comparable considering the difference in reagents used. When 2-vinylnitrobenzene was used as a substrate, the expected coupling product (**3z**) was obtained at the same time as an isoxazole co-product (**3z′**)^34^,^35^ (Fig. 7c). The formation of **3z′** suggests that 2-vinylnitrosobenzene (**8a**) is generated in the course of reaction. It is proposed that the alkyl radical attacks the nitrogen atom of **8a** to form **8b**, which undergoes cyclization and hydrogen atom abstraction to give **3z′**. Thus, the above data supports the intermediacy of nitrosoarene as a viable intermediate in the reactions.

To further support the involvement of alkyl radicals^36^, 2,2,6,6-tetramethyl-1-piperidinyloxy (TEMPO) was used as a radical trap for the coupling of nitrobenzene with 2-octyl iodide. A significant diminishment in the yield of amination was observed (Supplementary Fig. 6a). This result is consistent with the involvement of radicals. When cyclopropylmethyl bromide was used as a radical-clock substrate, both the conventional product (**6s**) and the ring-opened product (**6s′**) were obtained (Fig. 7d). This result is again consistent with the formation of alkyl radicals in the coupling reactions.

To probe whether alkyl zinc reagent was formed and acted as an intermediate, 2-octyl zinc iodide^37^ was treated with nitrobenzene under the coupling conditions, but only a trace of amine product was formed (Supplementary Fig. 6b). This result ruled out the intermediacy of alkyl zinc reagents.

## Discussion

**Figure 3:**
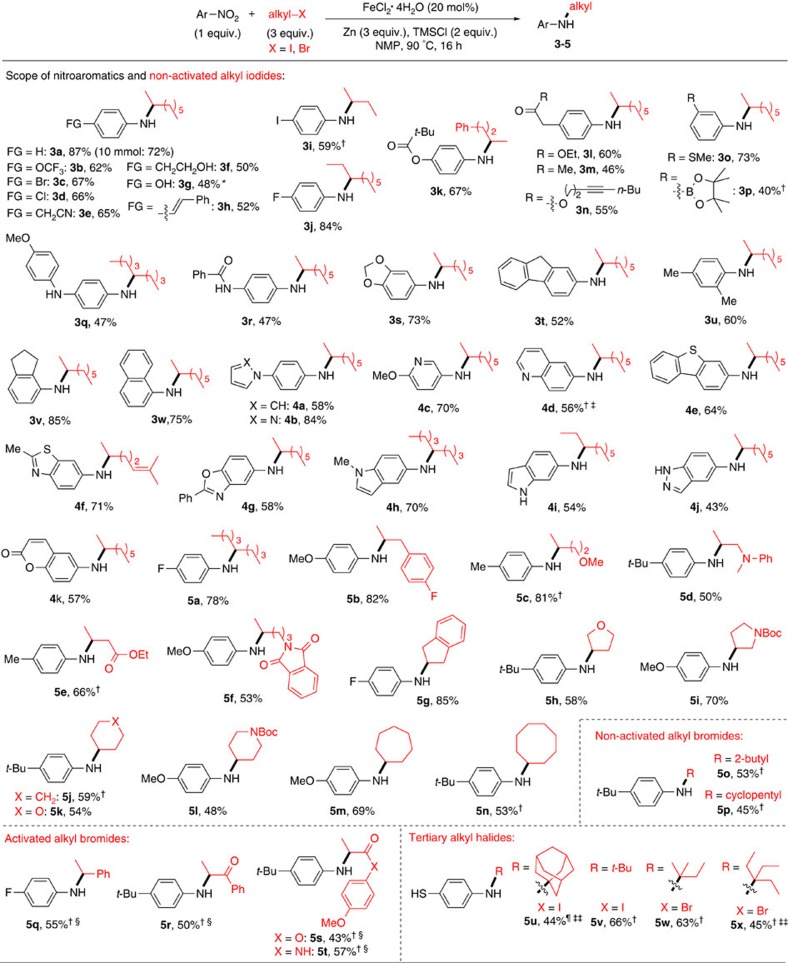
Amine synthesis via reductive coupling of nitroarenes with secondary and tertiary alkyl halides. Unless otherwise noted, the optimized conditions were applied; the data are reported as isolated yield. See Supplementary Materials for experimental details. Boc, *tert*-butyloxycarbonyl; Bu, butyl; Et, ethyl; Me, methyl; Ph, phenyl; *t*-Bu, *tert*-butyl. *RI (2 equiv.); ^†^RI (5 equiv.); ^‡^FeCl_2_·4H_2_O (40 mol%), Zn (3.5 equiv.); ^§^FeCl_2_·4H_2_O (30 mol%), Zn (3.5 equiv); ^¶^RI (4 equiv.); ^‡‡^FeCl_2_·4H_2_O (30 mol %), Zn (4 equiv.).

**Figure 4:**
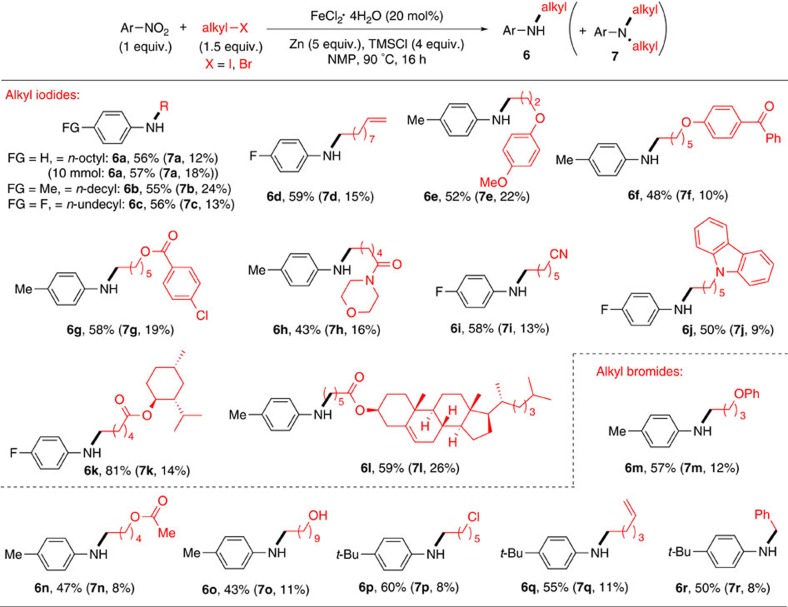
Amine synthesis via reductive coupling of primary alkyl halides. Unless otherwise noted, the optimized conditions were applied; the data are reported as isolated yields. See Supplementary Materials for experimental details. Me, methyl; Ph, phenyl.

**Figure 5:**
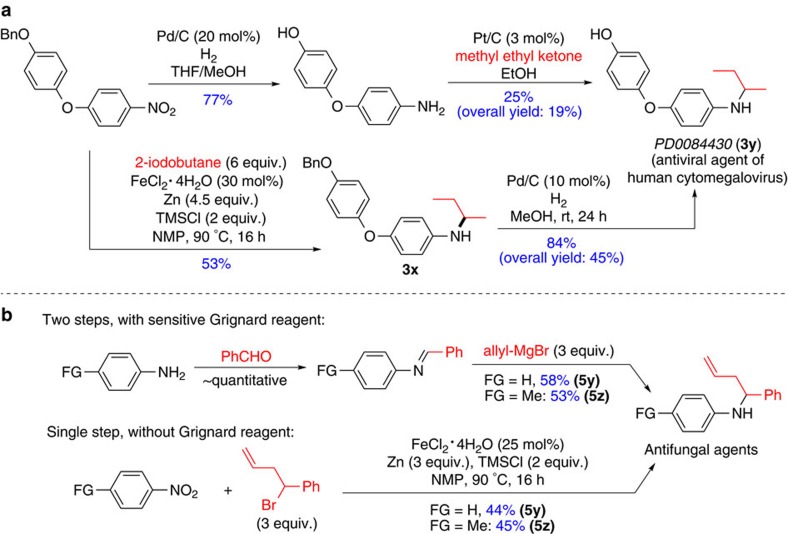
Application in medicinal chemistry. (**a**) Efficient synthesis of the key intermediate of an antiviral agent. (**b**) Single-step synthesis of antifungal agents. The data are reported as isolated yield. Bn, benzyl; Ph, phenyl.

**Figure 6:**
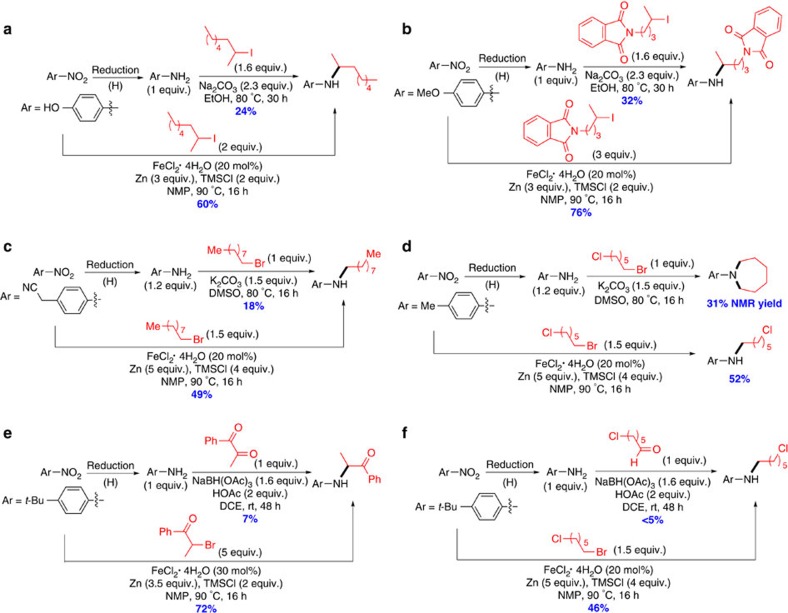
Comparison of iron-catalysed reductive coupling method and classical amination methods. (**a**,**b**) Comparison with direct alkylation of anilines using secondary alkyl halides. (**c**,**d**) Comparison with direct alkylation of anilines using primary alkyl halides. (**e**,**f**) Comparison with reductive alkylation. Without otherwise noted, GC yields were reported. DCE, 1,2-dichloroethane; DMSO, dimethyl sulfoxide; EtOH, ethanol; HOAc, acetic acid; Me, methyl; Ph, phenyl; *t*-Bu, *tert*-butyl.

**Figure 7:**
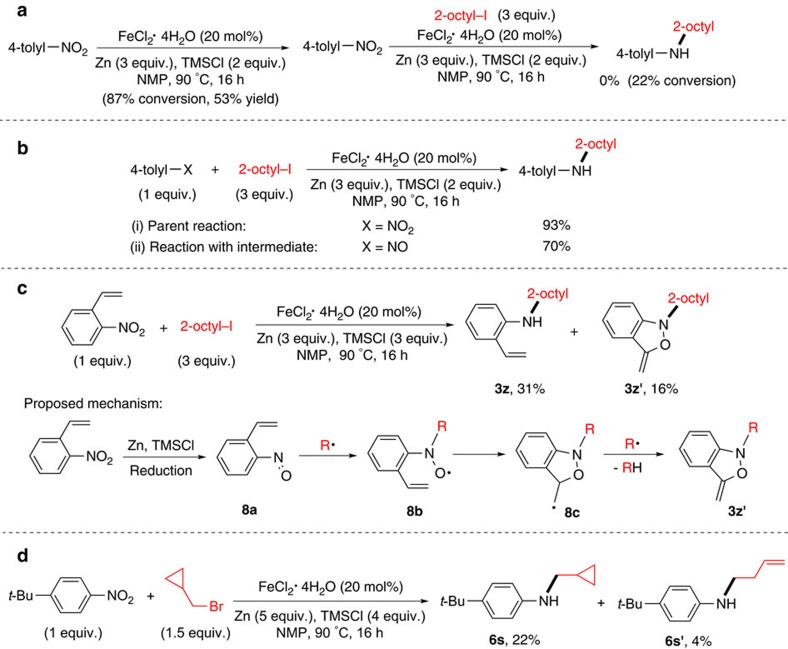
Mechanistic study. (**a**) Reaction of aniline with alkyl halide under the coupling conditions. (**b**) Comparison of amination reactions using nitroarene and nitrosoarene. (**c**) Trapping of nitrosoarene intermediate using 2-vinylnitrobenzene as the substrate. (**d**) Radical-clock experiment using cyclopropylmethyl bromide as the substrate. GC yields were shown in **a** and **b**, and isolated yields were shown in **c** and **d**. *t*-Bu, *tert*-butyl.

The present method allows the synthesis of alkyl aryl amines from functionalized nitroarenes, which are often cheaper than functionalized anilines. Moreover, anilines are generally synthesized by reduction of nitroarenes, so amination using directly nitroarenes is more step-economical than using anilines. Additionally, anilines containing easily reducible groups such as alkenes, ketones, and so on would be difficult to obtain by reduction, while the corresponding nitroarenes are readily available and suitable substrates for the current amination (Fig. 3). From these points of view only, the current method can be already considered as a valuable alternative to the conventional amination methods such as direct alkylation and reductive amination. Furthermore, the data in Figs 3, 4 and 6, and Supplementary Figs 1–4 also demonstrate potential advantages of the current method in terms of scope, group tolerance, and yield in certain reactions: (i) Our method is applicable for the synthesis of aryl amines substituted with a tertiary alkyl group, of which direct alkylation and reductive amination are incapable (Supplementary Fig. 3). (ii) Direct alkylation using a secondary alkyl halide often has a lower yield than the current method (Fig. 6a,b; Supplementary Fig. 1). (iii) Direct alkylation using a primary alkyl halide is intolerant to a functional group prone to S_N_2 substitution, which is not a problem for the current method as it involves radical alkylation (Fig. 6d). (iv) Reductive amination cannot tolerate a keto group or a leaving group prone to S_N_2 substitution by aniline, while the current method tolerates well these groups (Fig. 6e,f). All in all, our results prove that, despite its early stage of development, the present method exhibits certain potential advantages over the well-established amination methods.

The preliminary mechanistic study rules out direct alkylation of *in situ* formed anilines as a major reaction pathway. The data suggest that alkyl radicals and nitrosoarenes are viable intermediates. The proposed mechanism starts with the reduction of Fe(II) catalyst by Zn to form Fe(I), which activates an alkyl halide to give an alkyl radical. In parallel, Zn reduces nitroarenes to give nitrosoarenes, which is attacked by the alkyl radical to form the C–N bond. The resulting species is then further reduced by Zn and deoxygenated by TMSCl to give the secondary alkyl aryl amine. Consistent with this mechanism, when nitroalkanes were used as substrates, the conversion was full but no amination product was isolated. It is likely that the nitrosoalkane intermediates are less stable than their aryl counterparts and undergo isomerization to give oximes^38^,^39^,^40^, which rapidly dissociate into aldehydes/ketones and hydroxylamine without further reacting with alkyl radical. This mechanism is analogous with the mechanism proposed for the reductive coupling of nitroarenes^14^,^15^,^16^,^17^ and nitrite salts^41^ with alkenes, which also involves nitroso intermediates.

In conclusion, the Fe-catalysed reductive coupling methods presented here represented a general approach to synthesize various (hetero)aryl amines from nitro(hetero)arenes. The ability to couple primary, secondary and tertiary alkyl halides and the high functional group tolerance of the method shall attract applications. Mechanistic study suggests that the addition of an alkyl radical to nitrosoarene is the main reaction pathway.

## Methods

### General

Supplementary Figs 7–104 for the NMR spectra, Supplementary Tables 1–10 for the optimization of reactions and Supplementary Methods for the characterization data can be found in the Supplementary Information.

### General procedure for branched alkyl aryl amine synthesis

In a nitrogen-filled glove-box, an oven-dried 30 ml re-sealable screw-cap test tube equipped with a Teflon-coated magnetic stir bar was sequentially charged with zinc powder (Zn, 3 equiv. 1.5 mmol), iron(II) chloride tetrahydrate (20 mol%, 0.10 mmol, 20 mg), nitroarene (1 equiv., 0.50 mmol), alkyl iodide (3 equiv., 1.5 mmol), *N*-methylpyrrolidone solvent (1.0 ml), and chlorotrimethylsilane (3 equiv., 1.5 mmol). The resulting mixture was stirred at 90 °C in a preheated oil bath for 16 h. After the reaction, the reaction mixture was cooled down to room temperature, and the crude product was acidified with saturated NH_4_Cl solution and then neutralized with saturated NaHCO_3_ solution. The crude product in the aqueous fraction was extracted with ethyl acetate. The combined organic fractions were concentrated *in vacuo* with the aid of a rotary evaporator. The crude product residue was purified by flash column chromatography with silica gel using a solvent mixture (ethyl acetate, hexanes) as an eluent to afford the purified amine products (**3**–**5**).

### General procedure for linear alkyl aryl amine synthesis

An oven-dried 30 ml re-sealable screw-cap test tube equipped with a Teflon-coated magnetic stir bar was sequentially charged with zinc powder (5 equiv., 2.5 mmol, 164 mg), iron(II) chloride tetrahydrate (20 mol%, 0.10 mmol, 20 mg), nitroarene (1 equiv., 0.50 mmol), primary alkyl iodide (1.5 equiv., 0.75 mmol), *N*-methylpyrrolidone solvent (2.0 ml), and chlorotrimethylsilane (4 equiv., 2.0 mmol, 128 μl). The resulting mixture was stirred at 90 °C in a preheated oil bath for 16 h. After the reaction, the reaction mixture was cooled down to room temperature, and the crude product was acidified with saturated NH_4_Cl solution and then neutralized with saturated NaHCO_3_ solution. The crude product in the aqueous fraction was extracted with ethyl acetate. The combined organic fractions were concentrated *in vacuo* with the aid of a rotary evaporator. The crude product residue was purified by preparative thin layer chromatography using a solvent mixture (ethyl acetate, hexanes) as an eluent to afford the purified mono-alkylated (**6**) and di-alkylated anilines (**7**).

### Data Availability

The authors declare that the data supporting the findings of this study are available within the article and its Supplementary Information files.

## Additional information

**How to cite this article**: Cheung, C. W. & Hu, X. Amine synthesis via iron-catalysed reductive coupling of nitroarenes with alkyl halides. *Nat. Commun.* 7:12494 doi: 10.1038/ncomms12494 (2016).

## Supplementary Material

Supplementary Information Supplementary Figures 1-104, Supplementary Tables 1-10, Supplementary Methods and Supplementary References

## References

[b1] Lawrence S. A. Amines: Synthesis Properties and Applications Cambridge University Press (2004).

[b2] Ricci A. (ed.) Amino Group Chemistry: from Synthesis to the Life Sciences Wiley-VCH, Weinheim (2008).

[b3] Roughley S. D. & Jordan A. M. The medicinal chemist's toolbox: an analysis of reactions used in the pursuit of drug candidates. J. Med. Chem. 54, 3451–3479 (2011). 21504168 10.1021/jm200187y

[b4] Magano J. & Dunetz J. R. Large-scale applications of transition metal-catalyzed couplings for the synthesis of pharmaceuticals. Chem. Rev. 111, 2177–2250 (2011). 21391570 10.1021/cr100346g

[b5] Bartholow M. Top 200 Drugs of 2012. http://www.pharmacytimes.com/publications/issue/2013/July2013/Top-200-Drugs-of-2012 (2013).

[b6] Baxter E. W. & Reitz A. B. Reductive aminations of carbonyl compounds with borohydride and borane reducing agents. Org. Reactions 59, 1–714 (2004).

[b7] Salvatore R. N., Yoon C. H. & Jung K. W. Synthesis of secondary amines. Tetrahedron 57, 7785–7811 (2001).

[b8] Surry D. S. & Buchwald S. L. Biaryl phosphane ligands in palladium-catalyzed amination. Angew. Chem. Int. Ed. Engl. 47, 6338–6361 (2008). 18663711 10.1002/anie.200800497 PMC3517088

[b9] Hartwig J. F. Evolution of a fourth generation catalyst for the amination and thioetherification of aryl halides. Acc. Chem. Res. 41, 1534–1544 (2008). 18681463 10.1021/ar800098p PMC2819174

[b10] Surry D. S. & Buchwald S. L. Dialkylbiaryl phosphines in Pd-catalyzed amination: a user's guide. Chem. Sci. 2, 27–50 (2011). 22432049 10.1039/C0SC00331J PMC3306613

[b11] Ley S. V. & Thomas A. W. Modern synthetic methods for copper-mediated C(aryl)-O, C(aryl)-N, and C(aryl)-S bond formation. Angew. Chem. Int. Ed. Engl. 42, 5400–5449 (2003). 14618572 10.1002/anie.200300594

[b12] Surry D. S. & Buchwald S. L. Diamine ligands in copper-catalyzed reactions. Chem. Sci. 1, 13–31 (2010). 22384310 10.1039/C0SC00107D PMC3289286

[b13] Kahl T. . Ullmann's Encyclopedia of Industrial Chemistry Wiley-VCH (2000) doi:10.1002/14356007a02_303.pub2.

[b14] Gui J. . Practical olefin hydroamination with nitroarenes. Science 348, 886–891 (2015). 25999503 10.1126/science.aab0245

[b15] Villa M. & von Wangelin A. J. Hydroaminations of alkenes: a radical, revised, and expanded version. Angew. Chem. Int. Ed. Engl. 54, 11906–11908 (2015). 26358866 10.1002/anie.201506885

[b16] Zhu K., Shaver M. P. & Thomas S. P. Chemoselective nitro reduction and hydroamination using a single iron catalyst. Chem. Sci. 7, 3031–3035 (2016). 10.1039/c5sc04471e PMC6005157 29997793

[b17] Zhu K., Shaver M. P. & Thomas S. P. Amine-bis(phenolate) iron(III)-catalyzed formal hydroamination of olefins. Chem. Asian J. 11, 977–980 (2016). 26864731 10.1002/asia.201501098

[b18] Cheung C. W., Zhurkin F. E. & Hu X. *Z*-Selective olefin synthesis via iron-catalyzed reductive coupling of alkyl halides with terminal arylalkynes. J. Am. Chem. Soc. 137, 4932–4935 (2015). 25831473 10.1021/jacs.5b01784 PMC4415033

[b19] Fürstner A. . Preparation, structure, and reactivity of nonstabilized organoiron compounds. Implications for iron-catalyzed cross coupling reactions. J. Am. Chem. Soc. 130, 8773–8787 (2008). 18597432 10.1021/ja801466t

[b20] Adams C. J. . Iron(I) in Negishi cross-coupling reactions. J. Am. Chem. Soc. 134, 10333–10336 (2012). 22694754 10.1021/ja303250t

[b21] Barrow F. & Thorneycroft F. J. *N*-Oximino-ethers. Part V. Stereoisomeric *N*-aryl ethers of oximinophenylacetonitrile. J. Chem. Soc. 773–777 (1939).

[b22] Shine H. J., Zmuda H., Kwart H., Horgan A. G. & Brechbiel M. Benzidine rearrangements. 17. The concerted nature of the one-proton *p*-semidine rearrangement of 4-methoxyhydrazobenzene. J. Am. Chem. Soc. 104, 5181–5184 (1982).

[b23] Ruiz-Castillo P., Blackmond D. G. & Buchwald S. L. Rational ligand design for the arylation of hindered primary amines guided by reaction progress kinetic analysis. J. Am. Chem. Soc. 137, 3085–3092 (2015). 25651374 10.1021/ja512903g PMC4379963

[b24] Wanka L., Iqbal K. & Schreiner P. R. The lipophilic bullet hits the targets: medicinal chemistry of adamantane derivatives. Chem. Rev. 113, 3516–3604 (2013). 23432396 10.1021/cr100264t PMC3650105

[b25] Meyer A. L. . PD0084430: a non-nucleoside inhibitor of human cytomegalovirus replication *in vitro*. Antiviral Res. 52, 289–300 (2001). 11675146 10.1016/s0166-3542(01)00170-x

[b26] Urbina J. M. . Inhibitors of the fungal cell wall. Synthesis of 4-aryl-4-*N*-arylamine-1-butenes and related compounds with inhibitory activities on *β*(1–3) glucan and chitin synthases. Bioorg. Med. Chem. 8, 691–698 (2000). 10819157 10.1016/s0968-0896(00)00003-1

[b27] Srivastava S. K., Chauhan P. M. S. & Bhaduri A. P. A novel strategy for *N*-alkylation of primary amines. Synth. Commun. 29, 2085–2091 (1999).

[b28] Kivala M. . Two dimensional acetylenic scaffolding: extended donor-substituted perethynylated dehydroannulenes. Chem. Asian J. 1, 479–489 (2006). 17441085 10.1002/asia.200600131

[b29] Zachariasse K. A., Druzhinin S. I., Bosch W. & Machinek R. Intramolecular charge transfer with the planarized 4-aminobenzonitrile 1-*tert*-butyl-6-cyano-1,2,3,4-tetrahydroquinoline (NTC6). J. Am. Chem. Soc. 126, 1705–1715 (2004). 14871101 10.1021/ja037544w

[b30] Abdel-Magid A. F., Carson K. G., Harris B. D., Maryanoff C. A. & Shah R. D. Reductive amination of aldehydes and ketones with sodium triacetoxyborohydride. Studies on direct and indirect reductive amination procedures. J. Org. Chem. 61, 3849–3862 (1996). 11667239 10.1021/jo960057x

[b31] Ju Y. & Varma R. S. An efficient and simple aqueous *N*-heterocyclization of aniline derivatives: microwave-assisted synthesis of *N*-aryl azacycloalkanes. Org. Lett. 7, 2409–2411 (2005). 15932210 10.1021/ol050683t

[b32] Desai D. G., Swami S. S. & Hapase S. B. Rapid and inexpensive method for reduction of nitrobenzenes to anilines. Synth. Commun. 29, 1033–1036 (1999).

[b33] Mahdavi H. & Tamami B. Reduction of nitro-aryl compounds with zinc in the presence of poly[*N*-(2-aminoethyl)acrylamido]trimethylammonium chloride as a phase-transfer catalyst. Synth. Commun. 35, 1121–1127 (2005).

[b34] Haley N. F. 3-Methyl-2,1-benzisoxazolium, benzisothiazolium, and indazolium salts as new active-methyl compounds. J. Org. Chem. 43, 1233–1237 (1978).

[b35] Vander Meer R. K. & Olofson R. A. Reactions of anthranilium salts with nucleophiles: adduct formation and rearrangement. J. Org. Chem. 49, 3373–3377 (1984).

[b36] Bauer I. & Knölker H.-J. Iron catalysis in organic synthesis. Chem. Rev. 115, 3170–3387 (2015). 25751710 10.1021/cr500425u

[b37] Huo S. Highly efficient, general procedure for the preparation of alkylzinc reagents from unactivated alkyl bromides and chlorides. Org. Lett. 5, 423–425 (2003). 12583734 10.1021/ol0272693

[b38] Johnson K. & Degering E. F. The utilization of aliphatic nitro compounds. (I) The production of amines and (II) The production of oximes. J. Am. Chem. Soc. 61, 3194–3195 (1939).

[b39] Gilbert K. E. & Borden W. T. Peracid oxidation of aliphatic amines: general synthesis of nitroalkanes. J. Org. Chem. 44, 659–661 (1979).

[b40] Ohe K. & Uemura S. Sodium arenetellurolate-catalyzed reduction of nitroalkanes to oximes. Heteroatom Chem. 2, 507–511 (1991).

[b41] Leggans E. K., Barker T. J., Duncan K. K. & Boger D. L. Iron(III)/NaBH_4_-mediated additions to unactivated alkenes: synthesis of novel 200'-vinblastine analogues. Org. Lett. 14, 1428–1431 (2012). 22369097 10.1021/ol300173v PMC3306530

