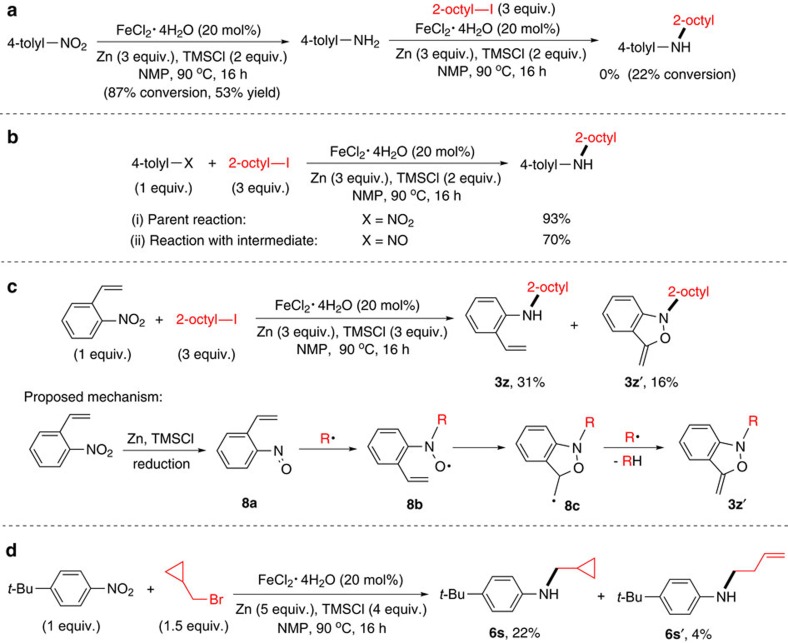# Erratum: Amine synthesis via iron-catalysed reductive coupling of nitroarenes with alkyl halides

**DOI:** 10.1038/ncomms13776

**Published:** 2016-12-06

**Authors:** Chi Wai Cheung, Xile Hu

Nature Communications 7: Article number: 12494; DOI: 10.1038/ncomms12494 (2016); Published 08 12 2016; Updated 12 06 2016

In Fig. 7a of this article, the structure of the intermediate compound was inadvertently misdrawn during the production process. The correct version of Fig. 7 appears below as Fig. 1.

## Figures and Tables

**Figure 1:**