# Supplementary material for: Amine synthesis via iron-catalysed reductive coupling of nitroarenes with alkyl halides
Source: Nat Commun. 2016 Aug 12;7:12494. doi: 10.1038/ncomms12494 (PMC4990657; doi:10.1038/ncomms12494)
Supplement: Supplementary Information — Supplementary Figures 1-104, Supplementary Tables 1-10, Supplementary Methods and Supplementary References [file ncomms12494-s1.pdf]

Supplementary Figures

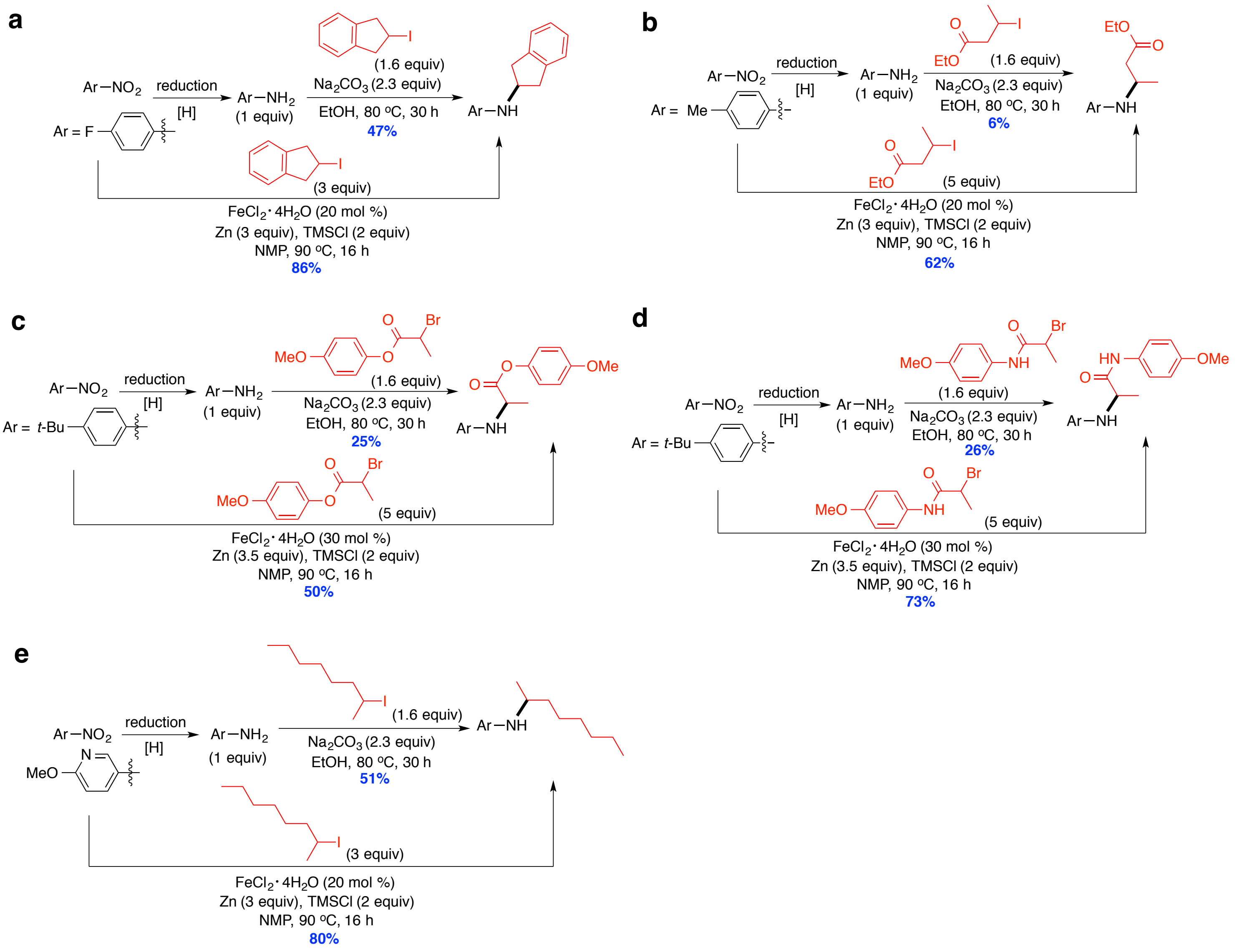

Supplementary Figures 1(a)-(e). Comparison between iron-catalyzed reductive coupling method and *N*-alkylation method in the reactions with secondary alkyl halides. GC yields were shown.

a

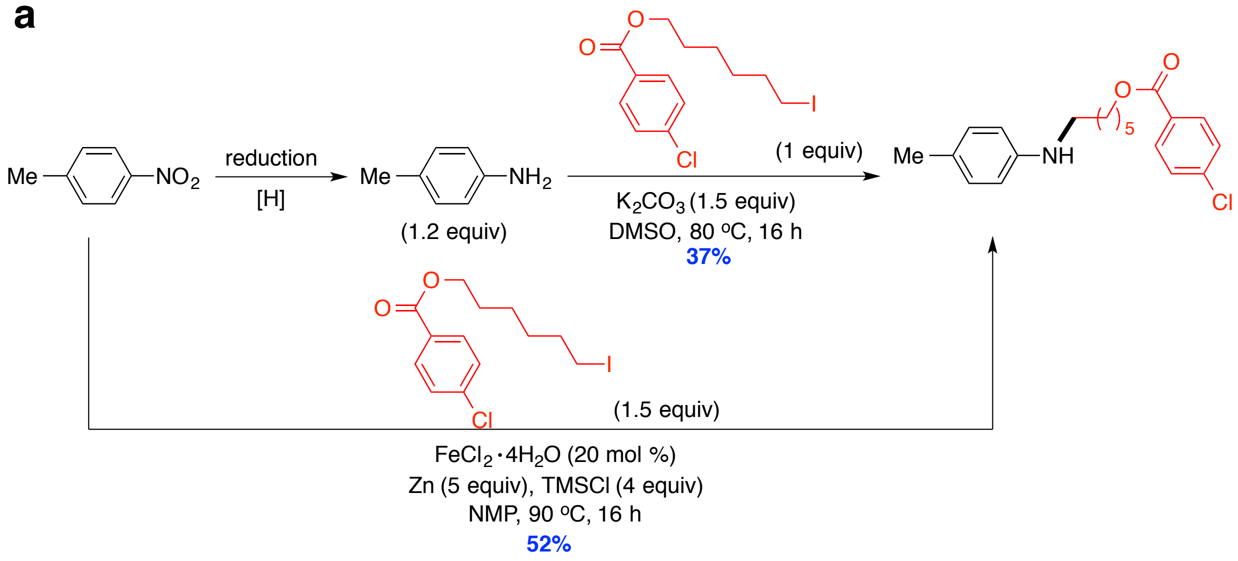

b

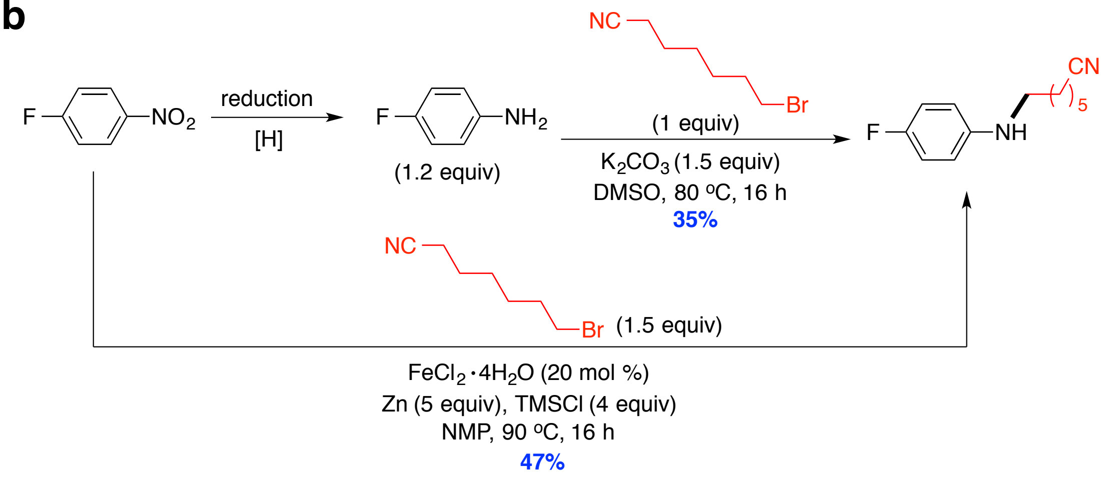

Supplementary Figures 2(a) and (b). Comparison between iron-catalyzed reductive coupling method and *N*-alkylation method in the reactions with primary alkyl halides. GC yields were shown.

a

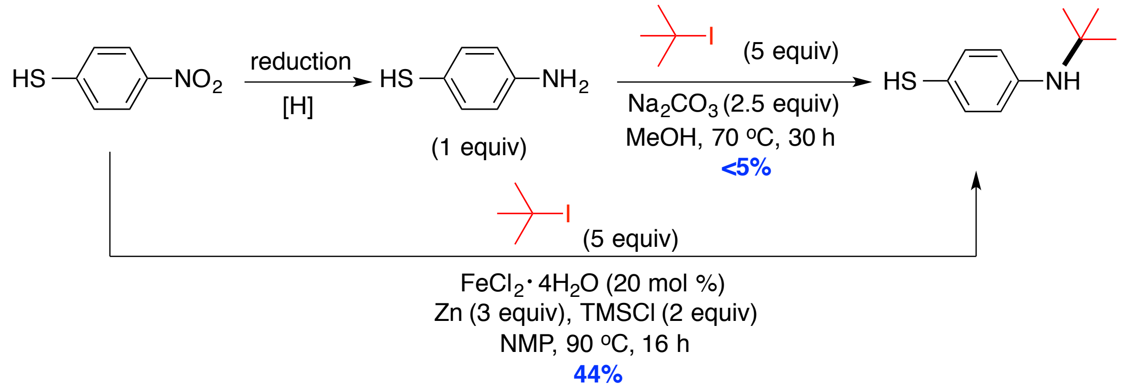

b

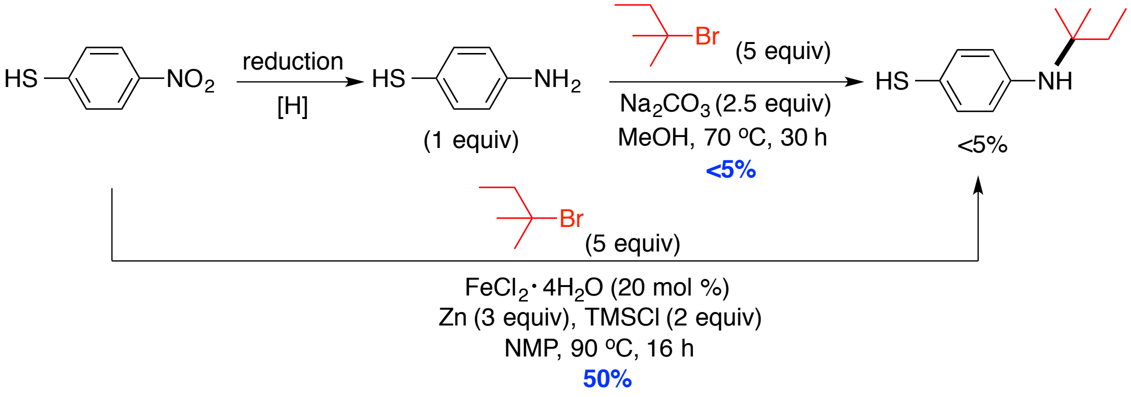

c

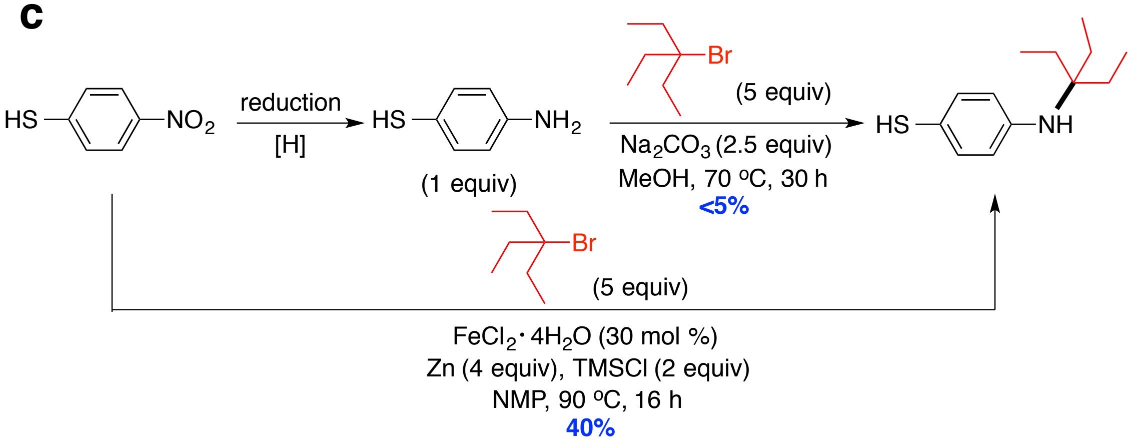

Supplementary Figures 3(a)-(c). Comparison between iron-catalyzed reductive coupling method and *N*-alkylation method in the reactions with tertiary alkyl halides. GC yields were shown.

a

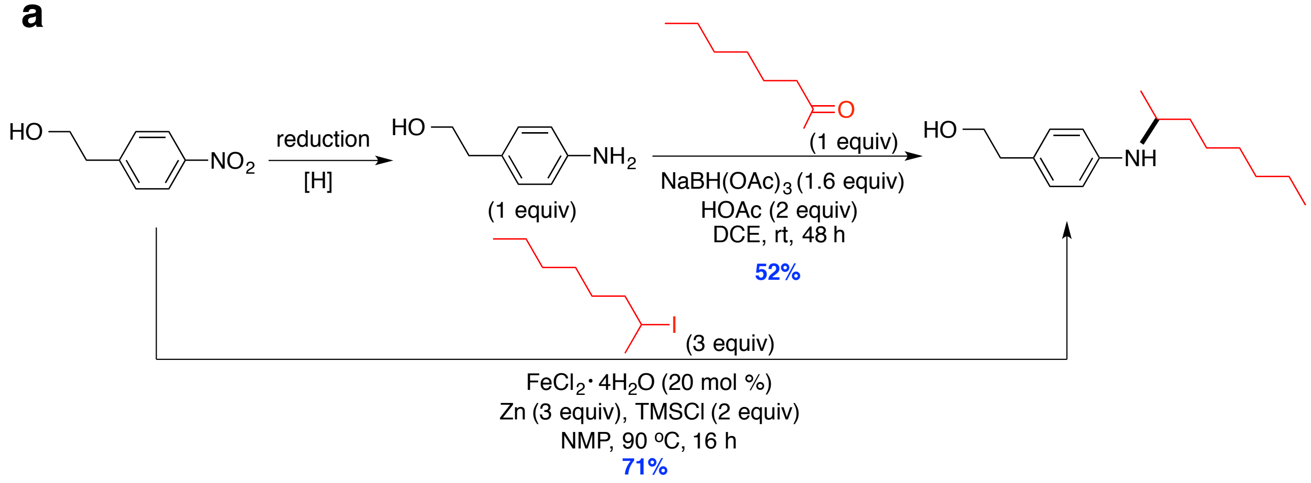

b

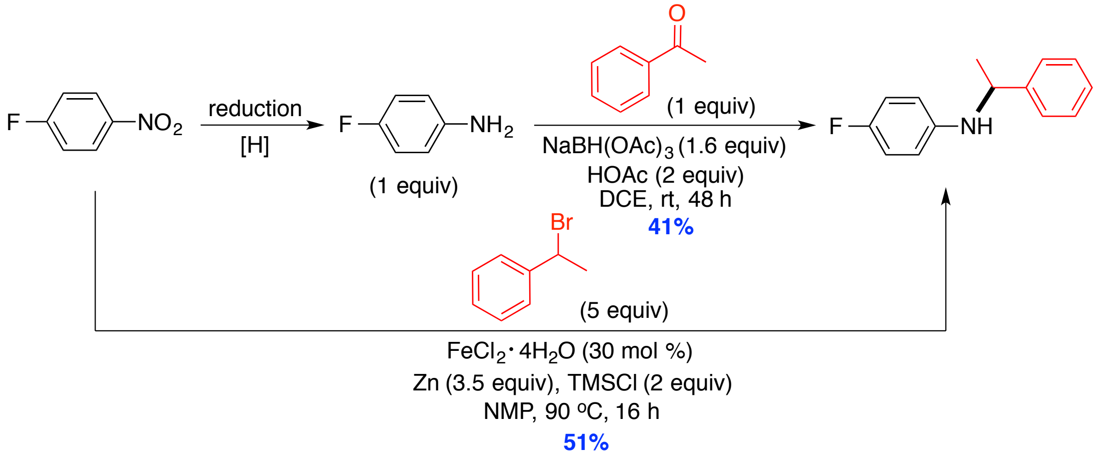

Supplementary Figures 4(a) and (b). Comparison between iron-catalyzed reductive coupling method and reductive amination method. GC yields were shown.

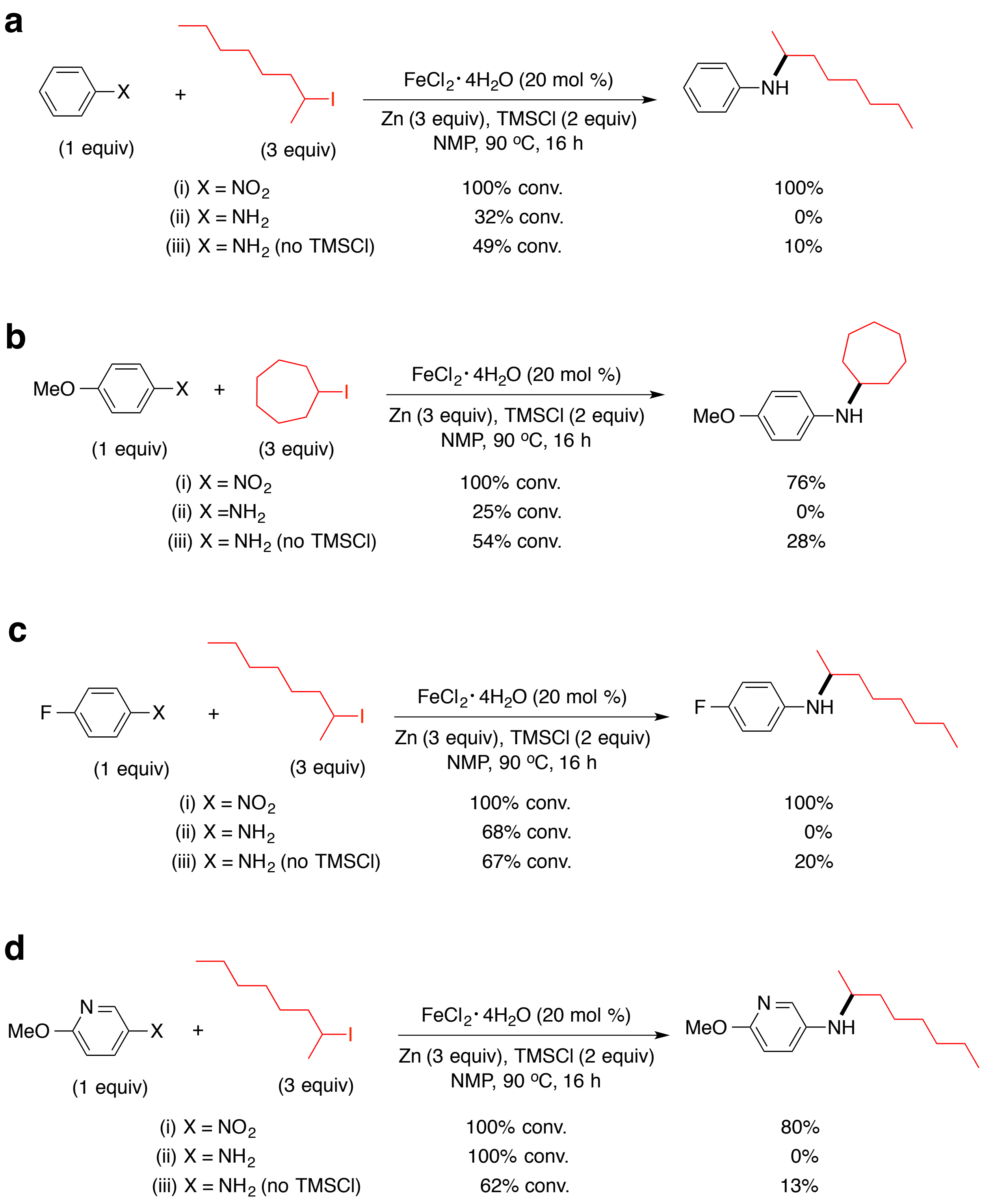

Supplementary Figure 5. Comparisons of the reactivity between nitroarenes and anilines under the Fe-catalyzed reductive coupling conditions. (a) Using electron-neutral aryl groups. (b) Using electron-rich aryl groups. (c) Using electron-deficient aryl groups. (d) Using heteroaryl groups. GC yields were shown.

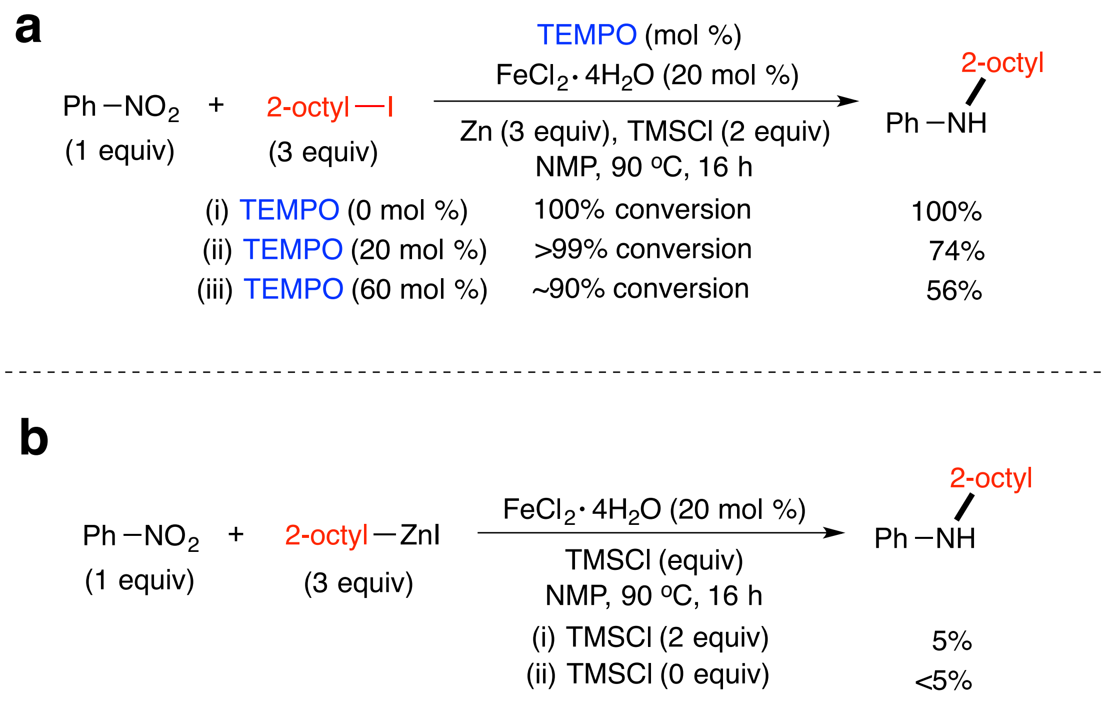

Supplementary Figure 6. To probe the reaction mechanisms via the radical pathways or the intermediacy of alkyl zinc reagent. (a) Effect of TEMPO as radical trap. (b) Reaction using alkyl zinc reagent instead of alkyl iodide/zinc. GC yields were shown.

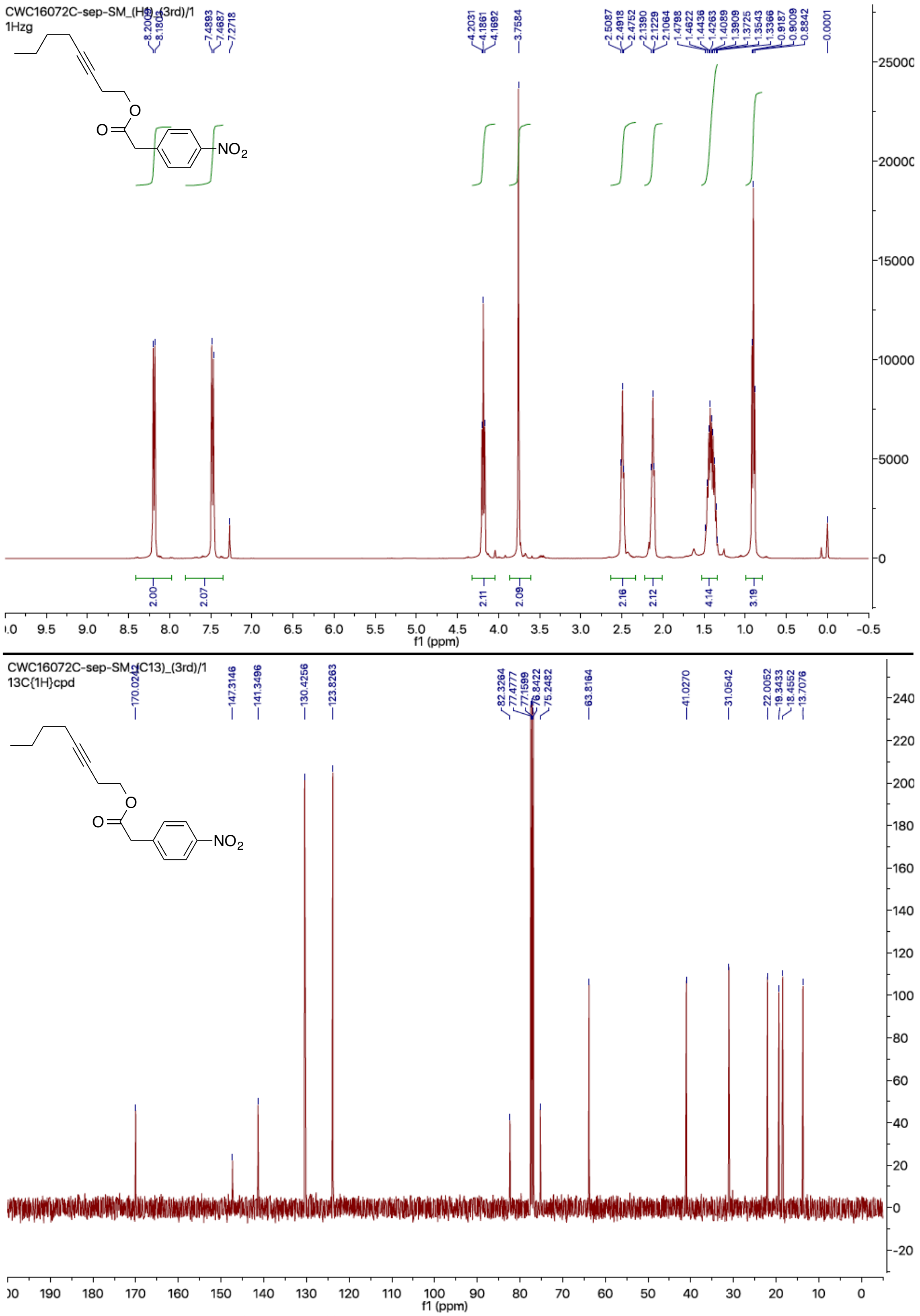

Supplementary Figure 7. **^{1}H and ^{13}C NMR spectra of Oct-3-yn-1-yl 2-(4-nitrophenyl)acetate (S1)**

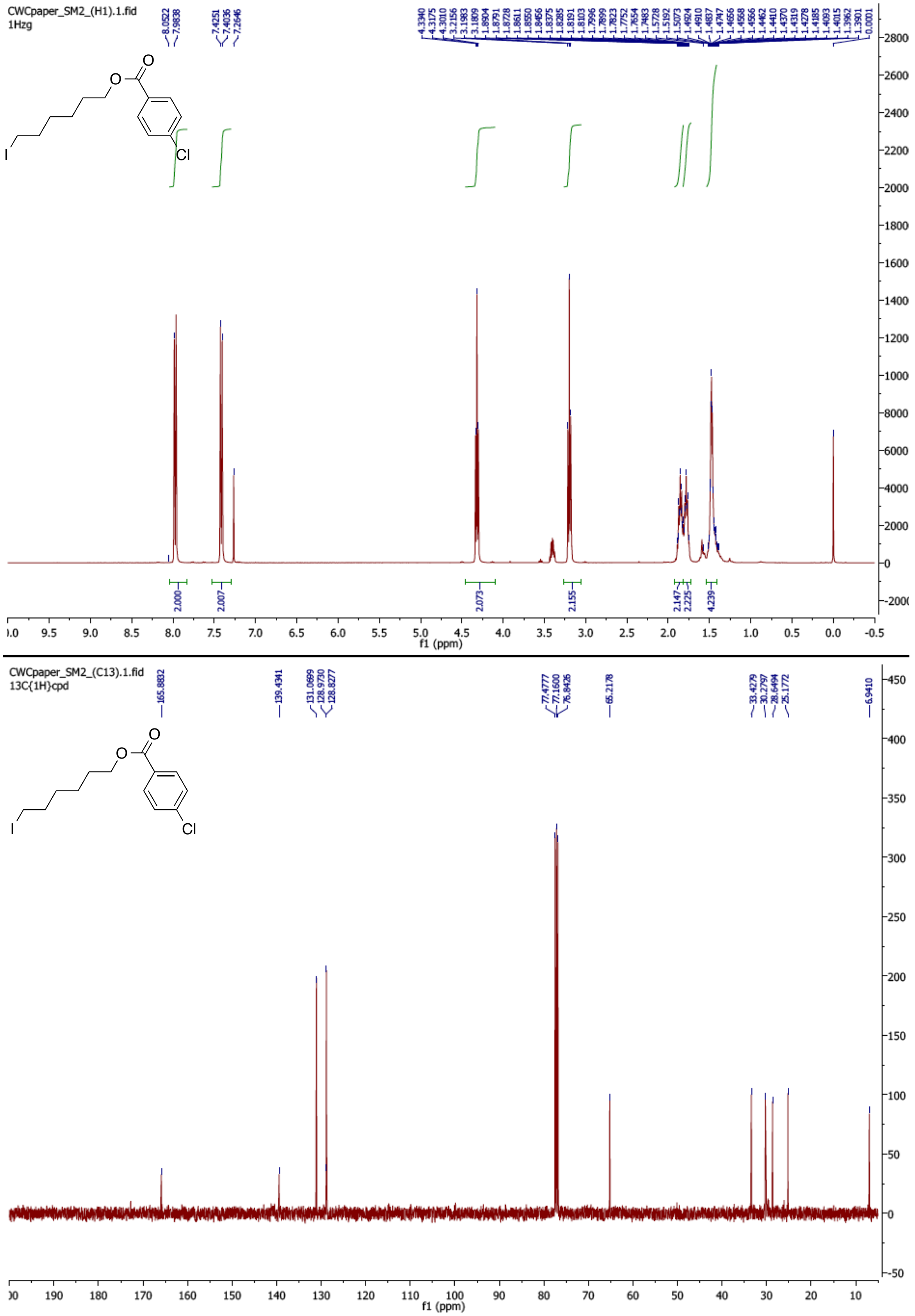

Supplementary Figure 8. ^{1}H and ^{13}C NMR spectra of 6-Iodohexyl 4-chlorobenzoate (S2)

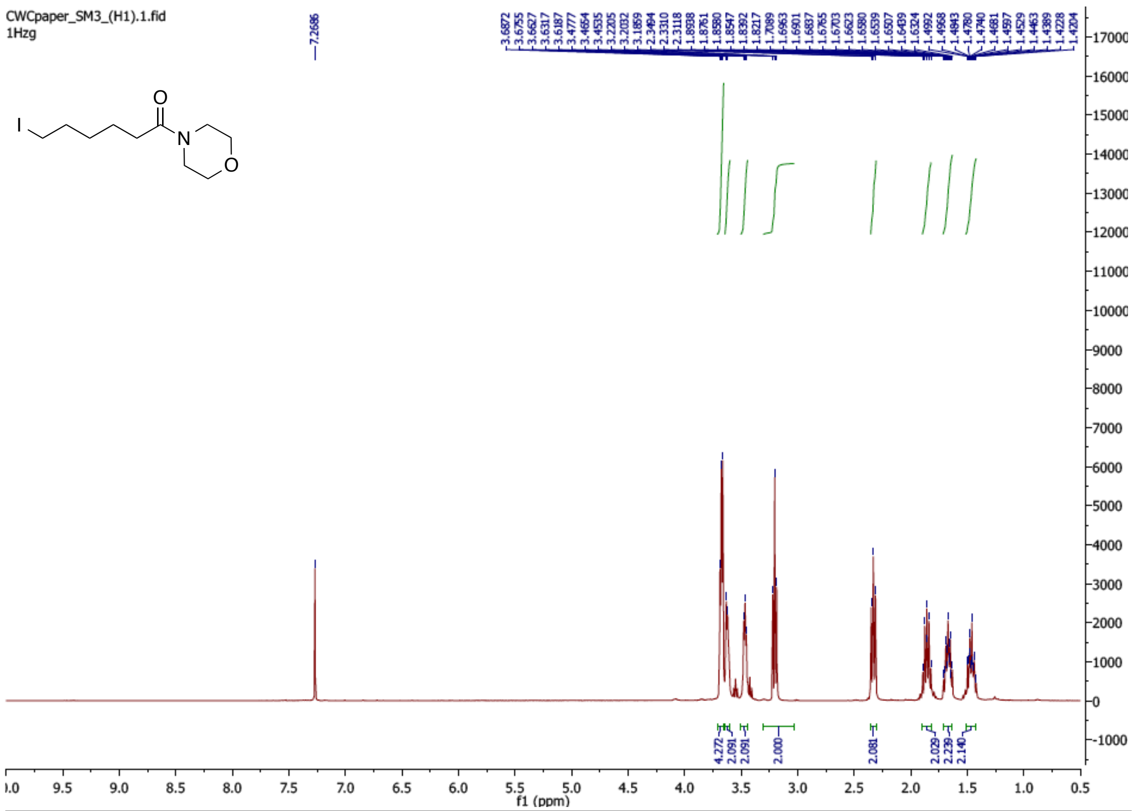

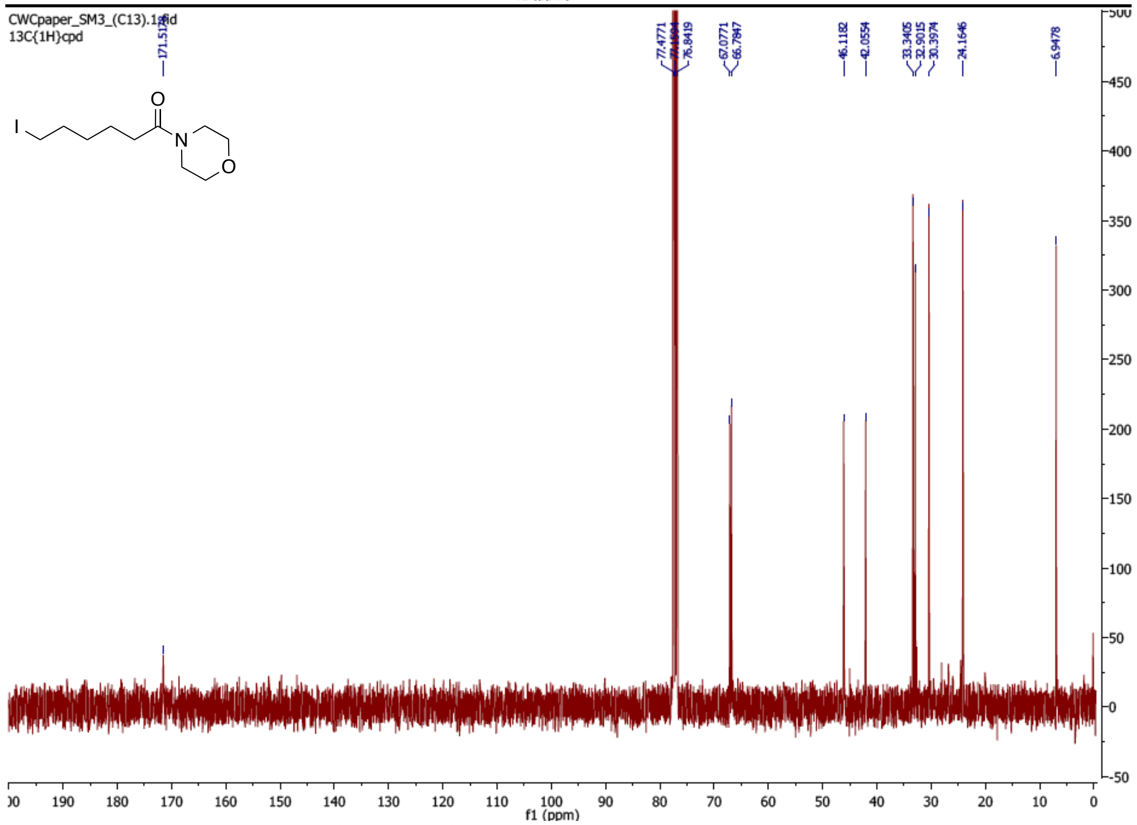

Supplementary Figure 9. ^{1}H and ^{13}C NMR spectra of 6-Iodo-1-morpholinohexan-1-one (S3)

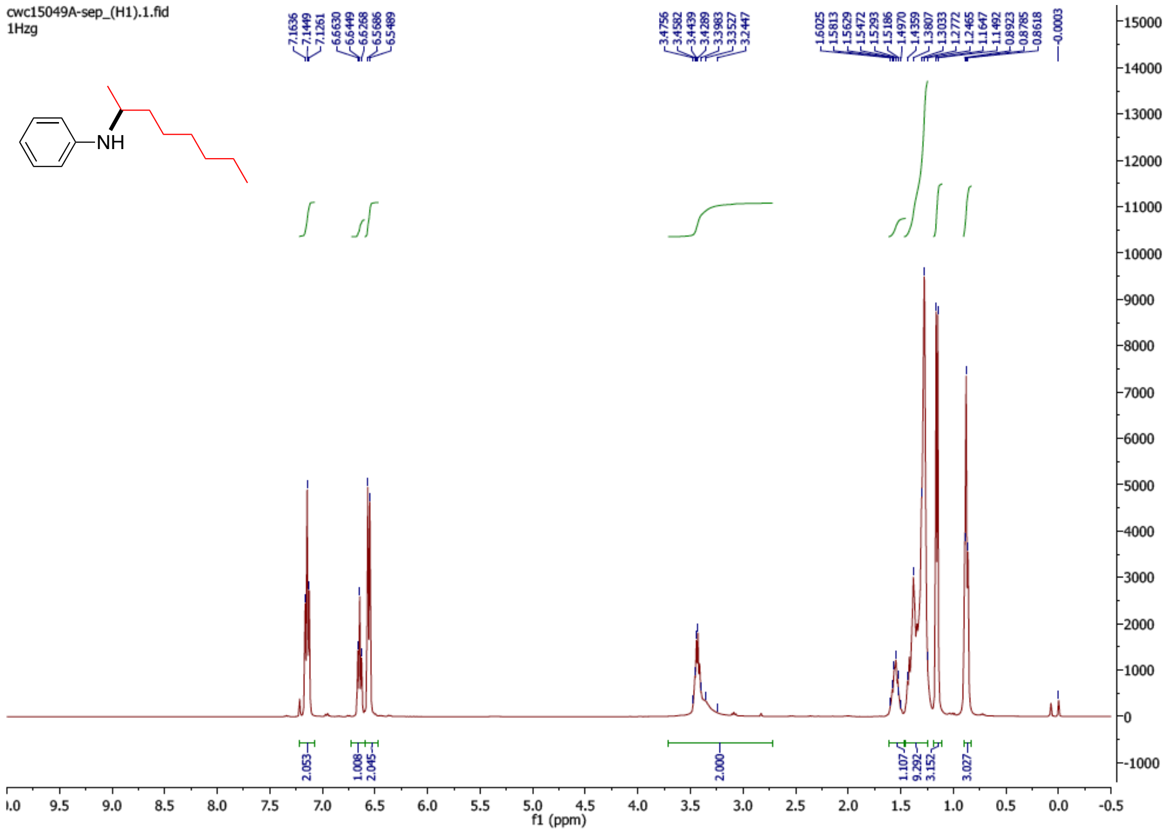

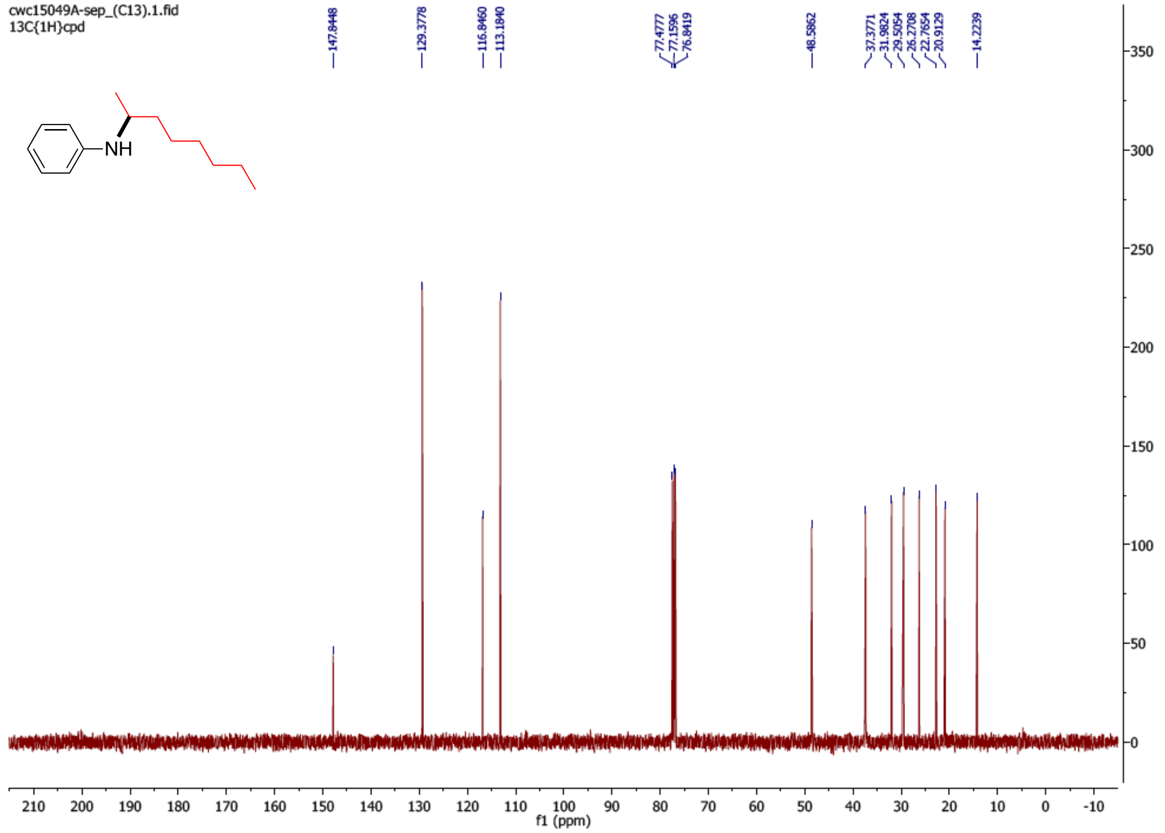

Supplementary Figure 10. ^{1}H and ^{13}C NMR spectra of *N*-(Octan-2-yl)aniline (3a)

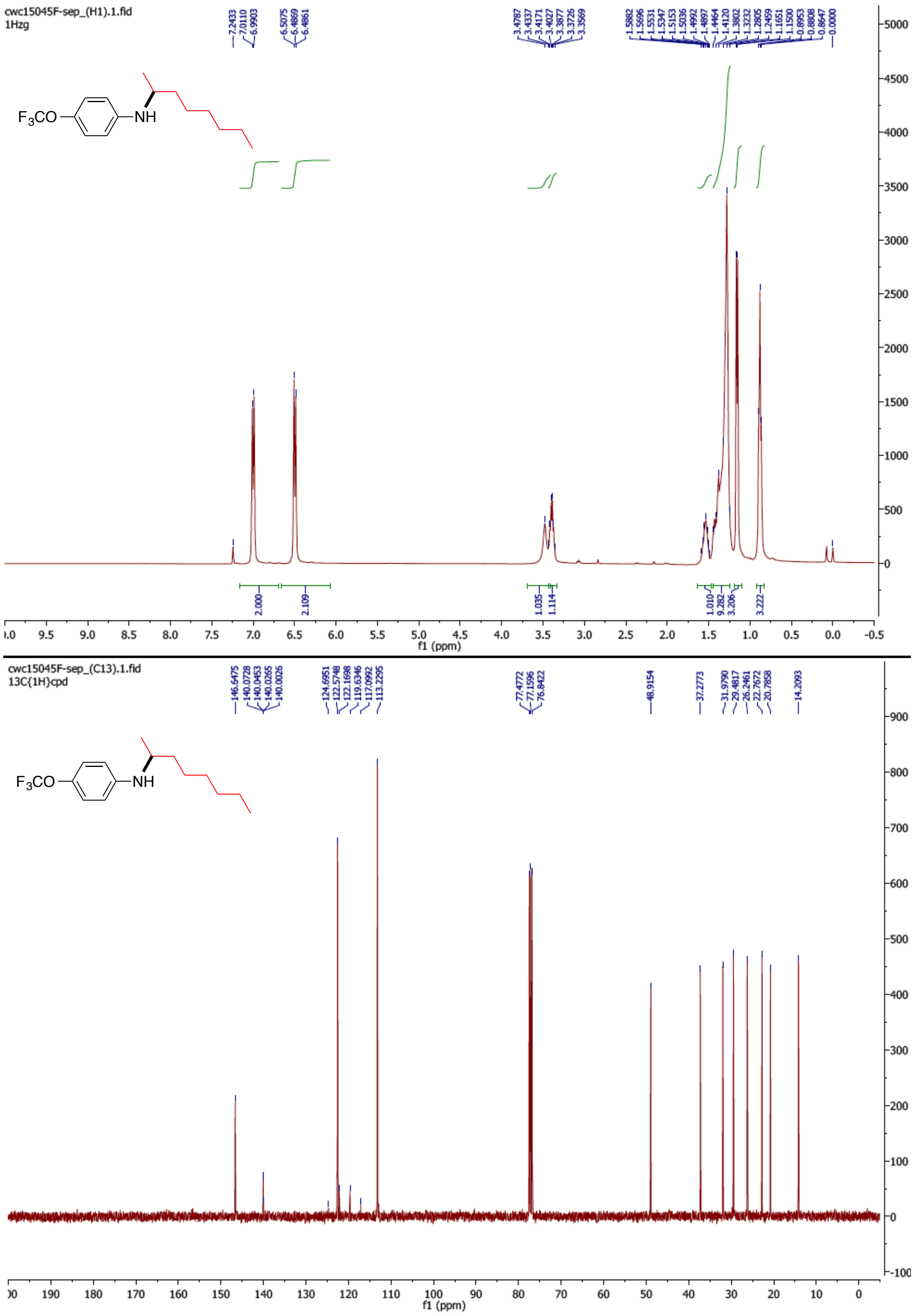

Supplementary Figure 11. ^{1}**H and** ^{13}**C NMR spectra of** ***N*****-(Octan-2-yl)-4-(trifluoromethoxy)aniline (3b)**

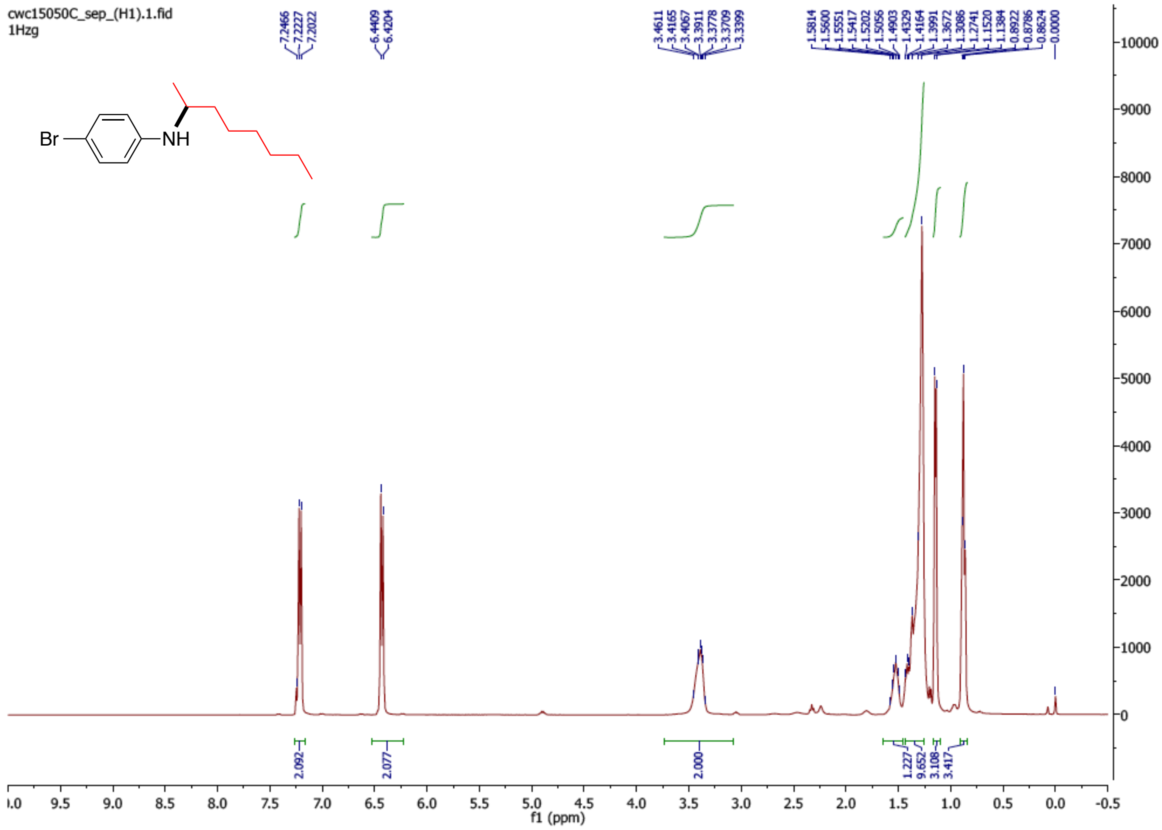

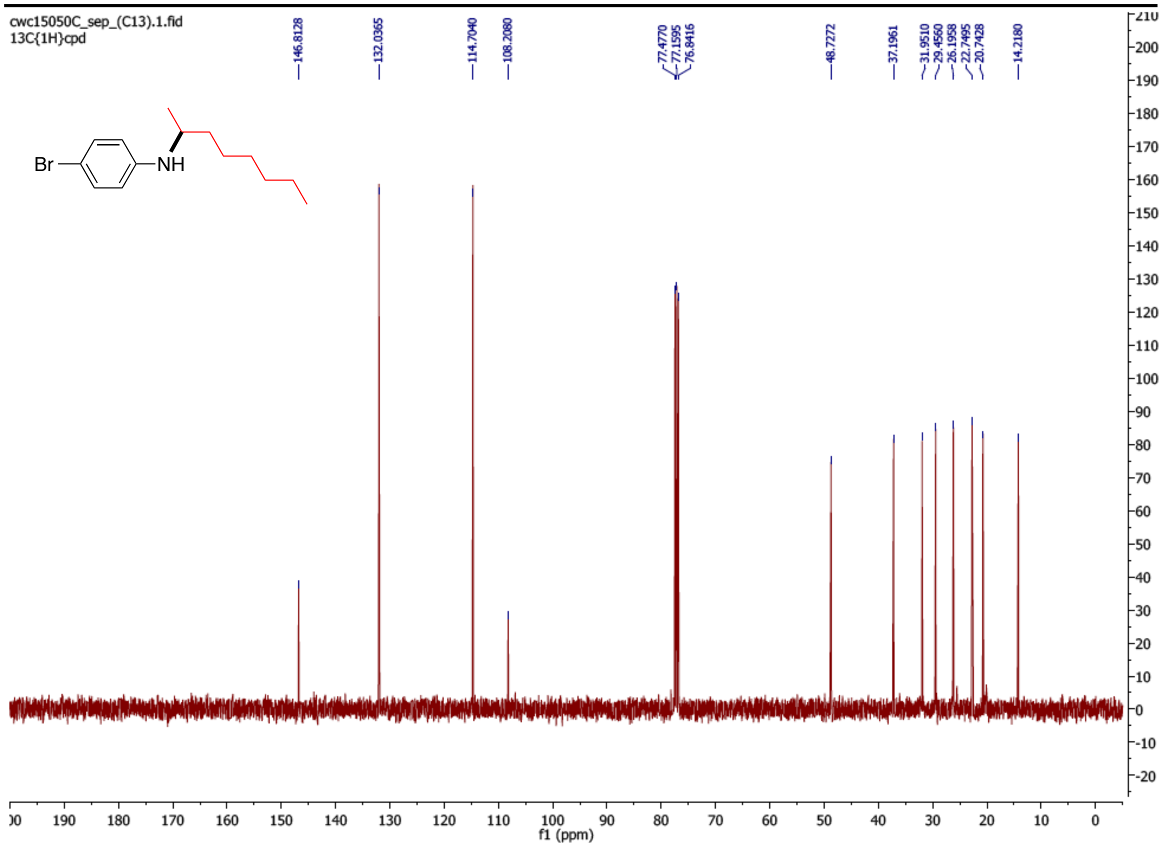

Supplementary Figure 12. ^{1}H and ^{13}C NMR spectra of 4-Bromo-*N*-(octan-2-yl)aniline (3c)

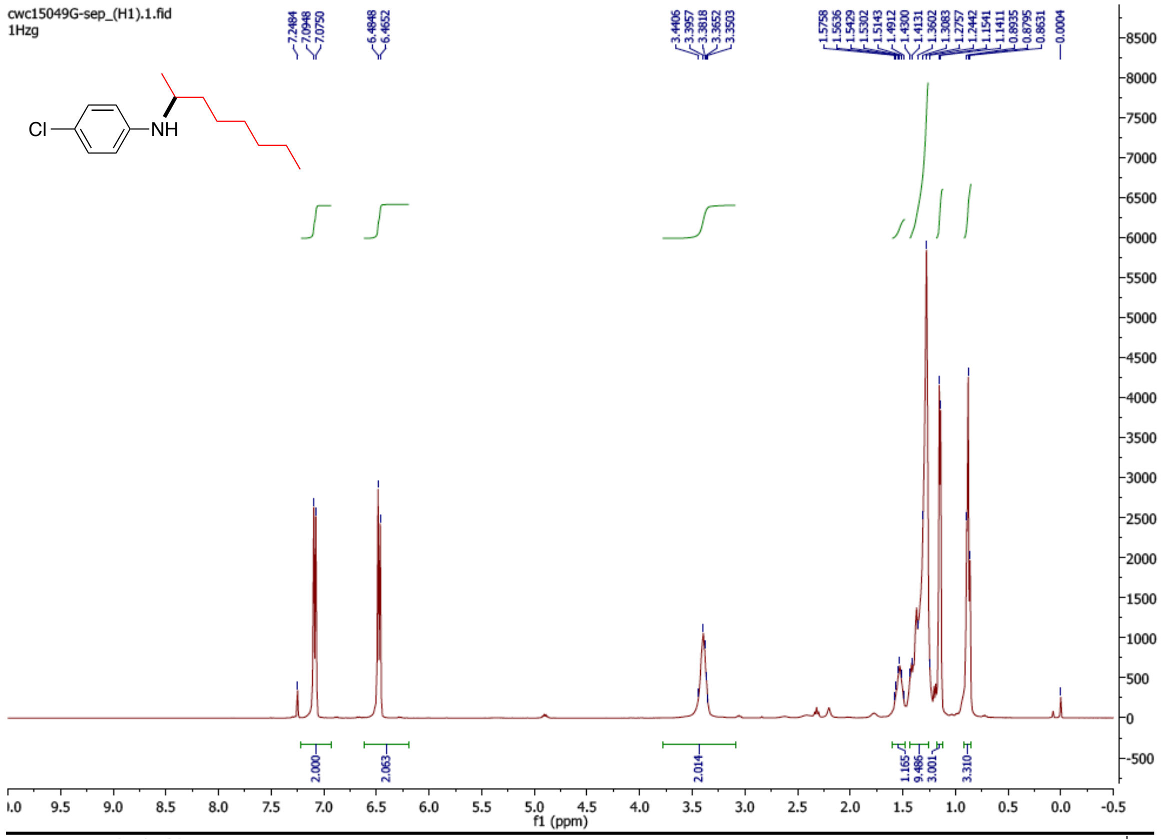

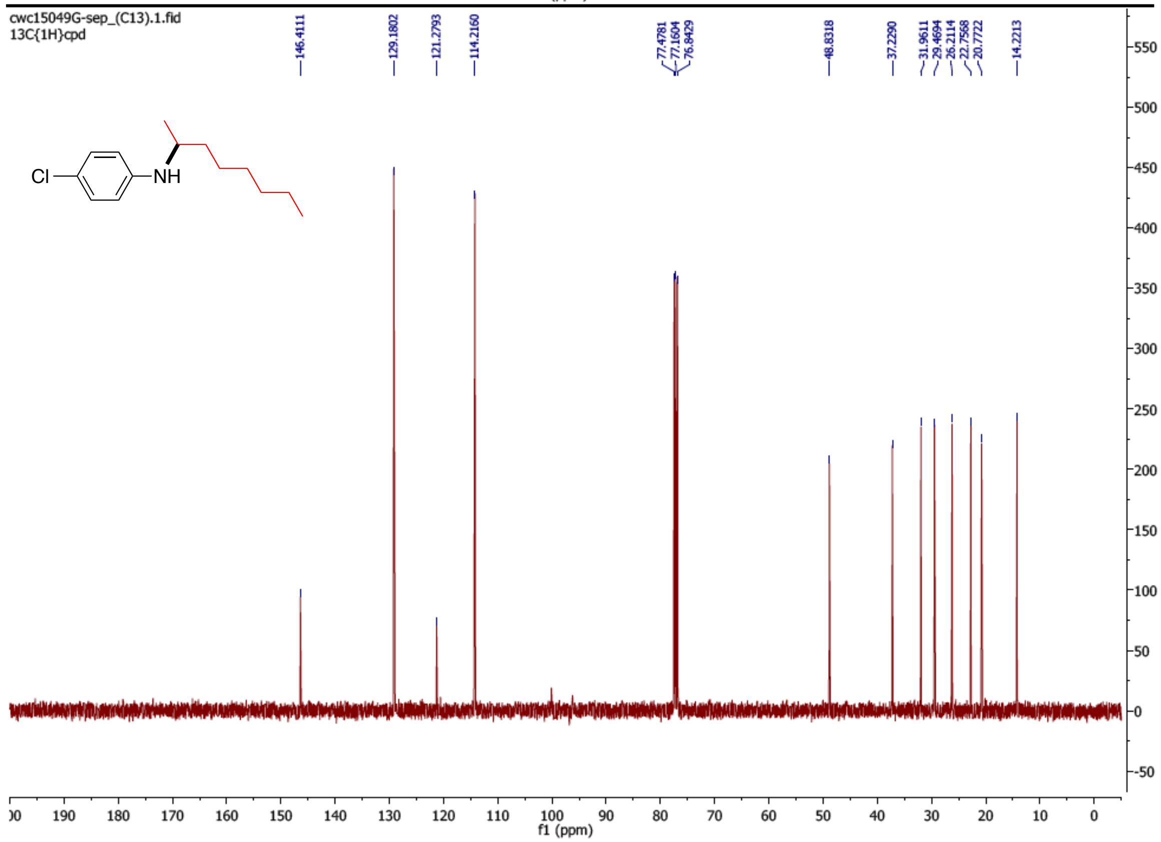

Supplementary Figure 13. ^{1}H and ^{13}C NMR spectra of 4-Chloro-*N*-(octan-2-yl)aniline (3d)

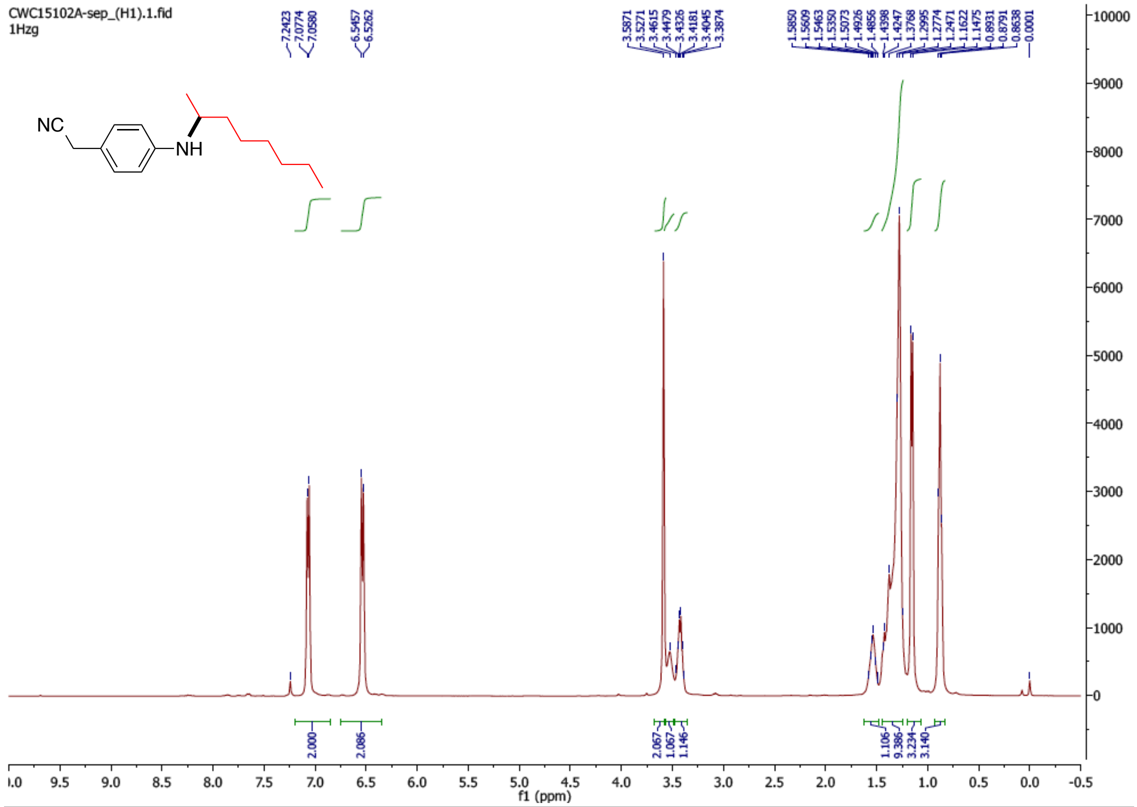

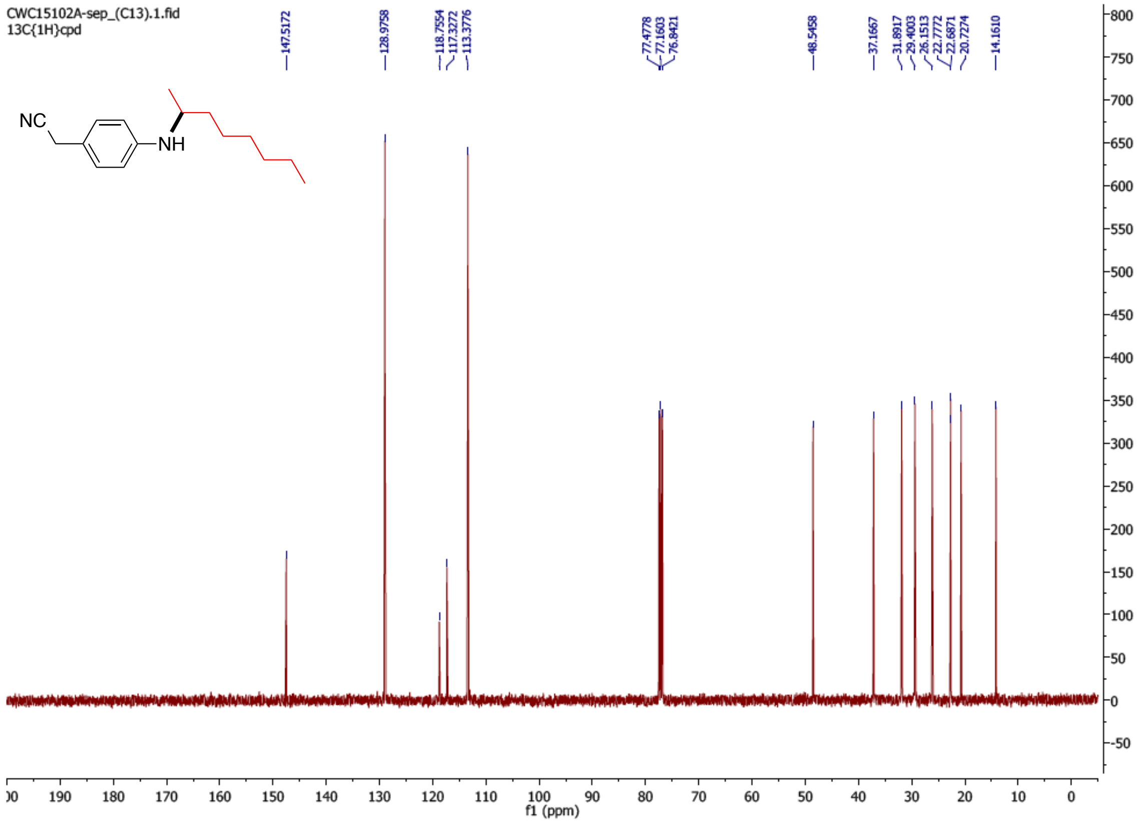

Supplementary Figure 14. ^{1}**H and** ^{13}**C NMR spectra of 2-(4-(Octan-2-ylamino)phenyl)acetonitrile (3e)**

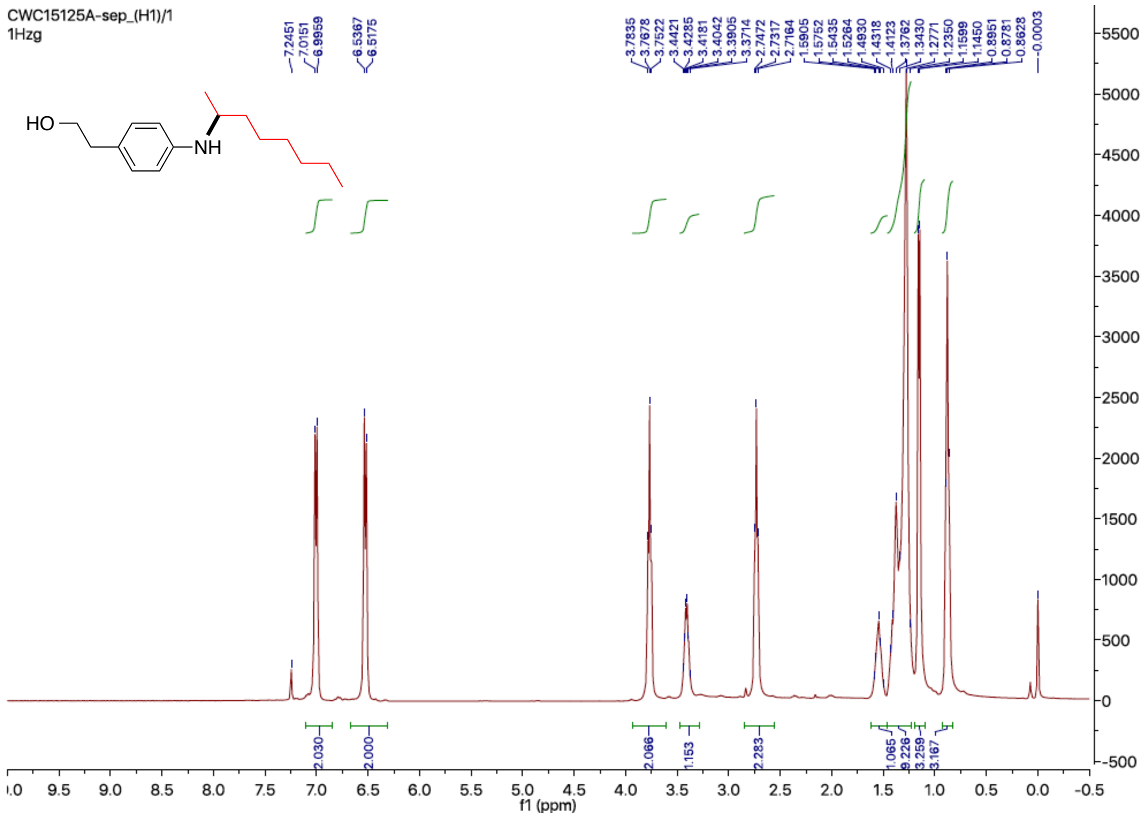

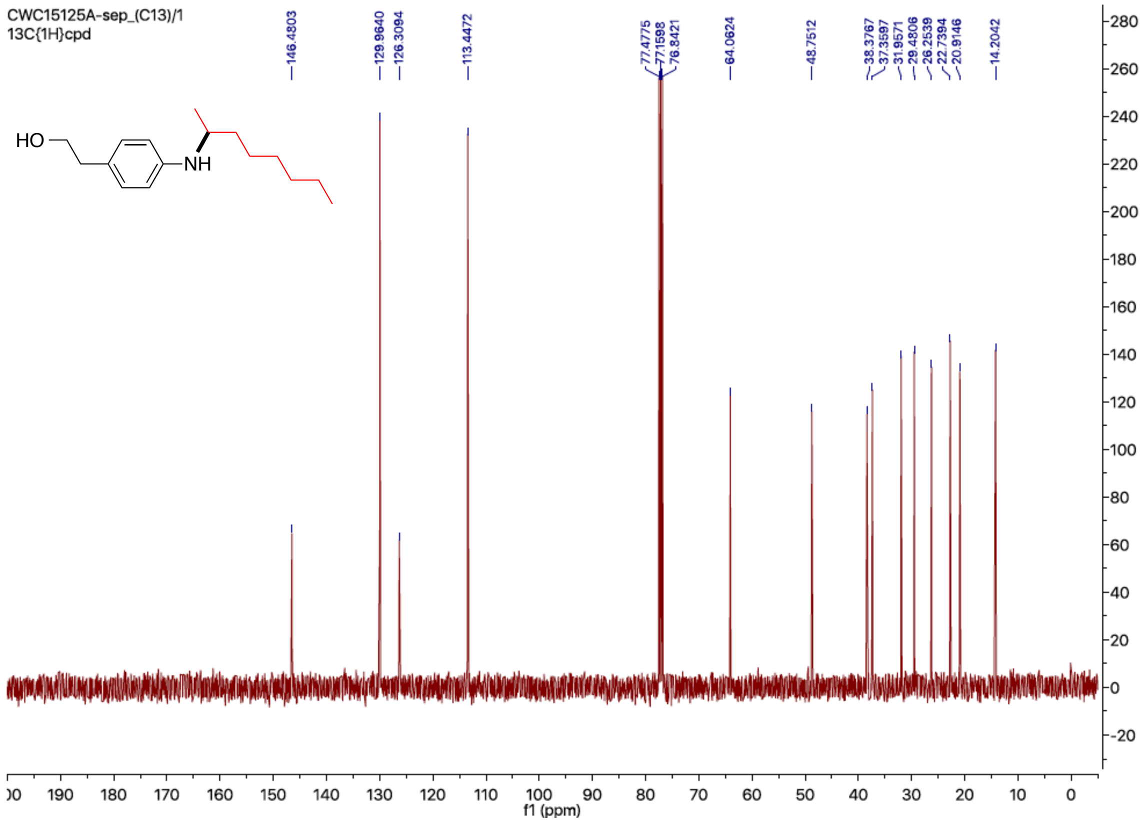

Supplementary Figure 15. ^{1}H and ^{13}C NMR spectra of 2-(4-(Octan-2-ylamino)phenyl)ethan-1-ol (3f)

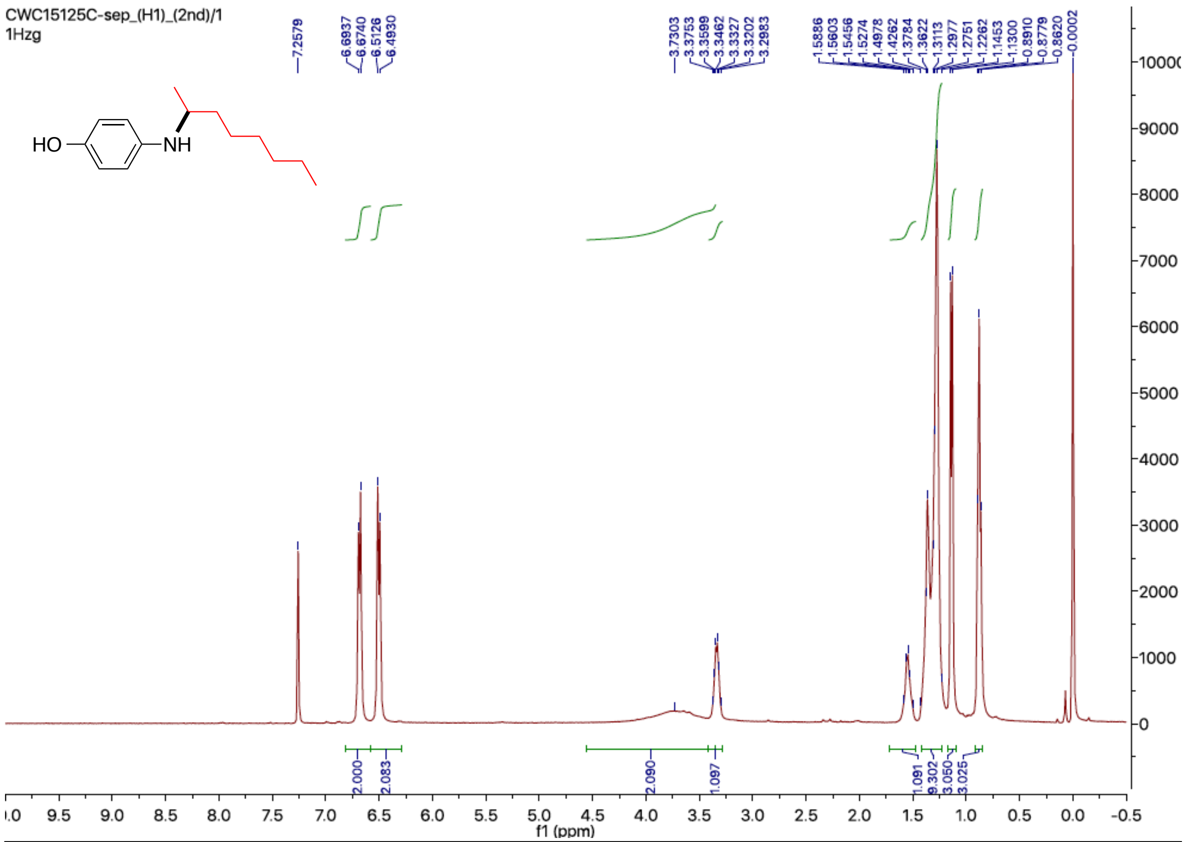

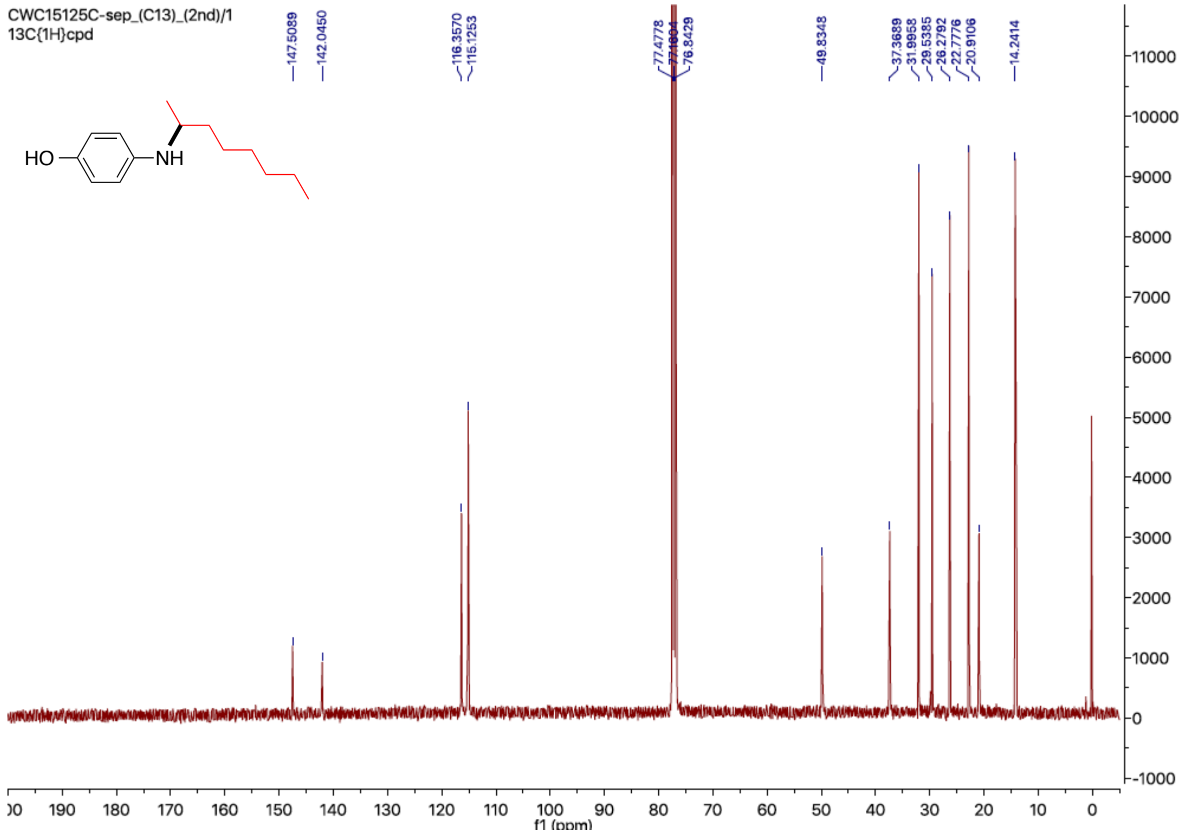

Supplementary Figure 16. ^{1}H and ^{13}C NMR spectra of 4-(Octan-2-ylamino)phenol (3g)

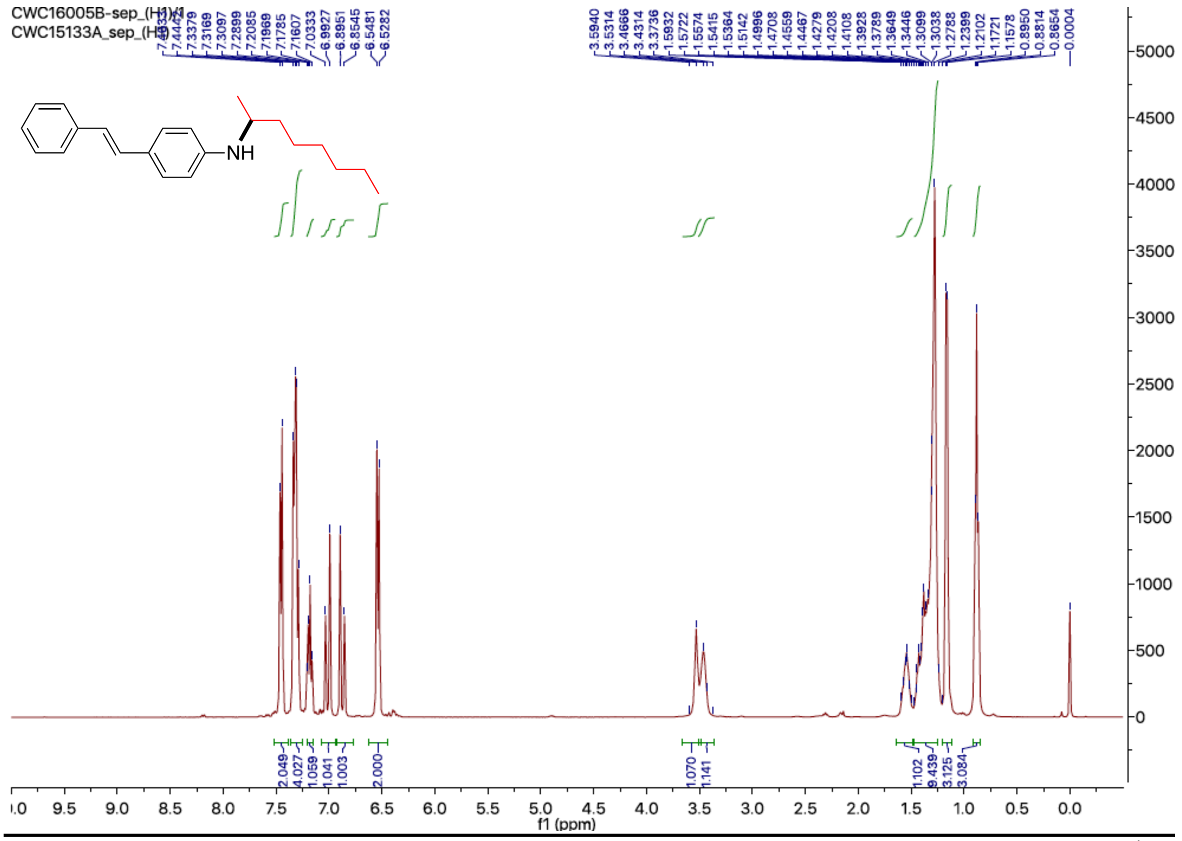

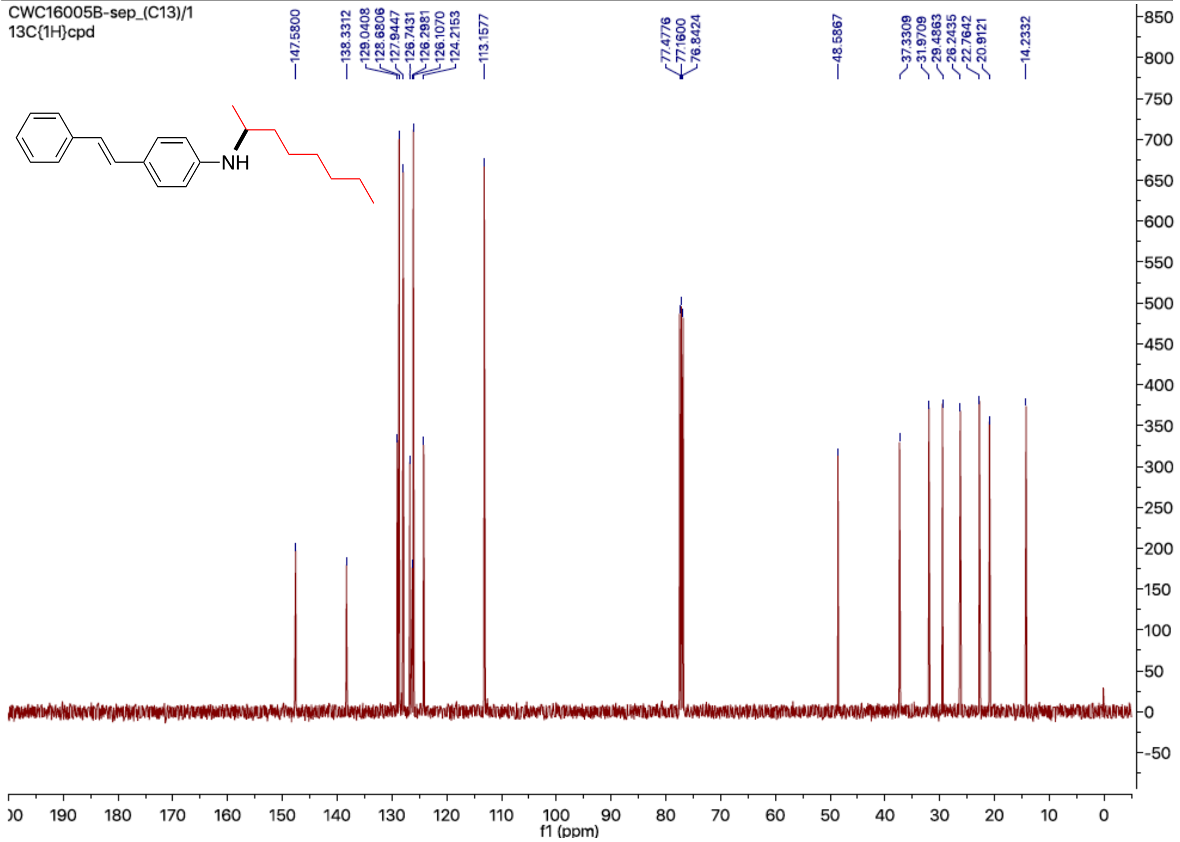

Supplementary Figure 17. ^{1}H and ^{13}C NMR spectra of *N*-(Octan-2-yl)-4-styrylaniline (3h)

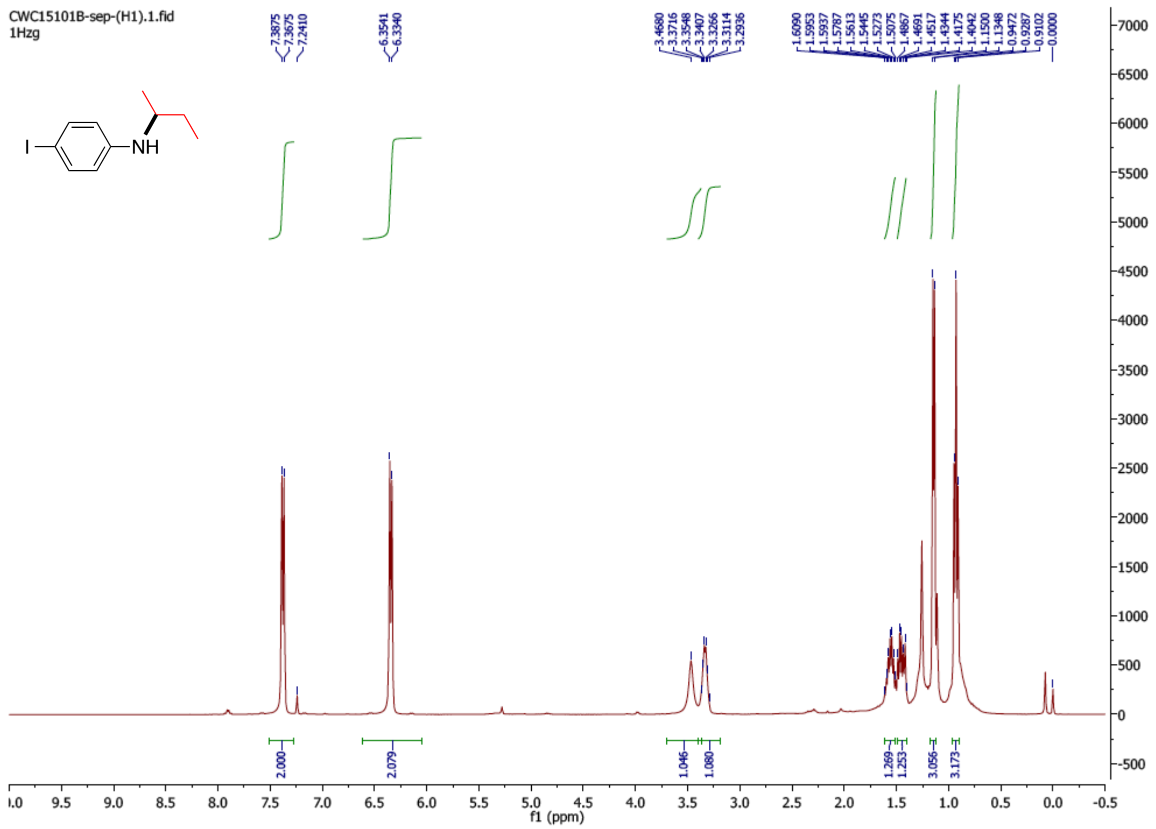

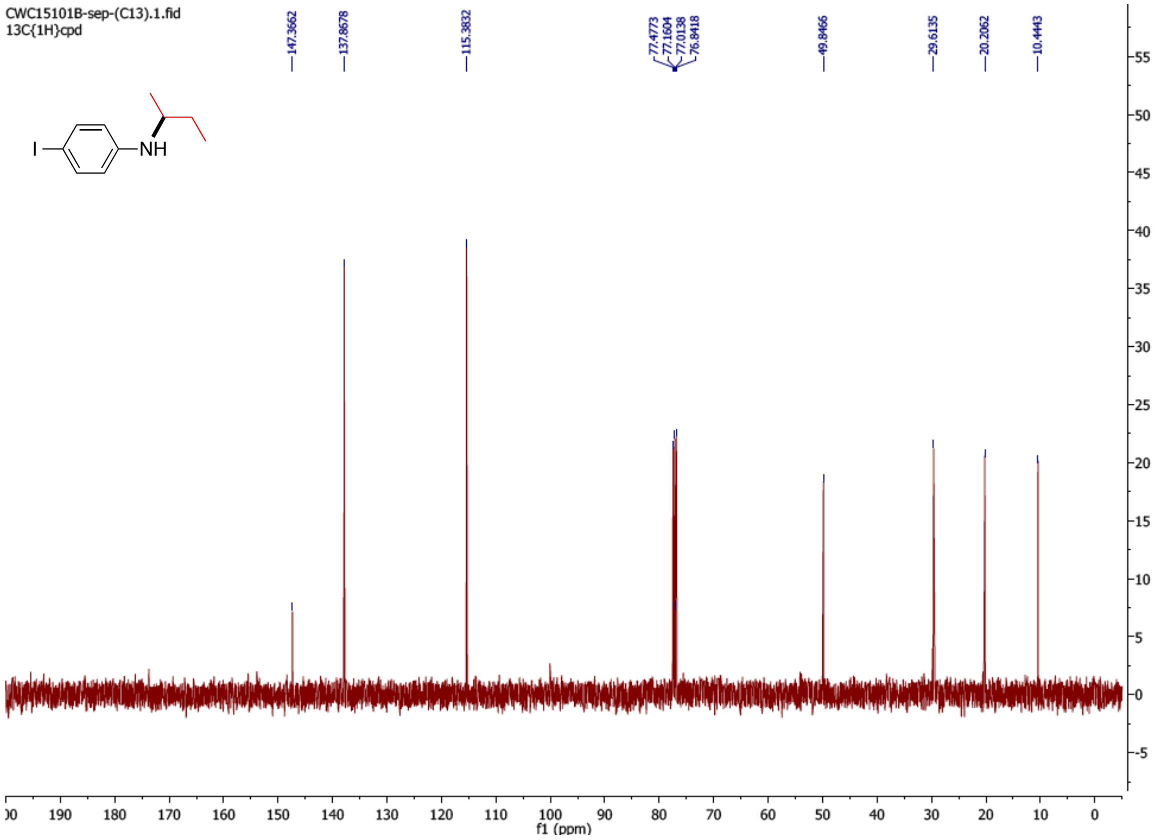

Supplementary Figure 18. ^{1}H and ^{13}C NMR spectra of *N*-(*sec*-Butyl)-4-iodoaniline (3i)

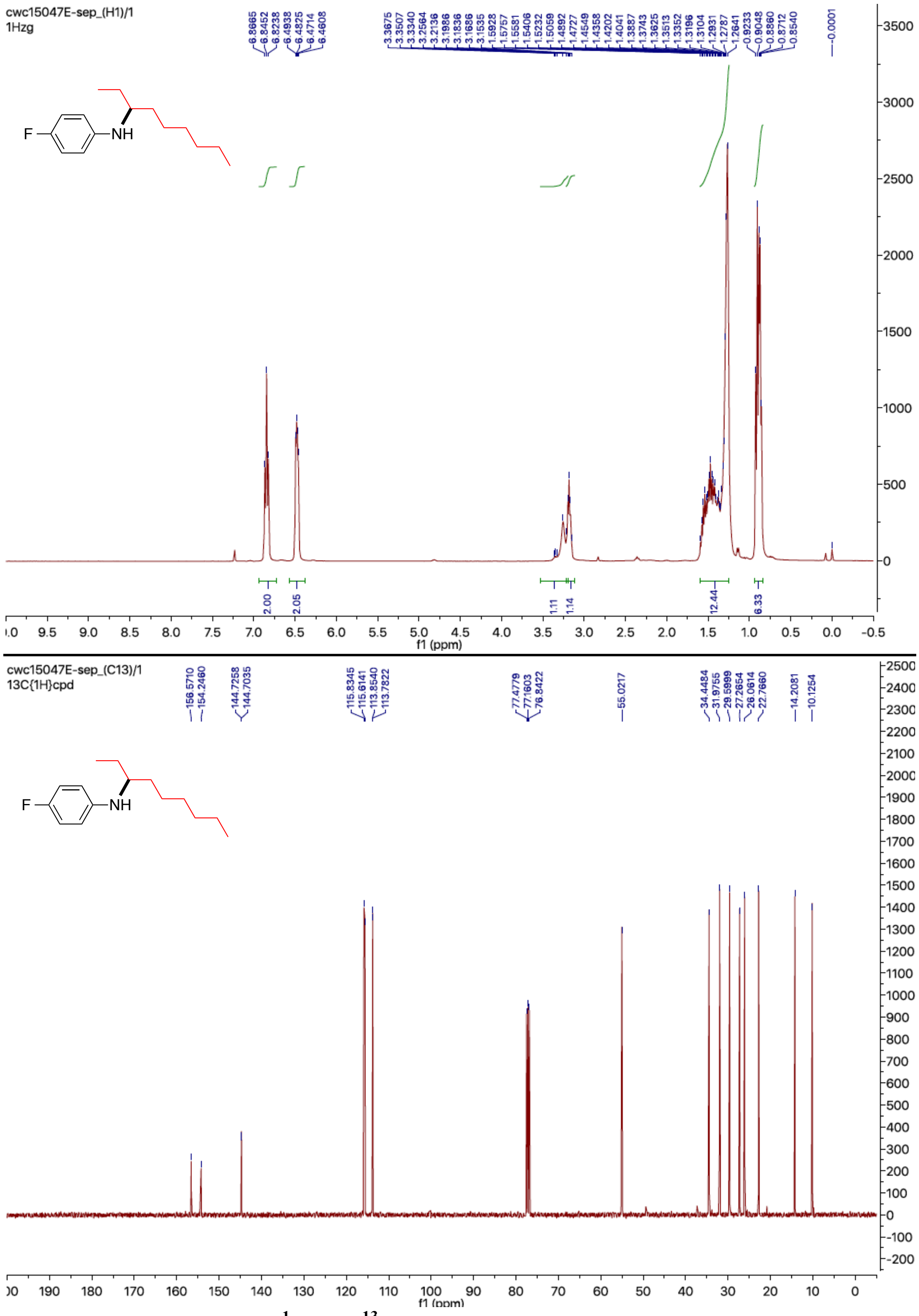

Supplementary Figure 19. ^{1}**H and** ^{13}**C NMR spectra of 4-Fluoro-*N*-(nonan-3-yl)aniline (3j)**

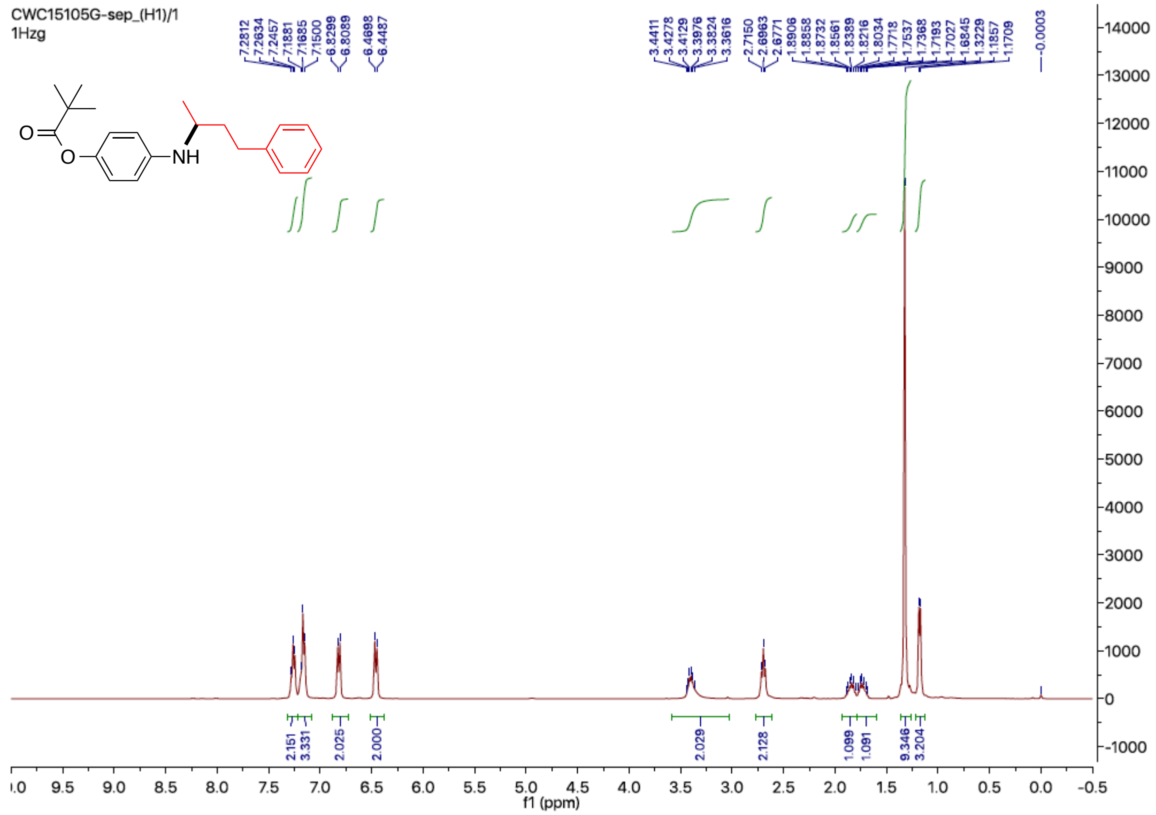

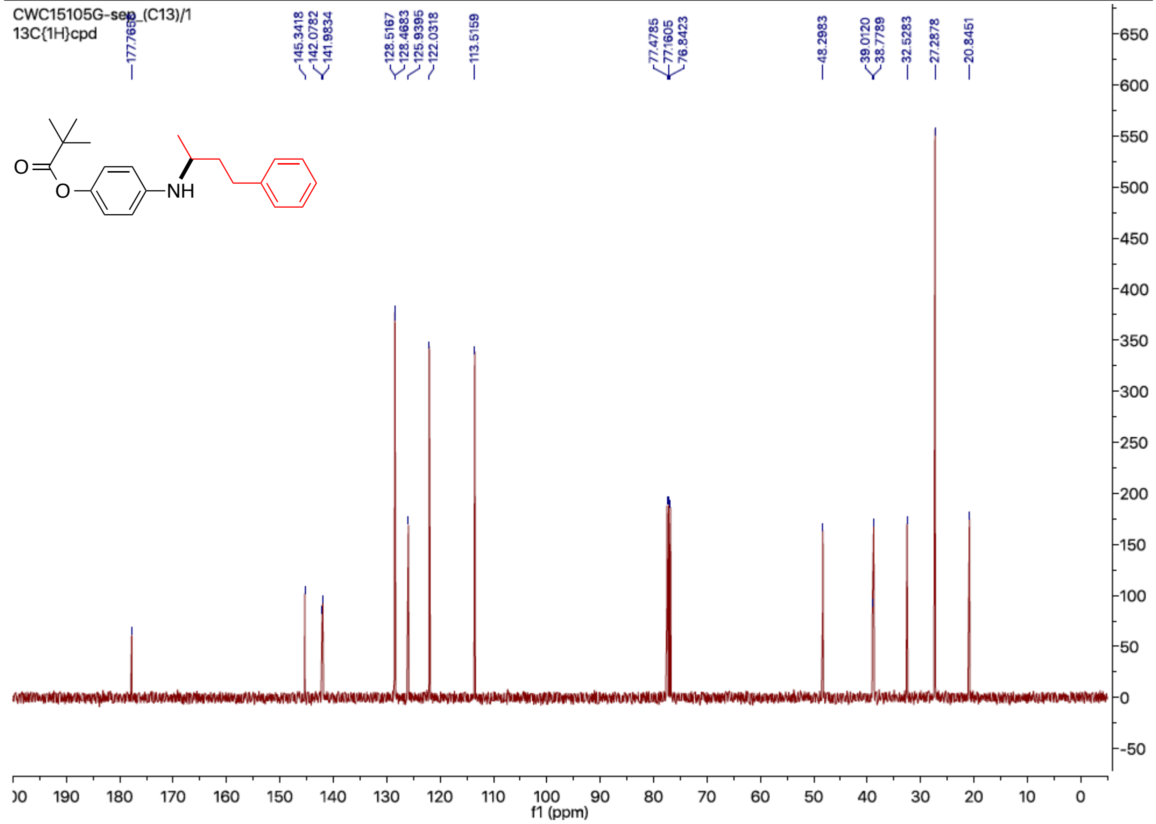

Supplementary Figure 20. ^{1}H and ^{13}C NMR spectra of 4-((4-Phenylbutan-2-yl)amino)phenyl pivalate (3k)

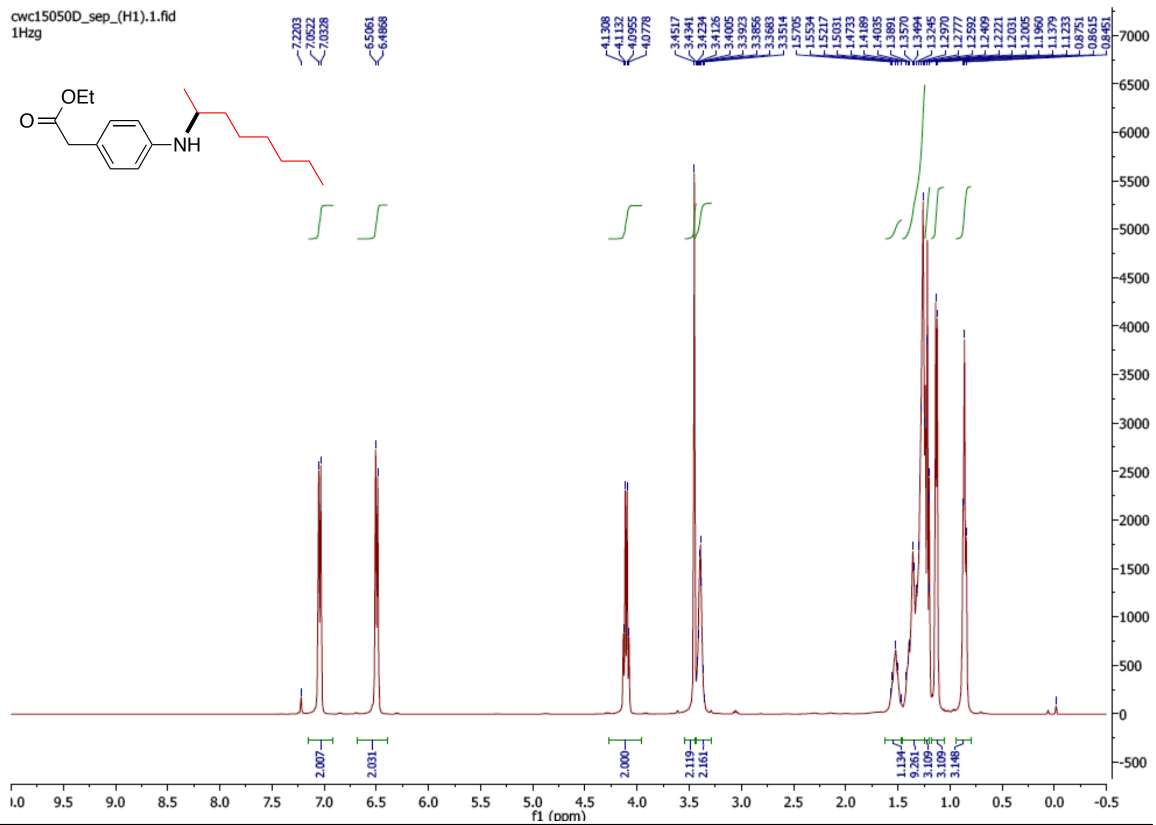

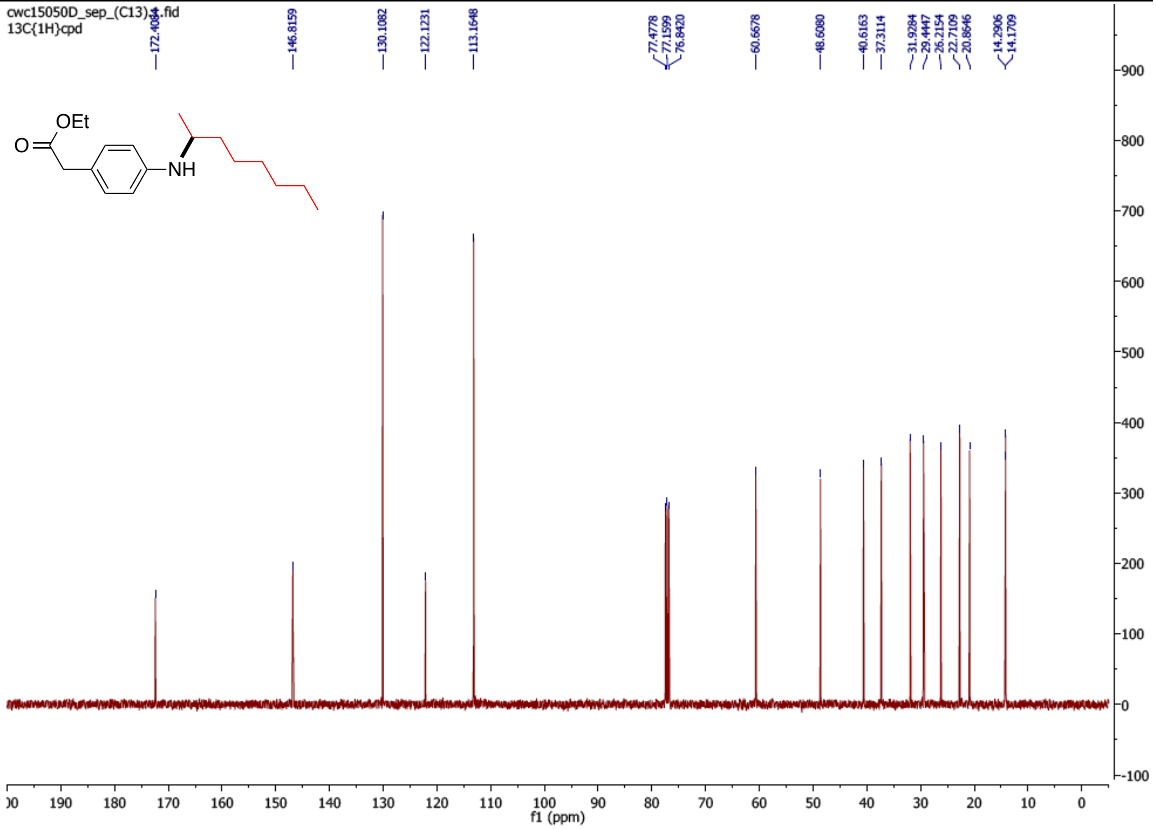

Supplementary Figure 21. ^{1}H and ^{13}C NMR spectra of Ethyl 2-(4-(Octan-2-ylamino)phenyl)acetate (3l)

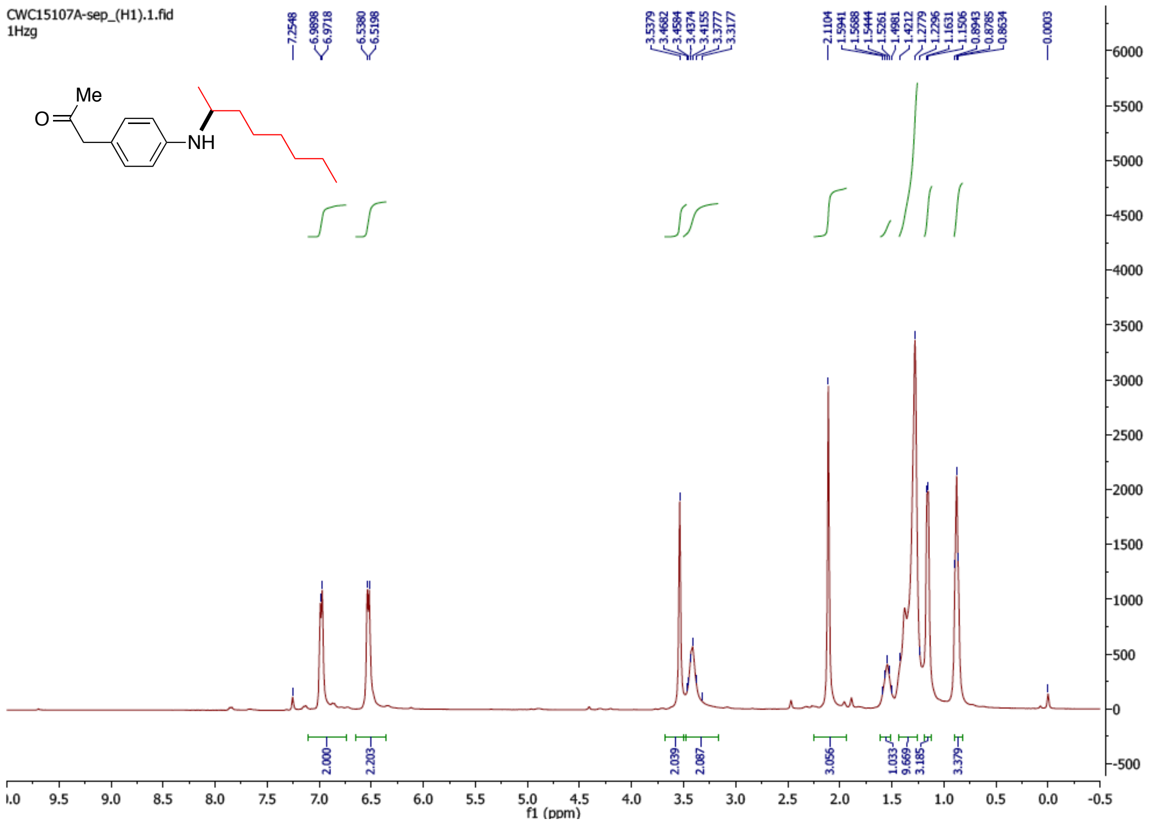

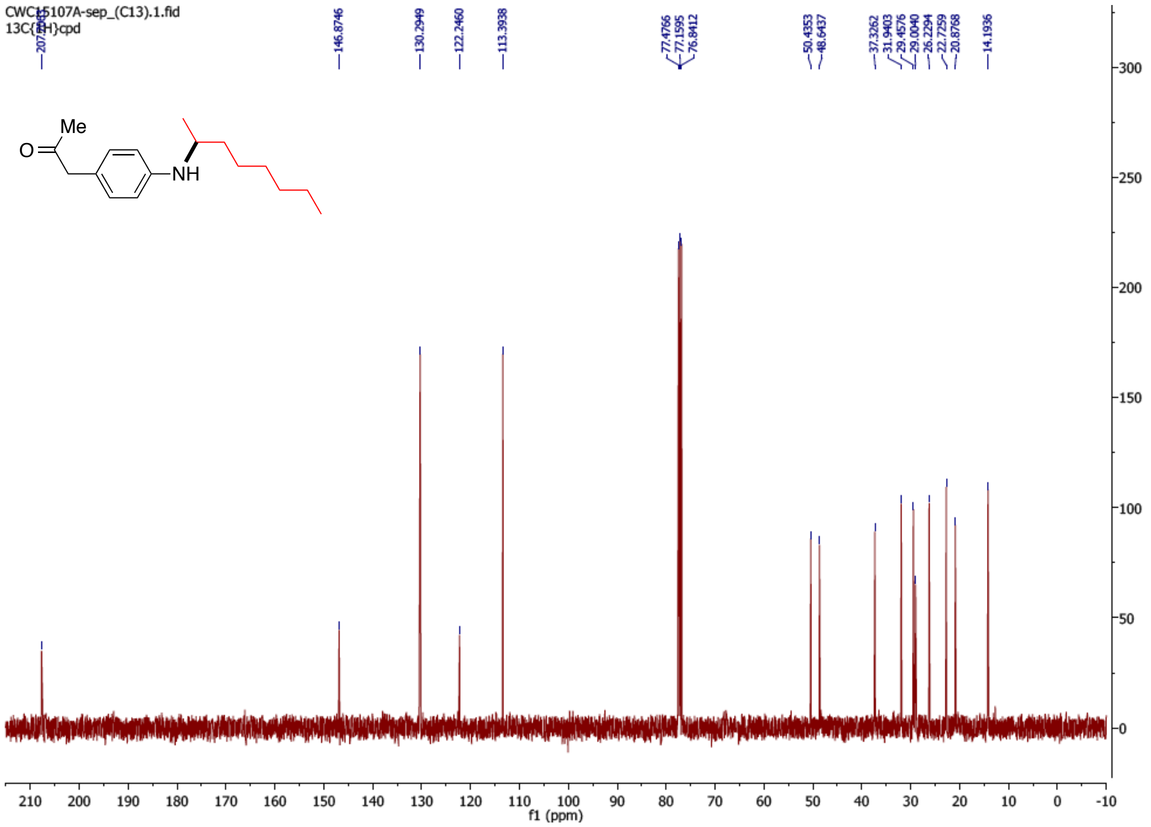

Supplementary Figure 22. ^{1}H and ^{13}C NMR spectra of 1-(4-(Octan-2-ylamino)phenyl)propan-2-one (3m)

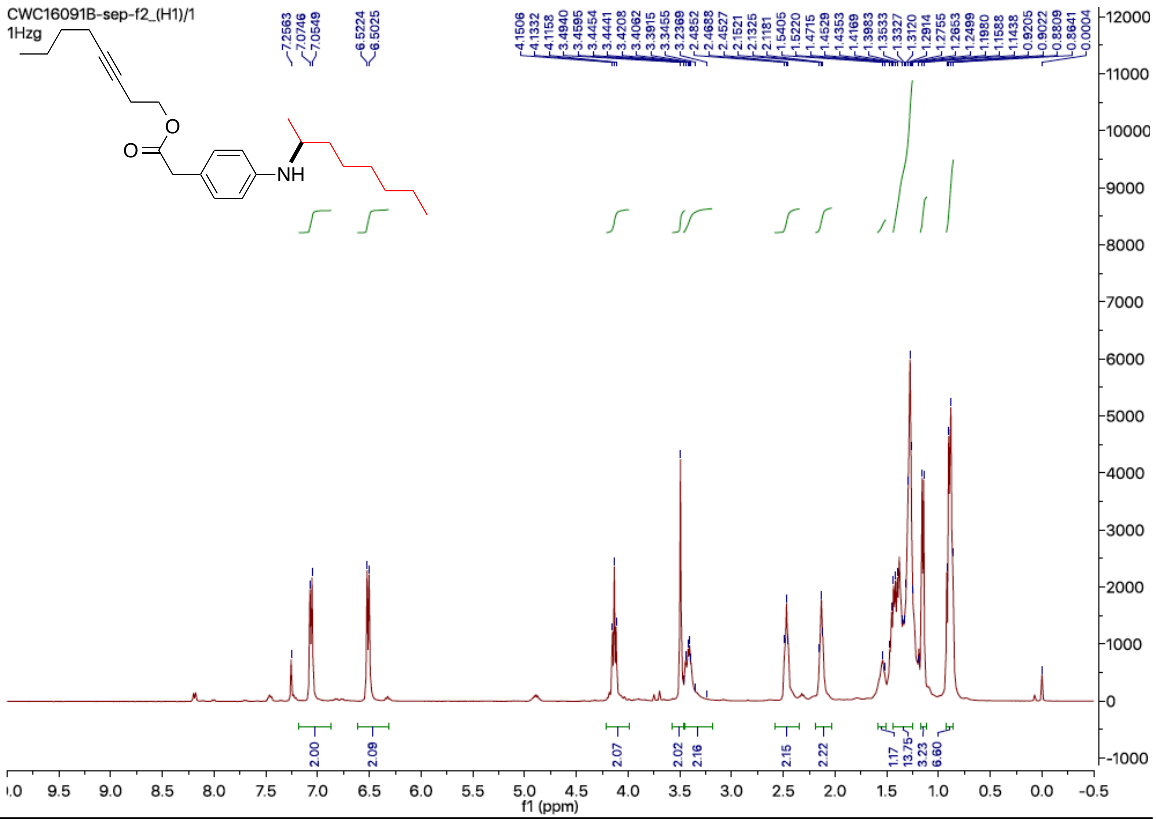

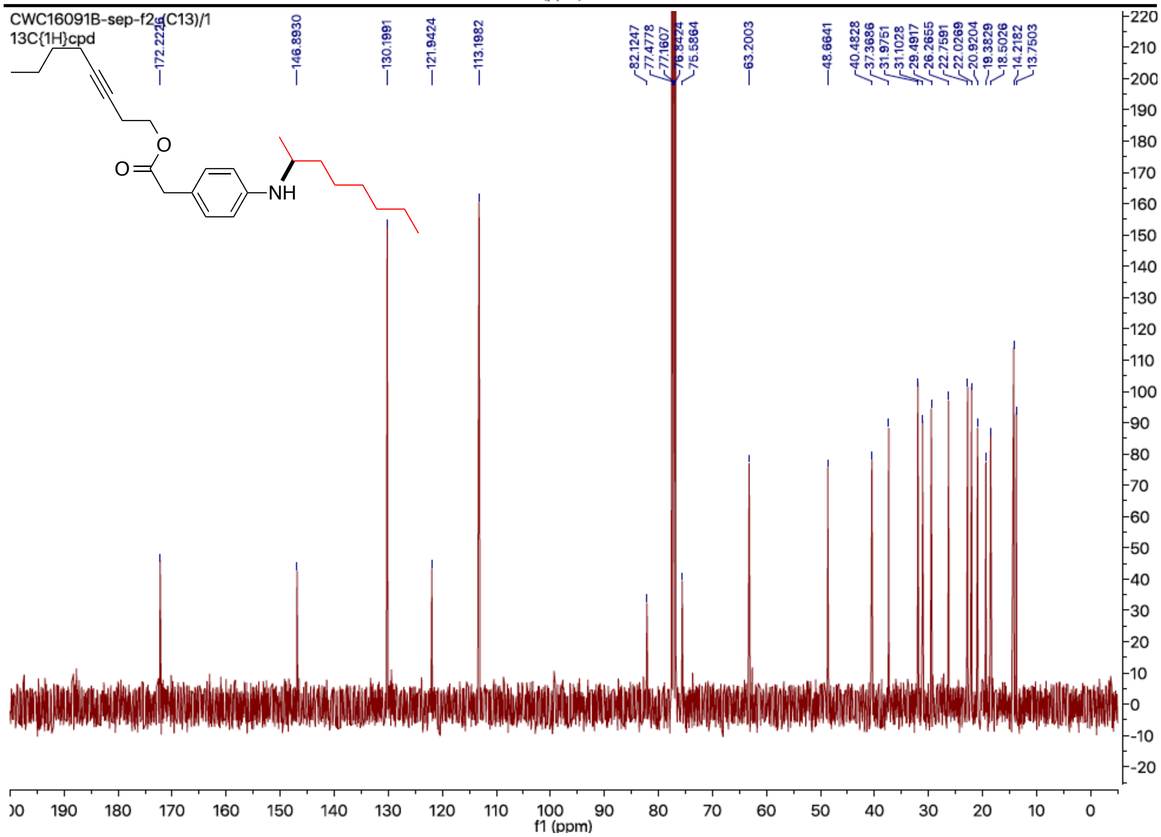

Supplementary Figure 23. ^{1}H and ^{13}C NMR spectra of Oct-3-yn-1-yl 2-(4-(octan-2-ylamino)phenyl)acetate (3n)

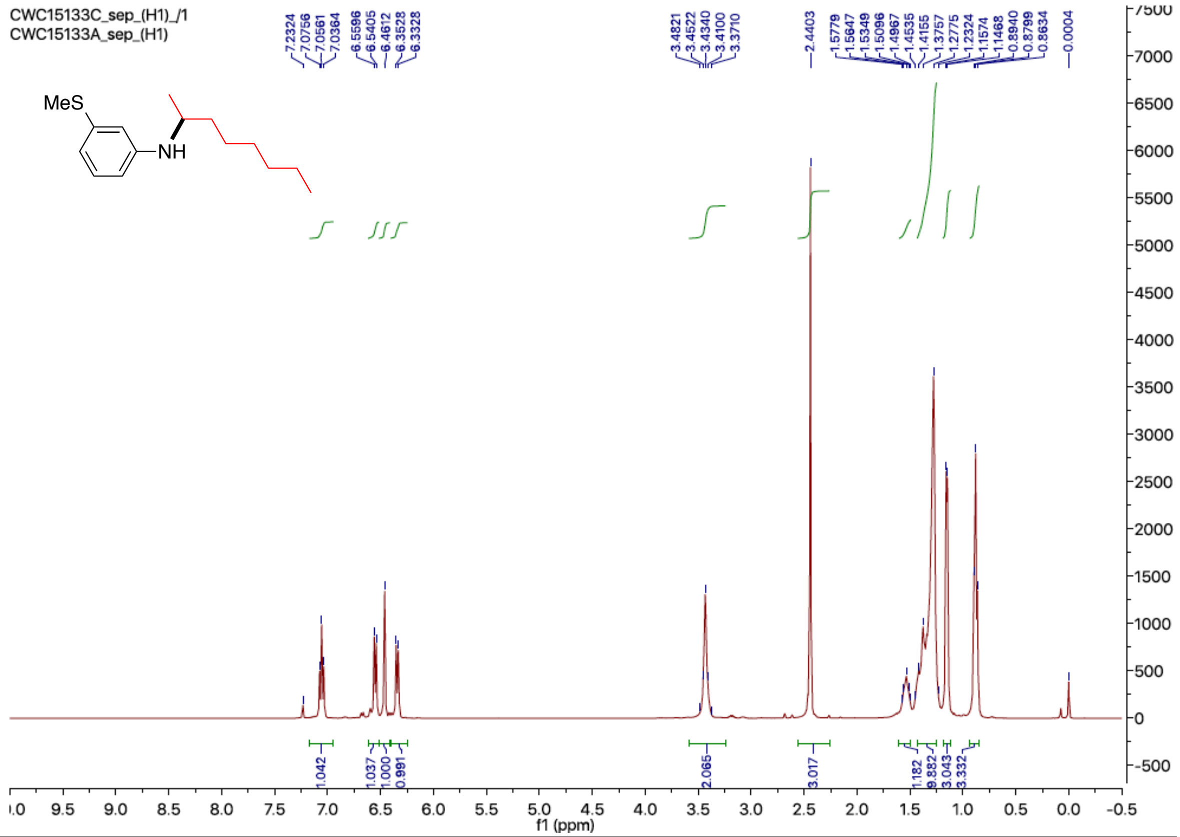

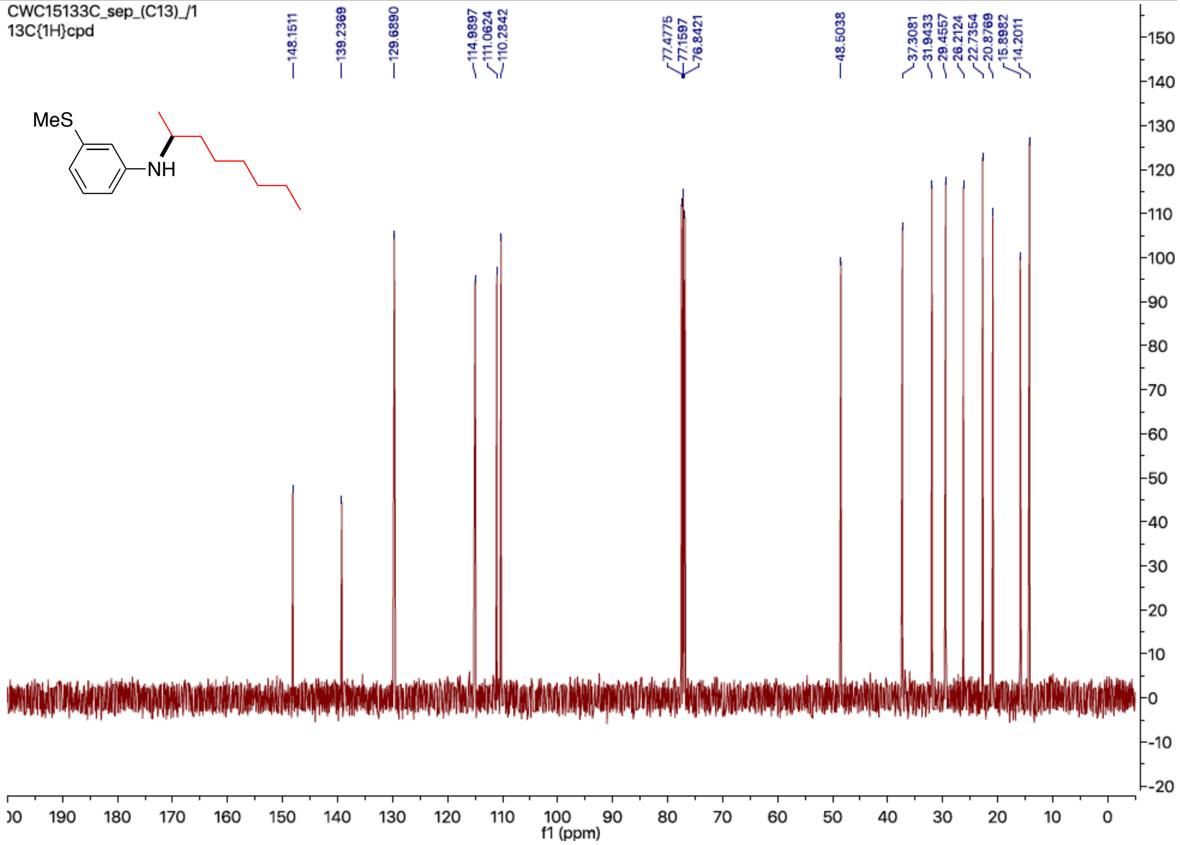

Supplementary Figure 24. ^{1}H and ^{13}C NMR spectra of 3-(Methylthio)-*N*-(octan-2-yl)aniline (3o)

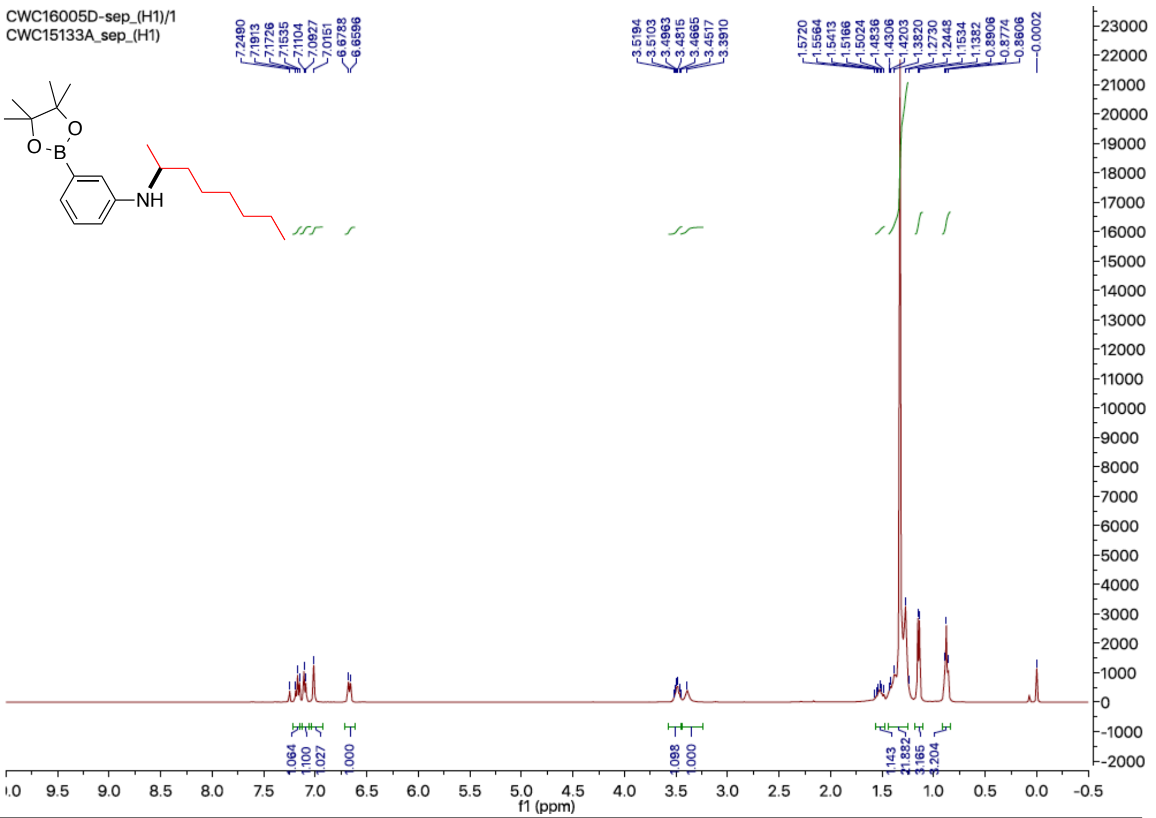

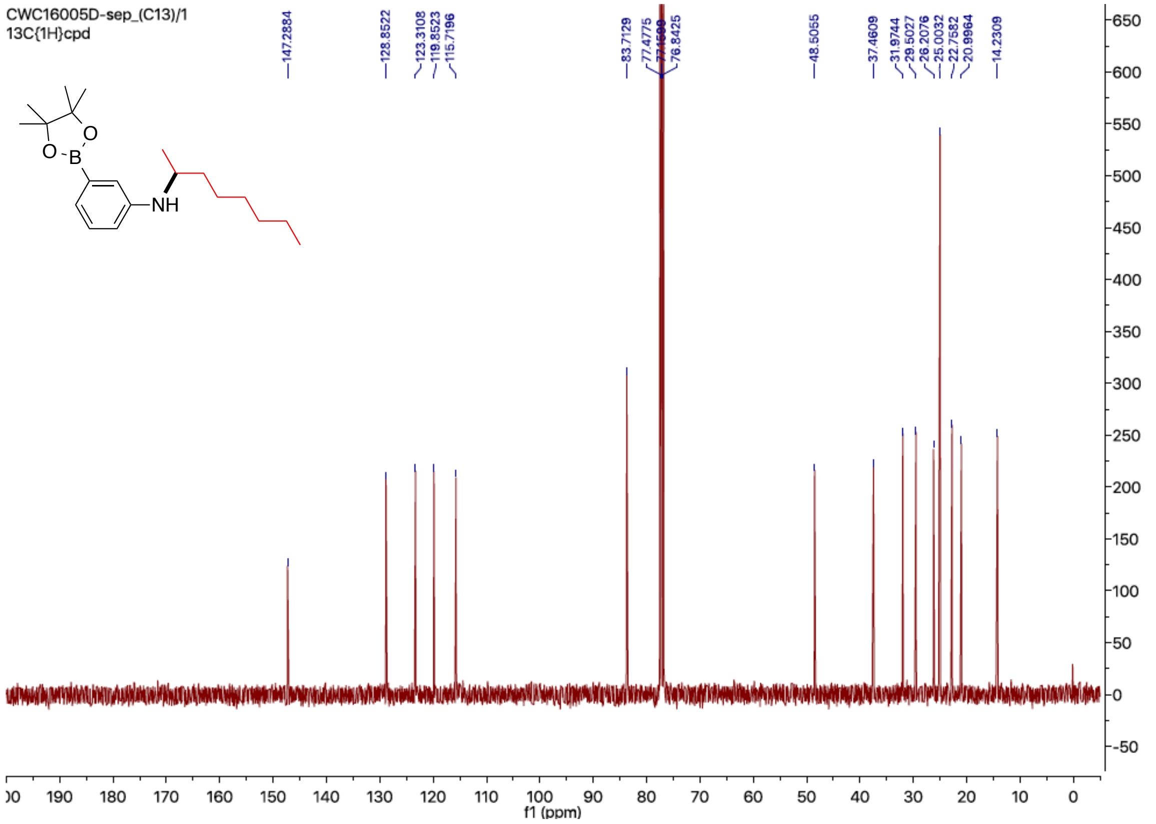

Supplementary Figure 25. ^{1}H and ^{13}C NMR spectra of *N*-(Octan-2-yl)-3-(4,4,5,5-tetramethyl-1,3,2-dioxaborolan-2-yl)aniline (3p)

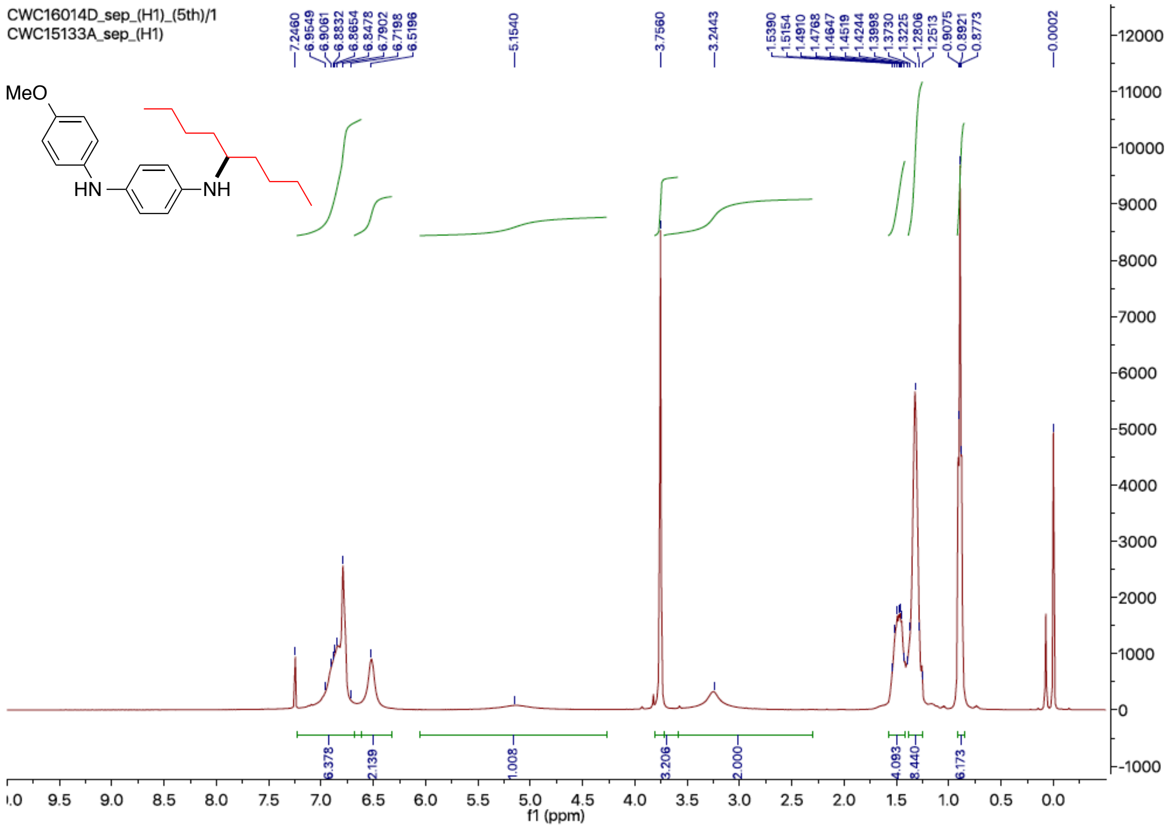

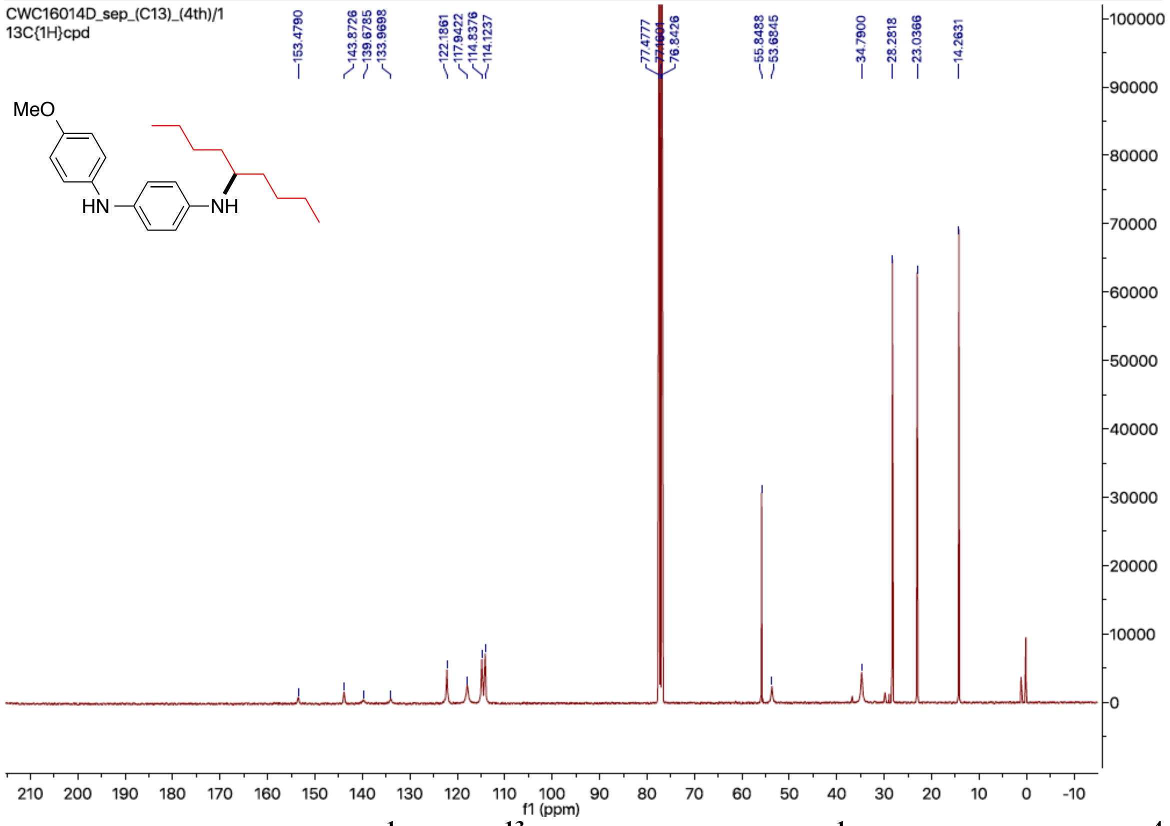

Supplementary Figure 26. ^{1}H and ^{13}C NMR spectra of *N*^{1}-(4-Methoxyphenyl)-*N*^{4}-(nonan-5-yl)benzene-1,4-diamine (3q)

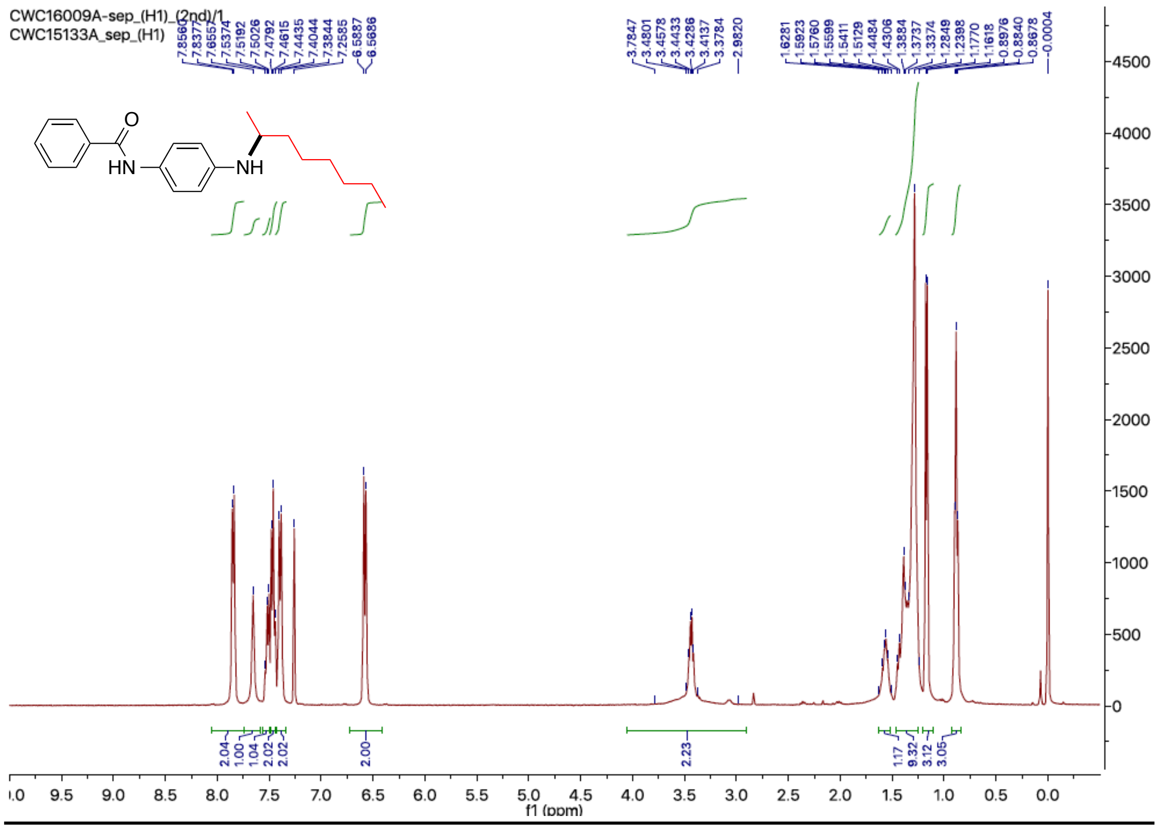

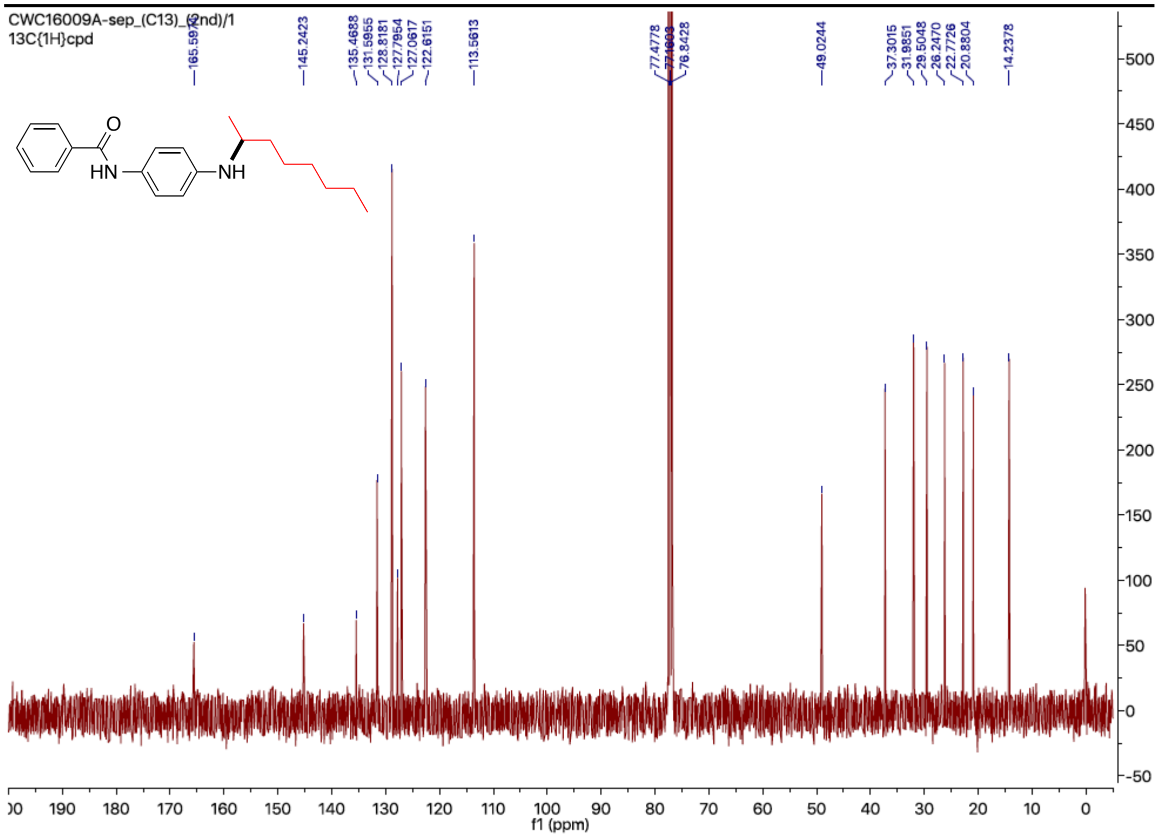

Supplementary Figure 27. ^{1}H and ^{13}C NMR spectra of *N*-(4-(Octan-2-ylamino)phenyl)benzamide (3r)

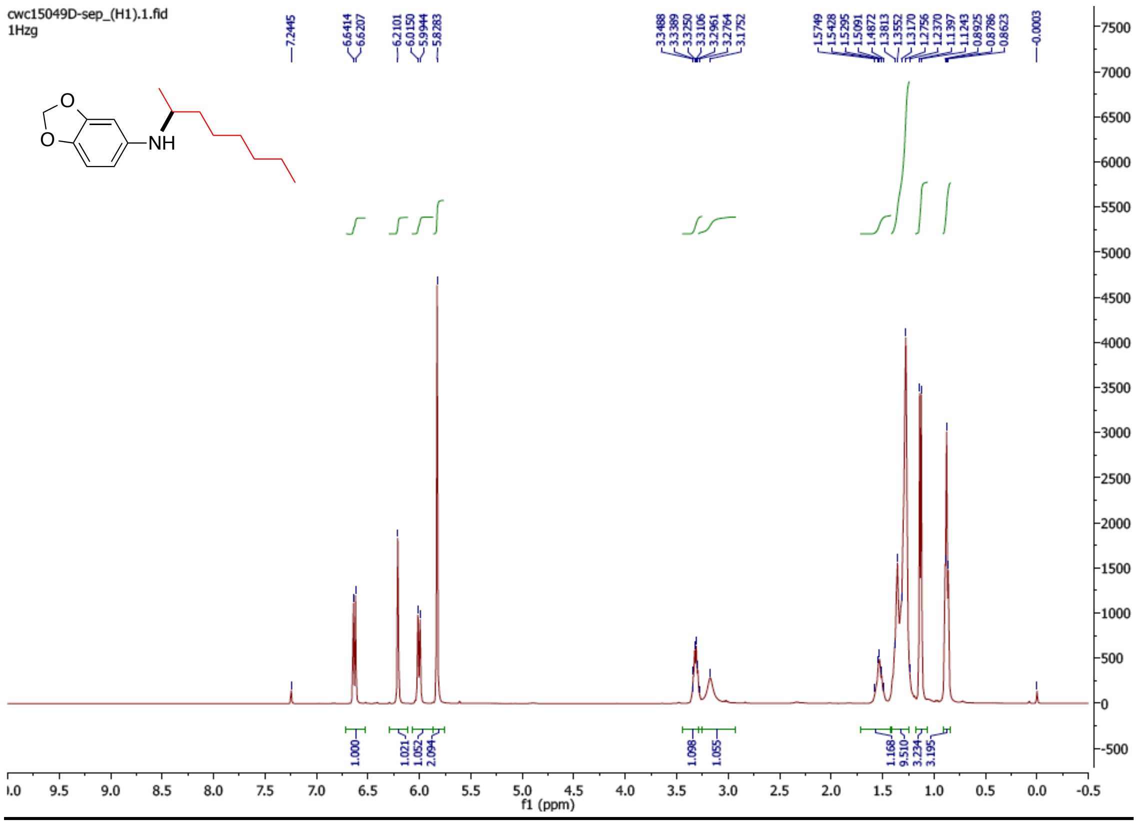

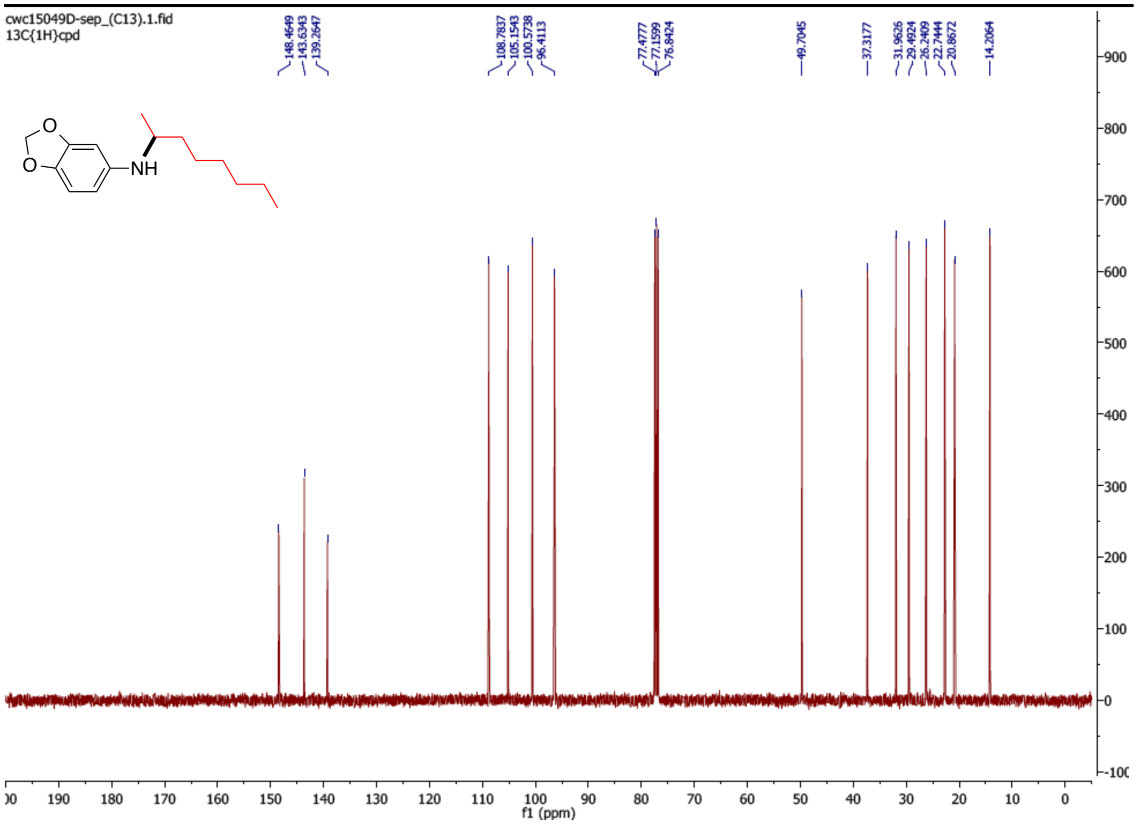

Supplementary Figure 28. ^{1}H and ^{13}C NMR spectra of *N*-(Octan-2-yl)benzo[*d*][1,3]dioxol-5-amine (3s)

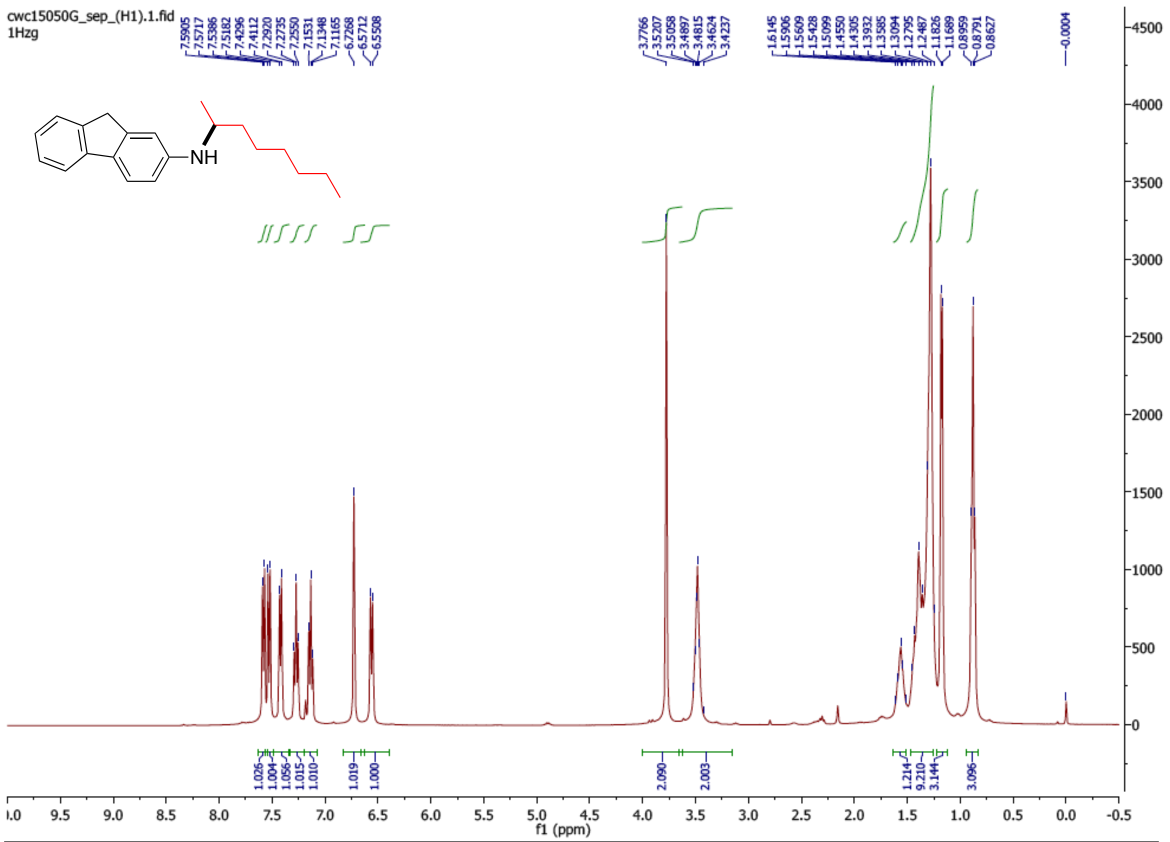

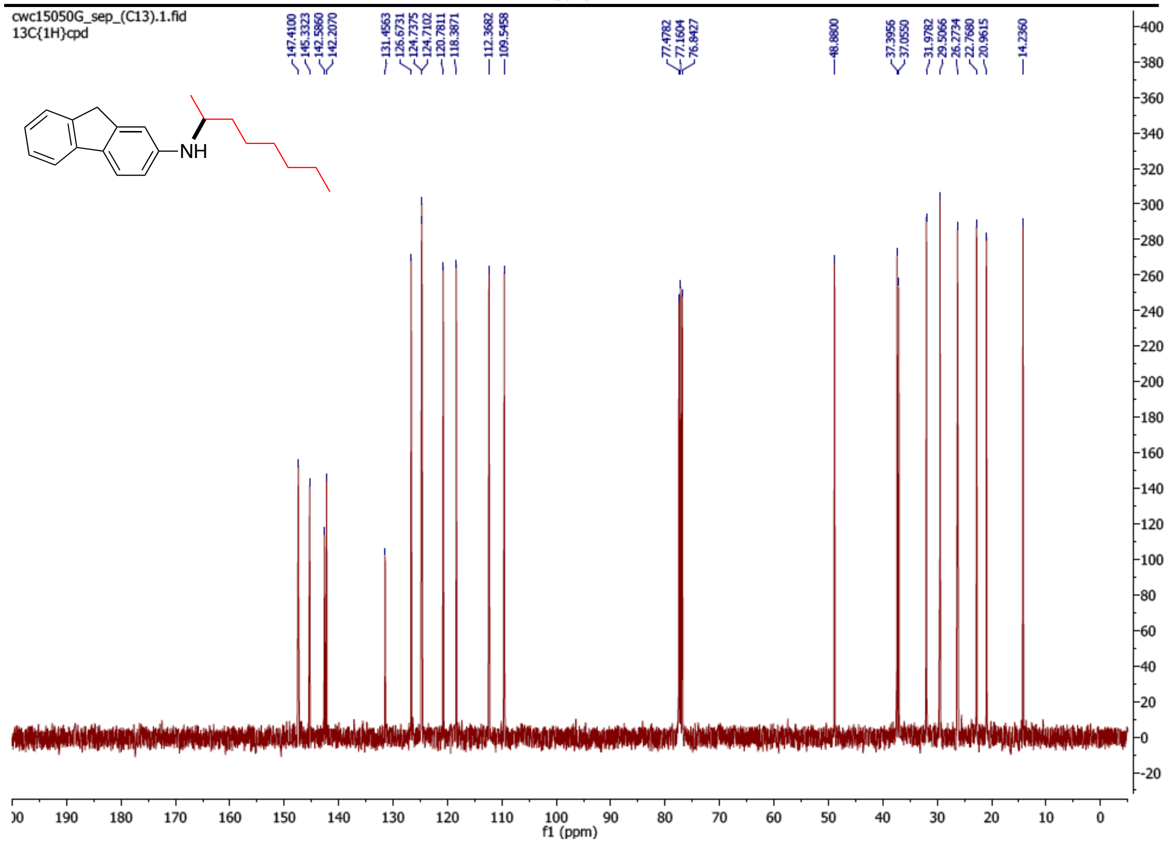

Supplementary Figure 29. ¹H and ¹³C NMR spectra of *N*-(Octan-2-yl)-9*H*-fluoren-2-amine (3t)

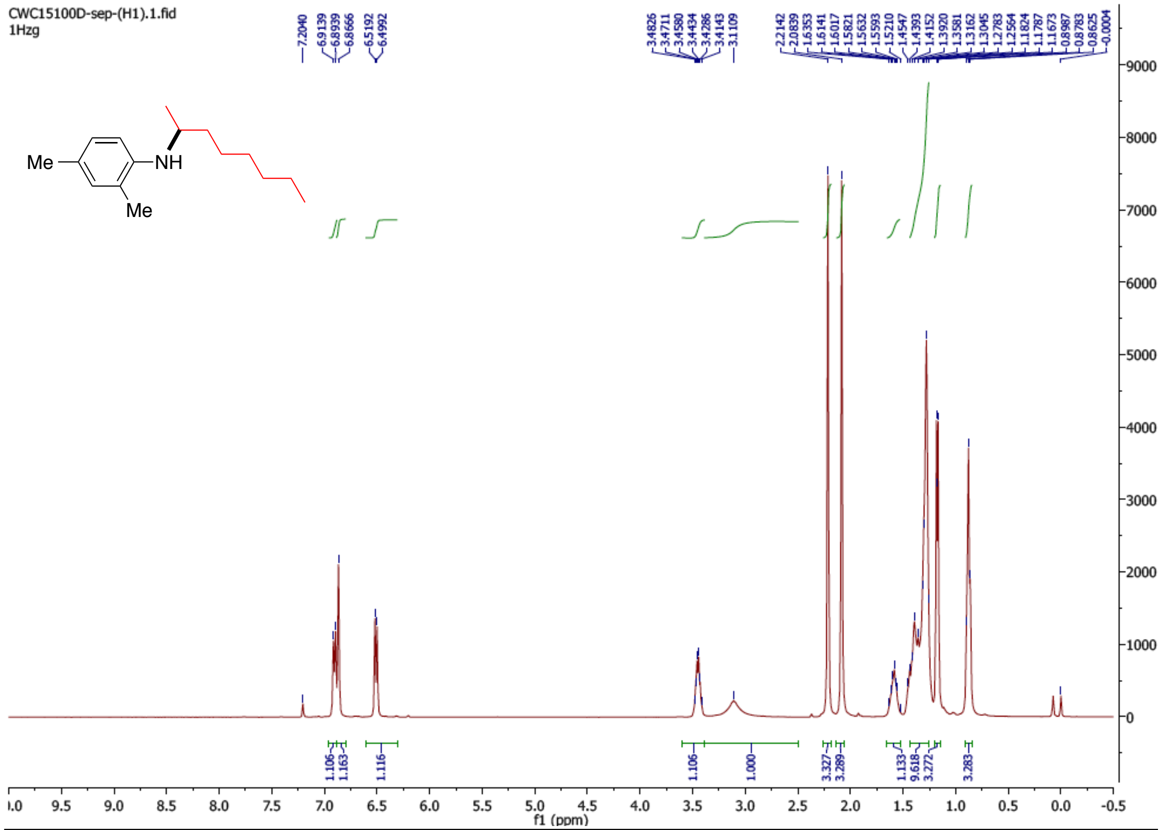

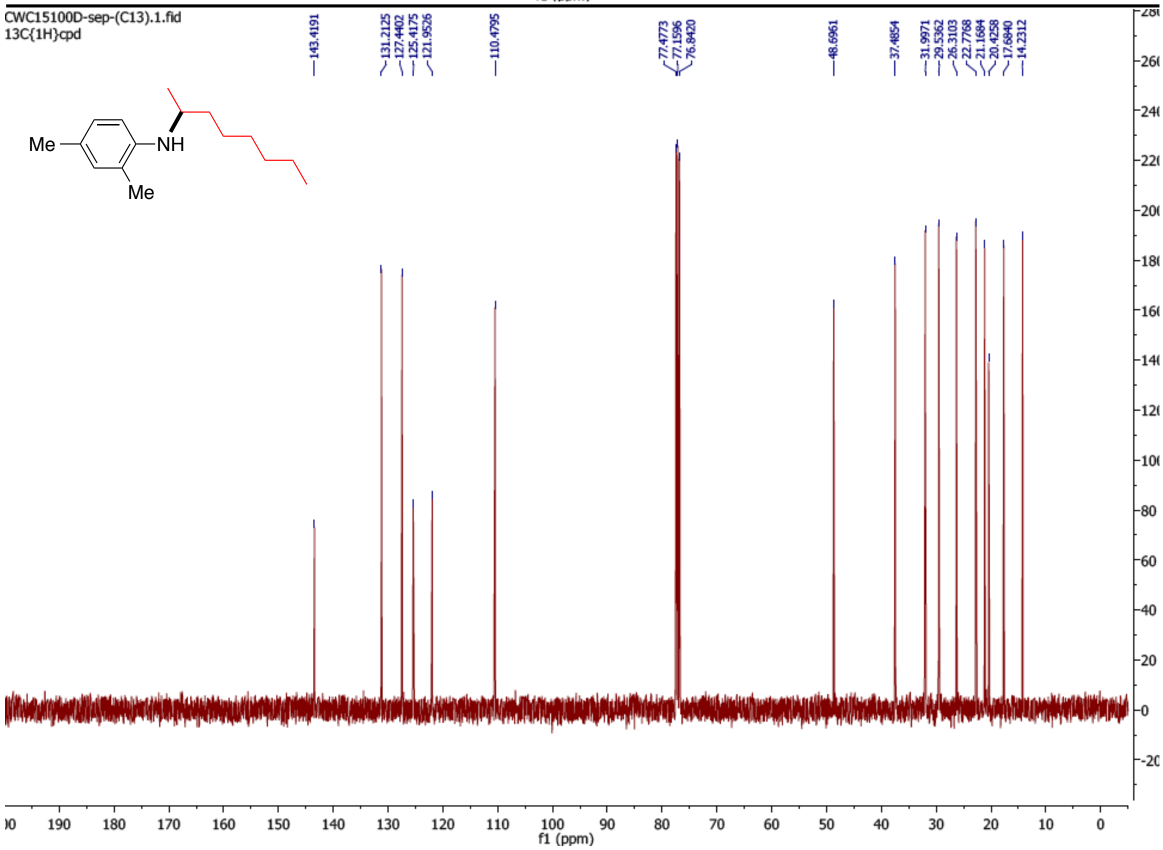

Supplementary Figure 30. ¹H and ¹³C NMR spectra of 2,4-Dimethyl-*N*-(octan-2-yl)aniline (3u)

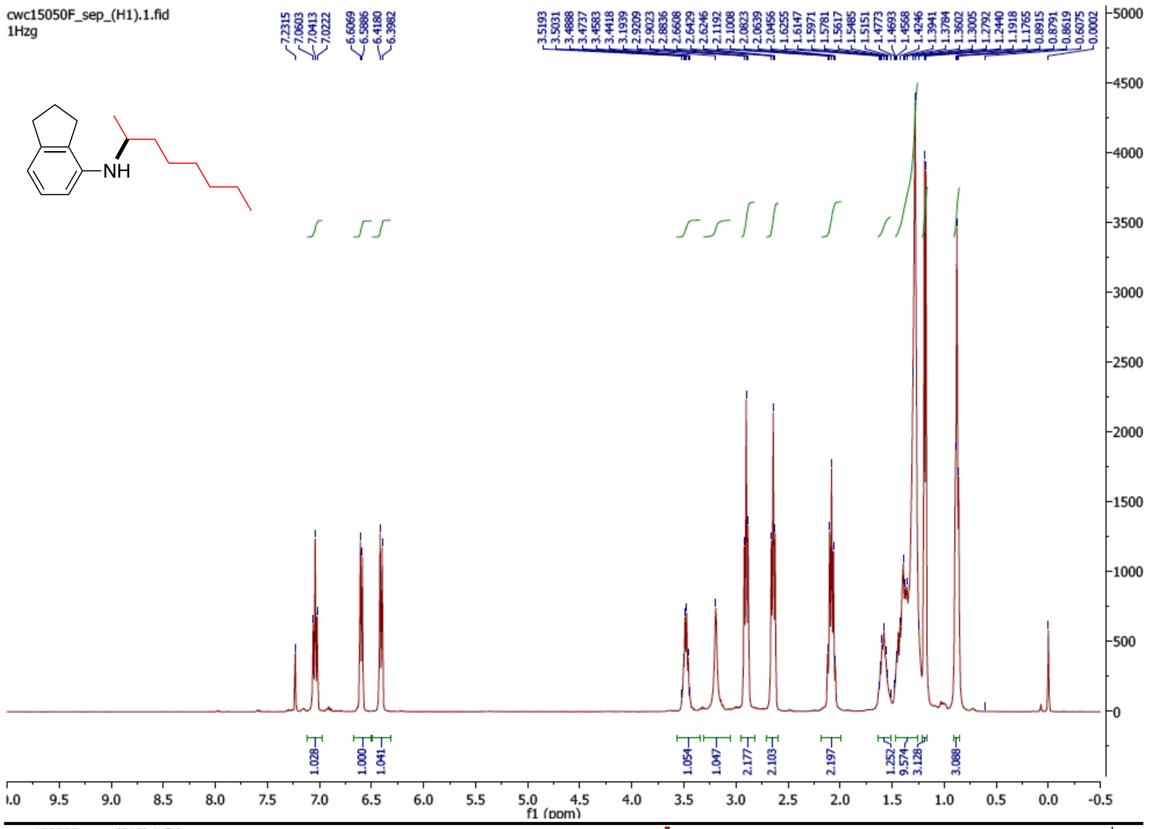

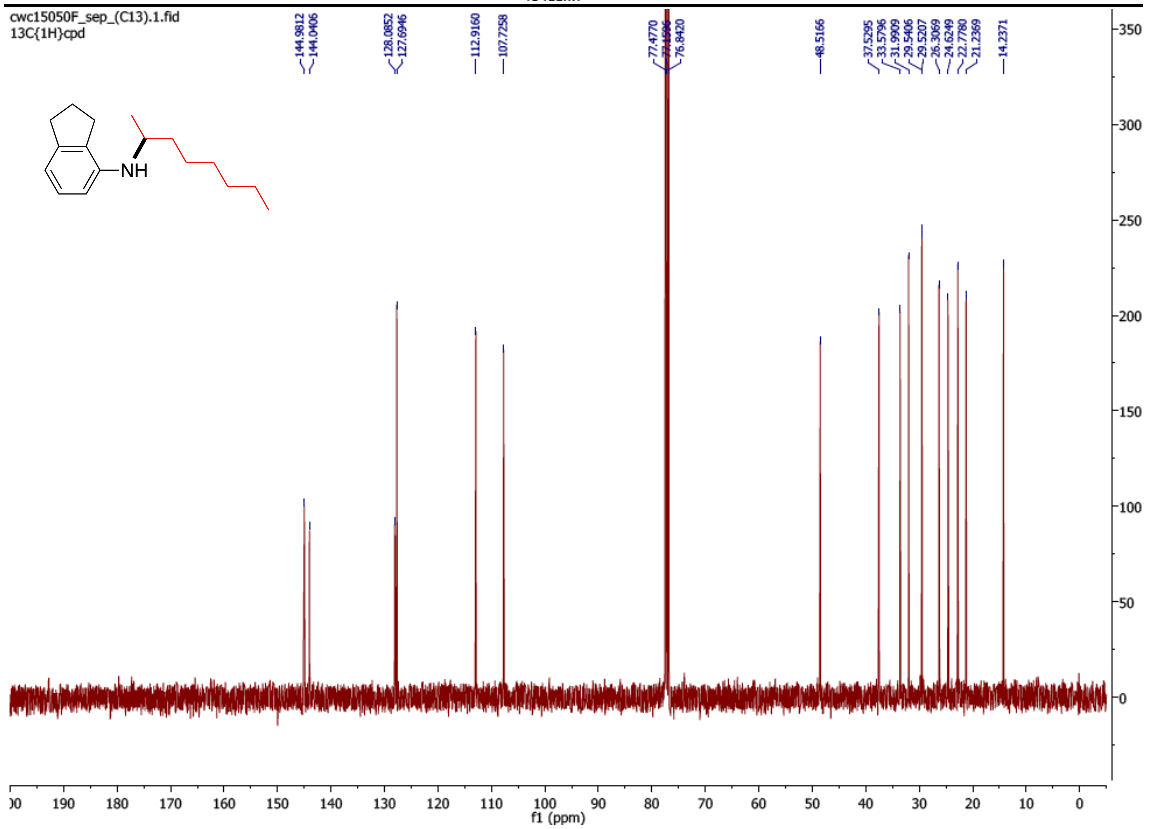

Supplementary Figure 31. ^{1}H and ^{13}C NMR spectra of *N*-(Octan-2-yl)-2,3-dihydro-1*H*-inden-4-amine (3v)

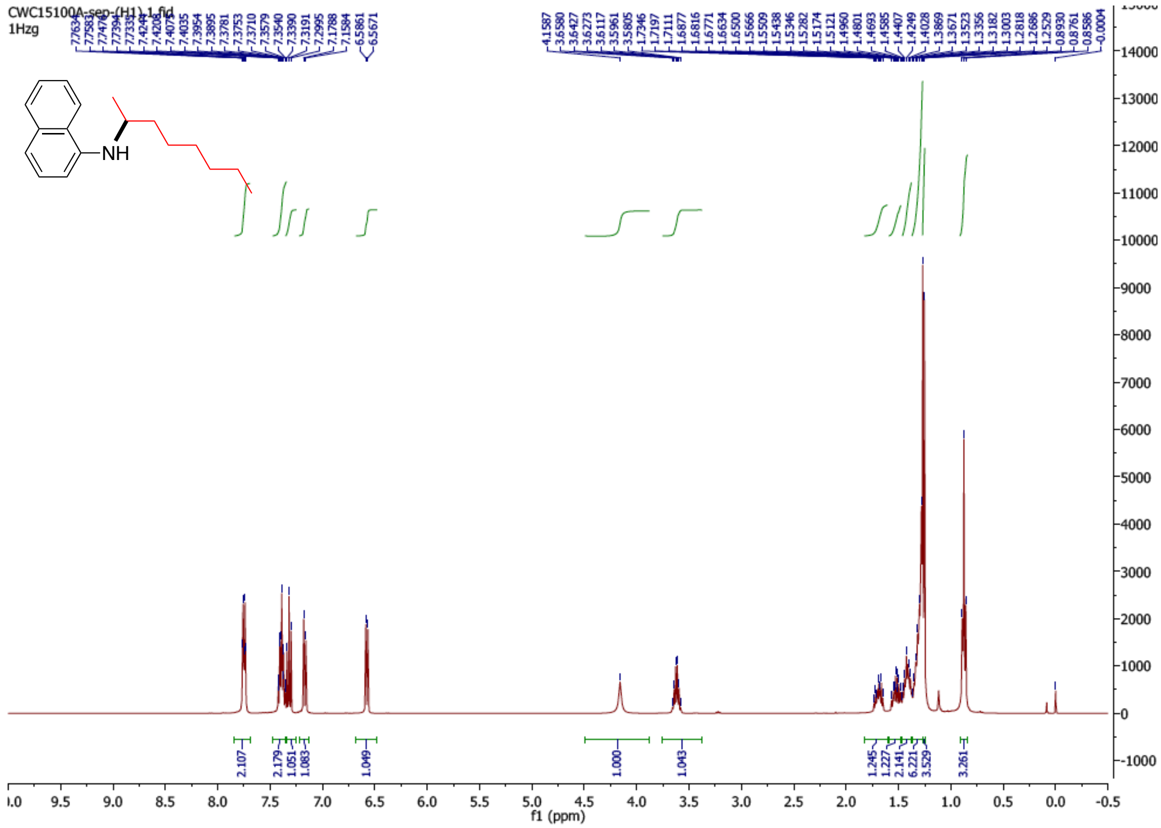

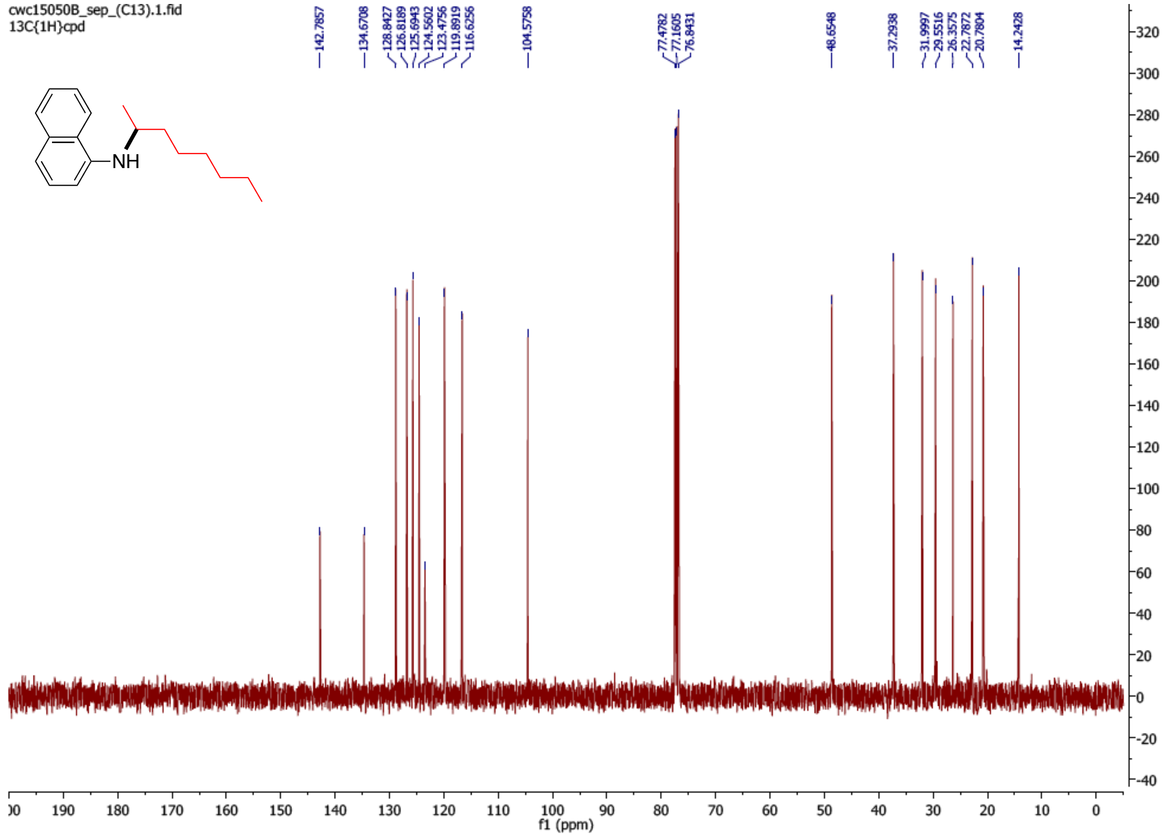

Supplementary Figure 32. ^{1}H and ^{13}C NMR spectra of *N*-(Octan-2-yl)naphthalen-1-amine (3w)

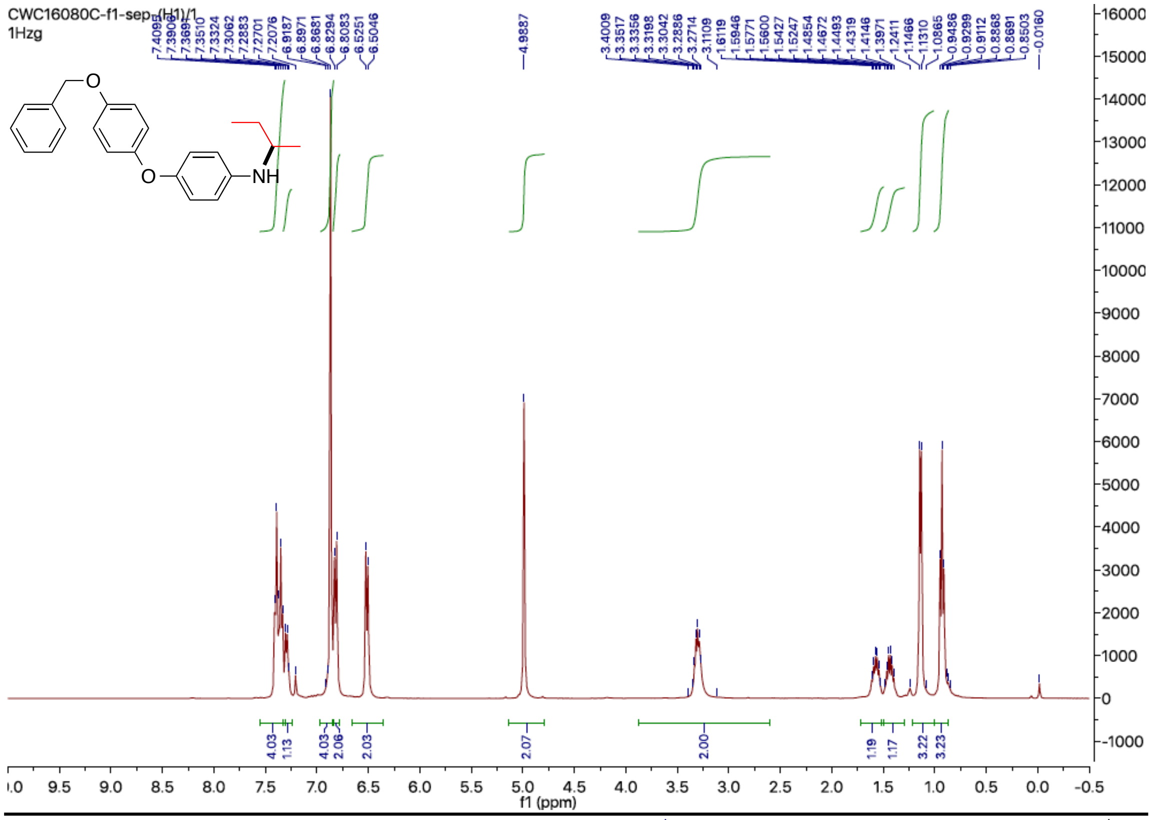

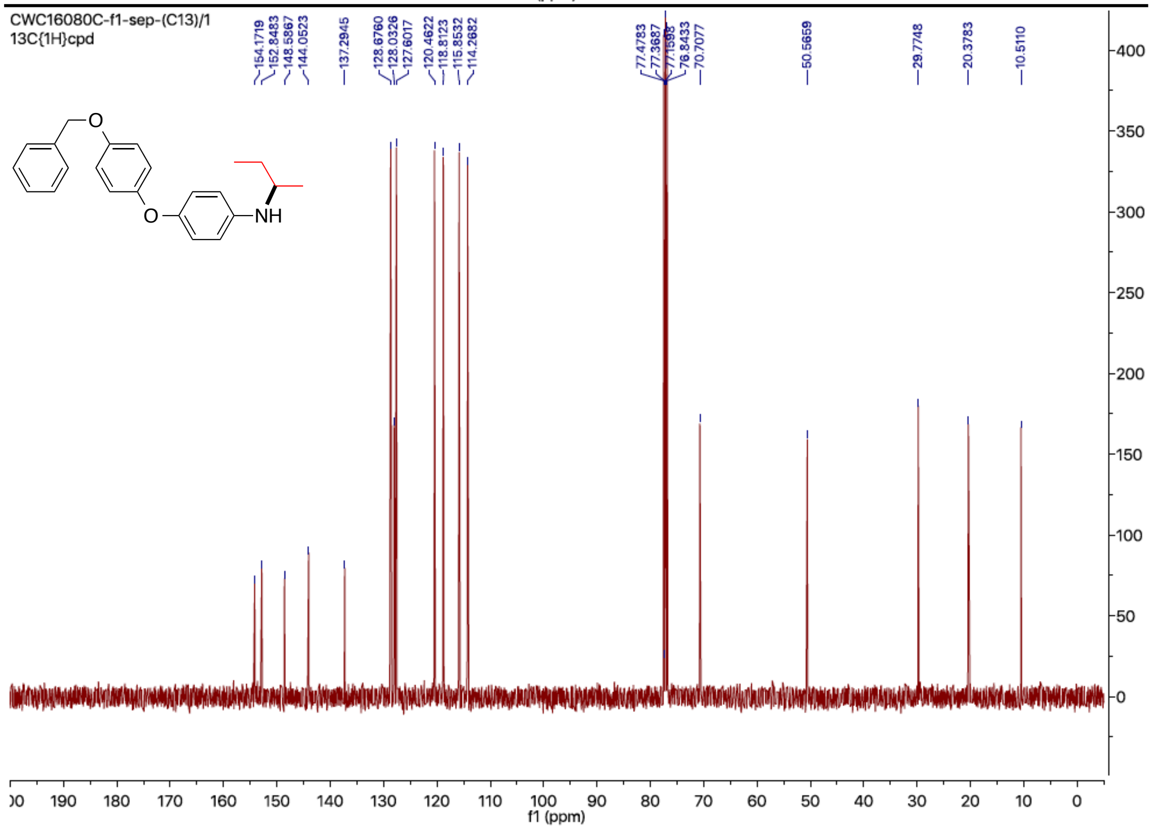

Supplementary Figure 33. ^{1}H and ^{13}C NMR spectra of 4-(4-(Benzyloxy)phenoxy)-*N*-(*sec*-butyl)aniline (3x)

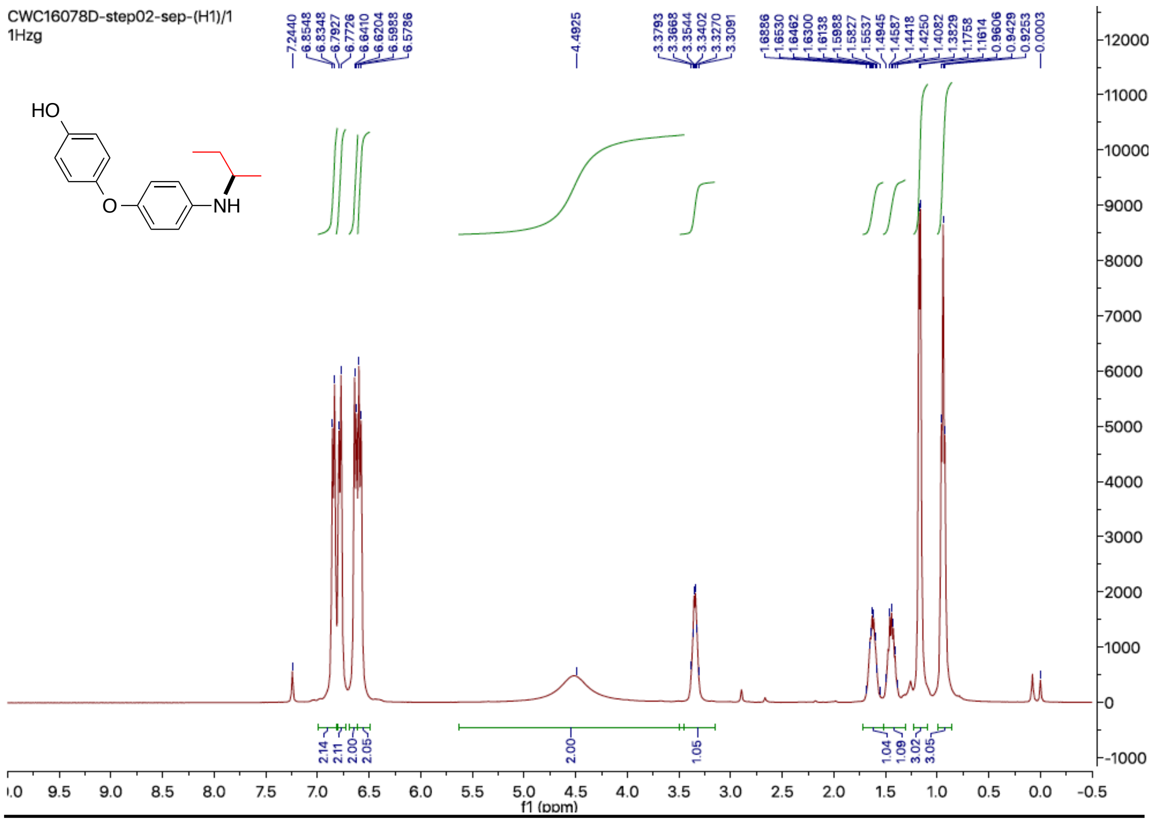

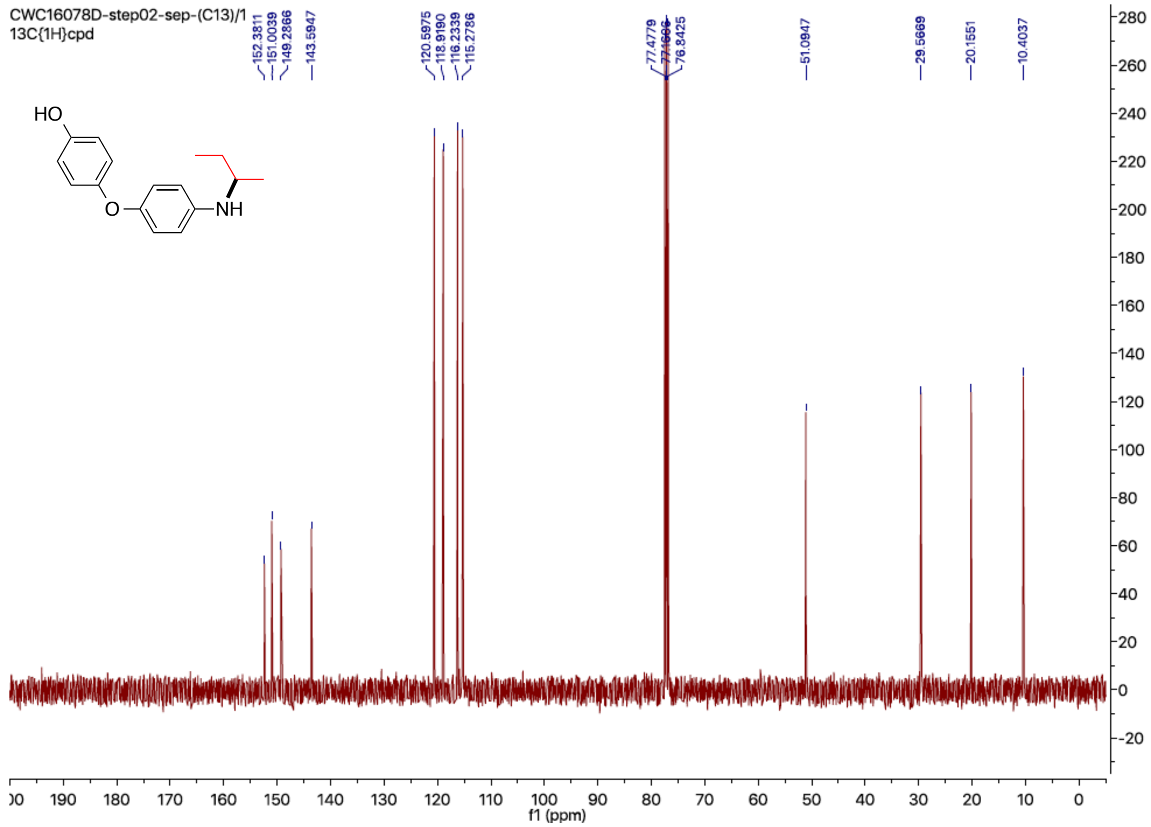

Supplementary Figure 34. ^{1}H and ^{13}C NMR spectra of 4-(4-(*sec*-butylamino)phenoxy)phenol (PD0084430, 3y)

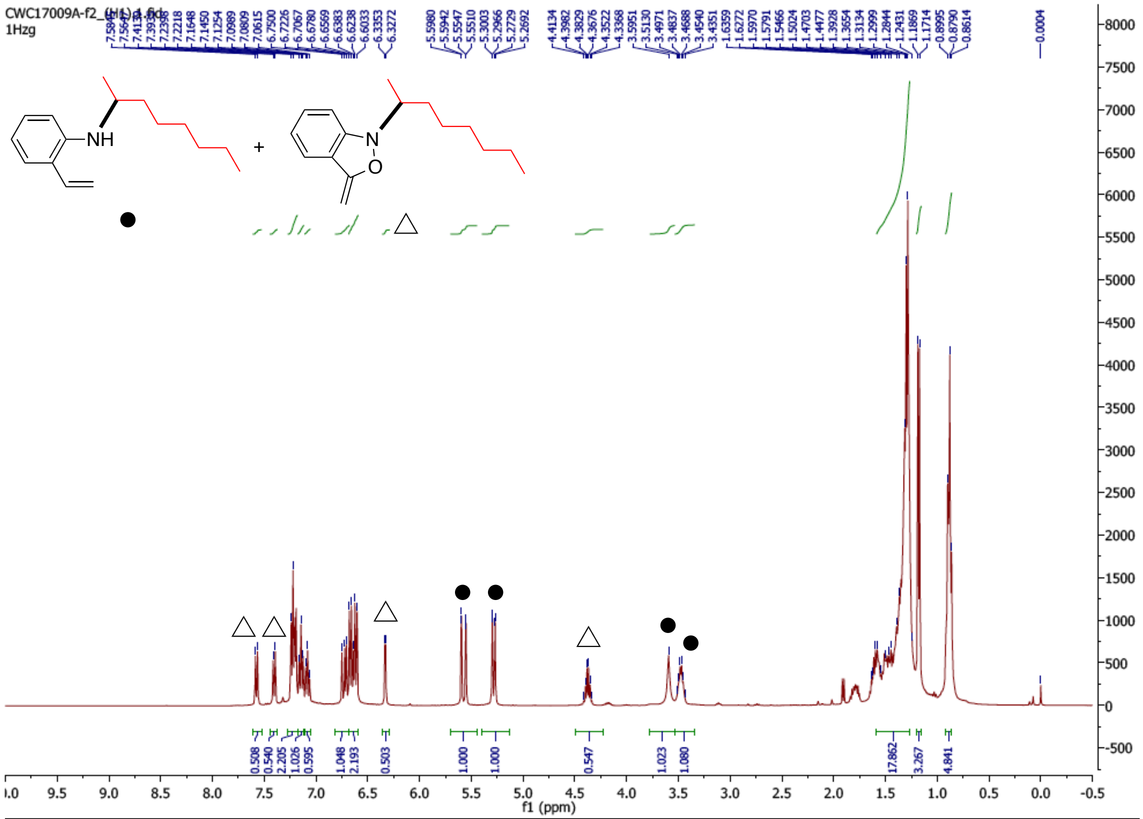

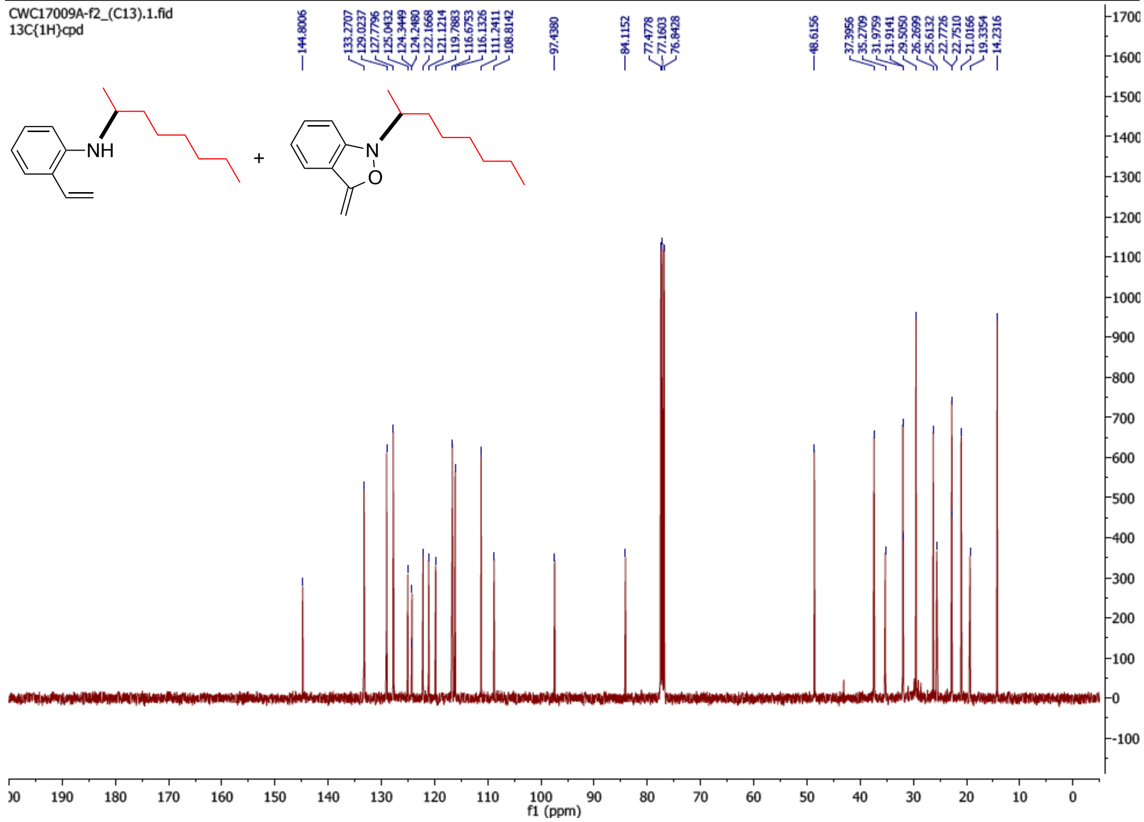

Supplementary Figure 35. ^{1}H and ^{13}C NMR spectra of *N*-(octan-2-yl)-2-vinylaniline (3z) and 3-methylene-1-(octan-2-yl)-1,3-dihydrobenzo[*c*]isoxazole (3z')

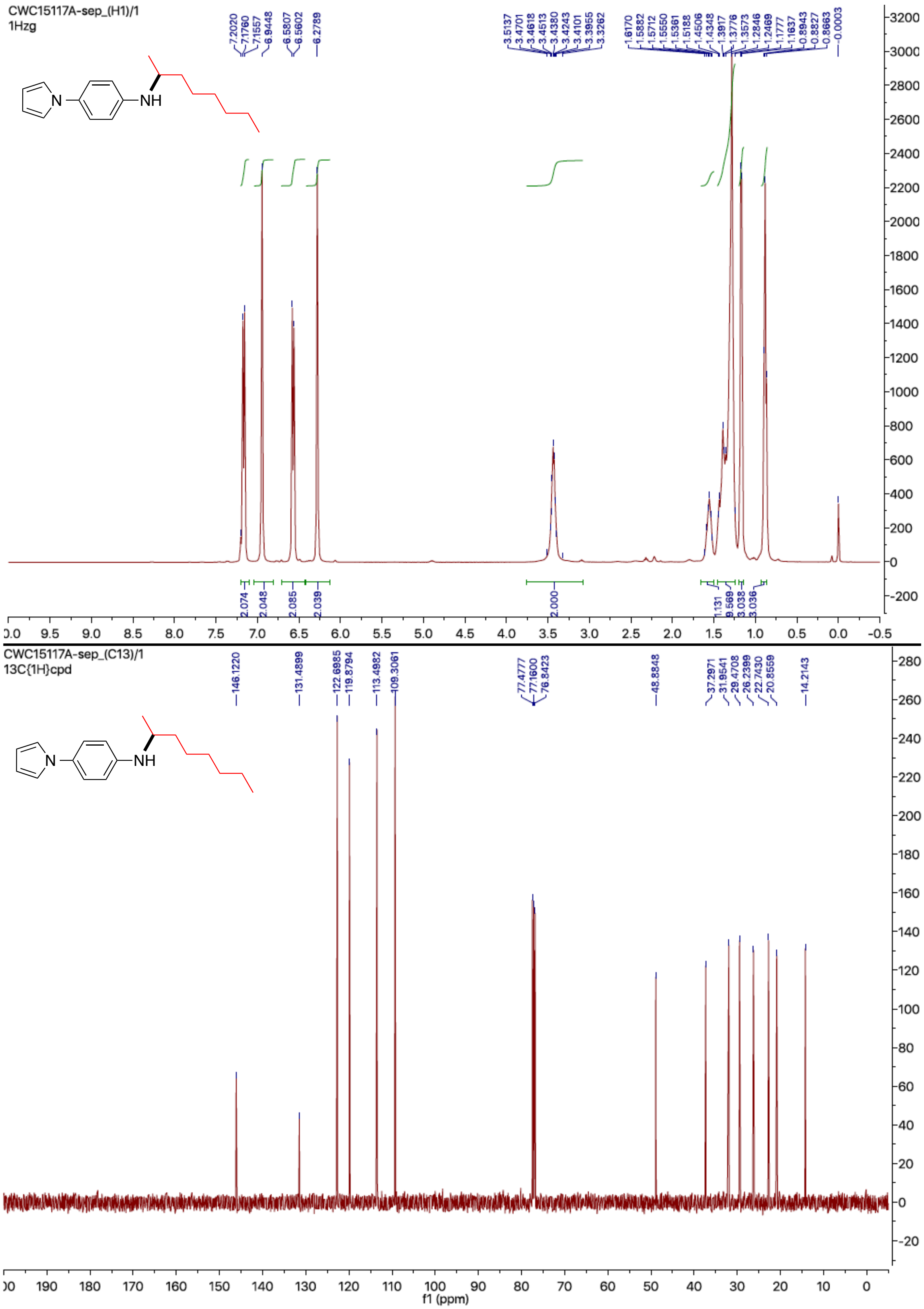

Supplementary Figure 36. ^{1}H and ^{13}C NMR spectra of *N*-(Octan-2-yl)-4-(1*H*-pyrrol-1-yl)aniline (4a)

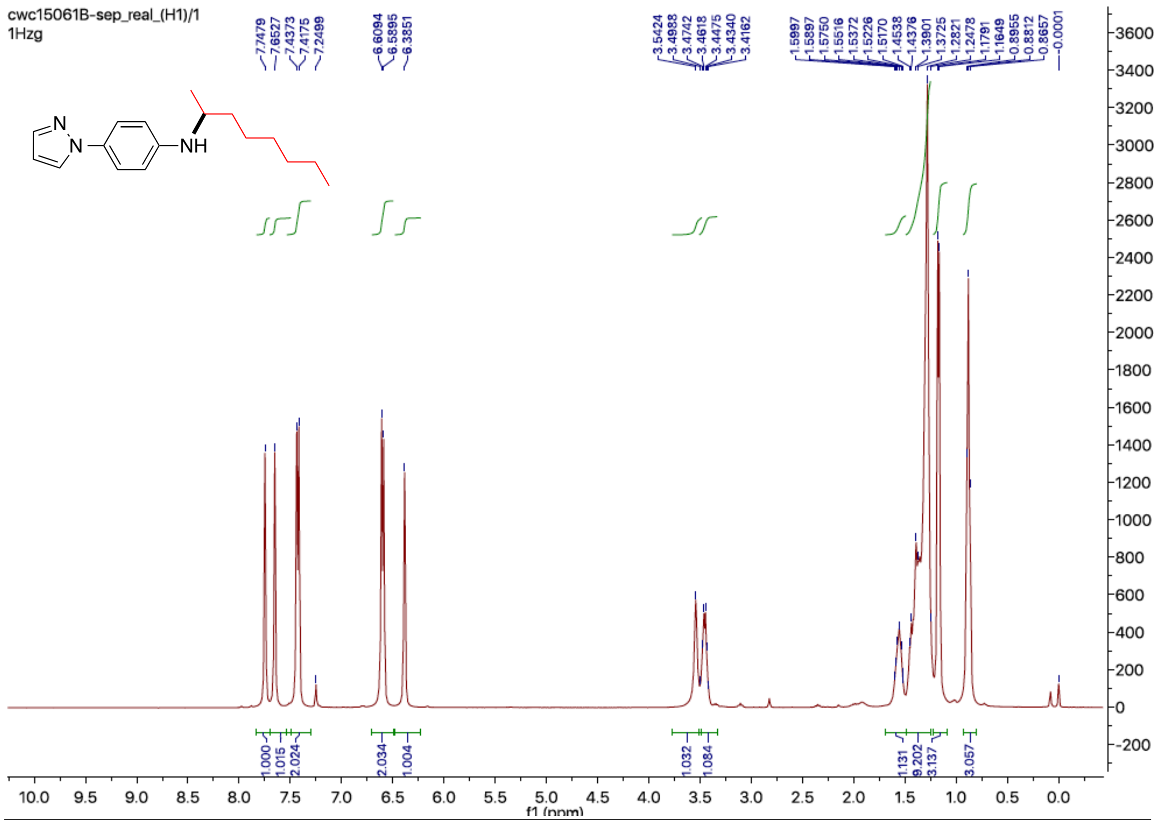

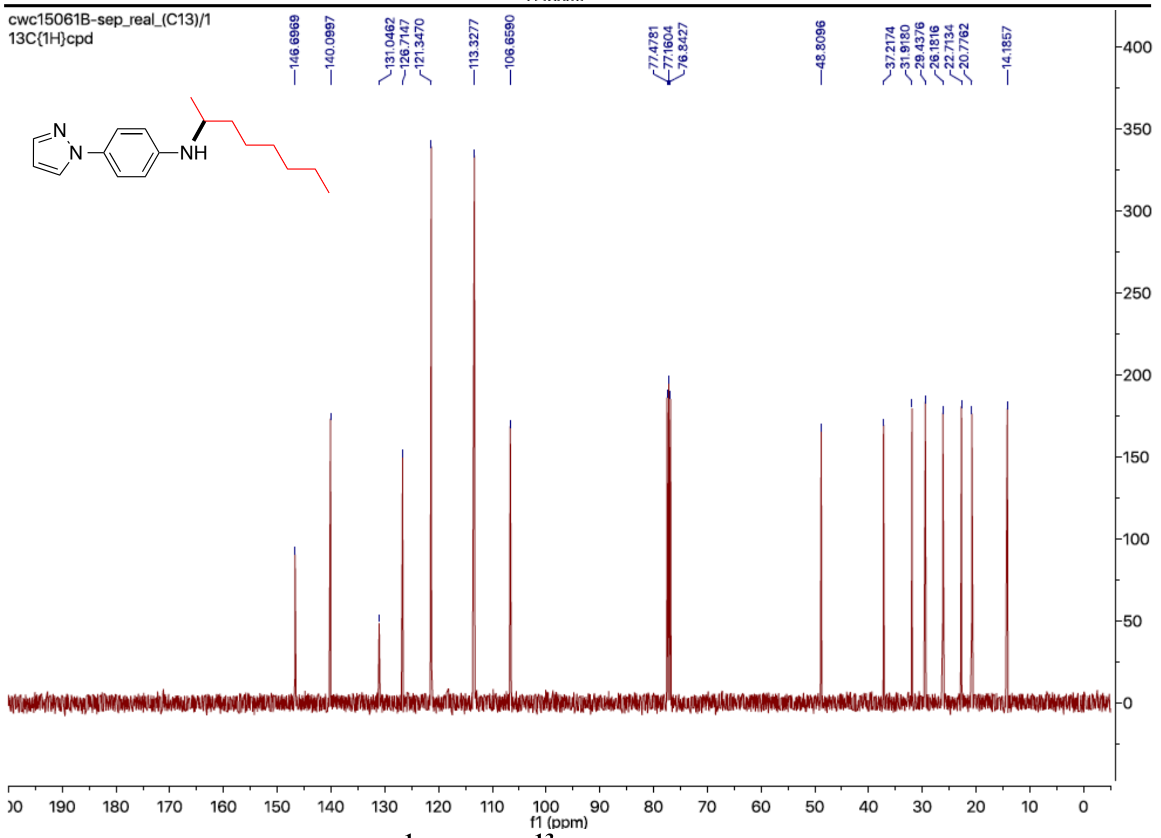

Supplementary Figure 37. ^{1}**H and** ^{13}**C NMR spectra of *N*-(Octan-2-yl)-4-(1*H*-pyrazol-1-yl)aniline (4b)**

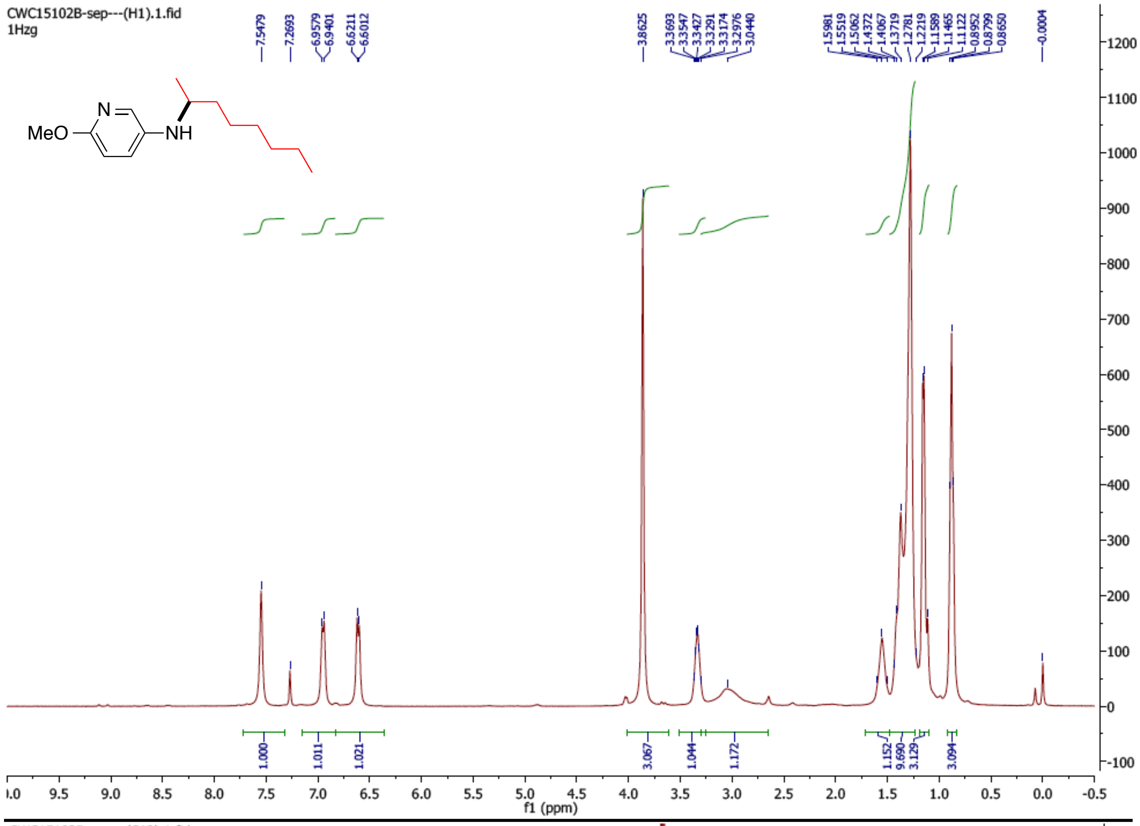

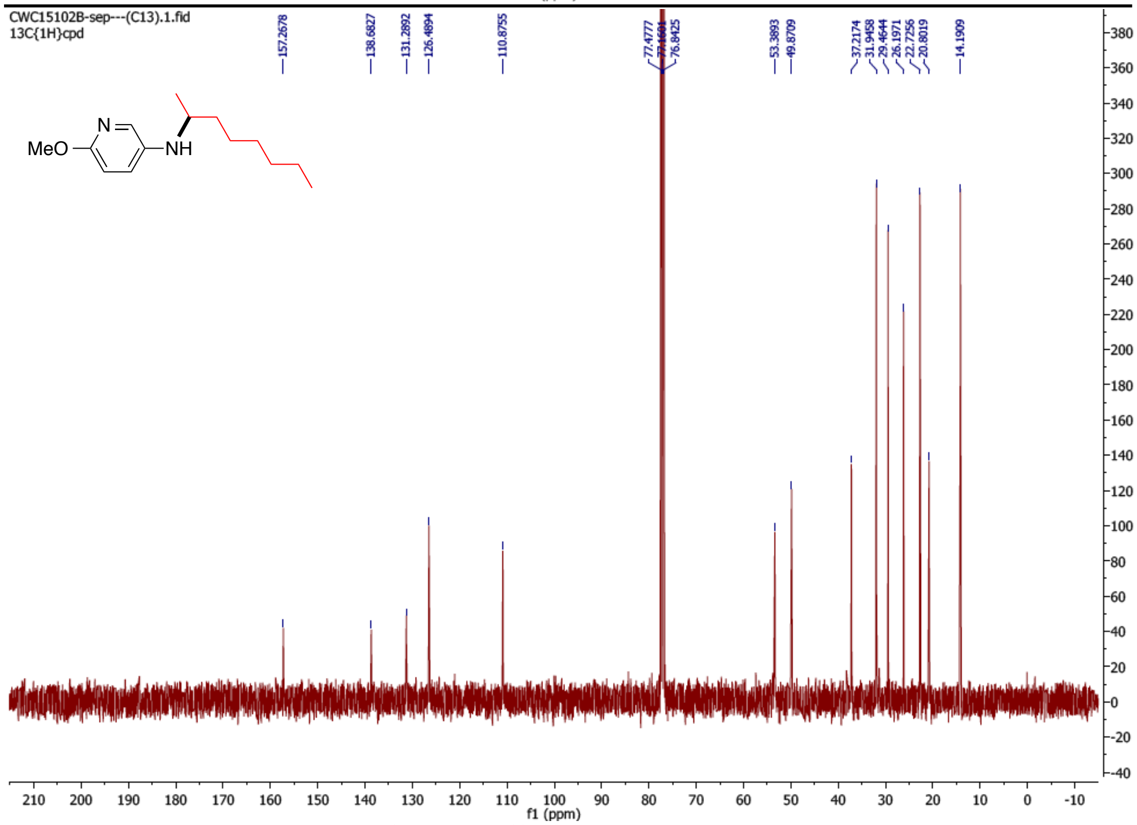

Supplementary Figure 38. ^{1}H and ^{13}C NMR spectra of 6-Methoxy-*N*-(octan-2-yl)pyridin-3-amine (4c)

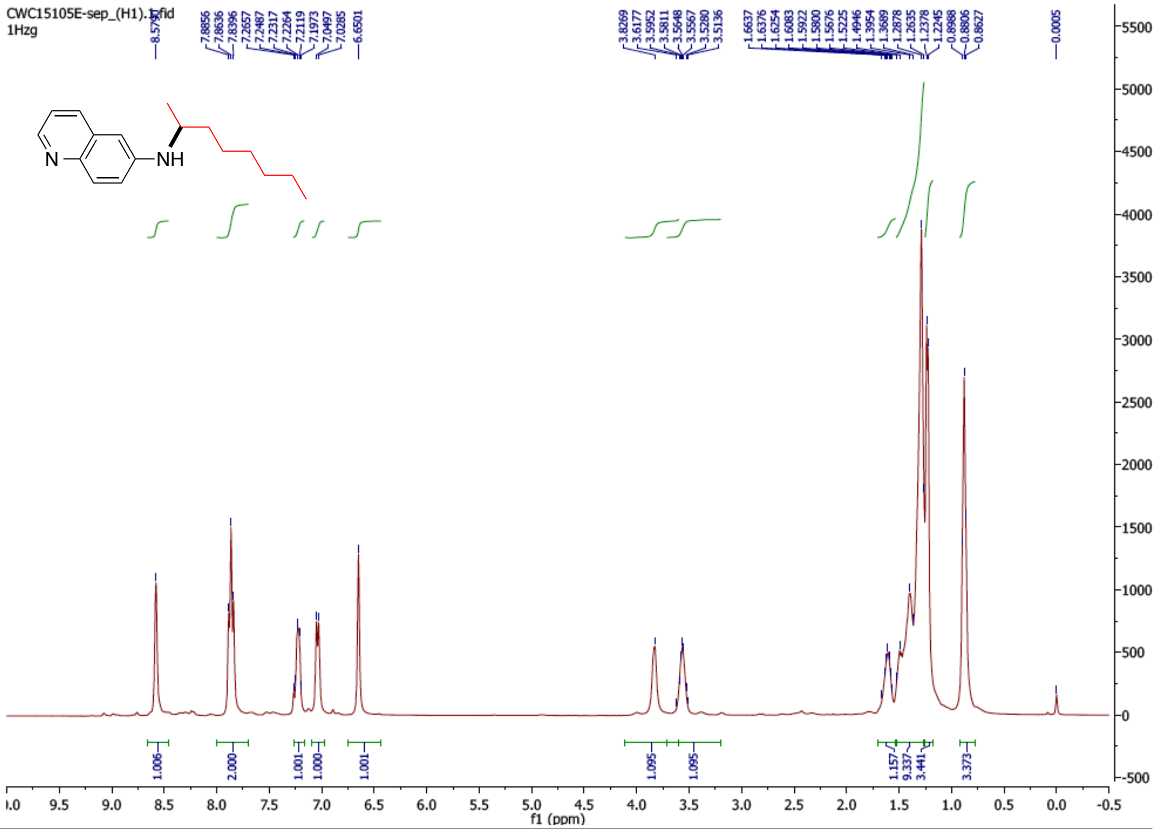

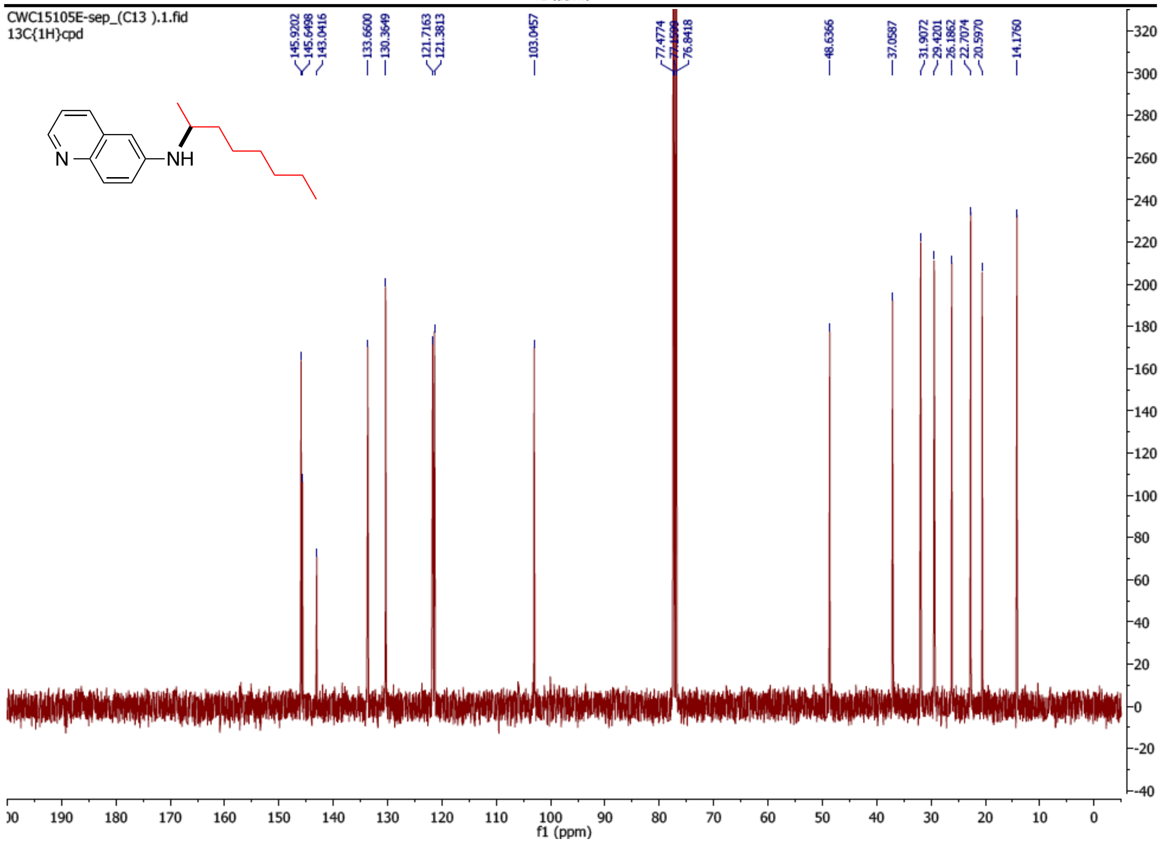

Supplementary Figure 39. ^{1}H and ^{13}C NMR spectra of *N*-(Octan-2-yl)quinolin-6-amine (4d)

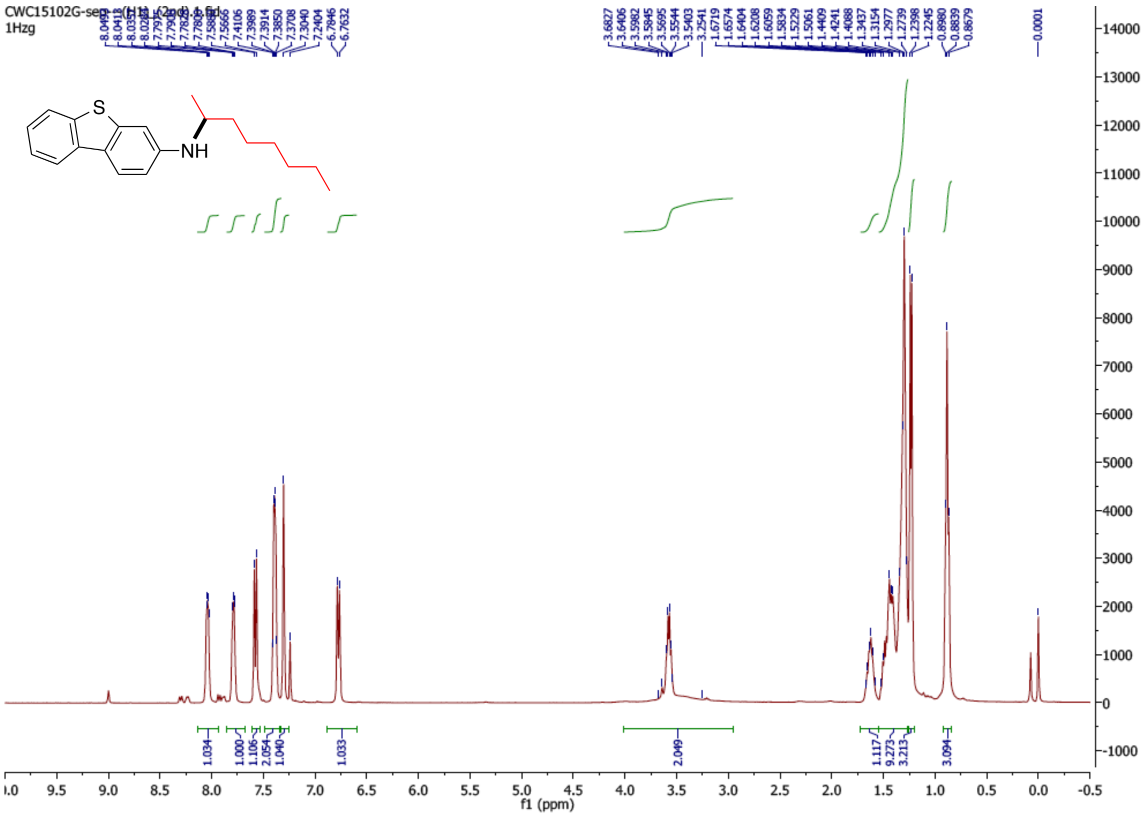

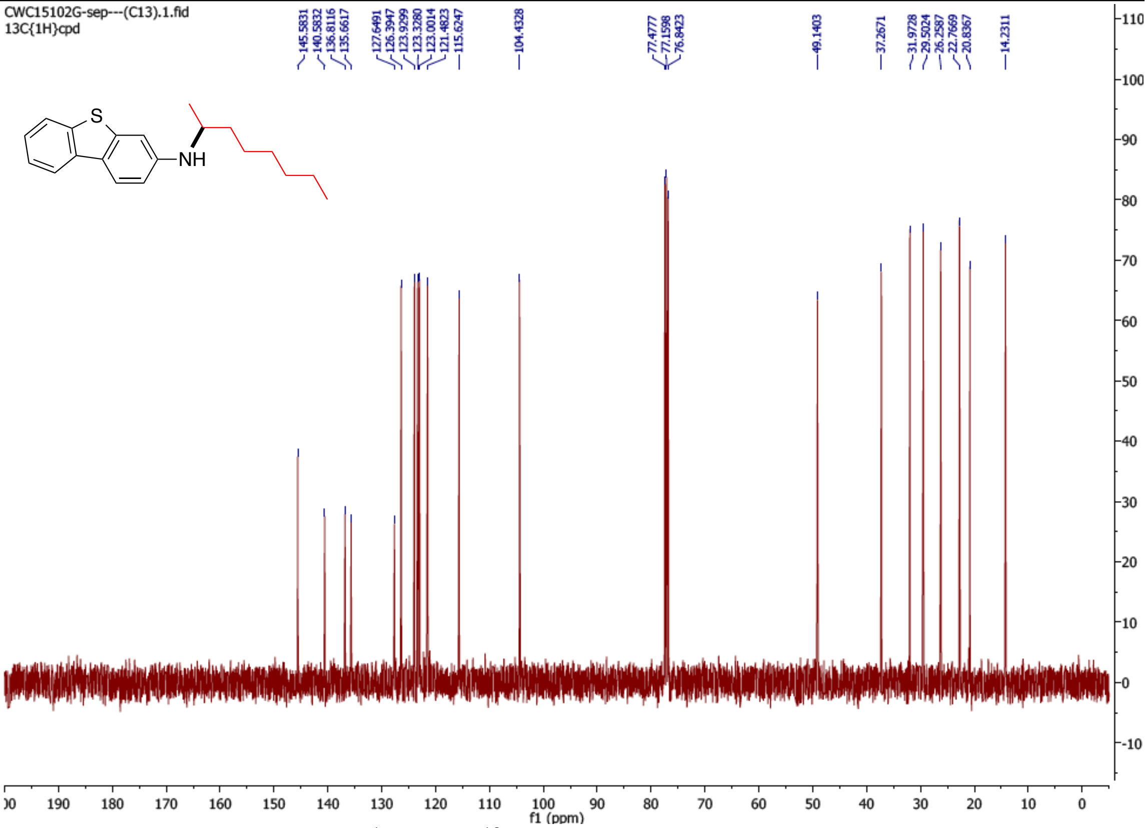

Supplementary Figure 40. ^{1}H and ^{13}C NMR spectra of *N*-(Octan-2-yl)dibenzo[*b*,*d*]thiophen-3-amine (4e)

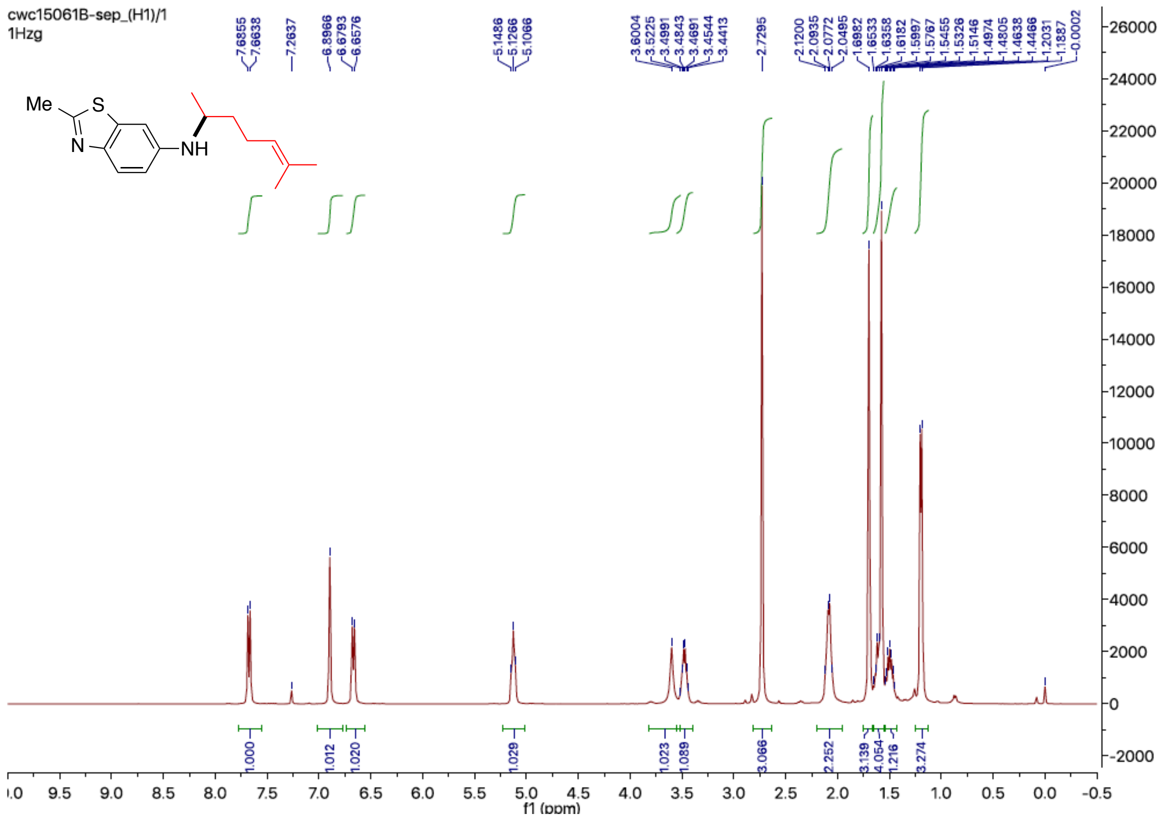

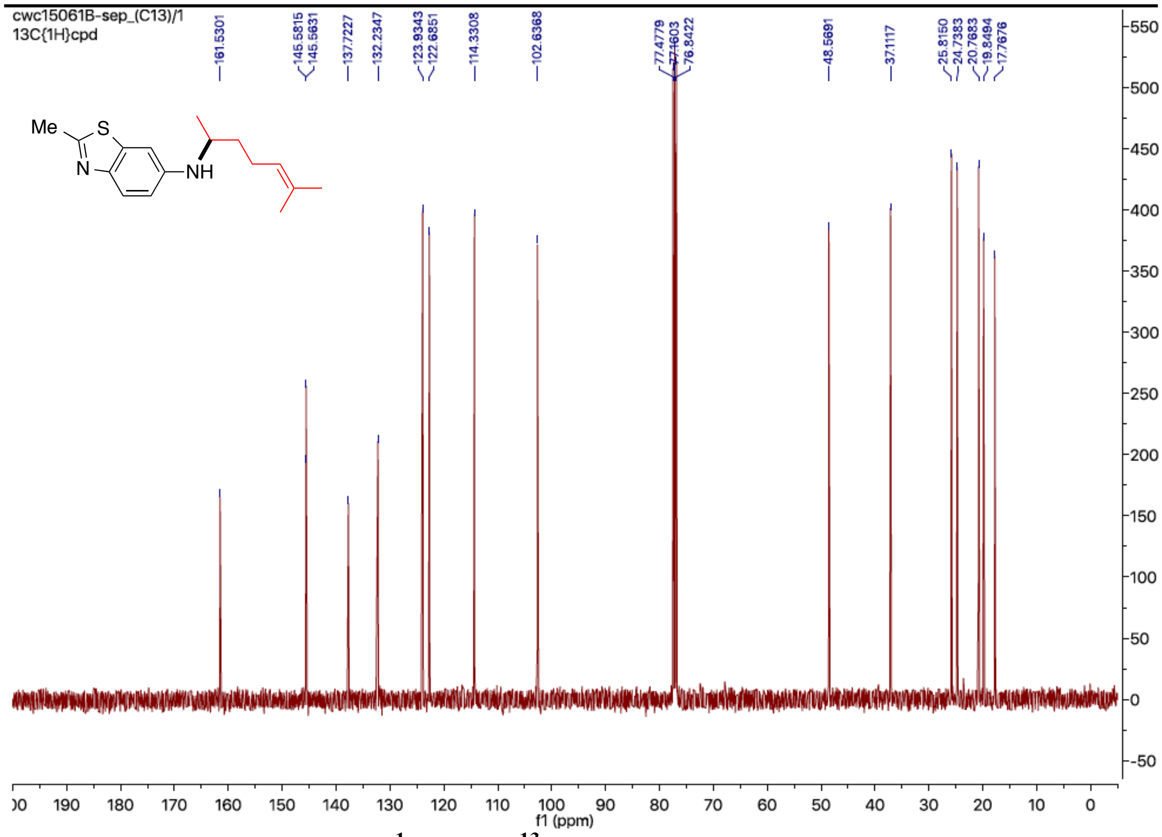

Supplementary Figure 41. ^{1}H and ^{13}C NMR spectra of 2-Methyl-*N*-(6-methylhept-5-en-2-yl)benzo[*d*]thiazol-6-amine (4f)

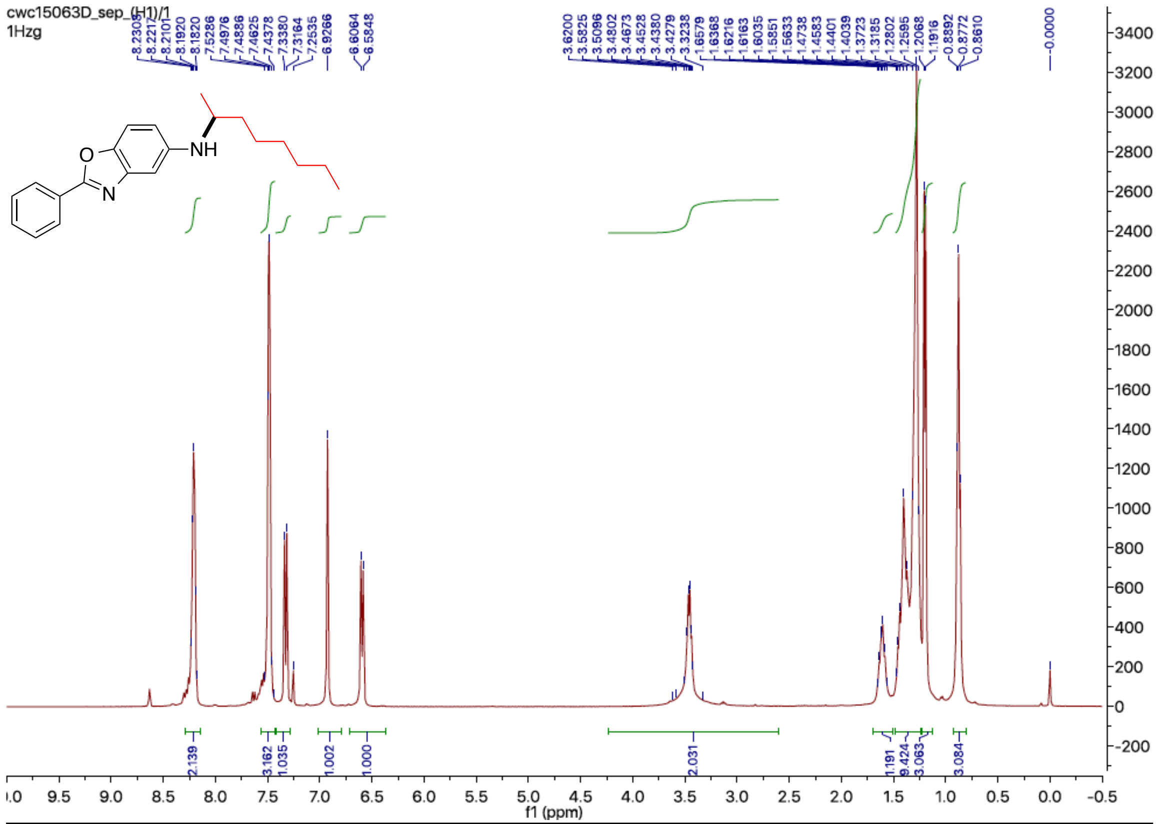

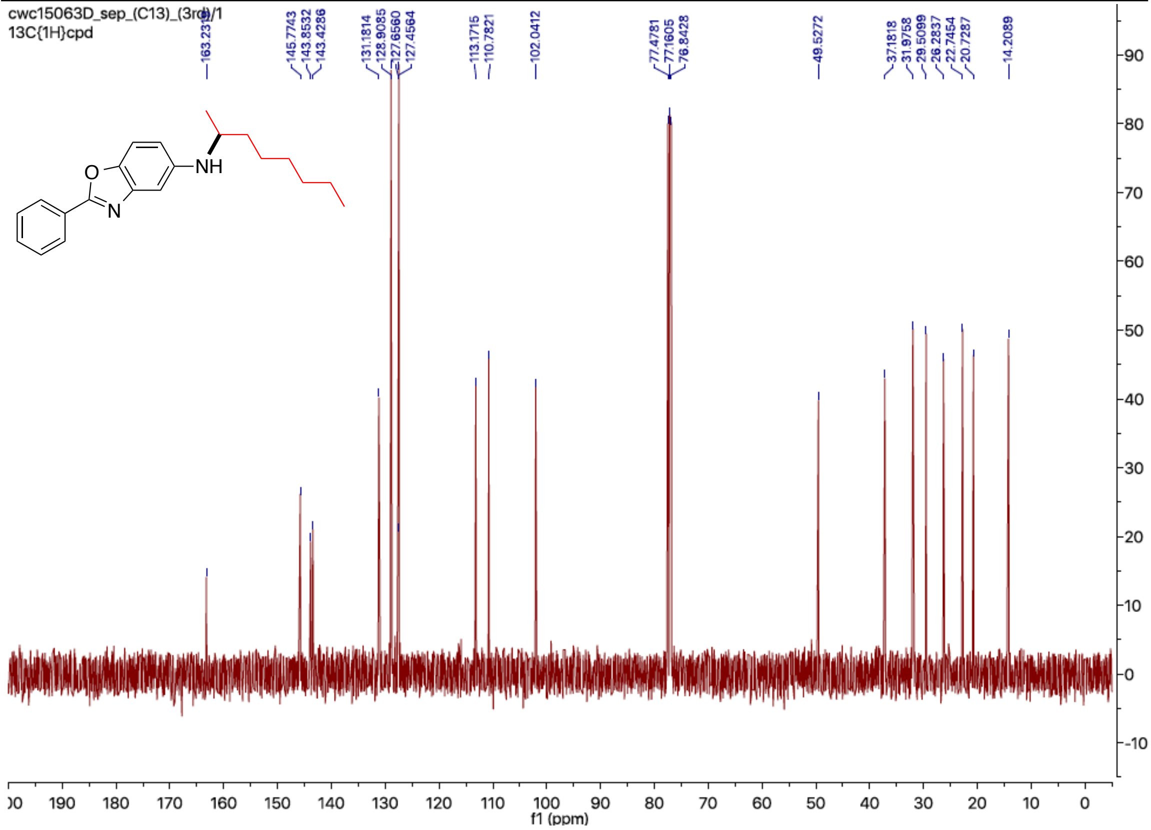

Supplementary Figure 42. ^{1}H and ^{13}C NMR spectra of *N*-(Octan-2-yl)-2-phenylbenzo[*d*]oxazol-5-amine (4g)

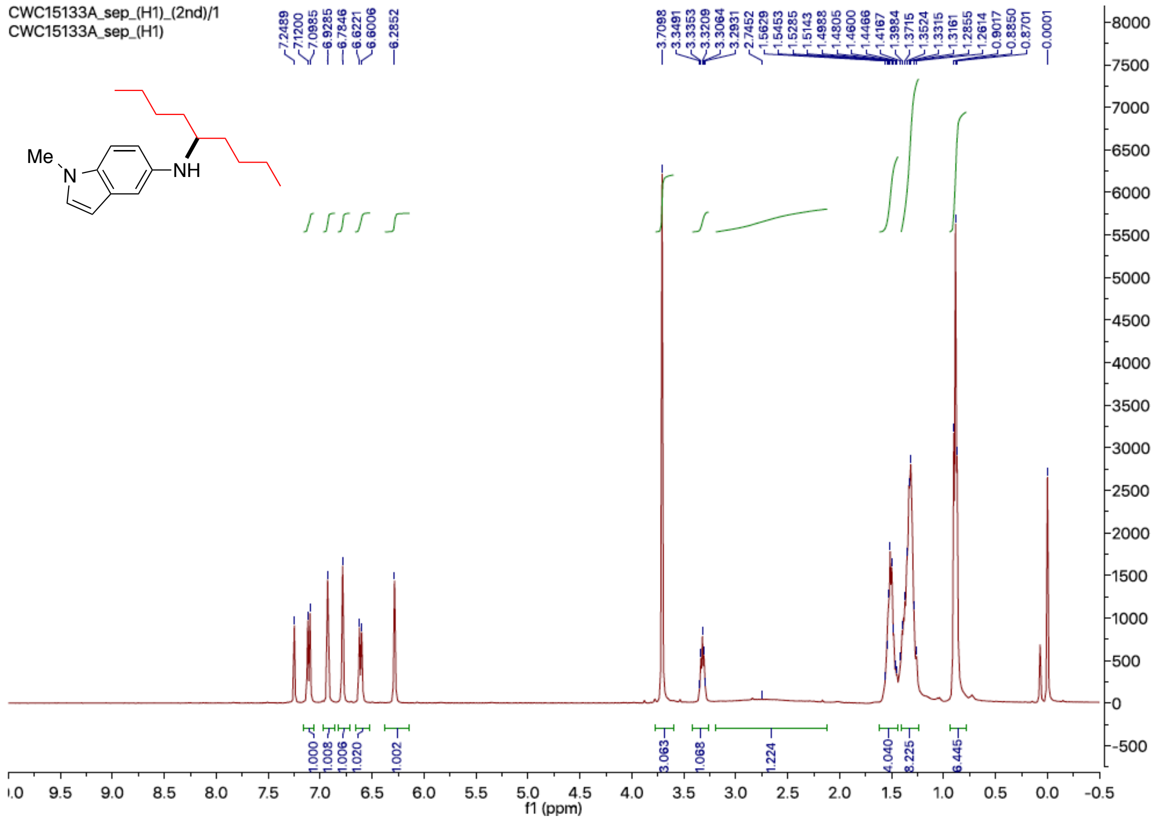

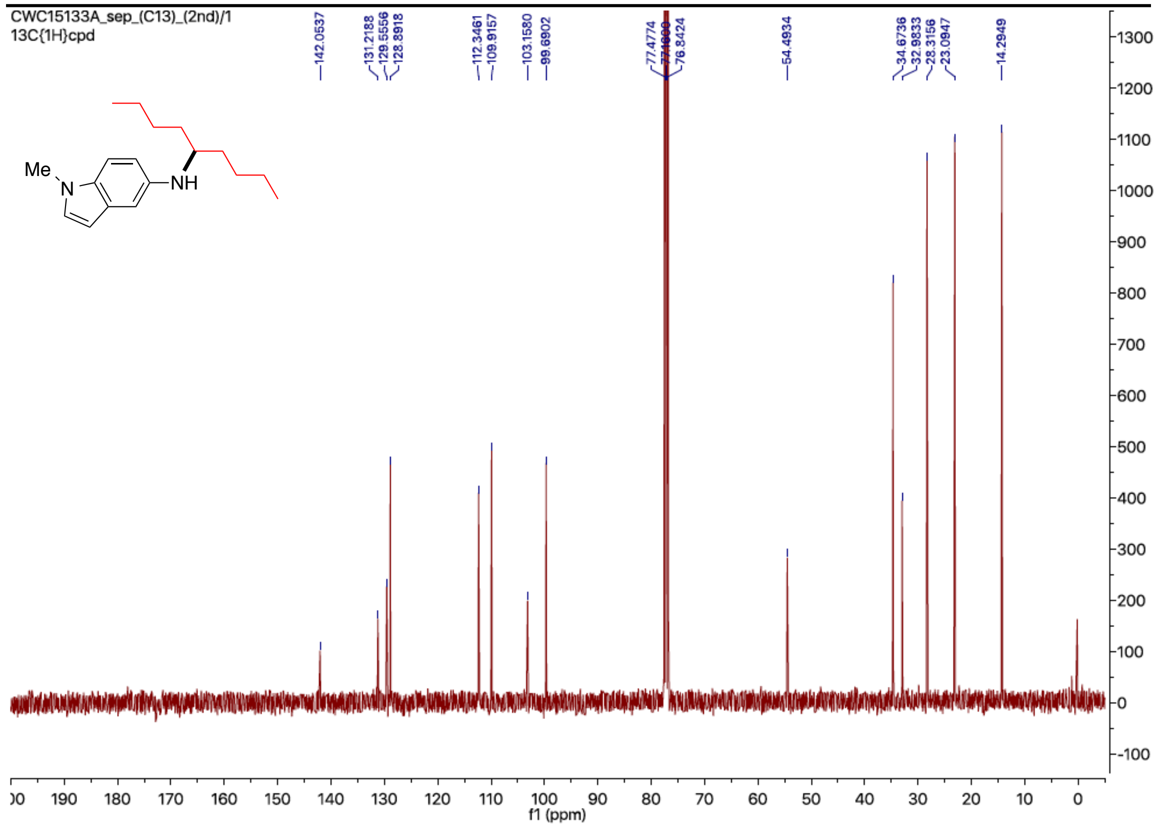

Supplementary Figure 43. ^{1}H and ^{13}C NMR spectra of 1-Methyl-*N*-(nonan-5-yl)-1*H*-indol-5-amine (4h)

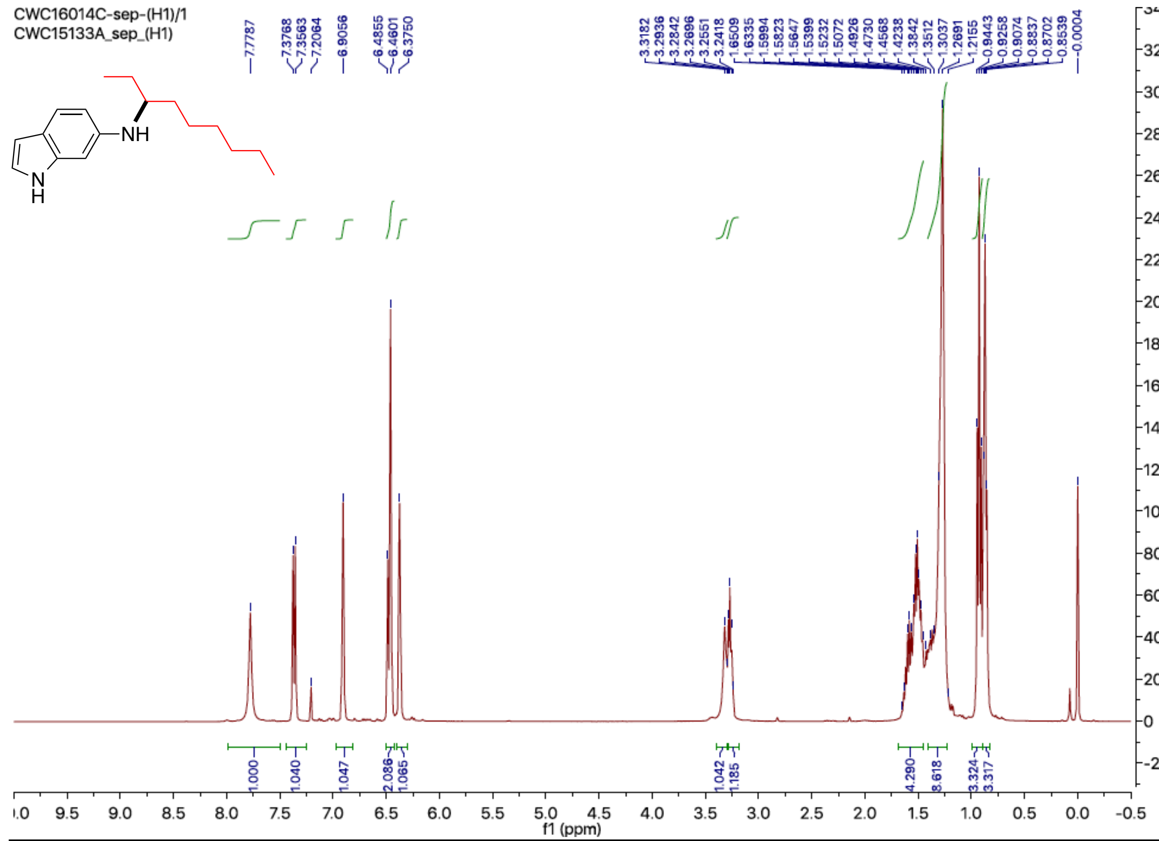

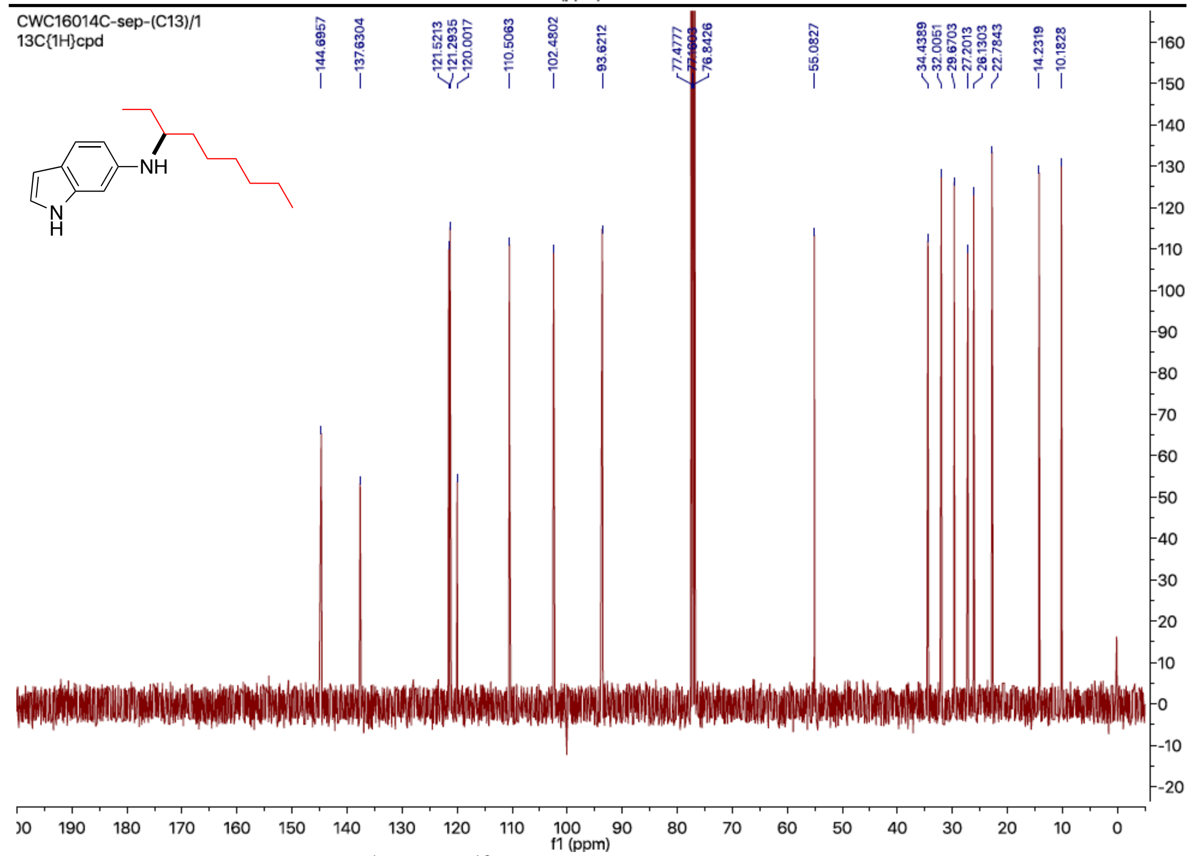

Supplementary Figure 44. ^{1}H and ^{13}C NMR spectra of *N*-(Nonan-3-yl)-1*H*-indol-5-amine (4i)

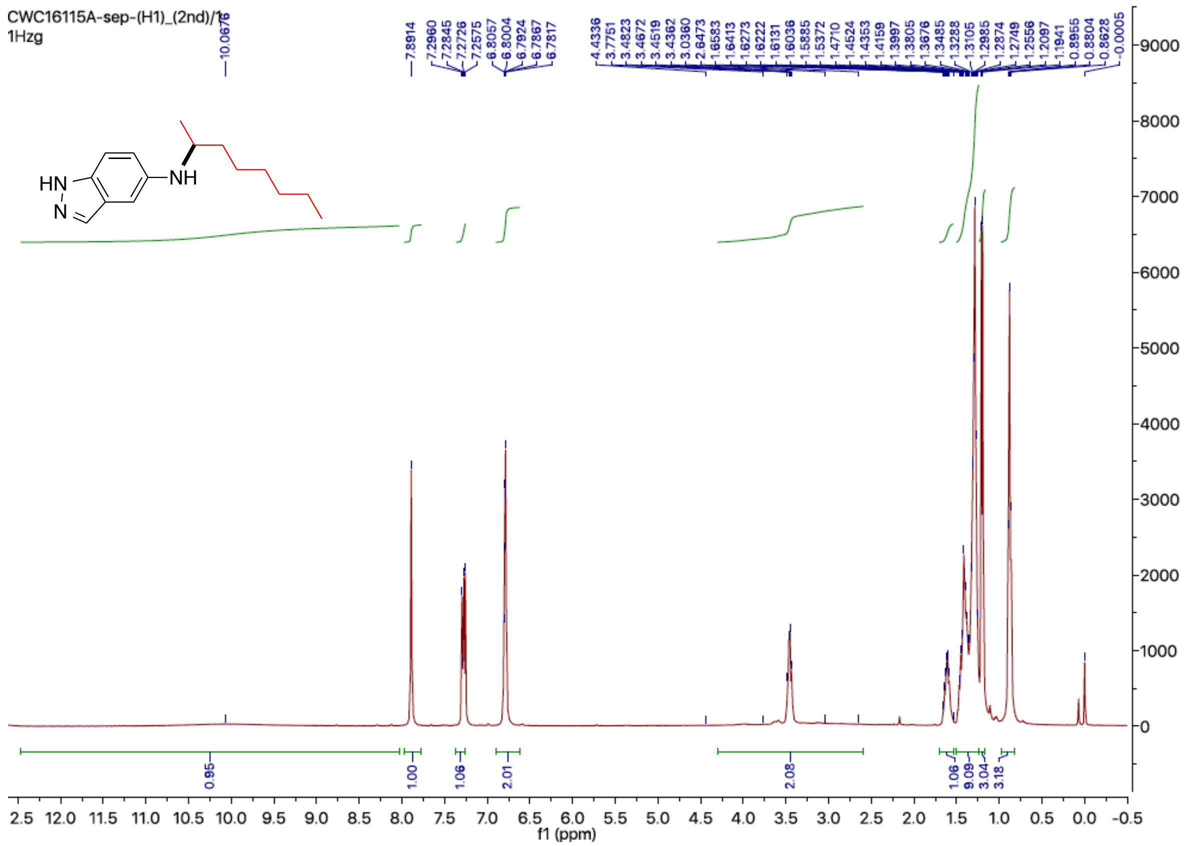

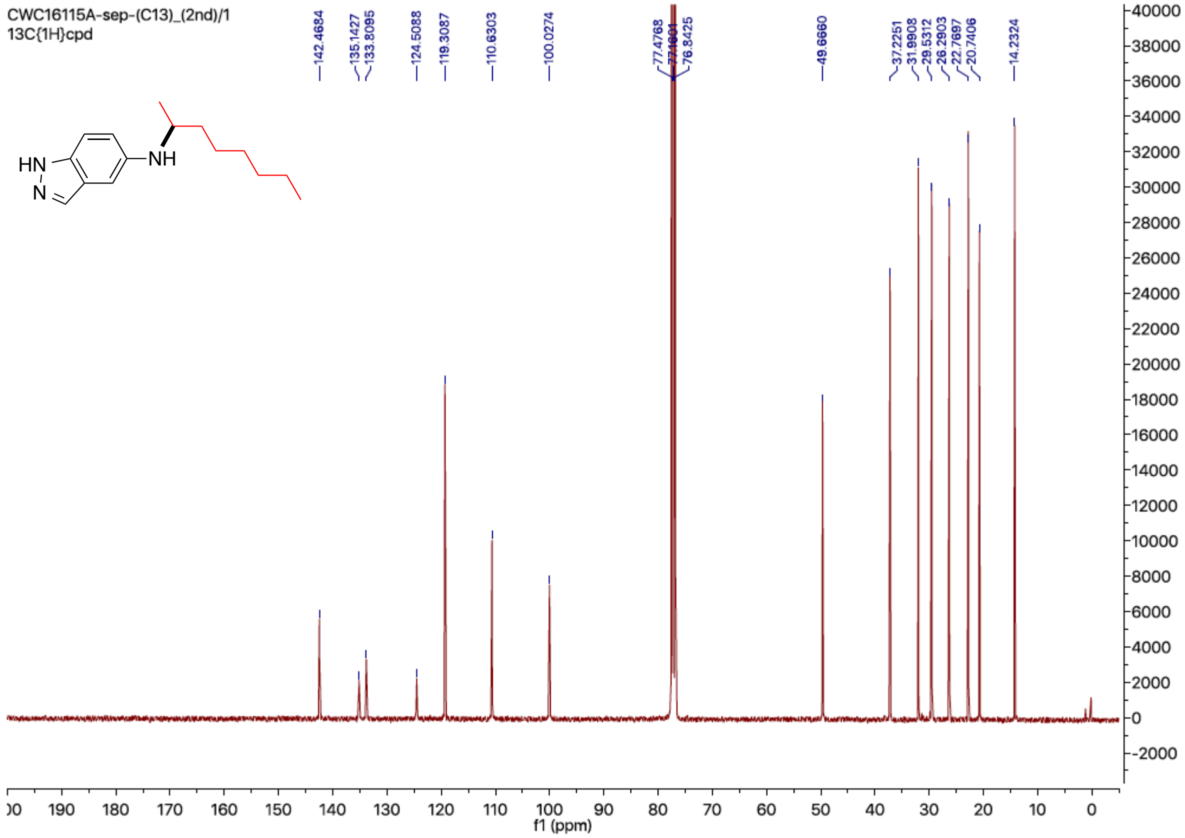

Supplementary Figure 45. **^{1}H and ^{13}C NMR spectra of *N*-(Octan-2-yl)-1*H*-indazol-5-amine (4j)**

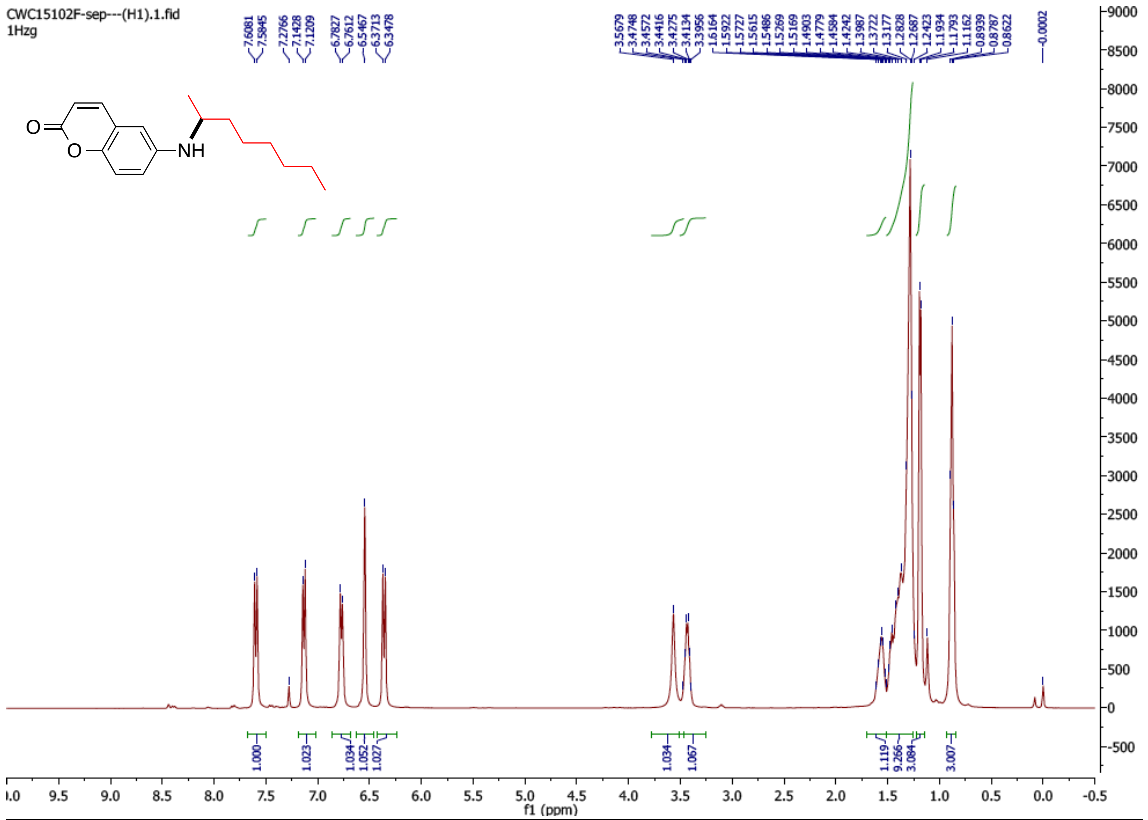

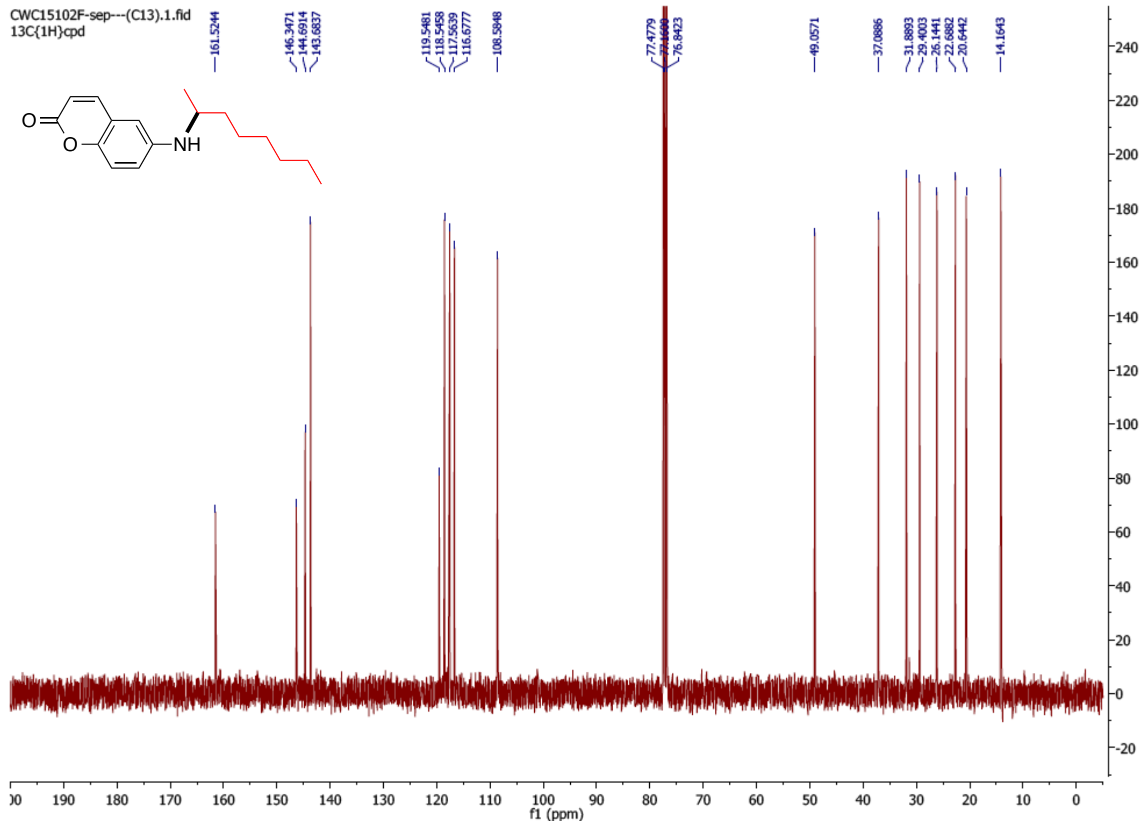

Supplementary Figure 46. ^{1}H and ^{13}C NMR spectra of 6-(Octan-2-ylamino)-2*H*-chromen-2-one (4k)

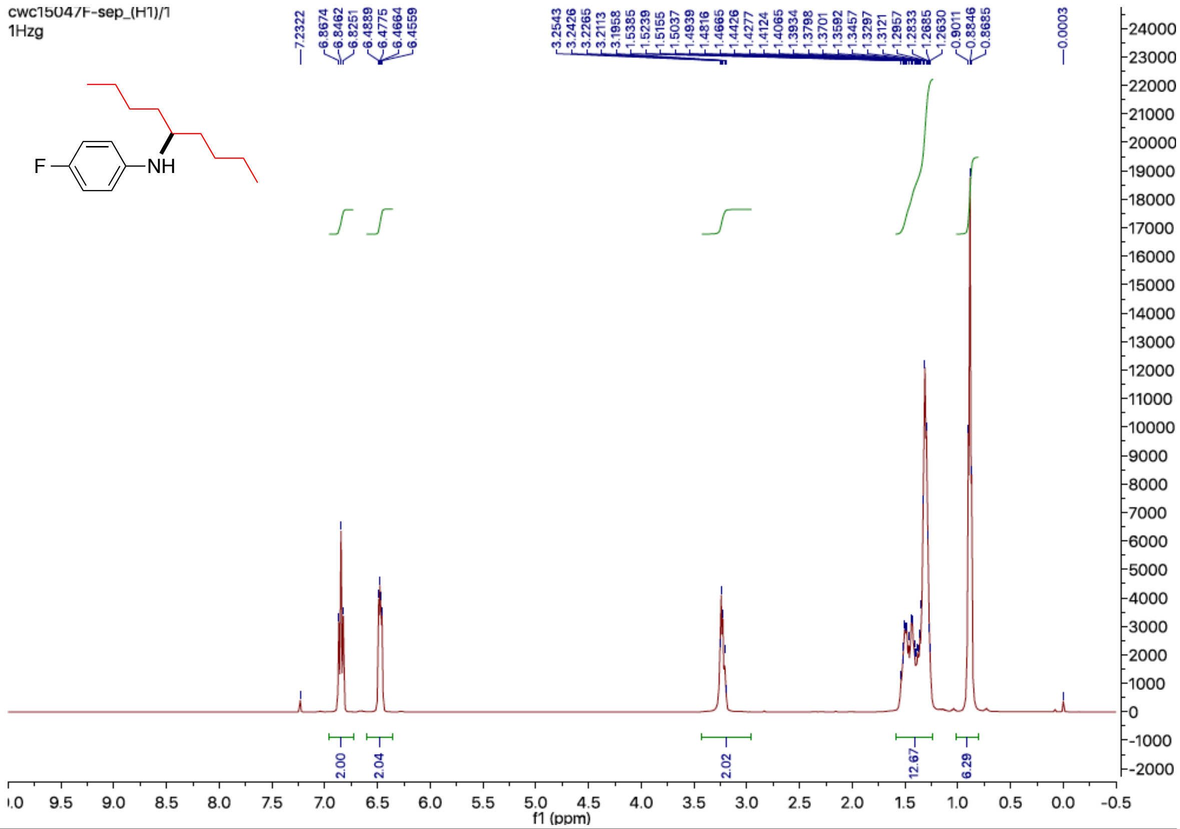

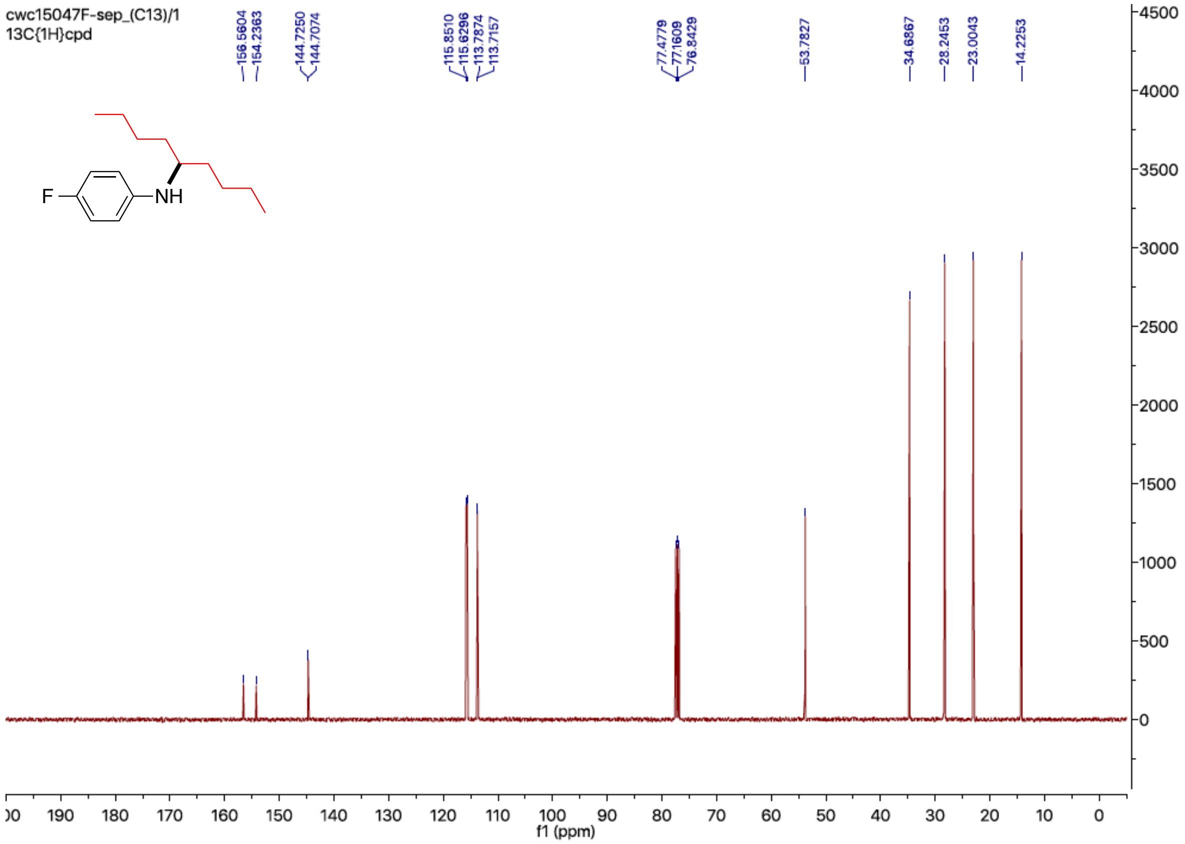

Supplementary Figure 47. ^{1}H and ^{13}C NMR spectra of 4-Fluoro-*N*-(nonan-5-yl)aniline (5a)

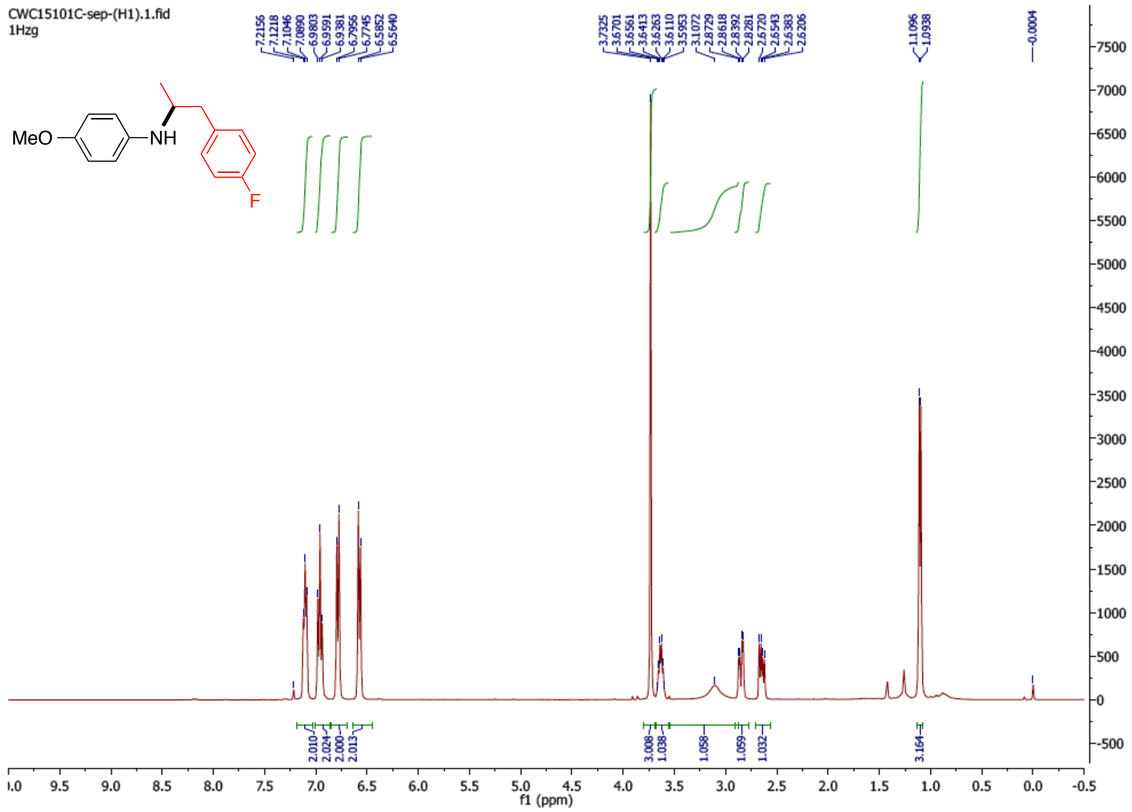

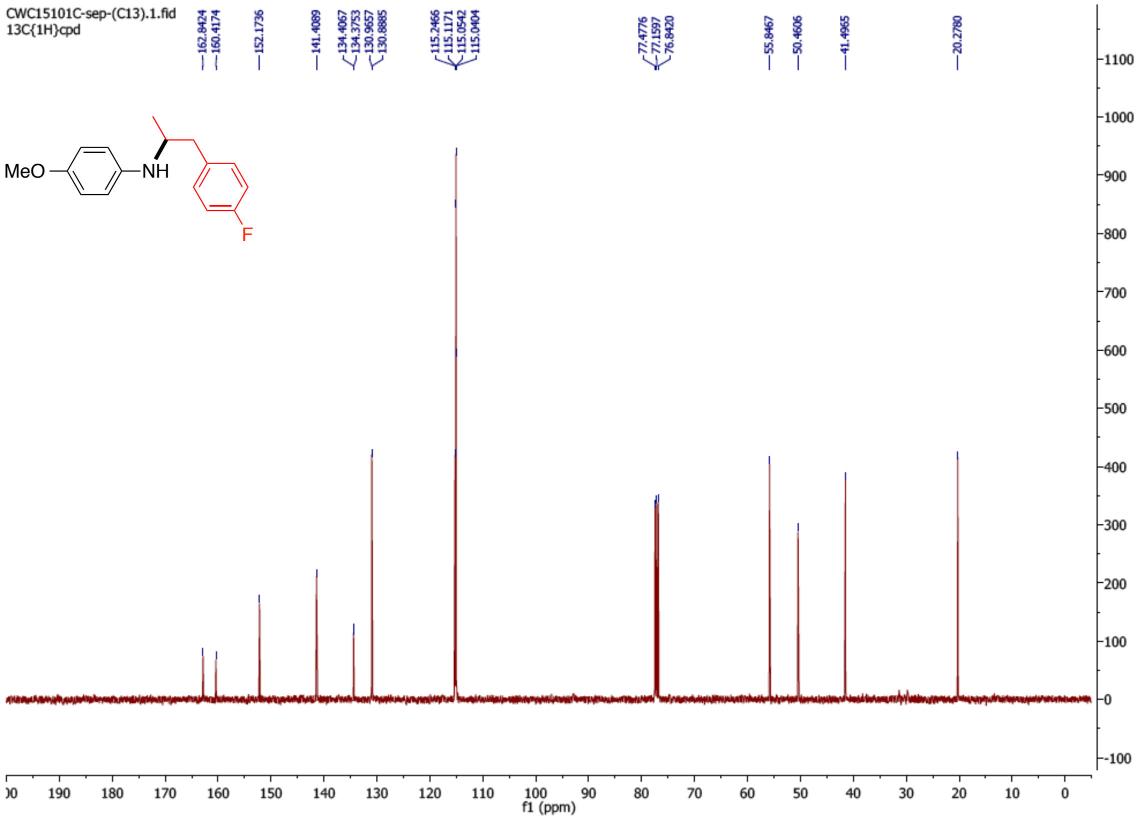

Supplementary Figure 48. ^{1}H and ^{13}C NMR spectra of *N*-(1-(4-Fluorophenyl)propan-2-yl)-4-methoxyaniline (5b)

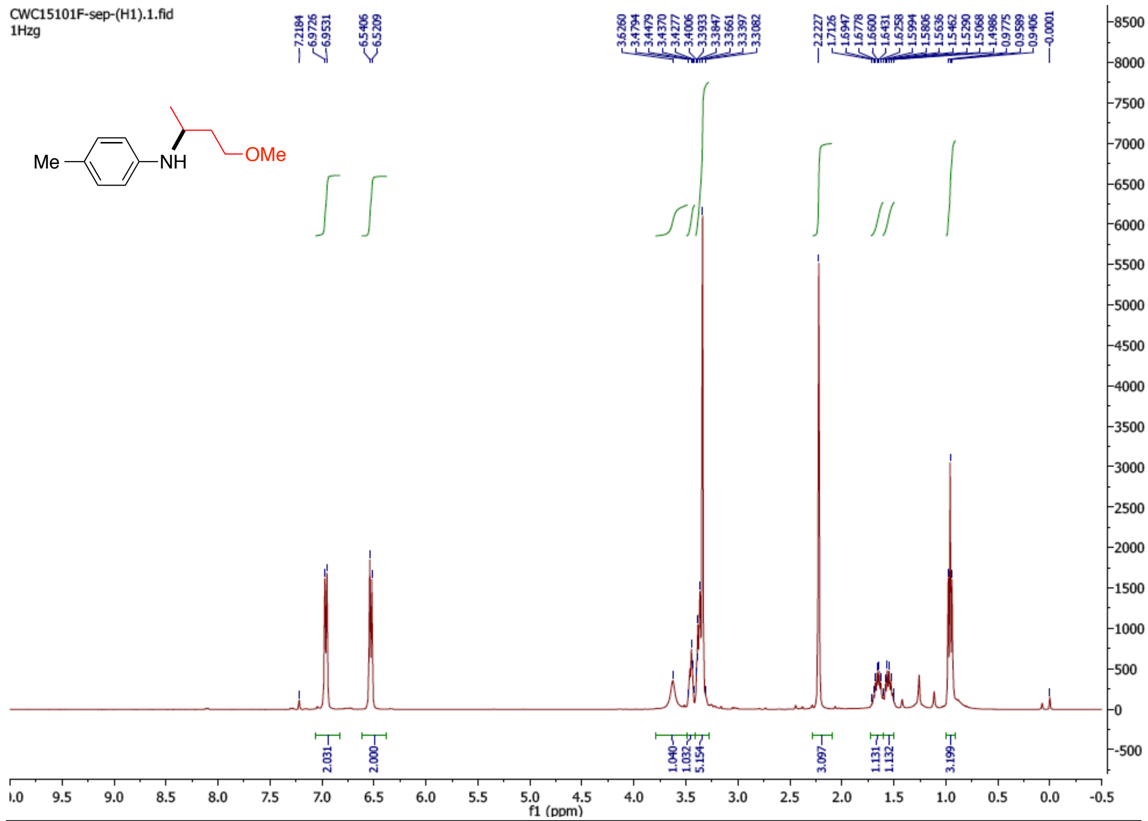

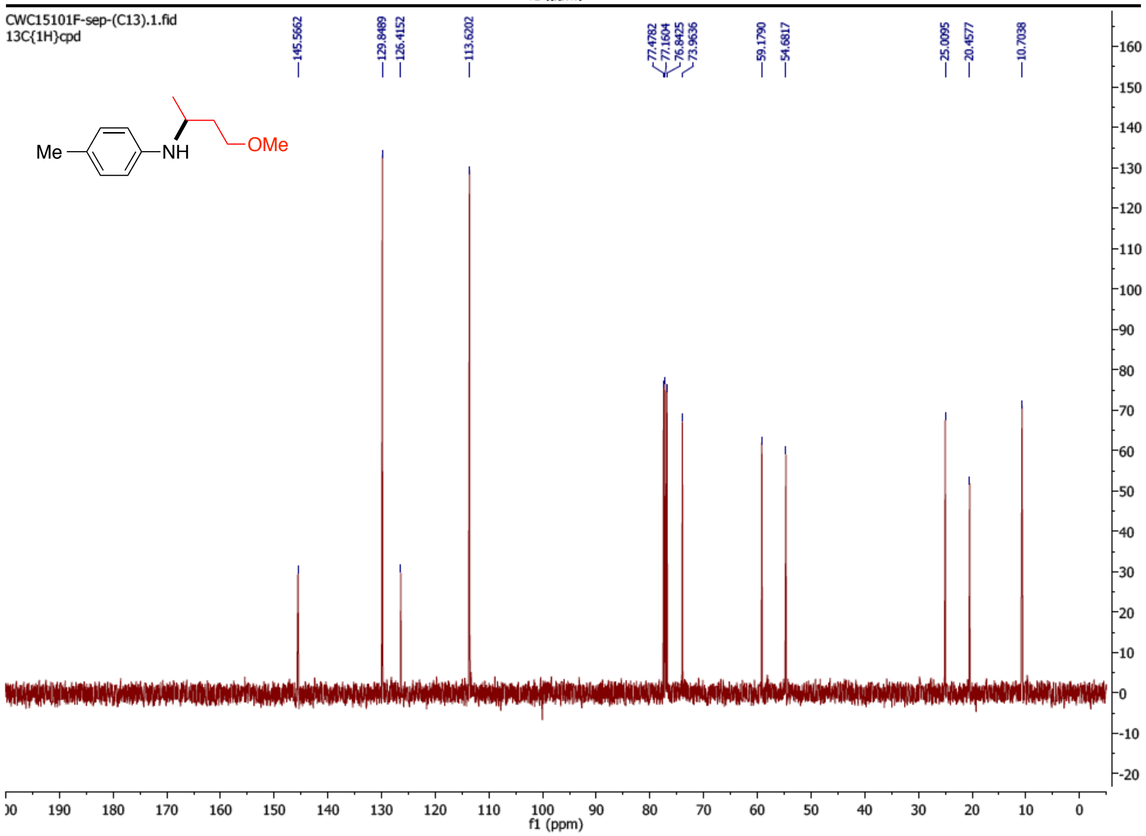

Supplementary Figure 49. ^{1}H and ^{13}C NMR spectra of *N*-(4-Methoxybutan-2-yl)-4-methylaniline (5c)

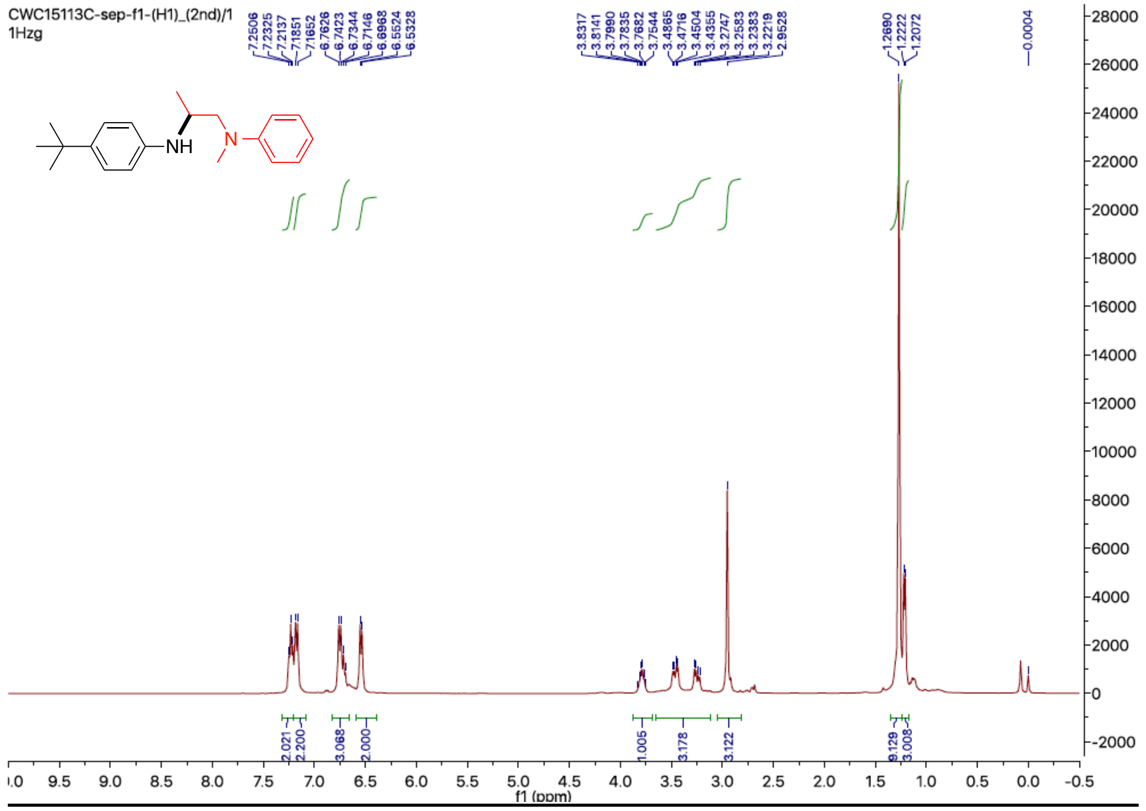

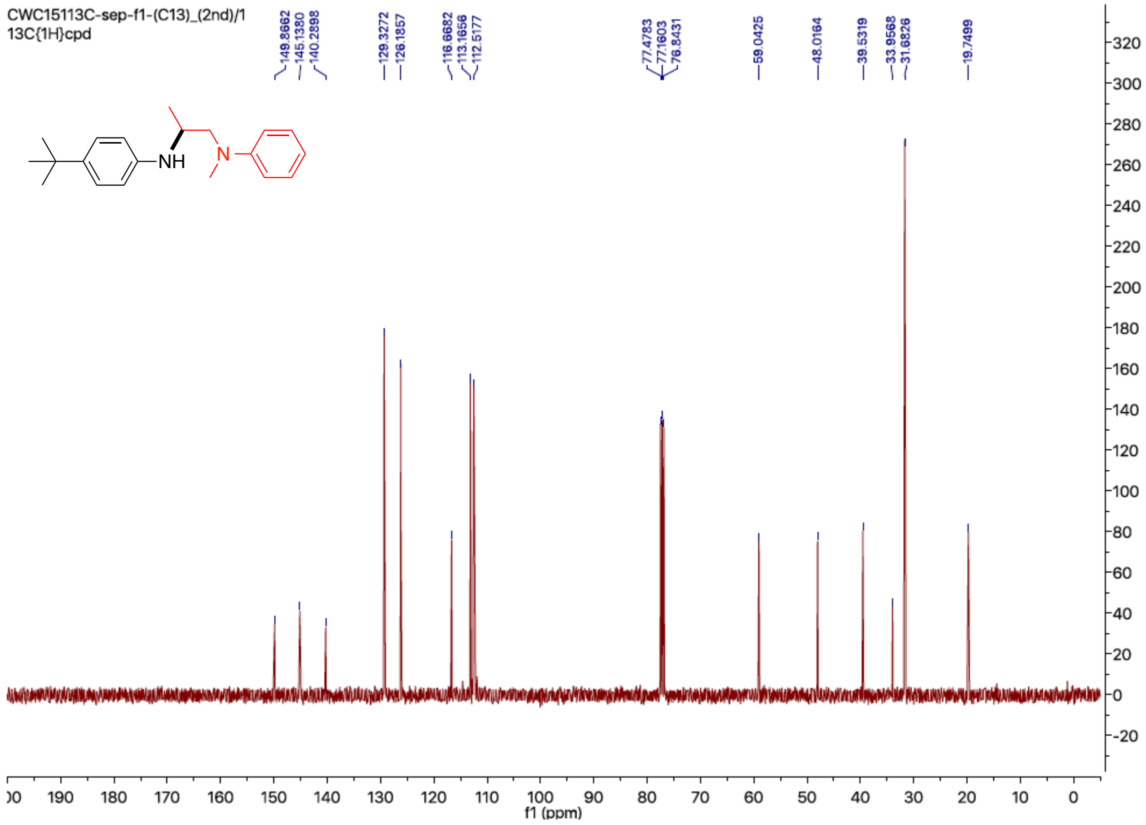

Supplementary Figure 50. ^{1}**H and** ^{13}**C NMR spectra of** ***N***^{2}**-(4-(*****tert*****-Butyl)phenyl)-*****N***^{1}**-methyl-*****N***^{1}**-phenylpropane-1,2-diamine (5d)**

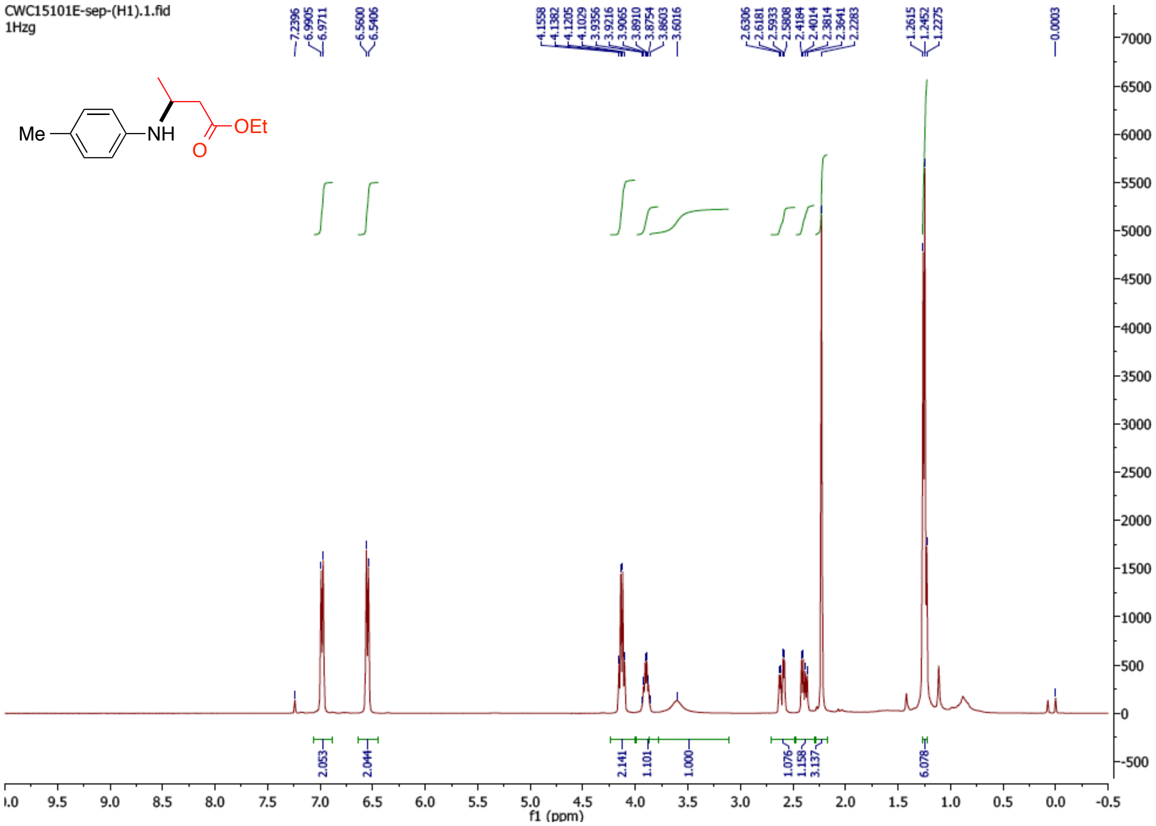

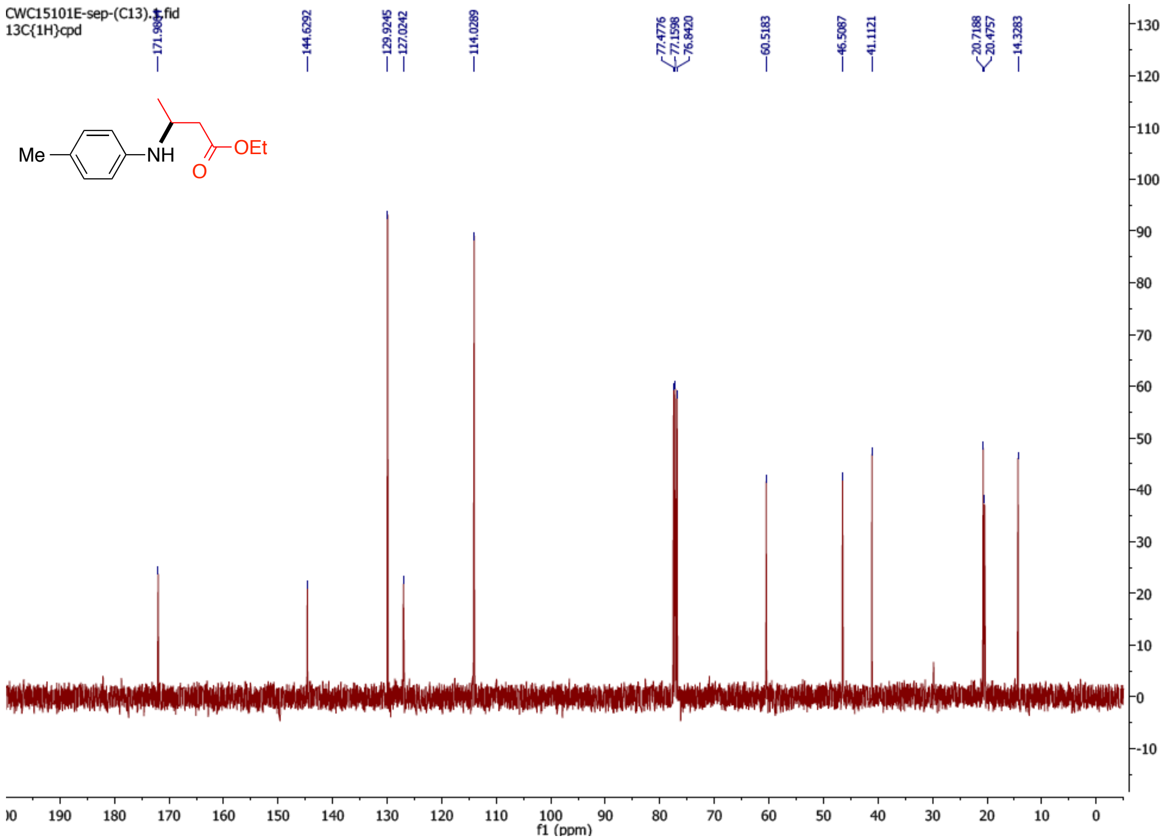

Supplementary Figure 51. ^{1}H and ^{13}C NMR spectra of Ethyl 3-(*p*-Tolylamino)butanoate (5e)

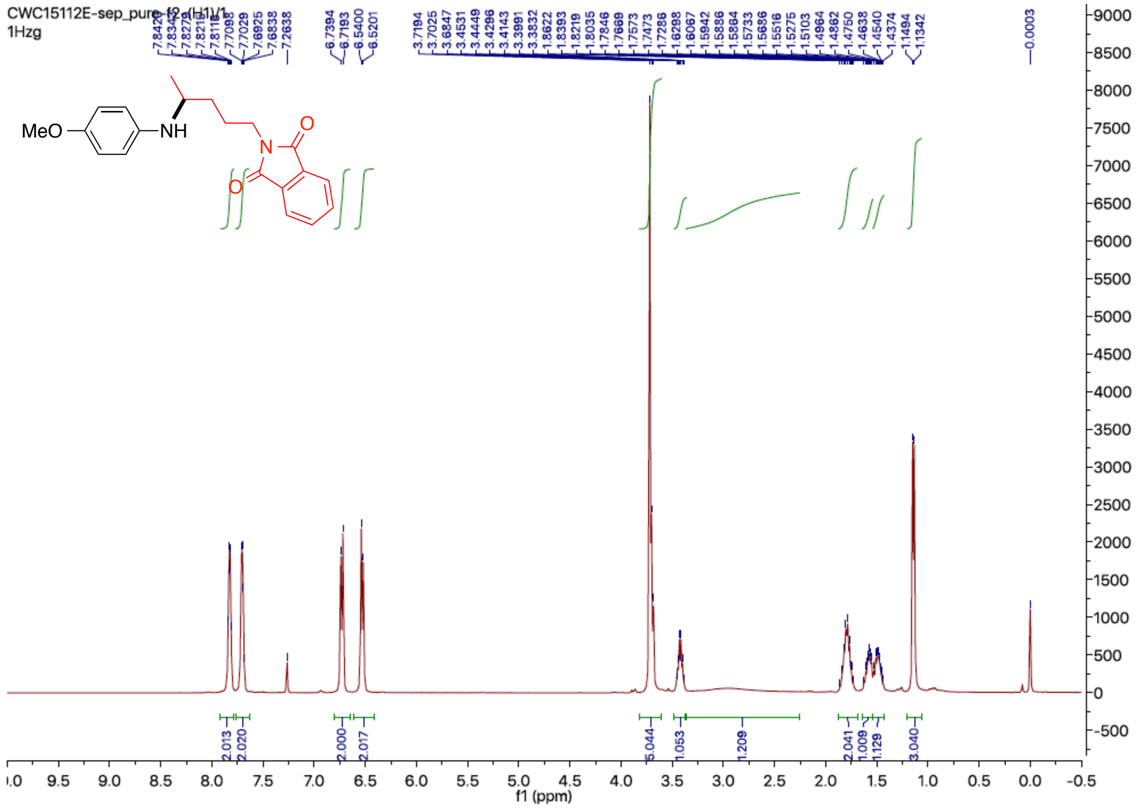

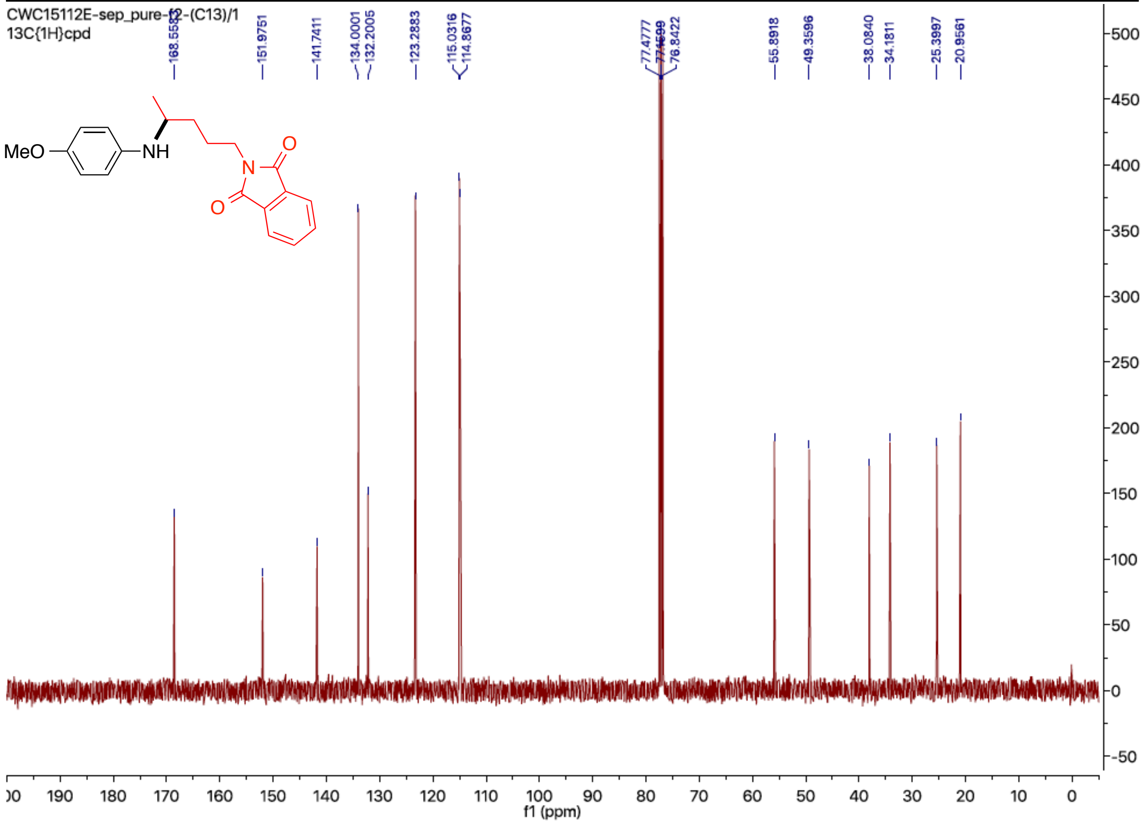

Supplementary Figure 52. ^{1}H **and** ^{13}C **NMR spectra of 2-(4-((4-Methoxyphenyl)amino)pentyl)isoindoline-1,3-dione (5f)**

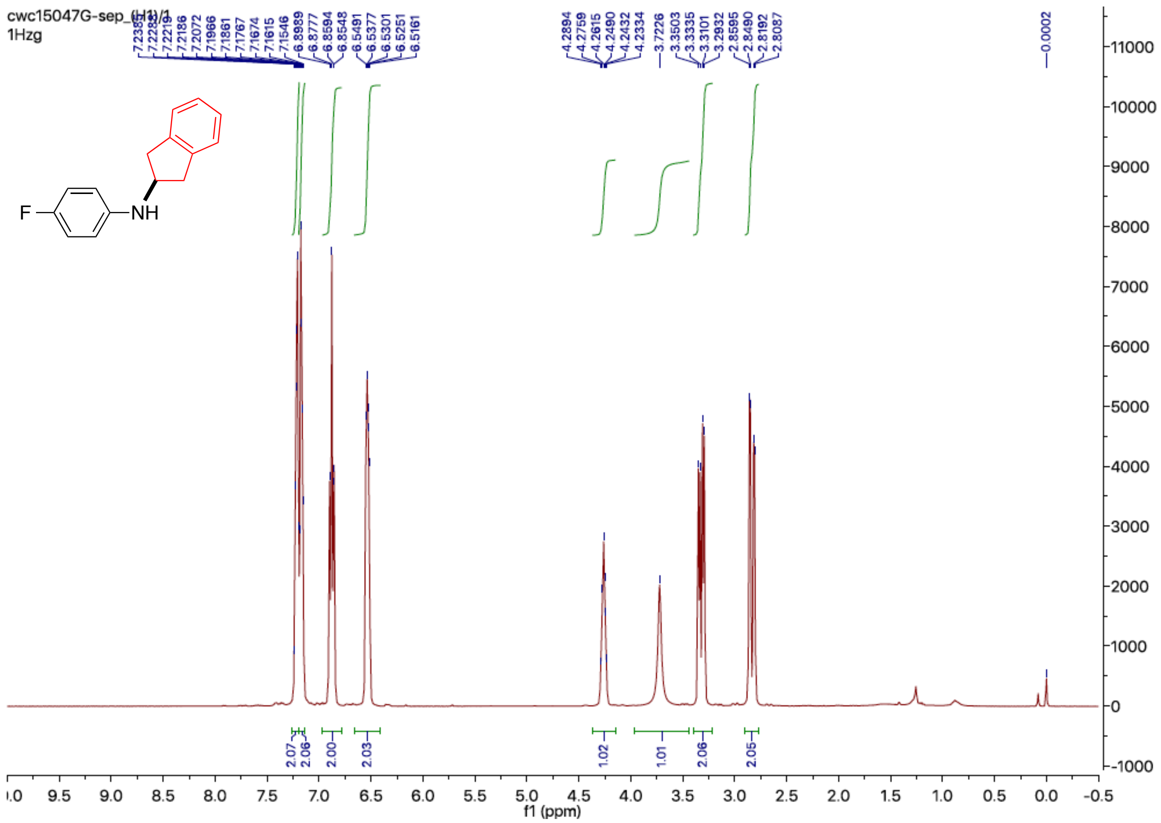

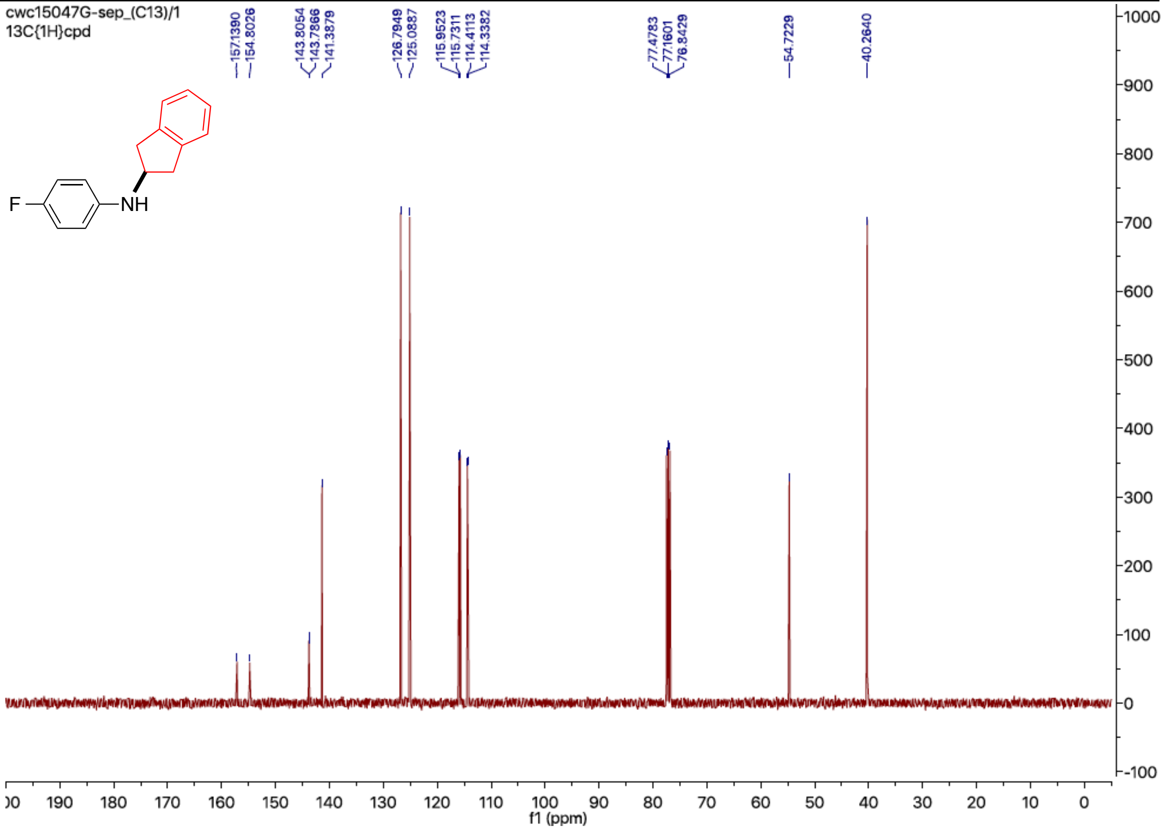

Supplementary Figure 53. ^{1}H and ^{13}C NMR spectra of *N*-(4-Fluorophenyl)-2,3-dihydro-1*H*-inden-2-amine (5g)

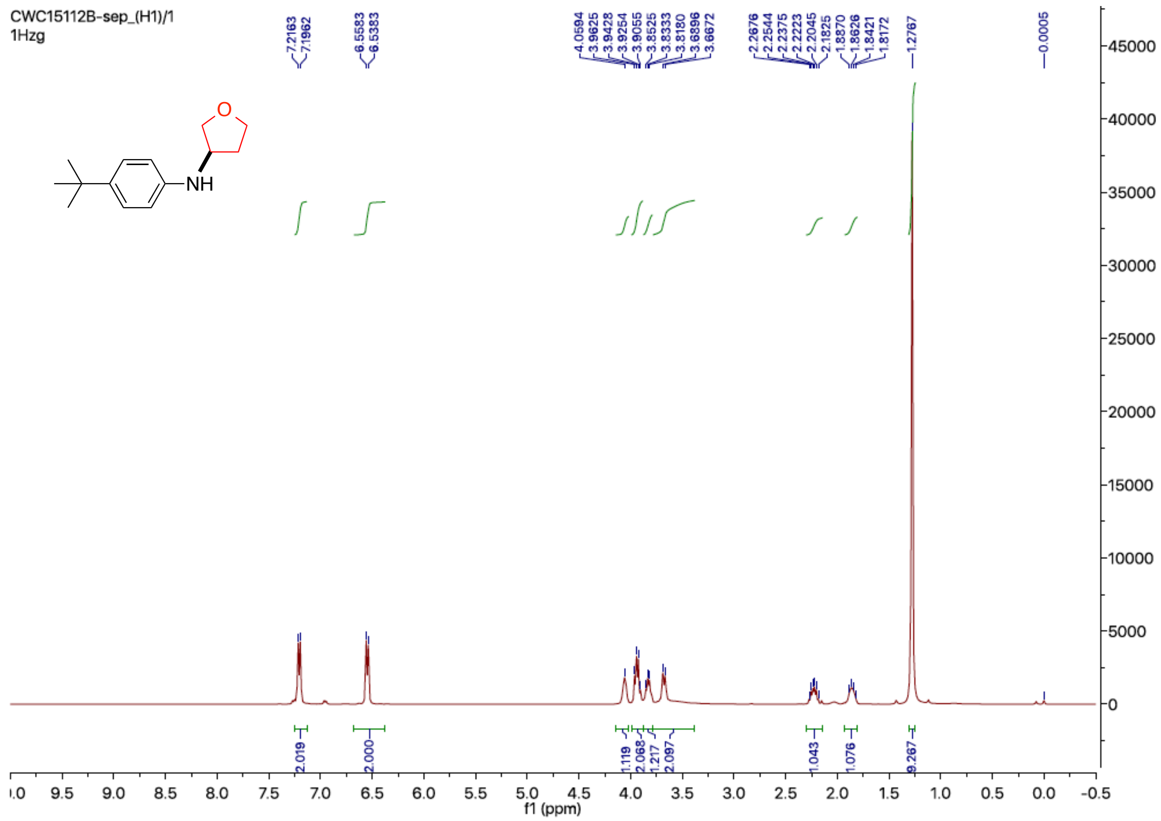

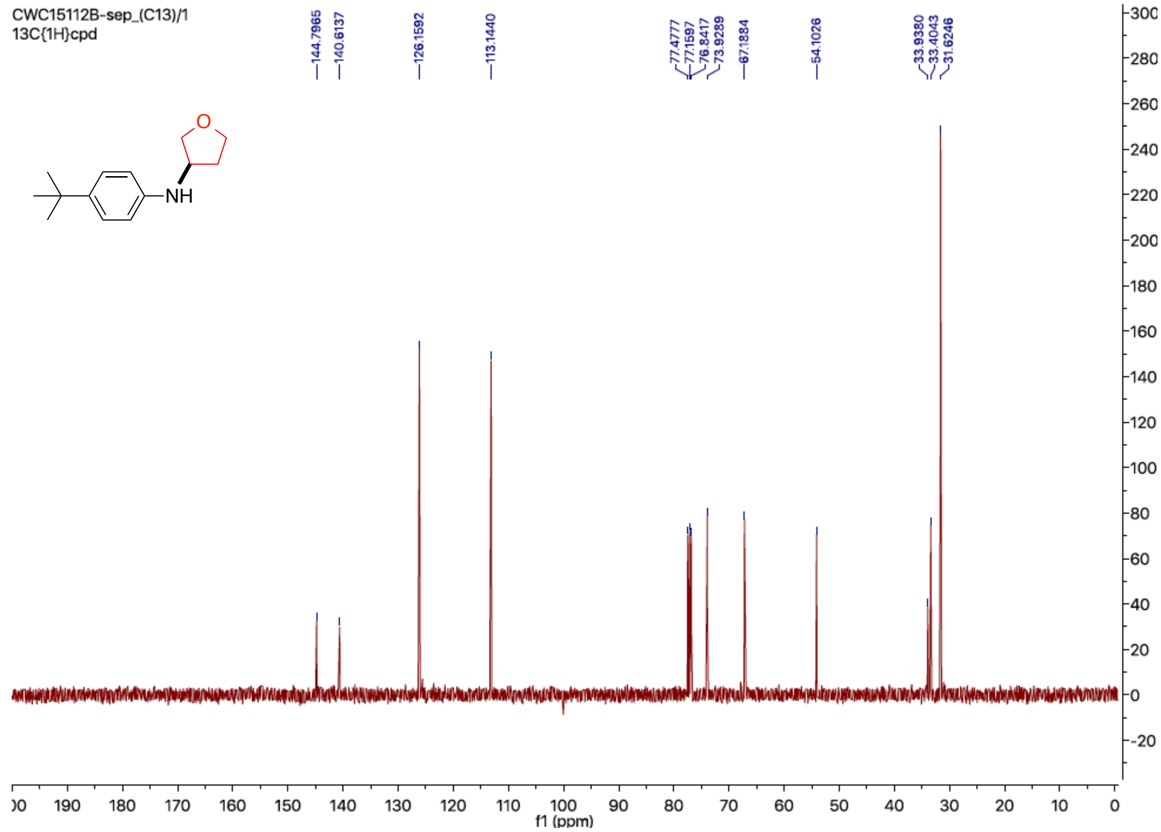

Supplementary Figure 54. **^{1}H and ^{13}C NMR spectra of *N*-(4-(*tert*-Butyl)phenyl)tetrahydrofuran-3-amine (5h)**

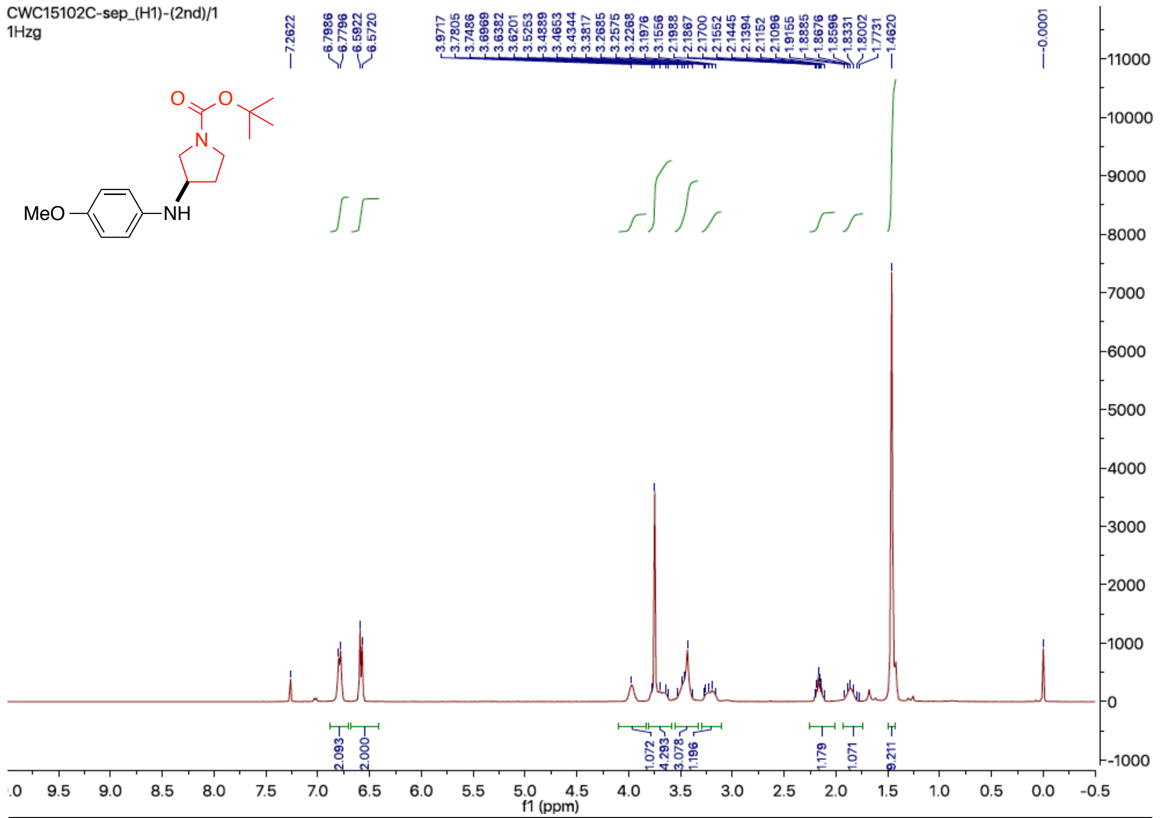

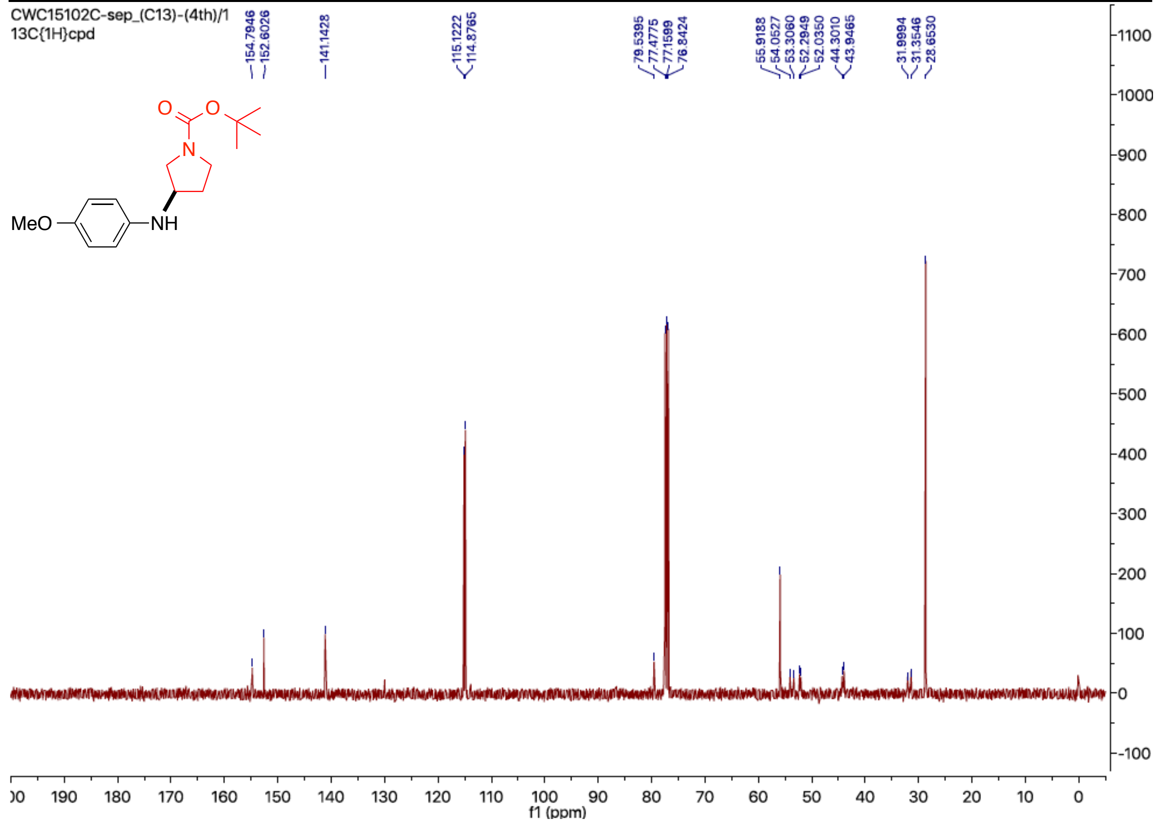

Supplementary Figure 55. ^{1}H and ^{13}C NMR spectra of *tert*-Butyl 3-((4-methoxyphenyl)amino)pyrrolidine-1-carboxylate (5i)

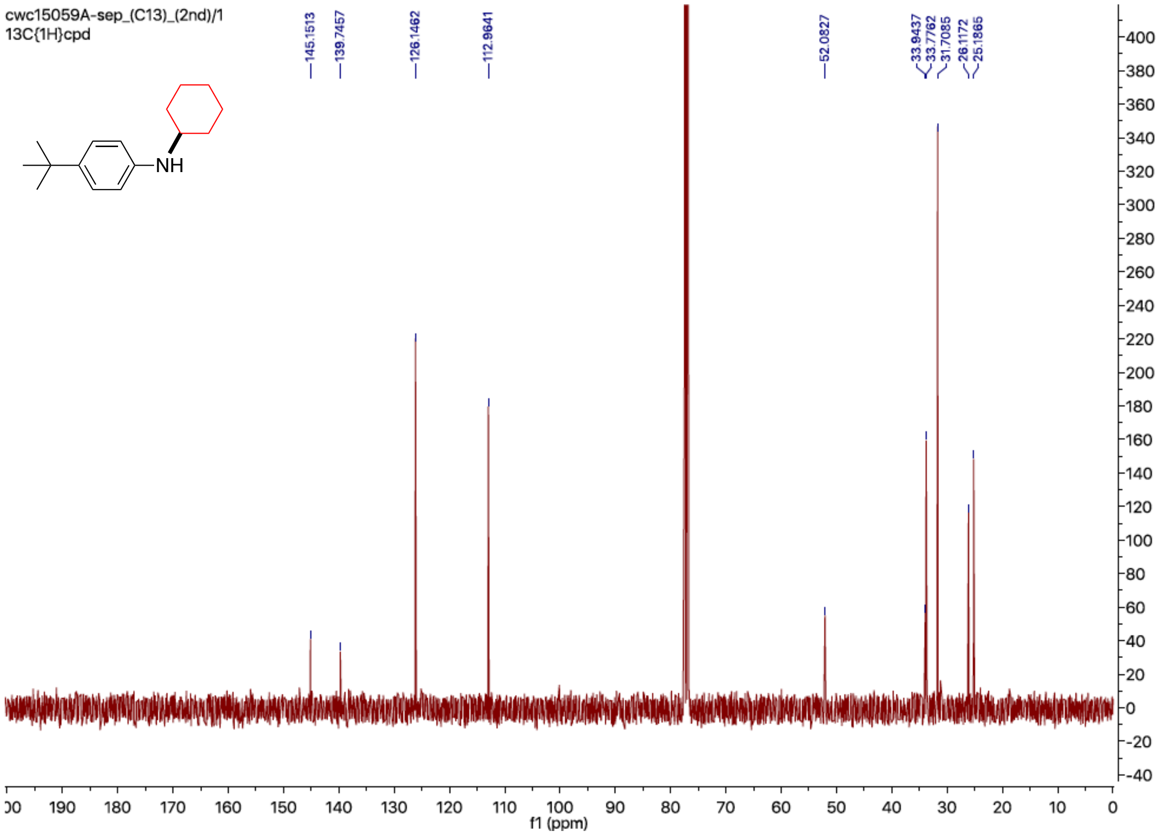

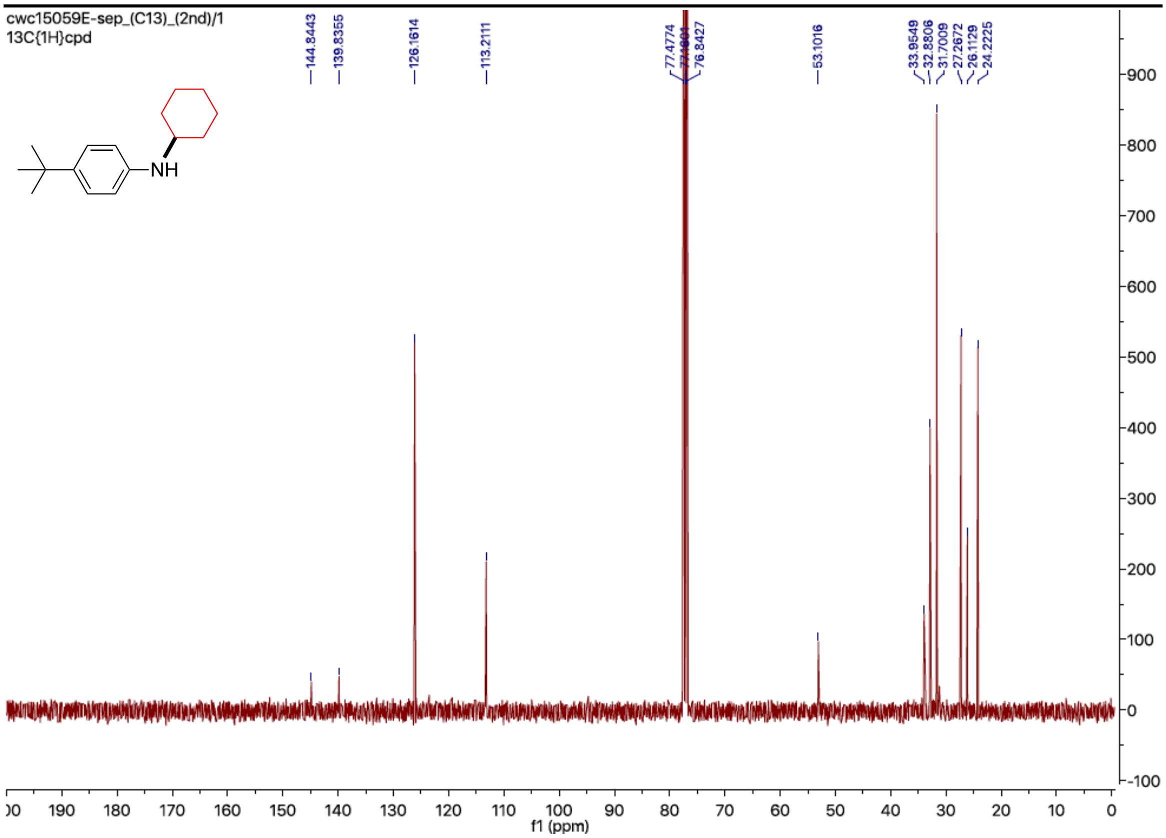

Supplementary Figure 56. ^{1}H and ^{13}C NMR spectra of 4-(*tert*-Butyl)-*N*-cyclohexylaniline (5j)

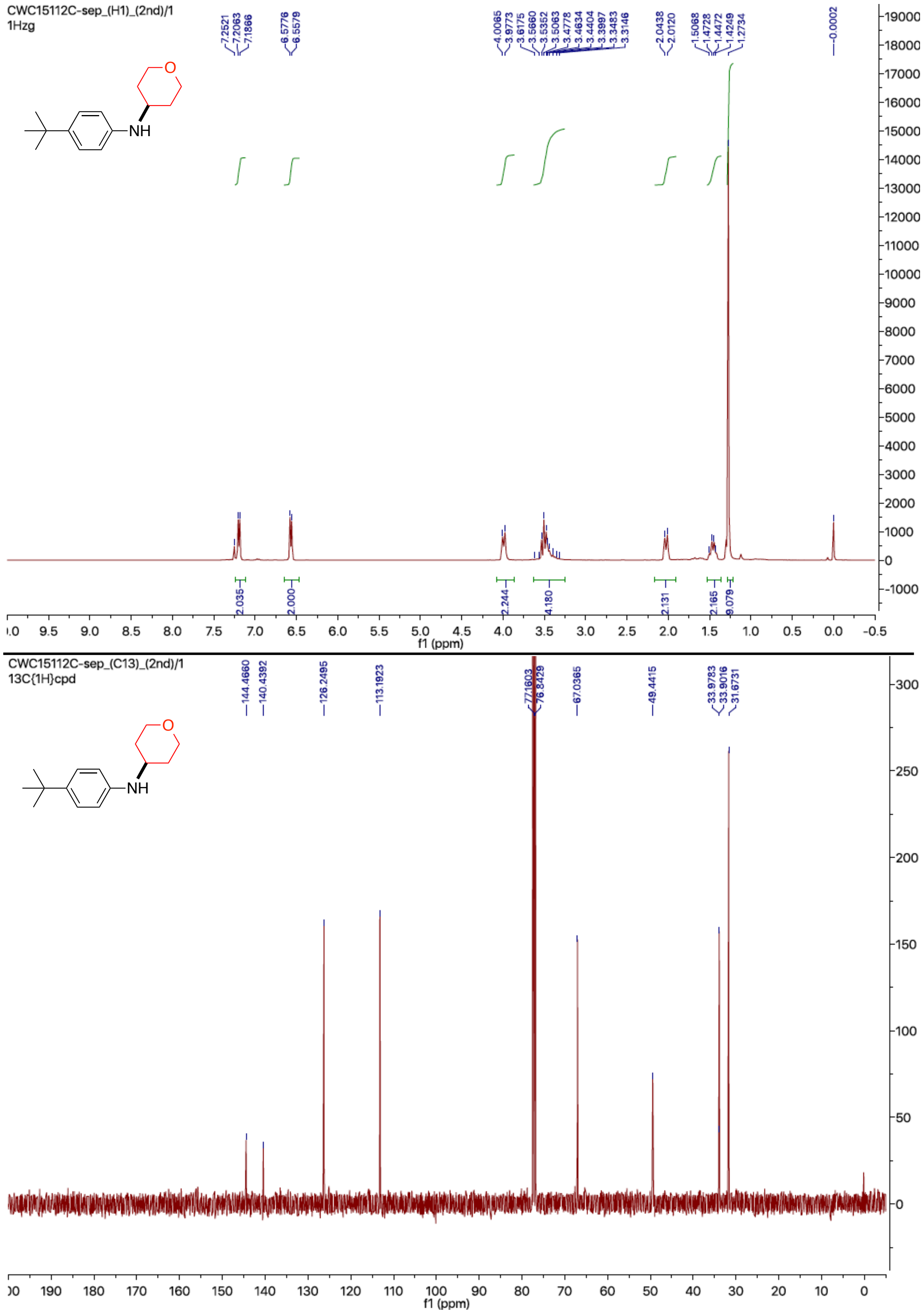

Supplementary Figure 57. ^{1}H **and** ^{13}C **NMR spectra of** ***N*****-(4-(*****tert*****-Butyl)phenyl)tetrahydro-2*****H*****-pyran-4-amine (5k)**

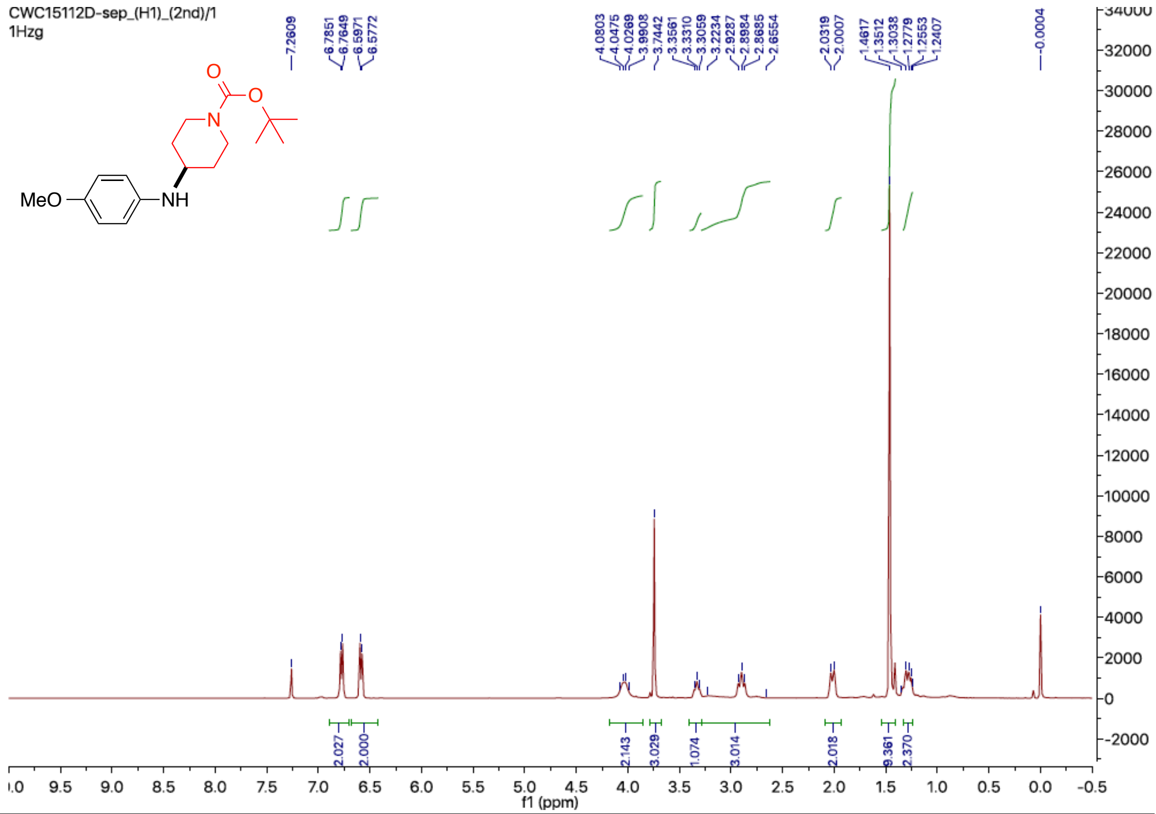

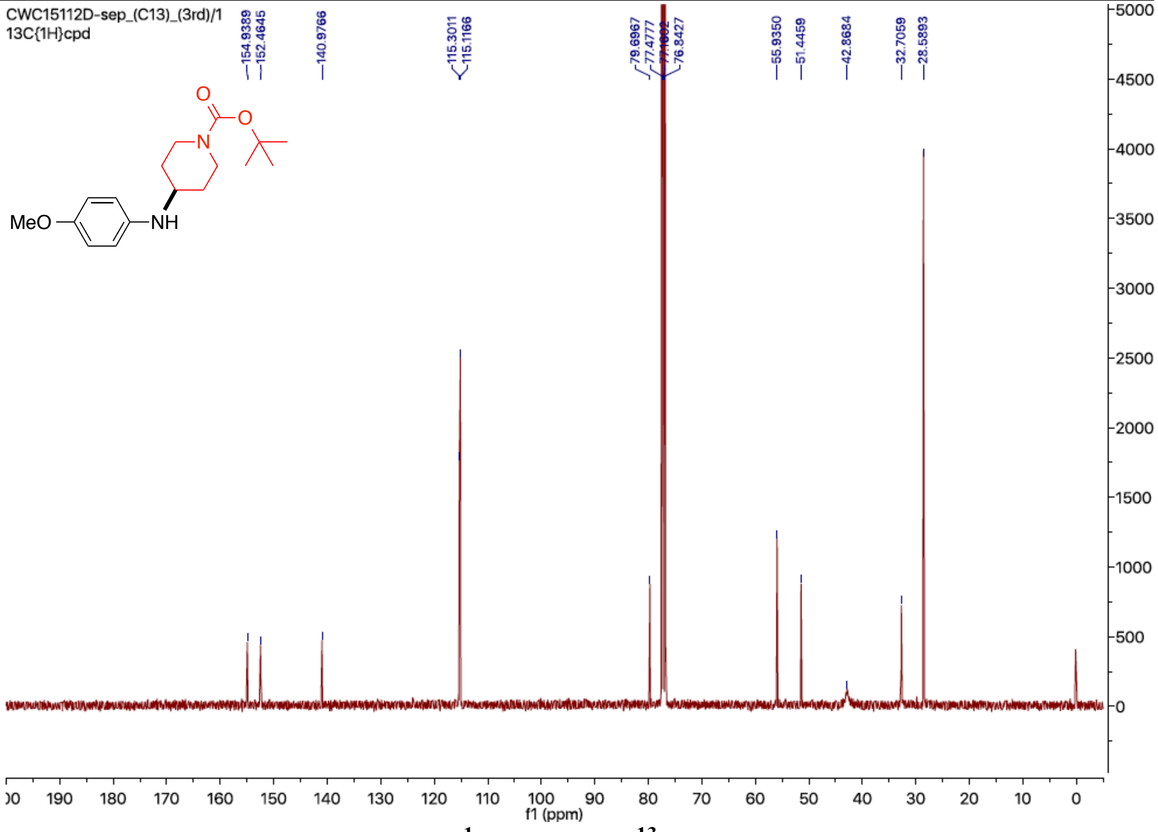

Supplementary Figure 58. **^{1}H and ^{13}C NMR spectra of *tert*-Butyl 4-((4-methoxyphenyl)amino)piperidine-1-carboxylate (5l)**

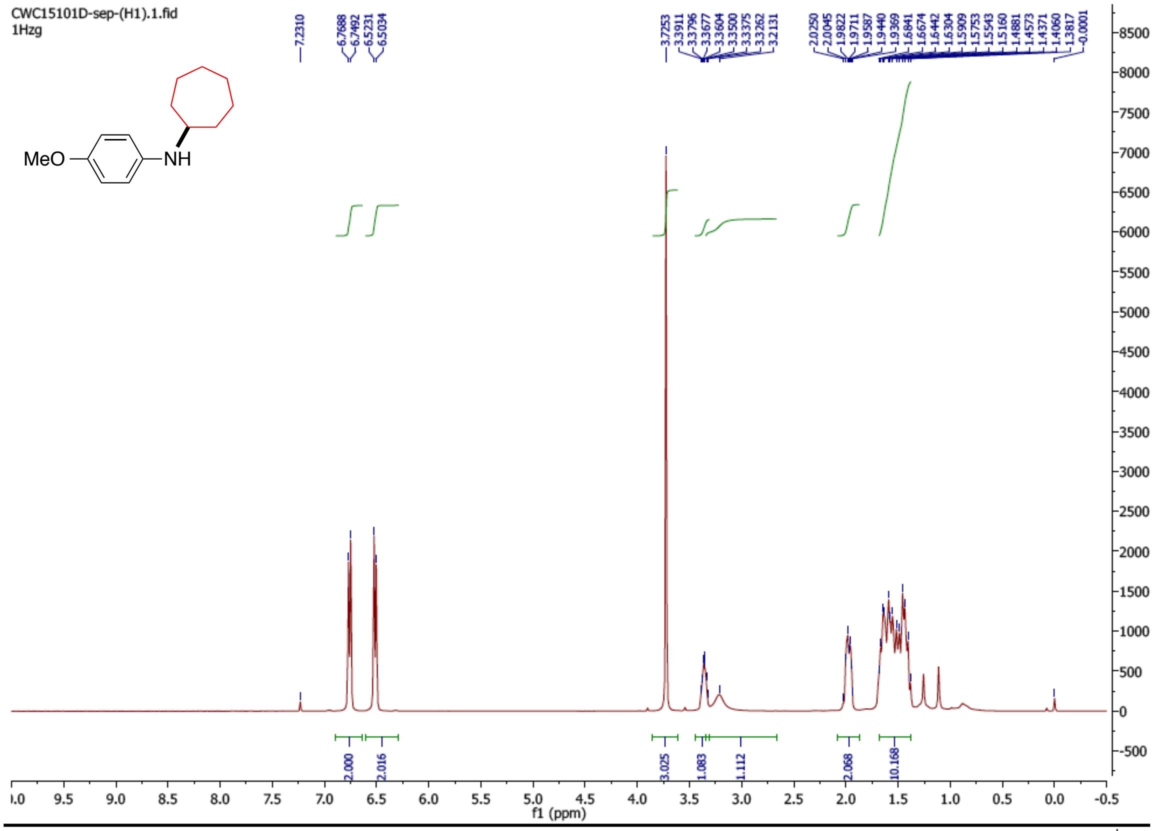

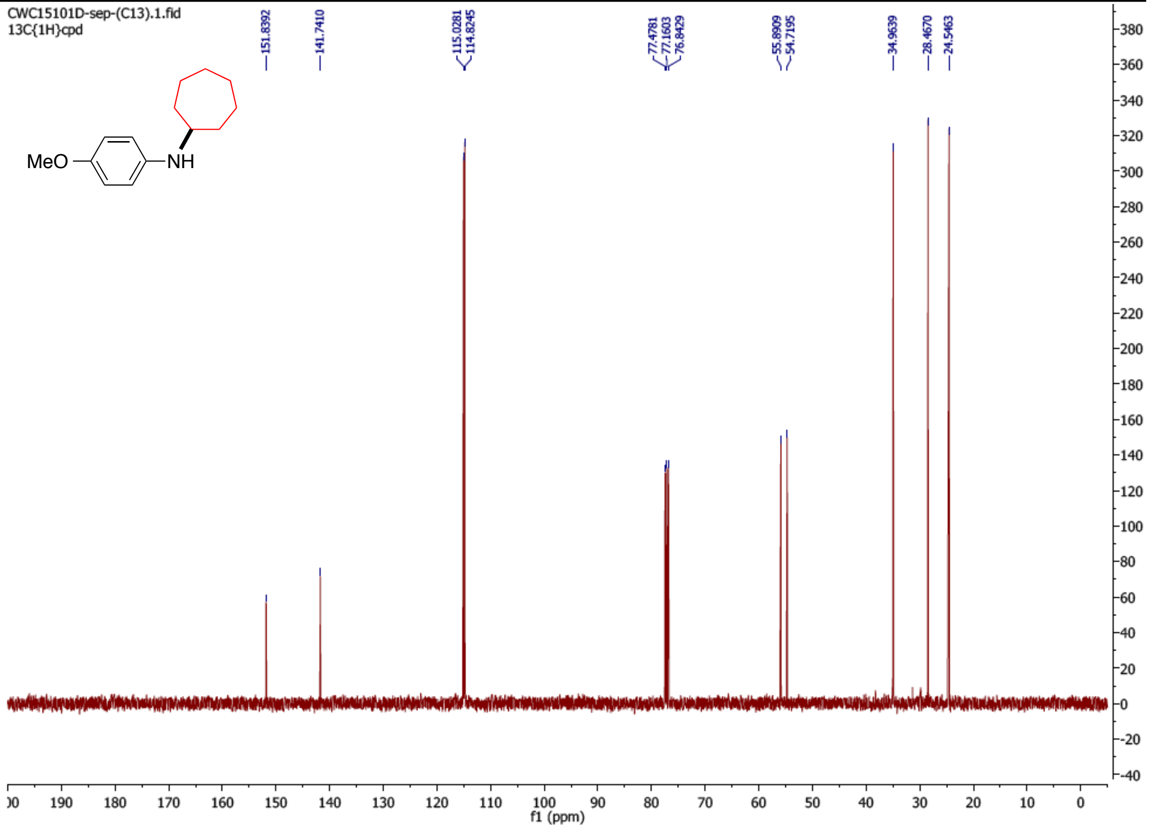

Supplementary Figure 59. ^{1}H and ^{13}C NMR spectra of *N*-(4-Methoxyphenyl)cycloheptanamine (5m)

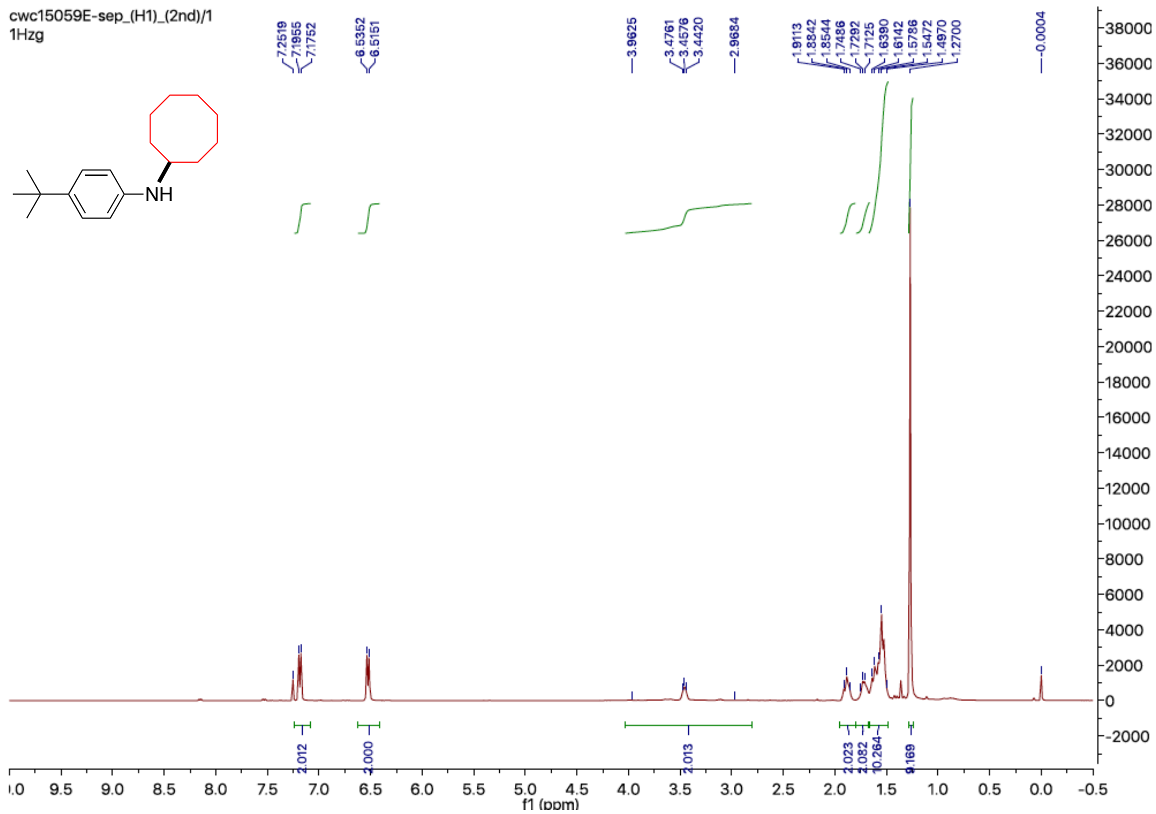

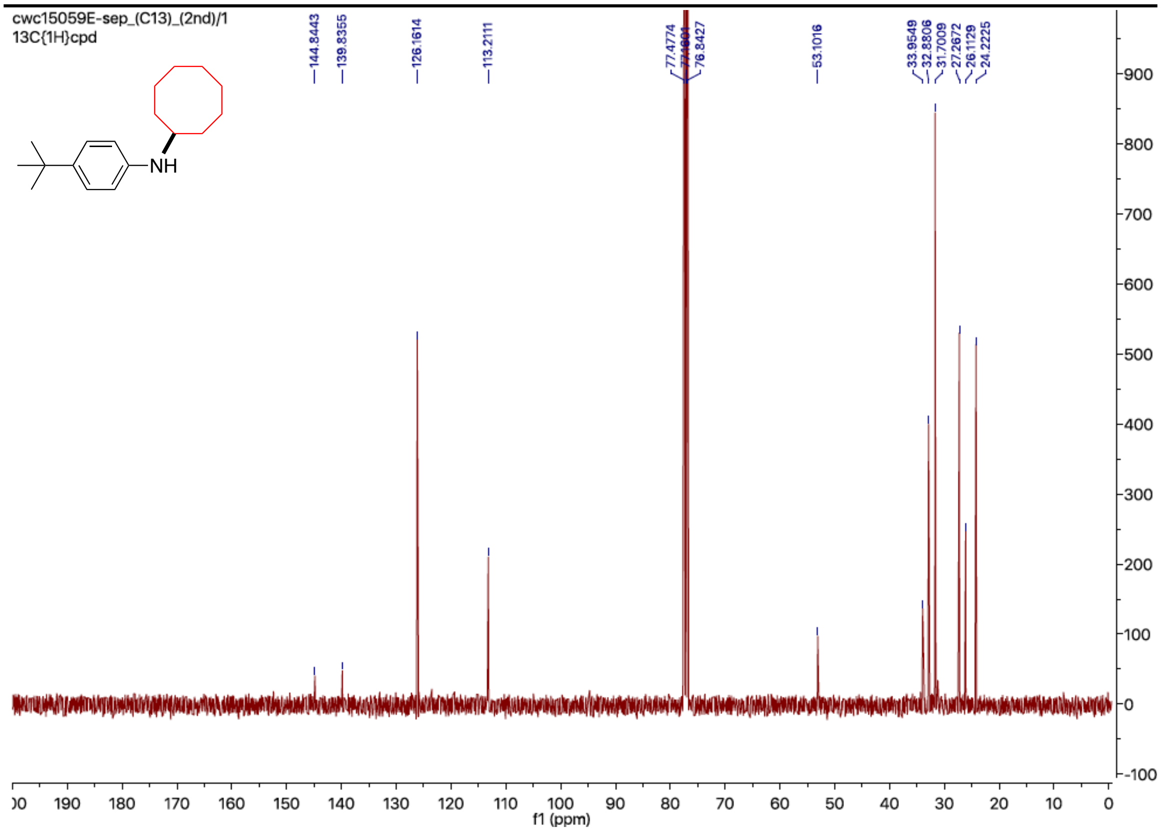

Supplementary Figure 60. ^{1}H and ^{13}C NMR spectra of *N*-(4-(*tert*-Butyl)phenyl)cyclooctanamine (5n)

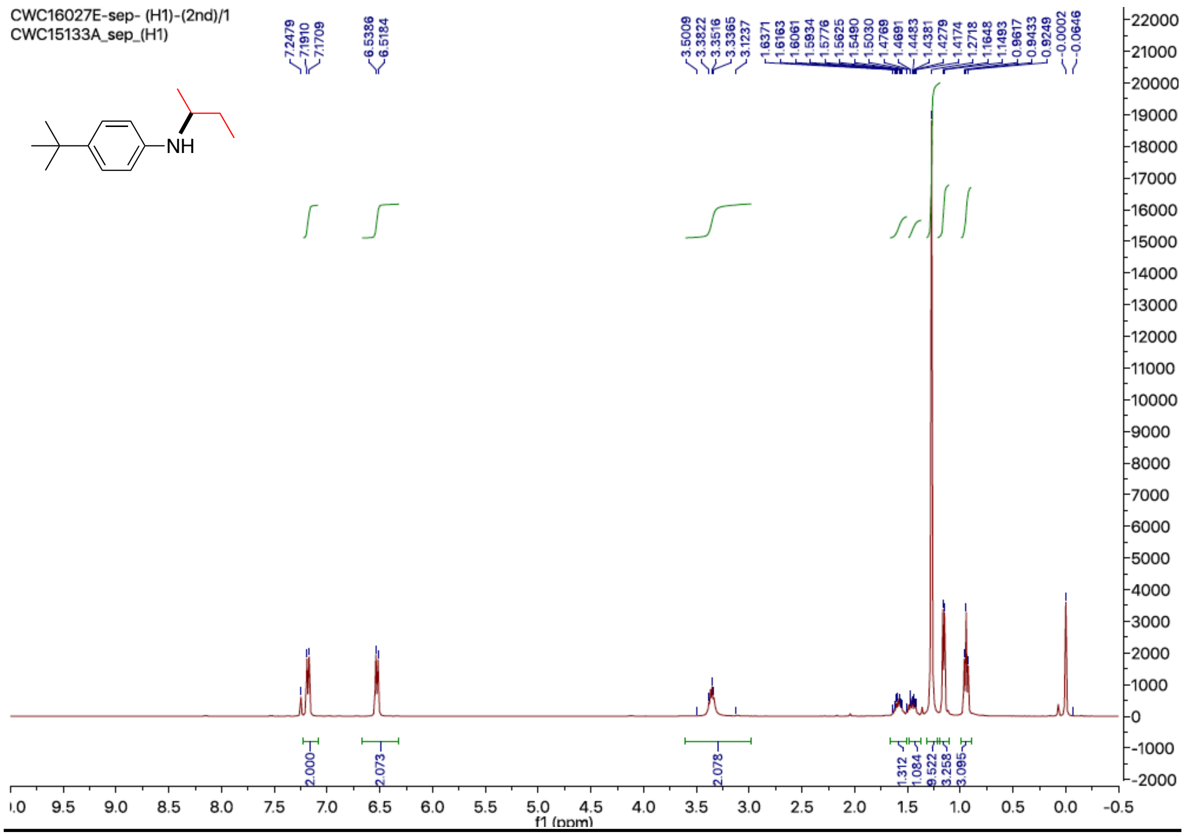

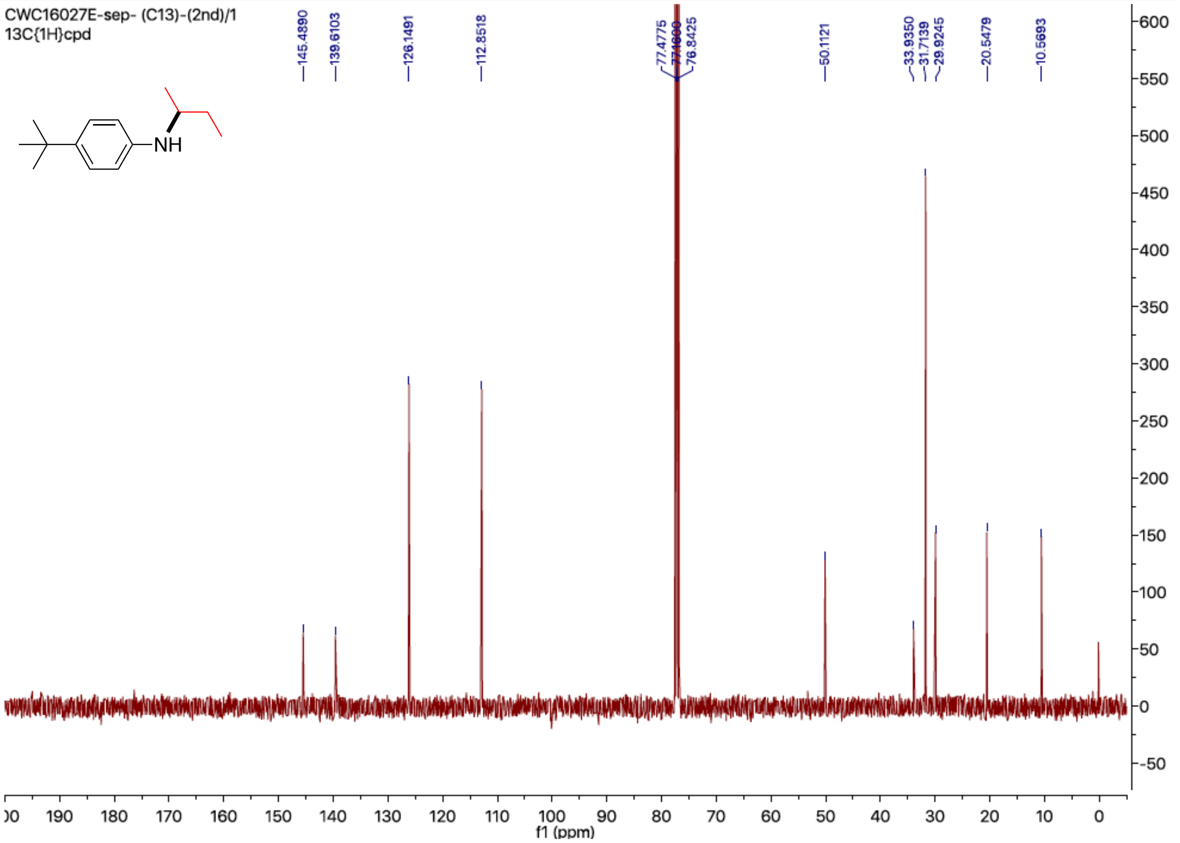

Supplementary Figure 61. ^{1}H and ^{13}C NMR spectra of *N*-(*sec*-Butyl)-4-(*tert*-butyl)aniline (5o)

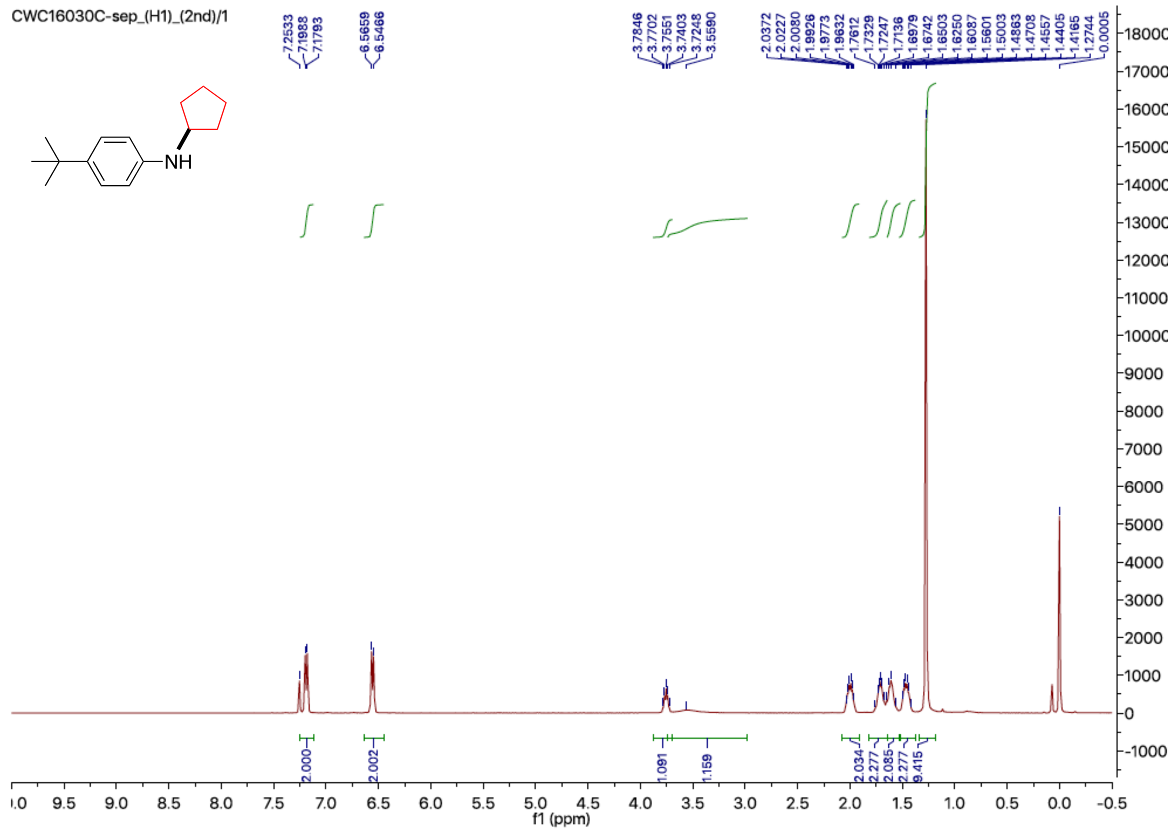

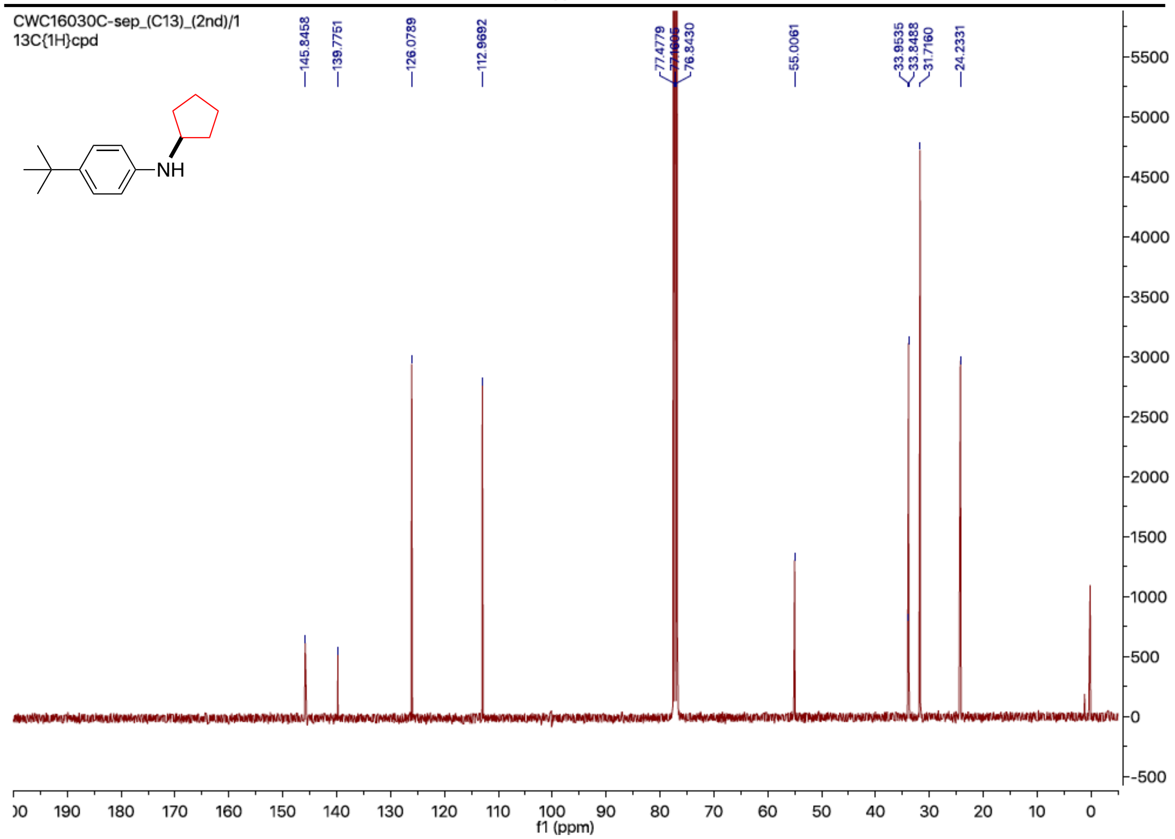

Supplementary Figure 62. ^{1}**H and** ^{13}**C NMR spectra of 4-(*tert*-Butyl)-*N*-cyclopentylaniline (5p)**

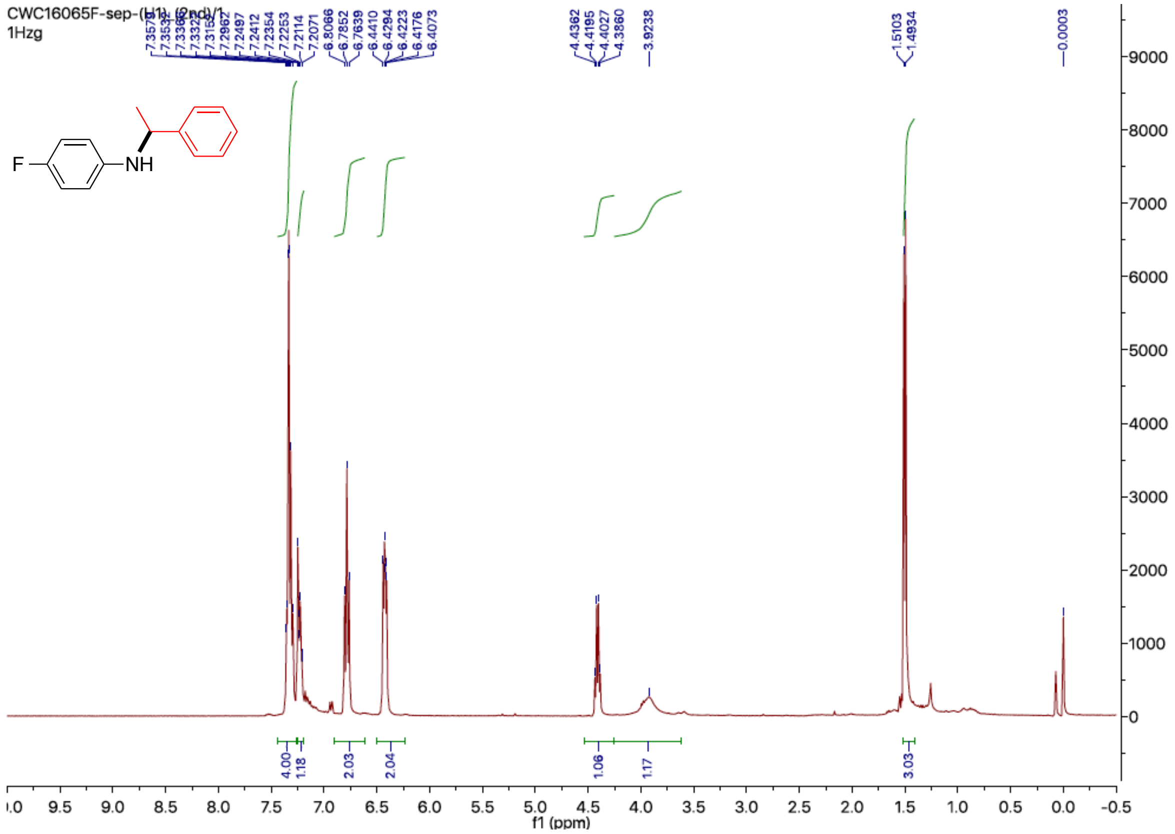

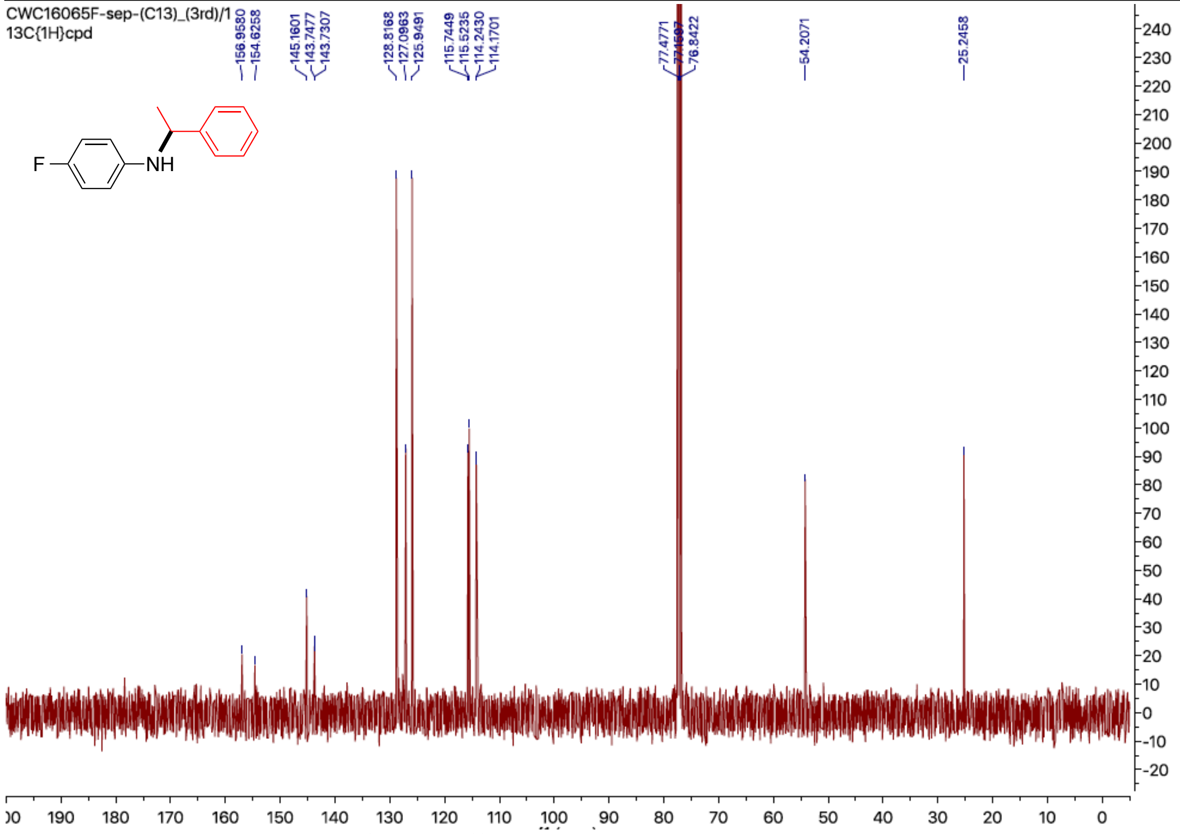

Supplementary Figure 63. ^{1}H and ^{13}C NMR spectra of 4-Fluoro-*N*-(1-phenylethyl)aniline (5q)

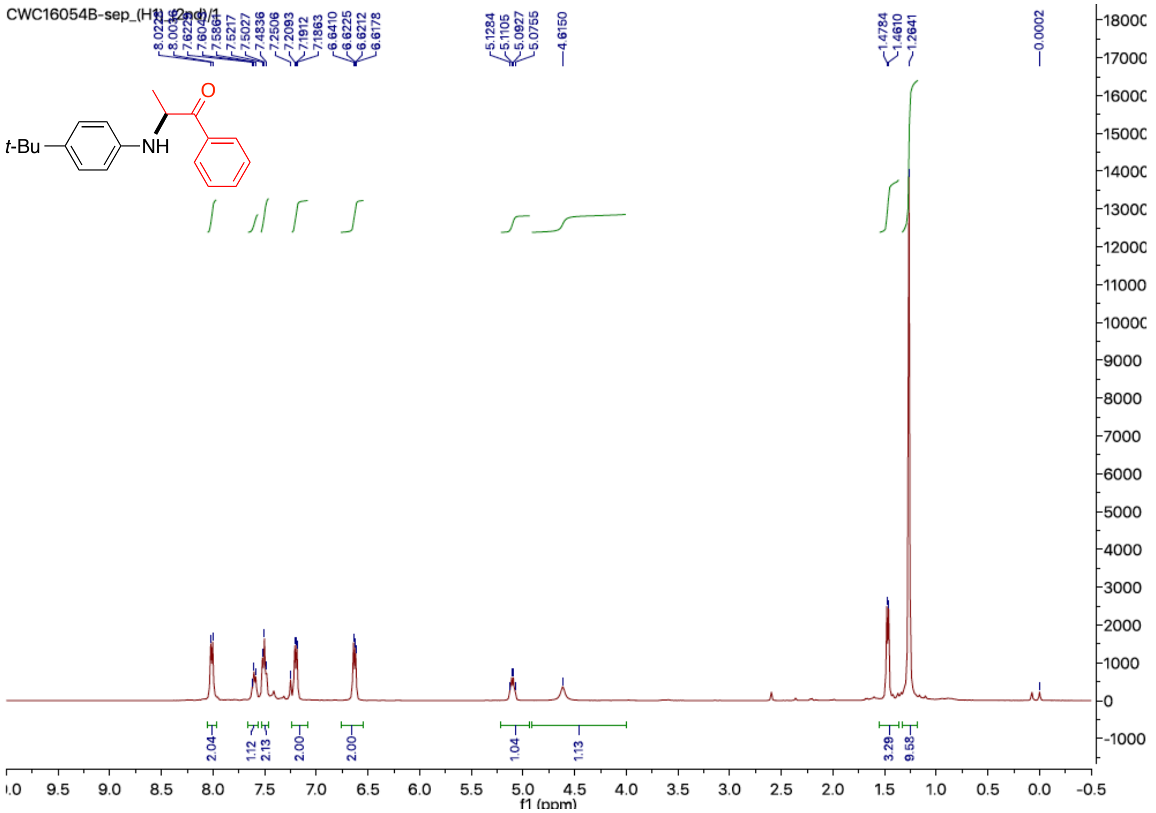

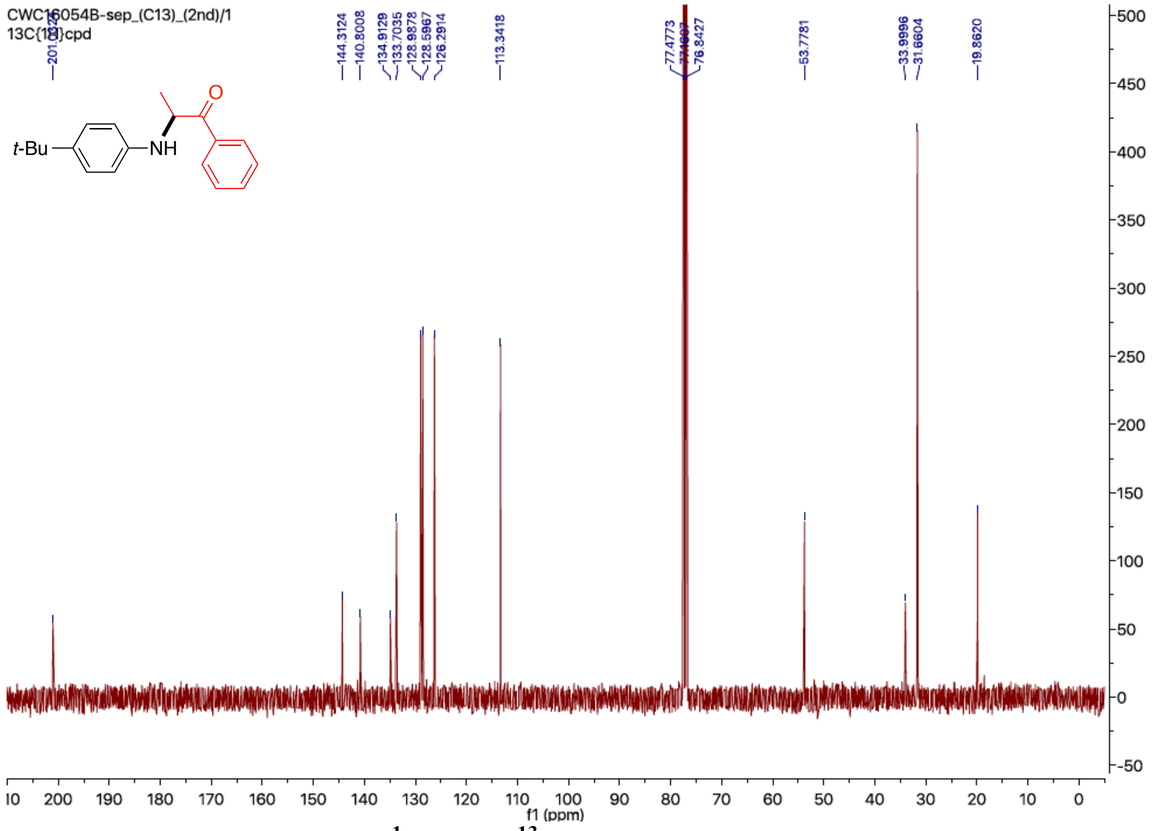

Supplementary Figure 64. ^{1}**H and** ^{13}**C NMR spectra of 2-((4-(*tert*-Butyl)phenyl)amino)-1-phenylpropan-1-one (5r)**

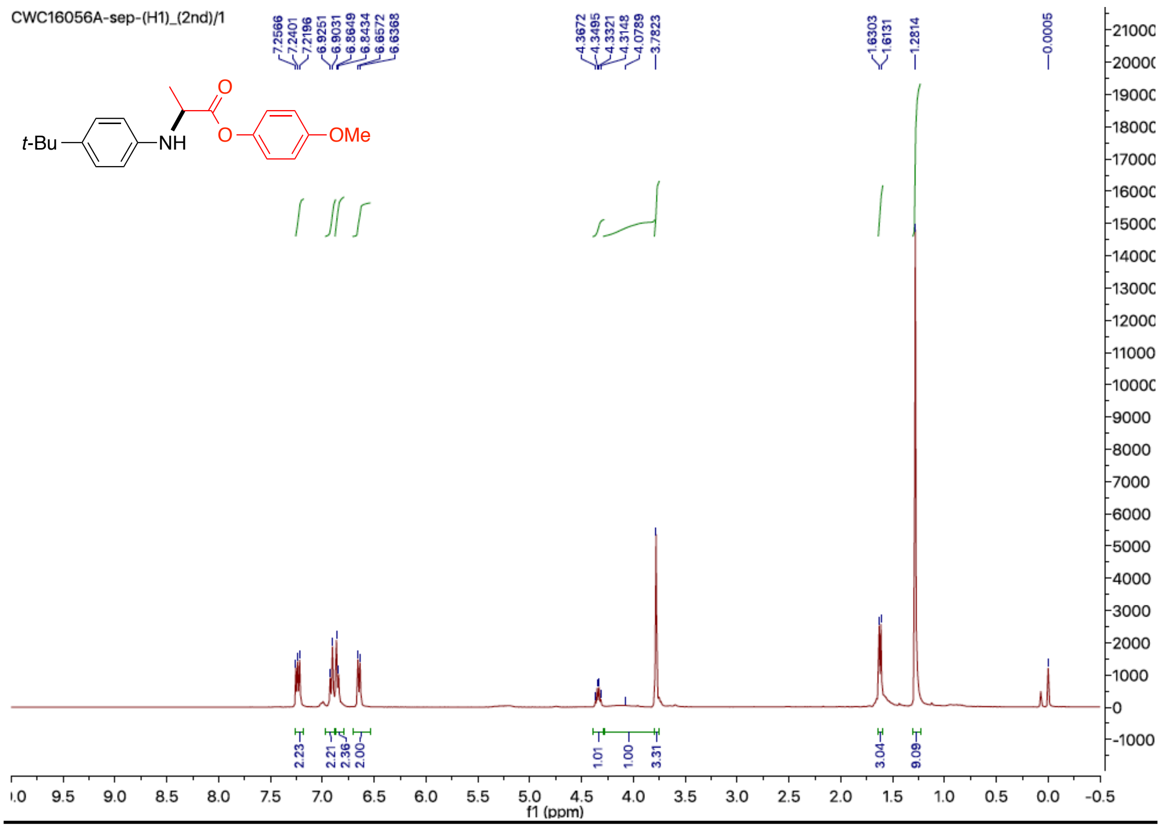

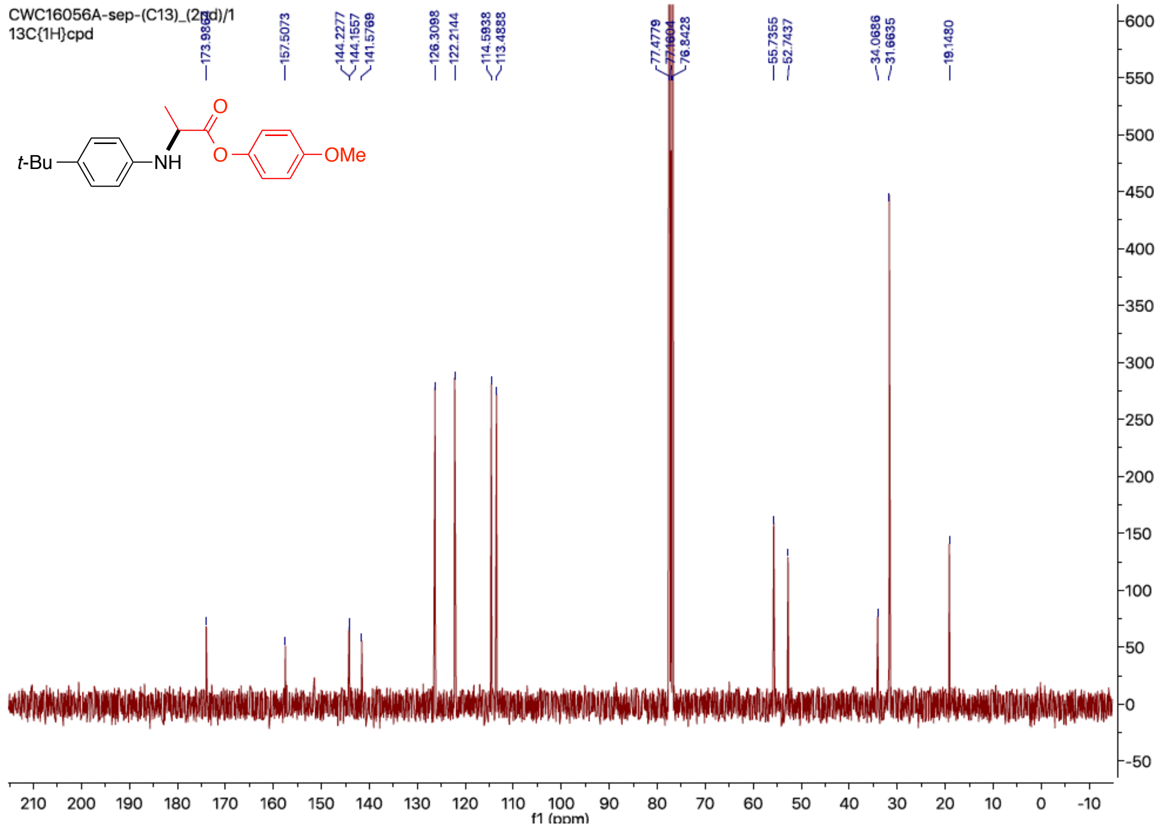

Supplementary Figure 65. ^{1}H and ^{13}C NMR spectra of 4-Methoxyphenyl (4-(*tert*-Butyl)phenyl)alaninate (5s)

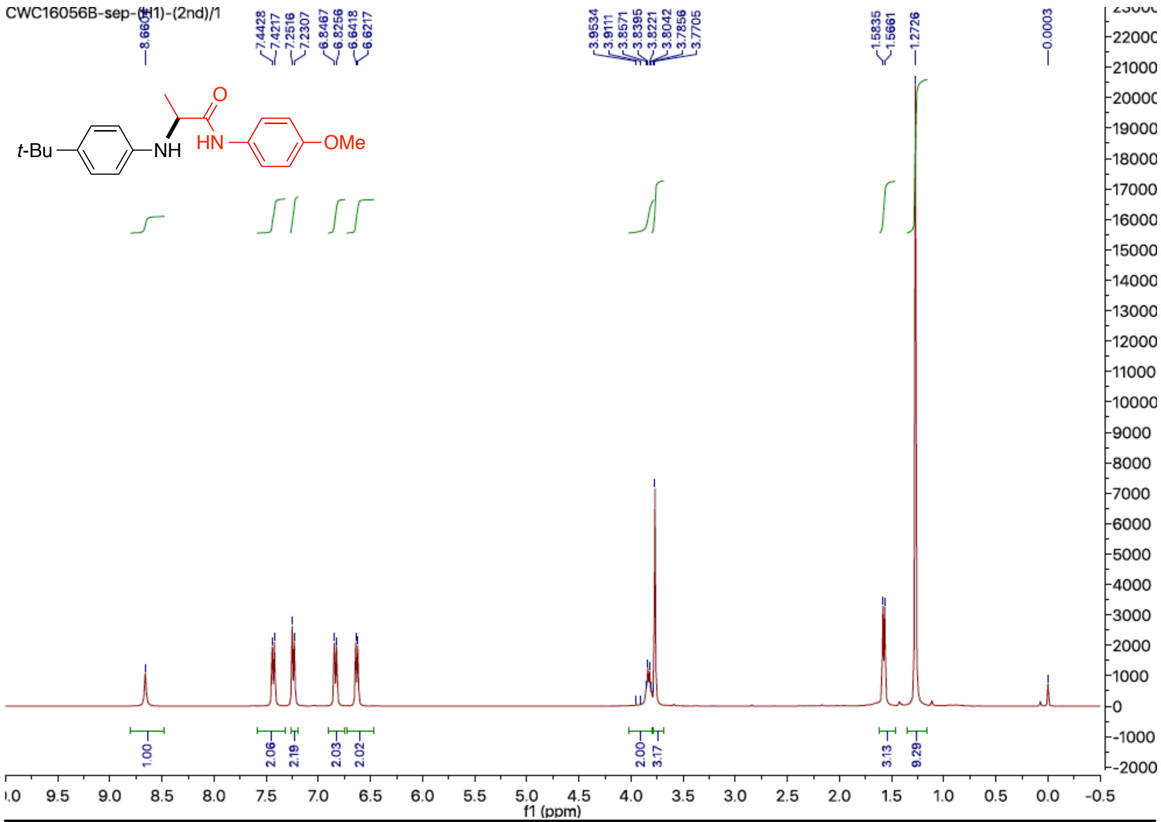

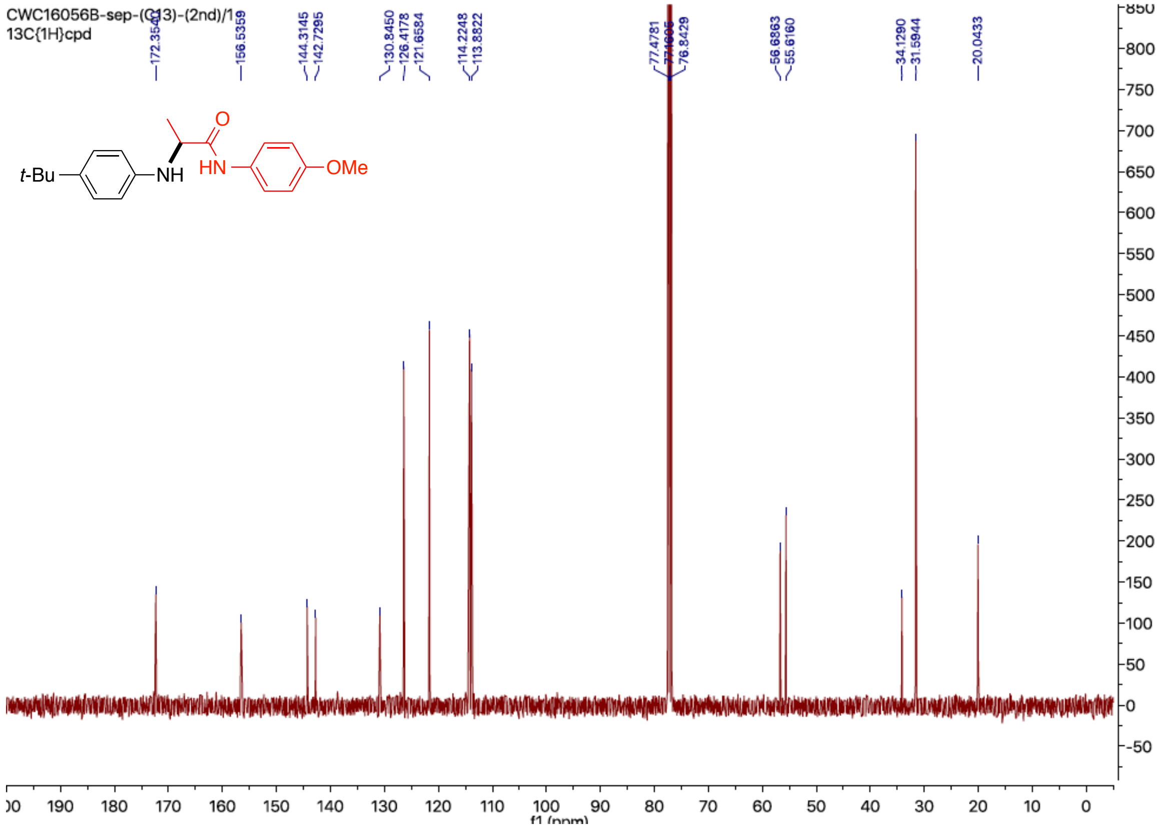

Supplementary Figure 66. ^{1}H and ^{13}C NMR spectra of 2-((4-(*tert*-Butyl)phenyl)amino)-*N*-(4-methoxyphenyl)propanamide (5t)

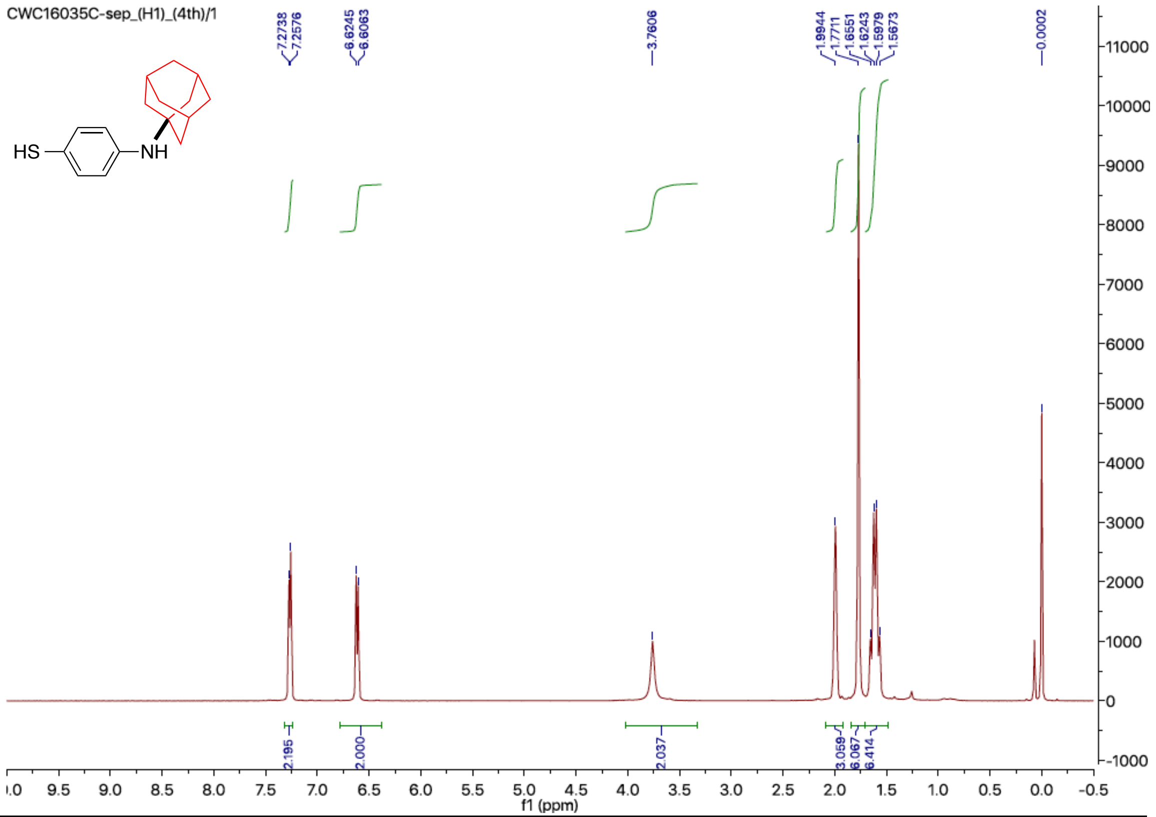

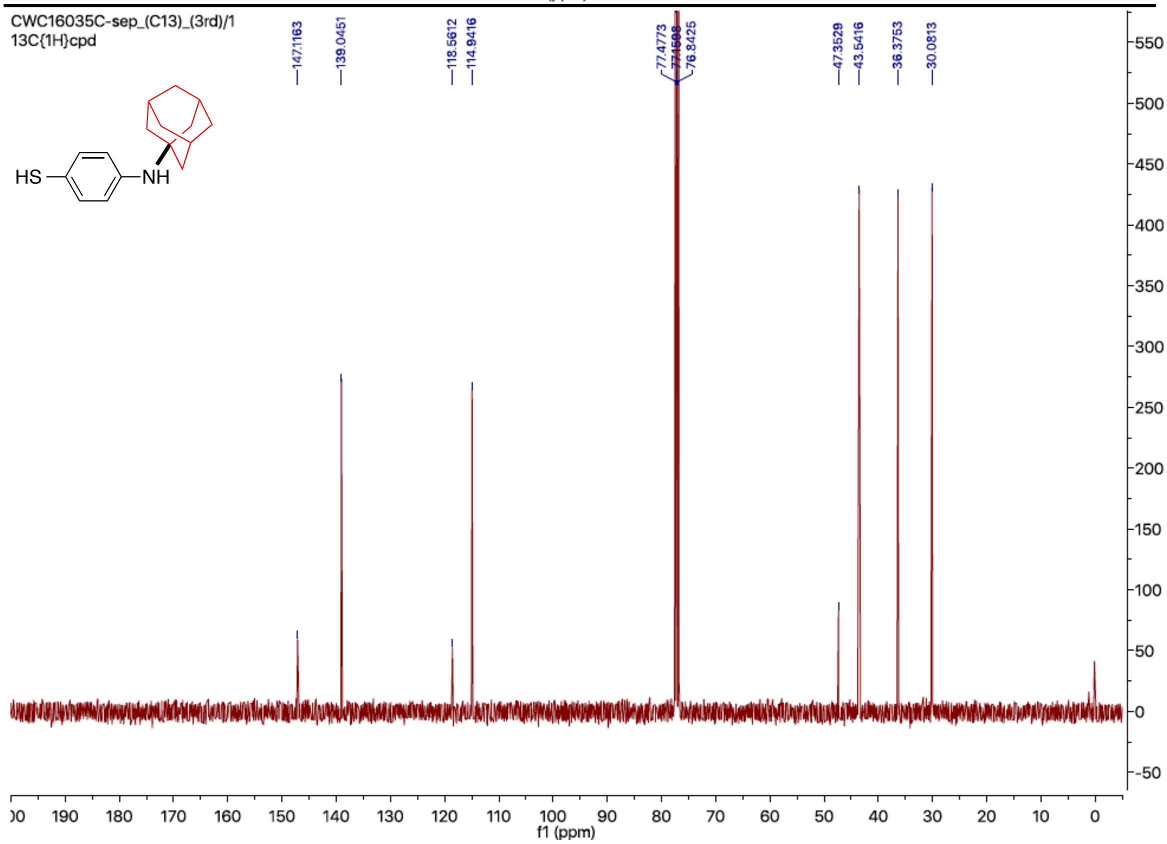

Supplementary Figure 67. ^{1}H and ^{13}C NMR spectra of 4-((Adamantan-1-yl)amino)benzenethiol (5u)

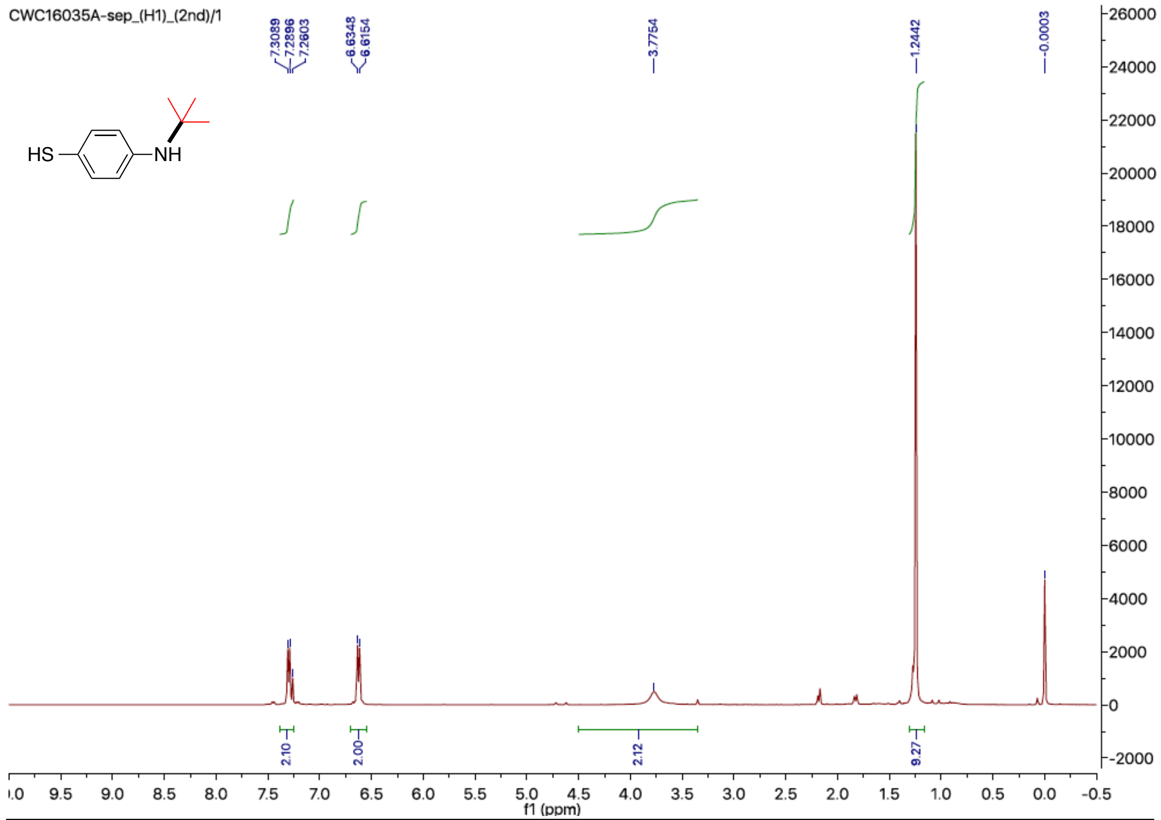

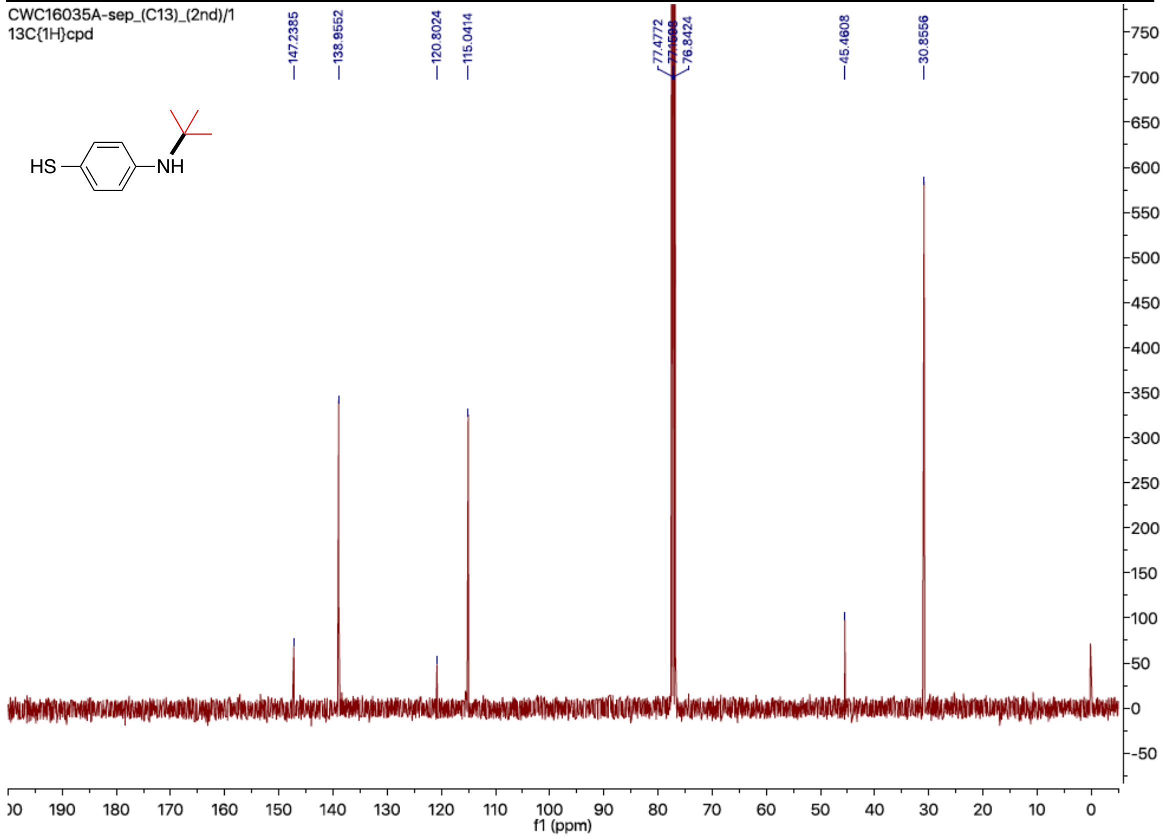

Supplementary Figure 68. ^{1}H and ^{13}C NMR spectra of 4-(*tert*-Butylamino)benzenethiol (5v)

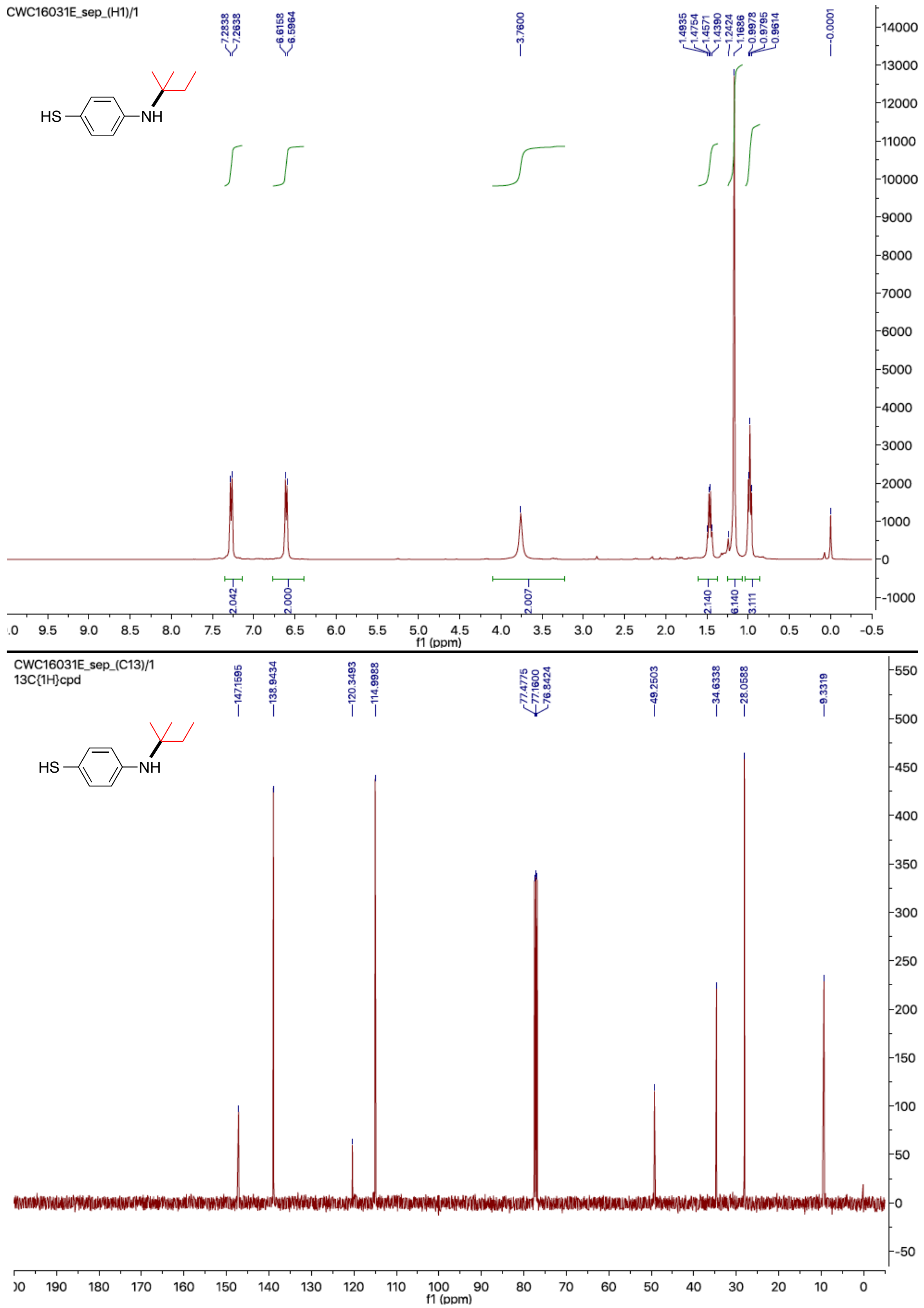

Supplementary Figure 69. ^{1}H and ^{13}C NMR spectra of 4-(*tert*-Pentylamino)benzenethiol (5w)

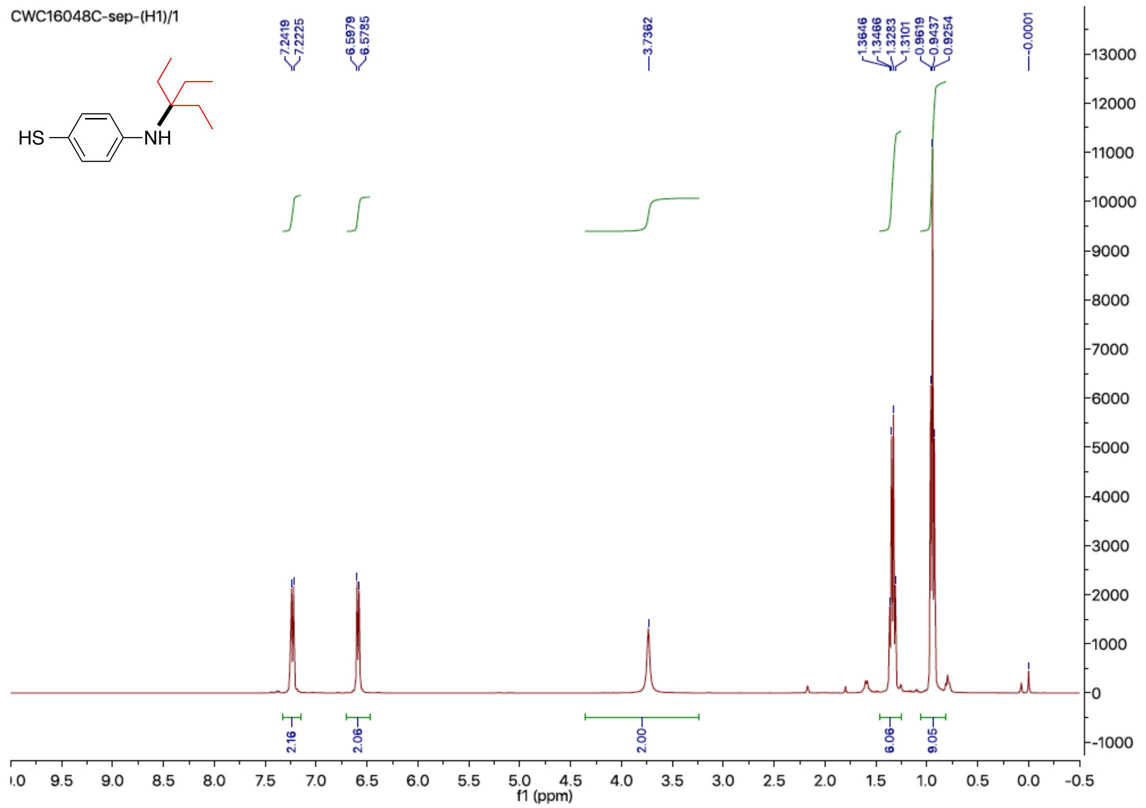

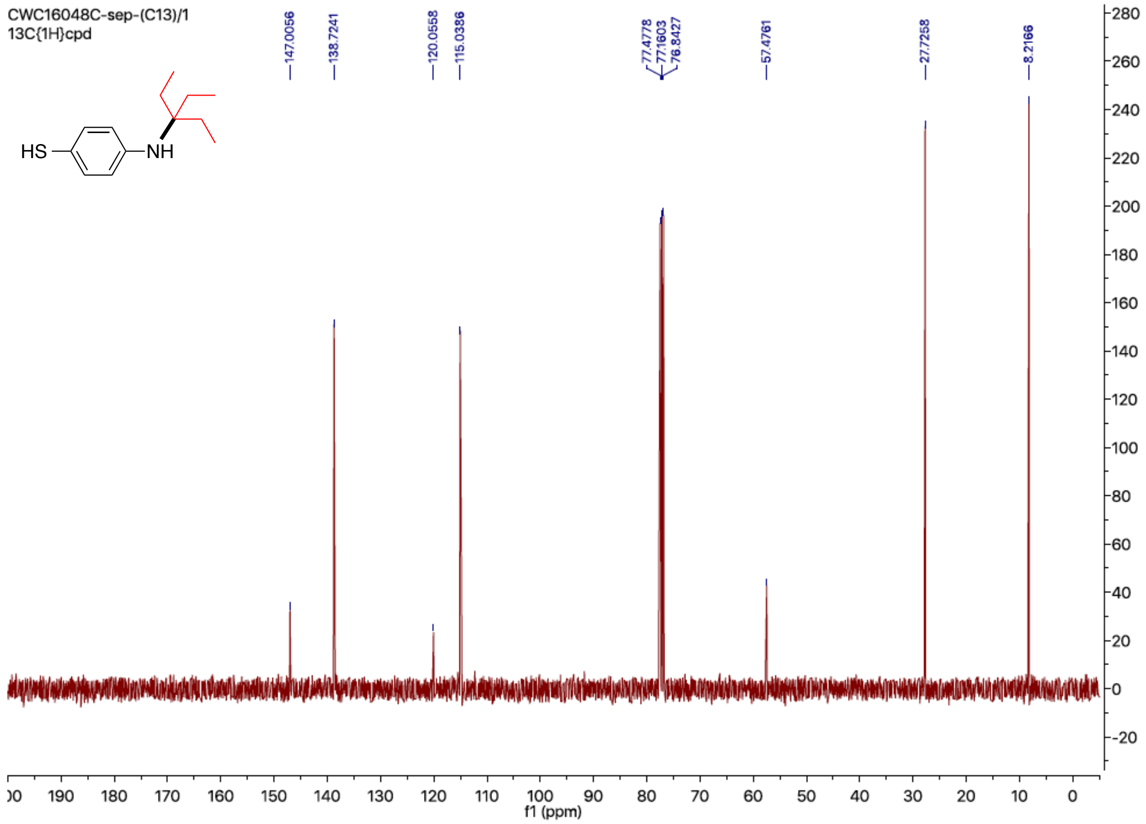

Supplementary Figure 70. **^{1}H and ^{13}C NMR spectra of 4-((3-Ethylpentan-3-yl)amino)benzenethiol (5x)**

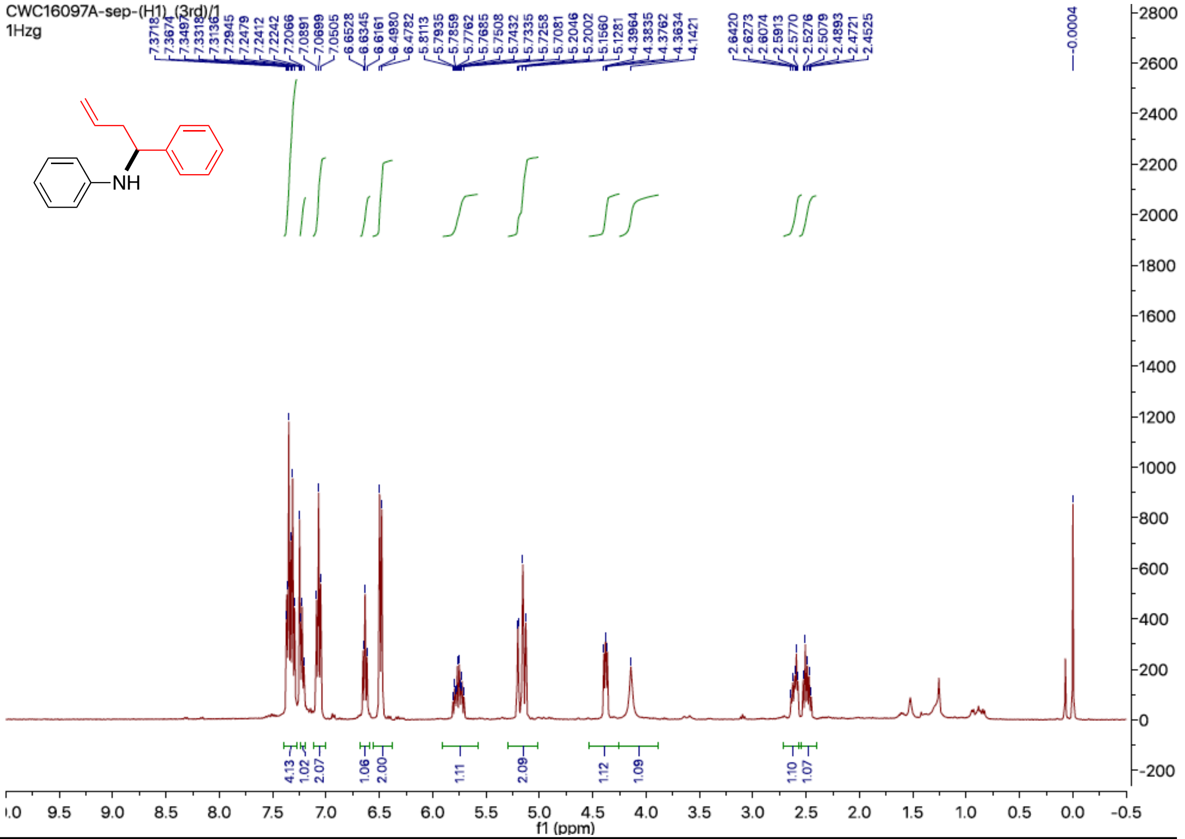

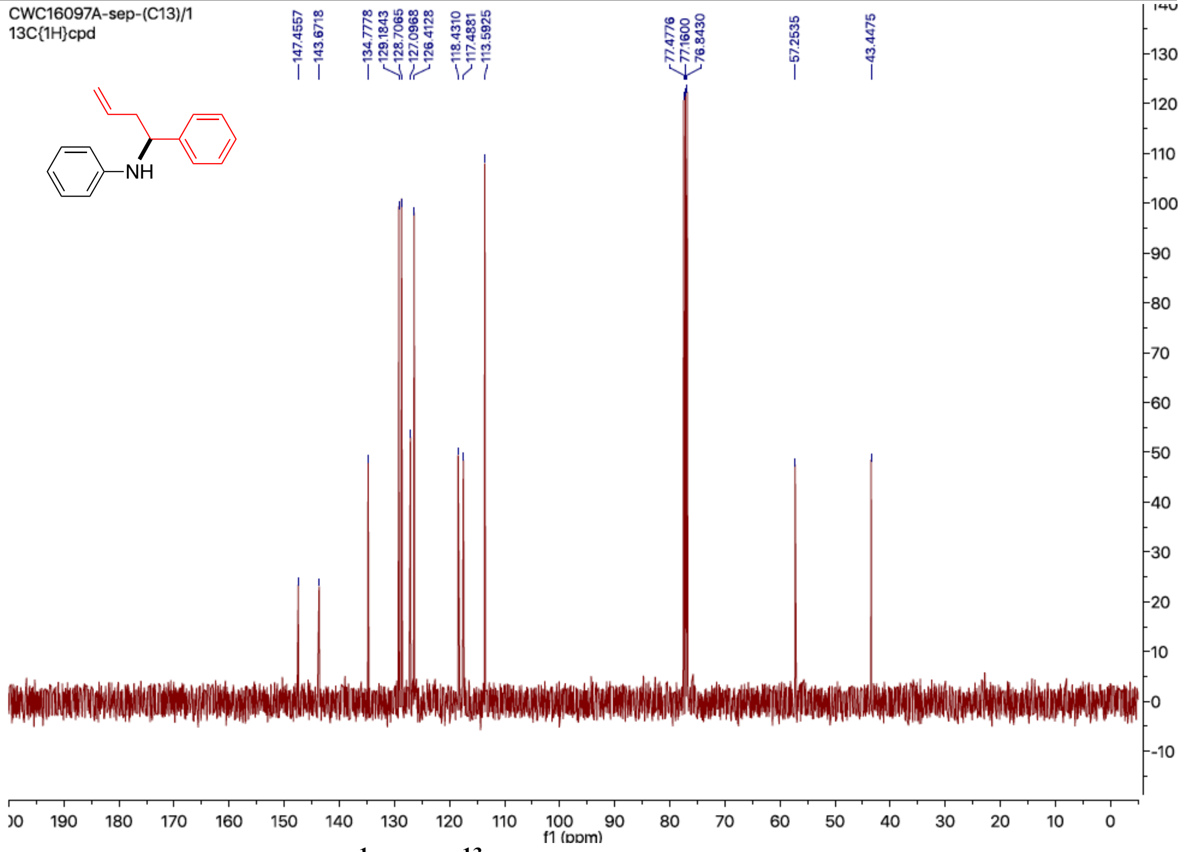

Supplementary Figure 71. ^{1}H and ^{13}C NMR spectra of *N*-(1-Phenylbut-3-en-1-yl)aniline (5y)

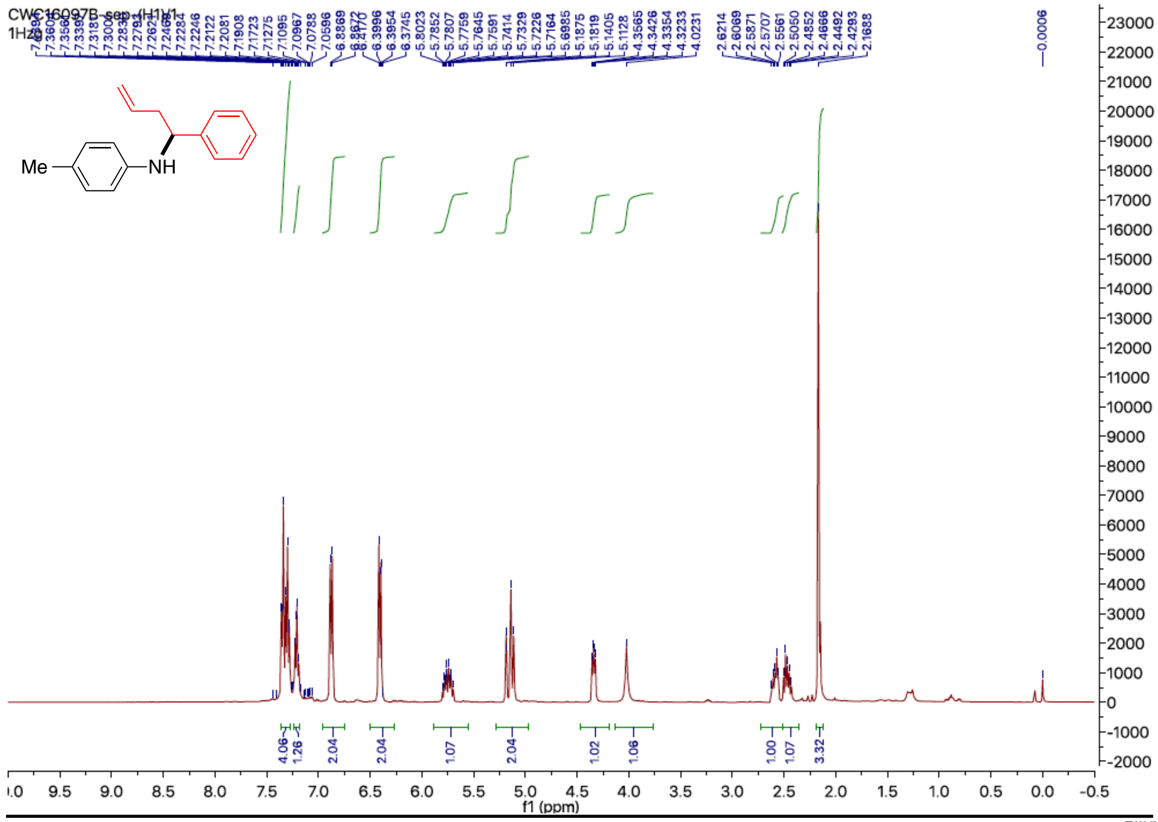

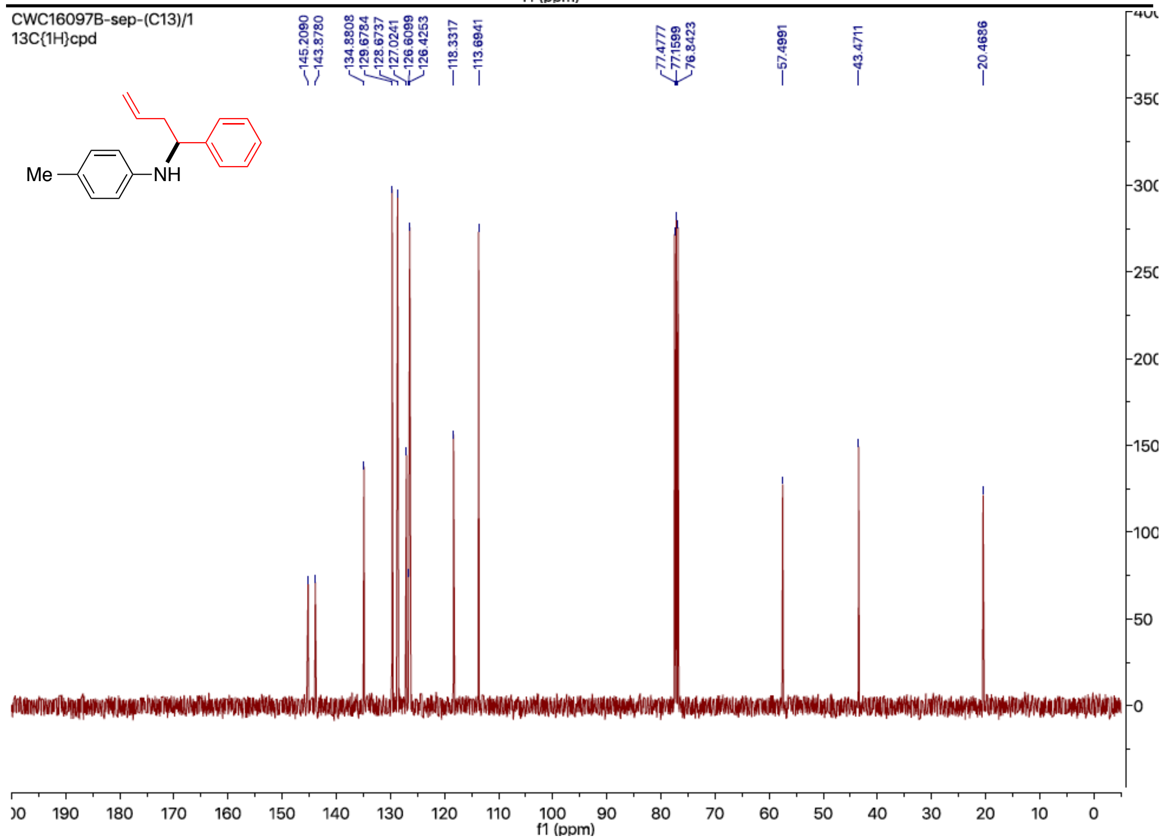

Supplementary Figure 72. ^{1}H and ^{13}C NMR spectra of 4-Methyl-*N*-(1-phenylbut-3-en-1-yl)aniline (5z)

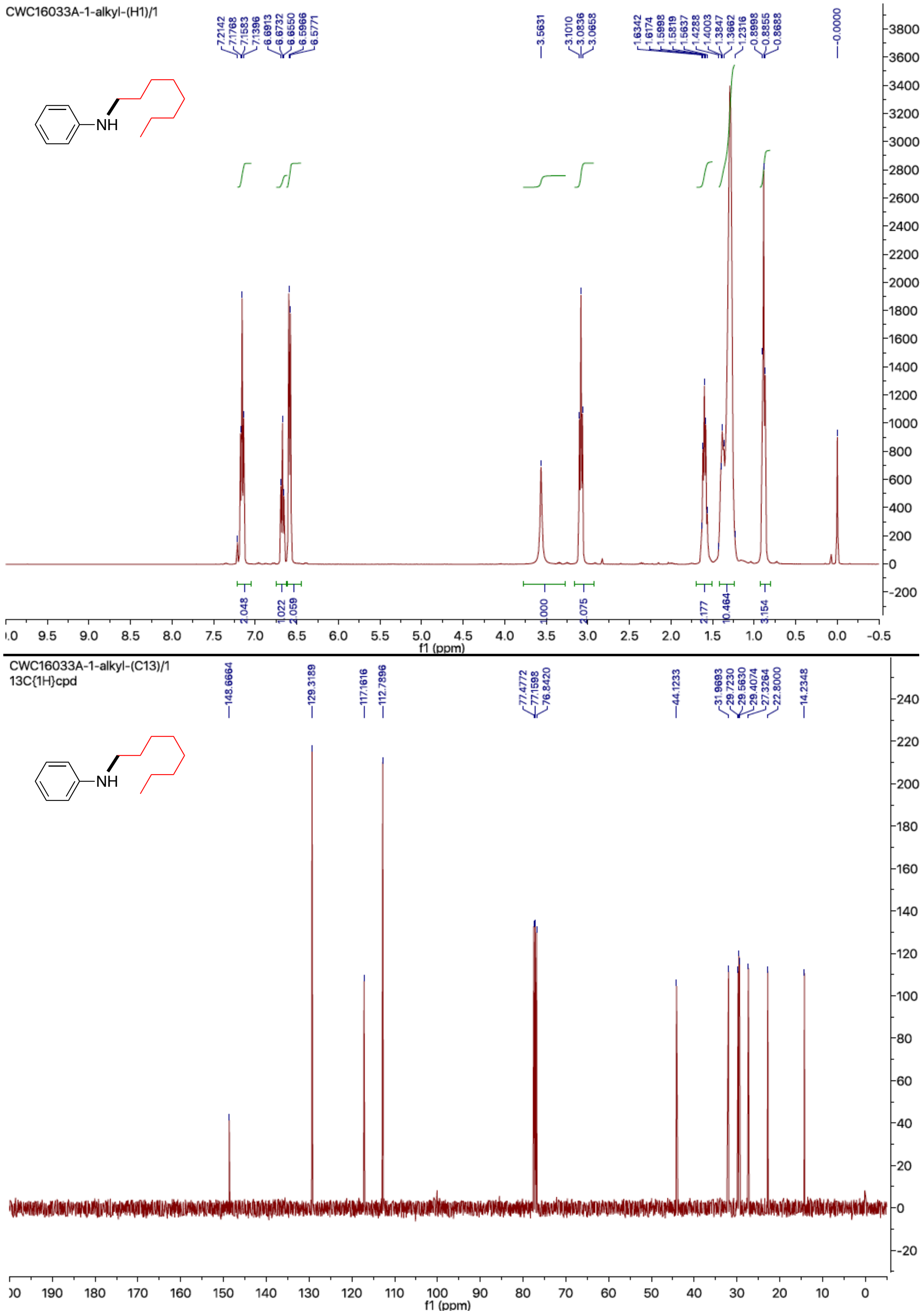

Supplementary Figure 73. ^{1}H and ^{13}C NMR spectra of *N*-Octylaniline (6a)

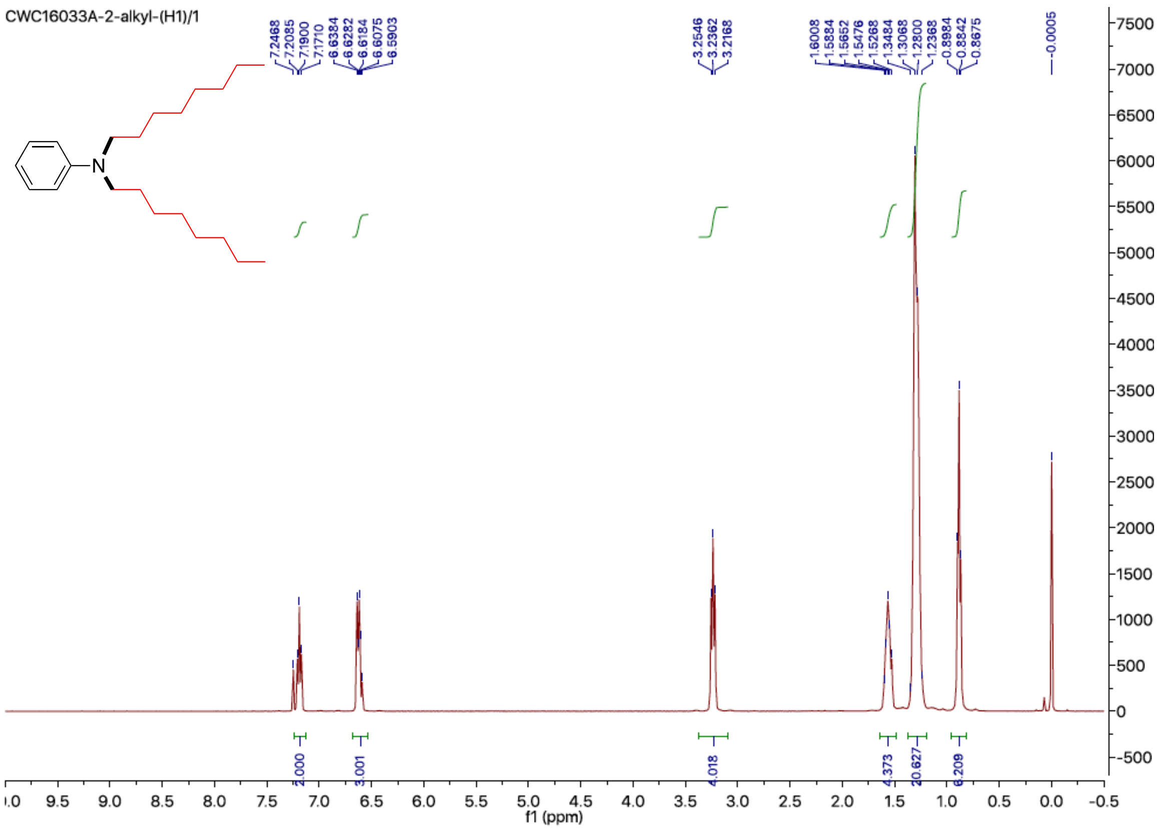

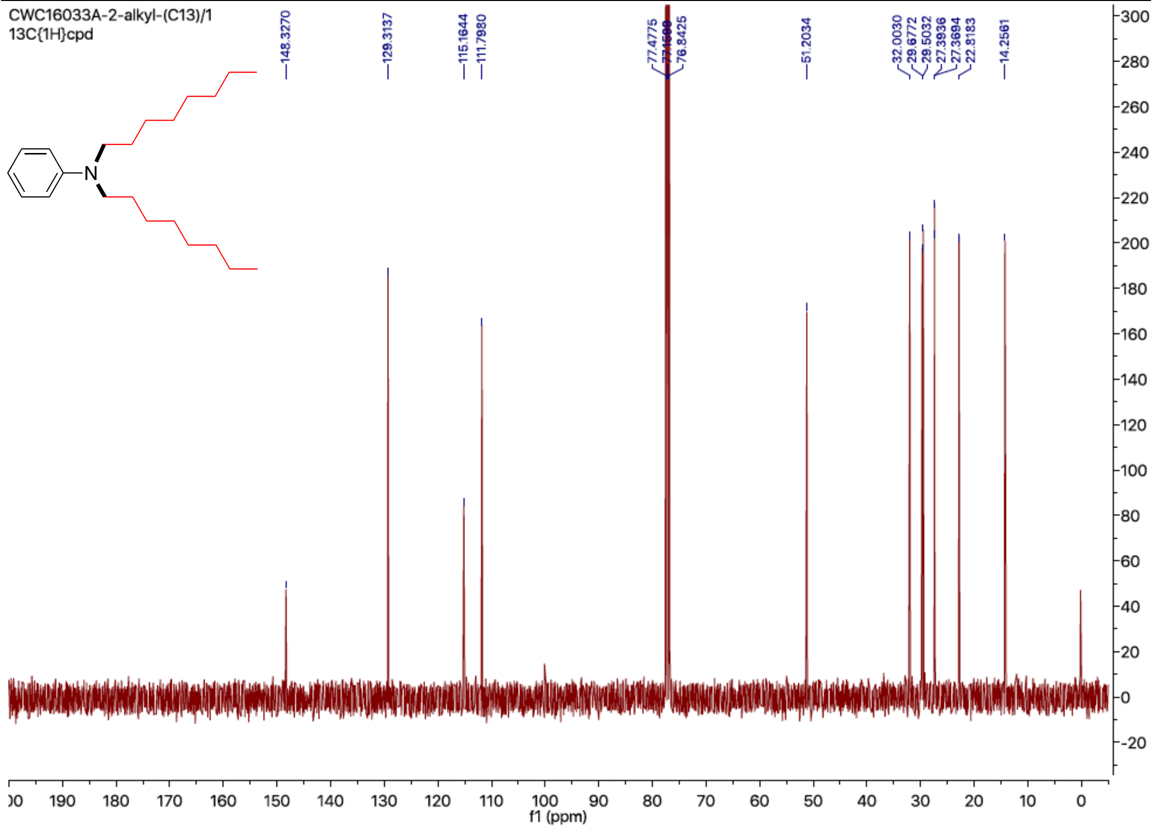

Supplementary Figure 74. ^{1}H and ^{13}C NMR spectra of *N*,*N*-Dioctylaniline (7a)

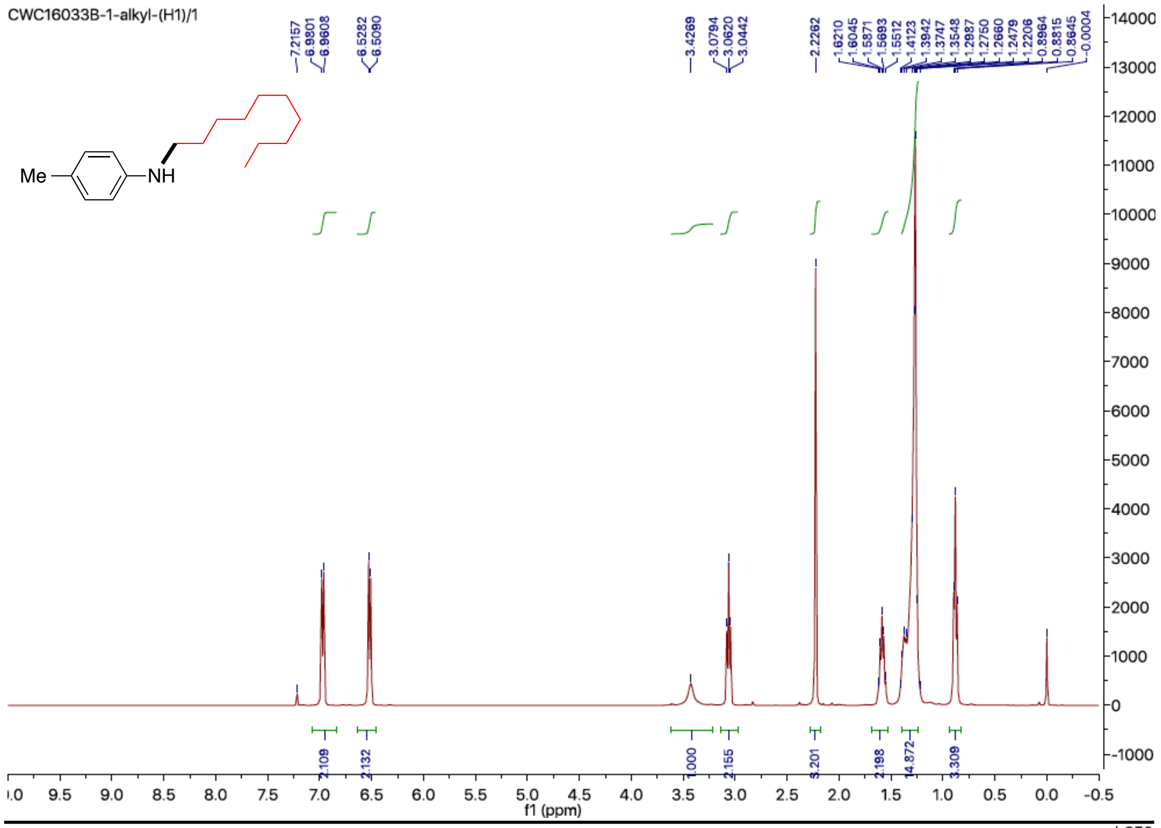

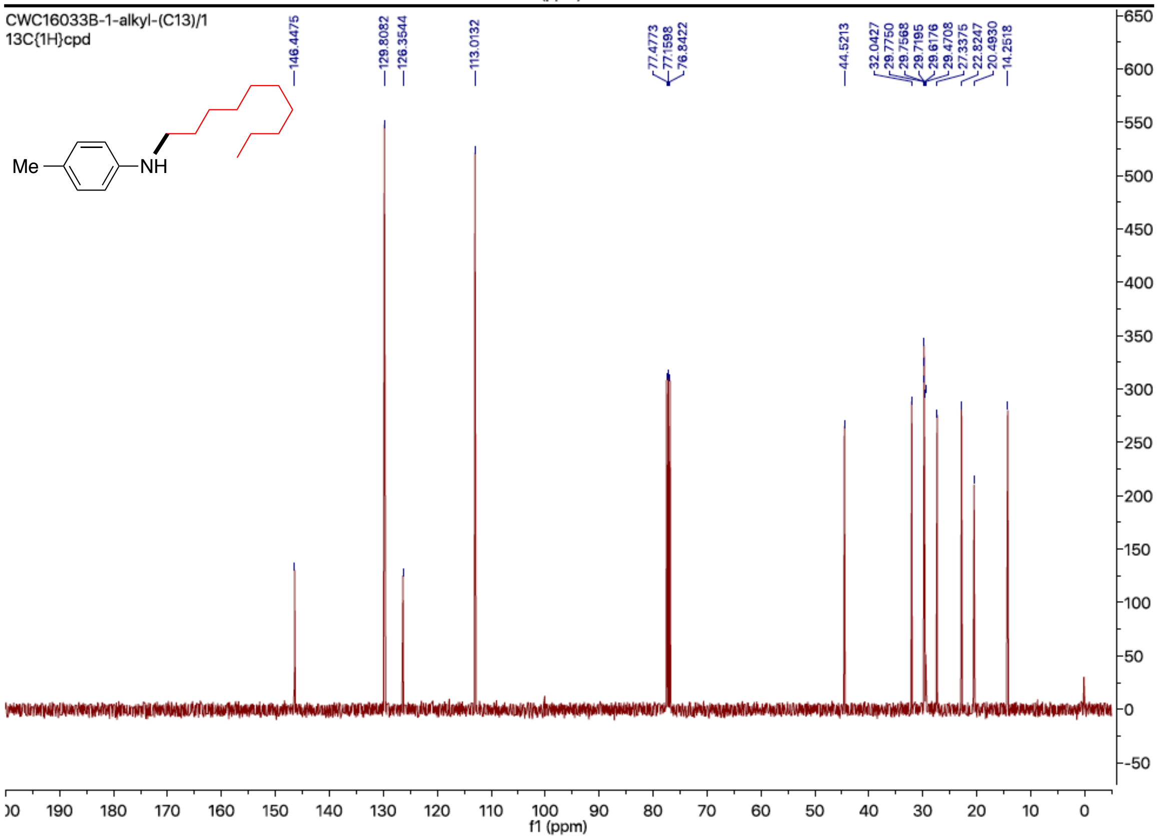

Supplementary Figure 75. ^{1}H and ^{13}C NMR spectra of *N*-Decyl-4-methylaniline (6b)

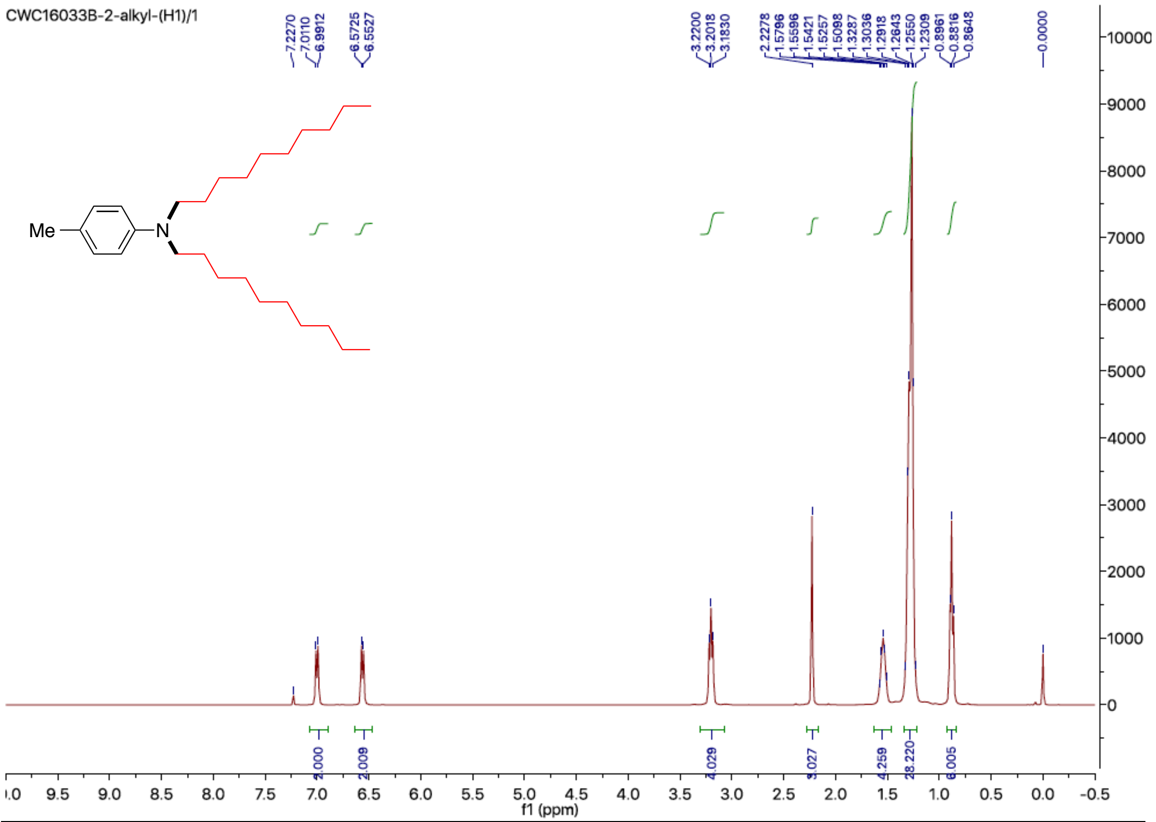

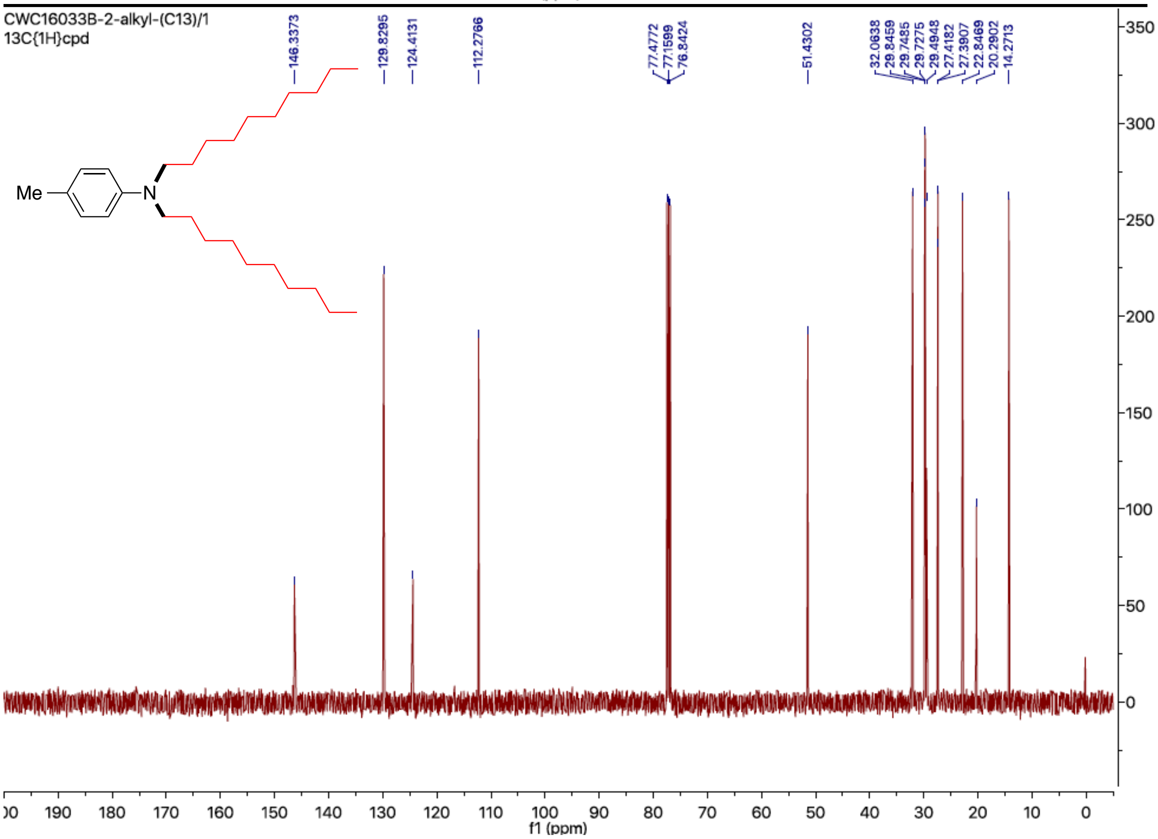

Supplementary Figure 76. ^{1}**H and** ^{13}**C NMR spectra of *N*,*N*-Didecyl-4-methylaniline (7b)**

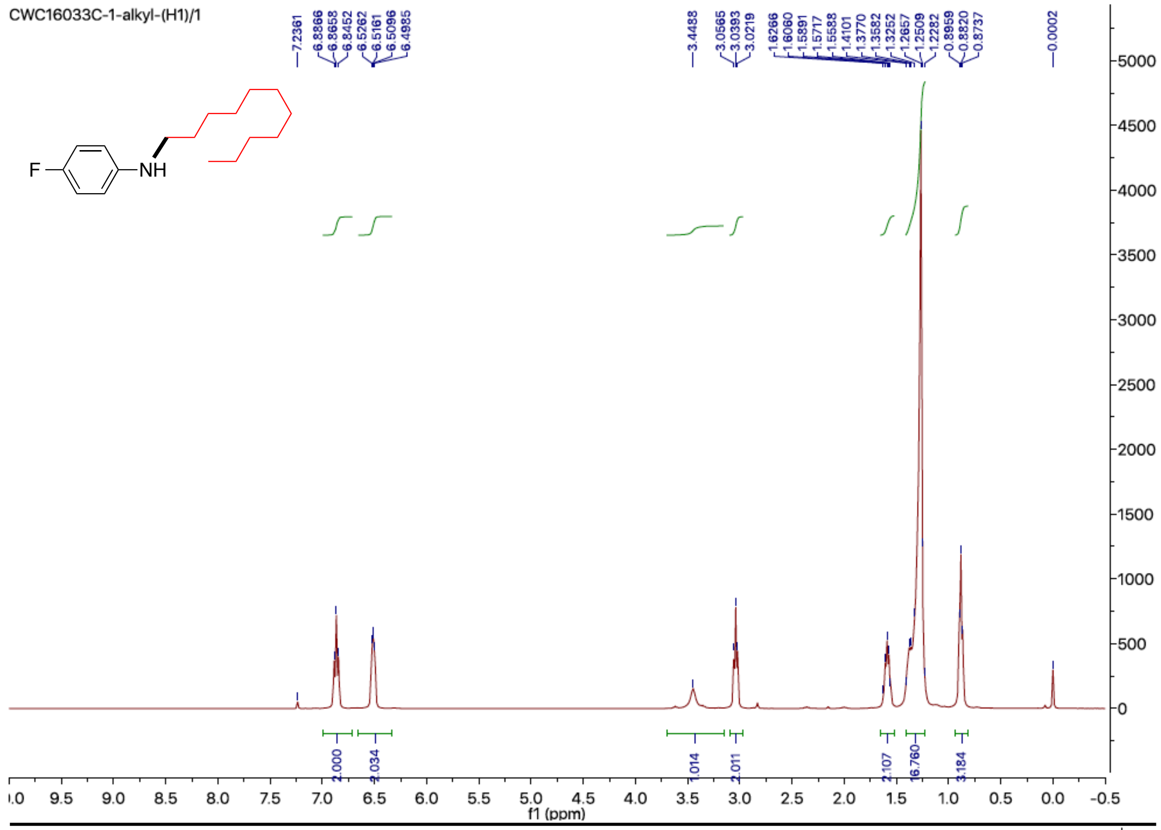

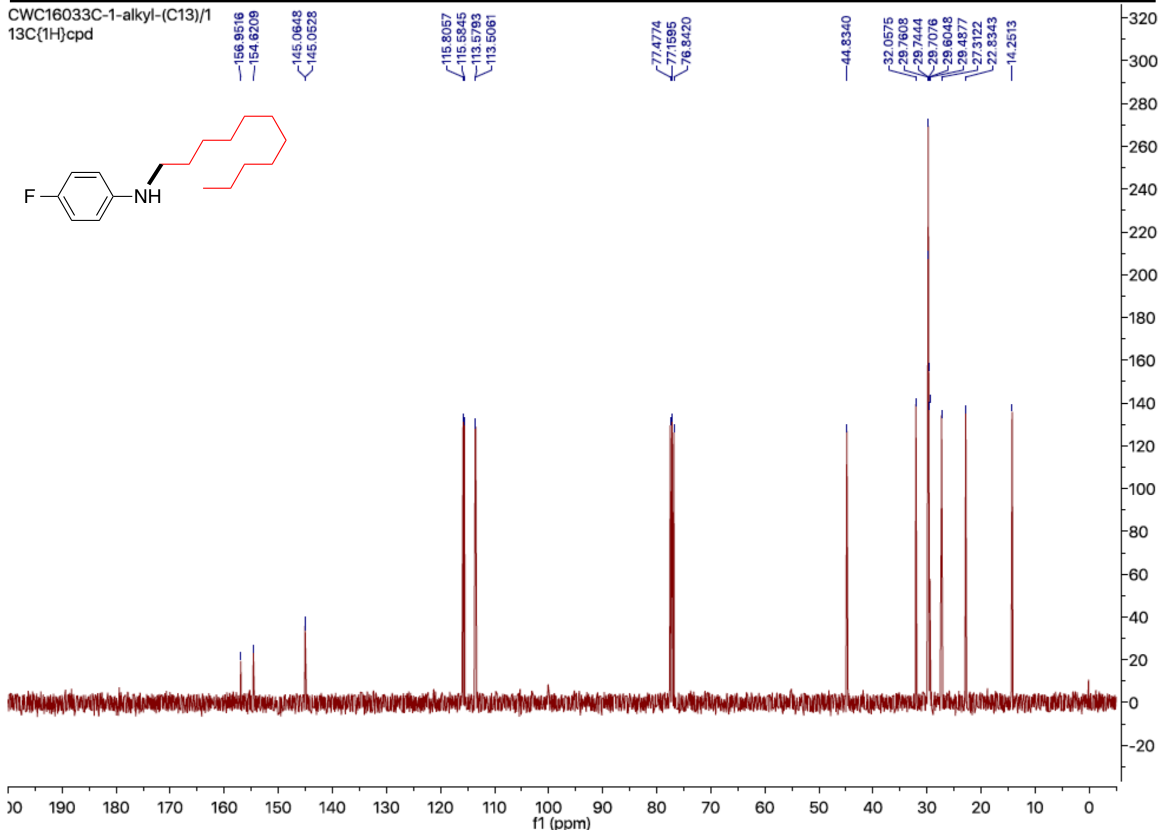

Supplementary Figure 77. ^{1}**H and** ^{13}**C NMR spectra of 4-Fluoro-*N*-undecylaniline (6c)**

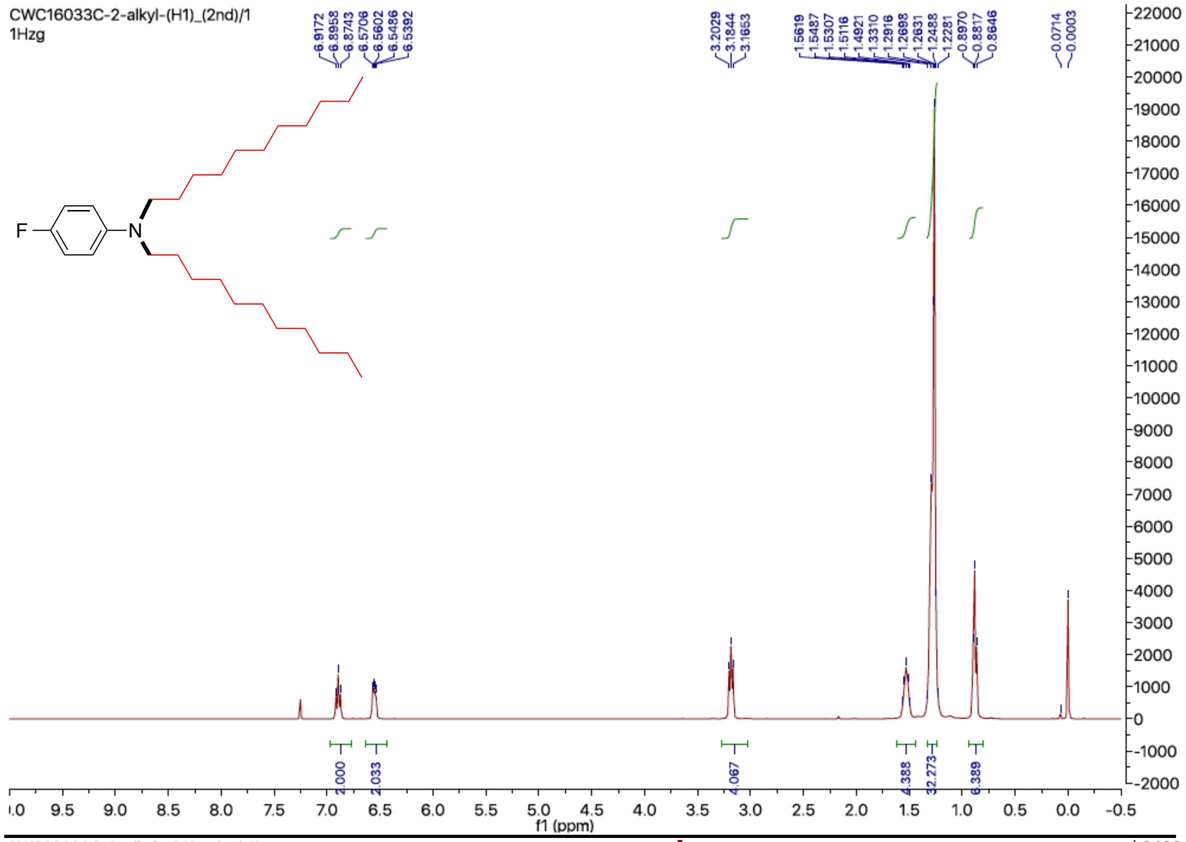

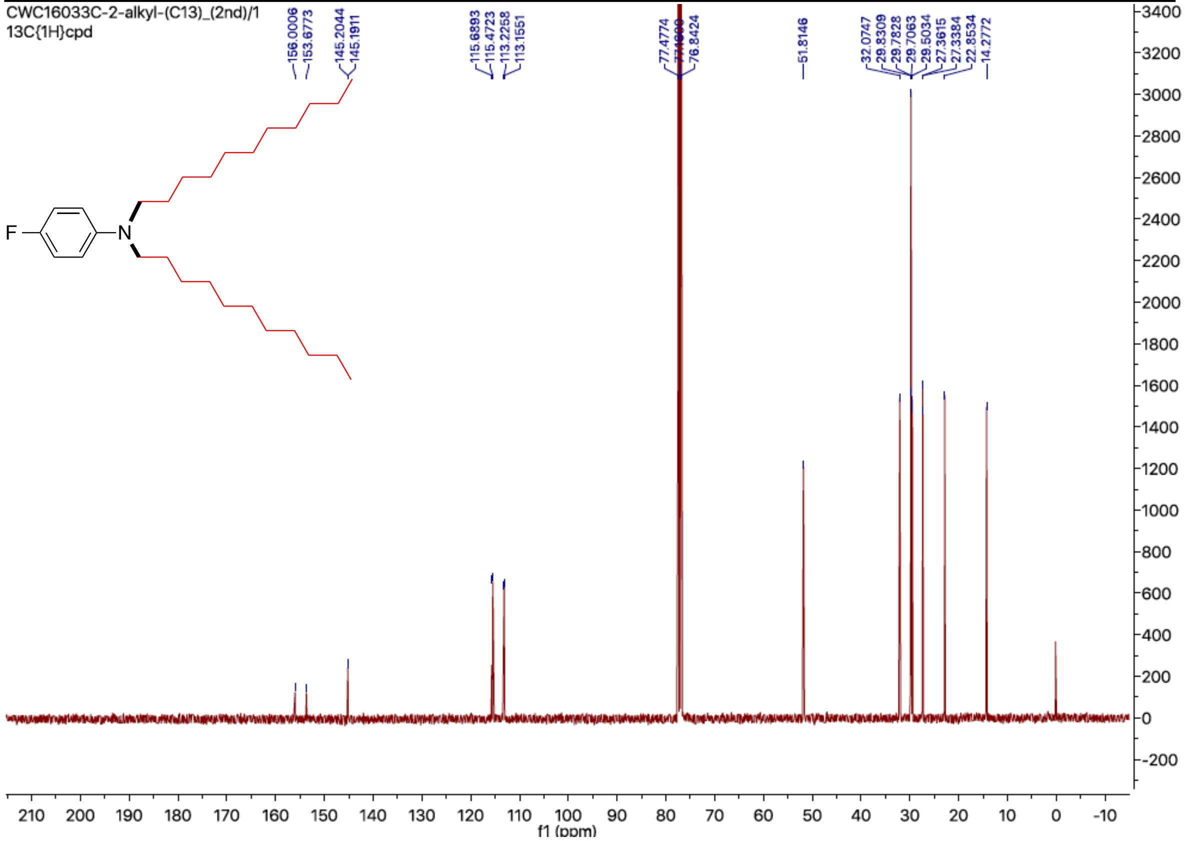

Supplementary Figure 78. ^{1}H and ^{13}C NMR spectra of 4-Fluoro-*N*,*N*-diundecylaniline (7c)

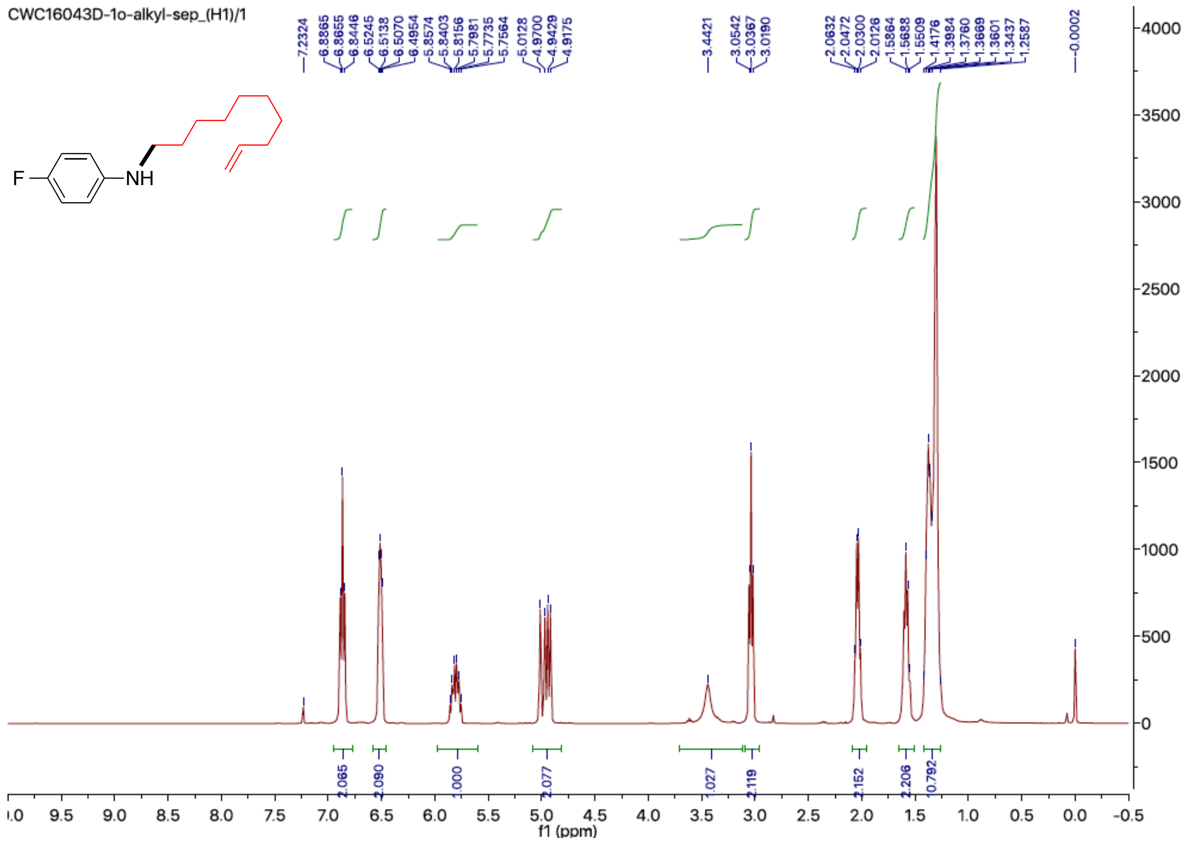

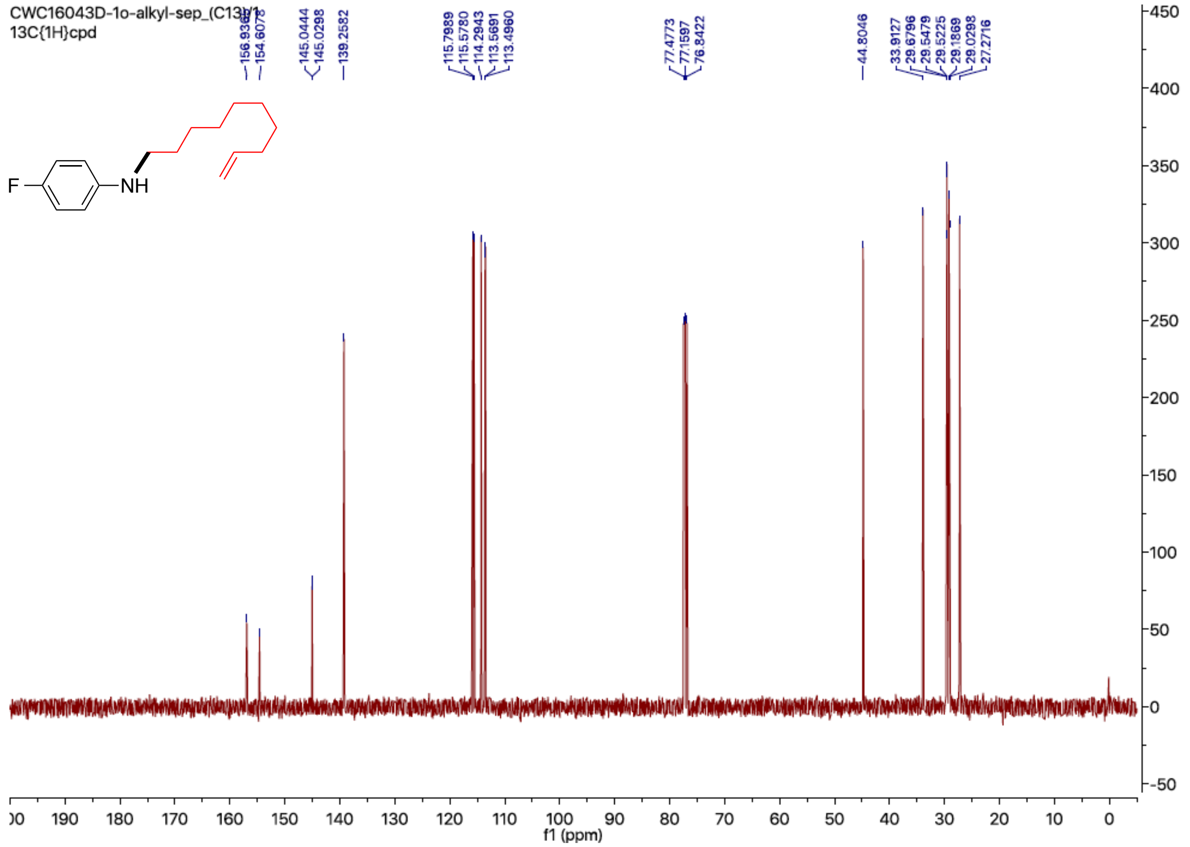

Supplementary Figure 79. ^{1}H and ^{13}C NMR spectra of *N*-(Dec-9-en-1-yl)-4-fluoroaniline (6d)

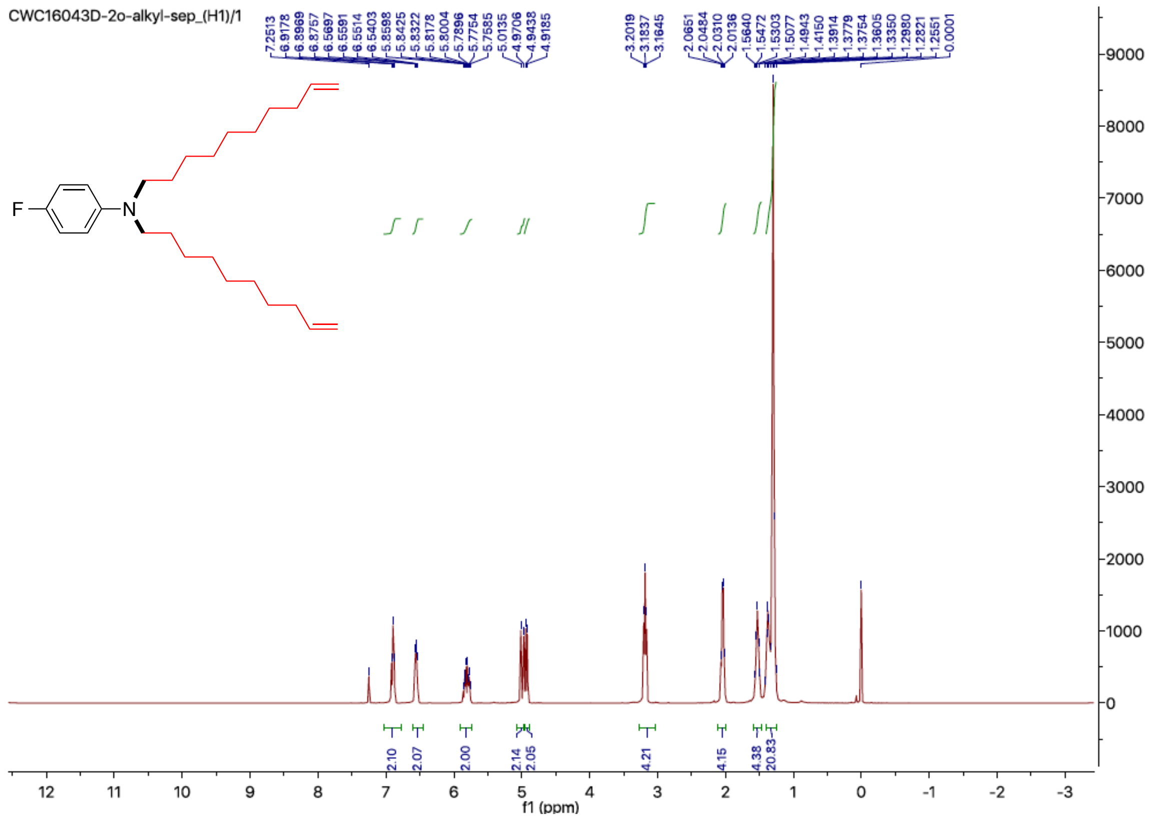

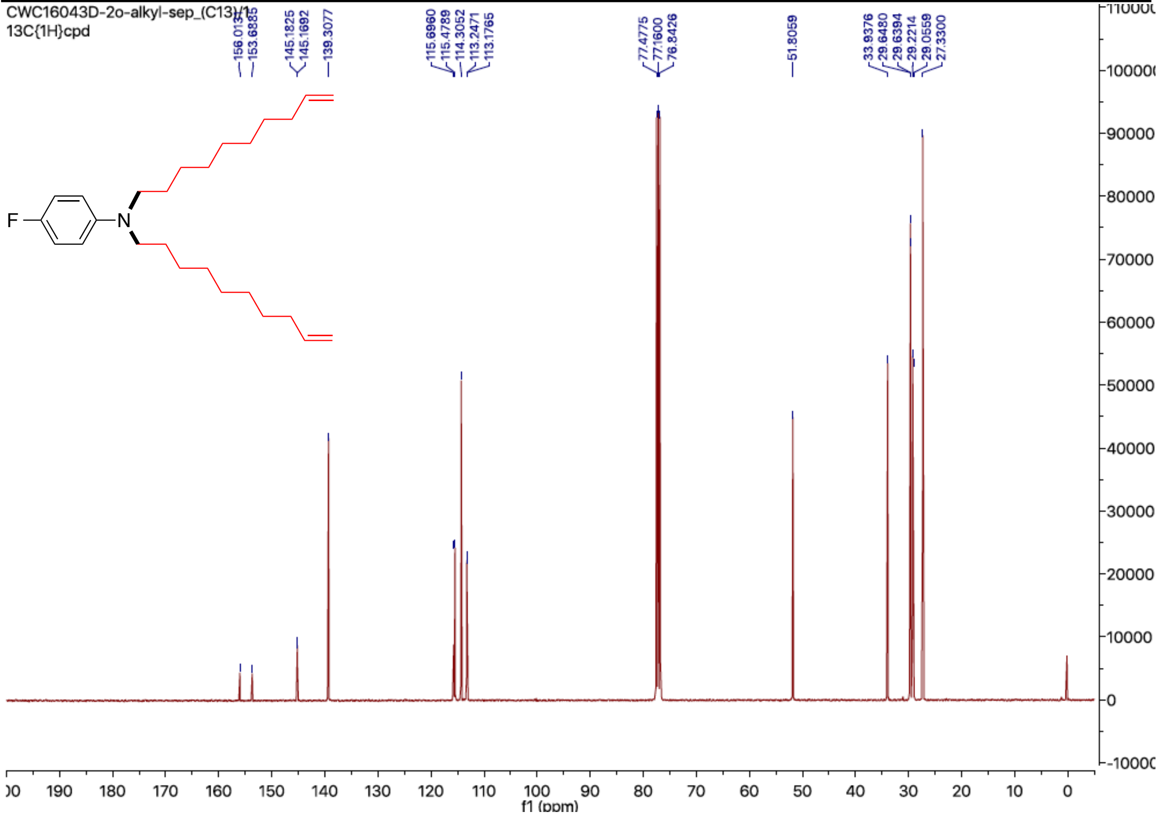

Supplementary Figure 80. ^{1}**H and** ^{13}**C NMR spectra of *N*,*N*-Di(dec-9-en-1-yl)-4-fluoroaniline (7d)**

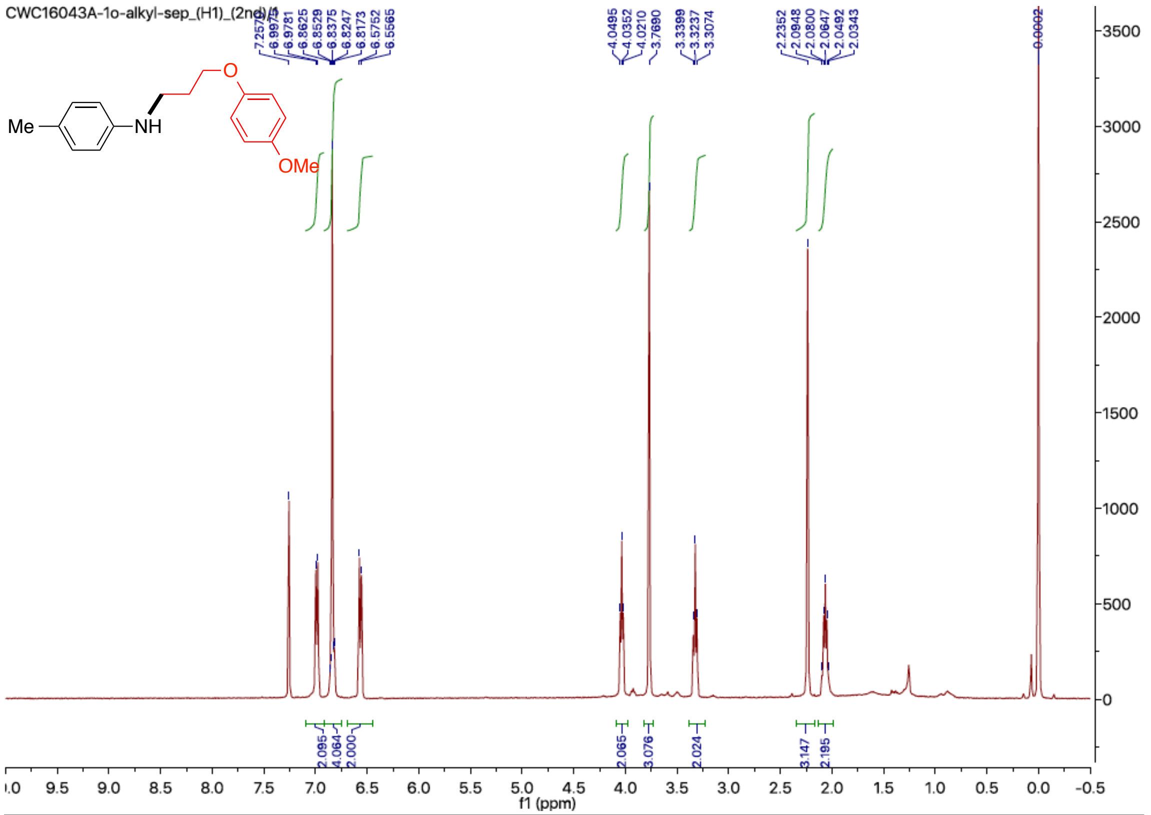

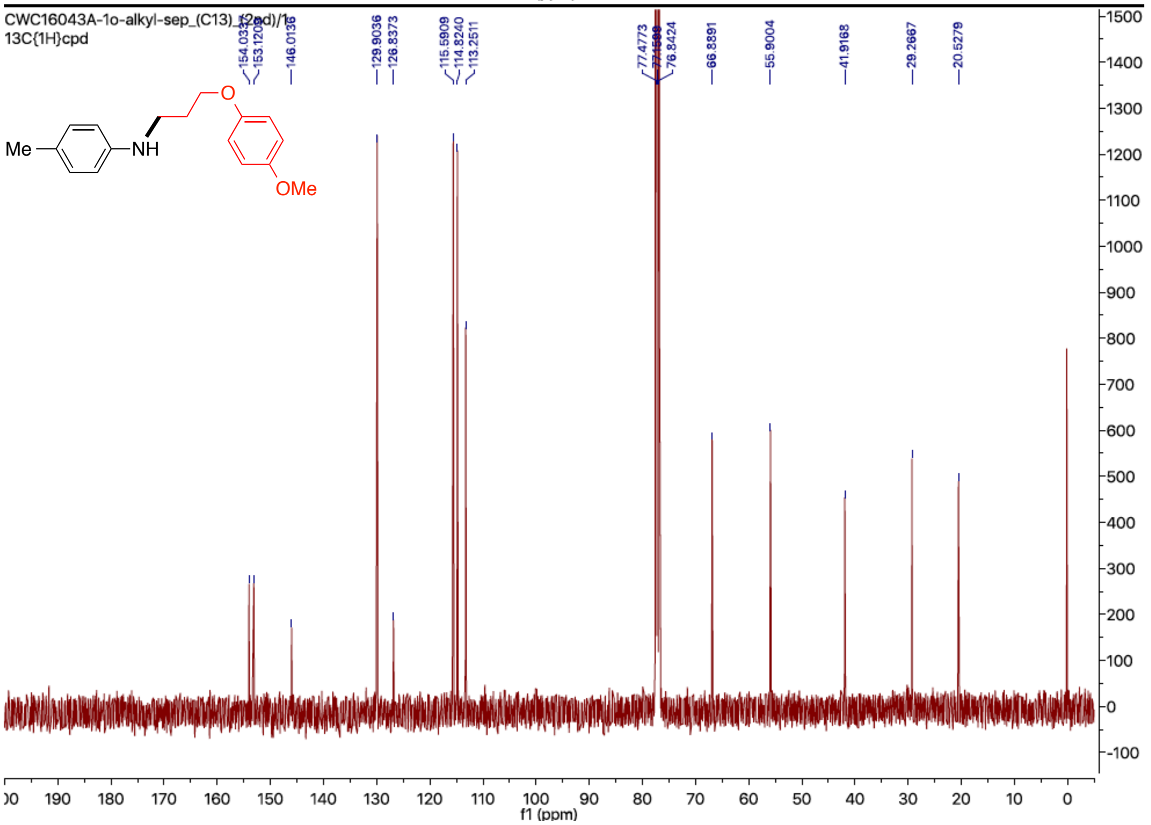

Supplementary Figure 81. ^{1}**H and** ^{13}**C NMR spectra of *N*-(3-(4-Methoxyphenoxy)propyl)-4-methylaniline (6e)**

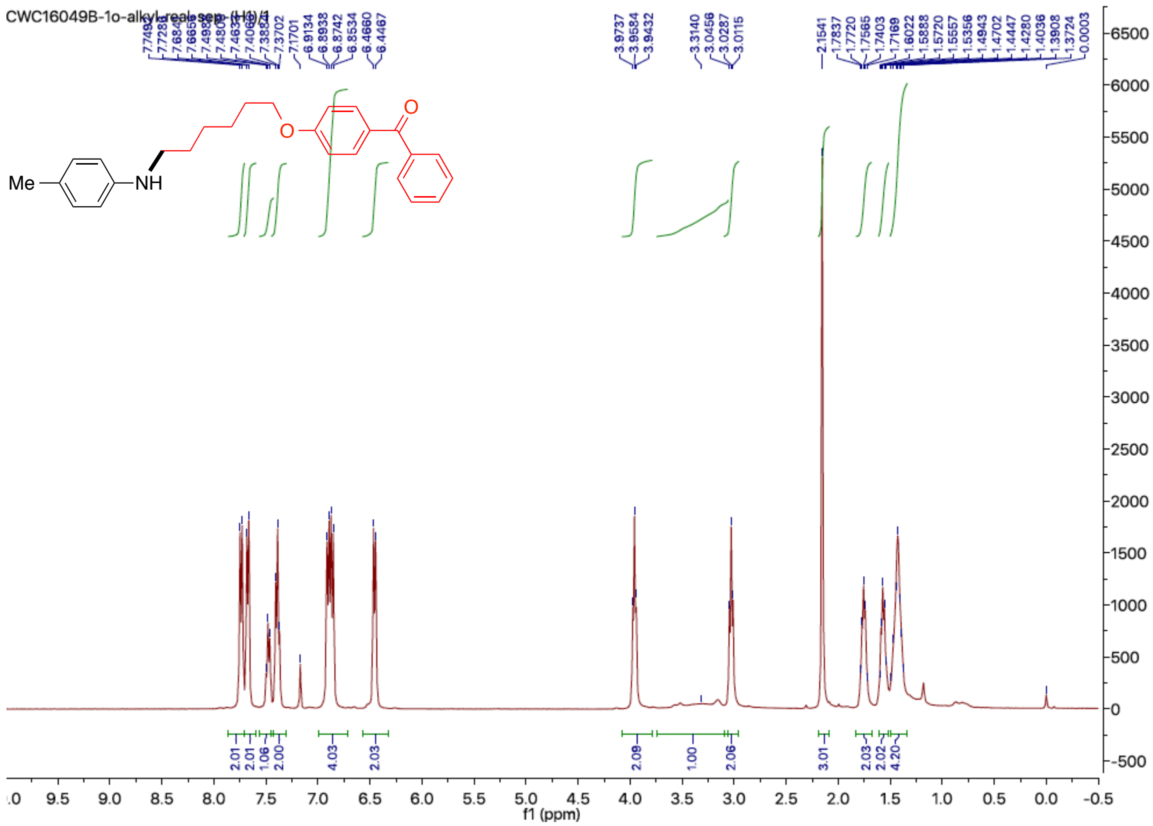

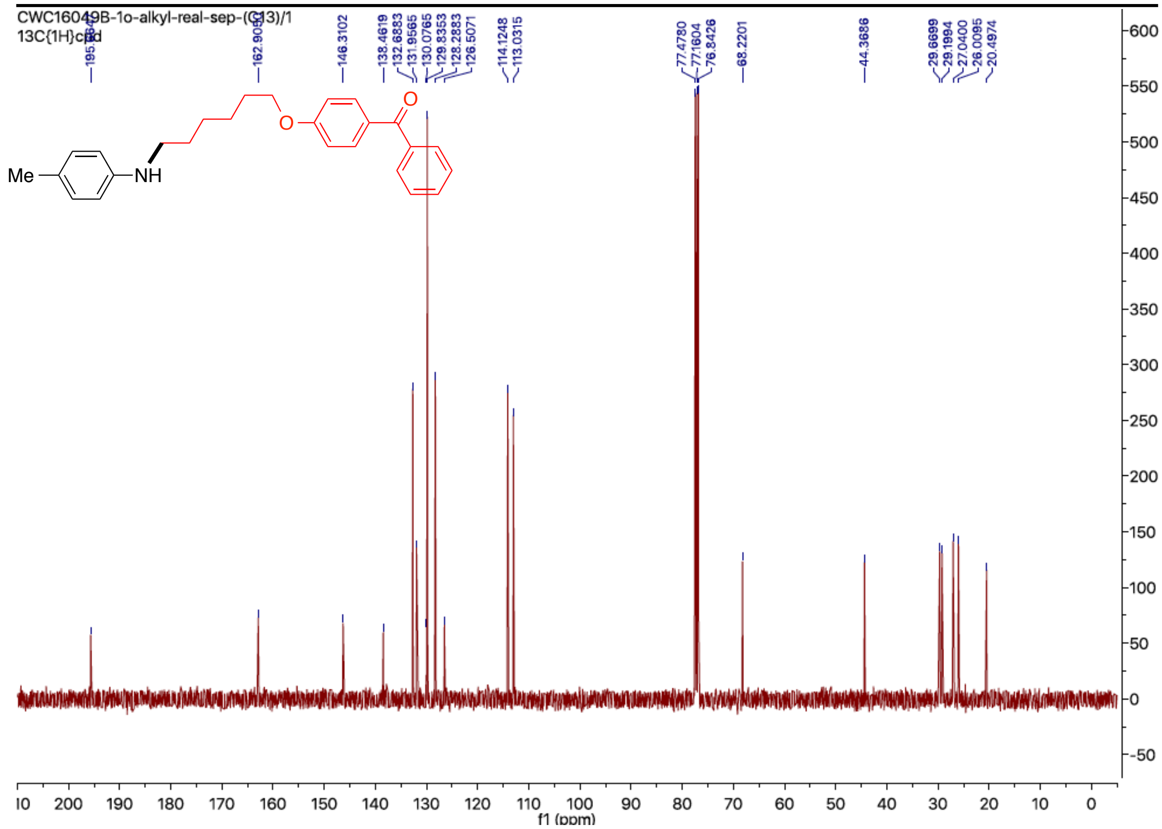

Supplementary Figure 82. ^{1}H and ^{13}C NMR spectra of Phenyl(4-((6-(*p*-tolylamino)hexyl)oxy)phenyl)methanone (6f)

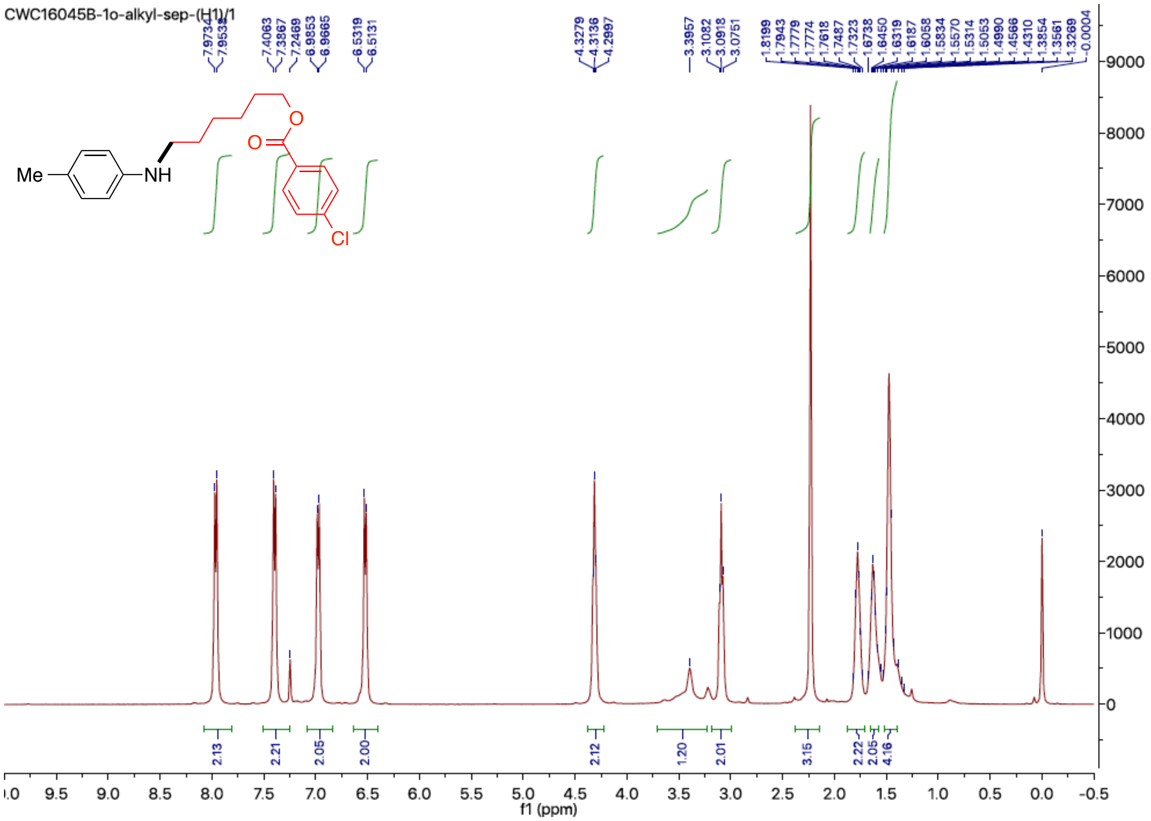

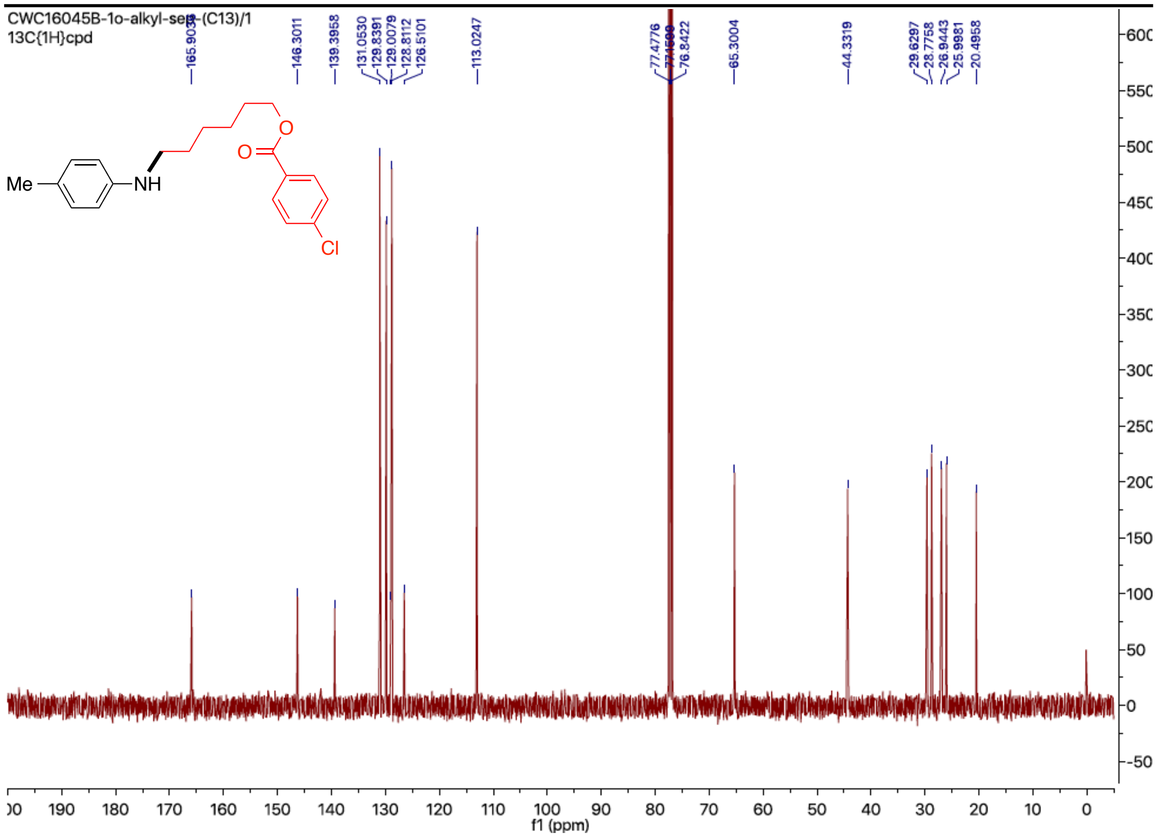

Supplementary Figure 83. ^{1}H and ^{13}C NMR spectra of 6-(*p*-Tolylamino)hexyl 4-Chlorobenzoate (6g)

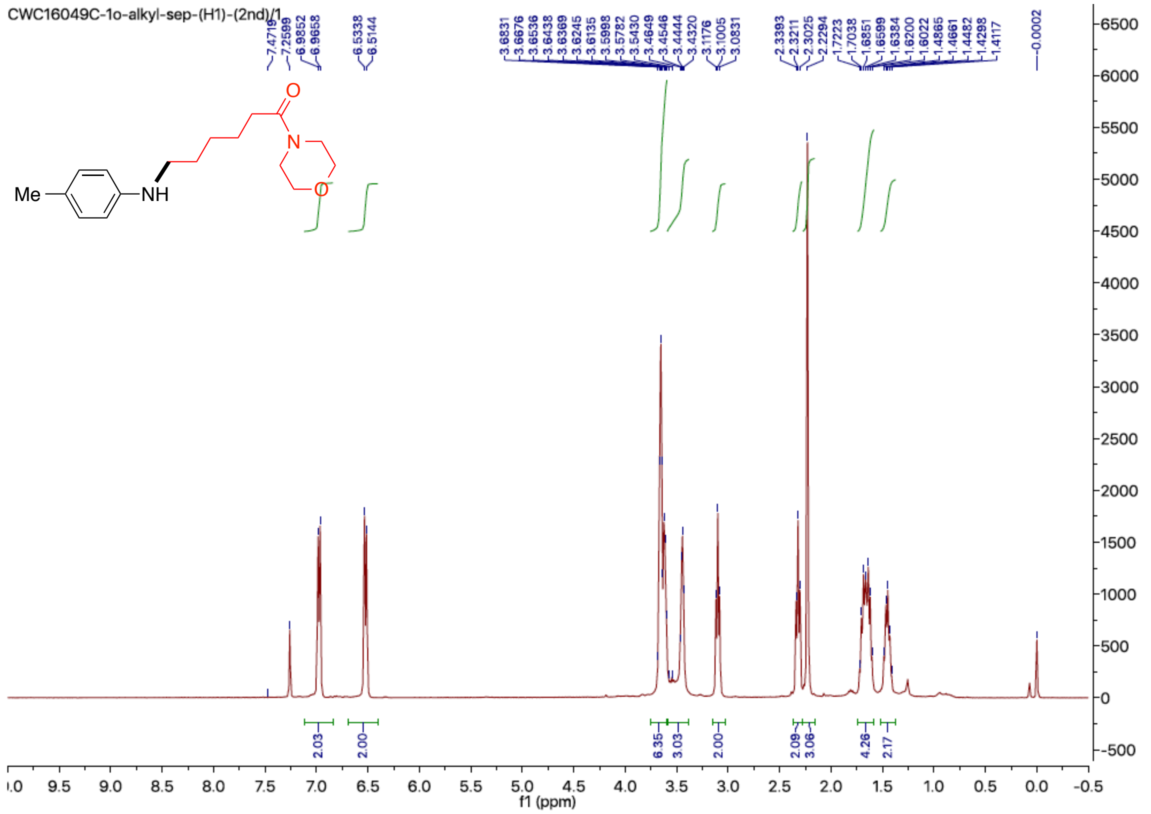

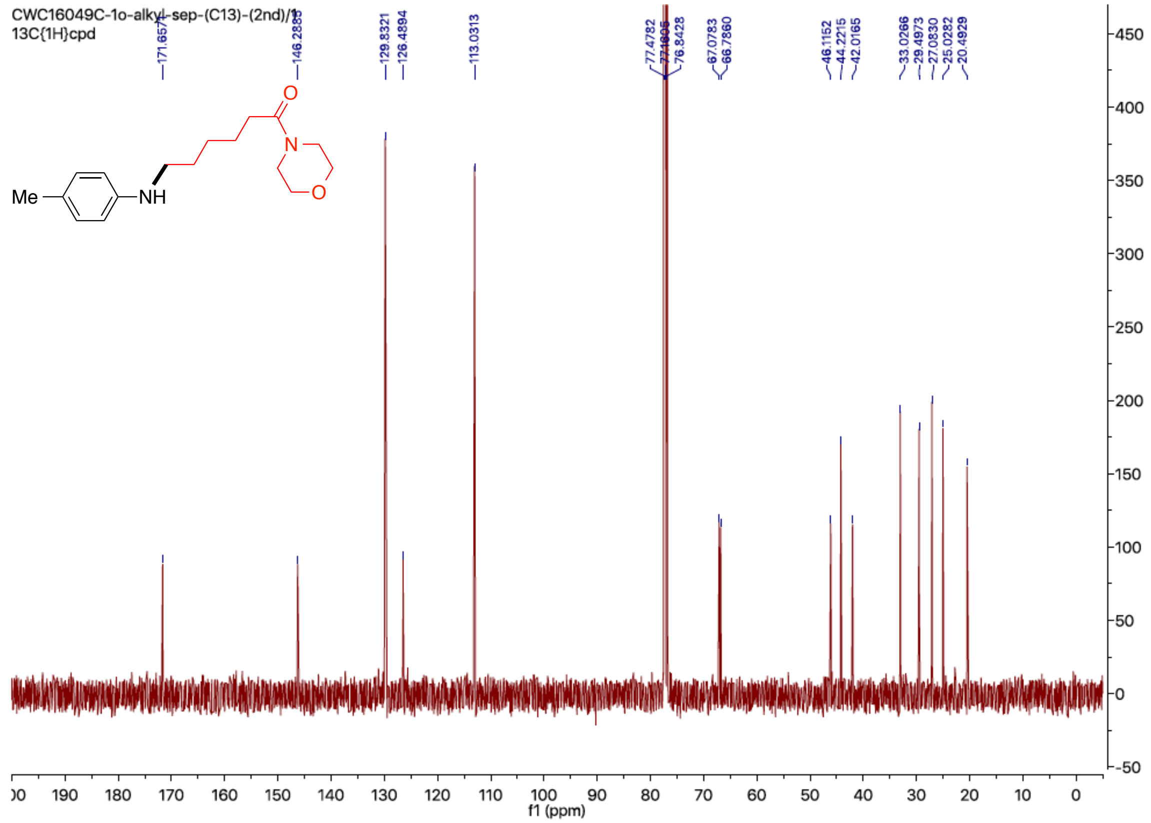

Supplementary Figure 84. ^{1}H and ^{13}C NMR spectra of 1-Morpholino-6-(*p*-tolylamino)hexan-1-one (6h)

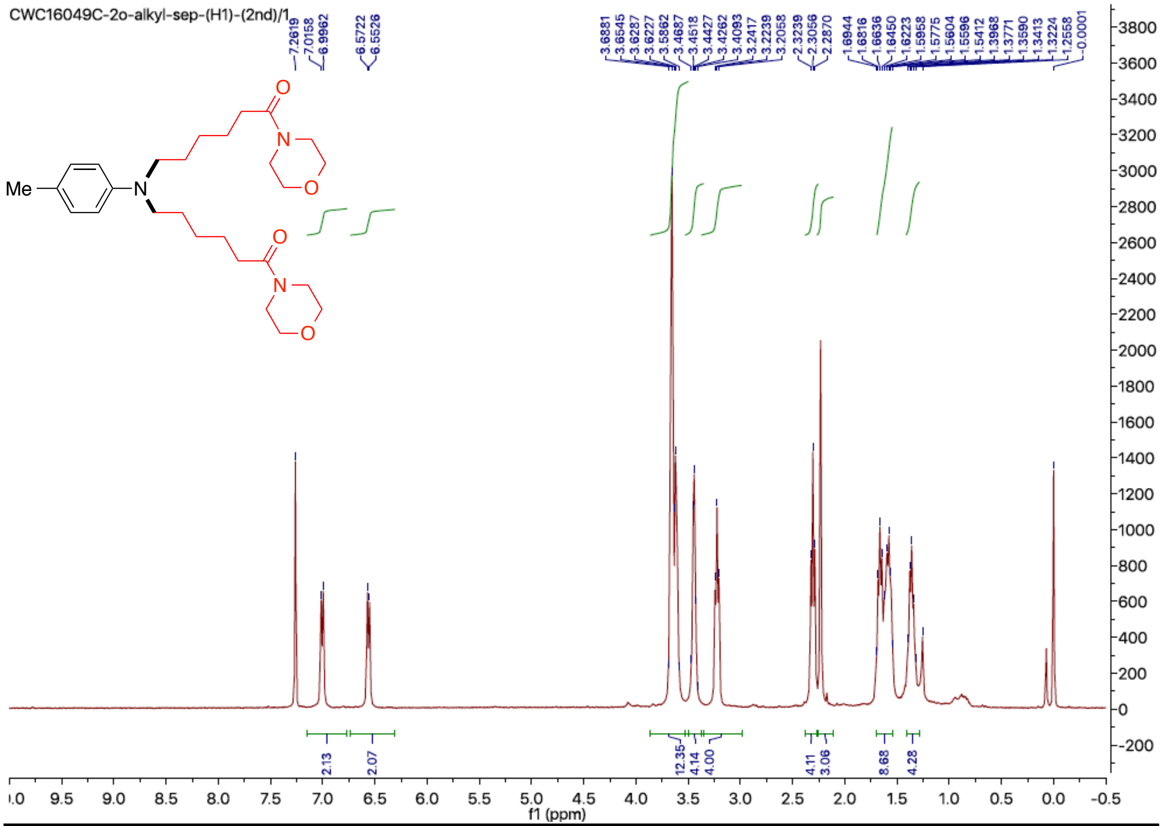

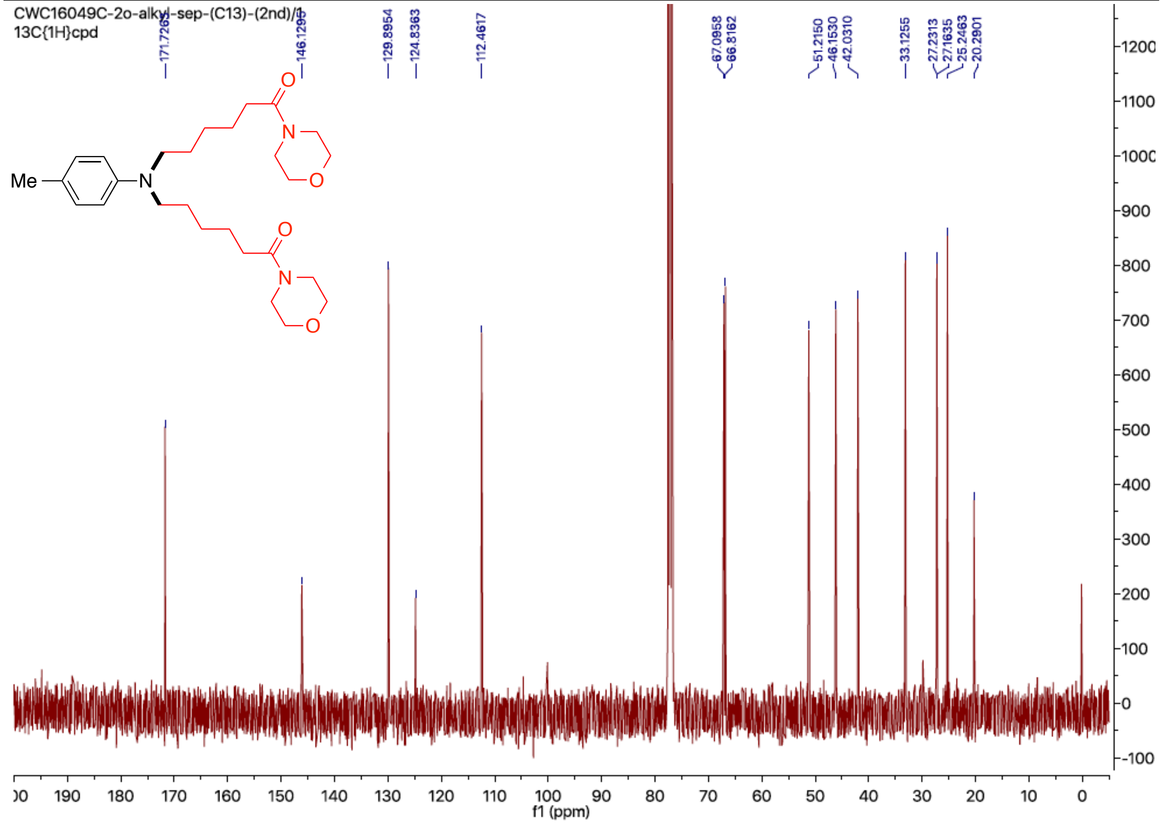

Supplementary Figure 85. ^{1}H and ^{13}C NMR spectra of 6,6'-(*p*-Tolylazanediyl)bis(1-morpholinohexan-1-one) (7h)

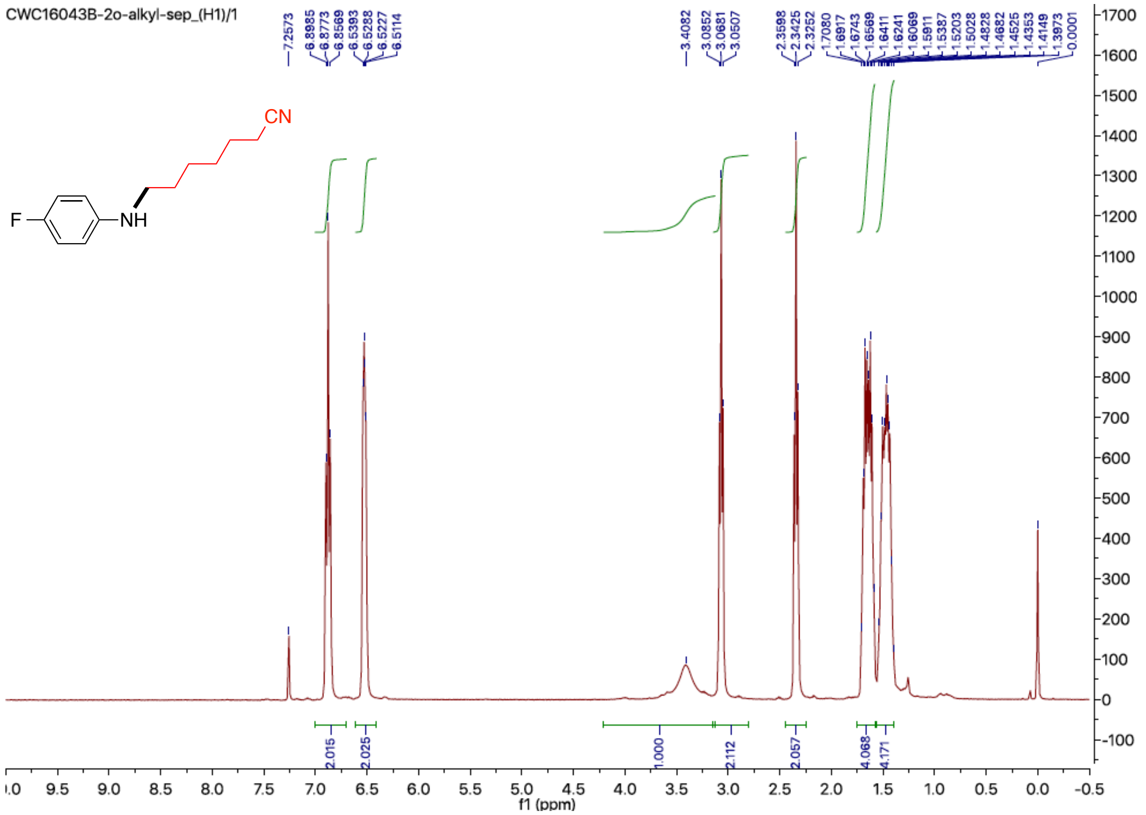

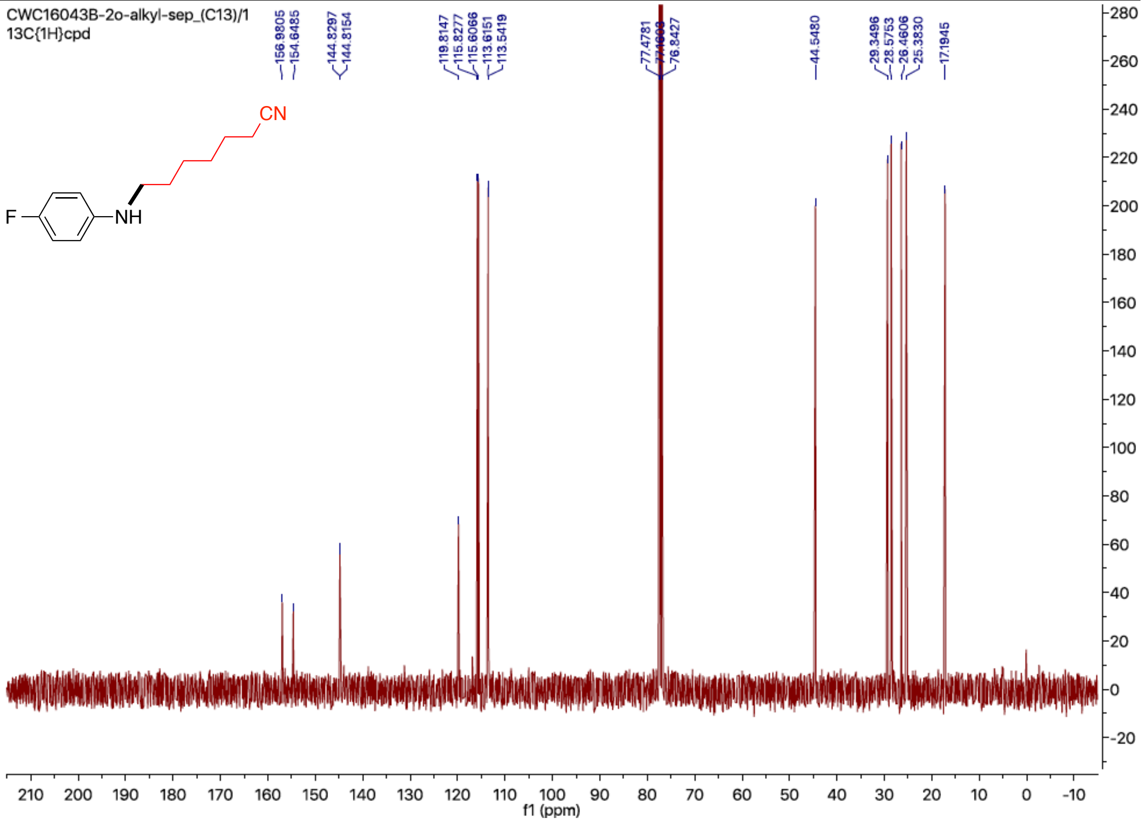

Supplementary Figure 86. ^{1}H and ^{13}C NMR spectra of 7-((4-Fluorophenyl)amino)heptanenitrile (6i)

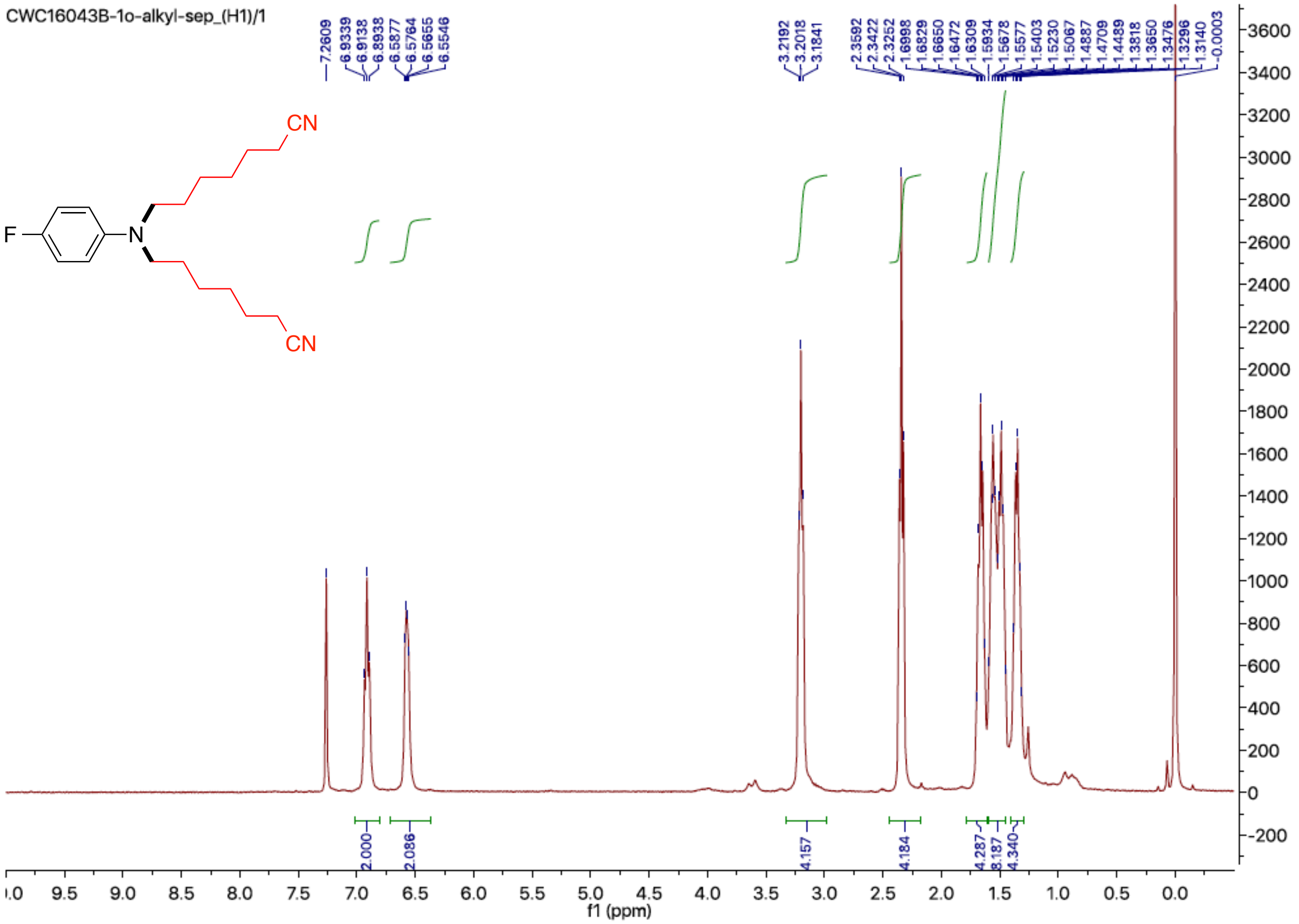

Supplementary Figure 87. ^{1}**H NMR spectrum of 7,7'-((4-Fluorophenyl)azanediyl)diheptanenitrile (7i)**

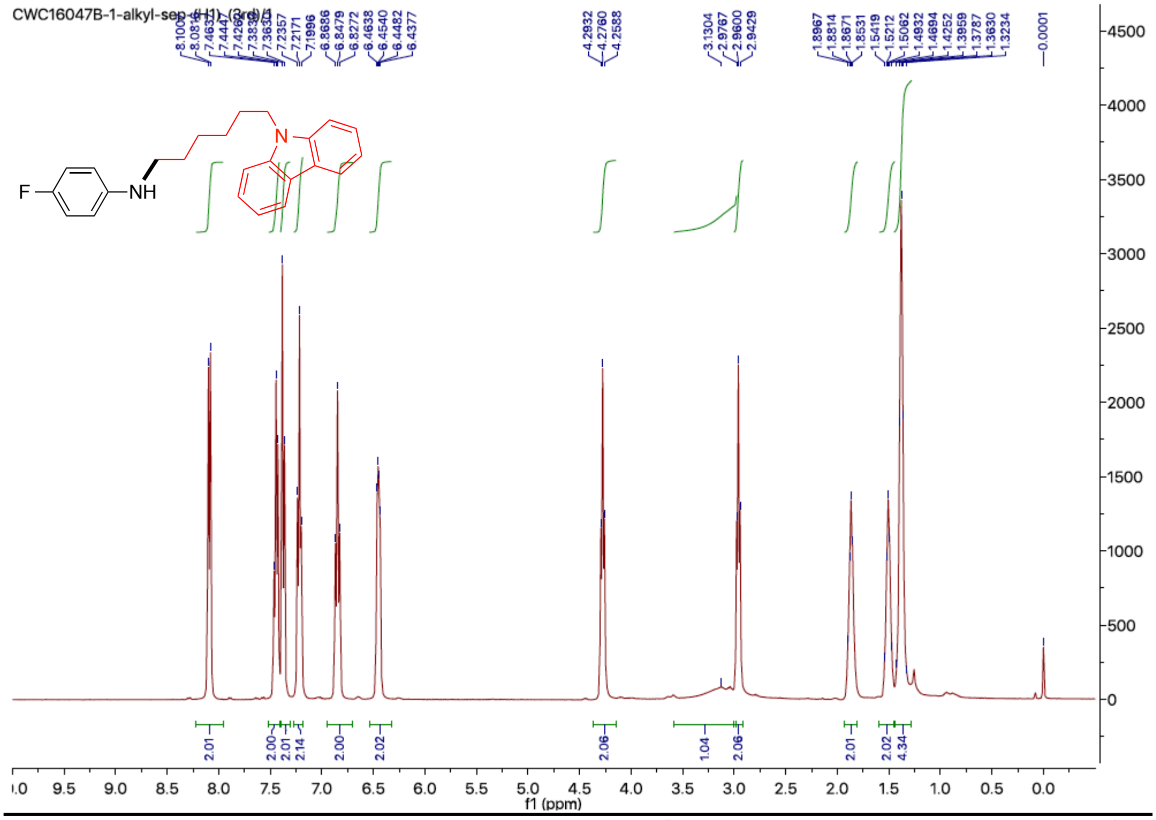

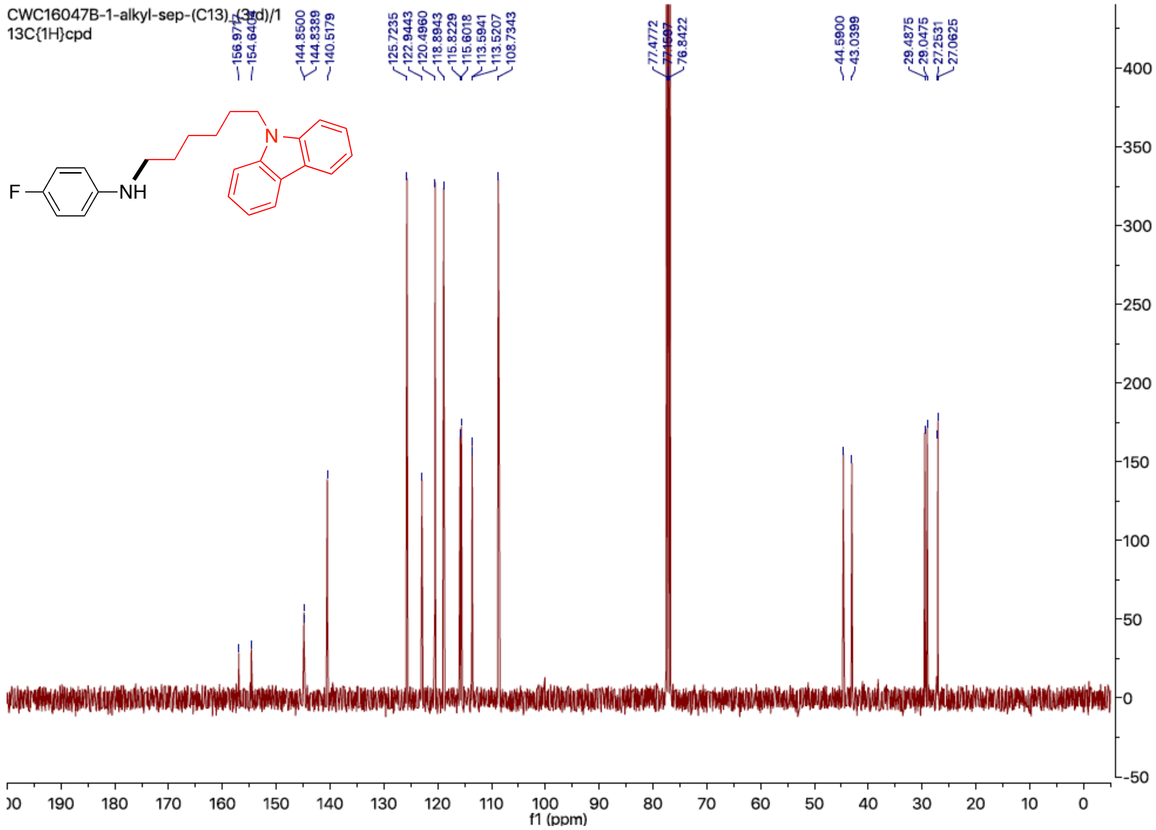

Supplementary Figure 88. ^{1}**H and** ^{13}**C NMR spectra of** ***N*-(6-(9*H*-Carbazol-9-yl)hexyl)-4-fluoroaniline (6j)**

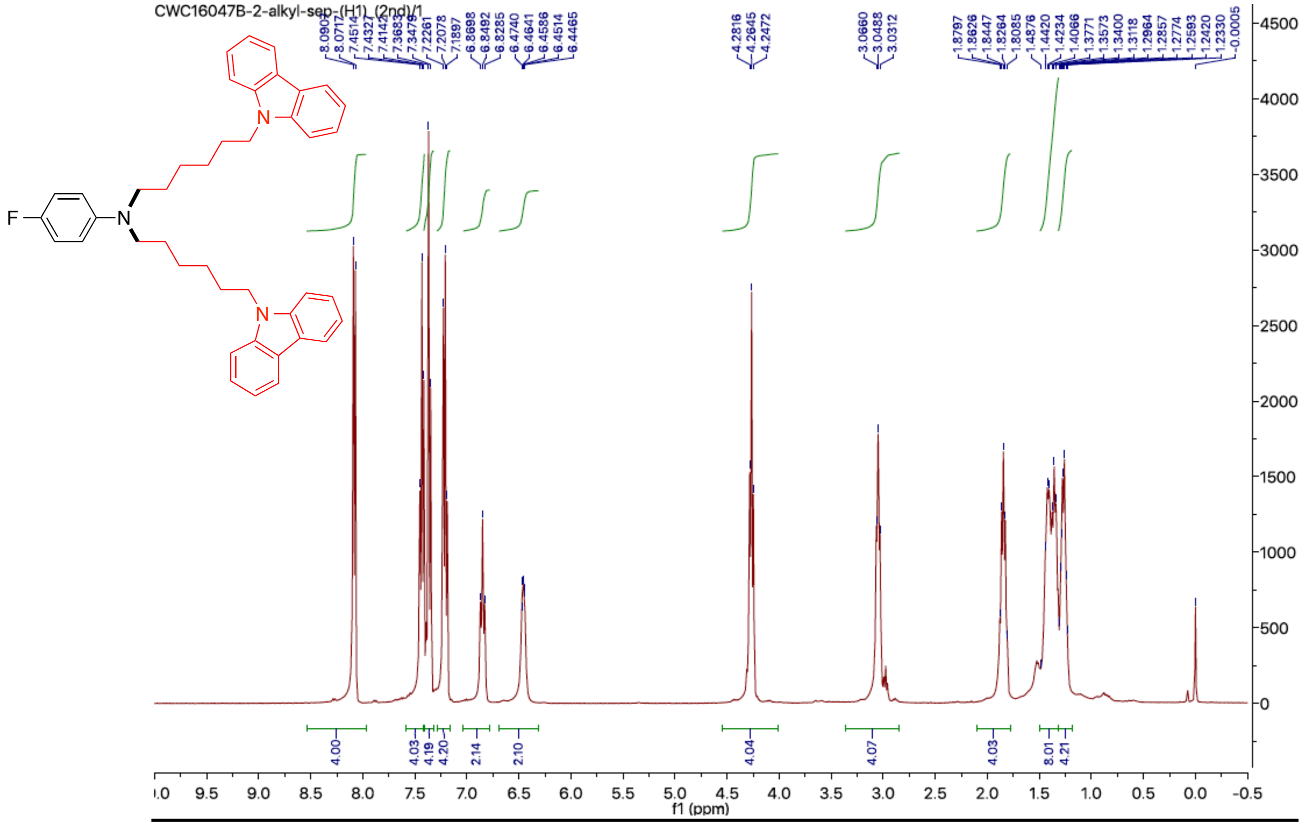

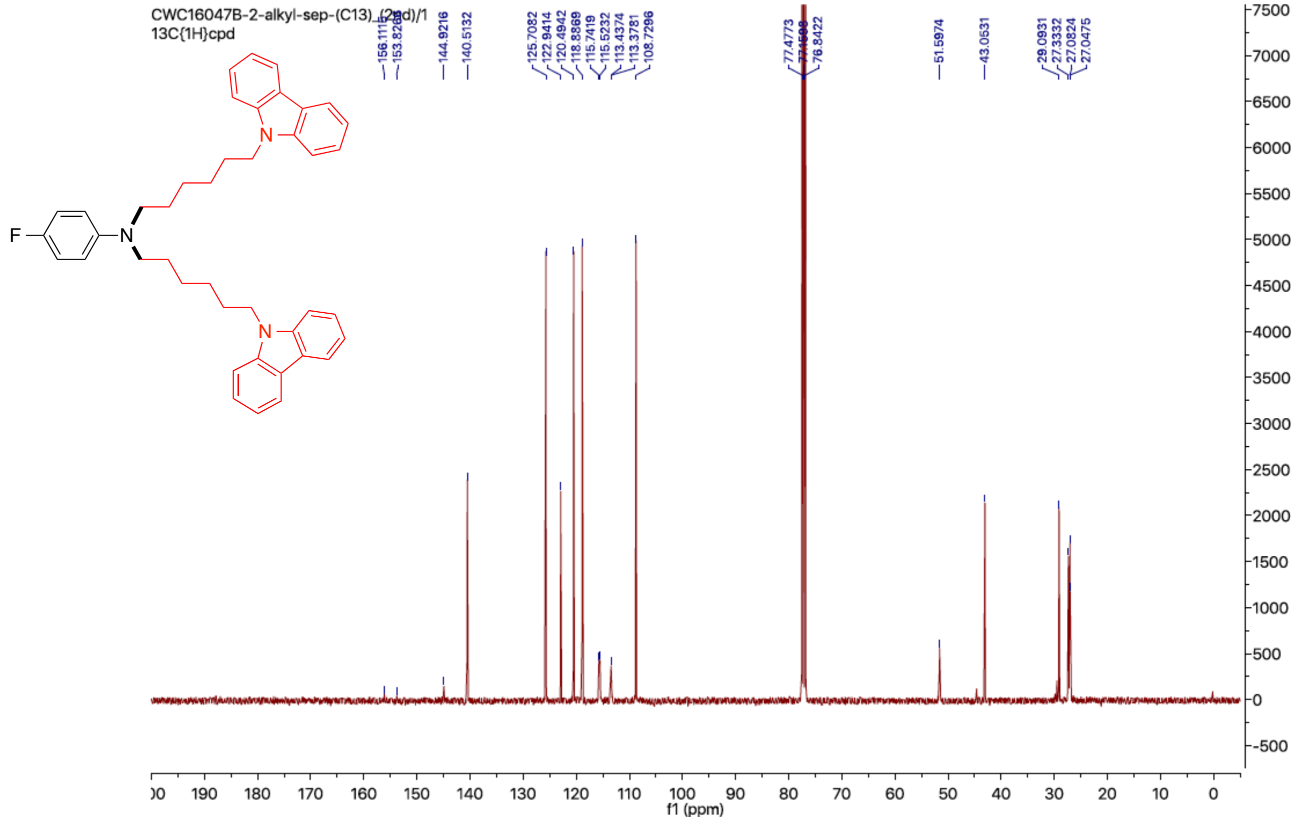

Supplementary Figure 89. ^{1}H and ^{13}C NMR spectra of *N*,*N*-bis(6-(9*H*-carbazol-9-yl)hexyl)-4-fluoroaniline (7j)

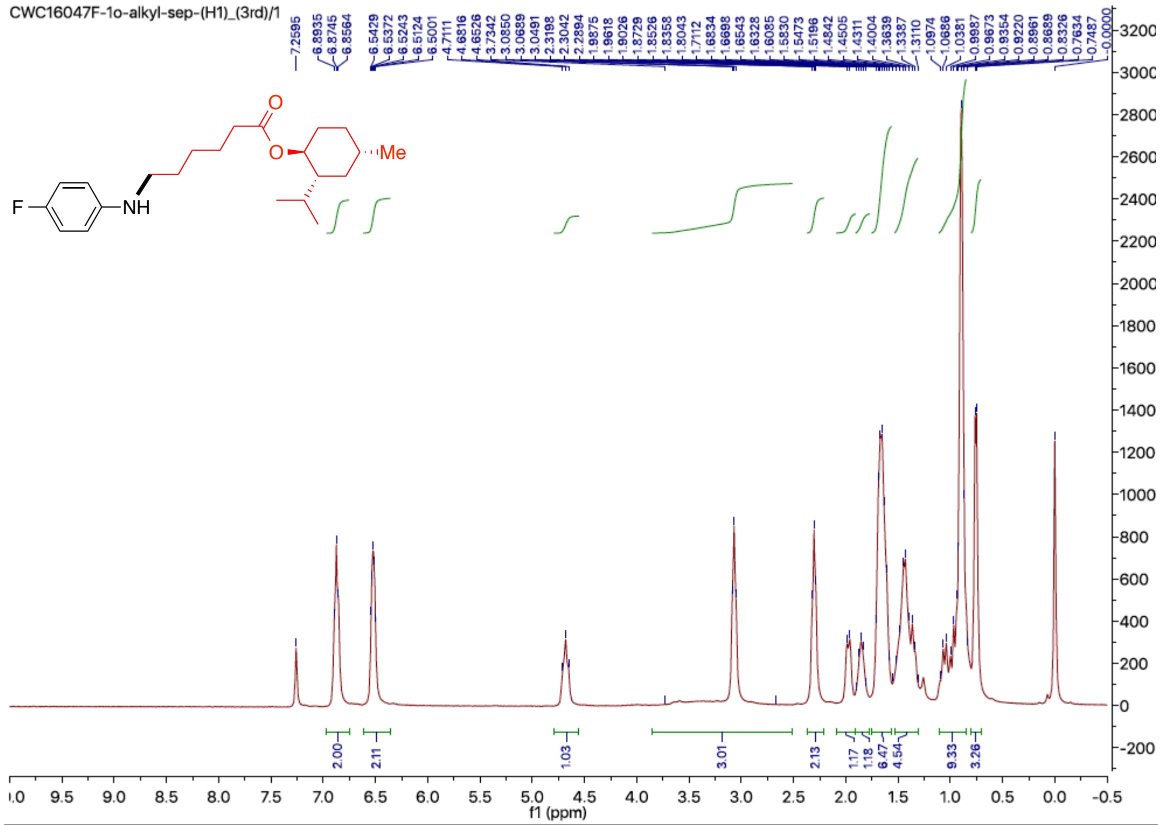

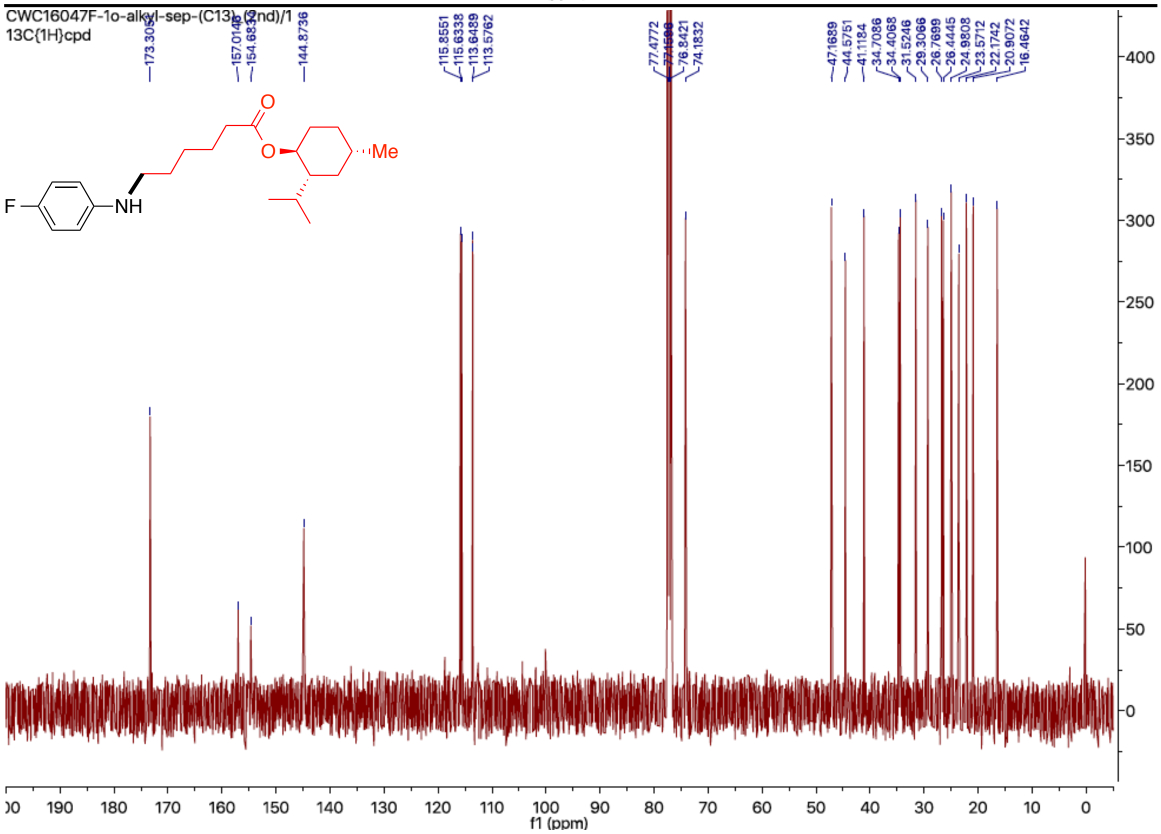

Supplementary Figure 90. ^{1}H and ^{13}C NMR spectra of (+/-)-2-Isopropyl-4-methylcyclohexyl 6-((4-Fluorophenyl)amino)hexanoate (6k)

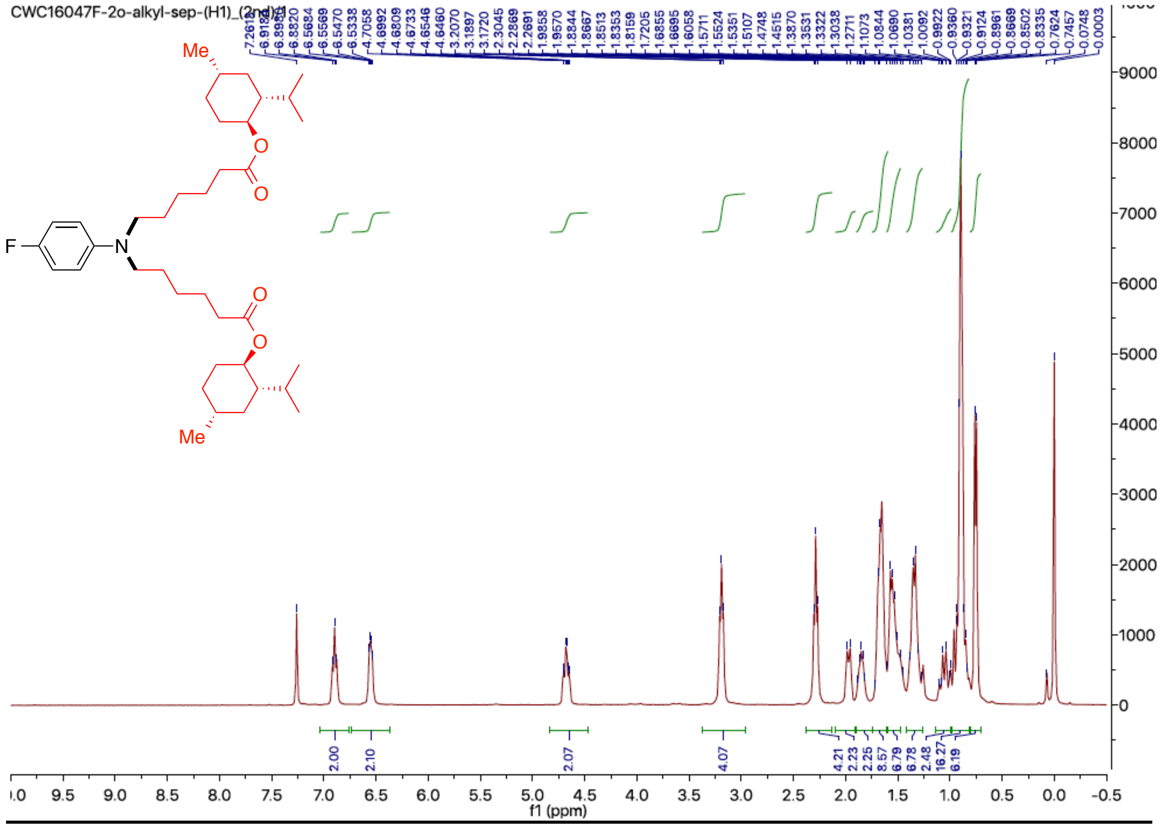

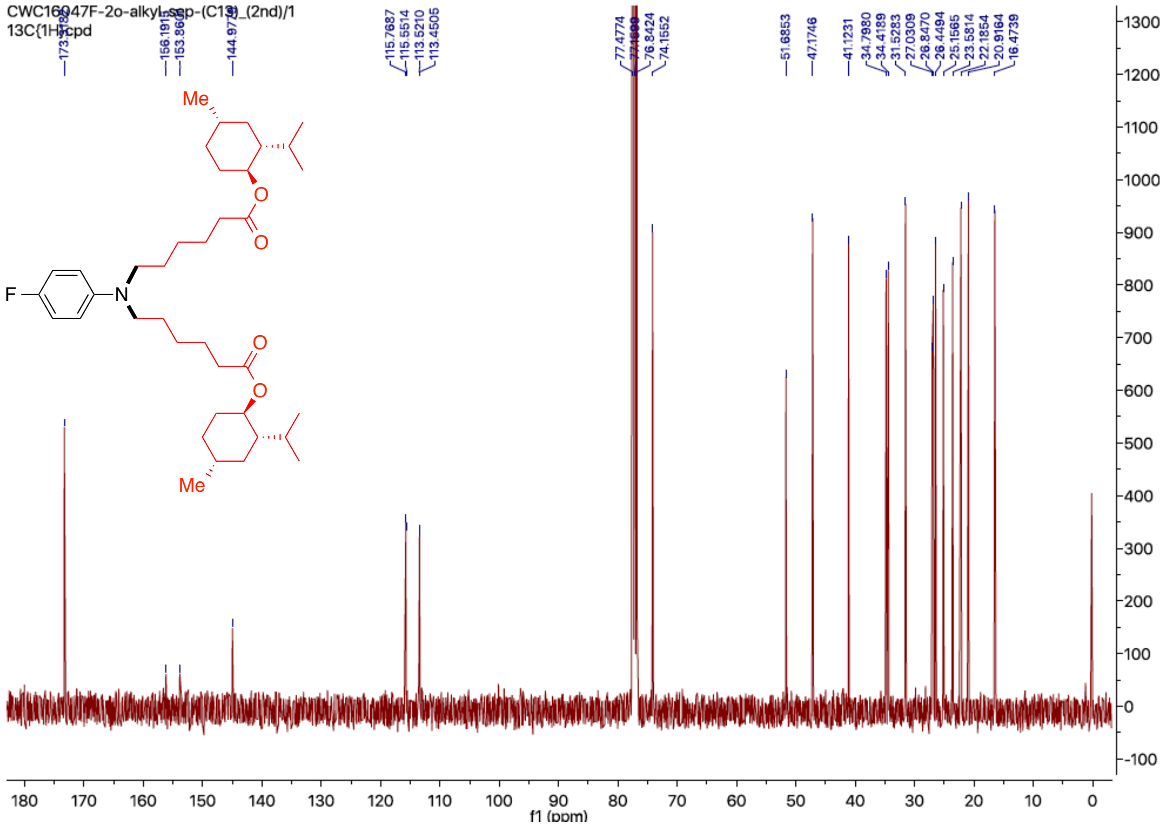

Supplementary Figure 91. ^{1}**H and** ^{13}**C NMR spectra of (+/-)-2-Isopropyl-4-methylcyclohexyl 6-((4-Fluorophenyl)(6-((2-isopropyl-4-methylcyclohexyl)oxy)-6-oxohexyl)amino)hexanoate (7k)**

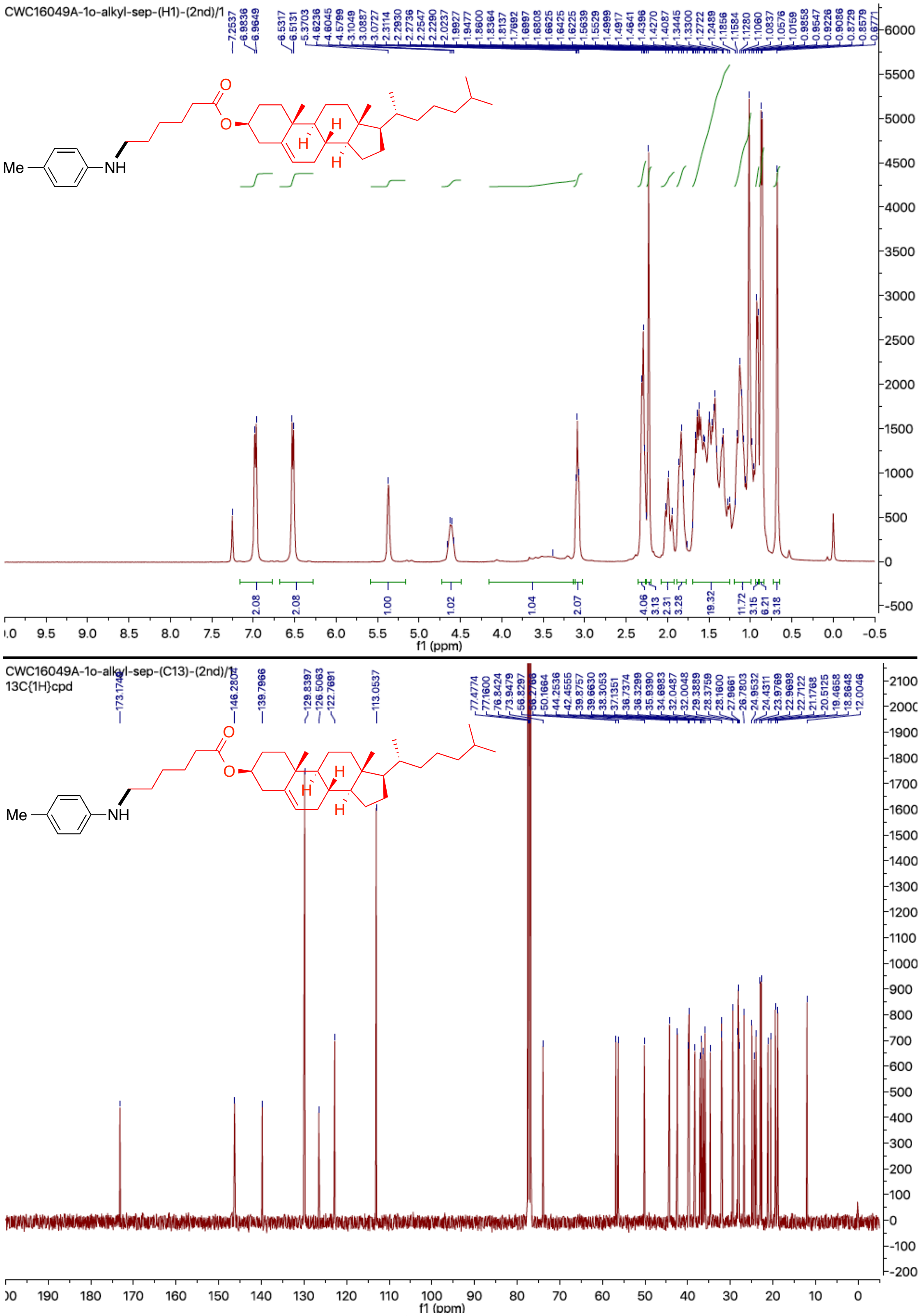

Supplementary Figure 92. **^{1}H and ^{13}C NMR spectra of (3*S*,8*S*,9*S*,10*R*,13*R*,14*S*,17*R*)-10,13-Dimethyl-17-((*R*)-6-methylheptan-2-yl)-2,3,4,7,8,9,10,11,12,13,14,15,16,17-tetradecahydro-1*H*-cyclopenta[*a*]phenanthren-3-yl 6-(*p*-Tolylamino)hexanoate (6l)**

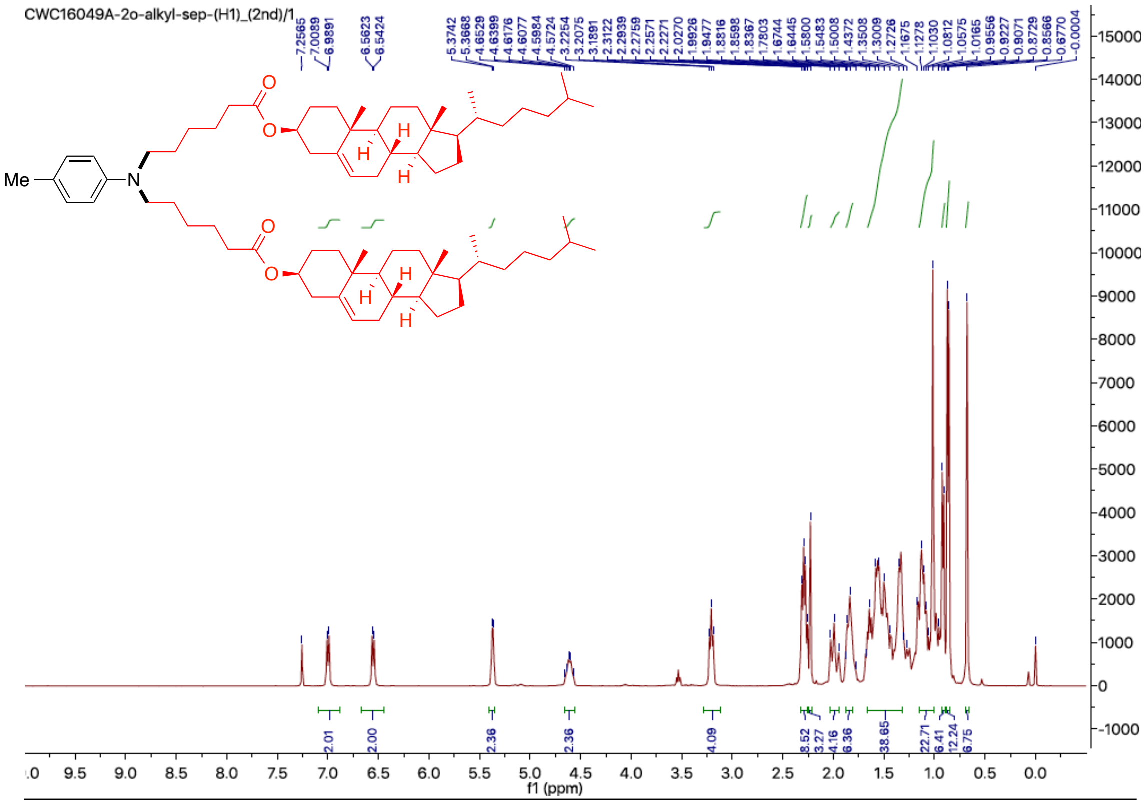

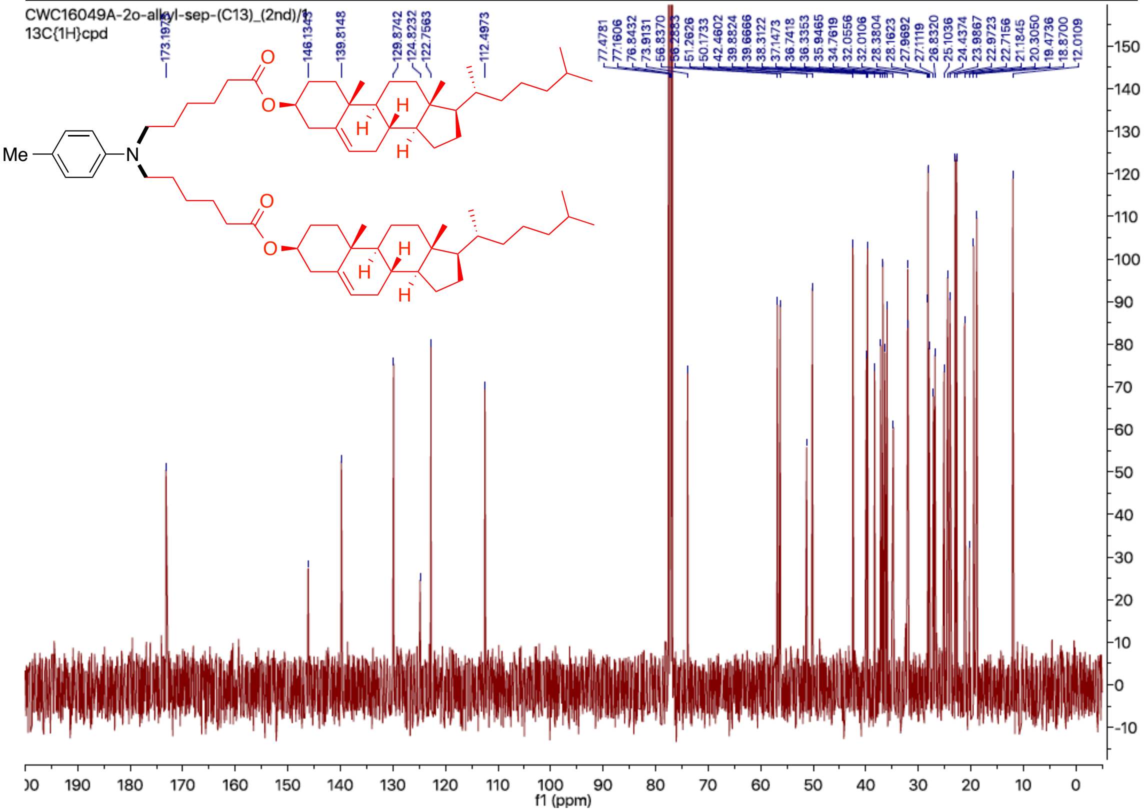

Supplementary Figure 93. ^{1}**H and** ^{13}**C NMR spectra of Bis((3*S*,8*S*,9*S*,10*R*,13*R*,14*S*,17*R*)-10,13-dimethyl-17-((*R*)-6-methylheptan-2-yl)-2,3,4,7,8,9,10,11,12,13,14,15,16,17-tetradecahydro-1*H*-cyclopenta[*a*]phenanthren-3-yl) 6,6'-(*p*-Tolylazanediyl)dihexanoate (7l)**

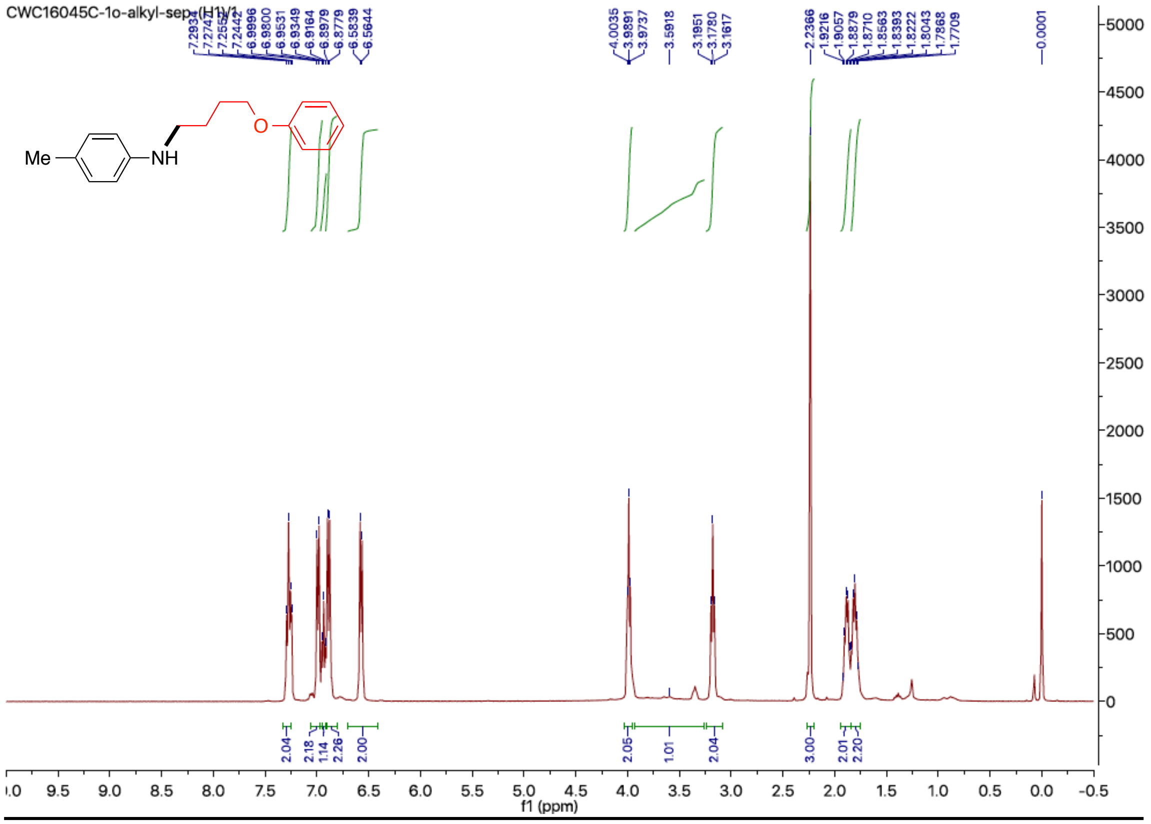

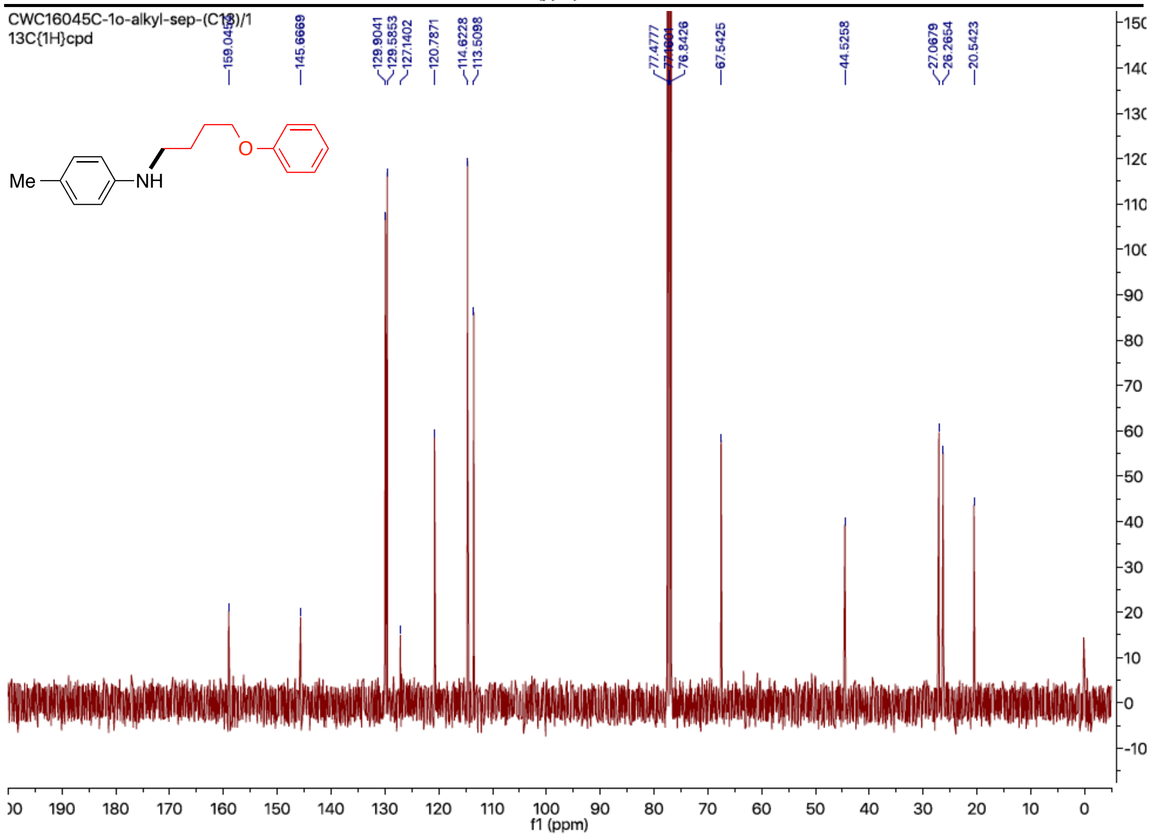

Supplementary Figure 94. ^{1}H and ^{13}C NMR spectra of Supplementary Figure 2. ^{1}H and ^{13}C NMR spectra of 4-Methyl-*N*-(4-phenoxybutyl)aniline (6m)

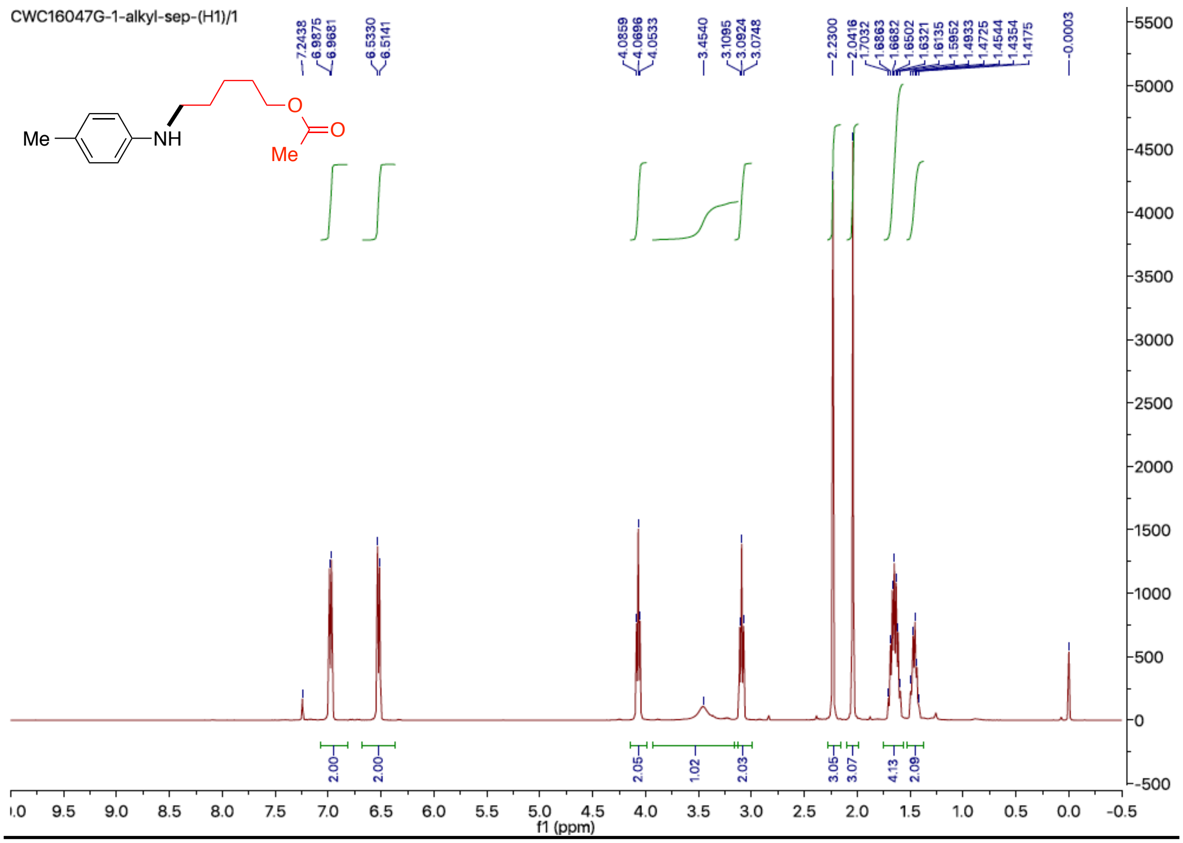

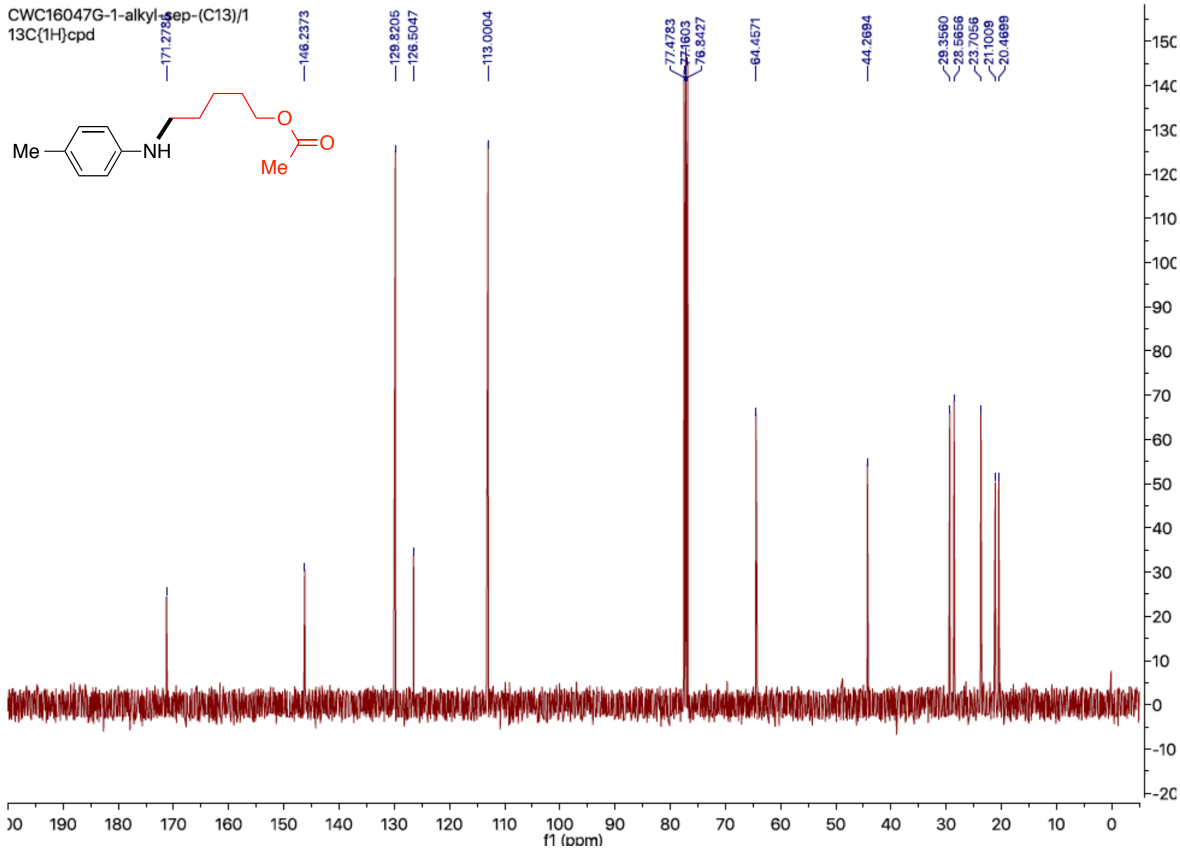

Supplementary Figure 95. ^{1}H and ^{13}C NMR spectra of 5-(*p*-Tolylamino)pentyl Acetate (6n)

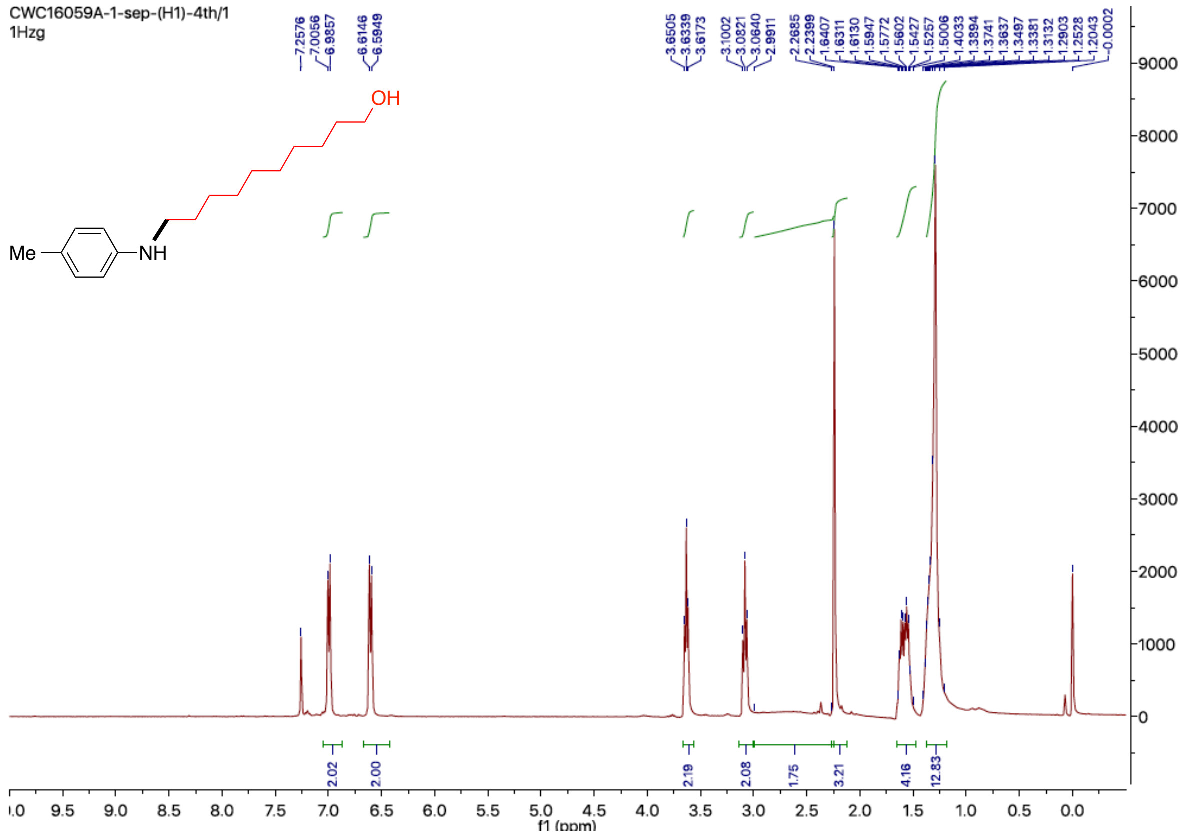

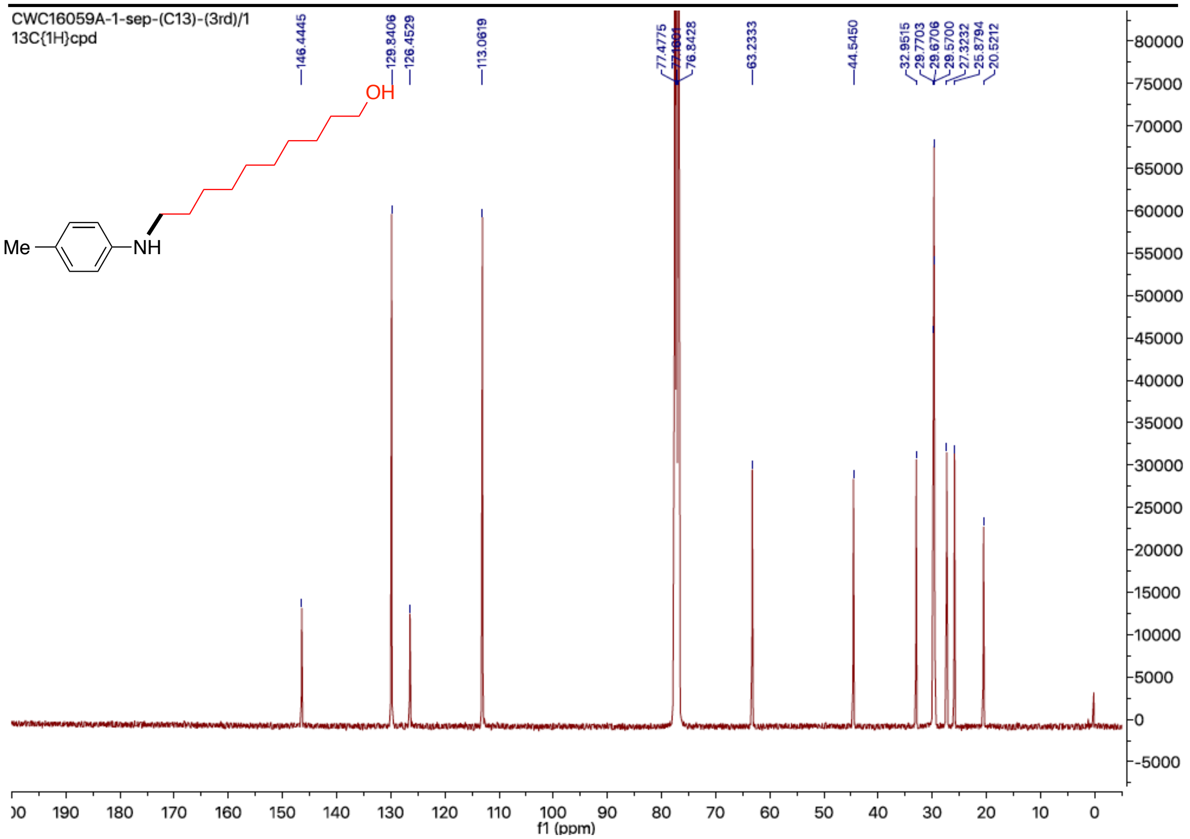

Supplementary Figure 96. ^{1}H and ^{13}C NMR spectra of 9-(*p*-Tolylamino)nonan-1-ol (6o)

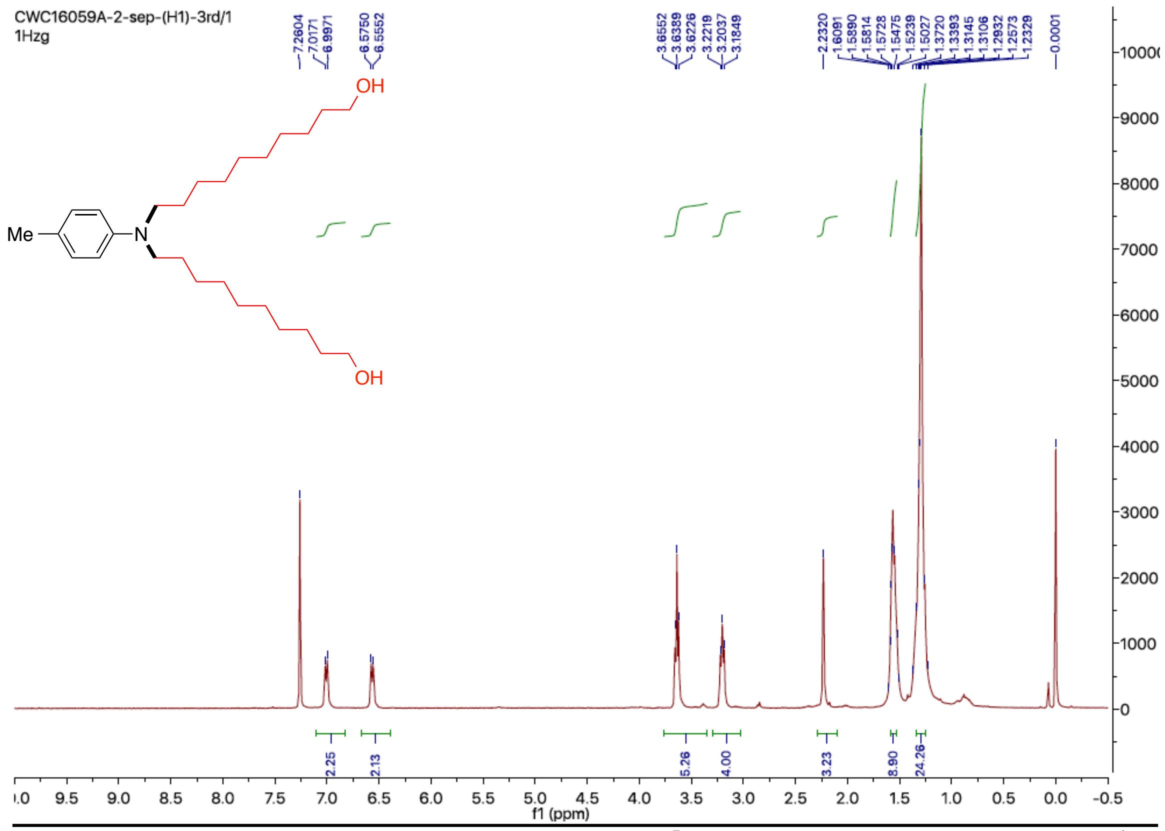

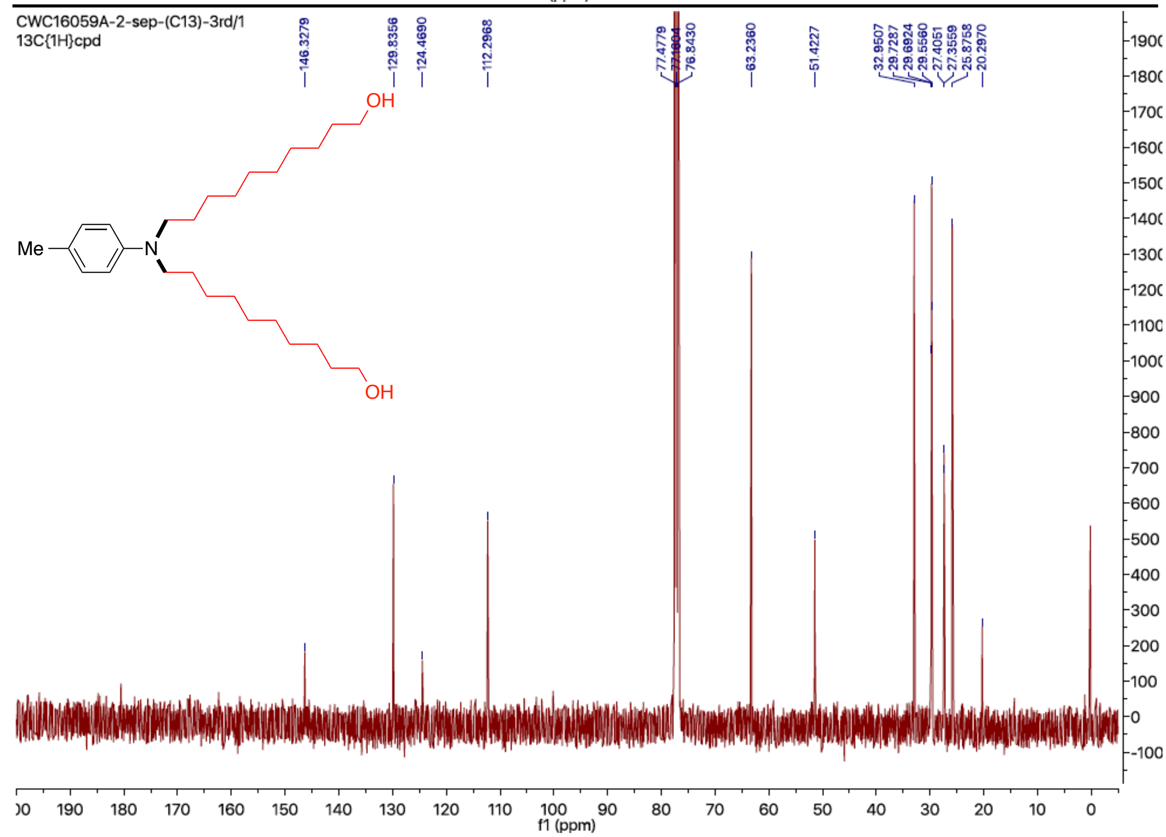

Supplementary Figure 97. ^{1}**H and** ^{13}**C NMR spectra of 10,10'-(*p*-Tolylazanediyl)bis(decan-1-ol) (7o)**

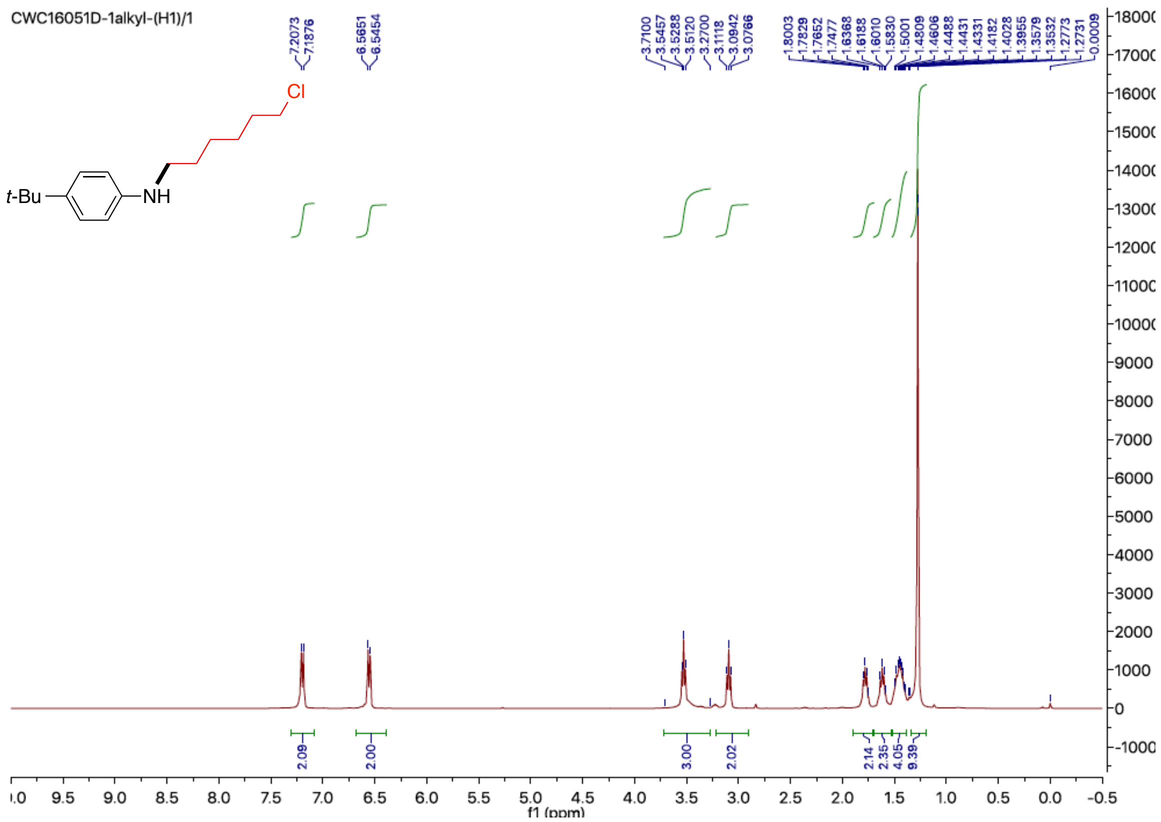

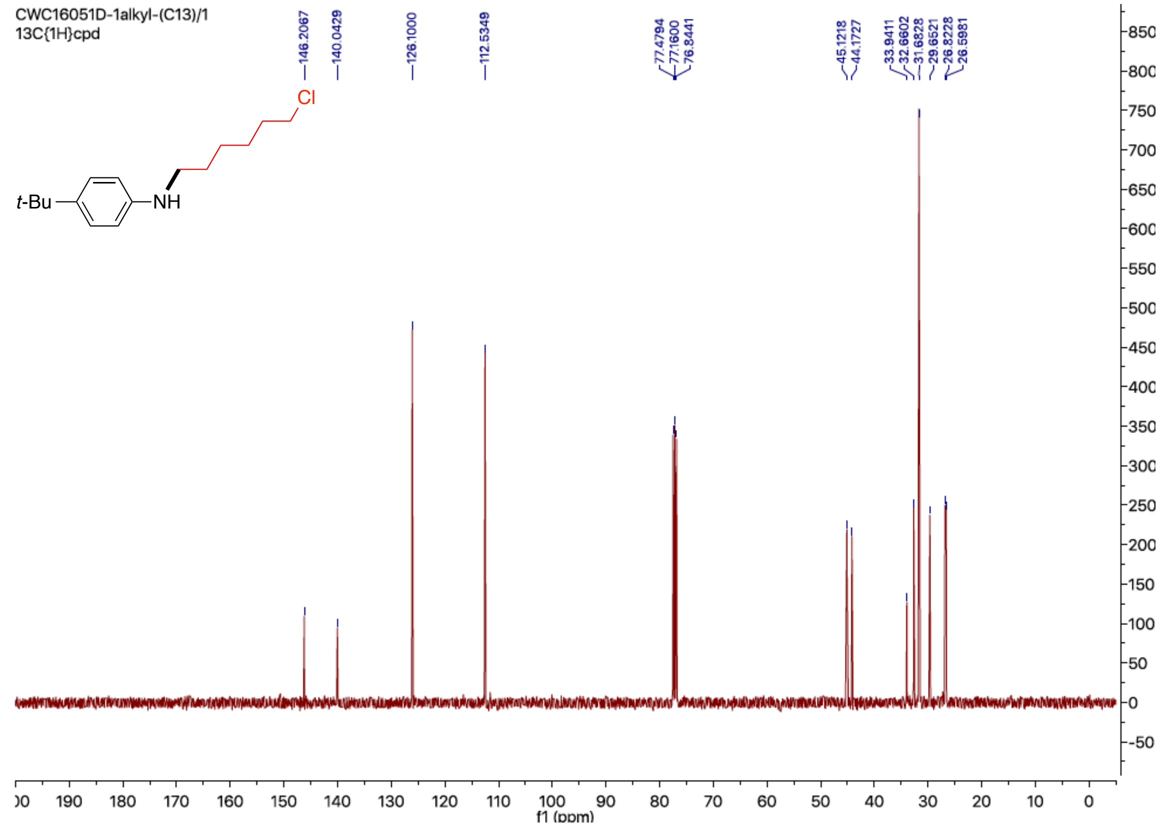

Supplementary Figure 98. ^{1}H and ^{13}C NMR spectra of 4-(*tert*-Butyl)-*N*-(6-chlorohexyl)aniline (6p)

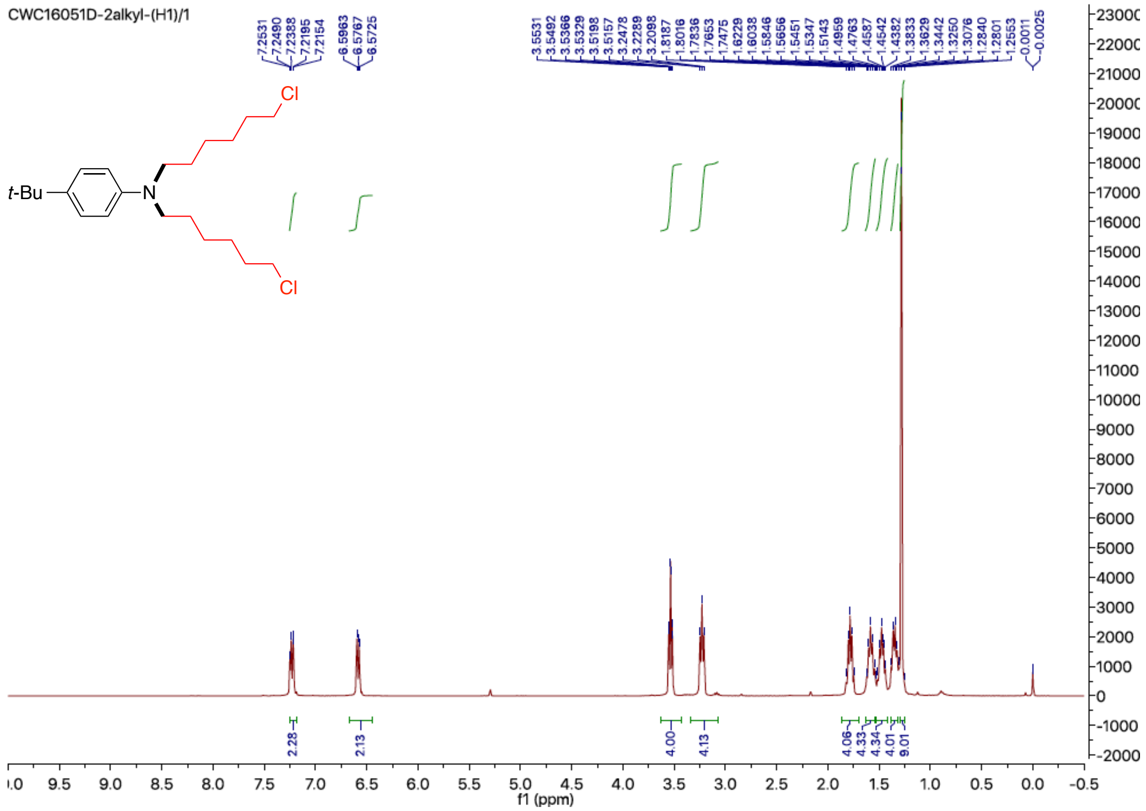

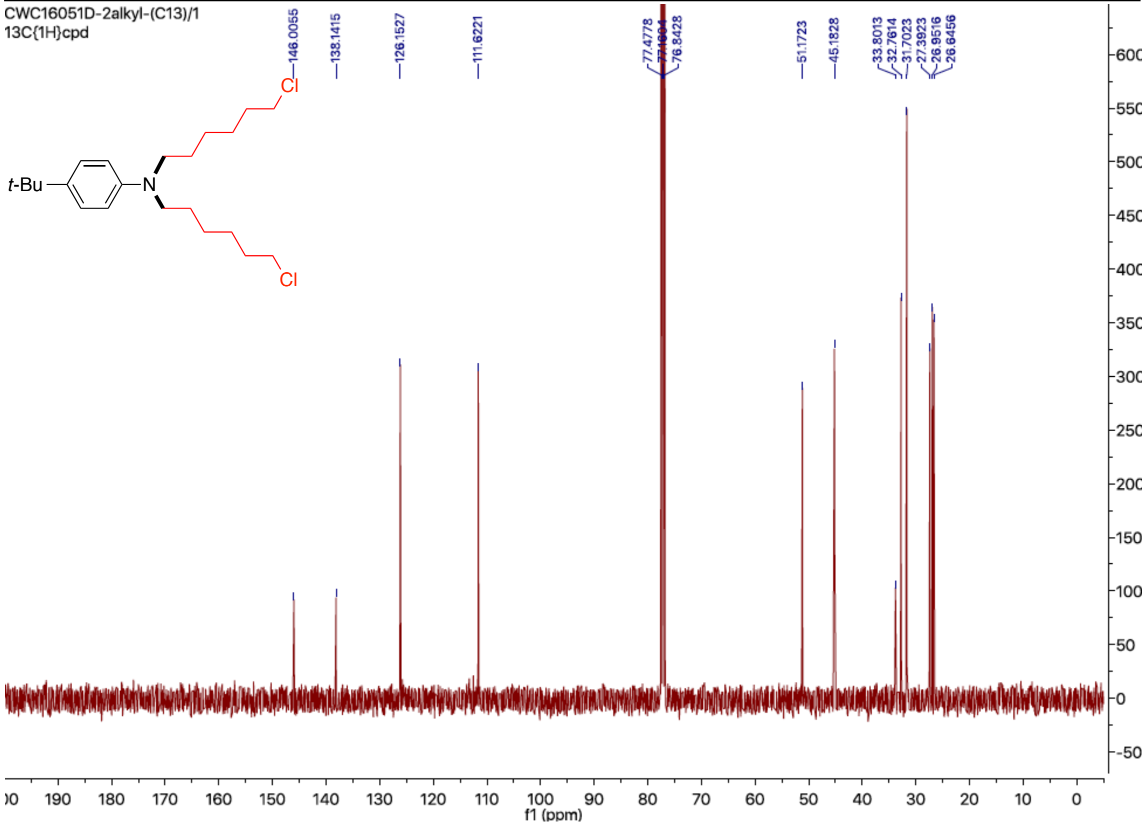

Supplementary Figure 99. ^{1}H and ^{13}C NMR spectra of 4-(*tert*-Butyl)-*N*,*N*-bis(6-chlorohexyl)aniline (7p)

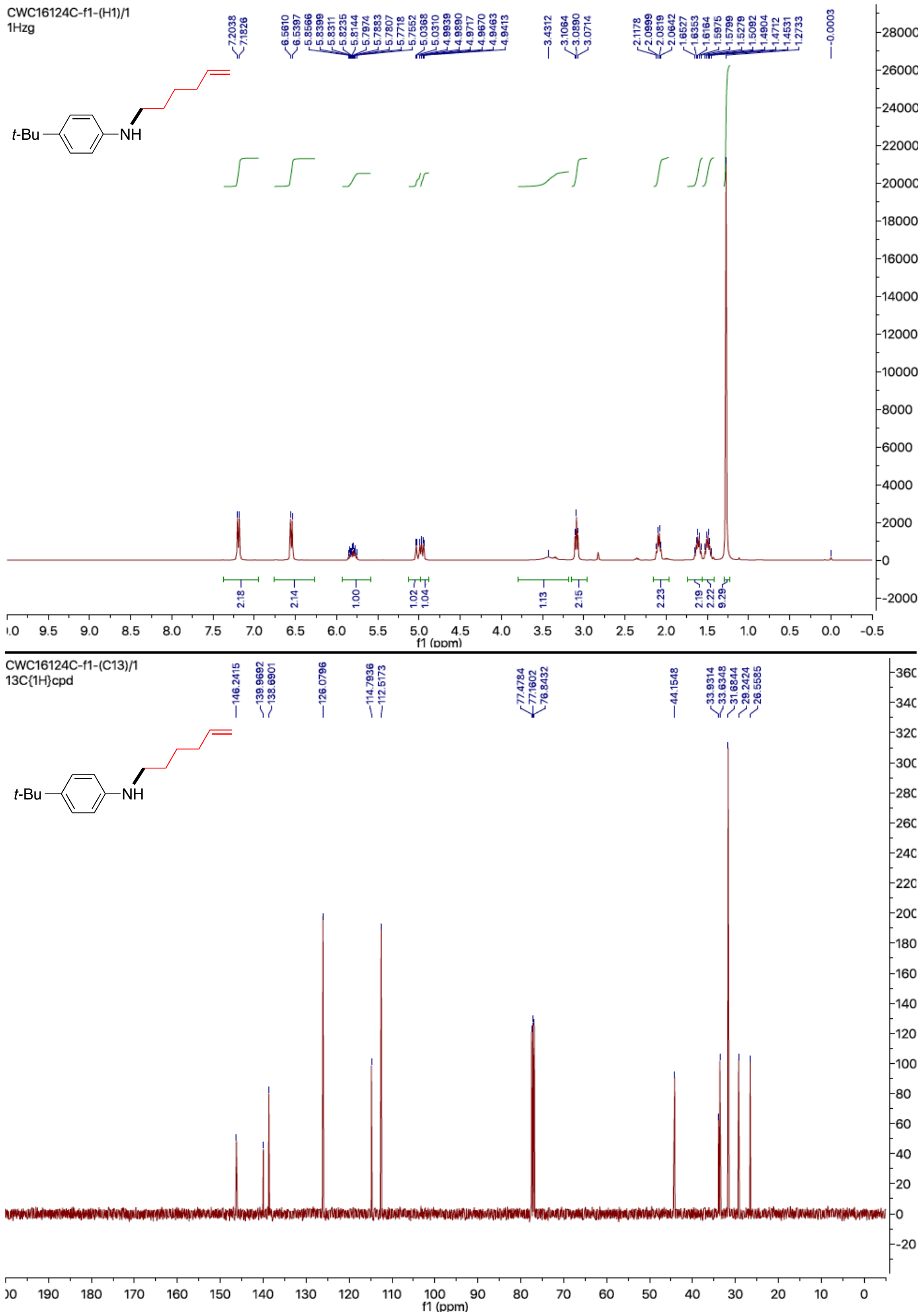

Supplementary Figure 100. ^{1}H and ^{13}C NMR spectra of 4-(*tert*-Butyl)-*N*-(hex-5-en-1-yl)aniline (6q)

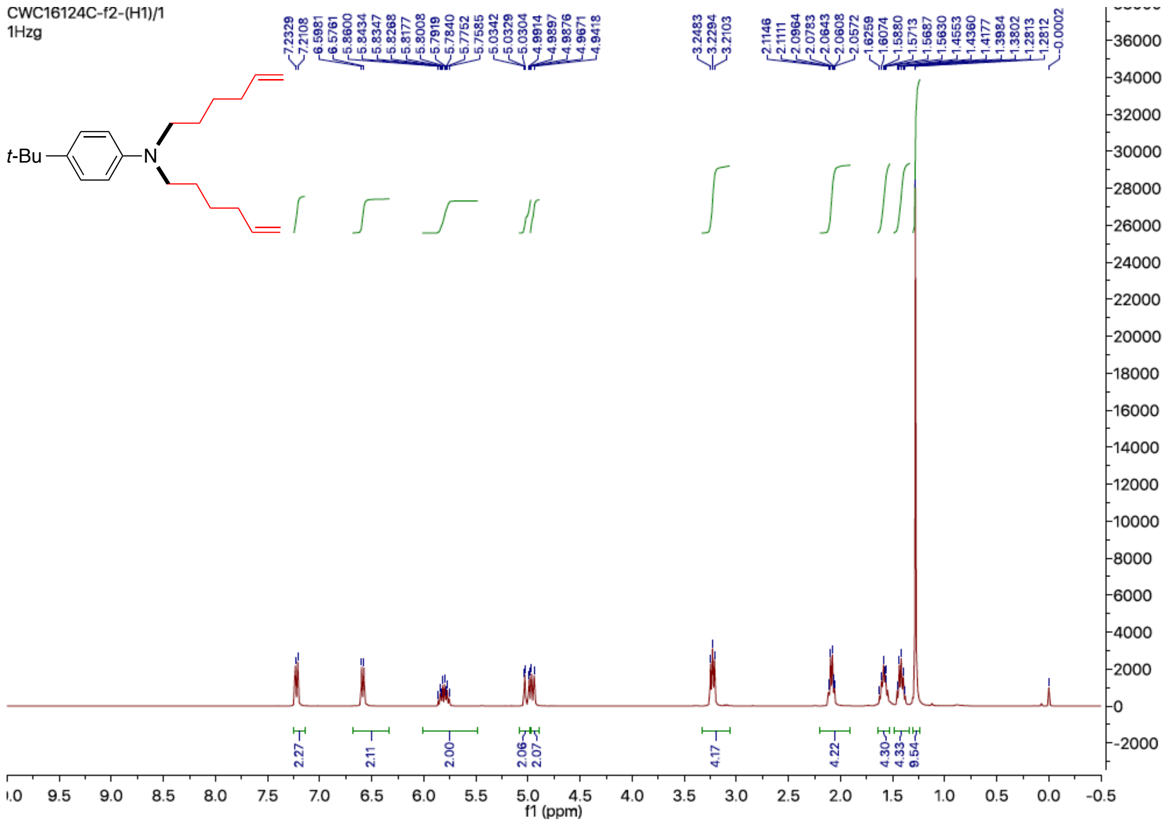

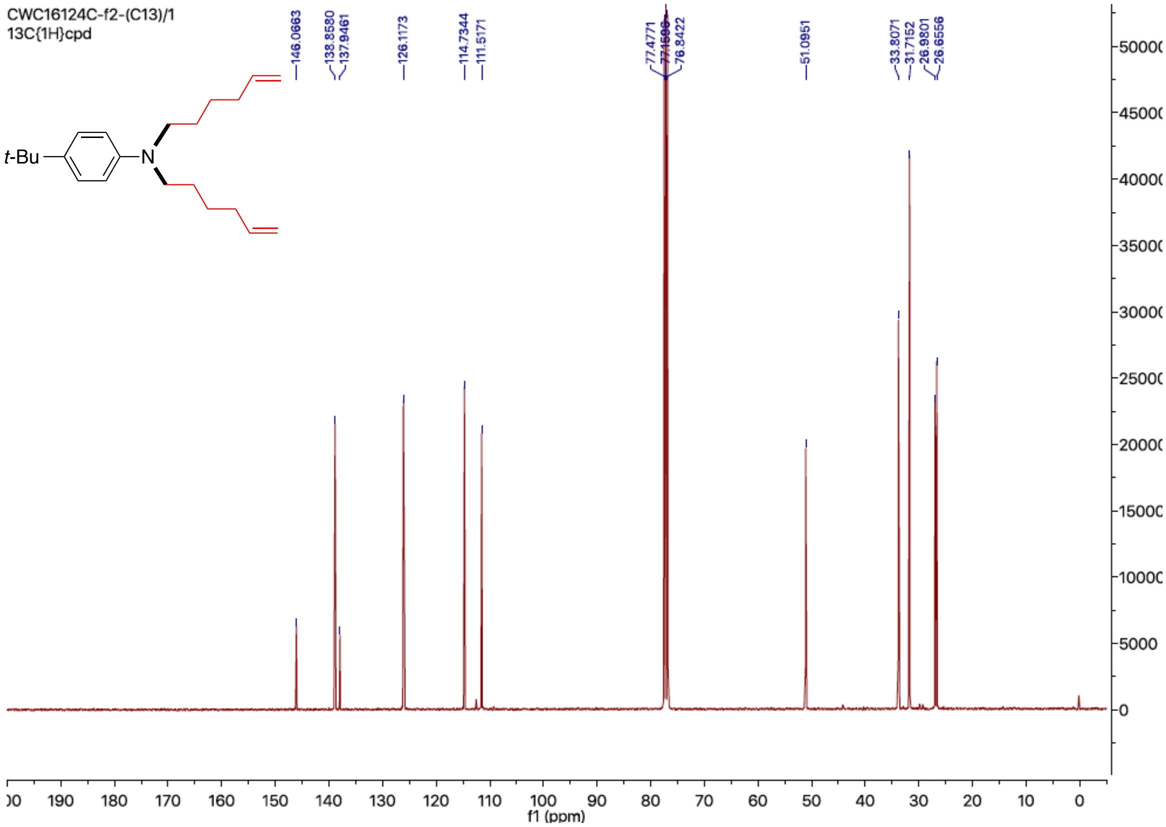

Supplementary Figure 101. ^{1}H and ^{13}C NMR spectra of 4-(*tert*-Butyl)-*N*,*N*-di(hex-5-en-1-yl)aniline (7q)

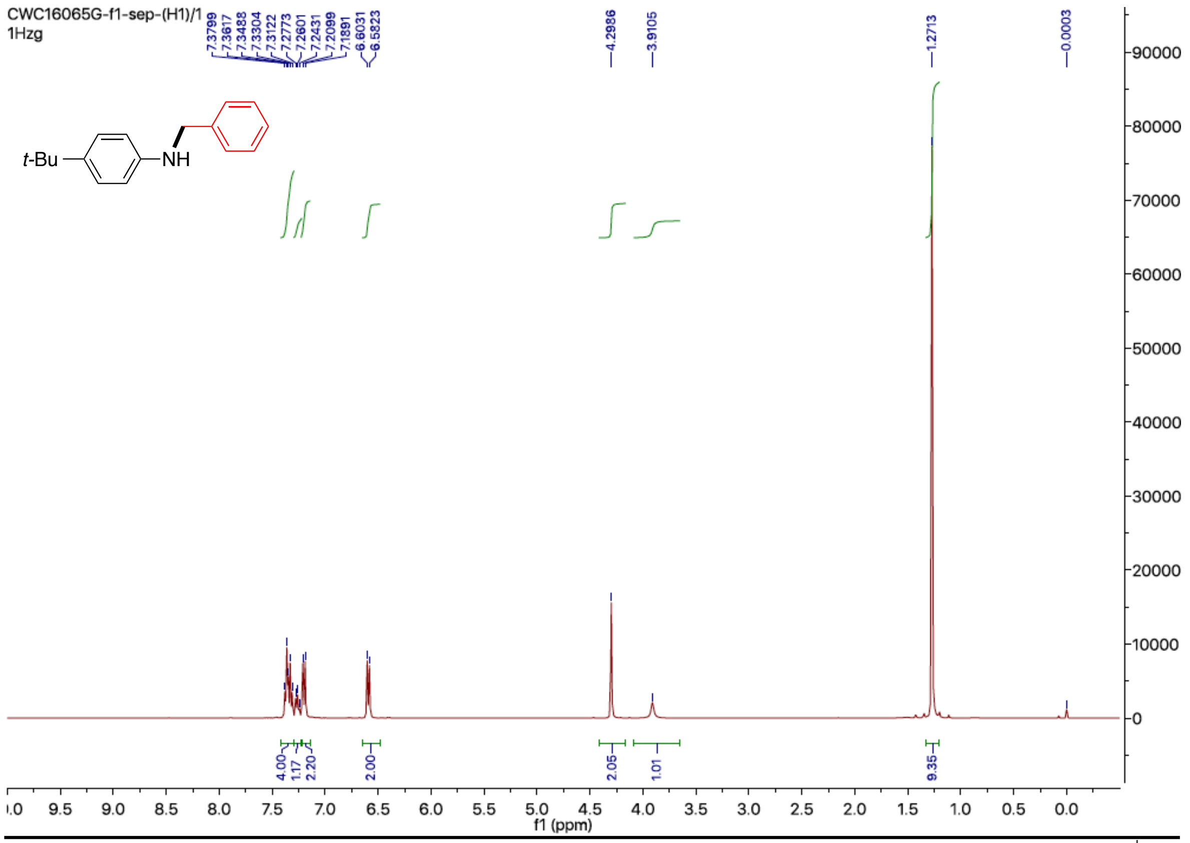

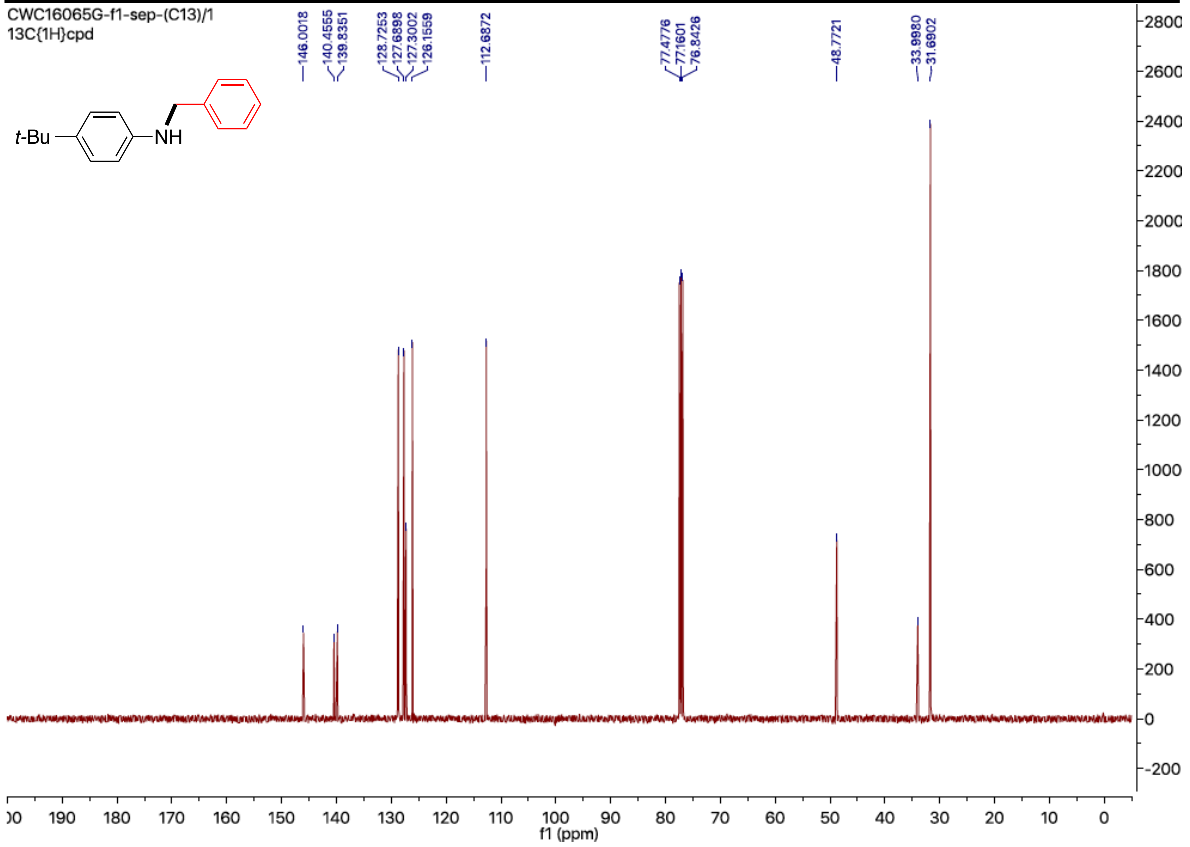

Supplementary Figure 102. ^{1}H and ^{13}C NMR spectra of *N*-Benzyl-4-(*tert*-butyl)aniline (6r)

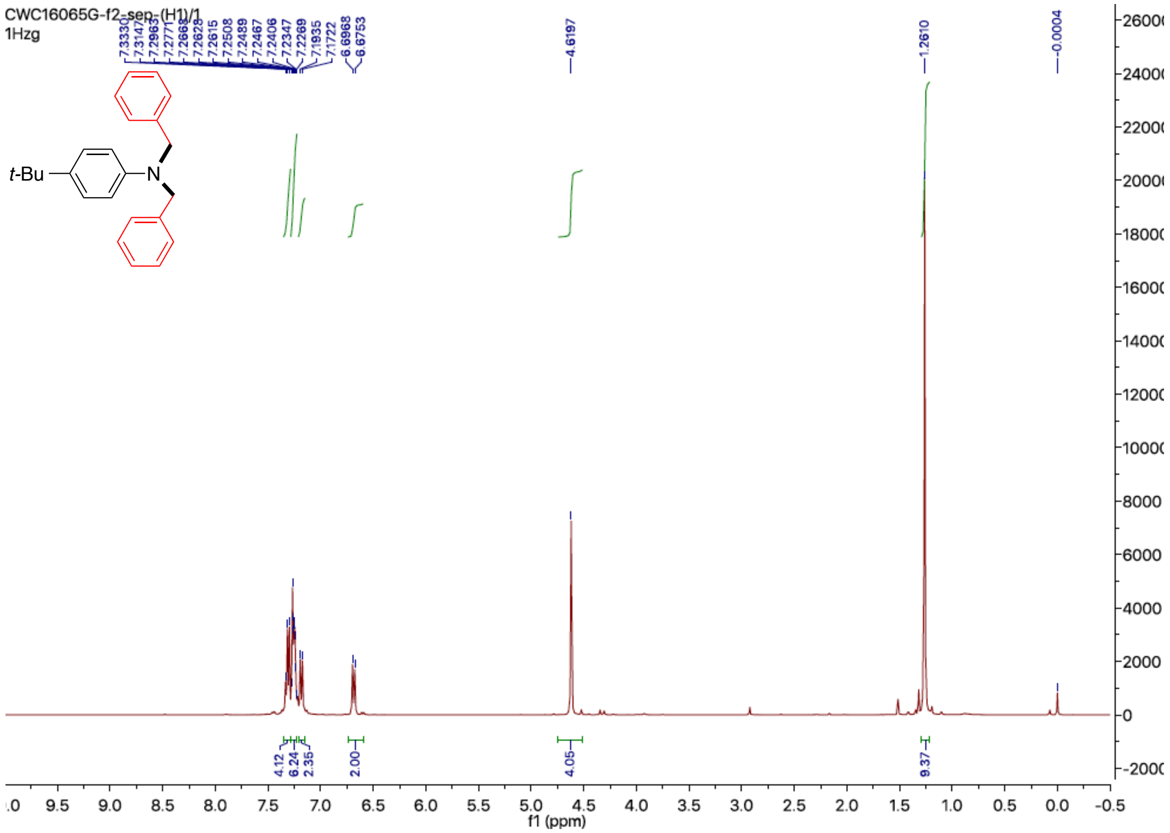

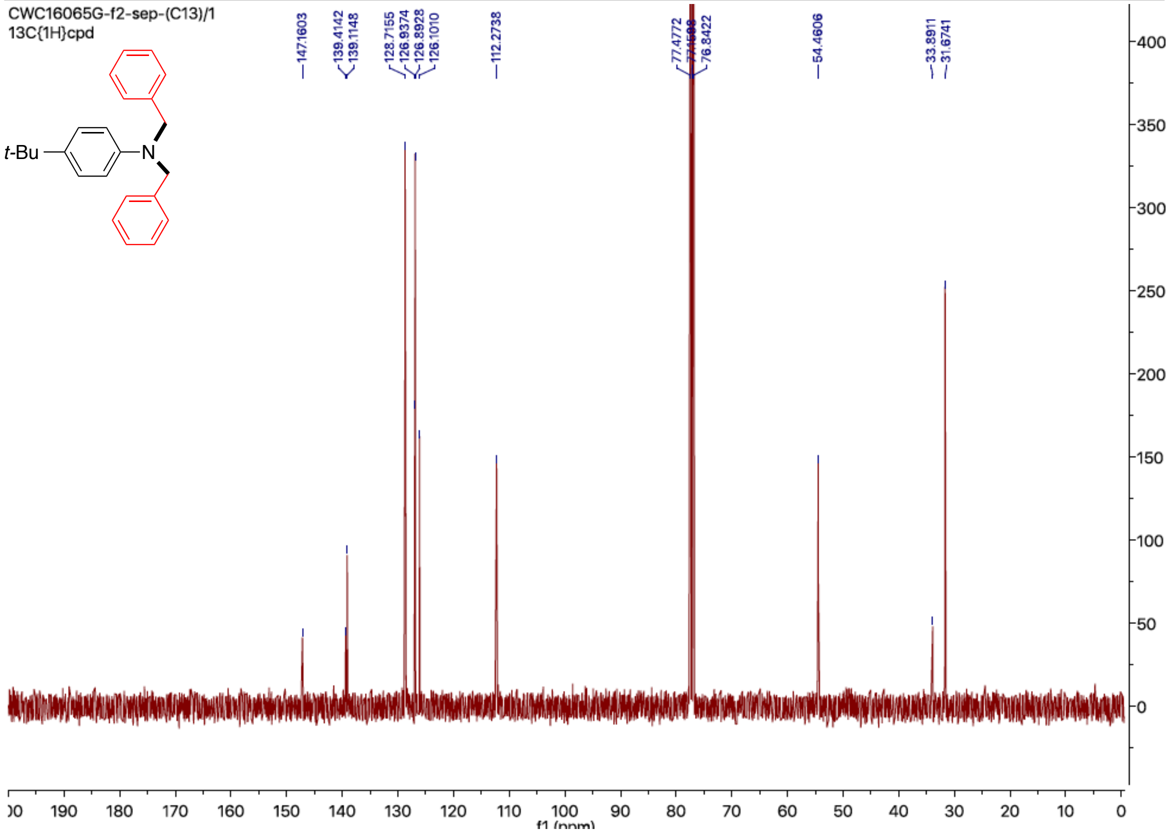

Supplementary Figure 103. ^{1}**H and** ^{13}**C NMR spectra of *N*,*N*-Dibenzyl-4-(*tert*-butyl)aniline (7r)**

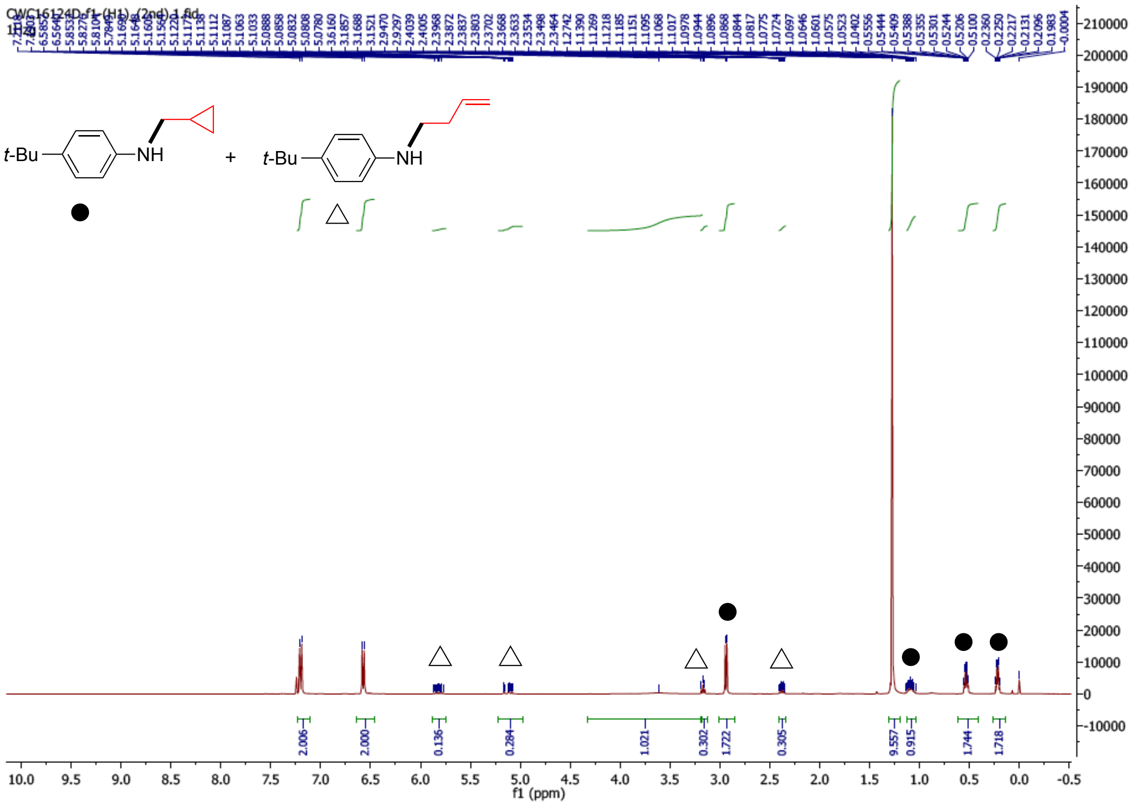

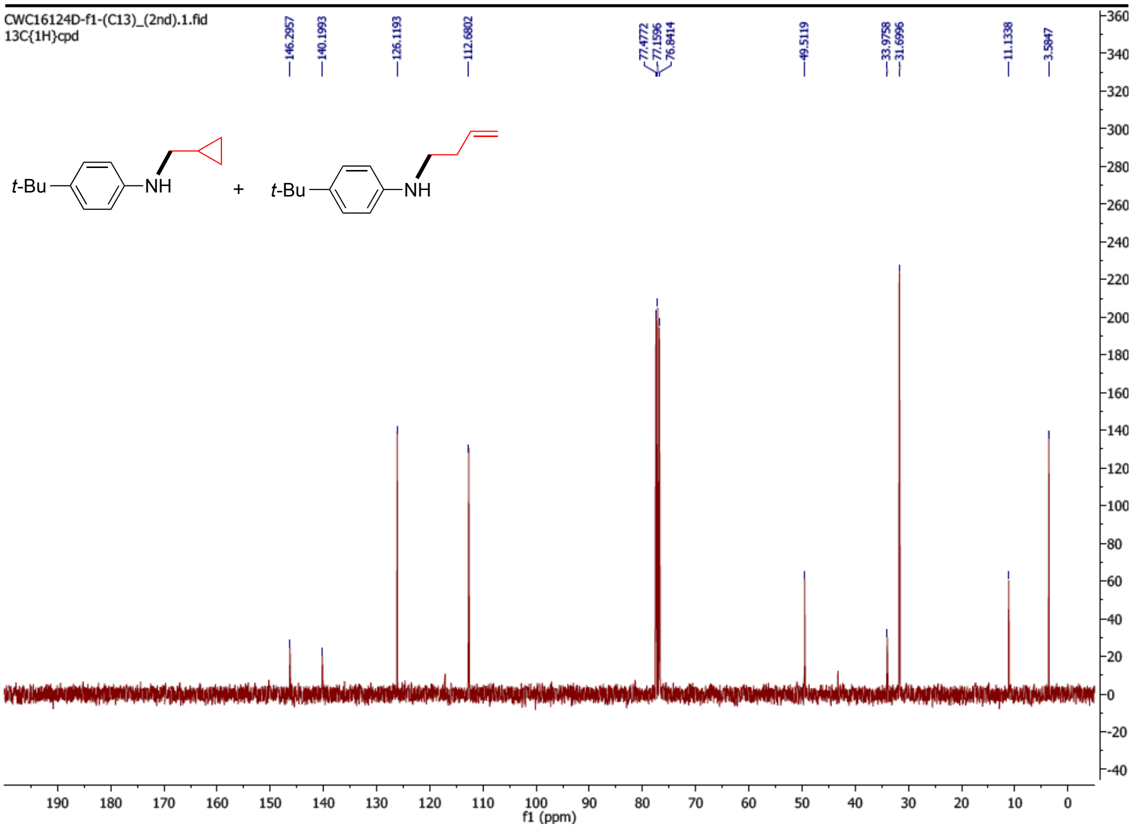

Supplementary Figure 104. ^{1}H and ^{13}C NMR spectra of 4-(*tert*-Butyl)-*N*-(cyclopropylmethyl)aniline (6s) and *N*-(but-3-en-1-yl)-4-(*tert*-butyl)aniline (6s').

Supplementary Tables

Supplementary Table 1. Optimization of Transition Metal Catalyst for Reductive Coupling of Nitrobenzene with Secondary Alkyl Iodide.

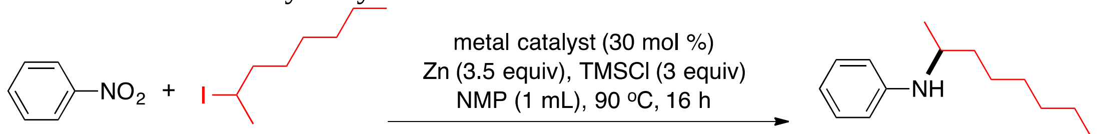

1a (1 equiv) (0.25 mmol) **2a** (3 equiv) **3a**

entry	metal catalyst	GC yield (%)[a]
1	$FeCl_2$	20
**2**	**$FeCl_2 \cdot 4H_2O$**	**100**
3	$FeBr_2$	52
4	FeI_2	44
5	FeF_2	55
6	$Fe(OTf)_2$	49
7	$Fe(OAc)_2$	21
8	$FeSO_4 \cdot 7H_2O$	69
9	$FeCl_3$	45
10	$FeCl_3 \cdot 6H_2O$	85
11	$FeBr_3$	89
12	$Fe(acac)_3$	18
13	FeF_3	44
14	$FeF_3 \cdot 3H_2O$	100
15	$Fe_2(SO_4)_3 \cdot 5H_2O$ (15 mol %)	83
16	$Fe(NO_3)_3 \cdot 9H_2O$	79
17	CuI	6
18	$CuBr_2$	12
19	$Cu(ClO_4)_2 \cdot 6H_2O$	64
20	$CoBr_2$	30
21	$CoCl_2 \cdot 6H_2O$	68
22	$Co(NO_3)_2 \cdot 6H_2O$	62
23	$NiCl_2 \cdot 6H_2O$	58
24	$Ni(BF_4)_2 \cdot 6H_2O$	70
25	$MnSO_4 \cdot H_2O$	46
26	$Mn(NO_3)_2 \cdot 4H_2O$	59
27	$CrCl_2$	47
28	$Pd(OAc)_2$	13
29	$AgNO_3$	5

(a) Corrected GC yield using *n*-dodecane as an internal standard.

Supplementary Table 2. Optimization of Loading of Iron Catalyst for Reductive Coupling of Nitrobenzene with Secondary Alkyl Iodide.

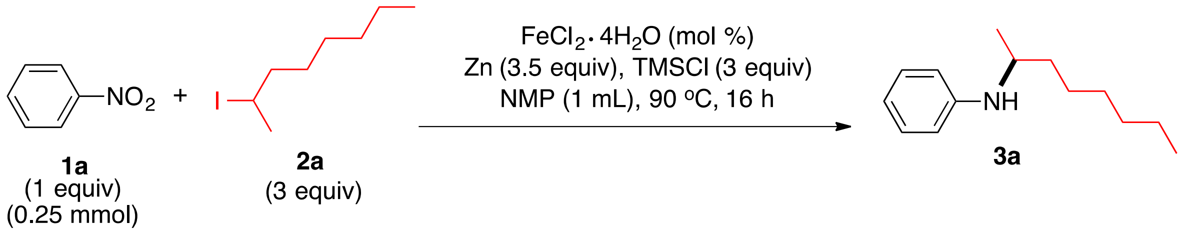

entry	$FeCl_2 \cdot 4H_2O$ (mol %)	GC yield (%)[a]
1	30	100
**2**	**20**	**98**
3	10	69

(a) Corrected GC yield using *n*-dodecane as an internal standard.

Supplementary Table 3. Optimization of Halotrimethylsilane for Reductive Coupling of Nitrobenzene with Secondary Alkyl Iodide.

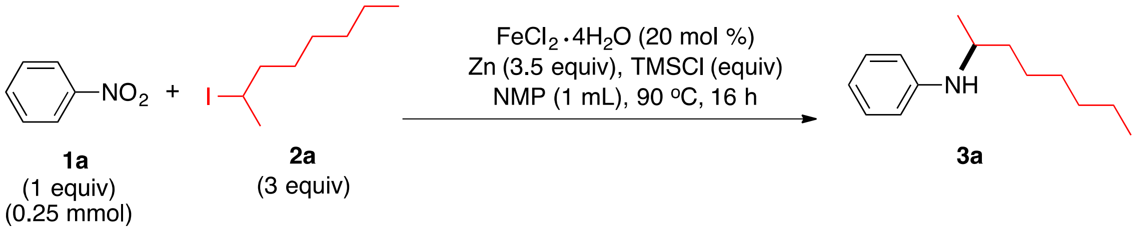

entry	TMSX (equiv)	GC yield (%)[a]
1	TMSCl (3)	96
2	TMSBr (3)	90
3	TMSI (3)	94
**4**	**TMSCl (2)**	**90**

(a) Corrected GC yield using *n*-dodecane as an internal standard.

Supplementary Table 4. Optimization of Solvent for Reductive Coupling of Nitrobenzene with Secondary Alkyl Iodide.

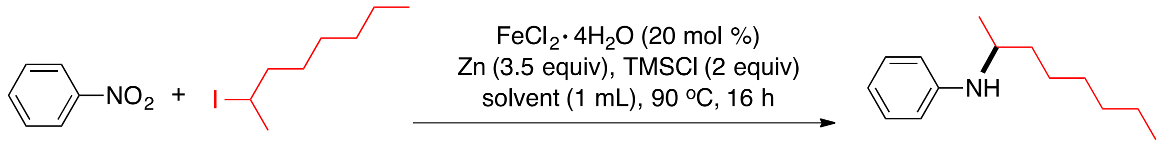

1a (1 equiv) (0.25 mmol) | **2a** (3 equiv) | **3a**

entry	solvent	GC yield (%)[a]
**1**	**NMP**	**90**
2	DMA	76
3	DMF	30
4	DMSO	51
5	THF	48
6	MeCN	<5

(a) Corrected GC yield using *n*-dodecane as an internal standard.

Supplementary Table 5. Optimization of Loadings of Alkyl Iodide, Zinc and Chlorotrimethylsilane, and Solvent Volume for Reductive Coupling of Nitrobenzene with Secondary Alkyl Iodide.

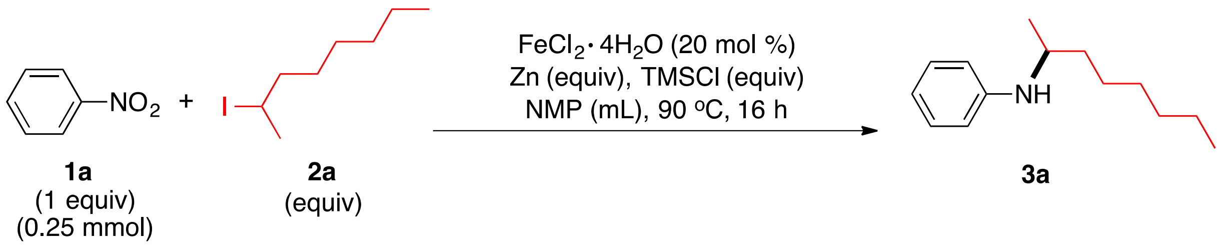

entry	alkyl iodide (equiv)	Zn (equiv)	TMSCl (equiv)	NMP (mL)	GC yield (%)[a]
1	3	3.5	2	1	90
2	3	3.5	2	2	78
3	3	3.5	2	0.5	100
**4**	**3**	**3**	**2**	**0.5**	**100**
5	3	2.5	2	0.5	87
6	3	2	2	0.5	55
7	3	3	1.5	0.5	92
8	3	3	1	0.5	82
9	3	3	0.5	0.5	78
10	3	3	0	0.5	73
11	2.5	3	2	0.5	90
12	2	3	2	0.5	80
13	2.5	2.5	2	0.5	77
14	2	3	3	0.5	89
15	2	3	4	0.5	76

(a) Corrected GC yield using *n*-dodecane as an internal standard.

Supplementary Table 6. Optimization of Reaction Temperature for Reductive Coupling of Nitrobenzene with Secondary Alkyl Iodide.

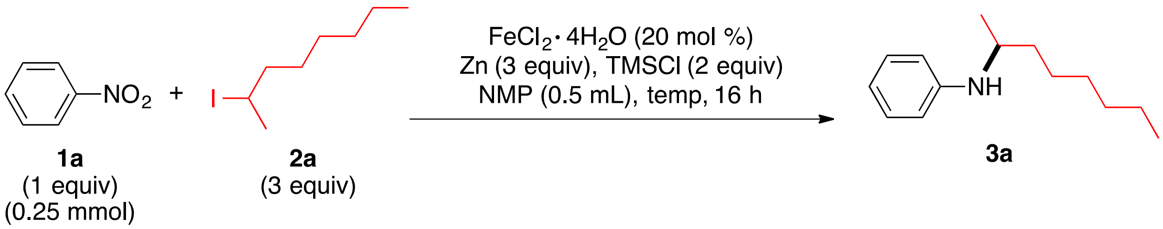

entry	temp (°C)	GC yield (%)[a]
**1**	**90**	**100**
2	60	84
3	rt	22

(a) Corrected GC yield using *n*-dodecane as an internal standard.

Supplementary Table 7. Effect of Additive in Reductive Coupling of Nitrobenzene with Secondary Alkyl Iodide.

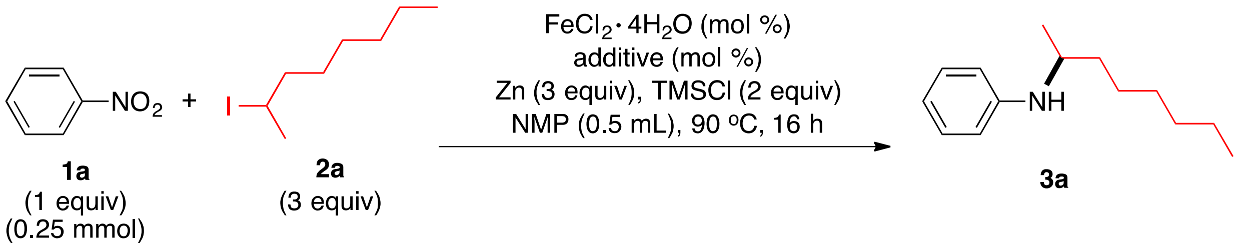

entry	$FeCl_2 \cdot 4H_2O$ (mol %)	additive (mol %)	GC yield (%)[a]
**1**	**20**	**---**	**100**
2	10	bipy (15)	67
3	10	phen (15)	54
4	10	TMEDA (20)	62
5	10	pyridine (40)	61
6	10	DMAP (40)	79
7	10	H_2O (200)	87
8	20	air atm	71

(a) Corrected GC yield using *n*-dodecane as an internal standard.

Supplementary Table 8. Control Experiment for Reductive Coupling of Nitrobenzene with Secondary Alkyl Iodide.

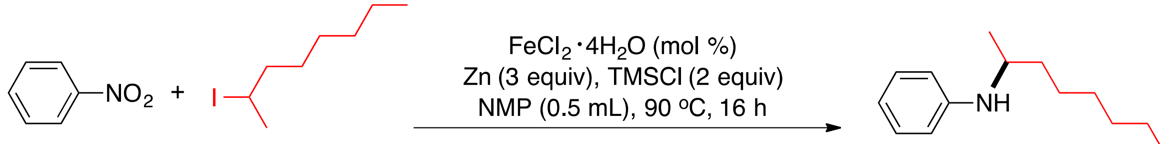

1a (1 equiv) (0.25 mmol); **2a** (3 equiv); **3a**

entry	$FeCl_2\cdot4H_2O$ (mol %, >99% purity)	GC yield (%)[a]
1	20	100
2	anhydrous $FeCl_2$ (20 mol %) instead	23
3	20 (99.99% purity)	96

(a) Corrected GC yield using *n*-dodecane as an internal standard.

Supplementary Table 9. Summary of Optimization of Iron-Catalyzed Reductive Coupling of Nitrobenzene with Secondary Alkyl Iodide.

"standard conditions":

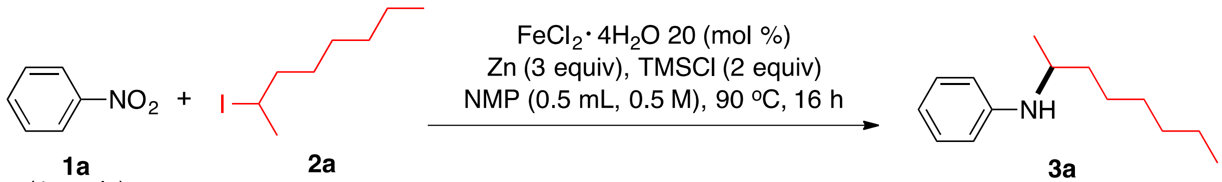

1a (1 equiv) (0.25 mmol); **2a** (3 equiv); **3a**

entry	variation from the "standard conditions"	yield (%)[a]
1	none	100
2	anhydrous $FeCl_2$ instead of $FeCl_2\cdot4H_2O$	23
3	No TMSCl was added	73
4	TMSBr instead of TMSCl	77
5	TMSI instead of TMSCl	75
6	2-iodooctane (2.5 equiv) instead of (3 equiv)	90
7	Zn (2.5 equiv) instead of (3 equiv)	87
8	60 °C instead of 90 °C	84
9	$FeCl_2\cdot4H_2O$ (10 mol %) with TMEDA (20 mol %) instead of $FeCl_2\cdot4H_2O$ (20 mol %)	62

(a) Corrected GC yield using *n*-dodecane as an internal standard.

Supplementary Table 10. Optimization of Fe-Catalyzed Reductive Coupling of Nitrobenzene with Primary Alkyl Iodide.

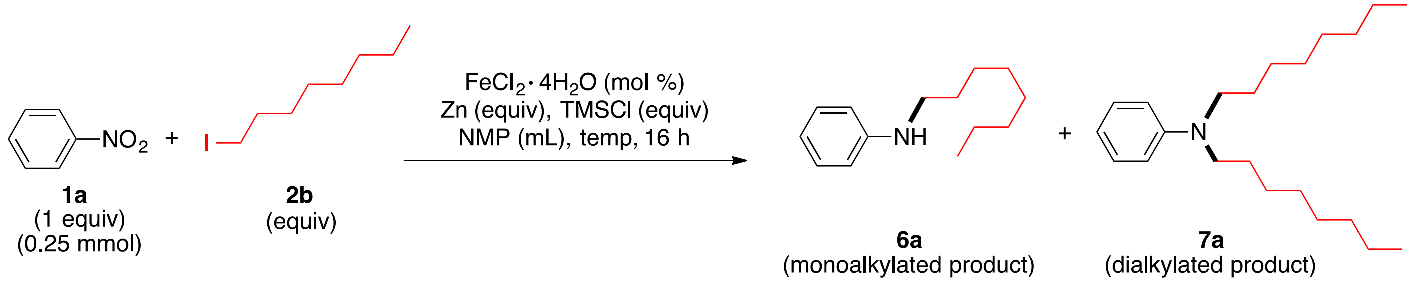

entry	RI (equiv)	Fe cat. (mol %)	Zn (equiv)	TMSCl (equiv)	NMP (mL)	temp (°C)	yield of **6a** (%)	yield of **7a** (%)
1	3	20	3	2	0.5	90	18	78
2	1.5	20	3	2	0.5	90	48	42
3	1.5	20	3	3	0.5	90	48	34
4	1.5	20	4	3	0.5	90	55	32
5	1.5	20	4	4	0.5	90	66	35
6	1.5	20	5	4	0.5	90	72	25
7	1.5	20	5	5	0.5	90	66	25
**8**	**1.5**	**20**	**5**	**4**	**1**	**90**	**76**	**15**
9	1.5	20	5	4	2	90	69	11
10	1.5	10	5	4	1	90	64	24
11	1.5	30	5	4	1	90	73	13
12	1.2	20	5	4	1	90	68	15
13	1.7	20	5	4	1	90	70	19
14	1.5	20	5	4	1	60	63	5
15	1.5	20	5	4	1	120	67	14

(a) Corrected GC yield using *n*-dodecane as an internal standard.

Supplementary Methods

(A) General Analytical Information.

Nuclear Magnetic Resonance spectra were recorded on a Bruker Avance 400 MHz instruments at ambient temperature. All ^1H NMR spectra were measured in part per million (ppm) relative to the signals of tetramethylsilane (TMS) added into the deuterated chloroform ($CDCl_3$) (0.00 ppm) unless otherwise stated.[1] Data for ^1H NMR were reported as follows: chemical shift, multiplicity (s = singlet, d = doublet, t = triplet, q = quartet, qu = quintet, sex = sextet, m = multiplet, ovrlp = overlap, br = broad), coupling constants, and integration. All ^{13}C NMR spectra were reported in ppm relative to $CDCl_3$ (77.16 ppm) unless otherwise stated (*31*), and were obtained with complete ^1H decoupling. All GC analyses were performed on a Perkin-Elmer Clarus 400 GC system with a FID detector. All GC-MS analyses were performed on an Agilent Technologies 7890A GC system equipped with a 5975C MS detector. High-resolution mass spectra (HRMS) by electrospray ionization (ESI) method were performed at the EPFL ISIC Mass Spectroscopy Service with a Micro Mass QTOF Ultima spectrometer.

(B) General Reagent Information.

Unless otherwise noted, all chemicals were used as received without further purifications. Anhydrous *N*-methylpyrrolidone (NMP) (99.8% purity) and anhydrous dimethylacetamide (DMA) (99.8% purity) were purchased from Aldrich Chemical Co. and Acros Chemicals, respectively, in Sure-Seal bottles and stored under nitrogen. Iron(II) chloride tetrahydrate ($FeCl_2 \bullet 4H_2O$, >99% and 99.99% purity), zinc powder (Zn, >98% purity), and chlorotrimethylsilane (TMSCl, ≥98% purity) were purchased from Aldrich Chemical Co.. All alkyl halides and the corresponding *N*-alkylated aniline products were in the form of racemic mixtures unless otherwise noted.

The following known starting materials (alkyl halides and nitroarenes) were prepared according to the literature procedures:[2-29]

(i) Nitroarenes

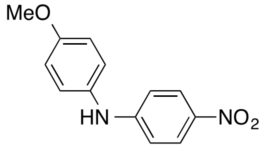

4-methoxy-*N*-(4-nitrophenyl)-aniline[2]

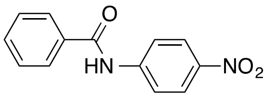

N-(4-nitrophenyl)-benzamide[3]

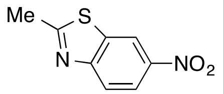

2-methyl-6-nitrobenzo-[*d*]thiazole[4]

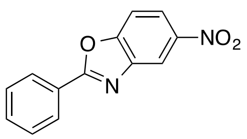

5-nitro-2-phenylbenzo-[*d*]oxazole[5]

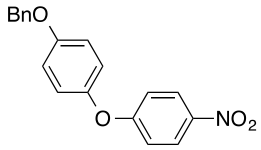

1-(benzyloxy)-4-(4-nitrophenoxy)benzene[6]

(ii) Alkyl Halides

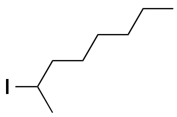

2-iodooctane[7]

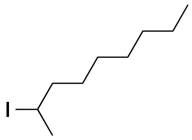

2-iodononane[8]

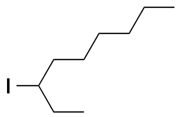

3-iodononane[9]

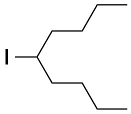

5-iodononane[8]

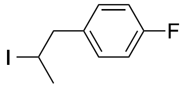

1-fluoro-4-(2-iodopropyl)-benzene[10]

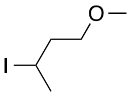

3-iodo-1-methoxy-butan[10]

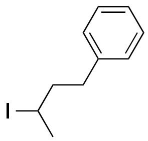

(3-iodobutyl)-benzene[11]

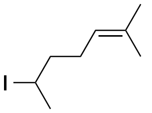

6-iodo-2-methy-lhept-2-ene[11]

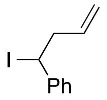

(1-iodobut-3-en-1-yl)benzene[12]

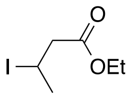

ethyl 3-iodo-butanoate[8]

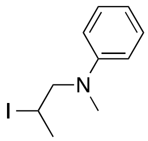

N-(2-iodopropyl)-*N*-methylaniline[8]

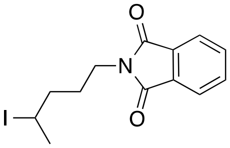

2-(4-iodopentyl)isoindoline-1,3-dione[13]

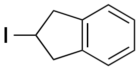

2-iodo-2,3-dihydro-1*H*-indene[14]

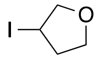

3-iodotetra-hydrofuran[15]

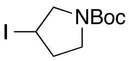

tert-butyl 3-iodo pyrrolidine-1-carboxylate[16]

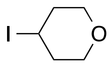

4-iodotetrahydro-2*H*-pyran[17]

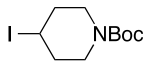

tert-butyl 4-iodo-piperidine-1-carboxylate[11]

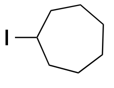

iodocyclo-heptane[18]

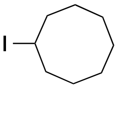

iodocyclo-octane[9]

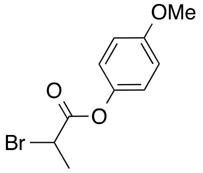

4-methoxyphenyl 2-bromopropanoate[19]

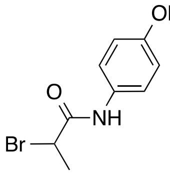

2-bromo-*N*-(4-methoxy phenyl)propanamide[20]

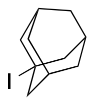

1-iodo-adamantane[21]

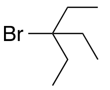

3-bromo-3-ethylpentane[22]

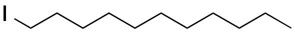

1-iodoundecane[23]

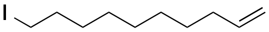

10-iododec-1-ene[24]

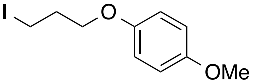

1-(3-iodopropoxy)-4-methoxybenzene[25]

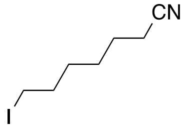

7-iodoheptanenitrile[26]

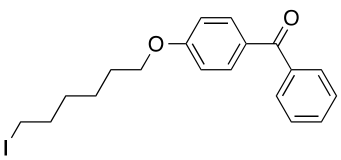

(4-((6-iodohexyl)oxy)phenyl)-(phenyl)methanon[27]

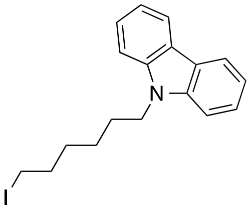

9-(6-iodohexyl)-9*H*-carbazole[28]

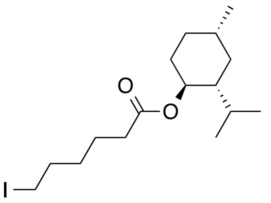

(+/-)-2-isopropyl-4-methyl-cyclohexyl 6-iodohexanoate[29]

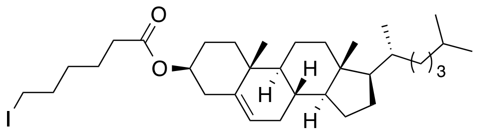

(3*S*,8*S*,9*S*,10*R*,13*R*,14*S*,17*R*)-10,13-dimethyl-17-((*R*)-4-methylpentan-2-yl)-2,3,4,7,8,9,10,11,12,13,14,15,16,17-tetradecahydro-1*H*-cyclopenta-[*a*]phenanthren-3-yl 6-iodohexanoate[29]

(C) General Manipulation Considerations.

All manipulations for the iron-catalyzed reductive coupling reactions of nitroarenes with organohalides were set up in a 30 mL Teflon-screw capped test tubes under an inert nitrogen (N_2) atmosphere using glove-box techniques. The test tubes were then sealed with air-tight electrical tapes and the reaction mixtures were stirred in a heated oil-bath. Flash column chromatography was performed using silica gel (Silicycle, ultra pure grade). Preparative thin-layer chromatography (preparative TLC) was performed using preparative TLC plate (Merck Millipore, TLC Silica gel 60 F_{254}, 20 x 20 cm, catalogue number: 1.05715.0001) in a developing tank. Notably, the silica gel and the TLC plate used for the purification of aniline products were washed with hexanes/triethylamine solution (volume ratio ~20:1) prior to the use in order to minimize the product loss. The eluents for column chromatography and preparative TLC were presented as ratios of solvent volumes. Yields reported in the publication are of isolated materials unless otherwise noted. All new starting materials (nitroarenes and alkyl halides) were characterized by ^{1}H and ^{13}C NMR spectroscopies and high-resolution mass spectrometry (HRMS) (or gas chromatography-mass spectrometry (GC-MS)). All new secondary amine products were characterized by ^{1}H and ^{13}C NMR spectroscopies and HRMS; in case the molecular ions could not be detected by HRMS, GC-MS was used instead. All known secondary amine products were characterized by ^{1}H and ^{13}C NMR spectroscopies which were further compared with the literature values whenever possible.

Synthesis of Starting Materials:

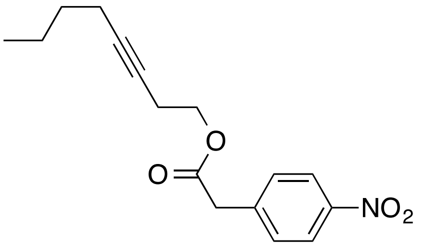

Oct-3-yn-1-yl 2-(4-nitrophenyl)acetate (S1). An oven-dried 30 mL re-sealable screw-cap test tube equipped with a Teflon-coated magnetic stir bar was charged with oct-3-yn-1-ol (1 equiv, 1.13 g, 9.0 mmol), 2-(4-nitrophenyl)acetic acid (1 equiv, 1.63 g, 9.0 mmol), THF solvent (20 mL), and concentrated sulfuric acid (1.0 mL). The resulting mixture was stirred at 60 ^{o}C in a preheated oil bath overnight. After the reaction, the reaction mixture was cooled down to room temperature, and the crude product was washed with EtOAc (~20 mL) and water (~100 mL). The aqueous fraction was removed, and the organic fraction was further washed with saturated Na_2CO_3 solution (~100 mL). The organic fraction was concentrated *in vacuo* with the aid of a rotary evaporator. The crude product residue was purified by column chromatography with silica gel (without prior washing with Et_3N/hexanes) using a solvent mixture of hexanes and EtOAc (10:1) as an eluent to afford the title compound (**S1**) as viscous brown oil (880 mg, 3.04 mmol, 34%). **^{1}H NMR** (400 MHz, $CDCl_3$): δ 8.19 (d, *J* = 8.2 Hz, 2 H), 7.48 (d, *J* = 8.2 Hz, 2 H), 4.19 (t, *J* = 6.8 Hz, 2 H), 3.76 (s, 2 H), 2.49 (t, *J* = 6.6 Hz, 2 H), 2.12 (t, *J* = 6.6 Hz, 2 H), 1.48-1.34 (ovrlp, 4 H), 0.90 (t, *J* = 6.7 Hz, 3 H). **^{13}C NMR** (100 MHz, $CDCl_3$): δ 170.0, 147.3, 141.3, 130.4, 123.8, 82.3, 75.2, 63.8, 41.0, 31.1, 22.0, 19.3, 18.5, 13.7. **HRMS**: Calcd for $C_{24}H_{37}NO_2$ [M+H]: 372.2903; Found: 372.2904.

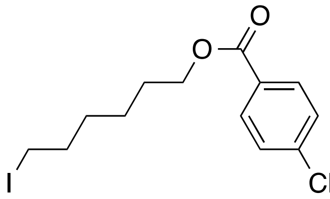

6-Iodohexyl 4-Chlorobenzoate (S2). An oven-dried 30 mL re-sealable screw-cap test tube equipped with a Teflon-coated magnetic stir bar was sequentially charged with 6-chlorohexan-1-ol (1 equiv, 4.10 g, 30 mmol), 4-chlorobenzoyl chloride (1.2 equiv, 6.30 g, 36 mmol), CH_2Cl_2 solvent (20 mL), and triethylamine (1.5 equiv, 6.3 mL, 45 mmol). The resulting mixture was stirred at room temperature overnight. After the reaction, the crude product was washed with diluted HCl solution (1 M, ~50 mL). The aqueous fraction was removed, and the organic fraction was further neutralized with saturated $NaHCO_3$ solution (~ 50 mL). The organic fraction was concentrated *in vacuo* with the aid of a rotary evaporator and further dried *in vacuo* to give a crude product, 6-bromohexyl 4-chlorobenzoate, in a nearly quantitative yield. The crude 6-chlorohexyl 4-chlorobenzoate was then introduced into a 250 mL round-bottom flask equipped with a Teflon-coated magnetic stir bar, followed by the addition of NaI (4 equiv, 18.0 g, 120 mmol), acetone (70 mL), and water (7 mL). The reaction mixture was then heated at 60 ^{o}C until all alkyl bromide was consumed as determined by GC analysis. After cooling to room temperature, the reaction mixture was concentrated *in vacuo* and

washed with CH_2Cl_2 (50 mL) and water (100 mL). The aqueous solution was further washed with CH_2Cl_2 (2 x 50 mL). The combined organic fractions were concentrated *in vacuo,* and the residue was purified by flash chromatography with silica gel (without prior washing with Et_3N/hexanes) using a mixture of hexanes/EtOAc (8:1) as an eluent to afford the title compound (**S2**) as colorless oil (7.47 g, 20.4 mmol, 68%). **^{1}H NMR** (400 MHz, $CDCl_3$): δ 7.97 (d, *J* = 8.6 Hz, 2 H), 7.41 (d, *J* = 8.6 Hz, 2 H), 4.32 (t, *J* = 6.6 Hz, 2 H), 3.20 (t, *J* = 7.0 Hz, 2 H), 1.86 (qu, *J* = 7.0 Hz, 2 H), 1.78 (qu, *J* = 6.8 Hz, 2 H), 1.51-1.43 (ovrlp, 4 H). **^{13}C NMR** (100 MHz, $CDCl_3$): δ 165.9, 139.4, 131.1, 129.0, 128.8, 65.2, 33.4, 30.3, 28.6, 25.2, 6.9. **GCMS**: $[M]^+$ = 366 and 368 (~3:1 ratio) was detected which corresponded to $C_{13}H_{16}ClIO_2$.

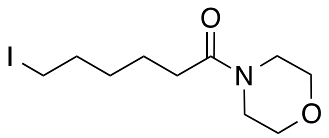

6-Iodo-1-morpholinohexan-1-one (S3). An oven-dried 30 mL re-sealable screw-cap test tube equipped with a Teflon-coated magnetic stir bar was sequentially charged with 6-bromohexanoyl chloride (1 equiv, 6.41 g, 30 mmol), morpholine (1.8 equiv, 4.8 mL, 54 mmol), CH_2Cl_2 solvent (20 mL), and triethylamine (2.0 equiv, 8.3 mL, 60 mmol). The resulting mixture was stirred at room temperature overnight. After the reaction, the crude product was washed with diluted HCl solution (1 M, ~50 mL). The aqueous fraction was removed, and the organic fraction was further neutralized with saturated $NaHCO_3$ solution (~50 mL). The organic fraction was concentrated *in vacuo* with the aid of a rotary evaporator and further dried *in vacuo* to give a crude product, 6-bromo-1-morpholinohexan-1-one, in a nearly quantitative yield. The crude 6-bromo-1-morpholinohexan-1-one was then introduced into a 250 mL round-bottom flask equipped with a Teflon-coated magnetic stir bar, followed by the addition of NaI (18.0 g, 120 mmol, 4 equiv), acetone (70 mL), and water (7 mL). The reaction mixture was then heated at 60 ^{o}C until all alkyl bromide was consumed as determined by GC analysis. After cooling to room temperature, the reaction mixture was concentrated *in vacuo* and washed with CH_2Cl_2 (50 mL) and water (100 mL). The aqueous solution was further washed with CH_2Cl_2 (2 x 50 mL). The combined organic fractions were concentrated *in vacuo*, and the residue was purified by flash chromatography with silica gel (without prior washing with Et_3N/hexanes) using a mixture of hexanes/EtOAc (2:1) as an eluent to afford the title compound (**S3**) as brown oil (4.95 g, 15.9 mmol, 53%). **^{1}H NMR** (400 MHz, $CDCl_3$): δ 3.69-3.66 (ovrlp, 4 H), 3.63-3.61 (m, 2 H), 3.48-3.45 (m, 2 H), 3.20 (t, *J* = 6.9 Hz, 2 H), 2.33 (t, *J* = 7.4 Hz, 2 H), 1.86 (qu, *J* = 7.5 Hz, 2 H), 1.67 (qu, *J* = 7.8 Hz, 2 H), 1.46 (qu, *J* = 8.3 Hz, 2 H). **^{13}C NMR** (100 MHz, $CDCl_3$): δ 171.5, 67.1, 66.8, 46.1, 42.1, 33.3, 32.9, 30.4, 24.2, 6.9. **GCMS**: $[M]^+$ = 311 was detected which corresponded to $C_{10}H_{18}INO_2$.

Synthesis of Alkyl Aryl Amines:

General Procedure for Iron-Catalyzed Reductive Coupling of Nitroarene with Secondary or Tertiary Alkyl Halide (General Procedure A). An oven-dried 30 mL re-sealable screw-cap test tube equipped with a Teflon-coated magnetic stir bar was sequentially charged with zinc powder (Zn, 3 equiv, 1.5 mmol, 98 mg), iron(II) chloride tetrahydrate ($FeCl_2•4H_2O$, >99% purity, 20 mol %, 0.10 mmol, 20 mg), nitroarene (1 equiv, 0.50 mmol), secondary/tertiary alkyl iodide (3 equiv, 1.5 mmol), *N*-methylpyrrolidone solvent (NMP, 1.0 mL), and chlorotrimethylsilane (TMSCl, 3 equiv, 1.5 mmol, 96 μL). The resulting mixture was stirred at 90 ^{o}C in a preheated oil bath for 16 h. After the reaction, the reaction mixture was cooled down to room temperature, and the crude product was acidified with saturated NH_4Cl solution (~4 mL) and then neutralized with saturated $NaHCO_3$ solution (~6 mL). The crude product in the aqueous fraction was extracted with EtOAc (~20 mL). The aqueous fraction was further washed with EtOAc (3 x ~10 mL). The combined organic fractions were concentrated *in vacuo* with the aid of a rotary evaporator. The crude product residue was purified by flash column chromatography with silica gel using a solvent mixture (EtOAc, hexanes) as an eluent to afford the purified secondary arylamine (**3-5**).

General Procedure for Iron-Catalyzed Reductive Coupling of Nitroarene with Primary Alkyl Halide (General Procedure B). An oven-dried 30 mL re-sealable screw-cap test tube equipped with a Teflon-coated magnetic stir bar was sequentially charged with zinc powder (Zn, 5 equiv, 2.5 mmol, 164 mg), iron(II) chloride tetrahydrate ($FeCl_2•4H_2O$, >99% purity, 20 mol %, 0.10 mmol, 20 mg), nitroarene (1 equiv, 0.50 mmol), primary alkyl iodide (1.5 equiv, 0.75 mmol), *N*-methylpyrrolidone solvent (NMP, 2.0 mL), and chlorotrimethylsilane (TMSCl, 4 equiv, 2.0 mmol, 128 μL). The resulting mixture was stirred at 90 ^{o}C in a preheated oil bath for 16 h. After the reaction, the reaction mixture was cooled down to room temperature, and the crude product was acidified with saturated NH_4Cl solution (~4 mL) and then neutralized with saturated $NaHCO_3$ solution (~6 mL). The crude product in the aqueous fraction was extracted with EtOAc (~20 mL). The aqueous fraction was further washed with EtOAc (3 x ~10 mL). The combined organic fractions were concentrated *in vacuo* with the aid of a rotary evaporator. The crude product residue was purified by preparative TLC using a solvent mixture (EtOAc, hexanes) as an eluent to afford the purified secondary (**6**) and tertiary anilines (**7**).

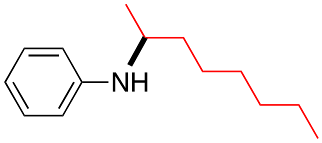

***N*-(Octan-2-yl)aniline (3a).**[30]

(i) 1.0 mmol scale: Following the general procedure A, the title compound was prepared using Zn (3.0 mmol, 196 mg), $FeCl_2 \bullet 4H_2O$ (0.20 mmol, 40 mg), nitrobenzene (1.0 mmol, 102 μL), 2-iodooctane (3.0 mmol, 720 mg), NMP (2.0 mL), and TMSCl (3.0 mmol, 256 μL). The crude product was purified by flash chromatography using hexanes as an eluent to afford the title compound (**3a**) as yellow oil (178 mg, 87%). **^{1}H NMR** (400 MHz, $CDCl_3$): δ 7.14 (t, J = 7.5 Hz, 2 H), 6.64 (t, J = 7.5 Hz, 1 H), 6.56 (d, J = 7.9 Hz, 2 H), 3.48-3.24 (ovrlp, 2 H), 1.60-1.50 (m, 1 H), 1.44-1.25 (ovrlp, 9 H), 1.16 (d, J = 6.3 Hz, 3 H), 0.88 (t, J = 6.7 Hz, 3 H). **^{13}C NMR** (100 MHz, $CDCl_3$): δ 147.8, 129.4, 116.8, 113.2, 48.6, 37.4, 32.0, 29.5, 26.3, 22.8, 20.9, 14.2.

(ii) 10 mmol scale: Following the general procedure A, the title compound was prepared using Zn (3 equiv, 30 mmol, 1.96 g), $FeCl_2 \bullet 4H_2O$ (20 mol %, 2.0 mmol, 398 mg), nitrobenzene (1 equiv, 10 mmol, 1.23 g), 2-iodooctane (3 equiv, 30 mmol, 7.21 g), NMP (15 mL), and TMSCl (2 equiv, 20 mmol, 2.6 mL), and the reaction was stirred at 90 ^{o}C for 2 days. The crude product was purified by flash chromatography using hexanes as an eluent to afford the title compound (**3a**) as yellow oil (2.05 g, 72%). Spectral and analytical data were identical to those reported for the same compound above.

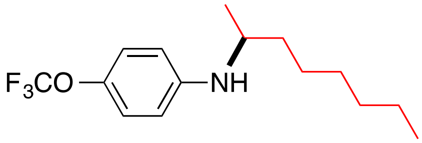

***N*-(Octan-2-yl)-4-(trifluoromethoxy)aniline (3b).** Following the general procedure A, the title compound was prepared using 1-nitro-4-(trifluoromethoxy)benzene (104 mg) and 2-iodooctane (360 mg). The crude product was purified by flash chromatography using hexanes as an eluent to afford the title compound (**3b**) as yellow oil (90 mg, 62%). **^{1}H NMR** (400 MHz, $CDCl_3$): δ 7.00 (d, J = 8.3 Hz, 2 H), 6.50 (d, J = 8.6 Hz, 2 H), 3.48 (br s, 1 H), 3.39 (sex, J = 6.0 Hz, 1 H), 1.59-1.49 (m, 1 H), 1.45-1.25 (ovrlp, 9 H), 1.16 (d, J = 6.0 Hz, 3 H), 0.88 (t, J = 6.4 Hz, 3 H). **^{13}C NMR** (100 MHz, $CDCl_3$): δ 146.6, 140.0 (q, $^3J_{CF}$ = 1.9 Hz), 122.6, 120.9 (q, $^1J_{CF}$ = 253.5 Hz), 113.2, 48.9, 37.3, 32.0, 29.5, 26.2, 22.8, 20.8, 14.2. **HRMS** (ESI): Calcd for $C_{15}H_{23}F_3NO$ [M+H]: 290.1732; Found: 290.1726.

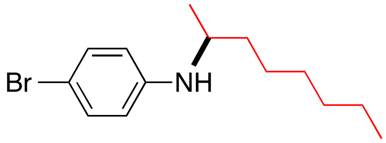

4-Bromo-*N*-(octan-2-yl)aniline (3c).[31] Following the general procedure A, the title compound was prepared using 1-bromo-4-nitrobenzene (101 mg) and 2-iodooctane (360 mg). The crude product was purified by flash chromatography using hexanes as an eluent to afford the title compound (**3c**) as pale-brown oil (95 mg, 67%). **^{1}H NMR** (400 MHz, $CDCl_3$): δ 7.21 (d, J = 8.2 Hz, 2 H), 6.43 (d, J = 8.2 Hz, 2 H), 3.46-3.34 (ovrlp, 2 H), 1.58-1.49 (m, 1 H), 1.43-1.24 (ovrlp, 9 H), 1.15 (d, J = 5.4 Hz, 3 H), 0.88 (t, J = 6.5 Hz, 3 H). **^{13}C NMR** (100 MHz, $CDCl_3$): δ 146.8, 132.0, 114.7, 108.2, 48.7, 37.2, 32.0, 29.5, 26.2, 22.7, 20.7, 14.2.

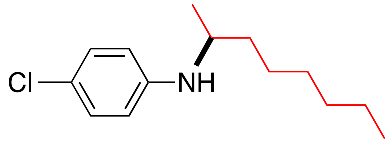

4-Chloro-*N*-(octan-2-yl)aniline (3d).[32] Following the general procedure A, the title compound was prepared using 1-chloro-4-nitrobenzene (79 mg) and 2-iodooctane (360 mg). The crude product was purified by flash chromatography using hexanes as an eluent to afford the title compound (**3d**) as yellow oil (79 mg, 66%). **^{1}H NMR** (400 MHz, $CDCl_3$): δ 7.08 (d, *J* = 7.9 Hz, 2 H), 6.47 (d, *J* = 7.8 Hz, 2 H), 3.44-3.35 (ovrlp, 2 H), 1.58-1.49 (m, 1 H), 1.43-1.24 (ovrlp, 9 H), 1.15 (d, *J* = 5.2 Hz, 3 H), 0.88 (d, *J* = 6.6 Hz, 3 H). **^{13}C NMR** (100 MHz, $CDCl_3$): δ 146.4, 129.2, 121.3, 114.2, 48.8, 37.2, 32.0, 29.5, 26.2, 22.8, 20.8, 14.2.

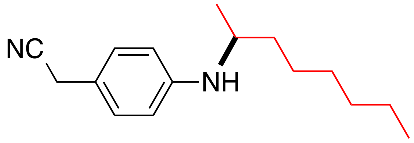

2-(4-(Octan-2-ylamino)phenyl)acetonitrile (3e). Following the general procedure A, the title compound was prepared using 2-(4-nitrophenyl)acetonitrile (81 mg) and 2-iodooctane (360 mg). The crude product was purified by flash chromatography using hexanes and then hexanes/EtOAc (10:1) as eluents to afford the title compound (**3e**) as viscous deep-brown oil (79 mg, 65%). **^{1}H NMR** (400 MHz, $CDCl_3$): δ 7.08 (d, *J* = 7.8 Hz, 2 H), 6.55 (d, *J* = 7.8 Hz, 2 H), 3.60 (s, 2 H), 3.54 (br s, 1 H), 3.44 (sex, *J* = 5.8 Hz, 1 H), 1.59-1.51 (m, 1 H), 1.45-1.23 (ovrlp, 9 H), 1.16 (d, *J* = 5.9 Hz, 3 H), 0.89 (t, *J* = 6.1 Hz, 3 H). **^{13}C NMR** (100 MHz, $CDCl_3$): δ 147.5, 129.0, 118.8, 117.3, 113.4, 48.5, 37.2, 31.9, 29.4, 26.2, 22.8, 22.7, 20.7, 14.2. **HRMS** (ESI): Calcd for $C_{16}H_{25}N_2$ [M+H]: 245.2018; Found: 245.2015.

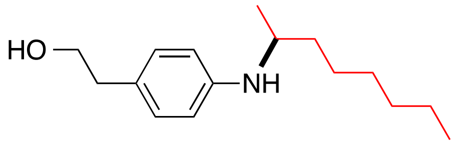

2-(4-(Octan-2-ylamino)phenyl)ethan-1-ol (3f). Following the general procedure A, the title compound was prepared using 2-(4-nitrophenyl)ethan-1-ol (84 mg) and 2-iodooctane (360 mg). The crude product was purified by flash chromatography using hexanes and then hexanes/EtOAc (10:1) as eluents to afford the title compound (**3f**) as viscous brown oil (62 mg, 50%). **^{1}H NMR** (400 MHz, $CDCl_3$): δ 7.01 (d, *J* = 7.7 Hz, 2 H), 6.53 (d, *J* = 7.7 Hz, 2 H), 3.77 (d, *J* = 6.2 Hz, 2 H), 3.42 (sex, *J* = 5.6 Hz, 1 H), 2.73 (t, *J* = 6.1 Hz, 2 H), 1.59-1.49 (m, 1 H), 1.43-1.24 (ovrlp, 9 H), 1.15 (d, *J* = 6.0 Hz, 3 H), 0.88 (t, *J* = 6.1 Hz, 3 H) (proton of N-H group was not observed likely due to the boarding effect). **^{13}C NMR** (100 MHz, $CDCl_3$): δ 146.5, 130.0, 126.3, 113.4, 64.1, 48.8, 38.4, 37.4, 32.0, 29.5, 26.3, 22.7, 20.9, 14.2. **HRMS** (ESI): Calcd for $C_{16}H_{28}NO$ [M+H]: 250.2165; Found: 250.2168.

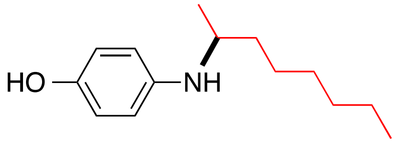

4-(Octan-2-ylamino)phenol (3g). Following the general procedure A, the title compound was prepared using 4-nitrophenol (1 equiv, 0.5 mmol, 70 mg) and 2-iodooctane (2 equiv, 1.0 mmol, 240 mg). The crude product was purified by flash chromatography using hexanes and then hexanes/EtOAc (10:1) as eluents to afford the title compound (**3g**) as a brown solid (53 mg, 48%). **^{1}H NMR** (400 MHz, $CDCl_3$): δ 6.69 (d, J = 7.9 Hz, 2 H), 6.50 (d, J = 7.8 Hz, 2 H), 4.65-3.38 (br ovrlp, 2 H), 3.34 (sex, J = 5.4 Hz, 1 H), 1.59-1.50 (m, 1 H), 1.43-1.23 (ovrlp, 9 H), 1.14 (d, J = 6.1 Hz, 3 H), 0.88 (t, J = 6.4 Hz, 3 H). **^{13}C NMR** (100 MHz, $CDCl_3$): δ 147.5, 142.0, 116.4, 115.1, 49.8, 37.4, 32.0, 29.5, 26.3, 22.8, 20.9, 14.2. **HRMS** (ESI): Calcd for $C_{14}H_{24}NO$ [M+H]: 222.1852; Found: 222.1857.

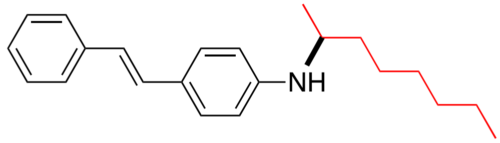

***N*-(Octan-2-yl)-4-styrylaniline (3h).** Following the general procedure A, the title compound was prepared using (*E*)-1-nitro-4-styrylbenzene (113 mg) and 2-iodooctane (360 mg). The crude product was purified by flash chromatography using hexanes as an eluent to afford the title compound (**3h**) as pale-brown solid (81 mg, 52%). **^{1}H NMR** (400 MHz, $CDCl_3$): δ 7.45 (d, J = 7.6 Hz, 2 H), 7.34-7.29 (ovrlp, 4 H), 7.18 (t, J = 7.1 Hz, 1 H), 7.01 (d, J = 16.2 Hz, 1 H), 6.87 (d, J = 16.2 Hz, 1 H), 6.54 (d, J = 8.0 Hz, 2 H), 3.53 (br s, 1 H), 3.51-3.37 (m, 1 H), 1.59-1.49 (m, 1 H), 1.46-1.24 (ovrlp, 9 H), 1.17 (d, J = 5.7 Hz, 3 H), 0.88 (t, J = 6.4 Hz, 3 H). **^{13}C NMR** (100 MHz, $CDCl_3$): δ 147.6, 138.3, 129.0, 128.7, 127.9, 126.7, 126.3, 126.1, 124.2, 113.2, 48.6, 37.3, 32.0, 29.5, 26.2, 22.8, 20.9, 14.2. **HRMS** (ESI): Calcd for $C_{22}H_{30}N$ [M+H]: 308.2373; Found: 308.2375

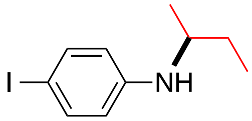

***N*-(*sec*-Butyl)-4-iodoaniline (3i).** Following the general procedure A, the title compound was prepared using 1-iodo-4-nitrobenzene (1 equiv, 0.50 mmol, 125 mg) and 2-iodobutane (5 equiv, 2.5 mmol, 286 μL). The crude product was purified by flash chromatography using hexanes as an eluent to afford the title compound (**3i**) as viscous brown oil (81 mg, 59%). **^{1}H NMR** (400 MHz, $CDCl_3$): δ 7.38 (d, J = 8.0 Hz, 2 H), 6.34 (d, J = 8.0 Hz, 2 H), 3.47 (br s, 1 H), 3.33 (sex, J = 5.6 Hz, 1 H), 1.61-1.51 (m, 1 H), 1.49-1.40 (m, 1 H), 1.14 (d, J = 6.1 Hz, 3 H), 0.93 (t, J = 7.4 Hz, 3 H). **^{13}C NMR** (100 MHz, $CDCl_3$): δ 147.4, 137.9, 115.4, 77.0, 49.8, 29.1, 20.2, 10.4. **HRMS** (ESI): Calcd for $C_{10}H_{15}IN$ [M+H]: 276.0249; Found: 276.0240.

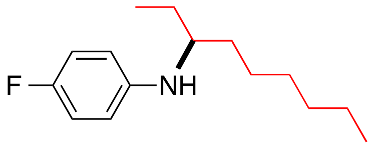

4-Fluoro-*N*-(nonan-3-yl)aniline (3j). Following the general procedure A, the title compound was prepared using 1-fluoro-4-nitrobenzene (71 mg) and 3-iodononane (381 mg). The crude product was purified by flash chromatography using hexanes as an eluent to afford the title compound (**3j**) as brown oil (100 mg, 84%). **^{1}H NMR** (400 MHz, $CDCl_3$): δ 6.85 (dd, $^3J_{HF}$ = 9.6 Hz, $^3J_{HH}$ = 8.5 Hz, 2 H), 6.49-6.46 (m, 2 H), 3.26 (br s, 1 H), 3.18 (qu, J = 6.0 Hz, 1 H), 1.59-1.23 (ovrlp, 12 H), 0.92-0.85 (ovrlp, 6 H). **^{13}C NMR** (100 MHz, $CDCl_3$): δ 155.4 (d, $^1J_{CF}$ = 232.5 Hz), 144.7 (d, $^4J_{CF}$ = 2.2 Hz), 115.7 (d, $^2J_{CF}$ = 22.0 Hz), 113.8 (d, $^3J_{CF}$ = 7.2 Hz), 55.0, 34.4, 32.0, 29.6, 27.3, 26.1, 22.8, 14.2, 10.1. **HRMS** (ESI): Calcd for $C_{15}H_{25}FN$ [M+H]: 238.1966; Found: 238.1968.

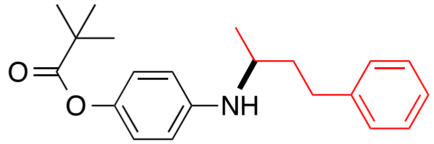

4-((4-phenylbutan-2-yl)amino)phenyl pivalate (3k). Following the general procedure A, the title compound was prepared using 4-nitrophenyl pivalate (112 mg) and (3-iodobutyl)benzene (390 mg). The crude product was purified by flash chromatography using hexanes and then hexanes/EtOAc (20:1) as eluents to afford the title compound (**3k**) as viscous brown oil (110 mg, 67%). **^{1}H NMR** (400 MHz, $CDCl_3$): δ 7.26 (d, J = 7.1 Hz, 2 H), 7.20-7.15 (ovrlp, 3 H), 6.82 (d, J = 8.4 Hz, 2 H), 6.46 (d, J = 8.4 Hz, 2 H), 3.47-3.29 (ovrlp, 2 H), 2.70 (t, J = 7.1 Hz, 2 H), 1.89-1.80 (m, 1 H), 1.77-1.68 (m, 1 H), 1.32 (s, 9 H), 1.18 (d, J = 5.9 Hz, 3 H). **^{13}C NMR** (100 MHz, $CDCl_3$): δ 177.8, 145.3, 142.1, 142.0, 128.52, 128.47, 125.9, 122.0, 113.5, 48.3, 39.0, 38.8, 32.5, 27.3, 20.8. **GCMS:** $[M]^+$ = 325 was detected which corresponds to $C_{21}H_{27}NO_2$.

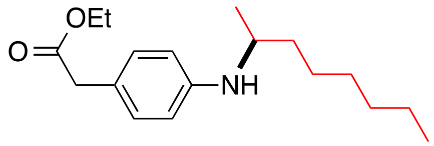

Ethyl 2-(4-(Octan-2-ylamino)phenyl)acetate (3l). Following the general procedure A, the title compound was prepared using ethyl 2-(4-nitrophenyl)acetate (105 mg) and 2-iodooctane (360 mg). The crude product was purified by flash chromatography using hexanes and then hexanes/EtOAc (20:1) as eluents to afford the title compound (**3l**) as viscous brown oil (88 mg, 60%). **^{1}H NMR** (400 MHz, $CDCl_3$): δ 7.04 (d, J = 7.8 Hz, 2 H), 6.50 (d, J = 7.7 Hz, 2 H), 4.10 (q, J = 7.1 Hz, 2 H), 3.45 (s, 2 H), 3.43-3.35 (ovrlp, 2 H), 1.57-1.47 (m, 1 H), 1.42-1.24 (ovrlp, 9 H), 1.22 (t, J = 6.7 Hz, 3 H), 1.13 (d, J = 5.8 Hz, 3 H), 0.88 (t, J = 7.0 Hz, 3 H). **^{13}C NMR** (100 MHz, $CDCl_3$): δ 172.4, 146.8, 130.1, 122.1, 113.2, 60.7, 48.6, 40.6, 37.3, 31.9, 29.4, 26.2, 22.7, 20.9, 14.3, 14.2. **HRMS** (ESI): Calcd for $C_{18}H_{30}NO_2$ [M+H]: 292.2277; Found: 292.2277.

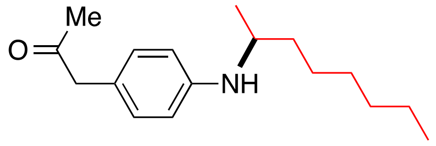

1-(4-(Octan-2-ylamino)phenyl)propan-2-one (3m) (Note: the product was not air-stable). Following the general procedure A, the title compound was prepared using 1-(4-nitrophenyl)propan-2-one (90 mg) and 2-iodooctane (360 mg). The crude product was purified by flash chromatography using hexanes and then hexanes/EtOAc (20:1) as eluents to afford the title compound (**3m**) as viscous brown oil (60 mg, 46%). **^{1}H NMR** (400 MHz, $CDCl_3$): δ 6.98 (d, *J* = 7.2 Hz, 2 H), 6.53 (d, *J* = 7.3 Hz, 2 H), 3.54 (s, 2 H), 3.48-3.31 (ovrlp, 2 H), 2.11 (s, 3 H), 1.59-1.50 (m, 1 H), 1.43-1.23 (ovrlp, 9 H), 1.16 (d, *J* = 5.0 Hz, 3 H), 0.88 (t, *J* = 6.0 Hz, 3 H). **^{13}C NMR** (100 MHz, $CDCl_3$): δ 207.7, 146.9, 130.3, 122.2, 113.4, 50.4, 48.6, 37.3, 31.9, 29.5, 29.0, 26.2, 22.7, 20.9, 14.2. **GCMS:** $[M]^+$ = 261 was detected which corresponds to $C_{17}H_{27}NO$.

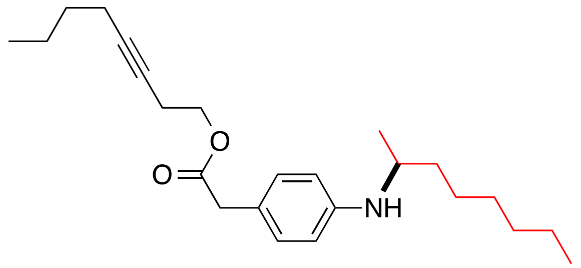

Oct-3-yn-1-yl 2-(4-(octan-2-ylamino)phenyl)acetate (3n). Following the general procedure A, the title compound was prepared using oct-3-yn-1-yl 2-(4-nitrophenyl)acetate (**S1**, 145 mg) and 2-iodooctane (360 mg). The crude product was purified by flash chromatography using hexanes and then hexanes/EtOAc (20:1) as eluents to afford the title compound (**3n**) as viscous brown oil (102 mg, 55%). **^{1}H NMR** (400 MHz, $CDCl_3$): δ 7.07 (d, *J* = 7.9 Hz, 2 H), 6.51 (d, *J* = 8.0 Hz, 2 H), 4.13 (t, *J* = 7.0 Hz, 2 H), 3.49 (s, 2 H), 3.50-3.24 (ovrlp, 2 H), 2.47 (t, *J* = 6.4 Hz, 2 H), 2.13 (t, *J* = 6.2 Hz, 2 H), 1.60-1.51 (m, 1 H), 1.47-1.24 (ovrlp, 13 H), 1.15 (d, *J* = 6.0 Hz, 3 H), 0.92-0.86 (ovrlp, 6 H). **^{13}C NMR** (100 MHz, $CDCl_3$): δ 172.2, 146.9, 130.2, 121.9, 113.2, 82.1, 75.6, 63.2, 48.7, 40.5, 37.4, 32.0, 31.1, 29.5, 26.3, 22.8, 22.0, 20.9, 19.4, 18.5, 14.2, 13.8. **HRMS** (ESI): Calcd for $C_{24}H_{38}NO_2$ [M+H]: 372.2903; Found: 372.2899.

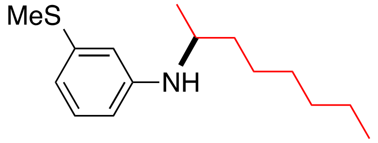

3-(Methylthio)-*N*-(octan-2-yl)aniline (3o). Following the general procedure A, the title compound was prepared using methyl(3-nitrophenyl)sulfane (85 mg) and 2-iodooctane (360 mg). The crude product was purified by flash chromatography using hexanes as an eluent to afford the title compound (**3o**) as pale-brown oil (92 mg, 73%). **^{1}H NMR** (400 MHz, $CDCl_3$): δ 7.06 (t, *J* = 7.9 Hz, 1 H), 6.55 (d,

J = 7.6 Hz, 1 H), 6.46 (s, 1 H), 6.34 (d, *J* = 8.0 Hz, 1 H), 3.49-3.36 (ovrlp, 2 H), 2.44 (s, 3 H), 1.58-1.50 (m, 1 H), 1.45-1.23 (ovrlp, 9 H), 1.15 (d, *J* = 4.2 Hz, 3 H), 0.88 (t, *J* = 6.6 Hz, 3 H). **^{13}C NMR** (100 MHz, $CDCl_3$): δ 148.2, 139.2, 129.7, 115.0, 111.1, 110.3, 48.5, 37.3, 31.9, 29.5, 26.2, 22.7, 20.9, 15.9, 14.2. **HRMS** (ESI): Calcd for $C_{15}H_{26}NS$ [M+H]: 252.1786; Found: 252.1784.

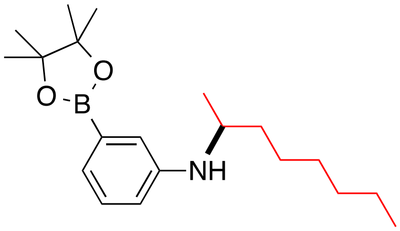

***N*-(Octan-2-yl)-3-(4,4,5,5-tetramethyl-1,3,2-dioxaborolan-2-yl)aniline (3p).** Following the general procedure A, the title compound was prepared using Zn (3 equiv, 1.05 mmol, 69 mg), $FeCl_2$•$4H_2O$ (20 mol %, 0.070 mmol, 14 mg), 4,4,5,5-tetramethyl-2-(3-nitrophenyl)-1,3,2-dioxaborolane (1 equiv, 0.35 mmol, 87 mg), 2-iodooctane (5 equiv, 1.75 mmol, 420 mg), NMP (0.70 mL), and TMSCl (2 equiv, 0.70 mmol, 89 *μ*L). The crude product was purified by flash chromatography using hexanes hexanes and then hexanes/EtOAc (10:1) as eluents to afford the title compound (**3p**) as viscous brown oil (47 mg, 40%). **^{1}H NMR** (400 MHz, $CDCl_3$): δ 7.17 (t, *J* = 7.6 Hz, 1 H), 7.10 (d, *J* = 7.1 Hz, 1 H), 7.02 (s, 1 H), 6.67 (d, *J* = 7.7 Hz, 1 H), 3.49 (sex, *J* = 5.9 Hz, 1 H), 3.39 (br s, 1 H), 1.57-1.48 (m, 1 H), 1.44-1.24 (ovrlp, 21 H), 1.15 (d, *J* = 6.1 Hz, 3 H), 0.88 (t, *J* = 6.7 Hz, 3 H). **^{13}C NMR** (100 MHz, $CDCl_3$): δ 147.3, 128.9, 123.3, 119.9, 115.7, 83.7, 48.5, 37.5, 32.0, 29.5, 26.2, 25.0, 22.8, 21.0, 14.2 (the *ipso*-carbon of C-B bonds was not observed due to quadruple relaxation). **HRMS** (ESI): Calcd for $C_{20}H_{35}BNO_2$ [M+H]: 332.2755; Found: 332.2760.

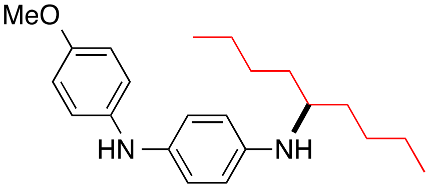

***N*^{1}-(4-Methoxyphenyl)-*N*^{4}-(nonan-5-yl)benzene-1,4-diamine (3q).** Following the general procedure A, the title compound was prepared using 4-methoxy-*N*-(4-nitrophenyl)aniline (122 mg) and 5-iodononane (384 mg). The crude product was purified by flash chromatography using hexanes and then hexanes and then hexanes/EtOAc (10:1) as eluents to afford the title compound (**3q**) as a deep brown solid (80 mg, 47%). **^{1}H NMR** (400 MHz, $CDCl_3$): δ 6.95-6.72 (ovrlp, 6 H), 6.52 (br s, 2 H), 5.15 (br s, 1 H), 3.76 (s, 3 H), 3.24 (br ovrlp, 2 H), 1.54-1.40 (m, 4 H), 1.37-1.25 (ovrlp, 8 H), 0.89 (t, *J* = 5.9 Hz, 6 H). **^{13}C NMR** (100 MHz, $CDCl_3$): δ 153.5, 143.9, 139.7, 134.0, 122.2, 117.9, 114.8, 114.1, 55.8, 53.7, 34.8, 28.3, 23.0. 14.3. **HRMS** (ESI): Calcd for $C_{22}H_{33}N_2O$ [M+H]: 341.2587; Found: 341.2589.

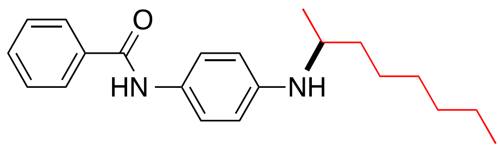

***N*-(4-(Octan-2-ylamino)phenyl)benzamide (3r).** Following the general procedure A, the title compound was prepared using *N*-(4-nitrophenyl)benzamide (121 mg) and 2-iodooctane (360 mg). The crude product was purified by flash chromatography using hexanes and then hexanes/EtOAc (8:1) as eluents to afford the title compound (**3r**) as brown solid (90 mg, 47%). **^{1}H NMR** (400 MHz, $CDCl_3$): δ 7.85 (d, *J* = 7.3 Hz, 2 H), 7.66 (br s, 1 H), 7.52 (t, *J* = 6.6 Hz, 1 H), 7.46 (t, *J* = 7.2 Hz, 2 H), 7.39 (d, *J* = 8.0 Hz, 2 H), 6.58 (d, *J* = 8.0 Hz, 2 H), 3.78-2.98 (ovrlp, 2 H), 1.63-1.51 (m, 1 H), 1.45-1.24 (ovrlp, 9 H), 1.17 (d, *J* = 6.1 Hz, 3 H), 0.88 (t, *J* = 6.5 Hz, 3 H). **^{13}C NMR** (100 MHz, $CDCl_3$): δ 165.6, 145.2, 135.5, 131.6, 128.8, 127.8, 127.1, 122.6, 133.6, 49.0, 37.3, 32.0, 29.5, 26.2, 22.8, 20.9, 14.2. **HRMS** (ESI): Calcd for $C_{21}H_{29}N_2O$ [M+H]: 325.2280; Found: 325.2279.

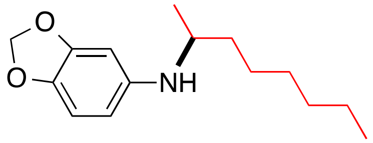

***N*-(Octan-2-yl)benzo[*d*][1,3]dioxol-5-amine (3s).** Following the general procedure A, the title compound was prepared using 5-nitrobenzo[*d*][1,3]dioxole (84 mg) and 2-iodooctane (360 mg). The crude product was purified by flash chromatography using hexanes and then hexanes/EtOAc (20:1) as eluents to afford the title compound (**3s**) as viscous brown oil (91 mg, 73%). **^{1}H NMR** (400 MHz, $CDCl_3$): δ 6.63 (d, *J* = 8.3 Hz, 1 H), 6.21 (s, 1 H), 6.00 (d, *J* = 8.2 Hz, 1 H), 5.83 (s, 2 H), 3.32 (sex, *J* = 5.8 Hz, 1 H), 3.18 (br s, 1 H), 1.57-1.49 (m, 1 H), 1.38-1.24 (ovrlp, 9 H), 1.13 (d, *J* = 6.2 Hz, 3 H), 0.88 (t, *J* = 6.5 Hz, 3 H). **^{13}C NMR** (100 MHz, $CDCl_3$): δ 148.5, 143.6, 139.3, 108.8, 105.2, 100.6, 96.4, 49.7, 37.3, 32.0, 29.5, 26.2, 22.7, 20.9, 14.2. **HRMS** (ESI): Calcd for $C_{15}H_{24}NO_2$ [M+H]: 250.1807; Found: 250.1801.

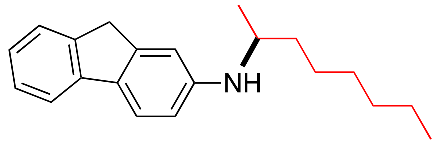

***N*-(Octan-2-yl)-9*H*-fluoren-2-amine (3t).** Following the general procedure A, the title compound was prepared using 2-nitro-9*H*-fluorene (106 mg) and 2-iodooctane (360 mg). The crude product was purified by flash chromatography using hexanes as an eluent to afford the title compound (**3t**) as viscous deep brown oil (77 mg, 52%). **^{1}H NMR** (400 MHz, $CDCl_3$): δ 7.58 (d, *J* = 7.5 Hz, 1 H), 7.53 (d, *J* = 8.2 Hz, 1 H), 7.42 (d, *J* = 7.4 Hz, 1 H), 7.27 (t, *J* = 7.4 Hz, 1 H), 7.13 (t, *J* = 7.3 Hz, 1 H), 6.73 (s, 1 H), 6.56 (d, *J* = 8.4 Hz, 1 H), 3.78 (s, 2 H), 3.52-3.42 (ovrlp, 2 H), 1.61-1.51 (m, 1 H), 1.46-1.25 (ovrlp, 9 H), 1.18 (d, *J* = 5.5 Hz, 3 H), 0.88 (t, *J* = 6.6 Hz, 3 H). **^{13}C NMR** (100 MHz, $CDCl_3$): δ 147.4, 145.3, 142.6, 142.2, 131.5, 126.7, 124.74, 124.71, 120.8, 118.4, 112.4, 109.5, 48.9, 37.4, 37.1, 32.0, 29.5, 26.3, 22.8, 21.0, 14.2. **HRMS** (ESI): Calcd for $C_{21}H_{28}N$ [M+H]: 294.2222; Found: 294.2220.

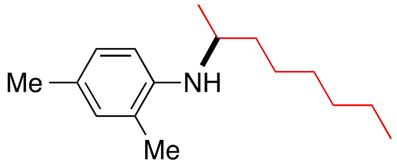

2,4-Dimethyl-*N*-(octan-2-yl)aniline (3u). Following the general procedure A, the title compound was prepared using 2,4-dimethyl-1-nitrobenzene (76 mg) and 2-iodooctane (360 mg). The crude product was purified by flash chromatography using hexanes as an eluent to afford the title compound (**3u**) as viscous brown oil (70 mg, 60%). ^{1}**H NMR** (400 MHz, $CDCl_3$): δ 6.88 (d, *J* = 8.3 Hz, 1 H), 6.84 (s, 1 H), 6.48 (d, *J* = 8.0 Hz, 1 H), 3.42 (sex, *J* = 5.6 Hz, 1 H), 3.08 (br s, 1 H), 2.17 (s, 3 H), 2.06 (s, 3 H), 1.59-1.51 (m, 1 H), 1.43-1.21 (ovrlp, 9 H), 1.15 (d, *J* = 6.0 Hz, 3 H), 0.85 (t, *J* = 5.7 Hz, 3 H). ^{13}**C NMR** (100 MHz, $CDCl_3$): δ 143.4, 131.2, 127.4, 125.4, 122.0, 110.5, 48.7, 37.5, 32.0, 29.5, 26.3, 22.8, 21.2, 20.4, 17.7, 14.2. **HRMS** (ESI): Calcd for $C_{16}H_{28}N$ [M+H]: 234.2222; Found: 234.2227.

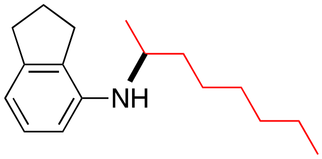

***N*-(Octan-2-yl)-2,3-dihydro-1*H*-inden-4-amine (3v).** Following the general procedure A, the title compound was prepared using 4-nitro-2,3-dihydro-1*H*-indene (82 mg) and 2-iodooctane (360 mg). The crude product was purified by flash chromatography using hexanes as an eluent to afford the title compound (**3v**) as deep brown oil (104 mg, 85%). ^{1}**H NMR** (400 MHz, $CDCl_3$): δ 7.04 (t, *J* = 7.6 Hz, 1 H), 6.60 (d, *J* = 7.3 Hz, 1 H), 6.41 (d, *J* = 7.9 Hz, 1 H), 3.48 (sex, *J* = 6.0 Hz, 1 H), 3.19 (br s, 1 H), 2.90 (t, *J* = 7.5 Hz, 2 H), 2.64 (t, *J* = 7.3 Hz, 2 H), 2.08 (qu, *J* = 7.4 Hz, 2 H), 1.63-1.55 (m, 1 H), 1.48-1.24 (ovrlp, 9 H), 1.18 (d, *J* = 6.1 Hz, 3 H), 0.88 (t, *J* = 6.9 Hz, 3 H). ^{13}**C NMR** (100 MHz, $CDCl_3$): δ 145.0, 144.0, 128.1, 127.7, 112.9, 107.7, 48.5, 37.5, 33.6, 32.0, 29.54, 29.52, 26.3, 24.6, 22.8, 21.2, 14.2. **HRMS** (ESI): Calcd for $C_{17}H_{28}N$ [M+H]: 246.2222; Found: 246.2222.

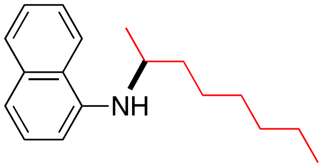

***N*-(Octan-2-yl)naphthalen-1-amine (3w).** Following the general procedure A, the title compound was prepared using 1-nitronaphthalene (87 mg) and 2-iodooctane (360 mg). The crude product was purified by flash chromatography using hexanes as an eluent to afford the title compound (**3v**) as viscous brown oil (96 mg, 75%). ^{1}**H NMR** (400 MHz, $CDCl_3$): δ 7.76-7.73 (ovrlp, 2 H), 7.42-7.35 (ovrlp, 2 H), 7.32 (t, *J* = 7.8 Hz, 1 H), 7.17 (d, *J* = 8.2 Hz, 1 H), 6.58 (d, *J* = 7.6 Hz, 1 H), 4.16 (br s, 1 H), 3.62 (sex, *J* = 6.2 Hz, 1 H), 1.73-1.65 (m, 1 H), 1.57-1.47 (m, 1 H), 1.46-1.37 (ovrlp, 2 H), 1.35-1.27 (ovrlp, 6 H), 1.26 (d, *J* = 6.3 Hz, 3 H), 0.88 (t, *J* = 7.0 Hz, 3 H). ^{13}**C NMR** (100 MHz, $CDCl_3$): δ 142.8, 134.7, 128.8, 126.8, 125.7, 124.6, 123.5, 119.9, 116.6, 104.6, 48.7, 37.3, 32.0, 29.6, 26.4, 22.8,

20.8, 14.2. **HRMS** (ESI): Calcd for $C_{18}H_{26}N$ [M+H]: 256.2065; Found: 256.2066.

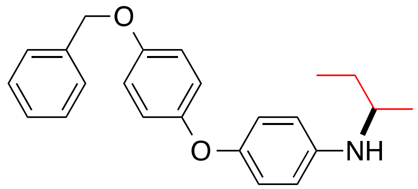

4-(4-(Benzyloxy)phenoxy)-*N*-(*sec*-butyl)aniline (3x). Following the general procedure A, the title compound was prepared using Zn (4.5 equiv, 2.25 mmol, 147 mg), $FeCl_2$•$4H_2O$ (30 mol %, 0.15 mmol, 30 mg), 1-(benzyloxy)-4-(4-nitrophenoxy)benzene (1 equiv, 0.50 mmol, 161 mg), and 2-iodobutane (6 equiv, 3.0 mmol, 347 μL). The crude product was purified by flash chromatography using hexanes and then hexanes/EtOAc (20:1) as eluents to afford the title compound (**3v**) as viscous brown oil (91 mg, 53 %). **^{1}H NMR** (400 MHz, $CDCl_3$): δ 7.41-7.33 (ovrlp, 4 H), 7.29 (t, *J* = 7.3 Hz, 1 H), 6.92-6.85 (ovrlp, 4 H), 6.82 (d, *J* = 8.4 Hz, 2 H), 6.52 (d, *J* = 8.2 Hz, 2 H), 4.99 (s, 2 H), 3.40-3.11 (ovrlp, 2 H), 1.61-1.52 (m, 1 H), 1.49-1.40 (m, 1 H), 1.14 (d, *J* = 6.2 Hz, 3 H), 0.93 29 (t, *J* = 7.5 Hz, 3 H). **^{13}C NMR** (100 MHz, $CDCl_3$): δ 154.2, 152.8, 148.6, 144.1, 137.3, 128.7, 128.0, 127.6, 120.5, 118.8, 115.9, 114.3, 70.7, 50.6, 29.8, 20.4, 10.5. **HRMS** (ESI): Calcd for $C_{23}H_{26}NO_2$ [M+H]: 348.1958; Found: 348.1961.

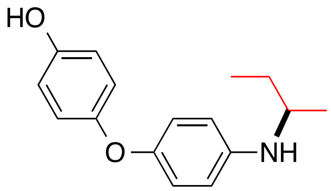

4-(4-(*sec*-butylamino)phenoxy)phenol (PD0084430, 3y). An oven-dried 250 mL round-bottom flask equipped with a Teflon-coated magnetic stir bar was charged with 4-(4-(benzyloxy)phenoxy)-*N*-(*sec*-butyl)aniline (**3x**) (1 equiv, 0.40 mmol, 139 mg), palladium on carbon (5 weight %, 10 mol % Pd, 0.040 mmol Pd, 86 mg), and methanol solvent (30 mL). The flask was then stopped with an adaptor connected with a hydrogen-filled balloon. The resulting mixture was stirred at room temperature for 24 h. After the reaction, the crude product was washed with CH_2Cl_2 (~30 mL) and water (~100 mL). The aqueous fraction was further extracted with CH_2Cl_2 (2 x ~10 mL). The combined organic fraction was concentrated *in vacuo* with the aid of a rotary evaporator. The crude product residue was purified by column chromatography using hexanes/EtOAc (10:1) as an eluent to afford the title compound (**3y**) as off-white solid (87 mg, 84%). **^{1}H NMR** (400 MHz, $CDCl_3$): δ 6.85 (d, *J* = 8.0 Hz, 2 H), 6.78 (d, *J* = 8.0 Hz, 2 H), 6.63 (d, *J* = 8.2 Hz, 2 H), 6.59 (d, *J* = 8.1 Hz, 2 H), 4.49 (br ovrlp, 2 H), 3.35 (sex, *J* = 5.7 Hz, 1 H), 1.69-1.55 (m, 1 H), 1.49-1.38 (m, 1 H), 1.17 (d, *J* = 5.8 Hz, 3 H), 0.94 (d, *J* = 7.0 Hz, 3 H). **^{13}C NMR** (100 MHz, $CDCl_3$): δ 152.4, 151.0, 149.3, 143.6, 120.6, 118.9, 116.2, 115.3, 51.1, 29.6, 20.2, 10.4. **HRMS** (ESI): Calcd for $C_{16}H_{20}NO_2$ [M+H]: 258.1489; Found: 258.1498.

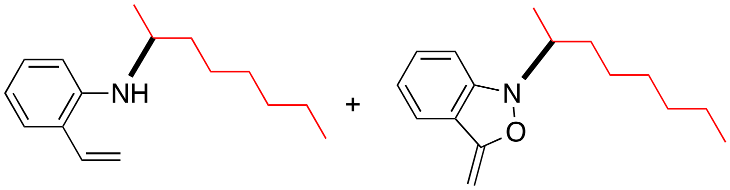

3z **3z'**

***N*-(octan-2-yl)-2-vinylaniline (3z) and 3-methylene-1-(octan-2-yl)-1,3-dihydrobenzo[*c*]isoxazole (3z').** Following the general procedure A, the title compound was prepared using 2-vinylnitrobenzene (75 mg) and 2-iodooctane (360 mg). The crude product was purified by flash chromatography using hexanes as an eluent to afford the an separable mixture of **3z** and **3z'** as brown oil (55 mg, total yield = 47%; **3z** ~ 36 mg, 31%; **3z'** ~ 19 mg, 16%) The ratio of **3z** to **3z'** was determined by ^{1}H NMR spectroscopy. **^{1}H NMR of an inseparable mixture of 3z and 3z'** (400 MHz, $CDCl_3$): δ 7.57 (d, *J* = 7.9 Hz, 0.5 H), 7.40 (d, *J* = 8.2 Hz, 0.5 H), 7.24-7.19 (ovrlp, 2 H), 7.15 (t, *J* = 7.8 Hz, 1 H), 7.08 (t, *J* = 7.8 Hz, 0.5 H), 6.75-6.70 (m, 1 H), 6.68-6.60 (ovrlp, 2 H), 6.33 (d, *J* = 3.2 Hz, 0.5 H), 5.57 (dd, *J* = 17.3 Hz, *J* = 1.5 Hz, 1 H), 5.28 (d, *J* = 11.0 Hz, *J* = 1.5 Hz, 1 H), 4.37 (sex, *J* = 6.1 Hz, 0.5 H), 3.60 (br s, 1 H), 3.47 (sex, *J* = 6.0 Hz, 1 H), 1.64-1.23 (ovrlp, ~17 H), 1.18 (d, *J* = 6.2 Hz, 3 H), 0.90-0.86 (ovrlp, 4.5 H). **^{13}C NMR of an inseparable mixture of 3z and 3z'** (100 MHz, $CDCl_3$): δ 144.8, 133.3, 129.0, 127.8, 125.0, 124.3, 124.2, 122.2, 121.1, 119.8, 116.7, 116.1, 111.2, 108.8, 97.4, 84.1, 48.6, 37.4, 35.3, 32.0, 31.9, 29.5, 26.3, 25.6, 22.78, 22.75, 21.0, 19.4, 14.2. **GCMS of 3z:** $[M]^+$ = 231 was detected which corresponds to $C_{16}H_{25}N$. **HRMS of 3z'**(ESI): Calcd for $C_{16}H_{24}NO$ [M+H]: 246.1858; Found: 246.1863.

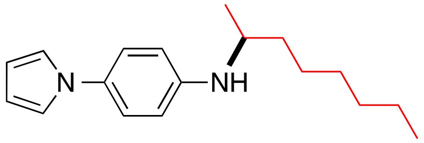

***N*-(Octan-2-yl)-4-(1*H*-pyrrol-1-yl)aniline (4a).** Following the general procedure A, the title compound was prepared using 1-(4-nitrophenyl)-1*H*-pyrrole (94 mg) and 2-iodooctane (360 mg). The crude product was purified by flash chromatography using hexanes and then hexanes/EtOAc (10:1) as eluents to afford the title compound (**4a**) as pale-brown oil (78 mg, 58%). **^{1}H NMR** (400 MHz, $CDCl_3$): δ 7.17 (d, *J* = 8.1 Hz, 2 H), 6.94 (s, 2 H), 6.57 (d, *J* = 8.2 Hz, 2 H), 6.28 (s, 2 H), 3.51-3.33 (ovrlp, 2 H), 1.62-1.52 (ovrlp, 1 H), 1.45-1.25 (ovrlp, 9 H), 1.17 (d, *J* = 5.6 Hz, 3 H), 0.88 (t, *J* = 6.6 Hz, 3 H). **^{13}C NMR** (100 MHz, $CDCl_3$): δ 146.1, 131.5, 122.7, 119.9, 113.4, 109.3, 48.9, 37.3, 32.0, 29.5, 26.2, 22.7, 20.9, 14.2. **HRMS** (ESI): Calcd for $C_{18}H_{27}N_2$ [M+H]: 271.2174; Found: 271.2180.

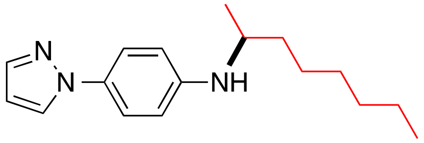

***N*-(Octan-2-yl)-4-(1*H*-pyrazol-1-yl)aniline (4b).** Following the general procedure A, the title compound was prepared using 1-(4-nitrophenyl)-1*H*-pyrazole (95 mg) and 2-iodooctane (360 mg). The crude product was purified by flash chromatography using hexanes and then hexanes/EtOAc (6:1)

as eluents to afford the title compound (**4b**) as brown oil (114 mg, 84%). **^{1}H NMR** (400 MHz, $CDCl_3$): δ 7.75 (s, 1 H), 7.65 (s, 1 H), 7.43 (d, *J* = 7.9 Hz, 2 H), 6.60 (d, *J* = 8.0 Hz, 2 H), 6.39 (s, 1 H), 3.54 (br s, 1 H), 3.46 (sex, *J* = 5.7 Hz, 1 H), 1.60-1.52 (m, 1 H), 1.46-1.25 (ovrlp, 9 H), 1.17 (d, *J* = 5.7 Hz, 3 H), 0.88 (t, *J* = 6.2 Hz, 3 H). **^{13}C NMR** (100 MHz, $CDCl_3$): δ 146.7, 140.1, 131.0, 126.7, 121.3, 113.3, 106.7, 48.8, 37.2, 31.9, 29.4, 26.2, 22.7, 20.8, 14.2. **HRMS** (ESI): Calcd for $C_{17}H_{26}N_3$ [M+H]: 272.2127; Found: 272.2128.

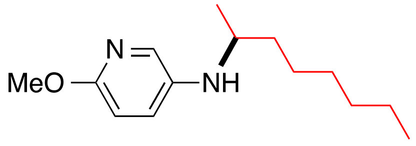

6-Methoxy-*N*-(octan-2-yl)pyridin-3-amine (4c). Following the general procedure A, the title compound was prepared using 2-methoxy-5-nitropyridine (77 mg) and 2-iodooctane (360 mg). The crude product was purified by flash chromatography using hexanes and then hexanes/EtOAc (6:1) as eluents to afford the title compound (**4c**) as viscous deep-brown oil (83 mg, 70%). **^{1}H NMR** (400 MHz, $CDCl_3$): δ 7.55 (s, 1 H), 6.95 (d, *J* = 7.1 Hz, 1 H), 6.61 (d, *J* = 8.0 Hz, 1 H), 3.86 (s, 3 H), 3.34 (sex, *J* = 5.4 Hz, 1 H), 3.04 (br s, 1 H), 1.60-1.51 (m, 1 H), 1.44-1.22 (ovrlp, 9 H), 1.15 (d, *J* = 5.0 Hz, 3 H), 0.88 (t, *J* = 6.0 Hz, 3 H). **^{13}C NMR** (100 MHz, $CDCl_3$): δ 157.3, 138.7, 131.3, 126.5, 110.9, 53.4, 49.9, 37.2, 31.9, 29.5, 26.2, 22.7, 20.8, 14.2. **HRMS** (ESI): Calcd for $C_{14}H_{25}N_2O$ [M+H]: 237.1967; Found: 237.1964.

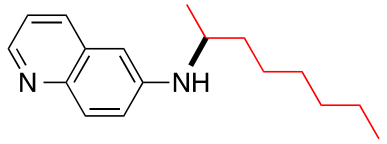

***N*-(Octan-2-yl)quinolin-6-amine (4d).** Following the general procedure A, the title compound was prepared using Zn (3.5 equiv, 1.75 mmol, 114.5 mg), 6-nitroquinoline (1 equiv, 0.50 mmol, 87 mg), $FeCl_2$•$4H_2O$ (40 mol %, 0.20 mmol, 40 mg), and 2-iodooctane (5 equiv, 2.5 mmol, 600 mg). The crude product was purified by flash chromatography using hexanes and then hexanes/EtOAc (6:1) as eluents to afford the title compound (**4d**) as viscous deep-brown oil (72 mg, 56%). **^{1}H NMR** (400 MHz, $CDCl_3$): δ 8.58 (s, 1 H), 7.89-7.84 (ovrlp, 2 H), 7.27-7.20 (m, 1 H), 7.04 (d, *J* = 8.5 Hz, 1 H), 6.65 (s, 1 H), 3.83 (br s, 1 H), 3.62-3.51 (m, 1 H), 1.66-1.57 (m, 1 H), 1.52-1.26 (ovrlp, 9 H), 1.23 (d, *J* = 5.3 Hz, 3 H), 0.88 (t, *J* = 7.2 Hz, 3 H). **^{13}C NMR** (100 MHz, $CDCl_3$): δ 145.9, 145.6, 143.0, 133.7, 130.4, 121.7, 121.4, 103.0, 48.6, 37.1, 31.9, 29.4, 26.2, 22.7, 20.6, 14.2. **HRMS** (ESI): Calcd for $C_{17}H_{25}N_2$ [M+H]: 257.2018; Found: 257.2010.

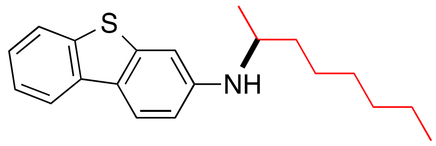

***N*-(Octan-2-yl)dibenzo[*b*,*d*]thiophen-3-amine (4e).** Following the general procedure A, the title

compound was prepared using 3-nitrodibenzo[*b,d*]thiophene (115 mg) and 2-iodooctane (360 mg). The crude product was purified by flash chromatography using hexanes as an eluent to afford the title compound (**4e**) as viscous deep-brown oil (99 mg, 64%). **^{1}H NMR** (400 MHz, $CDCl_3$): δ 8.05-0.03 (m, 1 H), 7.80-7.78 (m, 1 H), 7.50 (d, *J* = 8.6 Hz, 1 H), 7.41-7.37 (ovrlp, 2 H), 7.30 (s, 1 H), 6.77 (d, *J* = 8.6 Hz, 1 H), 3.68-3.25 (ovrlp, 2 H), 1.67-1.53 (m, 1 H), 1.52-1.26 (ovrlp, 9 H), 1.23 (d, *J* = 6.1 Hz, 3 H), 0.88 (t, *J* = 6.4 Hz, 3 H). **^{13}C NMR** (100 MHz, $CDCl_3$): δ 145.6, 140.6, 136.8, 135.7, 127.6, 126.4, 123.9, 123.3, 123.0, 121.5, 115.6, 104.4, 49.1, 37.3, 32.0, 29.5, 26.3, 22.8, 20.8, 14.2. **HRMS** (ESI): Calcd for $C_{20}H_{26}NS$ [M+H]: 312.1786; Found: 312.1783.

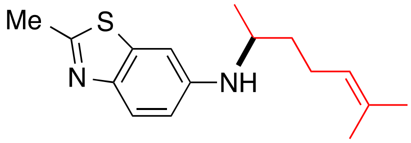

2-Methyl-*N*-(6-methylhept-5-en-2-yl)benzo[*d*]thiazol-6-amine (4f). Following the general procedure A, the title compound was prepared using 2-methyl-6-nitrobenzo[*d*]thiazole (97 mg) and 6-iodo-2-methylhept-2-ene (357 mg). The crude product was purified by flash chromatography using hexanes and then hexanes/EtOAc (10:1) as eluents to afford the title compound (**4f**) as viscous brown oil (98 mg, 71%). **^{1}H NMR** (400 MHz, $CDCl_3$): δ 7.68 (d, *J* = 8.7 Hz, 1 H), 6.90 (s, 1 H), 6.67 (d, *J* = 8.7 Hz, 1 H), 5.13 (t, *J* = 8.0 Hz, 1 H), 3.60 (br s, 1 H), 3.48 (sex, *J* = 6.1 Hz, 1 H), 2.73 (s, 3 H), 2.12-2.05 (m, 2 H), 1.70 (s, 3 H), 1.65-1.58 (ovrlp, 4 H), 1.55-1.45 (m, 1 H), 1.20 (d, *J* = 5.8 Hz, 3 H). **^{13}C NMR** (100 MHz, $CDCl_3$): δ 161.2, 145.58, 145.56, 137.7, 132.2, 123.9, 122.7, 114.3, 102.6, 48.6, 37.1, 25.8, 24.7, 20.8, 19.8, 17.8. **HRMS** (ESI): Calcd for $C_{16}H_{23}N_2S$ [M+H]: 275.1582; Found: 275.1590.

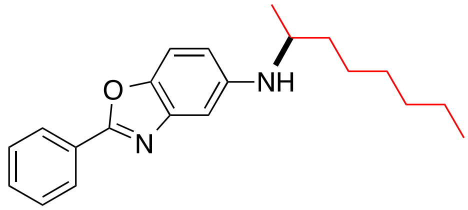

***N*-(Octan-2-yl)-2-phenylbenzo[*d*]oxazol-5-amine (4g).** Following the general procedure A, the title compound was prepared using Zn (3 equiv, 1.05 mmol, 69 mg), $FeCl_2$•$4H_2O$ (20 mol %, 0.070 mmol, 14 mg), 5-nitro-2-phenylbenzo[*d*]oxazole (1 equiv, 0.35 mmol, 84 mg), 2-iodooctane (3 equiv, 1.05 mmol, 252 mg), NMP (0.70 mL), and TMSCl (2 equiv, 0.70 mmol, 89 μL). The crude product was purified by flash chromatography using hexanes and then hexanes/EtOAc (20:1) as eluents to afford the title compound (**4g**) as viscous pale-brown oil (66 mg, 58%). **^{1}H NMR** (400 MHz, $CDCl_3$): δ 8.23-8.18 (m, 2 H), 7.52-7.44 (ovrlp, 3 H), 7.33 (d, *J* = 8.6 Hz, 1 H), 6.93 (s, 1 H), 6.60 (d, *J* = 8.6 Hz, 1 H), 3.62-3.32 (ovrlp, 2 H), 1.66-1.56 (m, 1 H), 1.47-1.24 (ovrlp, 9 H), 1.20 (d, *J* = 6.1 Hz, 3 H), 0.88 (t, *J* = 6.5 Hz, 3 H). **^{13}C NMR** (100 MHz, $CDCl_3$): δ 163.2, 145.8, 143.9, 143.4, 131.2, 128.9, 127.7, 127.5, 113.2, 110.8, 102.0, 49.5, 37.2, 32.0, 29.5, 26.3, 22.7, 20.7, 14.2. **HRMS** (ESI): Calcd for $C_{21}H_{27}N_2O$ [M+H]: 323.2118; Found: 323.2121.

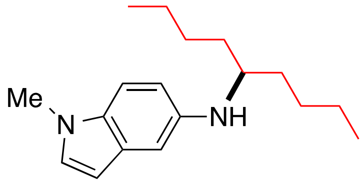

1-Methyl-*N*-(nonan-5-yl)-1*H*-indol-5-amine (4h). Following the general procedure A, the title compound was prepared using 1-methyl-5-nitro-1*H*-indole (88 mg) and 5-iodononane (384 mg). The crude product was purified by flash chromatography using hexanes and then hexanes/EtOAc (15:1) as eluents to afford the title compound (**4h**) as viscous deep-brown oil (95 mg, 70%). **^{1}H NMR** (400 MHz, $CDCl_3$): δ 7.11 (d, *J* = 8.6 Hz, 1 H), 6.93 (s, 1 H), 6.78 (s, 1 H), 6.61 (d, *J* = 8.6 Hz, 1 H), 6.29 (s, 1 H), 3.71 (s, 3 H), 3.32 (qu, *J* = 5.8 Hz, 1 H), 2.75 (br s, 1 H), 1.56-1.45 (m, 4 H), 1.42-1.26 (ovrlp, 8 H), 0.89 (t, *J* = 6.0 Hz, 6 H). **^{13}C NMR** (100 MHz, $CDCl_3$): δ 142.1, 131.2, 129.6, 128.9, 112.3, 109.9, 103.2, 99.7, 54.5, 34.7, 33.0, 28.3, 23.1, 14.3. **HRMS** (ESI): Calcd for $C_{18}H_{29}N_2$ [M+H]: 273.2325; Found: 273.2330.

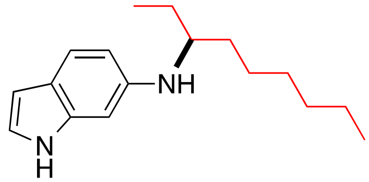

***N*-(Nonan-3-yl)-1*H*-indol-6-amine (4i).** Following the general procedure A, the title compound was prepared using 1-methyl-6-nitro-1*H*-indole (81 mg) and 3-iodononane (384 mg). The crude product was purified by flash chromatography using hexanes and then hexanes/EtOAc (10:1) as eluents to afford the title compound (**4i**) as viscous deep-brown solid (70 mg, 54%). **^{1}H NMR** (400 MHz, $CDCl_3$): δ 7.78 (br s, 1 H), 7.37 (d, *J* = 8.2 Hz, 1 H), 6.91 (s, 1 H), 6.49-6.46 (ovrlp, 2 H), 6.38 (s, 1 H), 3.32 (br s, 1 H), 3.27 (qu *J* = 5.8 Hz, 1 H), 1.65-1.44 (ovrlp, 4 H), 1.42-1.22 (ovrlp, 8 H), 0.93 (t, *J* = 7.4 Hz, 3 H), 0.87 (t, *J* = 6.5 Hz, 3 H). **^{13}C NMR** (100 MHz, $CDCl_3$): δ 144.7, 137.6, 121.5, 121.3, 120.0, 110.5, 102.5, 93.6, 55.1, 34.4, 32.0, 29.7, 27.2, 26.1, 22.8, 14.2, 10.2. **HRMS** (ESI): Calcd for $C_{17}H_{27}N_2$ [M+H]: 259.2174; Found: 259.2169.

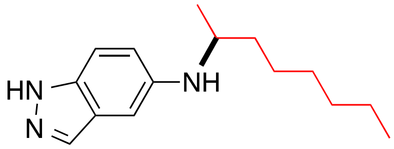

***N*-(Octan-2-yl)-1*H*-indazol-5-amine (4j).** Following the general procedure A, the title compound was prepared using 5-nitro-1*H*-indazole (82 mg) and 2-iodooctane (360 mg). The crude product was purified by flash chromatography using hexanes and then hexanes/EtOAc (6:1) as eluents to afford the title compound (**4j**) as viscous brown oil (53 mg, 43%). **^{1}H NMR** (400 MHz, $CDCl_3$): δ 10.1 (br s, 1 H), 7.89 (s, 1 H), 7.28 (d, *J* = 9.4 Hz, 1 H), 6.81-6.77 (ovrlp, 2 H), 4.43-2.65 (ovrlp, 2 H), 1.66-1.54 (m, 1 H), 1.49-1.25 (ovrlp, 9 H), 1.20 (d, *J* = 6.2 Hz, 3 H), 0.88 (t, *J* = 7.0 Hz, 3 H). **^{13}C NMR** (100 MHz, $CDCl_3$): δ 142.5, 135.1, 133.8, 124.5, 119.3, 110.6, 100.0, 49.7, 37.2, 32.0, 29.5, 26.3, 22.8, 20.7, 14.2. **HRMS** (ESI): Calcd for $C_{15}H_{24}N_3$ [M+H]: 246.1970; Found: 246.1976.

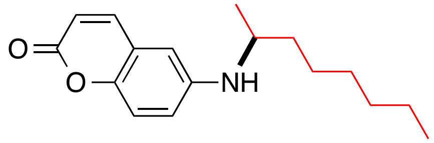

6-(Octan-2-ylamino)-2*H*-chromen-2-one (4k). Following the general procedure A, the title compound was prepared using 6-nitro-2*H*-chromen-2-one (96 mg) and 2-iodooctane (360 mg). The crude product was purified by flash chromatography using hexanes and then hexanes/EtOAc (20:1) as eluents to afford the title compound (**4k**) as low-melting, deep-yellow oil (78 mg, 57%). ^{1}**H NMR** (400 MHz, $CDCl_3$): δ 7.60 (d, J = 9.4 Hz, 1 H), 7.13 (d, J = 8.8 Hz, 1 H), 6.77 (d, J = 8.6 Hz, 1 H), 6.55 (s 1 H), 6.36 (d, J = 9.4 Hz, 1 H), 3.51 (br s, 1 H), 3.43 (sex, J = 5.6 Hz, 1 H), 1.62-1.52 (m, 1 H), 1.49-1.24 (ovrlp, 9 H), 1.19 (d, J = 5.6 Hz, 3 H), 0.88 (t, J = 6.6 Hz, 3 H).^{13}**C NMR** (100 MHz, $CDCl_3$): δ 161.5, 146.3, 144.7, 143.7, 119.5, 118.5, 117.6, 116.7, 108.6, 49.1, 37.1 31.9, 29.4, 26.1, 22.7, 20.6, 14.2. **HRMS** (ESI): Calcd for $C_{17}H_{24}NO_2$ [M+H]: 274.1807; Found: 274.1802.

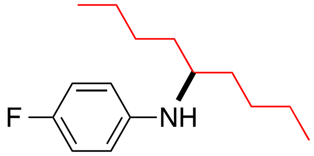

4-Fluoro-*N*-(nonan-5-yl)aniline (5a). Following the general procedure A, the title compound was prepared using 1-fluoro-4-nitrobenzene (71 mg) and 5-iodononane (381 mg). The crude product was purified by flash chromatography using hexanes as an eluent to afford the title compound (**5a**) as brown oil (93 mg, 78%). ^{1}**H NMR** (400 MHz, $CDCl_3$): 6.85 (dd, $^3J_{HF}$ = 9.4 Hz, $^3J_{HH}$ = 8.5 Hz, 2 H), 6.49-6.46 (m, 2 H), 3.29-3.19 (ovrlp, 2 H), 1.55-1.24 (ovrlp, 12 H), 0.88 (t, J = 6.4 Hz, 6 H). ^{13}**C NMR** (100 MHz, $CDCl_3$): δ 155.4 (d, $^1J_{CF}$ = 232.4 Hz), 144.7 (d, $^4J_{CF}$ = 1.8 Hz), 115.7 (d, $^2J_{CF}$ = 22.1 Hz), 113.8 (d, $^3J_{CF}$ = 7.2 Hz), 53.8, 34.7, 28.2, 23.0, 14.2. **HRMS** (ESI): Calcd for $C_{15}H_{25}FN$ [M+H]: 238.1966; Found: 238.1968.

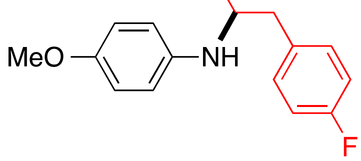

***N*-(1-(4-Fluorophenyl)propan-2-yl)-4-methoxyaniline (5b).** Following the general procedure A, the title compound was prepared using 1-methoxy-4-nitrobenzene (77 mg) and 1-fluoro-4-(2-iodopropyl)benzene (396 mg). The crude product was purified by flash chromatography using hexanes and then hexanes/EtOAc (25:1) as eluents to afford the title compound (**5b**) as viscous brown oil (106 mg, 82%). ^{1}**H NMR** (400 MHz, $CDCl_3$): δ 7.10 (dd, $^3J_{HH}$ = 8.2 Hz, $^4J_{HF}$ = 6.3 Hz, 2 H), 6.96 (dd, $^3J_{HF}$ = 9.6 Hz, $^3J_{HH}$ = 8.5 Hz, 2 H), 6.79 (d, J = 8.4 Hz, 2 H), 6.57 (d, J = 8.5 Hz, 2 H), 3.73 (s, 3 H), 3.63 (sex, J = 6.9 Hz, 1 H), 3.11 (br s, 1 H), 2.85 (dd, J = 13.4 Hz, J = 4.4 Hz, 1 H), 2.65 (dd, J = 13.4 Hz, J

= 7.1 Hz, 1 H), 1.10 (d, J = 6.3 Hz, 3 H). **^{13}C NMR** (100 MHz, $CDCl_3$): δ 161.6 (d, $^1J_{CF}$ = 240.3 Hz), 152.2, 141.4, 134.4 (d, $^4J_{CF}$ = 3.1 Hz), 130.9 (d, $^3J_{CF}$ = 7.7 Hz), 115.1 (d, $^2J_{CF}$ = 20.6 Hz), 115.12, 115.05, 55.8, 50.5, 41.5, 20.3. **HRMS** (ESI): Calcd for $C_{16}H_{19}FNO$ [M+H]: 260.1445; Found: 260.1448.

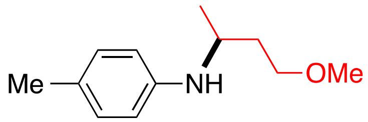

***N*-(4-Methoxybutan-2-yl)-4-methylaniline (5c).** Following the general procedure A, the title compound was prepared using 4-nitrotoluene (1 equiv, 0.50 mmol, 69 mg) and 3-iodo-1-methoxybutane (5 equiv, 2.5 mmol, 535 mg). The crude product was purified by flash chromatography using hexanes and then hexanes/EtOAc (25:1) as eluents to afford the title compound (**5c**) as brown oil (78 mg, 81%). **^{1}H NMR** (400 MHz, $CDCl_3$): δ 6.96 (d, J = 7.8 Hz, 2 H), 6.53 (d, J = 7.9 Hz, 2 H), 3.63 (br s, 1 H), 3.48-3.43 (m, 1 H), 3.40-3.31 (ovrlp, 5 H), 2.22 (s, 3 H), 1.71-1.61 (m, 1 H), 1.60-1.50 (m, 1 H), 0.96 (ovrlp, 3 H). **^{13}C NMR** (100 MHz, $CDCl_3$): δ 145.6, 129.8, 126.4, 113.6, 74.0, 59.2, 54.7, 25.0, 20.5, 10.7. **HRMS** (ESI): Calcd for $C_{12}H_{20}NO$ [M+H]: 194.1545; Found: 194.1541.

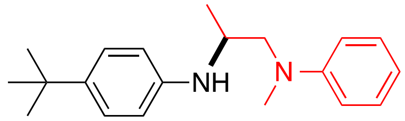

***N*^{2}-(4-(*tert*-Butyl)phenyl)-*N*^{1}-methyl-*N*^{1}-phenylpropane-1,2-diamine (5d).** Following the general procedure A, the title compound was prepared using Zn (3 equiv, 0.90 mmol, 59 mg), $FeCl_2$•$4H_2O$ (20 mol %, 0.060 mmol, 12 mg), 1-(*tert*-butyl)-4-nitrobenzene (1 equiv, 0.30 mmol, 54 mg), *N*-(2-iodopropyl)-*N*-methylaniline (3 equiv, 0.90 mmol, 248 mg), NMP (0.60 mL), and TMSCl (2 equiv, 0.60 mmol, 77 μL). The crude product was purified by flash chromatography using hexanes and then hexanes/EtOAc (25:1) as eluents to afford the title compound (**5d**) as viscous brown oil (45 mg, 50%). **^{1}H NMR** (400 MHz, $CDCl_3$): δ 7.23 (t, J = 7.5 Hz, 2 H), 7.18 (d, J = 8.0 Hz, 2 H), 6.76-6.70 (ovrlp, 3 H), 6.54 (d, J = 7.8 Hz, 2 H), 3.79 (sex, J = 6.2 Hz, 1 H), 3.59-3.12 (ovrlp, 3 H), 2.95 (s, 3 H), 1.27 (s, 9 H), 1.22 (d, J = 6.0 Hz, 3 H). **^{13}C NMR** (100 MHz, $CDCl_3$): δ 149.9, 145.1, 140.3, 129.3, 126.2, 116.7, 113.2, 112.5, 59.0, 48.0, 39.5, 34.0, 31.7, 19.7. **HRMS** (ESI): Calcd for $C_{20}H_{29}N_2$ [M+H]: 297.2325; Found: 297.2330.

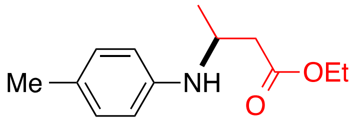

Ethyl 3-(*p*-Tolylamino)butanoate (5e). Following the general procedure A, the title compound was prepared using 4-nitrotoluene (1 equiv, 0.50 mmol, 69 mg) and ethyl 3-iodobutanoate (5 equiv, 2.5 mmol, 605 mg). The crude product was purified by flash chromatography using hexanes and then hexanes/EtOAc (20:1) as eluents to afford the title compound (**5e**) as brown oil (73 mg, 66%). **^{1}H NMR** (400 MHz, $CDCl_3$): δ 6.98 (d, J = 7.8 Hz, 2 H), 6.55 (d, J = 7.8 Hz, 2 H), 4.13 (q, J = 7.0 Hz, 2

H), 3.90 (sex, J = 6.2 Hz, 1 H), 3.60 (br s, 1 H), 2.61 (dd, J = 15.0 Hz, J = 5.0 Hz, 1 H), 2.39 (dd, J = 15.0 Hz, J = 6.8 Hz, 1 H), 2.23 (s, 3 H), 1.26-1.28 (ovrlp, 6 H). ^{13}**C NMR** (100 MHz, $CDCl_3$): δ 172.0, 144.6, 129.9, 127.0, 114.0, 60.5, 46.5, 41.1, 20.7, 20.5, 14.3. **HRMS** (ESI): Calcd for $C_{13}H_{20}NO_2$ [M+H]: 222.1489; Found: 222.1491.

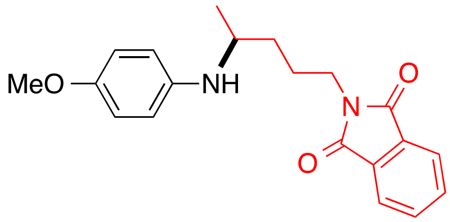

2-(4-((4-Methoxyphenyl)amino)pentyl)isoindoline-1,3-dione (5f). Following the general procedure A, the title compound was prepared using 4-nitroanisole (77 mg) and 2-(4-iodopentyl)isoindoline-1,3-dione (515 mg). The crude product was purified by flash chromatography using hexanes and then hexanes/EtOAc (25:1) as eluents to afford the title compound (**5f**) as viscous yellow oil (90 mg, 53%). ^{1}**H NMR** (400 MHz, $CDCl_3$): δ 7.84-7.81 (m, 2 H), 7.72-7.68 (m, 2 H), 6.73 (d, J = 8.0 Hz, 2 H), 6.53 (d, J = 8.0 Hz, 2 H), 3.72-3.68 (ovrlp, 5 H), 3.42 (sex, J = 6.1 Hz, 1 H), 2.95 (br s, 1 H), 1.86-1.73 (m, 2 H), 1.63-1.55 (m, 1 H), 1.53-1.44 (m, 1 H), 1.14 (d, J = 6.1 Hz, 3 H). ^{13}**C NMR** (100 MHz, $CDCl_3$): δ 168.6, 152.0 141.7, 134.0, 132.2, 123.3, 115.0, 114.9, 55.9, 49.4, 38.1, 34.2, 25.4, 21.0. **HRMS** (ESI): Calcd for $C_{20}H_{23}N_2O_3$ [M+H]: 339.1703; Found: 339.1710.

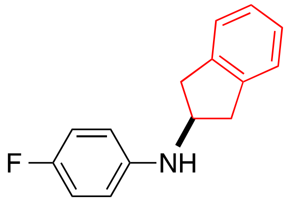

***N*-(4-Fluorophenyl)-2,3-dihydro-1*H*-inden-2-amine (5g).** Following the general procedure A, the title compound was prepared using 1-fluoro-4-nitrobenzene (71 mg) and 2-iodo-2,3-dihydro-1*H*-indene (366 mg). The crude product was purified by flash chromatography using hexanes as an eluent to afford the title compound (**5g**) as off-white solid (97 mg, 85%). ^{1}**H NMR** (400 MHz, $CDCl_3$): δ 7.24-7.20 (m, 2 H), 7.19-7.15 (m, 2 H), 6.88 (dd, $^3J_{HF}$ = 9.4 Hz, $^3J_{HH}$ = 8.5 Hz, 2 H), 6.55-6.52 (m, 2 H), 4.26 (qu, J = 5.8 Hz, 1 H), 3.72 (s, 1 H), 3.34 (d, J = 16.1 Hz, J = 6.7 Hz, 2 H), 3.34 (d, J = 16.1 Hz, J = 4.2 Hz, 2 H). ^{13}**C NMR** (100 MHz, $CDCl_3$): δ 114.4 (d, $^1J_{CF}$ = 233.6 Hz), 143.8 (d, $^4J_{CF}$ = 1.9 Hz), 141.4, 126.8, 125.1, 115.8 (d, $^2J_{CF}$ = 22.1 Hz), 113.8 (d, $^3J_{CF}$ = 7.3 Hz), 54.7, 40.3. **HRMS** (ESI): Calcd for $C_{15}H_{15}FN$ [M+H]: 228.1183; Found: 228.1186.

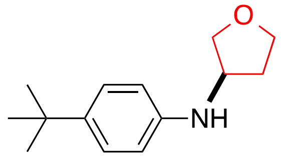

***N*-(4-(*tert*-Butyl)phenyl)tetrahydrofuran-3-amine (5h).** Following the general procedure A, the title compound was prepared using 1-(*tert*-butyl)-4-nitrobenzene (90 mg) and 3-iodotetrahydrofuran (297 mg). The crude product was purified by flash chromatography using hexanes and then hexanes/EtOAc (25:1) as eluents to afford the title compound (**5h**) as brown solid (64 mg, 58%). **^{1}H NMR** (400 MHz, $CDCl_3$): δ 7.21 (d, *J* = 8.0 Hz, 2 H), 6.55 (d, *J* = 8.0 Hz, 2 H), 4.09-4.03 (m, 1 H), 3.96-3.91 (ovrlp, 2 H), 3.85-3.80 (m, 1 H), 3.78-3.39 (ovrlp, 2 H), 2.67-2.18 (m, 1 H), 1.89-1.82 (m, 1 H), 1.28 (s, 9 H). **^{13}C NMR** (100 MHz, $CDCl_3$): δ 144.8, 140.6, 126.2, 113.1, 73.9, 67.2, 54.1, 33.9, 33.4, 31.6. **HRMS** (ESI): Calcd for $C_{14}H_{22}NO$ [M+H]: 220.1696; Found: 220.1698.

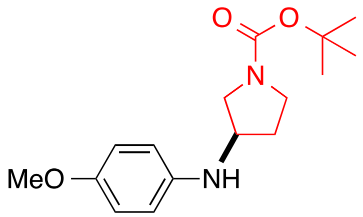

***tert*-Butyl 3-((4-methoxyphenyl)amino)pyrrolidine-1-carboxylate (5i).** Following the general procedure A, the title compound was prepared using 4-nitroanisole (77 mg) and *tert*-butyl 3-iodopyrrolidine-1-carboxylate (446 mg). The crude product was purified by flash chromatography using hexanes and then hexanes/EtOAc (20:1) as eluents to afford the title compound (**5i**) as brown solid (102 mg, 70%). **^{1}H NMR** (400 MHz, $CDCl_3$): δ 6.79 (d, *J* = 7.6 Hz, 2 H), 6.58 (d, *J* = 8.1 Hz, 2 H), 3.97 (br s, 1 H), 3.78-3.62 (ovrlp, 4 H), 3.53-3.38 (ovrlp, 3 H), 3.27-3.16 (m, 1 H), 2.20-2.11 (m, 1 H), 1.92-1.77 (m, 1 H), 1.46 (s, 9 H). **^{13}C NMR** (100 MHz, $CDCl_3$): δ 154.8, 152.6, 141.1, 115.1, 114.9, 79.5, 55.9, 54.1 (53.3), 52.3 (52.0), 44.3 (43.9), 32.0 (31.4), 28.7 (The value in parentheses corresponds to the chemical shift of the conformer of the same carbon). **HRMS** (ESI): Calcd for $C_{16}H_{25}N_2O_3$ [M+H]: 293.1860; Found: 293.1864.

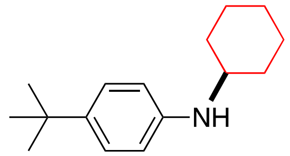

4-(*tert*-Butyl)-*N*-cyclohexylaniline (5j).[33] Following the general procedure A, the title compound was prepared using 1-(*tert*-butyl)-4-nitrobenzene (1 equiv, 0.50 mmol, 90 mg) and iodocyclohexane (5 equiv, 2.5 mmol, 324 μL). The crude product was purified by flash chromatography using hexanes as an eluent to afford the title compound (**5j**) as viscous brown oil (68 mg, 59%). **^{1}H NMR** (400 MHz, $CDCl_3$): δ 7.18 (d, *J* = 8.2 Hz, 2 H), 6.54 (d, *J* = 8.1 Hz, 2 H), 3.69-3.02 (ovrlp, 2 H), 2.05 (d, *J* = 12.0 Hz, 2 H), 1.75 (d, *J* = 13.0 Hz, 2 H), 1.64 (d, *J* = 12.5 Hz, 2 H), 1.43-1.31 (m, 2 H), 1.27 (s, 9 H), 1.23-1.10 (ovrlp, 3 H). **^{13}C NMR** (100 MHz, $CDCl_3$): δ 145.2, 139.7, 126.1, 113.0, 52.1, 33.9, 33.8, 31.7, 26.1, 25.2.

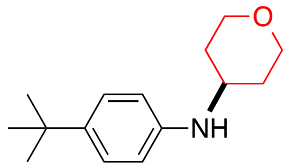

***N*-(4-(*tert*-Butyl)phenyl)tetrahydro-2*H*-pyran-4-amine (5k).** Following the general procedure A, the title compound was prepared using 1-(*tert*-butyl)-4-nitrobenzene (90 mg) and 4-iodotetrahydro-2*H*-pyran (318 mg). The crude product was purified by flash chromatography using hexanes and then hexanes/EtOAc (25:1) as eluents as an eluent to afford the title compound (**5k**) as viscous brown oil (63 mg, 54%). **^{1}H NMR** (400 MHz, $CDCl_3$): δ 7.20 (d, *J* = 7.9 Hz, 2 H), 6.57 (d, *J* = 7.9 Hz, 2 H), 3.99 (d, *J* = 11.7 Hz, 2 H), 3.62-3.31 (ovrlp, 4 H), 2.03 (d, *J* = 12.7 Hz, 2 H), 1.51-1.41 (m, 2 H), 1.27 (s, 9 H). **^{13}C NMR** (100 MHz, $CDCl_3$): δ 144.5, 140.4, 126.2, 113.2, 67.0, 49.4, 34.0, 33.9, 31.7. **HRMS** (ESI): Calcd for $C_{15}H_{24}NO$ [M+H]: 234.1852; Found: 234.1857.

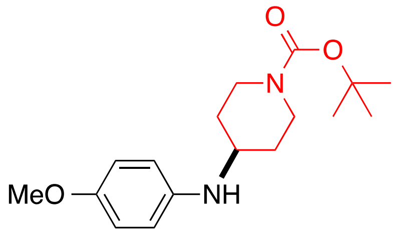

***tert*-Butyl 4-((4-methoxyphenyl)amino)piperidine-1-carboxylate (5l).** Following the general procedure A, the title compound was prepared using 4-nitroanisole (77 mg) and *tert*-butyl 4-iodopiperidine-1-carboxylate (467 mg). The crude product was purified by flash chromatography using hexanes and then hexanes/EtOAc (20:1) as eluents to afford the title compound (**5l**) as viscous brown oil (74 mg, 48%). **^{1}H NMR** (400 MHz, $CDCl_3$): δ 6.78 (d, *J* = 8.1 Hz, 2 H), 6.59 (d, *J* = 8.0 Hz, 2 H), 4.08-3.99 (m, 2 H), 3.74 (s, 3 H), 3.33 (t, *J* = 10.0 Hz, 1 H), 3.22-2.66 (ovrlp, 3 H), 2.02 (d, *J* = 12.3 Hz, 2 H), 1.46 (s, 9 H), 1.35-1.24 (m, 2 H). **^{13}C NMR** (100 MHz, $CDCl_3$): δ 154.9, 152.5, 141.0, 115.3, 115.1, 79.7, 55.9, 51.4, 42.9, 32.7, 28.6. **HRMS** (ESI): Calcd for $C_{17}H_{27}N_2O_3$ [M+H]: 307.2022; Found: 307.2022.

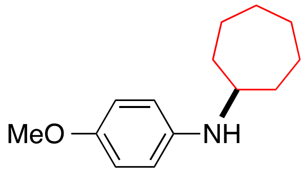

***N*-(4-Methoxyphenyl)cycloheptanamine (5m).** Following the general procedure A, the title compound was prepared using 4-nitroanisole (77 mg) and iodocycloheptane (336 mg). The crude product was purified by flash chromatography using hexanes and then hexanes/EtOAc (25:1) as eluents to afford the title compound (**5m**) as viscous brown oil (76 mg, 69%). **^{1}H NMR** (400 MHz, $CDCl_3$): δ 6.76 (d, *J* = 7.8 Hz, 2 H), 6.51 (d, *J* = 7.9 Hz, 2 H), 3.73 (s, 3 H), 3.39-3.33 (m, 1 H), 3.21 (br s, 1 H), 2.03-1.94 (m, 2 H), 1.68-1.38 (ovrlp, 10 H). **^{13}C NMR** (100 MHz, $CDCl_3$): δ 151.8, 141.7, 115.0, 114.8, 55.9, 54.7, 35.0, 28.5, 24.5. **HRMS** (ESI): Calcd for $C_{14}H_{22}NO$ [M+H]: 220.1651; Found: 220.1698.

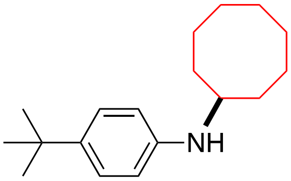

***N*-(4-(*tert*-Butyl)phenyl)cyclooctanamine (5n).** Following the general procedure A, the title compound was prepared using 1-(*tert*-butyl)-4-nitrobenzene (1 equiv, 0.50 mmol, 90 mg) and iodocyclooctane (5 equiv, 2.5 mmol, 595 mg). The crude product was purified by flash chromatography using hexanes as an eluent to afford the title compound (**5n**) as viscous brown oil (66 mg, 53%). **^{1}H NMR** (400 MHz, $CDCl_3$): δ 7.19 (d, *J* = 7.8 Hz, 2 H), 6.52 (d, *J* = 8.0 Hz, 2 H), 3.96-2.97 (ovrlp, 2 H), 1.91-1.85 (m, 2 H), 1.75-1.68 (m, 2 H), 1.64-1.50 (ovrlp, 10 H), 1.27 (s, 9 H). **^{13}C NMR** (100 MHz, $CDCl_3$): δ 144.8, 139.8, 126.2, 113.2, 53.1, 34.0, 32.9, 31.7, 27.7, 26.1, 24.2. **HRMS** (ESI): Calcd for $C_{18}H_{30}N$ [M+H]: 260.2373; Found: 260.2375.

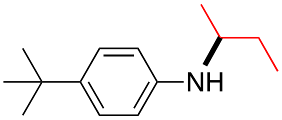

***N*-(*sec*-Butyl)-4-(*tert*-butyl)aniline (5o).** Following the general procedure A, the title compound was prepared using 1-(*tert*-butyl)-4-nitrobenzene (1 equiv, 0.50 mmol, 90 mg) and 2-bromobutane (5 equiv, 2.5 mmol, 274 μL). The crude product was purified by flash chromatography using hexanes as an eluent to afford the title compound (**5p**) as brown oil (55 mg, 53%). **^{1}H NMR** (400 MHz, $CDCl_3$): δ 7.17 (d, *J* = 8.0 Hz, 2 H), 6.53 (d, *J* = 8.1 Hz, 2 H), 3.45-3.32 (ovrlp, 2 H), 1.64-1.54 (m, 1 H), 1.51-1.42 (m, 1 H), 1.27 (s, 9 H), 1.16 (d, *J* = 6.2 Hz, 3 H), 0.94 (t, *J* = 7.4 Hz, 3 H). **^{13}C NMR** (100 MHz, $CDCl_3$): δ 145.5, 139.6, 126.1, 112.9, 50.1, 33.9, 31.7, 29.9, 20.5, 10.6. **HRMS** (ESI): Calcd for $C_{14}H_{24}N$ [M+H]: 206.1909; Found: 206.1907.

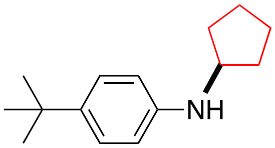

4-(*tert*-Butyl)-*N*-cyclopentylaniline (5p). Following the general procedure A, the title compound was prepared using 1-(*tert*-butyl)-4-nitrobenzene (1 equiv, 0.50 mmol, 90 mg) and bromocyclopentane (5 equiv, 2.5 mmol, 224 mg). The crude product was purified by flash chromatography using hexanes as an eluent to afford the title compound (**5o**) as brown oil (49 mg, 45%). **^{1}H NMR** (400 MHz, $CDCl_3$): δ 7.19 (d, *J* = 8.2 Hz, 2 H), 6.55 (d, *J* = 7.7 Hz, 2 H), 3.76 (qu, *J* = 5.9 Hz, 1 H), 3.56 (br s, 1 H), 2.04-1.96 (m, 2 H), 1.76-1.67 (m, 2 H), 1.65-1.56 (m, 2 H), 1.50-1.42 (m, 2 H), 1.27 (s, 9 H). **^{13}C NMR** (100 MHz, $CDCl_3$): δ 145.8, 139.8, 126.1, 113.0, 55.0, 34.0, 33.8, 31.7, 24.2. **HRMS** (ESI): Calcd for $C_{15}H_{24}N$ [M+H]: 218.1909; Found: 218.1901.

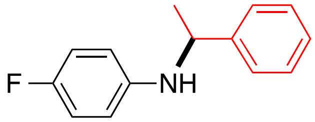

4-Fluoro-*N*-(1-phenylethyl)aniline (5q).[34] Following the general procedure A, the title compound was prepared using Zn (3.5 equiv, 1.75 mmol, 114.5 mg), $FeCl_2$•$4H_2O$ (30 mol%, 0.15 mmol, 30 mg), 1-fluoro-4-nitrobenzene (1 equiv, 0.50 mmol, 71 mg) and (1-bromoethyl)benzene (5 equiv, 2.5 mmol, 463 mg). The crude product was purified by flash chromatography using hexanes as an eluent to afford the title compound (**5q**) as viscous brown oil (59 mg, 55%). **^{1}H NMR** (400 MHz, $CDCl_3$): δ 7.36-7.30 (ovrlp, 4 H), 7.23 (t, J = 6.4 Hz, 1 H), 6.79 (dd, $^3J_{HF}$ = 9.3 Hz, $^3J_{HH}$ = 8.6 Hz, 2 H), 6.44-6.41 (m, 2 H), 4.41 (q, J = 6.7 Hz, 1 H), 3.92 (br s, 1 H), 1.50 (d, J = 6.8 Hz, 3 H). **^{13}C NMR** (100 MHz, $CDCl_3$): δ 155.8 (d, $^1J_{CF}$ = 233.2 Hz), 145.2, 143.7 (d, $^4J_{CF}$ = 1.7 Hz), 128.8, 127.1, 125.9, 115.6 (d, $^2J_{CF}$ = 22.1 Hz), 114.2 (d, $^3J_{CF}$ = 7.3 Hz), 54.2, 25.2.

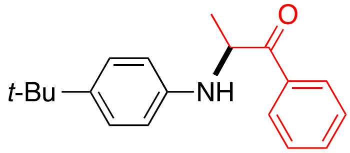

2-((4-(*tert*-Butyl)phenyl)amino)-1-phenylpropan-1-one (5r). Following the general procedure A, the title compound was prepared using Zn (3.5 equiv, 1.75 mmol, 114.5 mg), $FeCl_2$•$4H_2O$ (30 mol%, 0.15 mmol, 30 mg), 1-(*tert*-butyl)-4-nitrobenzene (1 equiv, 0.50 mmol, 90 mg), and 2-bromo-1-phenylpropan-1-one (5 equiv, 2.5 mmol, 533 mg). The crude product was purified by flash chromatography using hexanes and then hexanes/EtOAc (20:1) as eluents to afford the title compound (**5r**) as pale-brown solid (70 mg, 50%). **^{1}H NMR** (400 MHz, $CDCl_3$): δ 8.01 (d, J = 7.7 Hz, 2 H), 7.60 (t, J = 7.3 Hz, 1 H), 7.50 (t, J = 7.6 Hz, 2 H), 7.20 (d, J = 7.3 Hz, 2 H), 6.63 (d, J = 7.4 Hz, 2 H), 5.10 (qu, J = 7.1 Hz, 1 H), 4.62 (br s, 1 H), 1.47 (d, J = 7.0 Hz, 3 H), 1.26 (s, 9 H). **^{13}C NMR** (100 MHz, $CDCl_3$): δ 201.0, 144.3, 140.8, 134.9, 133.7, 129.0, 128.6, 126.3, 113.3, 53.8, 34.0, 32.0, 19.9. **HRMS** (ESI): Calcd for $C_{19}H_{24}NO$ [M+H]: 282.1858; Found: 282.1862.

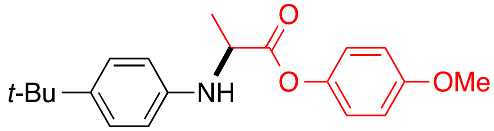

4-Methoxyphenyl (4-(*tert*-Butyl)phenyl)-*L*-alaninate (5s). Following the general procedure A, the title compound was prepared using Zn (3.5 equiv, 1.75 mmol, 114.5 mg), $FeCl_2$•$4H_2O$ (30 mol%, 0.15 mmol, 30 mg), 1-(*tert*-butyl)-4-nitrobenzene (1 equiv, 0.50 mmol, 90 mg), and 4-methoxyphenyl 2-bromopropanoate (5 equiv, 2.5 mmol, 648 mg). The crude product was purified by flash chromatography using hexanes and then hexanes/EtOAc (20:1) as eluents to afford the title compound (**5s**) as pale-brown solid (71 mg, 43%). **^{1}H NMR** (400 MHz, $CDCl_3$): δ 7.23 (d, J = 8.2 Hz, 2 H), 6.91 (d, J = 8.8 Hz, 2 H), 6.85 (d, J = 8.6 Hz, 2 H), 6.65 (d, J = 8.2 Hz, 2 H), 4.34 (qu, J = 7.0 Hz, 1 H),

4.08 (br s, 1 H), 3.78 (s, 3 H), 1.62 (d, *J* = 6.9 Hz, 3 H), 1.28 (s, 9 H). ^{13}**C NMR** (100 MHz, $CDCl_3$): δ 174.0, 157.5, 144.23, 144.16, 141.6, 126.3, 122.2, 114.6, 113.5, 55.7, 52.7, 34.1, 31.7, 19.1. **HRMS** (ESI): Calcd for $C_{20}H_{26}NO_3$ [M+H]: 328.1913; Found: 328.1905.

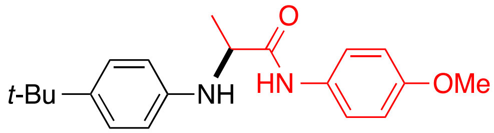

2-((4-(*tert*-Butyl)phenyl)amino)-*N*-(4-methoxyphenyl)propanamide (5t). Following the general procedure A, the title compound was prepared using Zn (3.5 equiv, 1.75 mmol, 114.5 mg), $FeCl_2$•$4H_2O$ (30 mol%, 0.15 mmol, 30 mg), 1-(*tert*-butyl)-4-nitrobenzene (1 equiv, 0.50 mmol, 90 mg), and 2-bromo-*N*-(4-methoxyphenyl)propanamide (5 equiv, 2.5 mmol, 645 mg). The crude product was purified by flash chromatography using hexanes and then hexanes/EtOAc (15:1) as eluents to afford the title compound (**5t**) as a white solid (94 mg, 57%). ^{1}**H NMR** (400 MHz, $CDCl_3$): δ 8.66 (br s, 1 H), 7.43 (d, *J* = 8.4 Hz, 2 H), 7.24 (d, *J* = 8.4 Hz, 2 H), 6.83 (d, *J* = 8.4 Hz, 2 H), 6.63 (d, *J* = 8.0 Hz, 2 H), 3.95-3.80 (ovrlp, 2 H), 3.77 (s, 3 H), 1.57 (d, *J* = 7.0 Hz, 3 H), 1.27 (s, 9 H). ^{13}**C NMR** (100 MHz, $CDCl_3$): δ 172.4, 156.5, 144.3, 142.7, 130.8, 126.4, 121.7, 114.2, 113.9, 56.7, 55.6, 34.1, 31.6, 20.0. **HRMS** (ESI): Calcd for $C_{20}H_{27}N_2O_2$ [M+H]: 327.2072; Found: 327.2076.

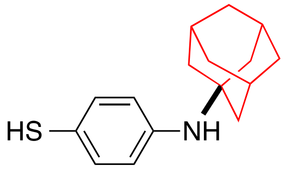

4-((Adamantan-1-yl)amino)benzenethiol (5u). Following the general procedure A, the title compound was prepared using Zn (4 equiv, 1.4 mmol, 92 mg), $FeCl_2$•$4H_2O$ (30 mol %, 0.105 mmol, 21 mg), 4-nitrobenzenethiol (1 equiv, 0.35 mmol, 54 mg), 1-iodoadamantane (4 equiv, 1.4 mmol, 367 mg), NMP (1.0 mL), and TMSCl (2 equiv, 0.70 mmol, 89 μL). The crude product was purified by flash chromatography using hexanes and then hexanes/EtOAc (30:1) as eluents to afford the title compound (**5u**) as an off-white solid (40 mg, 44%). ^{1}**H NMR** (400 MHz, $CDCl_3$): δ 7.27 (d, *J* = 6.5 Hz, 2 H), 6.62 (d, *J* = 7.3 Hz, 2 H), 3.76 (br ovrlp, 2 H), 2.02-1.97 (m, 3 H), 1.79-1.75 (m, 6 H), 1.66-1.57 (m, 6 H). ^{13}**C NMR** (100 MHz, $CDCl_3$): δ 147.1, 139.0, 118.6, 114.9, 47.4, 43.5, 36.4, 30.1. **HRMS** (ESI): Calcd for $C_{16}H_{22}NS$ [M+H]: 260.1467; Found: 260.1472.

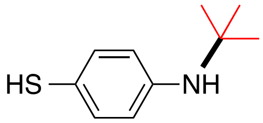

4-(*tert*-Butylamino)benzenethiol (5v). Following the general procedure A, the title compound was prepared using 4-nitrobenzenethiol (1 equiv, 0.50 mmol, 78 mg) and 2-iodo-2-methylpropane (5 equiv, 2.5 mmol, 460 mg). The crude product was purified by flash chromatography using hexanes and then hexanes/EtOAc (30:1) as eluents to afford the title compound (**5v**) as viscous brown oil (60

mg, 66%). **^{1}H NMR** (400 MHz, $CDCl_3$): δ 7.30 (d, *J* = 7.7 Hz, 2 H), 6.63 (d, *J* = 7.8 Hz, 2 H), 3.78 (br ovrlp, 2 H), 1.24 (s, 9 H). **^{13}C NMR** (100 MHz, $CDCl_3$): δ 147.2, 139.0, 120.8, 115.0, 45.5, 30.9. **HRMS** (ESI): Calcd for $C_{10}H_{16}NS$ [M+H]: 182.1003; Found: 182.1001.

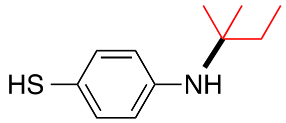

4-(*tert*-Pentylamino)benzenethiol (5w). Following the general procedure A, the title compound was prepared using Zn (3 equiv, 1.05 mmol, 69 mg), $FeCl_2$•$4H_2O$ (20 mol %, 0.070 mmol, 14 mg), 4-nitrobenzenethiol (1 equiv, 0.35 mmol, 54 mg), 2-bromo-2-methylbutane (5 equiv, 1.75 mmol, 264 mg), NMP (1.0 mL), and TMSCl (2 equiv, 0.70 mmol, 89 μL). The crude product was purified by flash chromatography using hexanes and then hexanes/EtOAc (30:1) as eluents to afford the title compound (**5w**) as viscous deep-brown oil (43 mg, 63%). **^{1}H NMR** (400 MHz, $CDCl_3$): δ 7.27 (d, *J* = 8.0 Hz, 2 H), 6.61 (d, *J* = 7.8 Hz, 2 H), 3.76 (br ovrlp, 2 H), 1.47 (qu, *J* = 7.3 Hz, 2 H), 1.17 (s, 6 H), 0.98 (t, *J* = 7.2 Hz, 3 H). **^{13}C NMR** (100 MHz, $CDCl_3$): δ 147.2, 138.9, 120.3, 115.0, 49.3, 34.6, 28.1, 9.3. **HRMS** (ESI): Calcd for $C_{11}H_{18}NS$ [M+H]: 196.1154; Found: 196.1159.

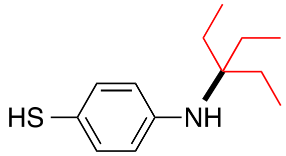

4-((3-Ethylpentan-3-yl)amino)benzenethiol (5x). Following the general procedure A, the title compound was prepared using Zn (4 equiv, 2.0 mmol, 131 mg), $FeCl_2$•$4H_2O$ (30 mol%, 0.15 mmol, 30 mg), 4-nitrobenzenethiol (1 equiv, 0.50 mmol, 78 mg), and 3-bromo-3-ethylpentane (5 equiv, 2.5 equiv, 448 mg). The crude product was purified by flash chromatography using hexanes and then hexanes/EtOAc (30:1) as eluents to afford the title compound (**5x**) as viscous brown oil (50 mg, 45%). **^{1}H NMR** (400 MHz, $CDCl_3$): δ 7.23 (d, *J* = 7.8 Hz, 2 H), 6.59 (d, *J* = 7.8 Hz, 2 H), 3.74 (br ovrlp, 2 H), 1.34 (q, *J* = 7.3 Hz, 6 H), 0.94 (t, *J* = 7.3 Hz, 9 H). **^{13}C NMR** (100 MHz, $CDCl_3$): δ 147.0, 138.7, 120.1, 115.0, 57.5, 27.7, 8.2. **HRMS** (ESI): Calcd for $C_{13}H_{22}NS$ [M+H]: 224.1467; Found: 224.1475.

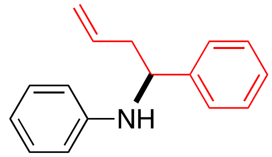

***N*-(1-Phenylbut-3-en-1-yl)aniline (5y).**[35] Following the general procedure A, the title compound was prepared using nitrobenzene (1 equiv, 0.50 mmol, 51 μL), $FeCl_2$•$4H_2O$ (25 mol %, 0.125 mmol, 25 mg), and (1-bromobut-3-en-1-yl)benzene (3 equiv, 1.5 mmol, 317 mg). The crude product was purified by flash chromatography using hexanes as an eluent to afford the title compound (**5y**) as viscous brown oil (49 mg, 44%). **^{1}H NMR** (400 MHz, $CDCl_3$): δ 7.37-7.29 (ovrlp, 4 H), 7.22 (t, *J* = 7.0 Hz, 1 H), 7.07 (t, *J* = 7.8 Hz, 2 H), 6.63 (t, *J* = 7.4 Hz, 1 H), 6.45 (d, *J* = 7.9 Hz, 2 H), 5.81-5.71

(m, 1 H), 5.20-5.13 (ovrlp, 2 H), 4.38 (dd, J = 8.1 Hz, J = 5.2 Hz, 1 H), 4.14 (br s, 1 H), 2.64-2.58 (m, 1 H), 2.53-2.45 (m, 1 H). **^{13}C NMR** (100 MHz, $CDCl_3$): δ 147.5, 143.7, 134.8, 129.2, 128.7, 127.1, 126.4, 118.4, 117.5, 113.6, 57.3, 43.4.

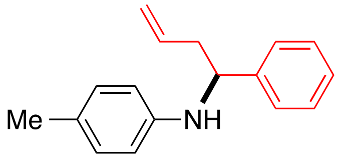

4-Methyl-*N*-(1-phenylbut-3-en-1-yl)aniline (5z).[35] Following the general procedure A, the title compound was prepared using 4-nitrotoluene (1 equiv, 0.50 mmol, 69 mg), $FeCl_2•4H_2O$ (25 mol %, 0.125 mmol, 25 mg), and (1-bromobut-3-en-1-yl)benzene (3 equiv, 1.5 mmol, 317 mg). The crude product was purified by flash chromatography using hexanes as an eluent to afford the title compound (**5y**) as viscous brown oil (53 mg, 45%). **^{1}H NMR** (400 MHz, $CDCl_3$): δ 7.36-7.28 (ovrlp, 4 H), 7.21 (t, J = 7.8 Hz, 1 H), 6.88 (d, J = 7.9 Hz, 2 H), 6.41 (d, J = 7.9 Hz, 2 H), 5.80-5.70 (m, 1 H), 5.19-5.11 (ovrlp, 2 H), 4.34 (dd, J = 8.4 Hz, J = 5.6 Hz, 1 H), 4.02 (br s, 1 H), 2.62-2.56 (m, 1 H), 2.51-2.43 (m, 1 H), 2.17 (s, 3 H). **^{13}C NMR** (100 MHz, $CDCl_3$): δ 145.2, 143.9, 134.9, 129.7, 128.7, 127.0, 126.6, 126.4, 118.3, 113.7, 57.5, 43.5, 20.5.

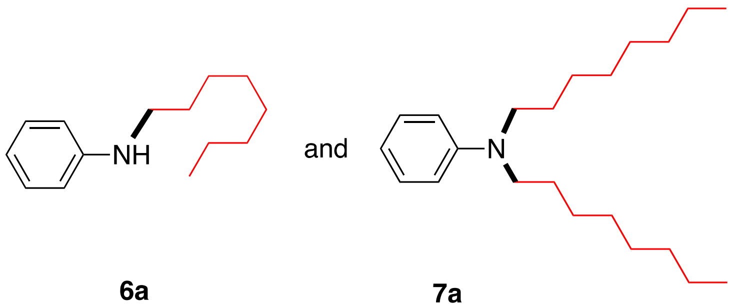

6a **7a**

***N*-Octylaniline (6a)**[36] **and *N,N*-Dioctylaniline (7a).**[37]

(i) 0.50 mmol scale: Following the general procedure B, the title compound was prepared using nitrobenzene (51 μL) and 1-iodooctane (180 mg). The crude product was purified by preparative TLC using hexanes as an eluent to afford the title compounds **6a** as pale-brown oil (58 mg, 56%) and **7a** as viscous colorless oil (20 mg, 12%).

Analysis of 6a: ^{1}H NMR (400 MHz, $CDCl_3$): δ 7.16 (t, J = 7.5 Hz, 2 H), 6.67 (t, J = 7.3 Hz, 1 H), 6.59 (d, J = 7.8 Hz, 2 H), 3.56 (br s, 1 H), 3.08 (t, J = 7.1 Hz, 2 H), 1.60 (qu, J = 7.2 Hz, 2 H), 1.43-1.23 (ovrlp 10 H), 0.89 (t, J = 6.7 Hz, 3 H). **^{13}C NMR** (100 MHz, $CDCl_3$): δ 148.7, 129.3, 117.2, 112.8, 44.1, 32.0, 29.7, 29.6, 29.4, 27.3, 22.8, 14.2.

Analysis of 7a: ^{1}H NMR (400 MHz, $CDCl_3$): δ 7.19 (t, J = 7.6 Hz, 2 H), 6.64-6.59 (ovrlp, 3 H), 3.24 (t, J = 7.8 Hz, 4 H), 1.57 (qu, J = 7.0 Hz, 4 H), 1.35-1.24 (ovrlp 20 H), 0.88 (t, J = 6.7 Hz, 6 H). **^{13}C NMR** (100 MHz, $CDCl_3$): δ 148.3, 129.3, 115.2, 111.8, 51.2, 32.0, 29.7, 29.5, 27.39, 27.37, 22.8,

14.3.

(ii) 10 mmol scale: The reaction was set up in a 250 mL Teflon-screw capped round-bottom flask instead of a 30 mL glass tube. Following the general procedure B, the title compound was prepared using Zn (5 equiv, 50 mmol, 3.27 g), $FeCl_2•4H_2O$ (20 mol %, 2.0 mmol, 398 mg), nitrobenzene (1 equiv, 10 mmol, 1.23 g), 1-iodooctane (1.5 equiv, 15 mmol, 3.61 g), NMP (40 mL), and TMSCl (4 equiv, 40 mmol, 5.1 mL), and the reaction was stirred at 90 °C for 2 days. The crude product was purified by flash chromatography (note: silica gel was not washed with hexanes/Et_3N) using hexanes as an eluent to afford the title compound **6a** as pale-brown oil (1.18 g, 57%) and **7a** as viscous colorless oil (583 mg, 18%). Spectral and analytical data were identical to those reported for the same compound above.

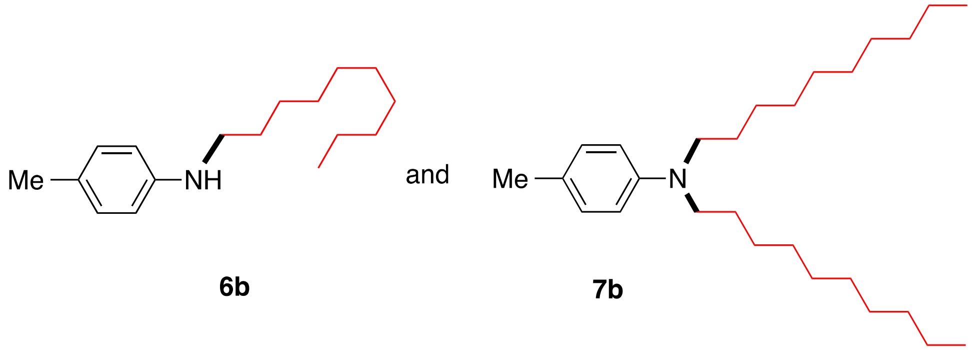

6b **7b**

***N*-Decyl-4-methylaniline (6b)**[38] **and *N,N*-Didecyl-4-methylaniline (7b).**[38] Following the general procedure B, the title compound was prepared using 4-nitrotoluene (69 mg) and 1-iododecane (201 mg). The crude product was purified by preparative TLC using hexanes as an eluent to afford the title compounds **6b** as white solid (69 mg, 55%) and **7b** as viscous pale-brown oil (47 mg, 24%).

Analysis of 6b: ^{1}**H NMR** (400 MHz, $CDCl_3$): δ 6.97 (d, *J* = 7.7 Hz, 2 H), 6.52 (d, *J* = 7.7 Hz, 2 H), 3.43 (br s, 1 H), 3.06 (t, *J* = 7.1 Hz, 2 H), 2.23 (s, 3 H), 1.59 (qu, *J* = 7.1 Hz, 2 H), 1.41-1.22 (ovrlp, 14 H), 0.88 (t, *J* = 6.8 Hz, 3 H). ^{13}**C NMR** (100 MHz, $CDCl_3$): δ 146.4, 129.8, 126.4, 113.0, 44.5, 32.0, 29.78, 29.76, 29.72, 29.6, 29.5, 27.3, 22.8, 20.5, 14.3.

Analysis of 7b: ^{1}**H NMR** (400 MHz, $CDCl_3$): δ 7.00 (d, *J* = 7.9 Hz, 2 H), 6.56 (d, *J* = 7.9 Hz, 2 H), 3.20 (t, *J* = 7.5 Hz, 4 H), 2.23 (s, 3 H), 1.54 (qu, *J* = 7.0 Hz, 4 H), 1.33-1.23 (ovrlp, 28 H), 0.88 (t, *J* = 6.7 Hz, 6 H). ^{13}**C NMR** (100 MHz, $CDCl_3$): δ 146.3, 129.8, 124.4, 112.3, 51.4, 32.1, 29.85, 29.75, 29.73, 29.5, 27.42, 27.39, 22.8, 20.3, 14.3.

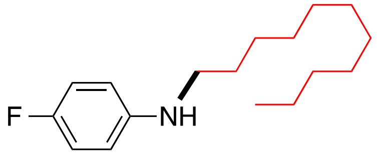

and

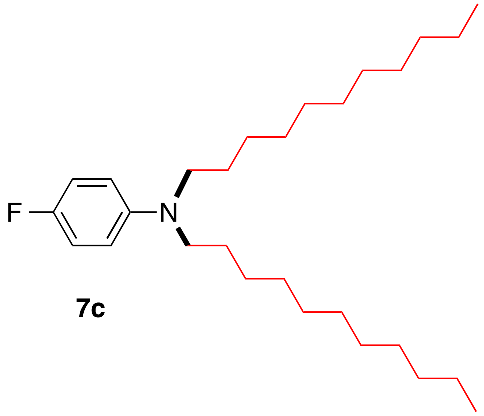

6c **7c**

4-Fluoro-*N*-undecylaniline (6c) and 4-Fluoro-*N*,*N*-diundecylaniline (7c). Following the general procedure B, the title compound was prepared using 1-fluoro-4-nitrobenzene (71 mg) and 1-iodoundecane (212 mg). The crude product was purified by preparative TLC using hexanes as an eluent to afford the title compounds **6c** as pale-brown oil (75 mg, 56%) and **7c** as viscous colorless oil (27 mg, 13%).

Analysis of 6c: ^{1}**H NMR** (400 MHz, $CDCl_3$): δ 6.87 (dd, $^3J_{HF}$ = 8.6 Hz, $^3J_{HH}$ = 8.3 Hz, 2 H), 6.53-6.50 (m, 2 H), 3.45 (br s, 1 H), 3.04 (t, J = 7.0 Hz, 2 H), 1.59 (qu, J = 7.0 Hz, 2 H), 1.41-1.23 (ovrlp, 16 H), 0.88 (t, J = 5.6 Hz, 3 H). ^{13}**C NMR** (100 MHz, $CDCl_3$): δ 155.8 (d, $^1J_{CF}$ = 233.1 Hz), 145.1 (d, $^4J_{CF}$ = 1.2 Hz), 115.7 (d, $^2J_{CF}$ = 22.1 Hz), 113.5 (d, $^3J_{CF}$ = 7.3 Hz), 44.8, 32.1, 29.8, 29.74, 29.71, 29.6, 29.5, 27.3, 22.8, 14.3. **HRMS** (ESI): Calcd for $C_{17}H_{29}FN$ [M+H]: 266.2284; Found: 266.2287.

Analysis of 7c: ^{1}**H NMR** (400 MHz, $CDCl_3$): δ 6.90 (dd, $^3J_{HF}$ = 8.6 Hz, $^3J_{HH}$ = 8.6 Hz, 2 H), 6.57-6.54 (m, 2 H), 3.18 (t, J = 7.6 Hz, 4 H), 1.53 (qu, J = 7.6 Hz, 4 H), 1.33-1.23 (ovrlp, 32 H), 0.88 (t, J = 6.8 Hz, 6 H). ^{13}**C NMR** (100 MHz, $CDCl_3$): δ 154.9 (d, $^1J_{CF}$ = 232.3 Hz), 145.2 (d, $^4J_{CF}$ = 1.3 Hz), 115.6 (d, $^2J_{CF}$ = 21.7 Hz), 113.2 (d, $^3J_{CF}$ = 7.1 Hz), 51.8, 32.1, 29.83, 29.78, 29.7, 29.5, 27.4, 27.3, 22.9, 14.3. **HRMS** (ESI): Calcd for $C_{28}H_{51}FN$ [M+H]: 420.4002; Found: 420.4005.

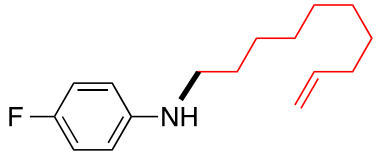

and

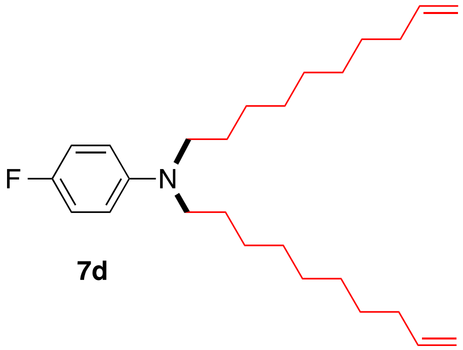

6d **7d**

***N*-(Dec-9-en-1-yl)-4-fluoroaniline (6d) and *N*,*N*-Di(dec-9-en-1-yl)-4-fluoroaniline (7d).** Following the general procedure B, the title compound was prepared using 1-fluoro-4-nitrobenzene (71 mg) and 10-iododec-1-ene (200 mg). The crude product was purified by preparative TLC using hexanes as an eluent to afford the title compounds **6d** as pale-brown oil (73 mg, 59%) and **7d** as viscous colorless oil (30 mg, 15%).

Analysis of 6d: ^{1}**H NMR** (400 MHz, $CDCl_3$): δ 6.87 (dd, $^3J_{HF}$ = 9.2 Hz, $^3J_{HH}$ = 8.4 Hz, 2 H), 6.52-6.50 (m, 2 H), 5.86-5.76 (m, 1 H), 5.01-4.92 (m, 2 H), 3.44 (br s, 1 H), 3.04 (t, J = 7.1 Hz, 2 H), 2.04 (q, J = 6.9 Hz, 2 H), 1.57 (qu, J = 7.2 Hz, 2 H), 1.42-1.26 (ovrlp, 10 H). ^{13}**C NMR** (100 MHz, $CDCl_3$):

δ 155.8 (d, $^{1}J_{CF}$ = 232.9 Hz), 145.0 (d, $^{4}J_{CF}$ = 1.5 Hz), 139.3, 115.7 (d, $^{2}J_{CF}$ = 22.1 Hz), 114.3, 113.5 (d, $^{3}J_{CF}$ = 7.3 Hz), 44.8, 33.9, 29.7, 29.55, 29.52, 29.2, 29.0, 27.3. **HRMS** (ESI): Calcd for $C_{16}H_{25}FN$ [M+H]: 250.1971; Found: 250.1975.

Analysis of 7d: ^{1}**H NMR** (400 MHz, $CDCl_3$): δ 6.90 (dd, $^{3}J_{HF}$ = 9.2 Hz, $^{3}J_{HH}$ = 8.4 Hz, 2 H), 6.57-6.54 (m, 2 H), 5.86-5.76 (m, 2 H), 4.99 (d, *J* = 17.2 Hz, 2 H), 4.93 (d, *J* = 10.1 Hz, 2 H), 3.18 (t, *J* = 7.7 Hz, 4 H), 2.04 (qu, *J* = 7.0 Hz, 4 H), 1.53 (qu, *J* = 6.8 Hz, 4 H), 1.42-1.26 (ovrlp, 20 H). ^{13}**C NMR** (100 MHz, $CDCl_3$): δ 154.8 (d, $^{1}J_{CF}$ = 232.5 Hz), 145.2 (d, $^{4}J_{CF}$ = 1.3 Hz), 139.3, 115.6 (d, $^{2}J_{CF}$ = 21.7 Hz), 114.3, 113.2 (d, $^{3}J_{CF}$ = 7.1 Hz), 51.8, 33.9, 29.65, 29.64, 29.2, 29.1, 27.3. **HRMS** (ESI): Calcd for $C_{26}H_{43}FN$ [M+H]: 388.3380; Found: 388.3382.

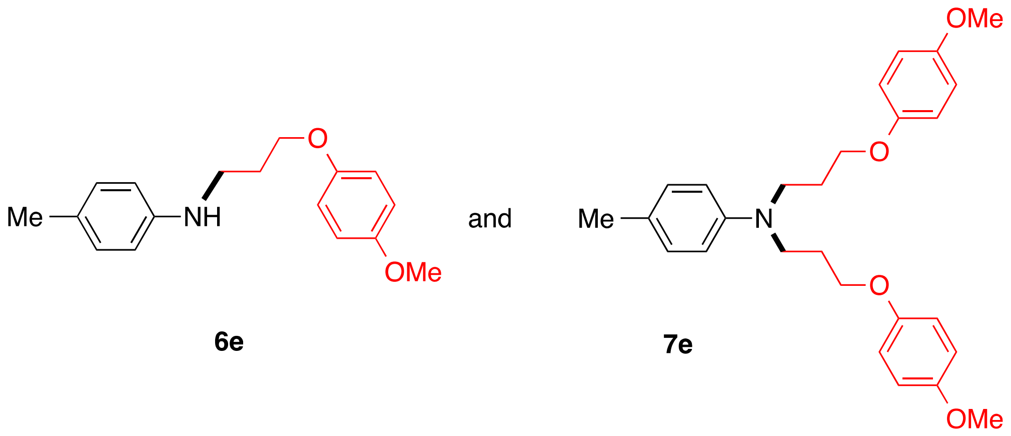

***N*-(3-(4-Methoxyphenoxy)propyl)-4-methylaniline (6e) and *N*,*N*-Bis(3-(4-methoxyphenoxy)propyl)-4-methylaniline (7e).** Following the general procedure B, the title compound was prepared using 4-nitrotoluene (69 mg) and 1-(3-iodopropoxy)-4-methoxybenzene (219 mg). The crude product was purified by preparative TLC using hexanes/EtOAc (40:1) as an eluent to afford the title compounds **6e** as off-white solid (70 mg, 52%). The dialkylated aniline, **7e**, could not be purified in pure form since it was co-isolated with **6e** (~48 mg, 22%); its formation was confirmed by HRMS analysis.

Analysis of 6e: ^{1}**H NMR** (400 MHz, $CDCl_3$): δ 6.99 (d, *J* = 7.8 Hz, 2 H), 6.86-6.82 (ovrlp, 4 H), 6.57 (d, *J* = 7.5 Hz, 2 H), 4.04 (t, *J* = 5.7 Hz, 2 H), 3.77 (s, 3 H), 3.32 (t, *J* = 6.5 Hz, 2 H), 2.24 (s, 3 H), 2.06 (qu, *J* = 6.2 Hz, 2 H). ^{13}**C NMR** (100 MHz, $CDCl_3$): δ 154.0, 153.1, 146.0, 129.9, 126.8, 115.6, 114.8, 113.3, 66.9, 55.9, 41.9, 29.3, 20.5. **HRMS** (ESI): Calcd for $C_{17}H_{22}NO_2$ [M+H]: 272.1650; Found: 272.1657.

Analysis of 7e: HRMS (ESI): Calcd for $C_{27}H_{33}NO_4$ [M+H]: 436.2488; Found: 436.2492.

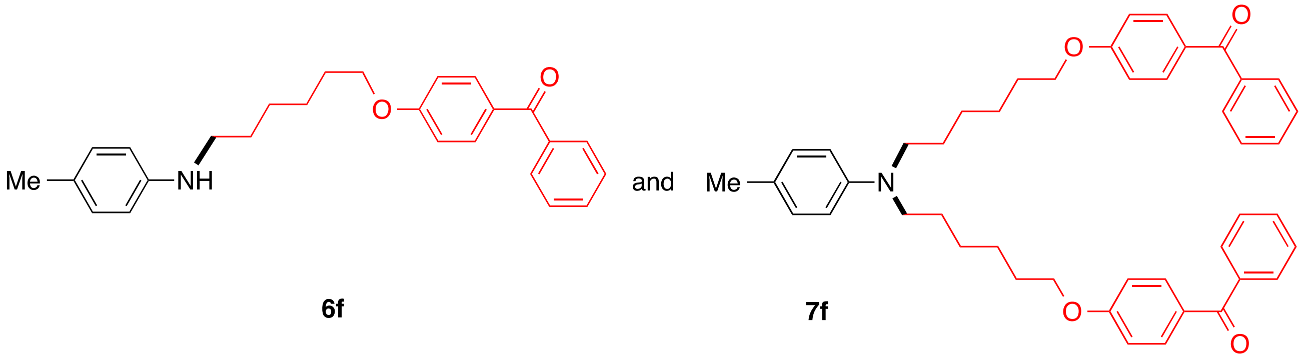

Phenyl(4-((6-(*p*-tolylamino)hexyl)oxy)phenyl)methanone (6f) and ((((*p*-tolylazanediyl)bis(hexane-6,1-diyl))bis(oxy))bis(4,1-phenylene))bis(phenylmethanone) (7f). Following the general procedure B, the title compound was prepared using 4-nitrotoluene (69 mg) and (4-((6-iodohexyl)oxy)phenyl)(phenyl)methanone (306 mg). The crude product was purified by preparative TLC using hexanes/EtOAc (50:1) as an eluent to afford the title compounds **6f** as pale-brown oil (94 mg, 48%). The dialkylated aniline, **7f**, could not be purified in pure form since it was co-isolated with **6f** (~34 mg, 10%); its formation was confirmed by HRMS analysis.

Analysis of 6f: ^{1}**H NMR** (400 MHz, $CDCl_3$): δ 7.74 (d, *J* = 8.2 Hz, 2 H), 7.68 (d, *J* = 7.4 Hz, 2 H), 7.48 (t, *J* = 7.0 Hz, 1 H), 7.39 (t, *J* = 7.3 Hz, 2 H), 6.91-6.85 (ovrlp, 4 H), 6.46 (d, *J* = 7.7 Hz, 2 H), 3.96 (t, *J* = 6.1 Hz, 2 H), 3.31 (br s, 1 H), 3.03 (t, *J* = 6.9 Hz, 2 H), 2.15 (s, 3 H), 1.76 (qu, *J* = 6.5 Hz, 2 H), 1.57 (qu, *J* = 6.5 Hz, 2 H), 1.49-1.37 (ovrlp, 4 H). ^{13}**C NMR** (100 MHz, $CDCl_3$): δ 195.7, 162.9, 146.3, 138.5, 132.7, 132.0, 130.1, 129.8, 128.3, 126.5, 114.1, 113.0, 68.2, 44.4, 29.7, 29.2, 27.0, 26.0, 20.5. **HRMS** (ESI): Calcd for $C_{26}H_{30}NO_2$ [M+H]: 388.2277; Found: 388.2279.

Analysis of 7f: HRMS (ESI): Calcd for $C_{45}H_{50}NO_4$ [M+H]: 668.3740; Found: 668.3730.

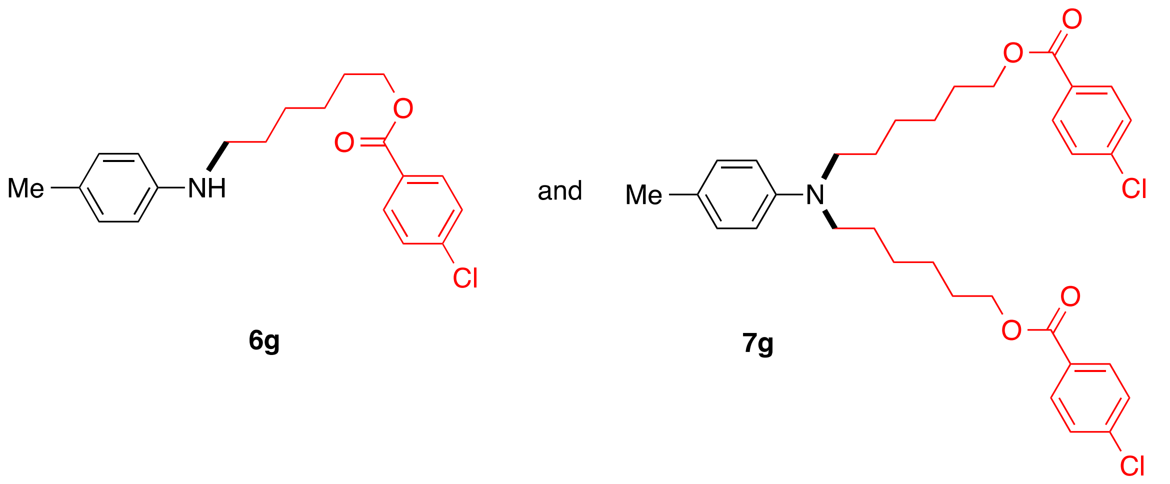

6-(*p*-Tolylamino)hexyl 4-Chlorobenzoate (6g) and (*p*-tolylazanediyl)bis(hexane-6,1-diyl) bis(4-chlorobenzoate) (7g). Following the general procedure B, the title compound was prepared using 4-nitrotoluene (69 mg) and 6-iodohexyl 4-chlorobenzoate (**S2**, 275 mg). The crude product was purified by preparative TLC using hexanes/EtOAc (50:1) as an eluent to afford the title compounds **6g** as brown solid (100 mg, 58%). The dialkylated aniline, **7g**, could not be purified in pure form since it was co-isolated with **6g** (~72 mg, 19%); its formation was confirmed by HRMS analysis.

Analysis of 6g: ^{1}**H NMR** (400 MHz, $CDCl_3$): δ 7.96 (d, J = 7.8 Hz, 2 H), 7.40 (d, J = 7.8 Hz, 2 H), 6.98 (d, J = 7.5 Hz, 2 H), 6.52 (d, J = 7.5 Hz, 2 H), 4.31 (t, J = 5.6 Hz, 2 H), 3.40 (br s, 1 H), 3.09 (t, J = 6.7 Hz, 2 H), 2.23 (s, 3 H), 1.82-1.73 (m, 2 H), 1.67-1.50 (m, 2 H), 1.53-1.39 (ovrlp, 4 H). ^{13}**C NMR** (100 MHz, $CDCl_3$): δ 165.9, 146.3, 139.4, 131.1, 129.8, 129.0, 128.8, 126.5, 113.0, 65.3, 44.3, 29.6, 28.8, 26.9, 26.0, 20.5. **HRMS** (ESI): Calcd for $C_{20}H_{25}ClNO_2$ [M+H]: 346.1568; Found: 346.1580.

Analysis of 7g: HRMS (ESI): Calcd for $C_{33}H_{40}Cl_2NO_4$ [M+H]: 584.2329; Found: 584.2346.

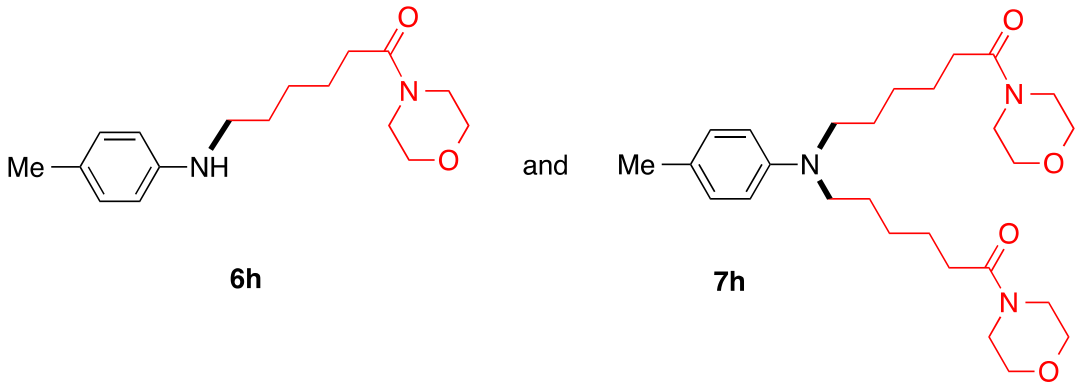

1-Morpholino-6-(*p*-tolylamino)hexan-1-one (6h) and 6,6'-(*p*-Tolylazanediyl)bis(1-morpholinohexan-1-one) (7h). Following the general procedure B, the title compound was prepared using 1-nitrotoluene (69 mg) and 6-iodo-1-morpholinohexan-1-one (**S3**, 233 mg). The crude product was purified by preparative TLC using hexanes/EtOAc (40:1) as an eluent to afford the title compounds **6h** as off-white solid (62 mg, 43%) and **7h** as viscous pale-brown oil (38 mg, 16%).

Analysis of 6h: ^{1}**H NMR** (400 MHz, $CDCl_3$): δ 6.97 (d, J = 7.8 Hz, 2 H), 6.52 (d, J = 7.8 Hz, 2 H), 3.68-3.58 (ovrlp, 6 H), 3.54-3.42 (ovrlp, 3 H), 3.10 (t, J = 7.0 Hz, 2 H), 2.32 (t, J = 7.4 Hz, 2 H), 2.23 (s, 3 H), 1.72-1.60 (ovrlp, 4 H), 1.45 (qu, J = 7.4 Hz, 2 H). ^{13}**C NMR** (100 MHz, $CDCl_3$): δ 171.7, 146.3, 129.8, 126.5, 113.0, 67.1, 66.8, 46.1, 44.2, 42.0, 33.0, 29.5, 27.1, 25.0, 20.5. **HRMS** (ESI): Calcd for $C_{17}H_{27}N_2O_2$ [M+H]: 291.2072; Found: 291.2076.

Analysis of 7h: ^{1}**H NMR** (400 MHz, $CDCl_3$): δ 7.00 (d, J = 7.8 Hz, 2 H), 6.56 (d, J = 7.8 Hz, 2 H), 3.69-3.59 (ovrlp, 12 H), 3.47-3.41 (m, 4 H), 3.22 (t, J = 7.2 Hz, 4 H), 2.31 (t, J = 7.4 Hz, 4 H), 2.29 (s, 3 H), 1.69-1.54 (ovrlp, 8 H), 1.36 (qu, J = 7.1 Hz, 4 H). ^{13}**C NMR** (100 MHz, $CDCl_3$): δ 171.7, 146.1, 129.9, 124.8, 112.5, 67.1, 68.8, 51.2, 46.2, 42.0, 33.1, 27.23, 27.16, 25.2, 20.3. **HRMS** (ESI): Calcd for $C_{27}H_{44}N_3O_4$ [M+H]: 474.3332; Found: 474.3329.

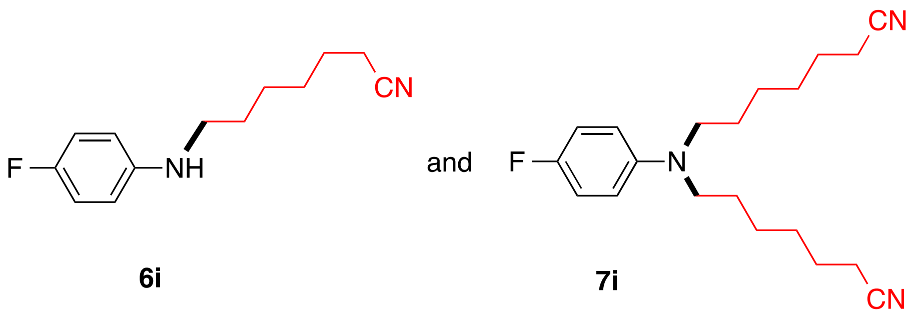

7-((4-Fluorophenyl)amino)heptanenitrile (6i) and 7,7'-((4-Fluorophenyl)azanediyl)diheptanenitrile (7i). Following the general procedure B, the title compound was prepared using 1-fluoro-4-nitrobenzene (71 mg) and 7-iodoheptanenitrile (178 mg). The crude product was purified by preparative TLC using hexanes/EtOAc (40:1) as an eluent to afford the title compounds **6i** as viscous pale-brown oil (64 mg, 58%) and **7i** as viscous pale-brown oil (22 mg, 13%).

Analysis of 6i: ^{1}H NMR (400 MHz, $CDCl_3$): δ 6.88 (dd, $^{3}J_{CF}$ = 9.5 Hz, $^{3}J_{HH}$ = 8.5 Hz, 2 H), 6.54-6.51 (m, 2 H), 3.41 (br s, 1 H), 3.07 (t, J = 7.0 Hz, 2 H), 2.34 (t, J = 6.9 Hz, 2 H), 1.71-1.59 (ovrlp, 4 H), 1.54-1.40 (ovrlp, 4 H). **^{13}C NMR** (100 MHz, $CDCl_3$): δ 155.8 (d, $^{1}J_{CF}$ = 233.2 Hz), 144.8 (d, $^{4}J_{CF}$ = 1.4 Hz), 119.8, 115.7 (d, $^{2}J_{CF}$ = 22.1 Hz), 113.6 (d, $^{3}J_{CF}$ = 7.3 Hz), 44.5, 29.3, 28.6, 26.5, 25.4, 17.2. **HRMS** (ESI): Calcd for $C_{13}H_{18}FN_2$ [M+H]: 221.1454; Found: 221.1454.

Analysis of 7i: ^{1}H NMR (400 MHz, $CDCl_3$): δ 6.91 (dd, $^{3}J_{CF}$ = 9.6 Hz, $^{3}J_{HH}$ = 8.0 Hz, 2 H), 6.59-6.55 (m, 2 H), 3.20 (t, J = 7.1 Hz, 4 H), 2.34 (t, J = 6.8 Hz, 4 H), 1.67 (qu, J = 7.1 Hz, 4 H), 1.59-1.45 (ovrlp, 8 H), 1.35 (qu, J = 7.2 Hz, 4 H). **HRMS (**ESI): Calcd for $C_{20}H_{29}FN_3$ [M+H]: 330.2346; Found: 330.2353. (^{13}C NMR was not shown due to the insufficient amount of **7b** for unambiguous splitting of signals in the presence of fluorine.)

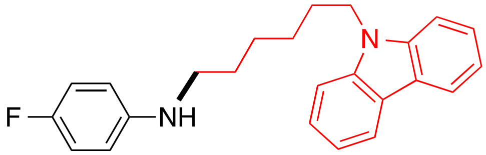

6j

and

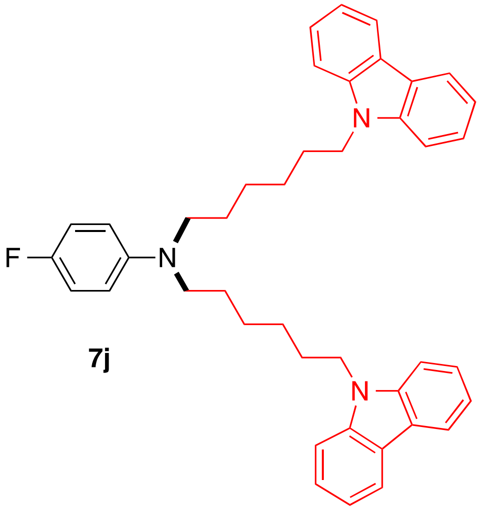

7j

***N*-(6-(9*H*-Carbazol-9-yl)hexyl)-4-fluoroaniline (6j) and *N*,*N*-bis(6-(9*H*-carbazol-9-yl)hexyl)-4-fluoroaniline (7j).** Following the general procedure B, the title compound was prepared using 1-fluoro-4-nitrobenzene (71 mg) and 9-(6-iodohexyl)-9*H*-carbazole (283 mg). The crude product was purified by preparative TLC using hexanes/EtOAc (50:1) as an eluent to afford the title compounds **6j** as viscous pale-yellow oil (90 mg, 50%) and **7j** as viscous pale-yellow oil (27 mg, 9%).

Analysis of 6j: ^{1}H NMR (400 MHz, $CDCl_3$): δ 8.09 (d, J = 7.6 Hz, 2 H), 7.44 (t, J = 7.4 Hz, 2 H), 7.37 (d, J = 8.1 Hz, 2 H), 7.22 (t, J = 7.4 Hz, 2 H), 6.85 (dd, $^{3}J_{HF}$ = 9.6 Hz, $^{3}J_{HH}$ = 8.3 Hz, 2 H), 6.46-6.44 (m, 2 H), 4.28 (t, J = 6.9 Hz, 2 H), 3.13 (br s, 1 H), 2.96 (t, J = 6.8 Hz, 2 H), 1.88 (qu, J = 5.6 Hz, 2 H), 1.51 (qu, J = 5.2 Hz, 2 H), 1.43-1.32 (ovrlp, 4 H). **^{13}C NMR** (100 MHz, $CDCl_3$): δ 155.8 (d, $^{1}J_{CF}$ = 253.1 Hz), 144.8 (d, $^{4}J_{CF}$ = 1.1 Hz), 140.5, 125.7, 122.9, 120.5, 118.9, 115.7 (d, $^{2}J_{CF}$ = 22.1 Hz), 113.6 (d, $^{3}J_{CF}$ = 7.3 Hz), 108.7, 44.6, 43.0, 29.4, 29.0, 27.3, 27.1. **HRMS** (ESI): Calcd for $C_{24}H_{26}FN_2$ [M+H]: 361.2075; Found: 361.2087.

Analysis of 7j: ^{1}H NMR (400 MHz, $CDCl_3$): δ 8.08 (d, J = 7.6 Hz, 4 H), 7.43 (t, J = 7.4 Hz, 4 H), 7.36 (d, J = 8.2 Hz, 4 H), 7.21 (t, J = 7.2 Hz, 4 H), 6.85 (dd, $^{3}J_{HF}$ = 9.4 Hz, $^{3}J_{HH}$ = 8.3 Hz, 2 H), 6.47-

6.45 (m, 2 H), 4.26 (t, J = 6.9 Hz, 4 H), 3.05 (t, J = 7.0 Hz, 4 H), 1.84 (qu, J = 7.3 Hz, 4 H), 1.49-1.31 (ovrlp, 8 H), 1.26 (qu, J = 6.9 Hz, 4 H). **^{13}C NMR** (100 MHz, $CDCl_3$): δ 155.0 (d, $^{1}J_{CF}$ = 228.5 Hz), 144.9, 140.5, 125.7, 122.9, 120.5, 118.9, 115.6 (d, $^{2}J_{CF}$ = 21.9 Hz), 113.4 (d, $^{3}J_{CF}$ = 5.9 Hz), 108.7, 51.6, 43.1, 29.1, 27.3, 27.1, 27.0. **HRMS** (ESI): Calcd for $C_{42}H_{45}FN_3$ [M+H]: 610.3592; Found: 610.3608.

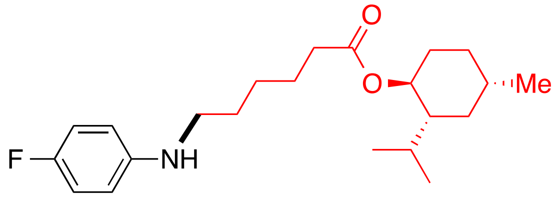

and

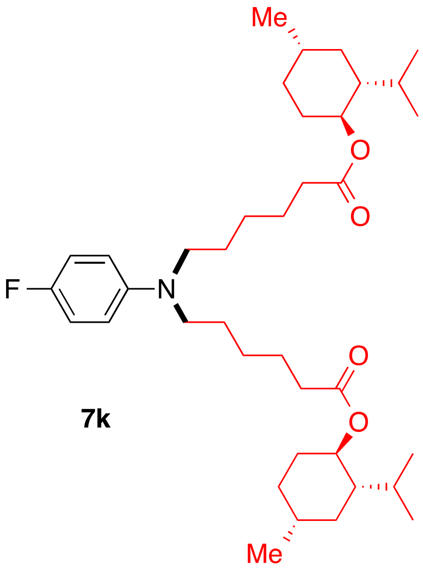

6k **7k**

(+/-)-2-Isopropyl-4-methylcyclohexyl 6-((4-Fluorophenyl)amino)hexanoate (6k) and (+/-)-2-Isopropyl-4-methylcyclohexyl 6-((4-Fluorophenyl)(6-((2-isopropyl-4-methylcyclohexyl)oxy)-6-oxohexyl)amino)hexanoate (7k). Following the general procedure B, the title compound was prepared using 1-fluoro-4-nitrobenzene (71 mg) and (+/-)-2-isopropyl-4-methylcyclohexyl 6-iodohexanoate (275 mg). The crude product was purified by preparative TLC using hexanes/EtOAc (40:1) as an eluent to afford the title compounds **6k** as off-white solid (147 mg, 81%) and **7k** as viscous colorless oil (42 mg, 14%).

Analysis of 6k: ^{1}H NMR (400 MHz, $CDCl_3$): δ 6.87 (dd, $^{3}J_{CF}$ = 9.2 Hz, $^{3}J_{HH}$ = 7.6 Hz, 2 H), 6.54-6.50 (m, 2 H), 4.68 (t, J = 11.8 Hz, 1 H), 3.73-2.67 (ovrlp, 3 H), 2.30 (t, J = 6.2 Hz, 2 H), 1.98 (d, J = 10.3 Hz, 1 H), 1.90-1.80 (m, 1 H), 1.71-1.58 (ovrlp, 6 H), 1.55-1.31 (ovrlp, 4 H), 1.10-0.83 (ovrlp, 9 H), 0.76 (d, J = 5.9 Hz, 3 H). **^{13}C NMR** (100 MHz, $CDCl_3$): δ 173.3, 155.9 (d, $^{1}J_{CF}$ = 233.1 Hz), 144.9, 115.7 (d, $^{2}J_{CF}$ = 22.1 Hz), 113.6 (d, $^{3}J_{CF}$ = 7.3 Hz), 74.2, 47.2, 44.6, 41.1, 34.7, 34.4, 31.5, 29.3, 26.8, 26.4, 25.0, 23.6, 22.2, 20.9, 16.5. **HRMS** (ESI): Calcd for $C_{22}H_{35}FNO_2$ [M+H]: 364.2646; Found: 364.2658.

Analysis of 7k: ^{1}H NMR (400 MHz, $CDCl_3$): δ 6.90 (dd, $^{3}J_{CF}$ = 9.2 Hz, $^{3}J_{HH}$ = 8.2 Hz, 2 H), 6.59-6.53 (m, 2 H), 4.68 (td, J = 10.0 Hz, J = 3.0 Hz, 2 H), 3.19 (t, J = 7.1 Hz, 4 H), 2.29 (t, J = 7.1 Hz, 4 H), 1.97 (d, J = 11.5 Hz, 2 H), 1.88-1.82 (m, 2 H), 1.72-1.61 (ovrlp, 8 H), 1.57-1.45 (ovrlp, 6 H), 1.39-1.27 (ovrlp, 6 H), 1.11-0.99 (m, 2 H), 0.94-0.83 (ovrlp, 16 H), 0.76 (d, J = 6.7 Hz, 6 H). **^{13}C NMR** (100 MHz, $CDCl_3$): δ 173.3, 155.1 (d, $^{1}J_{CF}$ = 233.1 Hz), 145.0, 115.7 (d, $^{2}J_{CF}$ = 21.7 Hz), 113.5 (d, $^{3}J_{CF}$ = 7.1 Hz), 74.2, 51.7, 47.2, 42.1, 34.8, 34.4, 31.5, 27.0, 26.8, 26.4, 25.2, 23.6, 22.2, 20.9, 16.5. **HRMS** (ESI): Calcd for $C_{38}H_{63}FNO_4$ [M+H]: 616.4736; Found: 616.4753.

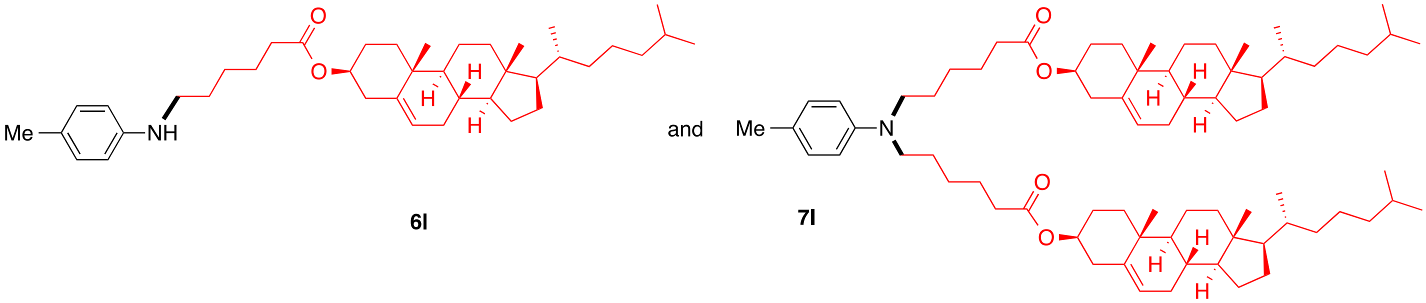

6l **7l**

(3*S*,8*S*,9*S*,10*R*,13*R*,14*S*,17*R*)-10,13-Dimethyl-17-((*R*)-6-methylheptan-2-yl)-2,3,4,7,8,9,10,11,12,13,14,15,16,17-tetradecahydro-1*H*-cyclopenta[*a*]phenanthren-3-yl 6-(*p*-Tolylamino)hexanoate (6l) and Bis((3*S*,8*S*,9*S*,10*R*,13*R*,14*S*,17*R*)-10,13-dimethyl-17-((*R*)-6-methylheptan-2-yl)-2,3,4,7,8,9,10,11,12,13,14,15,16,17-tetradecahydro-1*H*-cyclopenta[*a*]phenanthren-3-yl) 6,6'-(*p*-Tolylazanediyl)dihexanoate (7l). Following the general procedure B, the title compound was prepared using 4-nitrotoluene (69 mg) and (3*S*,8*S*,9*S*,10*R*,13*R*,14*S*,17*R*)-10,13-dimethyl-17-((*R*)-4-methylpentan-2-yl)-2,3,4,7,8,9,10,11,12,13,14,15,16,17-tetradecahydro-1*H*-cyclopenta-[*a*]phenanthren-3-yl 6-iodohexanoate (458 mg). The crude product was purified by preparative TLC (2 pieces) using hexanes/EtOAc (40:1) as an eluent to afford the title compounds **6l** as off-white solid (173 mg, 59%) and **7l** as low-melting, pale brown oil (140 mg, 26%).

Analysis of 6l: ^{1}**H NMR** (400 MHz, $CDCl_3$): δ 6.97 (d, J = 7.5 Hz, 2 H), 6.52 (d, J = 7.4 Hz, 2 H), 5.37 (s, 1 H), 4.65-4.58 (m, 1 H), 3.39 (br s, 1 H), 3.09 (t, J = 6.4 Hz, 2 H), 2.31-2.25 (ovrlp, 4 H), 2.23 (s, 3 H), 2.02-1.95 (ovrlp, 2 H), 1.86-1.77 (ovrlp, 3 H), 1.70-1.25 (ovrlp, 19 H), 1.19-0.99 (ovrlp, 11 H), 0.92 (d, J = 5.6 Hz, 3 H), 0.87 (d, J = 6.0 Hz, 6 H), 0.68 (s, 3 H). ^{13}**C NMR** (100 MHz, $CDCl_3$): δ 173.2, 146.3, 140.0, 129.8, 126.5, 122.8, 113.1, 73.9, 56.8, 56.3, 50.2, 44.3, 42.5, 39.9, 39.7, 38.3, 37.1, 36.7, 36.3, 35.9, 34.7, 32.05, 32.00, 29.4, 28.4, 28.2, 28.0, 26.8, 25.0, 24.4, 24.0, 23.0, 22.7, 21.2, 20.5, 19.5, 18.7, 12.0. **HRMS** (ESI): Calcd for $C_{40}H_{64}NO_2$ [M+H]: 590.4937; Found: 590.4940.

Analysis of 7l: ^{1}**H NMR** (400 MHz, $CDCl_3$): δ 7.00 (d, J = 7.9 Hz, 2 H), 6.55 (d, J = 7.8 Hz, 2 H), 5.37 (d, J = 3.0 Hz, 2 H), 4.65-4.57 (m, 2 H), 3.21 (t, J = 7.4 Hz, 4 H), 2.31-2.26 (ovrlp, 8 H), 2.23 (s, 3 H), 2.03-1.95 (ovrlp, 4 H), 1.88-1.78 (ovrlp, 6 H), 1.67-1.27 (ovrlp, 38 H), 1.17-0.99 (ovrlp, 22 H), 0.91 (d, J = 6.2 Hz, 6 H), 0.86 (d, J = 6.5 Hz, 12 H), 0.68 (s, 6 H). ^{13}**C NMR** (100 MHz, $CDCl_3$): δ 173.2, 146.1, 139.8, 129.9, 124.8, 122.8, 112.5, 73.9, 56.8, 56.3, 51.3, 50.2, 42.5, 39.9, 39.7, 38.7, 37.1, 36.7, 36.3, 35.9, 34.8, 32.1, 32.0, 28.4, 28.2, 28.0, 27.1, 26.8, 25.1, 24.4, 24.0, 23.0, 22.7, 21.2, 20.3, 19.5, 18.9, 12.0. **HRMS** (ESI): Calcd for $C_{73}H_{118}NO_4$ [M+H]: 1072.9061; Found: 1072.9059.

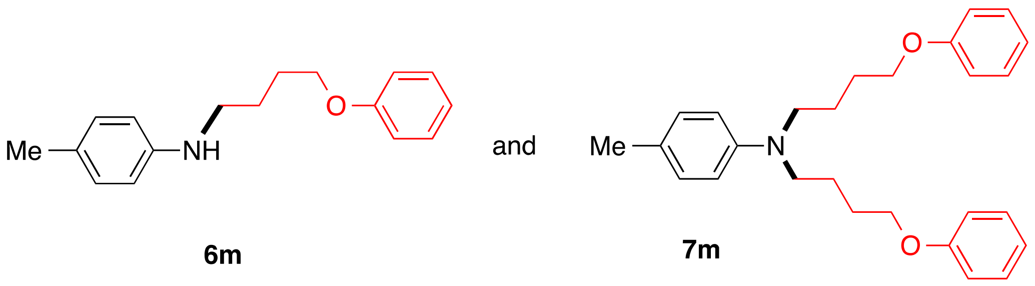

6m **7m**

4-Methyl-*N*-(4-phenoxybutyl)aniline (6m) and 4-methyl-*N*,*N*-bis(4-phenoxybutyl)aniline (7m). Following the general procedure B, the title compound was prepared using 4-nitrotoluene (69 mg) and

(4-bromobutoxy)benzene (172 mg). The crude product was purified by preparative TLC using hexanes/EtOAc (50:1) as an eluent to afford the title compounds **6m** as viscous pale-brown oil (73 mg, 57%). The dialkylated aniline, **7m**, could not be purified in pure form since it was co-isolated with **6g** (~25 mg, 12%); its formation was confirmed by HRMS analysis.

Analysis of 6m: ^{1}**H NMR** (400 MHz, $CDCl_3$): δ 7.27 (t, *J* = 7.8 Hz, 2 H), 6.99 (d, *J* = 7.8 Hz, 2 H), 6.93 (t, *J* = 7.3 Hz, 1 H), 6.89 (d, *J* = 8.1 Hz, 2 H), 6.57 (d, *J* = 7.8 Hz, 2 H), 3.99 (t, *J* = 6.2 Hz, 2 H), 3.59 (br s, 1 H), 3.18 (t, *J* = 6.5 Hz, 2 H), 2.24 (s, 3 H), 1.89 (qu, *J* = 6.8 Hz, 2 H), 1.80 (qu, *J* = 7.0 Hz, 2 H). ^{13}**C NMR** (100 MHz, $CDCl_3$): δ 159.0, 145.7, 129.9, 129.6, 127.1, 120.8, 114.6, 113.5, 67.5, 44.5, 27.1, 26.2, 20.5. **HRMS** (ESI): Calcd for $C_{17}H_{22}NO$ [M+H]: 256.1701; Found: 256.1702.

Analysis of 7m: HRMS (ESI): Calcd for $C_{27}H_{34}NO_2$ [M+H]: 404.2589; Found: 404.2590.

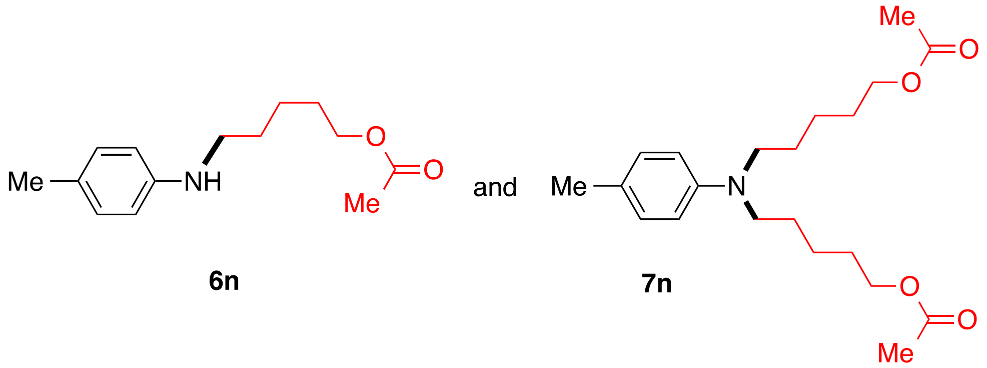

6n **7n**

5-(*p*-Tolylamino)pentyl Acetate (6n) and (*p*-tolylazanediyl)bis(pentane-5,1-diyl) diacetate (7n). Following the general procedure B, the title compound was prepared using 4-nitrotoluene (69 mg) and (4-bromobutoxy)benzene (157 mg). The crude product was purified by preparative TLC using hexanes/EtOAc (50:1) as an eluent to afford the title compounds **6n** as viscous pale-brown oil (56 mg, 47%). The dialkylated aniline, **7n**, could not be purified in pure form since it was co-isolated with **6g** (14 mg, 8%); its formation was confirmed by HRMS analysis.

Analysis of 6n: ^{1}**H NMR** (400 MHz, $CDCl_3$): δ 6.98 (d, *J* = 7.8 Hz, 2 H), 6.52 (d, *J* = 7.6 Hz, 2 H), 4.07 (t, *J* = 6.5 Hz, 2 H), 3.45 (br s, 1 H), 3.09 (t, *J* = 7.0 Hz, 2 H), 2.23 (s, 3 H), 2.04 (s, 3 H), 1.70-1.60 (ovrlp, 4 H), 1.45 (qu, *J* = 7.6 Hz, 2 H). ^{13}**C NMR** (100 MHz, $CDCl_3$): δ 171.3, 146.2, 129.8, 126.5, 113.0, 64.5, 44.3, 29.4, 28.6, 23.7, 21.0, 20.5. **HRMS** (ESI): Calcd for $C_{14}H_{22}NO_2$ [M+H]: 236.1645; Found: 236.1653.

Analysis of 7n: HRMS (ESI): Calcd for $C_{21}H_{34}NO_4$ [M+H]: 364.2482; Found: 364.2494.

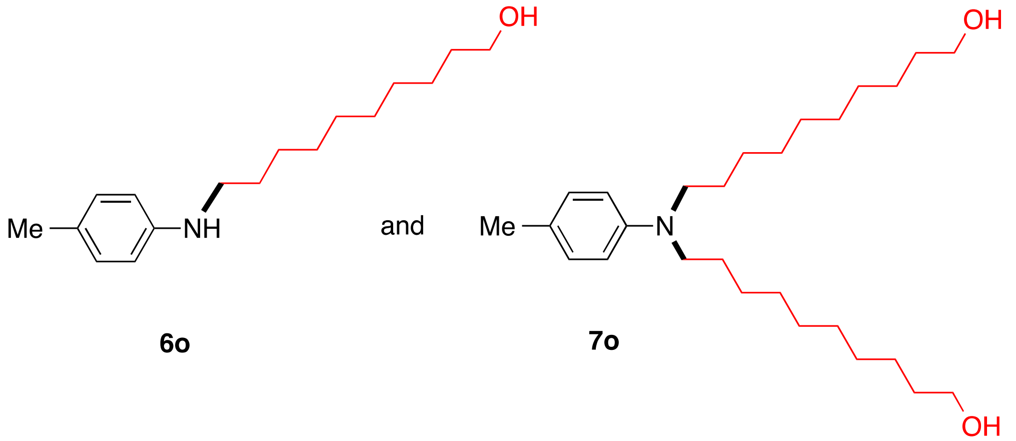

6o **7o**

10-(*p*-tolylamino)decan-1-ol (6o) and 10,10'-(*p*-Tolylazanediyl)bis(decan-1-ol) (7o). Following the general procedure B, the title compound was prepared using 4-nitrotoluene (69 mg) and 9-bromononan-1-ol (178 mg). The crude product was purified by preparative TLC using hexanes/EtOAc (50:1) as an eluent to afford the title compounds **6o** as brown solid (57 mg, 43%) and **7o** as viscous brown oil (23 mg, 11%).

Analysis of 6o: ^{1}**H NMR** (400 MHz, $CDCl_3$): δ 6.99 (d, *J* = 8.0 Hz, 2 H), 6.60 (d, *J* = 7.9 Hz, 2 H), 3.63 (t, *J* = 6.6 Hz, 2 H), 3.08 (t, *J* = 7.2 Hz, 2 H), 2.99-2.27 (br ovrlp, 2 H), 2.24 (s, 3 H), 1.64-1.50 (ovrlp, 4 H), 1.40-1.20 (ovrlp, 12 H). ^{13}**C NMR** (100 MHz, $CDCl_3$): δ 146.4, 129.8, 126.5, 113.1, 63.2, 44.5, 33.0, 29.8, 29.7, 29.6, 27.3, 25.9, 20.5. **HRMS** (ESI): Calcd for $C_{17}H_{30}NO$ [M+H]: 264.2327; Found: 264.2330.

Analysis of 7o: ^{1}**H NMR** (400 MHz, $CDCl_3$): δ 7.01 (d, *J* = 8.0 Hz, 2 H), 6.56 (d, *J* = 7.9 Hz, 2 H), 3.69-3.40 (ovrlp, 5 H), 3.20 (t, *J* = 7.5 Hz, 4 H), 2.23 (s, 3 H), 1.59-1.51 (ovrlp, 8 H), 1.37-1.23 (ovrlp, 24 H). ^{13}**C NMR** (100 MHz, $CDCl_3$): δ 146.3, 129.8, 124.5, 112.3, 63.2, 51.4, 33.0, 29.73, 29.69, 29.6, 27.41, 27.36, 25.9, 20.3. **HRMS** (ESI): Calcd for $C_{27}H_{50}NO_2$ [M+H]: 420.3836; Found: 420.3840.

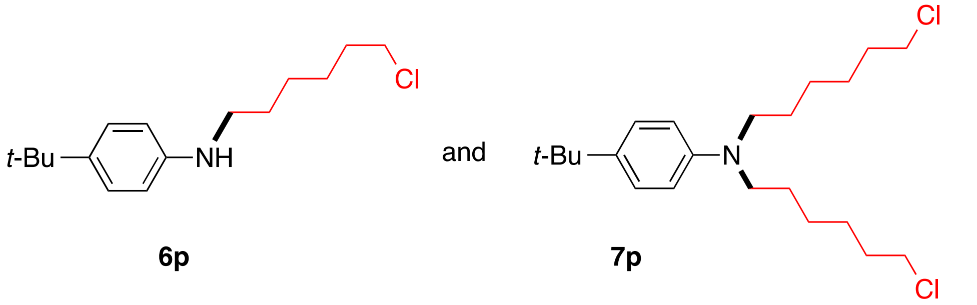

6p **7p**

4-(*tert*-Butyl)-*N*-(6-chlorohexyl)aniline (6p). Following the general procedure B, the title compound was prepared using 1-(*tert*-butyl)-4-nitrobenzene (90 mg) and 1-bromo-6-chlorohexane (150 mg). The crude product was purified by preparative TLC using hexanes as an eluent to afford the title compounds **6p** as visous brown oil (68 mg, 60%) and **7p** as viscous brown oil (14 mg, 8%).

Analysis of 6p: ^{1}**H NMR** (400 MHz, $CDCl_3$): δ 7.20 (d, *J* = 7.9 Hz, 2 H), 6.55 (d, *J* = 7.9 Hz, 2 H), 3.71-3.27 (ovrlp, 3 H), 3.09 (t, *J* = 7.0 Hz, 2 H), 1.78 (qu, *J* = 7.1 Hz, 2 H), 1.62 (qu, *J* = 7.2 Hz, 2 H), 1.50-1.40 (ovrlp, 4 H), 1.27 (s, 9 H). ^{13}**C NMR** (100 MHz, $CDCl_3$): δ 146.2, 140.0, 126.1, 112.5, 45.1, 44.2, 33.9, 32.7, 31.7, 29.7, 26.8, 26.6. **HRMS** (ESI): Calcd for $C_{16}H_{27}ClN$ [M+H]: 268.1832; Found: 268.1840.

Analysis of 7p: **^{1}H NMR** (400 MHz, $CDCl_3$): δ 7.23 (d, *J* = 7.7 Hz, 2 H), 6.59 (d, *J* = 7.8 Hz, 2 H), 3.54 (t, *J* = 6.6 Hz, 4 H), 3.23 (t, *J* = 7.6 Hz, 4 H), 1.78 (qu, *J* = 7.3 Hz, 4 H), 1.58 (qu, *J* = 7.6 Hz, 4 H), 1.48 (qu, *J* = 7.8 Hz, 4 H), 1.34 (qu, *J* = 7.7 Hz, 4 H), 1.28 (s, 9 H). **^{13}C NMR** (100 MHz, $CDCl_3$): δ 146.0, 138.1, 126.2, 111.6, 51.2, 45.2, 33.8, 32.8, 31.7, 27.4, 27.0, 26.6. **HRMS** (ESI): Calcd for $C_{22}H_{38}Cl_2N$ [M+H]: 386.2381; Found: 386.2383.

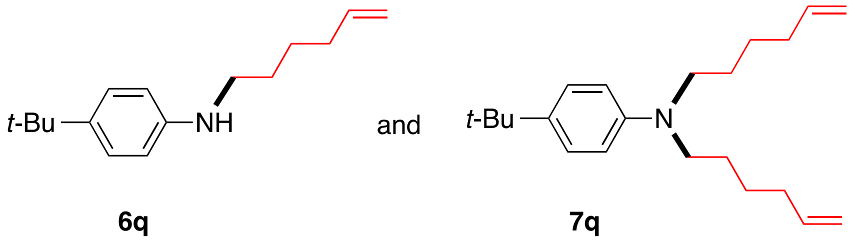

6q **7q**

4-(*tert*-Butyl)-*N*-(hex-5-en-1-yl)aniline (6q) and 4-(*tert*-Butyl)-*N*,*N*-di(hex-5-en-1-yl)aniline (7q). Following the general procedure B, the title compound was prepared using 1-(*tert*-butyl)-4-nitrobenzene (90 mg) and 6-bromo-1-hexene (122 mg). The crude product was purified by preparative TLC using hexanes as an eluent to afford the title compounds **6q** as viscous pale-brown oil (63 mg, 55%) and **7q** as viscous pale-brown oil (18 mg, 11%).

Analysis of 6q: **^{1}H NMR** (400 MHz, $CDCl_3$): δ 7.19 (d, *J* = 8.5 Hz, 2 H), 6.55 (d, *J* = 8.5 Hz, 2 H), 5.86-5.76 (m, 1 H), 5.01 (d, *J* = 16.7 Hz, 1 H), 4.96 (d, *J* = 10.4 Hz, 1 H), 3.09 (t, *J* = 7.0 Hz, 2 H), 2.09 (q, *J* = 7.2 Hz, 2 H), 1.62 (qu, *J* = 7.6 Hz, 2 H), 1.49 (qu, *J* = 7.7 Hz, 2 H), 1.27 (s, 9 H). **^{13}C NMR** (100 MHz, $CDCl_3$): δ 146.2, 140.0, 138.7, 126.1, 114.8, 112.5, 44.2, 33.9, 33.6, 31.7, 29.2, 26.6. **HRMS** (ESI): Calcd for $C_{16}H_{26}N$ [M+H]: 232.2065; Found: 232.2082.

Analysis of 7q: **^{1}H NMR** (400 MHz, $CDCl_3$): δ 7.22 (d, *J* = 8.8 Hz, 2 H), 6.59 (d, *J* = 8.8 Hz, 2 H), 5.86-5.76 (m, 2 H), 5.01 (d, *J* = 17.3 Hz, 2 H), 4.95 (d, *J* = 10.1 Hz, 2 H), 3.23 (t, *J* = 7.6 Hz, 4 H), 2.09 (q, *J* = 7.2 Hz, 4 H), 1.59 (qu, *J* = 7.8 Hz, 4 H), 1.42 (qu, *J* = 7.7 Hz, 4 H), 1.28 (s, 9 H). **^{13}C NMR** (100 MHz, $CDCl_3$): δ 146.1, 138.9, 137.9, 126.1, 114.7, 111.5, 51.1, 33.8, 31.7, 27.0, 26.7. **HRMS** (ESI): Calcd for $C_{22}H_{36}N$ [M+H]: 314.2848; Found: 314.2857.

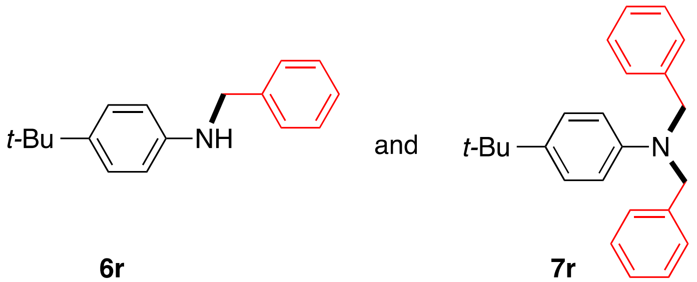

6r **7r**

***N*-Benzyl-4-(*tert*-butyl)aniline (6r)**[39] **and *N*,*N*-Dibenzyl-4-(*tert*-butyl)aniline (7r).**[40] Following the general procedure B, the title compound was prepared using 1-(*tert*-butyl)-4-nitrobenzene (90 mg) and benzyl bromide (128 mg). The crude product was purified by preparative TLC using hexanes as an eluent to afford the title compounds **6r** as pale-brown solid (60 mg, 50%) and **7r** as viscous colorless oil (13 mg, 8%).

Analysis of 6r: ^{1}**H NMR** (400 MHz, $CDCl_3$): δ 7.38-7.31 (ovrlp, 4 H), 7.26 (t, *J* = 7.6 Hz, 1 H), 7.20 (d, *J* = 8.0 Hz, 2 H), 6.59 (d, *J* = 8.4 Hz, 2 H), 4.30 (s, 2 H), 3.91 (br s, 1 H), 1.27 (s, 9 H). ^{13}**C NMR** (100 MHz, $CDCl_3$): δ 146.0, 140.5, 139.8, 128.7, 127.7, 127.3, 126.2, 112.7, 48.8, 34.0, 31.7.

Analysis of 7r: ^{1}**H NMR** (400 MHz, $CDCl_3$): δ 7.33-7.29 (ovrlp, 4 H), 7.28-7.23 (ovrlp, 6 H), 7.18 (d, *J* = 8.5 Hz, 2 H), 6.69 (d, *J* = 8.6 Hz, 2 H), 4.62 (s, 4 H), 1.26 (s, 9 H). ^{13}**C NMR** (100 MHz, $CDCl_3$): δ 147.2, 139.4, 139.1, 128.7, 126.94, 126.89, 126.1, 112.3, 54.5, 33.9, 31.7.

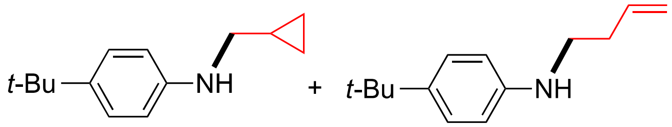

6s **6s'**

4-(*tert*-Butyl)-*N*-(cyclopropylmethyl)aniline (6s) and *N*-(but-3-en-1-yl)-4-(*tert*-butyl)aniline (6s'). Following the general procedure B, the title compounds were prepared using 1-(*tert*-butyl)-4-nitrobenzene (90 mg) and cyclopropylmethyl bromide (101 mg). The crude product was purified by preparative TLC using hexanes as an eluent to afford an inseparable mixture of the title compounds **6s** and **6s'** as pale-brown oil (27 mg, 26% total yield). The ratio of **6s** to **6s'** in the isolated products was found to be 5.7:1 as determined by ^{1}H NMR spectroscopy. **Analysis of 6s (major product):** ^{1}**H NMR** (400 MHz, $CDCl_3$): δ 7.20 (d, *J* = 8.7 Hz, 2 H), 6.57 (d, *J* = 8.6 Hz, 2 H), 3.63 (br s, 1 H), 2.94 (d, *J* = 6.9 Hz, 2 H), 1.27 (s, 9 H), 1.14-1.04 (m, 1 H), 0.56-0.51 (m, 2 H), 0.24-0.20 (m, 2 H). ^{13}**C NMR** (100 MHz, $CDCl_3$): δ 146.3, 140.2, 126.1, 112.7, 49.5, 34.0, 31.7, 11.1, 3.6. **GCMS of 6s and 6s':** $[M]^+$ = 203 was detected for both compounds which corresponds to $C_{14}H_{21}N$.

Supplementary References

1. Fulmer, G. R., Miller, A. J. M., Sherden, N. H., Gottlieb, H. E., Nudelman, A., Stoltz, B. M., Bercaw, J. E. & Goldberg, K. I. NMR chemical shifts of trace impurities: common laboratory solvents, organics, and gases in deuterated solvents relevant to the organometallic chemist. *Organometallics* **29**, 2176-2179 (2010).

2. Liou, G.-S. & Lin, H.-Y. Synthesis and electrochemical properties of novel aromatic poly(amine−amide)s with anodically highly stable yellow and blue electrochromic behaviors. *Macromolecules* **42**, 125-134 (2009).

3. Yoshii, R., Hirose, A., Tanaka, K. & Chujo, Y. Functionalization of boron diiminates with unique optical properties: multicolor tuning of crystallization-induced emission and introduction into the main chain of conjugated polymers. *J. Am. Chem. Soc.* **136**, 18131-18139 (2014).

4. P. F. Santos, L. V. Reis, I. Duarte, J. P. Serrano, P. Almeida, A. S. Oliveira, L. F. V. Ferreira, Synthesis and photochemical evaluation of iodinated squarylium cyanine dyes**.** *Helv. Chim. Acta.* **88**, 1135-1143 (2005).

5. Kawashita, Y., Nakamichi, N., Kawabata, H. & Hayashi, M. Direct and practical synthesis of 2-arylbenzoxazoles promoted by activated carbon. *Org. Lett.* **5**, 3713-3715 (2003).

6. Baell, J. B., Duggan, P. J., Forsyth, S. A., Lewis, R. J., Lok, Y. P. & Schroeder, C. I. Synthesis and biological evaluation of nonpeptide mimetics of ω-conotoxin GVIA. *Bioorg. Med. Chem.* **12**, 4025-4037 (2004).

7. Cao, J. & Perlmutter, P. Solvent-free, microwave-assisted conversion of tosylates into iodides. *Aust. J. Chem.* **67**, 1360-1361 (2014).

8. Cheung, C. W., Ren, P. & Hu, X. Mild and phosphine-free iron-catalyzed cross-coupling of nonactivated secondary alkyl halides with alkynyl Grignard reagents. *Org. Lett.* **16**, 2566-2569 (2014).

9. Ren, P., Vechorkin, O., von Allmen, K., Scopelliti, R. & Hu, X. A structure–activity study of Ni-catalyzed alkyl–alkyl Kumada coupling. Improved catalysts for coupling of secondary alkyl halides. *J. Am. Chem. Soc.* **133**, 7084-7095 (2011).

10. Cheung, C. W. & Hu, X. Stereoselective synthesis of trisubstituted alkenes through sequential iron-catalyzed reductive *anti*-carbozincation of terminal alkynes and base-metal-catalyzed Negishi cross-coupling. *Chem. Eur. J.* **21**, 18439-18444 (2015).

11. Yi, J., Lu, X., Sun, Y.-Y., Xiao, B. & Liu, L. Nickel- catalyzed Sonogashira reactions of non-activated secondary alkyl bromides and iodides. *Angew. Chem. Int. Ed.* **52**, 12409-12413 (2013).

12. Gu, X., Ndungu, J. M., Qiu, W., Ying, J., Carducci, M. D.; Wooden, H. & Hruby, V. J. Large scale enantiometric synthesis, purification, and characterization of *ω*-unsaturated amino acids via a Gly-Ni(II)-BPB-complex. *Tetrahedron* **60**, 8233-8243 (2004).

13. Chen, E. H., Saggiomo, A. J., Tanabe, K., Verma, B. L. & Nodiff, E. A. Large scale enantiomeric synthesis, purification, and characterization of ω-unsaturated amino acids via a Gly-Ni (II)-BPB-complex. *J. Med. Chem.* **20**, 1107-1109 (1977).

14. Dai, Y., F. Wu, Z. Zang, H. You, H. Gong, Ni- catalyzed reductive allylation of unactivated alkyl halides with allylic carbonates. *Chem. Eur. J.* **18**, 808-812 (2012).

15. Liu, W., Li, L., Chen, Z., Li, C.-J. A transition-metal-free Heck-type reaction between alkenes and alkyl iodides enabled by light in water. *Org. Biomol. Chem.* **13**, 6170-6174 (2015).

16. Barre, B., Gonnard, L., Campagne, R., Reymond, S., Marin, J., Ciapetti, P., Brellier, M., Guerinot, A. & Cossy, J. Iron- and cobalt-catalyzed arylation of azetidines, pyrrolidines, and piperidines with Grignard reagents. *Org. Lett.* **16**, 6160-6163 (2014).

17. Fernández-Salas, J. A., Maestro, M. C., Rodríguez-Fernández, M. M., García-Ruano, J. L. & Alonso, I. Intermolecular alkyl radical additions to enantiopure *N-tert*-butanesulfinyl aldimines. *Org. Lett.* **15**, 1658-1661 (2013).

18. Laughrey, Z. R., Upton, T. G. & Gibb, B. C. A deuterated deep-cavity cavitand confirms the importance of C–H···X–R hydrogen bonds in guest binding. *Chem. Commun.* 970-972 (2006).

19. Tehfe, M.-A., Schweizer, S., Chany, A.-C., Ysacco, C., Clement, J.-L., Gigmes, D., Morlet-Savary, F., Fouassier, J.-P., Neuburger, M., Tschamber, T., Blanchard, N. & Lalevee, J. On the synthesis, characterization and reactivity of *N*-heteroaryl–boryl radicals, a new radical class based on five-membered ring ligands. *Chem. Eur. J.* **20**, 5054-5063 (2014).

20. Yu, J., Wang, Y., Zhang, P. & Wu, J. Direct amination of phenols under metal-free conditions. *Synlett* **24**, 1448-1454 (2013).

21. Shepherd, B. D., Powell, D. R. & West, R. Synthesis, geometrical isomerism, and crystal structure of a highly hindered disilene. *Organometallics* **8**, 2664-2669 (1989).

22. Hiromitsu, M. & Yoshiharu, M. A convenient method for the preparation of highly pure t-alkyl bromides and iodides. *Bull. Chem. Soc. Jpn*, **53**, 1181-1182 (1980).

23. Kamal, A., Ramesh, G. & Laxman, N. New halogenation reagent system for one-pot conversion of alcohols into iodides and azides. *Synth. Commun.* **31**, 827-833 (2001).

24. Iwasaki, K., Wan, K. K., Oppedisano, A., Crossley, S. W. M. & Shenvi, R. A. Simple, chemoselective hydrogenation with thermodynamic stereocontrol. *J. Am. Chem. Soc.* **136**, 1300-1303 (2014).

25. Enders, D., Geibel, G. & Osborne, S. Diastereo- and enantioselective total synthesis of Stigmatellin A. *Chem. Eur. J.* **6**, 1302-1309 (2000).

26. Yasui, K., Fugami, K., Tanaka, S. & Tamaru, Y. Unsymmetrical ketone synthesis via a three-component connection reaction of organozincs, allylating agents, and carbon monoxide. *J. Org. Chem.* **60**, 1365-1380 (1995).

27. Porosa, L., Mocella, A., Wolfaardt, G. & Foucher, D. (Nano Safe Coatings Incorporated, USA). UV Cured Benzophenone Terminated Quaternary Ammonium Antimicrobials for Surfaces. US Patent 2014089680, Jun 19, 2014.

28. Gao, Y., Qi, J., Zhang, J., Kang, S., Qiao, W., Li, M., Sun, H., Zhang, J. & Ariga, K. Fabrication of both the photoactive layer and the electrode by electrochemical assembly: towards a fully solution-processable device. *Chem. Commun.* **50**, 10448-10451 (2014).

29. Bauer, G., Cheung, C. W. & Hu, X. Cross-coupling of nonactivated primary and secondary alkyl halides with aryl Grignard reagents catalyzed by chiral iron pincer complexes. *Synthesis* **47**, 1726-1732 (2015).

30. Hollmann, D., Bähn, S., Tillack, A. & Beller, M. A general ruthenium- catalyzed synthesis of aromatic amines. *Angew. Chem. Int. Ed.* **46**, 8291-8294 (2007).

31. Zhao, Y., Lin, M., Chen, Z., Pei, H., Li, Y., Chen, Y., Wang, X., Li, L., Cao, Y., Zhang, Y. & Li, W. Titanium and zirconium amido complexes supported by imidazole-containing ligands: syntheses, characterization and catalytic activities. *RSC Adv.* **2**, 144-150 (2012).

32. Khedkar, V., Tillack, A. & Beller, M. A dramatic effect of aryloxo ligands on the titanium-catalyzed hydroamination of alkynes. *Org. Lett.* **5,** 4767-4770 (2003).

33. Urgaonkar, S., Nagarajan, M. & Verkade, J. G. P(*i*-$BuNCH_2CH_2)_3N$: an effective ligand in the palladium-catalyzed amination of aryl bromides and iodides. *J. Org. Chem.* **68**, 452-459 (2003).

34. Lei, Q., Wei, Y., Talwar, D., Wang, C., Xue, D. & Xiao, J. Fast reductive amination by transfer hydrogenation "on water". *Chem. Eur. J.* **19**, 4021-4029 (2013).

35. Urbina, J. M., Cortés, J. C. G., Palma, A., López, S. N., Zacchino, S. A., Enriz, R. D., Ribas, J. C. & Kouznetzov, V. V. Inhibitors of the fungal cell wall. Synthesis of 4-aryl-4-*N*-arylamine-1-butenes and related compounds with inhibitory activities on β(1–3) glucan and chitin synthases. *Bioorg. Med. Chem.* **8**, 691-698 (2000).

36. Chen, Z., Zeng, H., Girard, S. A., Wang, F., Chen, N. & Li, C.-J. Formal direct cross- coupling of phenols with amines. *Angew. Chem. Int. Ed.* **54**, 14487-14491 (2015).

37. Feng, C., Liu, Y., Peng, S., Shuai, Q., Deng, G. & Li, C.-J. Ruthenium-catalyzed tertiary amine formation from nitroarenes and alcohols. *Org. Lett.* **12**, 4888-4891 (2010).

38. Cui, X., Deng, Y. & Shi, F. Reductive *N*-alkylation of nitro compounds to *N*-alkyl and *N*,*N*-dialkyl amines with glycerol as the hydrogen source. *ACS Catal.* **3**, 808−811 (2013).

39. Shimizu, K., Imaiida, N., Kon, K., Siddiki, S. M. A. H. & Satsuma, A. Heterogeneous Ni catalysts for *N*-alkylation of amines with alcohols. *ACS Catal.* **3**, 998-1005 (2013).

40. So, C. M., Zhou, Z., Lau, C. P. & Kwong, F. Y. Palladium-catalyzed amination of aryl mesylates. *Angew. Chem. Int. Ed.* **47**, 6402-6406 (2008).
